# Development and *In Vivo* Evaluation of Small-Molecule Ligands for Positron Emission Tomography of Immune Checkpoint Modulation Targeting Programmed Cell Death 1 Ligand 1

**DOI:** 10.1021/acs.jmedchem.3c02342

**Published:** 2024-03-05

**Authors:** Karsten Bamminger, Verena Pichler, Chrysoula Vraka, Tanja Limberger, Boryana Moneva, Katharina Pallitsch, Barbara Lieder, Anna Sophia Zacher, Stefanie Ponti, Katarína Benčurová, Jiaye Yang, Sandra Högler, Petra Kodajova, Lukas Kenner, Marcus Hacker, Wolfgang Wadsak

**Affiliations:** † CBmed GmbH - Center for Biomarker Research in Medicine, 8010 Graz, Austria; ‡ Department of Biomedical Imaging and Image-guided Therapy, Division of Nuclear Medicine, Medical University of Vienna, 1090 Vienna, Austria; § Department of Pharmaceutical Sciences, Division of Pharmaceutical Chemistry, University of Vienna, 1090 Vienna, Austria; ∥ Institute of Clinical Pathology, Medical University of Vienna, 1090 Vienna, Austria; ⊥ Institute of Organic Chemistry, University of Vienna, 1090 Vienna, Austria; # Institute of Physiological Chemistry, University of Vienna, 1090 Vienna, Austria; ∇ Institute of Clinical Nutrition, University of Hohenheim, 70599 Stuttgart, Germany; ○ Unit of Laboratory Animal Pathology, University of Veterinary Medicine Vienna, 1210 Vienna, Austria

## Abstract

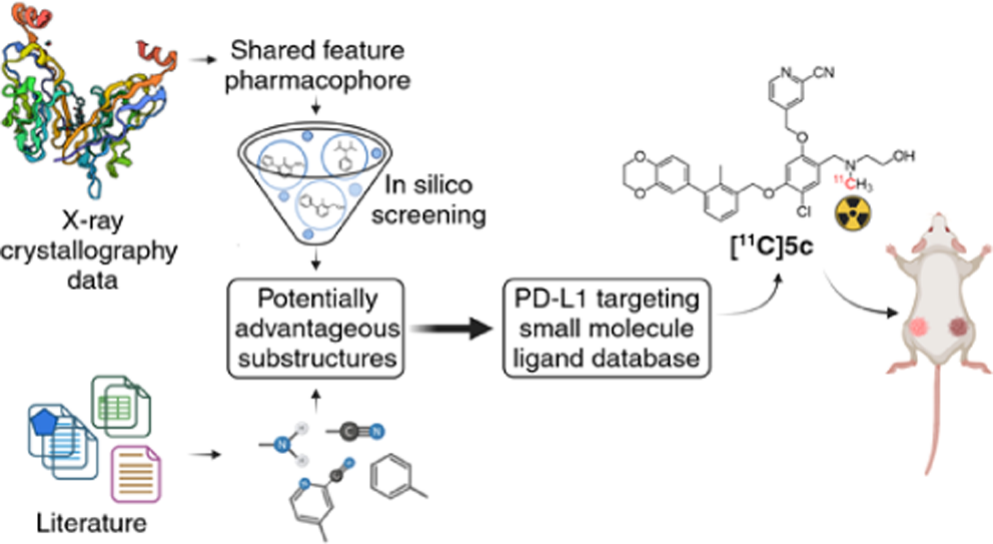

A substantial portion of patients do not benefit from programmed cell death protein 1/programmed cell death 1 ligand 1 (PD-1/PD-L1) checkpoint inhibition therapies, necessitating a deeper understanding of predictive biomarkers. Immunohistochemistry (IHC) has played a pivotal role in assessing PD-L1 expression, but small-molecule positron emission tomography (PET) tracers could offer a promising avenue to address IHC-associated limitations, i.e., invasiveness and PD-L1 expression heterogeneity. PET tracers would allow for improved quantification of PD-L1 through noninvasive whole-body imaging, thereby enhancing patient stratification. Here, a large series of PD-L1 targeting small molecules were synthesized, leveraging advantageous substructures to achieve exceptionally low nanomolar affinities. Compound **5c** emerged as a promising candidate (IC_50_ = 10.2 nM) and underwent successful carbon-11 radiolabeling. However, a lack of *in vivo* tracer uptake in xenografts and notable accumulation in excretory organs was observed, underscoring the challenges encountered in small-molecule PD-L1 PET tracer development. The findings, including structure–activity relationships and *in vivo* biodistribution data, stand to illuminate the path forward for refining small-molecule PD-L1 PET tracers.

## Introduction

Cancer immunotherapy has transformed the landscape of cancer treatment over the past decade. Among the remarkable advances in this field, immune checkpoint therapy, particularly the blocking of programmed cell death 1 ligand 1 (PD-L1) and its receptor, programmed cell death protein 1 (PD-1), has emerged as a pivotal strategy. This approach harnesses the power of the immune system to target and eliminate cancer cells, leading to unprecedented clinical responses in a subset of patients.^1^ However, the clinical success of PD-L1 checkpoint therapy has unveiled complex challenges, including response heterogeneity, resistance, and the need for accurate patient stratification. In this context, positron emission tomography (PET) radiotracers, particularly radiolabeled antibodies, have emerged as promising tools to address these challenges by enabling longitudinal, noninvasive, real-time assessment of PD-L1 expression and immune response dynamics.^2,3^ One of the most promising applications of PD-L1 PET radiotracers is patient stratification. By identifying patients with high PD-L1 expression and an active antitumor immune response, PET imaging can guide the selection of individuals who are most likely to respond to PD-L1 checkpoint inhibitor therapy.^2^ This personalized approach holds the potential to minimize treatment-related adverse events (fatigue, pruritus, diarrhea, endocrine dysfunction, pneumonitis) and optimize therapeutic outcomes.

Human studies of radiolabeled anti-PD-1/PD-L1 antibodies, e.g., [^89^Zr]Zr-atezolizumab, [^89^Zr]Zr-durvalumab and [^89^Zr]Zr-pembrolizumab, demonstrated that radiotracer tumor uptake was higher in patients with a response to immune checkpoint therapy.^2,4,5^ Additionally, tumor uptake correlated better with clinical response than immunohistochemistry or RNA-sequencing,^2,4^ and substantial intra- and intertumoral uptake heterogeneity was observed, reflecting the heterogeneity of PD-L1 expression. Recently, the peptide-based radiotracer [^68^Ga]Ga-NOTA-WL12 was investigated in a first-in-human study indicating its potential benefits for clinical immunotherapy.^6^ Nonetheless, ongoing efforts are focused on the potential development of novel and enhanced antibody-, antibody-fragment- and peptide-based PD-L1 PET imaging probes.^7−16^

Small-molecule PET tracers targeting PD-L1 represent a promising avenue for addressing critical challenges associated with antibody- and peptide-based radiotracers. These small molecules offer potential advantages, including expedited pharmacokinetics, cost-effectiveness, increased stability, and enhanced tissue and tumor penetration, facilitating comprehensive evaluation of PD-L1 expression within the heterogeneous tumor microenvironment. Significant efforts have been invested in advancing small molecules for therapeutic applications despite the intricate nature of the target. PD-L1 lacks a dedicated binding pocket for small molecules and its binding mode with the endogenous receptor PD-1 is characterized by a large and flat protein–protein interaction interface.^17^ This characteristic makes it challenging to effectively target PD-L1 with small molecules. Among these compounds, the biphenyl substructure emerged as a prominent and recurrent moiety found in potent inhibitors patented by companies and institutes in the pharmaceutical field, e.g., Bristol Myers Squibb (BMS), Polaris Pharmaceuticals, Incyte Corporation and Institute of Materia Medica.^18^ These compounds exhibited selectivity for human PD-L1 (*h*PD-L1) over murine PD-L1 (*m*PD-L1)^19,20^ and induced dimerization of PD-L1 through binding modes that overlap with anti-PD-L1 antibodies, e.g., atezolizumab and durvalumab.^21,22^ Nevertheless, the development of nonpeptidic small-molecule PD-L1 PET tracers is still in its early stages with limited published research and constrained achievements to date (Table 1). The observed uptakes in PD-L1 expressing (PD-L1^+^) tumor xenograft over controls were modest, with increases of 2.2-fold, 2.9-fold, 3.0-fold, ∼1.4-fold, and 1.9-fold, resulting in uptake values of 1.2% ID/g, 4.0% ID/g, 3.5% ID/mL, ≤ 5% ID/g, and 4.2% ID/g for radiotracers [^18^F]LN,^23^ [^18^F]LG-1,^24^ [^18^F]LP-F,^25^ [^64^Cu]Cu-43b,^26^ and [^68^Ga]BMSH,^27^ respectively. [^18^F]FDHPA^28^ and [^18^F]LGSu-1^29^ demonstrated ≤1% ID/g and 3.3% ID/mL uptake, respectively; however, control xenografts were not available for comparison.

**Table 1:** Overview of Reported Small-Molecule PET Radiotracers Targeting PD-L1 and the Corresponding Results of *In Vivo* Investigations, Specifically Tumor Uptake

tracer	tumor model	PD-L1^+^ tumor uptake	PD-L1^–^ tumor uptake	uptake ratio	reference
[^18^F**]**LN	A375	1.96 ± 0.27% ID/g	0.89 ± 0.31% ID/g	2.2	(23)
[^18^F]LG-1	A375	3.98 ± 0.21% ID/g	1.38 ± 0.34% ID/g	2.9	(24)
[^18^F]LP-F	A375	3.53 ± 0.46% ID/mL	∼1.19% ID/mL	3.0	(25)
[^64^Cu]Cu-43b	PC3	∼4.8% ID/g	∼3.5% ID/g	∼1.4	(26)
[^68^Ga]BMSH	A549	4.22 ± 0.65% ID/g	2.23 ± 0.41% ID/g	1.9	(27)
[^18^F]FDHPA	MDA-MB-23	≤1% ID/g	N/A	N/A	(28)
[^18^F]LGSu-1	B16–F10	3.33 ± 0.24% ID/mL	N/A	N/A	(29)

Recent research explored the potential of the 4-fluorophenylthiophene-3-carbonitrile moiety as an alternative to the biphenyl core substructure. *Ex vivo* tissue section autoradiography experiments showed that this radiotracer (2-((4-(aminomethyl)benzyl)oxy)-4-(4-[^18^F]fluorophenyl)thiophene-3-carbonitrile) exhibited a 1.4-fold higher uptake in PD-L1^+^ compared to PD-L1^–^ H358 tumors (lung adenocarcinoma). This difference was not observed in PD-L1^±^ ES2 tumors (ovarian carcinoma), in contrast to a radiolabeled biphenyl-based BMS-1166 derivative.^30^ We previously demonstrated that commercially accessible biphenyl-based lead structures and derivatives designed via a ligand-based drug design approach exhibit suboptimal binding affinity. Nonetheless, this investigation provided valuable insights into the effects of structural modifications.^31^

**Figure 1:**
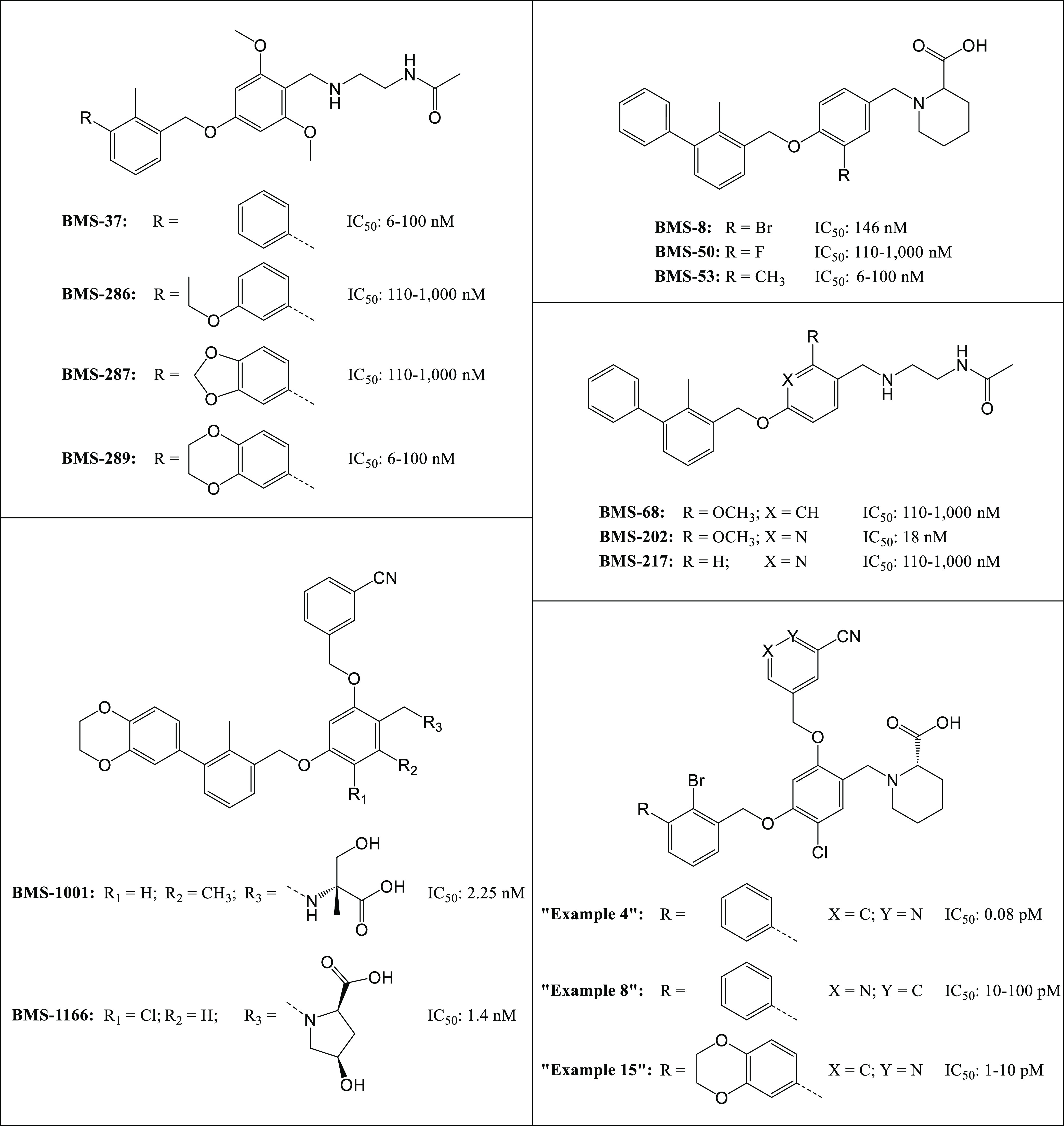
Overview of exemplary structures from previously reported biphenyl-based ligands,^32−34^ featuring potentially advantageous substructures that serve as the foundation for the development of our compounds. A comprehensive patent review has been published before.^18^

Our primary objective was to design and develop small molecules for noninvasive PET imaging targeting PD-L1, with the overarching goal of enhancing patient stratification within the framework of personalized medicine. This endeavor was rooted in the identification of promising substructures through rigorous *in silico* investigations and an extensive review of existing literature^18,32−34^ (Figure 1). *De novo* synthesized compounds underwent extensive *in vitro* evaluations, with a particular focus on assessing their binding affinity toward PD-L1. Viable candidates were subjected to carbon-11 radiolabeling processes, culminating in the selection of the most promising candidate for further investigations, both *in vitro* and *in vivo*.

## Results and Discussion

### Pharmacophore-Based Virtual Screening

**Figure 2:**
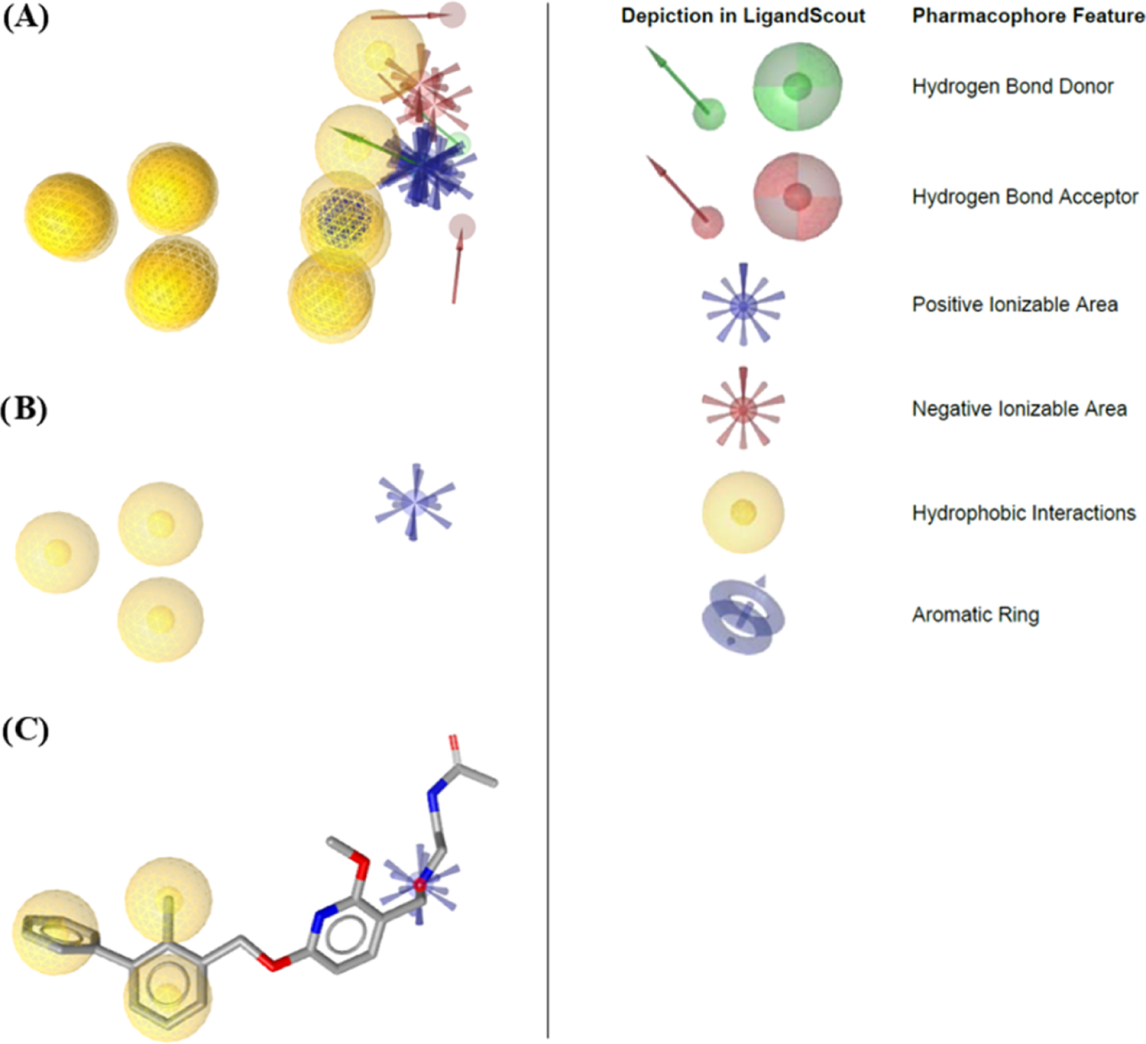
(A) Superposition of PDB 5J89, 5J8O, 5N2D, 5N2F, 6R3K, and 6NM8 pharmacophores. (B) Generated shared feature pharmacophore. (C) Shared feature pharmacophore model aligned with the PDB 5J89 ligand BMS-202 (gray sticks). Pharmacophore feature definitions are represented according to the LigandScout program.

A consensus feature-based (“shared feature”) pharmacophore model was derived from six distinct crystallographic data sets (PDB: 5J89, 5J8O, 5N2D, 5N2F, 6R3K, and 6NM8) using small-molecule ligands that interact with PD-L1 (Figure S1). This model encompassed three hydrophobic features and a positive ionizable area (Figure 2B). Hydrophobic features represent the 2-methylbiphenyl core substructure situated at the base of the hydrophobic pocket formed within the interplay of two PD-L1 monomers.^21,31^

To identify novel potential structures substituting the 2-methylbiphenyl moiety, the generated pharmacophore model underwent screening against a data set of 34,207 low molecular weight compounds (≤200 g/mol) including bioactive molecules with drug-like properties, marking the positive ionizable area as an optional feature. A total of 2695 *in silico* hits (7.9%) were acquired, exhibiting Pharmacophore-Fit Scores spanning from 34.73 to 38.89. Upon transposition to the PDB entry 5J89, Binding Affinity Scores were computed, encompassing a range from −34.55 to 26.44. These hits were then ranked based on their scores and were allocated up to 10 points per score. Top 10 hits are represented in Table 2. All hits passed the Pan Assay Interference Compounds (PAINS) test.

**Table 2:**
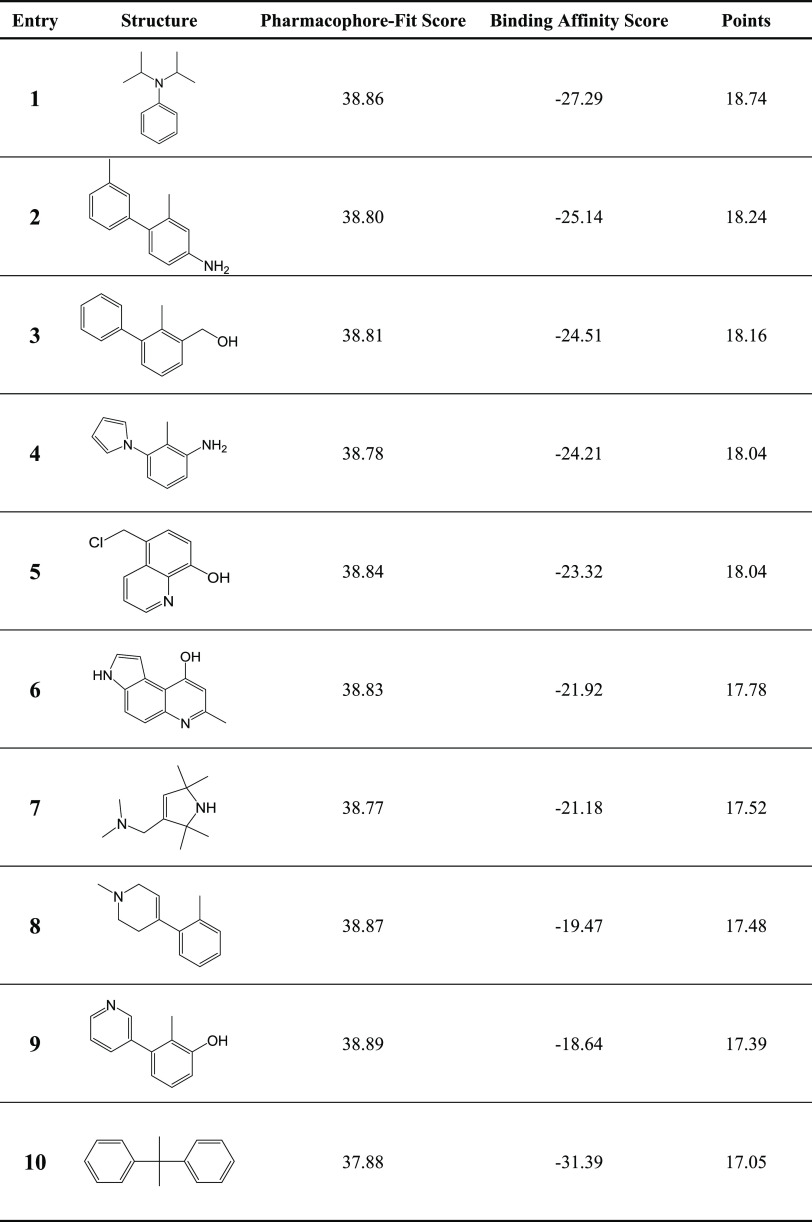
Top 10 Hit Structures of the Pharmacophore Screening Accounting to Their Performance According to the Pharmacophore-Fit Score and Binding Affinity Score Expressed as Overall Points (max. 20)^a^

a Higher Pharmacophore-Fit Score and lower Binding Affinity Score indicate better pharmacophore fitting and affinity, respectively.

The phenyl moiety and its bioisosteric counterparts emerged as recurring substructures, with the 2-methylbiphenyl structure (entry 3) and modifications being frequently represented among the top hits. It is worth mentioning that the tertiary amine in entry 1 (calculated p*K*_a_: 7.34) would undergo protonation within the acidic tumor microenvironment (pH 6.4–7^35^), which might have adverse effects on the binding mode. In the case of entry 2, the presence of an additional methyl group at the distal phenyl ring compared to entry 3 implies the applicability of specific modifications. However, it has been shown that methoxy, ethoxy, and methylenedioxy substituents exhibit detrimental effects, while compounds containing an ethylenedioxy group displayed comparable or enhanced binding affinities.^32^ Interestingly, pyrrole (entry 4) was identified as a superior bioisosteric replacement for the distal phenyl ring compared to pyridine (entry 9), underscoring the significance of the heteroatom’s position and basicity.

**Scheme 1:**
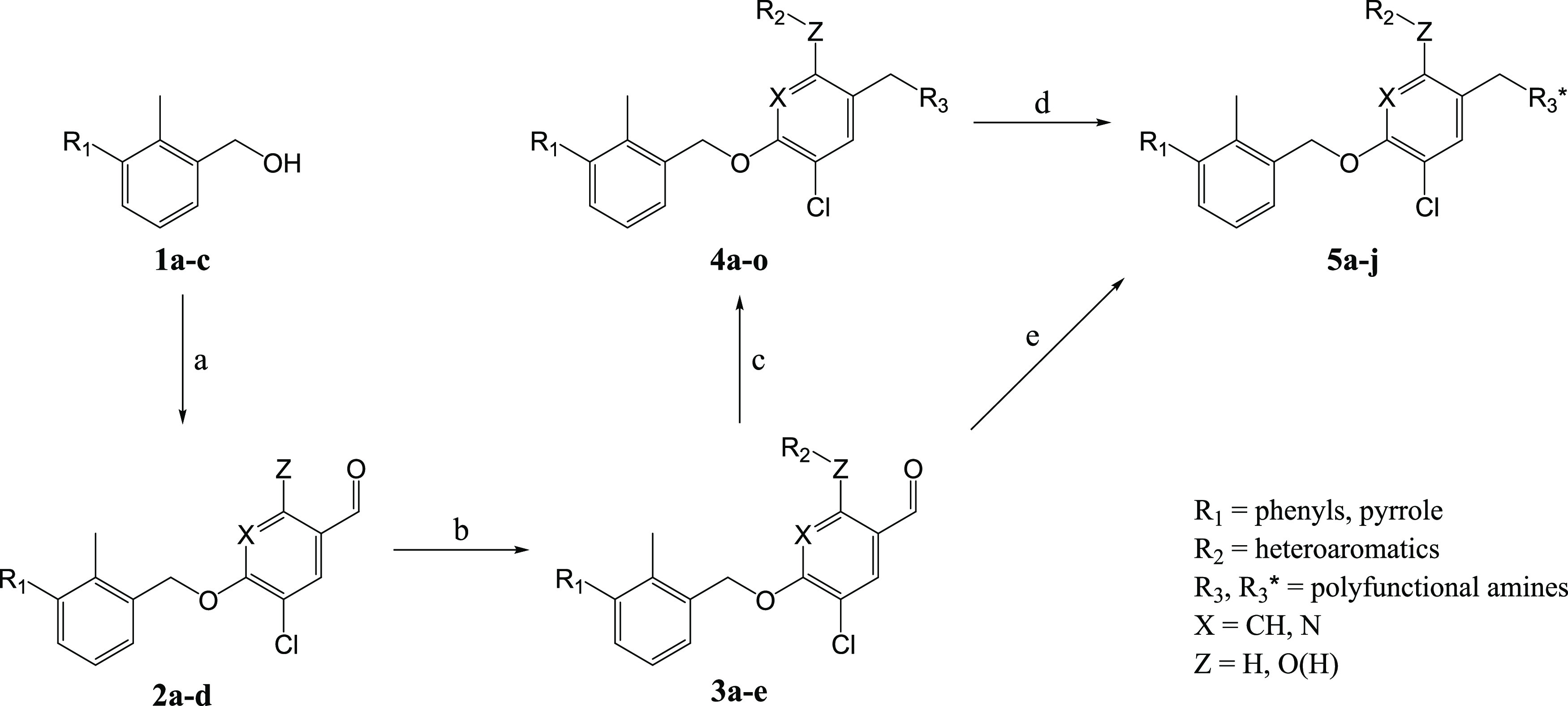
Synthesis Scheme of Intermediates **1**, **2**, and **3**, Intermediates/Precursors **4**, as well as Methylated, Carbonylated, or Fluorinated Final Products **5** Reagents and conditions: (a) DEAD, PPh_3_, DCM or THF, 0 °C → RT, 24–52%; (b) appropriate halide, Cs_2_CO_3_, DMF, RT, 68–83%; (c) appropriate amine, NaBH(OAc)_3_ or NaBH_3_CN, DCM or DMF/MeOH, RT, 14–64%; (d) 2-fluoroethyl *p*-toluenesulfonate, DMSO, 50–100 °C, 40–52%; (e) appropriate amine, NaBH(OAc)_3_ or NaBH_3_CN, DCM or DMF/MeOH, RT, 12–17%. DEAD = diethyl azodicarboxylate. DCM = dichloromethane. THF = tetrahydrofuran. RT = room temperature. DMF = *N*,*N*-dimethylformamide. MeOH = methanol. DMSO = dimethylsulfoxide.

In summary, our pharmacophore-based virtual screening investigations did not uncover any novel structures capable of enhancing pharmacophore fitting and binding affinity beyond the 2-methylbiphenyl structure (entry 3). Anyway, it is worth mentioning that pyrrole may serve as a potential bioisosteric replacement with reduced hydrophilicity. Following this observation, we synthesized compounds with pyrrole substitutions, replacing the 2-methylbiphenyl moiety at R_1_ (Scheme 1) in the subsequent step.

### Multistep *De Novo* Synthesis of Ligands

Novel ligands were synthesized by incorporating potentially beneficial substructures identified through pharmacophore-based virtual screening (*vide supra*) and extensive literature research,^32−34^ along with previously unexplored molecular entities and bioisosteric replacements. The multistep synthetic pathway is presented in Scheme 1.

Compounds **1** represent the main pharmacophore deemed essential for PD-L1 binding as described before.^21^ These compounds are sourced either from commercial suppliers (**1a**) or synthesized through Suzuki coupling (**1b**) of a boronic acid and aryl halide, or reduction (**1c**) of the respective carboxylic acid, resulting in good yields of 64 and 74% (Table 3). Intermediates **1** were subsequently joined with polysubstituted (hetero)aromatic molecules, that bear functional groups suitable for subsequent modifications, via Mitsunobu reactions delivering yields within the range of 24–52% (**2a**–**d**). (Un)substituted (hetero)cyclic aromatic molecules were added through nucleophilic substitutions under basic condition in good yields of 68–83% (**3a**–**e**). Intermediates **3** served as precursors for intermediates **4b**–**o** or final compounds **5a**–**d**, **5f**, and **5i** in reductive amination reactions using NaBH(OAc)_3_ or NaBH_3_CN as reducing agents achieving yields of 12–64%. Compound **4a**, which lacks R_2_, was synthesized from **2a** through reductive amination with 41% yield. Intermediates **4** were used as starting material for nucleophilic substitution reactions (i.e., fluoroethylation and carbamylation) giving final compounds **5e,g,h,j** in 40–52% yields, and as precursors for radiolabeling (i.e., ^11^C-methylation). All intermediates and products passed the PAINS test.

**Table 3:**
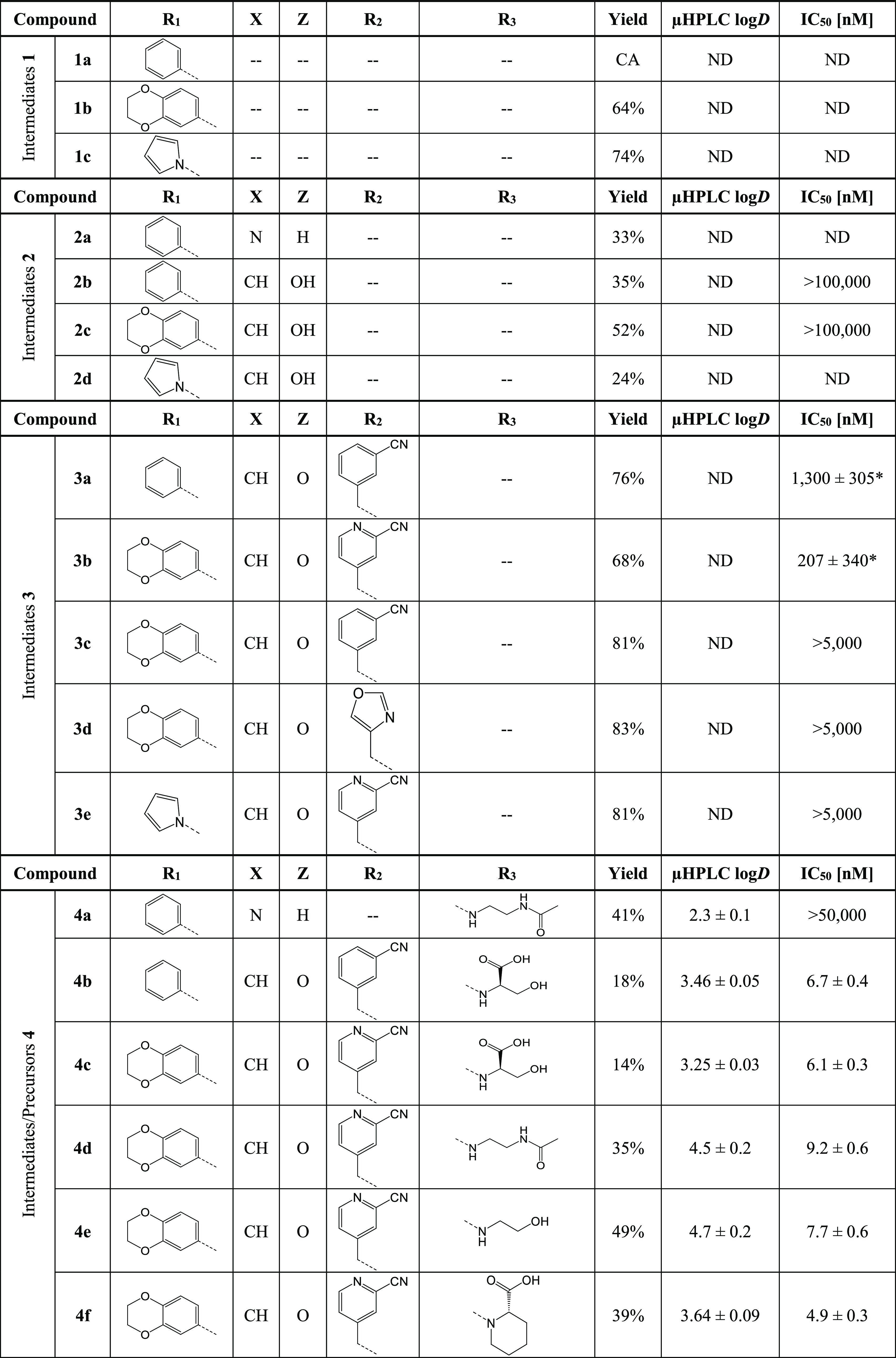

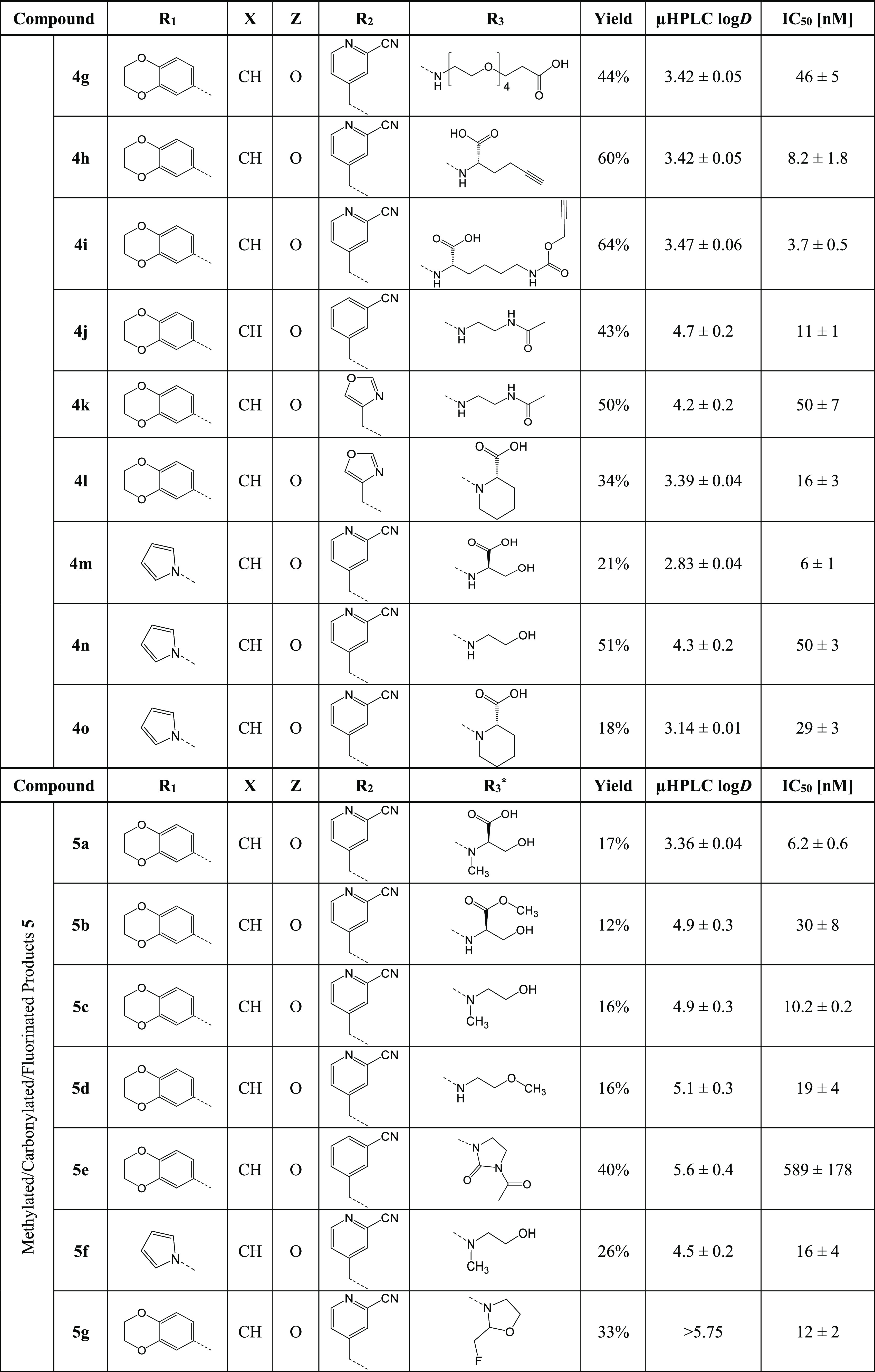

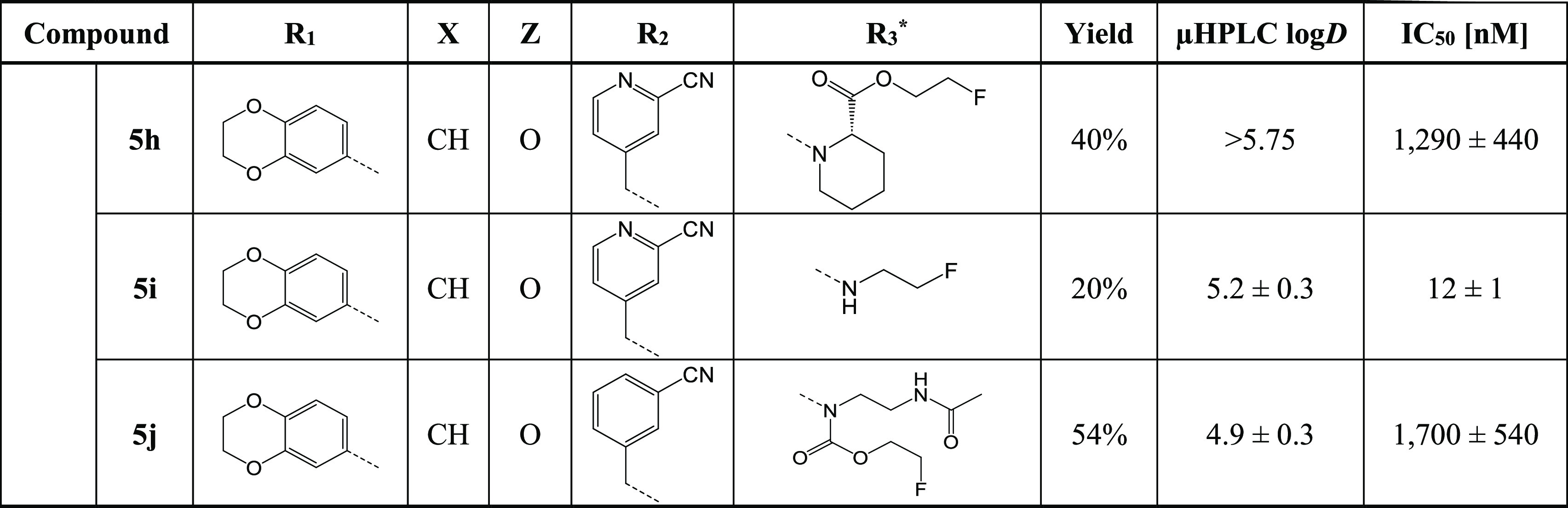
Overview of Molecular Structures and Yields of Intermediates **1a**–**c**, **2a**–**d**, **3a**–**e**, and **4a**–**o**, and Final Compounds **5a**–**j**, as well as Measured Lipophilicity (μHPLC log*D*) and *h*PD-L1 Binding Affinities (HTRF IC_50_)^a^

a CA = commercially available. ND = not determined. * No full dose–response curves were observed, and values are represented as relative IC_50_.

In summary, the synthetic pathways involving Mitsunobu reactions, nucleophilic substitutions, and reductive aminations yielded the desired final products effectively. This approach resulted in the generation of 37 compounds that in the further course facilitated the exploration of structure–activity relationships. Furthermore, it provided a collection of six methylated or carbonylated and four fluorinated products, which were used for subsequent *in vitro* evaluations and served as essential reference compounds for radiolabeling endeavors.

### Structure–Activity Relationships

The lipophilicity of the compounds was evaluated using an established HPLC method^36,37^ as the logarithm of the partition coefficient at pH 7.4 (μHPLC log*D*_pH7.4_) (Table 3). Obtained lipophilicity data was compared with calculated parameters such as the clog*P*, clog*D*_pH7.4_, and the topological polar surface area (tPSA) (Table S2). The measured μHPLC log*D* values for compounds **4a**–**o** and **5a**–**j** fell within the range of 2.33–5.6 except for compounds **5g** and **5h** with log*D* values >5.75, indicating their overall lipophilic nature.

Our measurements clearly showcased how structural modifications affected lipophilicity (μHPLC log *D*). The introduction of pyrrole at R_1_ reduced lipophilicity compared to the distal 1,4-benzodioxanyl moiety, and the introduction of one or more heteroatoms at R_2_ in the form of picolinonitrile or oxazole reduced lipophilicity compared to benzonitrile. R_3_ and R_3_* significantly influenced the lipophilic character of our compounds. Indeed, fluoroethylation and methylation increased lipophilicity, although *O*-methylation resulted in a more significant increase in lipophilicity compared to *N*-methylation.

Both calculated parameters, clog*P* and clog*D*, successfully predicted an increase in lipophilicity based on chemical modifications such as methylation and fluoroethylation, and these calculations exhibited a very strong correlation with measured μHPLC log*D* values (ρ = 0.89, *p* < 0.001, *n* = 25 and ρ = 0.93, *p* < 0.001, *n* = 25, respectively), although calculated values tended to overestimate the lipophilic characteristics of our compounds. There was only a weak inverse correlation between tPSA and μHPLC log*D* (ρ = −0.32, *p* = 0.12, *n* = 25) (Figure S2).

Compound’s binding affinity toward *h*PD-L1 was determined through a homogeneous time-resolved fluorescence (HTRF) assay (Table 3). The high-affinity anti-PD-L1 antibody atezolizumab was used for reference. PD-L1 binding affinities (IC_50_ values) ranged from >100,000 nM for intermediates **2a**–**d**, 207 nM to >5000 nM for intermediates **3a**–**e**, 3.72 to >50,000 nM for intermediates **4a**–**o**, and 6.18–1700 nM for products **5a**–**j**.

Our smallest tested compounds (intermediates **2b**,**c**) did not exhibit binding to PD-L1 in the competitive HTRF assay. First observations of PD-L1 binding proficiency were made with intermediates **3** upon the introduction of R_2_. This is in contrast to the findings by Skalniak et al.,^38^ where ^1^H–^15^N HMQC NMR measurements have elucidated that the minimal functional fragment capable of engaging with PD-L1 corresponds to the biphenyl structure, mirroring our **1b** intermediate. Significantly enhanced binding affinities were achieved with the incorporation of polar residues R_3_, surpassing, in certain instances, the antibody atezolizumab (IC_50_ = 4.1 nM, Table S2). Compounds **4b**–**o** encompassing all three residues (R_1_, R_2_, and R_3_) demonstrated exceptional IC_50_ values, spanning from 3.7 to 50 nM. Furthermore, final products **5**, featuring chemically modified R_3_ residues (R_3_*), also displayed remarkable PD-L1 binding affinities in the low nanomolar range, but not superior when compared to compounds **4**.

**Figure 3:**
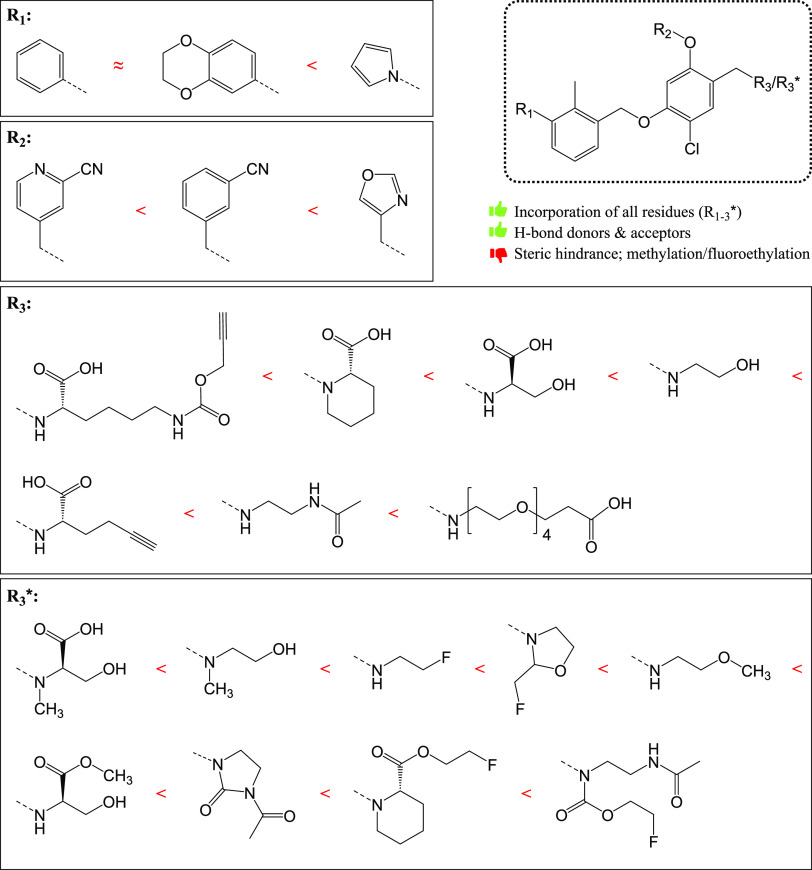
Comprehensive overview of the identified structure–activity relationships concerning residues R_1–3_*. The moieties are systematically arranged based on ascending HTRF IC_50_ values.

The observed IC_50_ values were profoundly influenced by the structural characteristics of the compounds, allowing for the deduction of structure–activity relationships: an adequate molecular size (>500 g/mol) was needed for sufficient molecular interactions to compete with the endogenous receptor PD-1 for PD-L1 binding in the competitive HTRF assay, as demonstrated by intermediates **2a**–**d**, **3a**–**e** and **4a**, which lack R_2_ and/or R_3_ residues. Introduction of pyrrole at R_1_ reduced affinity compared to the distal 1,4-benzodioxanyl moiety 1.03-fold (**4c** vs **4m**), 6.40-fold (**4e** vs **4n**), 5.99-fold (**4f** vs **4o**), and 1.61-fold (**5c** vs **5f**) (Figure 3). Nevertheless, the reduction in lipophilicity and preservation of nanomolar affinities suggests that pyrrole is a viable option for bioisosteric replacement. The influence of R_2_ on affinity can be ranked by ascending IC_50_ values: picolinonitrile (**4d**,**f**) < benzonitrile (**4j**) < oxazole (**4k**,**l**). Hence, picolinonitrile was a prevalent recurring motif in our compounds. Both R_3_ and R_3_* had a large impact on affinity. The influence of R_3_ on affinity can be ranked by ascending IC_50_ values: *N*-ε-propargyloxycarbonyl-l-lysine (**4i**) < (*S*)-piperidine-2-carboxylic acid (**4f**) < d-serine (**4c**) < 2-aminoethan-1-ol (**4e**) < (*S*)-2-aminohex-5-ynoic acid (**4h**) < *N*-(2-aminoethyl)acetamide (**4d**) < NH_2_–PEG_4_-COOH (**4g**). Similarly, the influence of R_3_* on affinity ranked by ascending IC_50_ values: (*R*)-3-hydroxy-2-(methylamino)propanoic acid (**5a**) < 2-(methylamino)ethanol (**5c**) < 2-fluoroethylamine (**5i**) < 2-(fluoromethyl)oxazolidine (**5g**) < 2-methoxyethylamine (**5d**) < d-serine methyl ester (**5b**) < 1-acetylimidazolidin-2-one (**5e**) < 2-fluoroethyl (*S*)-piperidine-2-carboxylate (**5h**) < 2-fluoroethyl (2-acetamidoethyl)carbamate (**5j**). Methylation at R_3_* generally leads to a decrease in affinity 1.02-fold (**4c** vs **5a**), 4.98-fold (**4c** vs **5b**), 1.32-fold (**4e** vs **5c**), and 2.42-fold (**4e** vs **5d**), with *O*-methylation having a more adverse effect than *N*-methylation. However, there was one exception in which methylation improved binding affinity (**4o** vs **5f**). Additionally, fluoroethylation and fluoroethyl carbamylation at R_3_* decreased affinity 265-fold (**4f** vs **5h**) and 160-fold (**4j** vs **5j**).

**Figure 4:**
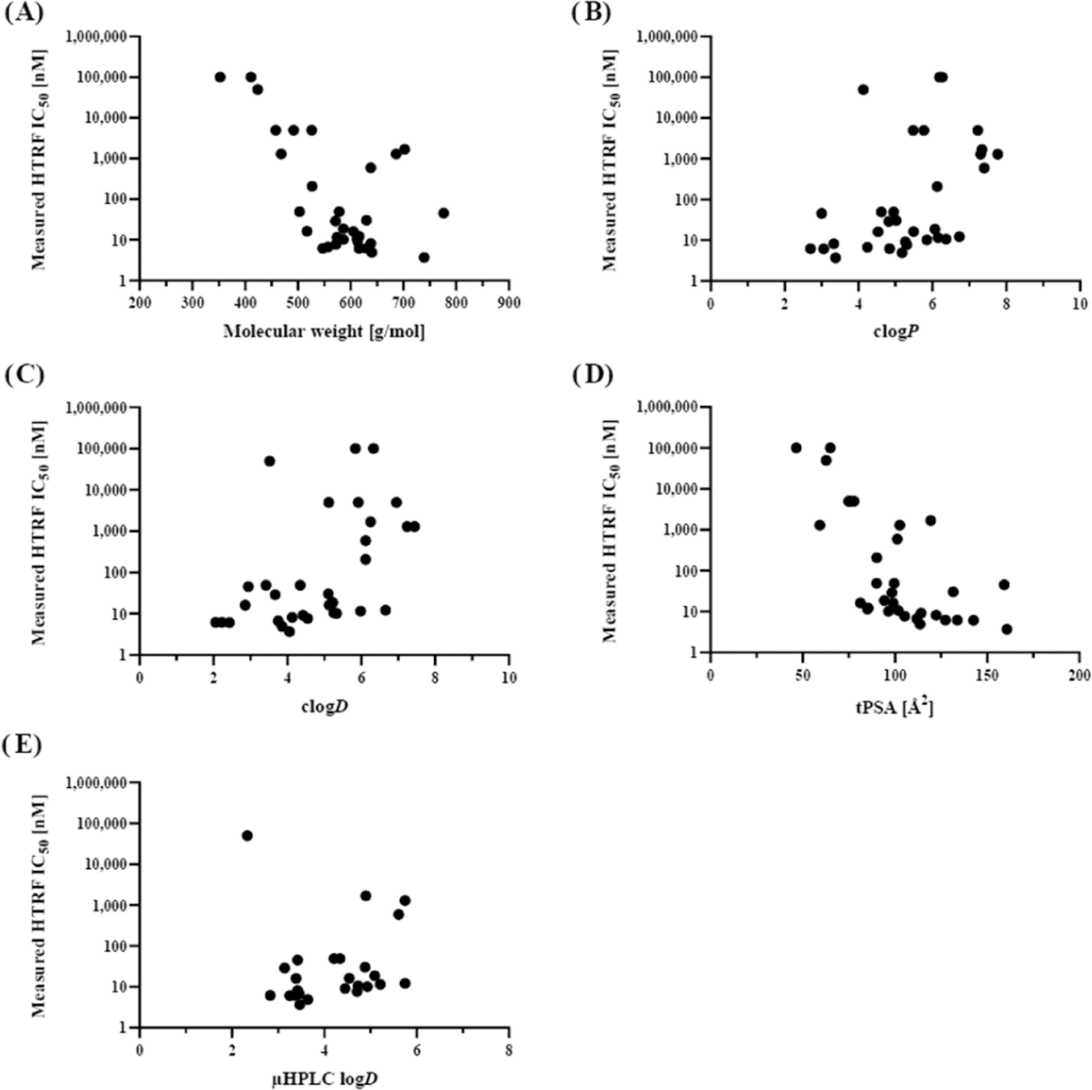
Correlation analysis of various computationally and experimentally obtained parameters with PD-L1 binding affinities (HTRF IC_50_). Correlation of (A) molecular weight, (B) clog*P*, (C) clog*D*, (D) tPSA, and (E) μHPLC log*D*.

The correlation analysis between various calculated and measured parameters and IC_50_ values revealed the following observations: a moderate correlation (ρ = −0.51, *p* = 0.003, *n* = 32) was found between molecular weight and affinity (IC_50_) supporting the indication that an adequate molecular size was required for sufficient binding (Figure 4A). There was a moderate correlation between clog*P* (ρ = 0.51, *p* = 0.003, *n* = 32) and clog*D* (ρ = 0.55, *p* = 0.001, *n* = 32) and measured IC_50_ values (Figure 4B,C). Calculated tPSA demonstrated a strong inverse correlation (ρ = −0.70, *p* = 0.000007, *n* = 32), implying an affinity–hydrophilicity relationship, although only a weak and statistically not significant correlation was observed for the measured μHPLC log*D* values (ρ = 0.30, *p* = 0.14, *n* = 25) (Figure 4D,E). These results highlight the complexity of reliably predicting PD-L1 binding affinities and suggest that multiple factors beyond molecular size, lipophilicity, and polar surface area may influence binding.

In further attempts to predict affinity *in silico*, 37 literature-known ligands encompassing a broad range of affinities^32,33^ and 25 novel compounds **4a**–**o** and **5a**–**j** underwent ligand docking (Table S3). Subsequently, the calculated binding affinity parameters were extracted and subjected to correlation analysis with literature HTRF IC_50_ or measured HTRF IC_50_ values. The results of our extensive ligand docking study revealed only very weak and weak correlations between docking parameters (i.e., Binding Affinity Score and Affinity) and the affinity of both literature-known ligands (ρ = −0.10, *p* = 0.57 and ρ = 0.26, *p* = 0.11, respectively) and novel compounds (ρ = 0.01, *p* = 0.95 and ρ = 0.33, *p* = 0.11, respectively) (Figure S3). These findings emphasize the challenges and significance of comprehending the precise determinants of binding affinity.

In summary, *de novo* synthesized small-molecule compounds reached excellent PD-L1 binding affinities in the low nanomolar range comparable to the antibody atezolizumab. This work represents a significant advancement in binding capabilities when compared to commercially available small molecules, such as PD-1/PD-L1 Inhibitor 1 (BMS-1; IC_50_ = 202 nM), PD-1/PD-L1 Inhibitor 2 (BMS-202; IC_50_ = 101 nM) and PD-1/PD-L1 Inhibitor 3 (a macrocyclic peptide; IC_50_ = 113 nM) (Table S2), as well as small molecules described in our previously reported ligand-based drug design approach.^31^

### Cell Viability and Cell-Based Competitive Binding Assay

**Figure 5:**
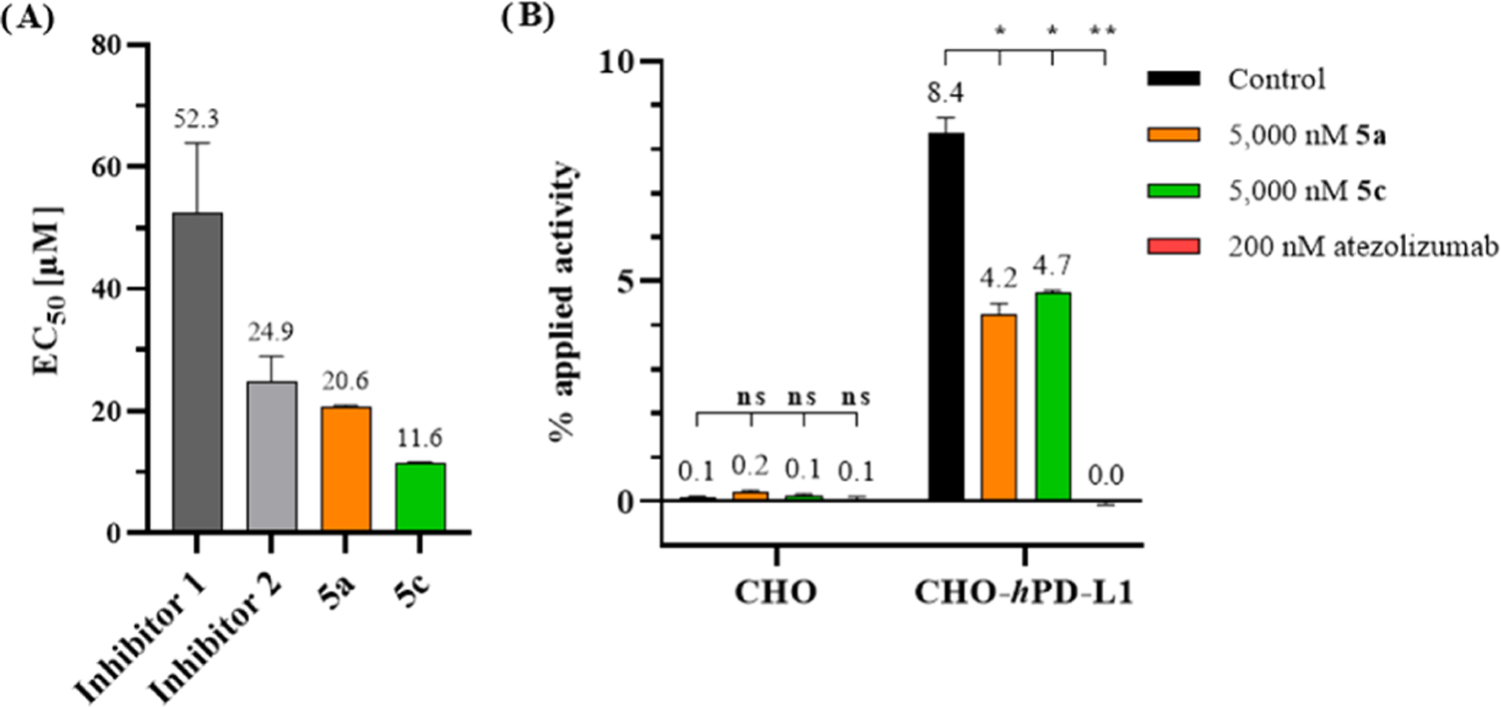
Results of cell viability and cell-based competitive binding assays. (A) Concentration-dependent effects of lead structures **5a** and **5c**, as well as published small molecules (PD-1/PD-L1 Inhibitor 1 and 2), on CHO-*h*PD-L1 cell viability after 24 h were assessed using the MTT assay. Relative EC_50_ is shown for **5a**. (B) Competitive radioligand binding assay using PD-L1-negative (CHO) and PD-L1-positive (CHO-*h*PD-L1) cells applying the radioligand [^89^Zr]Zr-atezolizumab. Radioligand binding was blocked by preincubation with 5000 nM **5a**, 5000 nM **5c**, or 200 nM atezolizumab. Statistical significance compared to control: *p* > 0.05 (ns), *p* ≤ 0.05 (*), *p* ≤ 0.01 (**).

Compounds **5a** and **5c**, exhibiting high affinities (IC_50_) of 6.2 and 10 nM, respectively, were chosen as promising candidates and underwent further *in vitro* evaluation using cell-based assays. The MTT assay, employed to assess cell viability, revealed cytotoxicity profiles similar to those of small-molecule compounds, including PD-1/PD-L1 Inhibitor 1 and 2 (BMS-1 & BMS-202) (Figures 5A and S4), as well as various other BMS compounds.^38^ Cell viability provided the basis for establishing a concentration range for subsequent cell-based *in vitro* investigations to avoid interferences with the results based on cellular death.

The cell-based PD-L1 binding affinity of **5a** and **5c** was evaluated using a competitive radioligand binding assay with the zirconium-89 labeled anti-PD-L1 antibody atezolizumab ([^89^Zr]Zr-atezolizumab) (Figure 5B). Indeed, [^89^Zr]Zr-atezolizumab exhibited no binding to PD-L1 negative CHO cells, but bound to PD-L1 positive CHO-*h*PD-L1 cells, and this binding was effectively blocked by preincubation with excess (>100-fold) unlabeled antibody (*p* = 0.0081), demonstrating its specificity. When **5a** and **5c** were administered at their highest noncytotoxic concentrations, a 50% blockade of antibody binding was observed (*p* = 0.034, *p* = 0.025, respectively). This can be translated into *K*_*i*_ values using the Cheng–Prusoff equation,^39^ resulting in a range of ∼700 to 4500 nM. The variation in *K*_*i*_ values is contingent upon the published *K*_D_ values for atezolizumab, which span from 0.195 to 9.96 nM.^7,8,20,40−43^

These results indicate that, on one hand, small-molecule compounds and atezolizumab share binding motifs on the PD-L1 protein as anticipated.^21,22^ On the other hand, it implies that their binding affinities might not be as robust as initially indicated by the cell-free HTRF assay, when using a more complex, biological system.

### Radiolabeling of High-Affinity Ligands

**Scheme 2:**
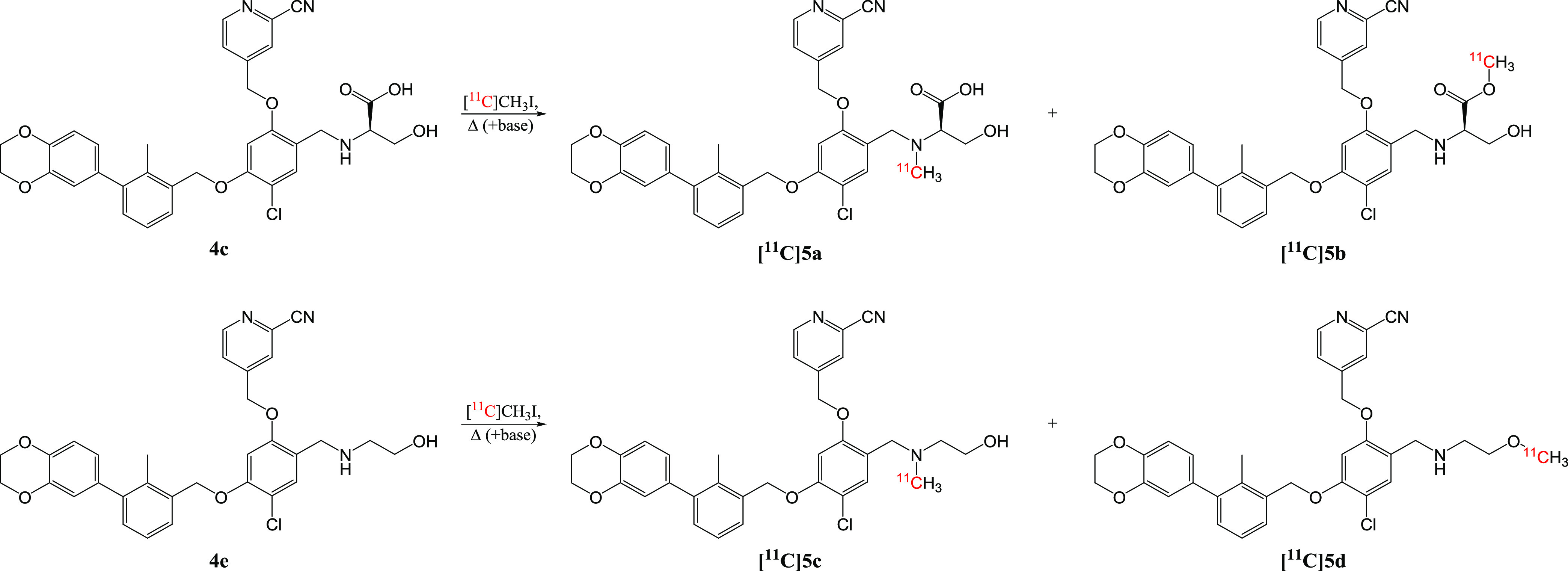
Radiolabeling Scheme for Desired Radiotracers **[**^**11**^**C]5a** and **[**^**11**^**C]5c** and Their Constitutional Isomers

Lead structures **5a** and **5c** were subjected to carbon-11 labeling by conventional ^11^C-methylation. Small-scale reactions were conducted to optimize the reaction conditions for enhanced radiochemical conversion (RCC) and selectivity for the desired *N*-methylated products **[**^**11**^**C]5a** or **[**^**11**^**C]5c** over their less affine *O*-methylated constitutional isomers **[**^**11**^**C]5b** or **[**^**11**^**C]5d**. These experiments involved varying precursor concentration, reaction temperature, and the addition of a base, using the demethylated precursors **4c** or **4e** and the [^11^C]CH_3_I synthon (Scheme 2).

**Figure 6:**
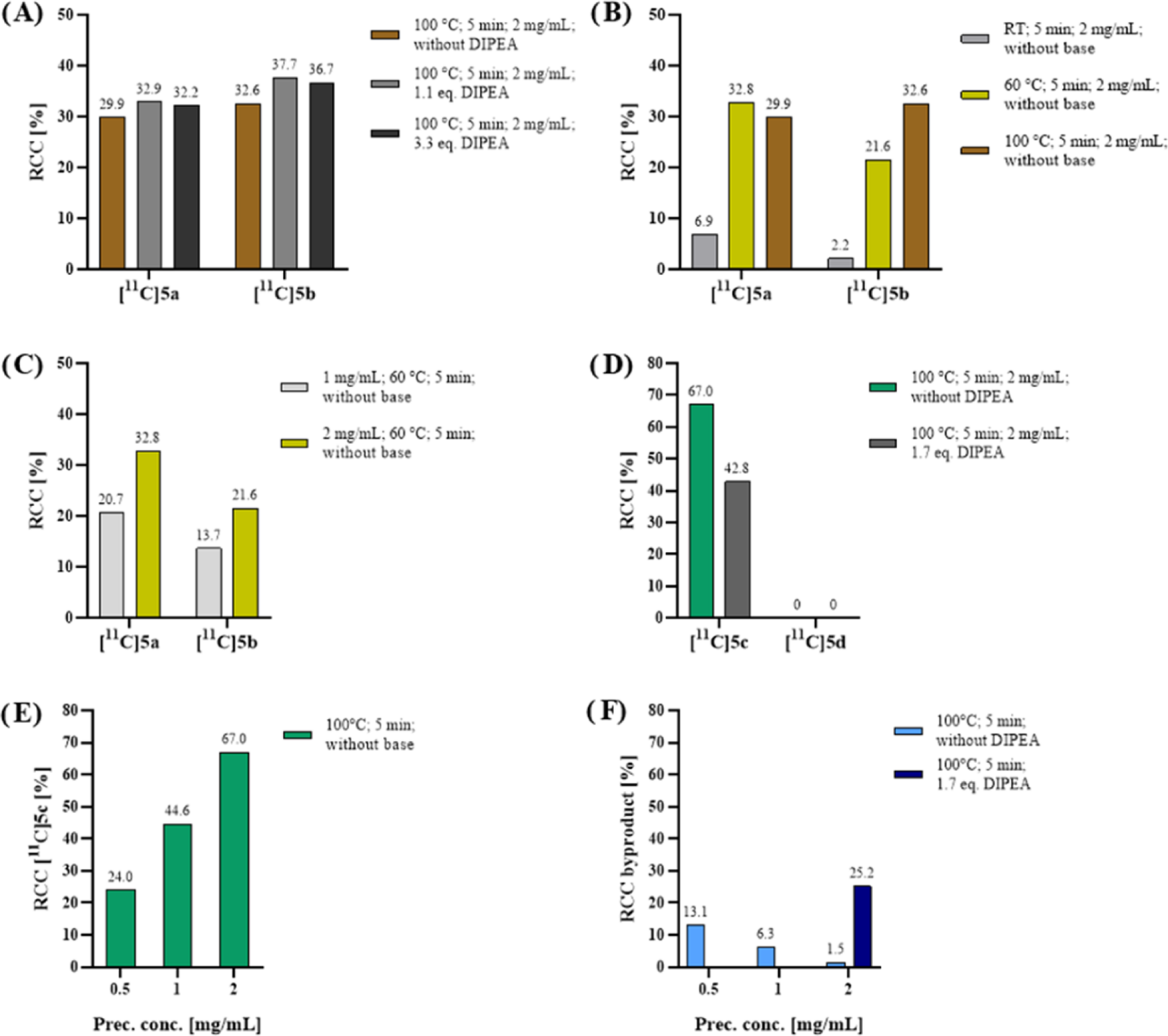
Results of small-scale radiolabeling using [^11^C]CH_3_I. (A) Base-dependent, (B) temperature-dependent, and (C) precursor concentration-dependent RCC of desired product **[**^**11**^**C]5a** and isomeric byproduct **[**^**11**^**C]5b** using precursor **4c**. (D) Base-dependent and (E) precursor concentration-dependent RCC of desired product **[**^**11**^**C]5c** and isomeric byproduct **[**^**11**^**C]5d** using precursor **4e**. (F) Precursor concentration- and base-dependent formation of an unidentified lipophilic byproduct during **[**^**11**^**C]5c** reactions. RCC = radiochemical conversion. DIPEA = *N*,*N*-diisopropylethylamine. RT = room temperature.

**[**^**11**^**C]5a** was obtained with an RCC of up to 32.9% and concurrent formation of 37.7% byproduct **[**^**11**^**C]5b** at 100 °C (Figures 6A and S5). Optimization of reaction conditions improved selectivity (Figure 6B). A reaction temperature of 60 °C, without the addition of a base, appeared to strike a favorable balance between achieving high radiochemical conversion and maintaining selectivity. Furthermore, a precursor concentration-dependent RCC was found, although the precursor concentration did not impact the isomer selectivity (Figure 6C).

Similarly, **[**^**11**^**C]5c** was selectively produced with exceptional RCCs of up to 67% at 100 °C (Figures 6D and S6). The absence of the *O*-methylated byproduct **[**^**11**^**C]5d** even in the presence of a base, suggests that DIPEA (calculated p*K*_a_ = 10.7) may not effectively deprotonate the alcohol of **4e** (calculated p*K*_a_ = 15.6). Alternatively, it could indicate that the formation of **[**^**11**^**C]5c** is kinetically favored over **[**^**11**^**C]5d**. Again, a precursor concentration-dependent correlation with RCC was identified (Figure 6E). As precursor concentration decreased and base was added, a more lipophilic, unidentified product emerged (Figure 6F), concurrent with the decrease of **[**^**11**^**C]5c** (Figure 6D), suggesting the formation of a potential dimethylated byproduct.

In summary, small-scale reactions successfully attained satisfactory RCC for both **[**^**11**^**C]5a** and **[**^**11**^**C]5c**. The superior RCC, coupled with feasible chromatographic separation of the product from precursor and byproducts, rendered **[**^**11**^**C]5c** the more favorable choice for subsequent *in vitro* and *in vivo* assessments.

Upscaling of **[**^**11**^**C]5c** radiosynthesis was performed using the GE TRACERlab FX2 C synthesis module paired with a semiprep. HPLC purification system (Figure S7) resulting in 2.3 ± 1.1 GBq of isolated product (*n* = 5) after 44.4 ± 2.8 min synthesis time, corresponding to 9 ± 4% radiochemical yield (decay corrected), with 95.5 ± 1.5% radiochemical purity (Figure S8), a molar activity of 107 ± 21 GBq/μmol, an osmolality of 271 ± 4 mmol/kg, and a pH of 5.02 ± 0.03.

A limit of detection (LOD) and limit of quantification (LOQ) of 1.39 μg/mL (2.37 μM) and 4.20 μg/mL (7.17 μM), respectively, was calculated from the standard curve (Figure S9).

### Plasma Stability, Plasma Protein Binding, and Metabolic Stability

**Figure 7:**
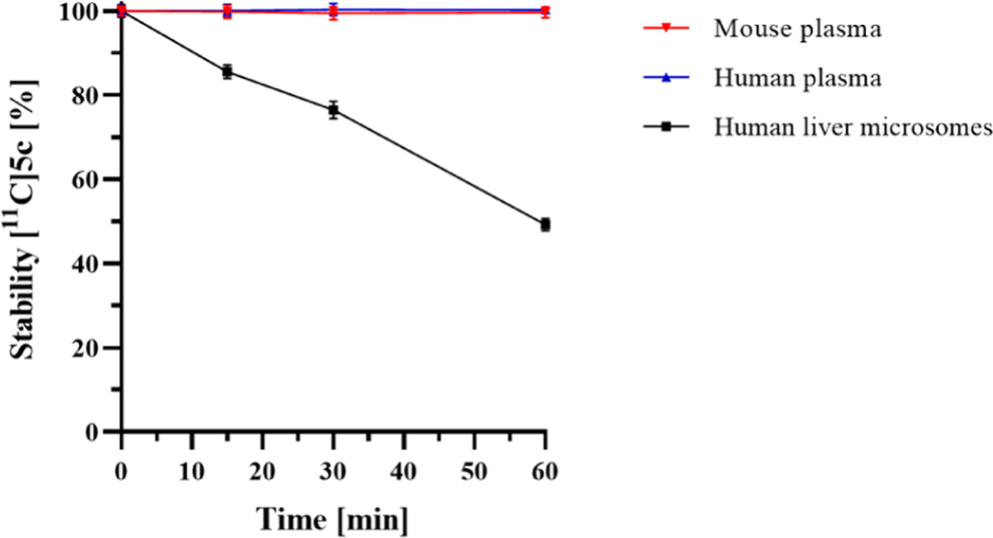
Stability of **[**^**11**^**C]5c** over time in mouse plasma, human plasma, or human liver microsomes at 37 °C.

Radiotracer **[**^**11**^**C]5c** underwent additional evaluation to assess its plasma stability and metabolic stability for subsequent *in vivo* investigations. It exhibited remarkable stability, with over 99% remaining intact for 60 min in both mouse and human plasma (Figure 7). A high plasma protein binding of 98.9% aligns with expectations, given the tracer’s lipophilic properties. Furthermore, when subjected to incubation with human liver microsomes for 60 min, 49.3% of the radiotracer remained intact. These findings connote that **[**^**11**^**C]5c** exhibits sufficient stability for *in vivo* investigations.

### *In Vivo* μPET Imaging and Biodistribution

**Figure 8:**
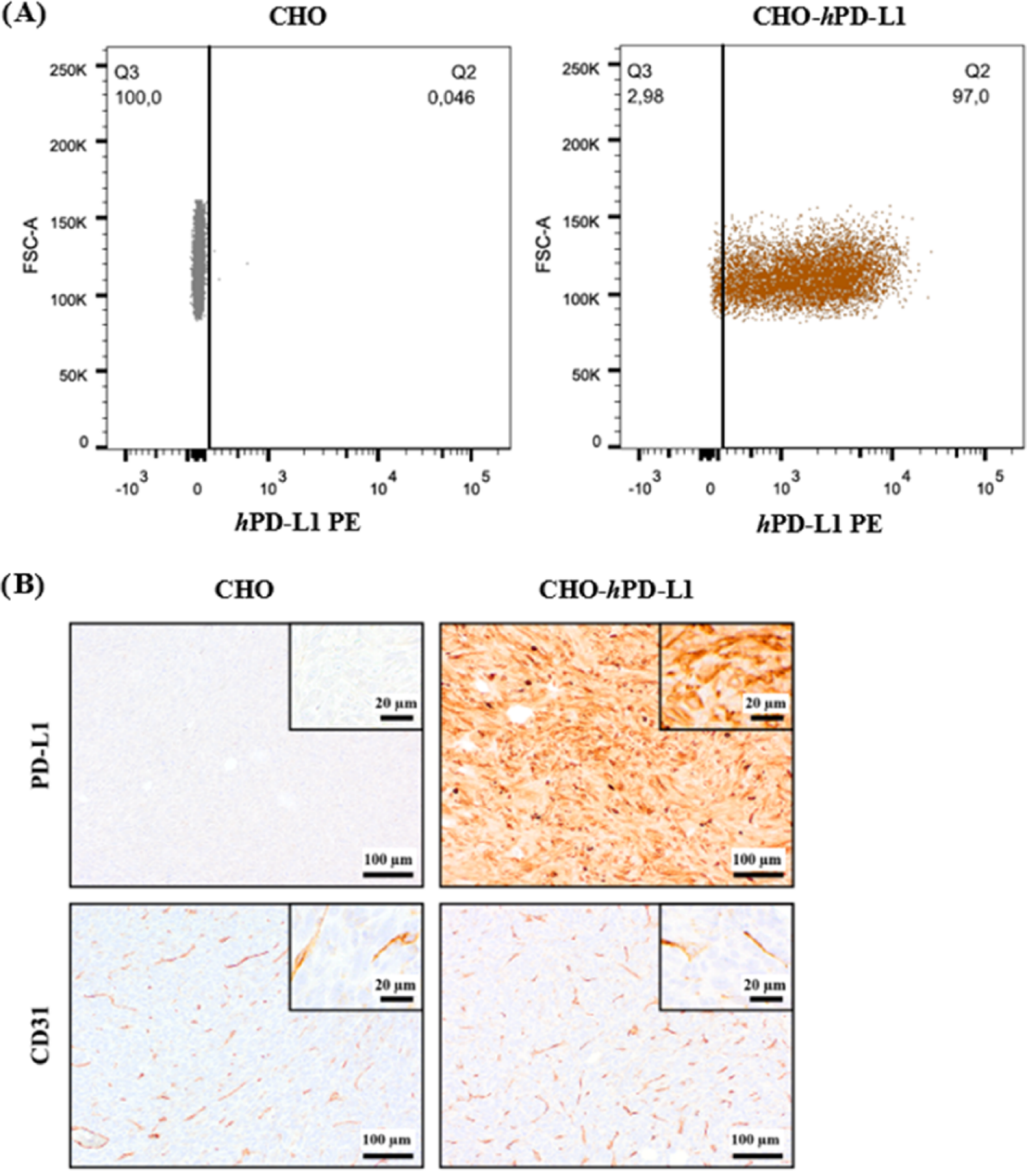
Verification of PD-L1 expression in CHO and CHO-*h*PD-L1 cell lines. (A) Flow cytometry of CHO (left) and CHO-*h*PD-L1 (right) cells. (B) *Ex vivo* immunohistochemistry analysis of CHO (left) and CHO-*h*PD-L1 (right) xenografts regarding PD-L1 and CD31 expression (brown staining).

PD-L1 expression in both cell lines (CHO and CHO-*h*PD-L1) was confirmed *in vitro* through flow cytometry (Figure 8A) and *ex vivo via* immunohistochemistry (Figure 8B). Tumor vascularization was verified by CD31 staining (Figure 8B). Dynamic μPET/CT imaging was conducted using **[**^**11**^**C]5c** to evaluate its *in vivo* potential for quantifying PD-L1 expression. NSG mice with both PD-L1 negative (CHO) and PD-L1 overexpressing (CHO-*h*PD-L1) xenografts were used for this assessment. Following the imaging, the mice were sacrificed for subsequent *ex vivo* biodistribution analysis.

**Figure 9:**
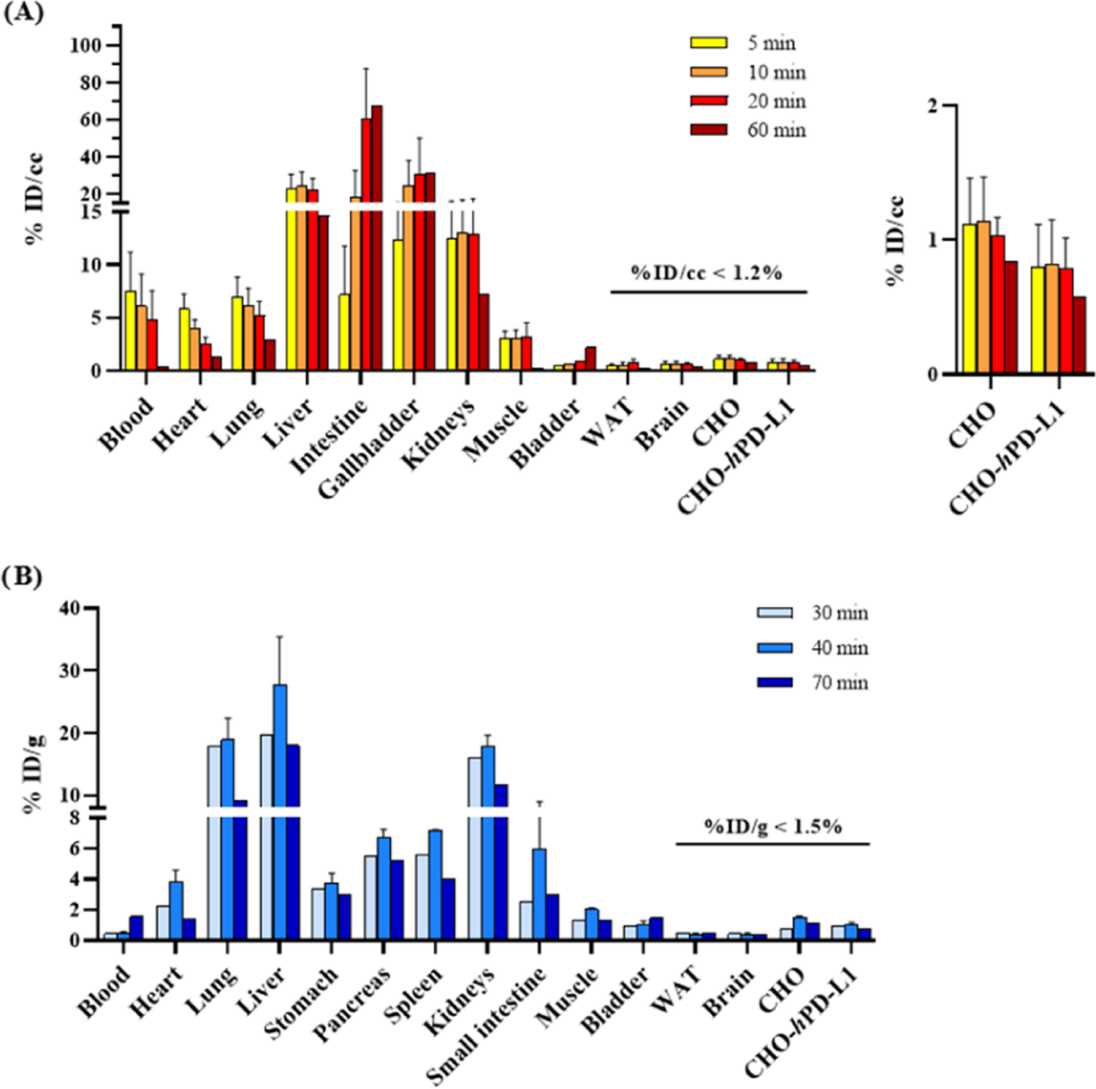
Biodistribution of tracer **[**^**11**^**C]5c** during dynamic μPET/CT scans and as assessed by *ex vivo* biodistribution. (A) *In vivo* biodistribution of tracer accumulation performed for 20 (*n* = 3) or 60 (*n* = 1) min. (B) *Ex vivo* biodistribution of tracer accumulation after 30 (*n* = 1), 40 (*n* = 2) or 70 (*n* = 1) min. % ID/cc and % ID/g are shown at indicated time points post radiotracer injection. Error bars are expressed as mean ± standard deviation. WAT = white adipose tissue.

**Figure 10:**
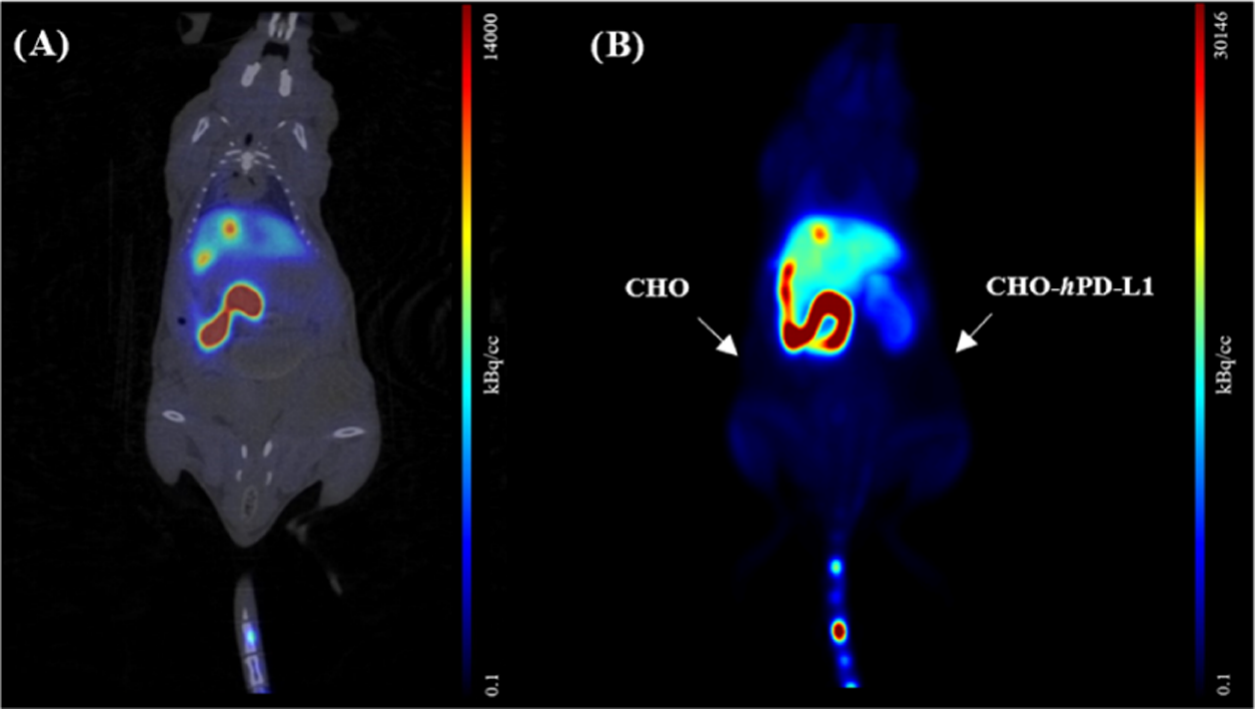
*In vivo* distribution of **[**^**11**^**C]5c** during dynamic a μPET/CT scan. (A) Coronal μPET/CT fusion image acquired after 60 min scan time and (B) averaged (0–60 min p.i.) maximum-intensity projection μPET image of an NSG mouse bearing CHO and CHO-*h*PD-L1 xenografts after i.v. injection of 31 MBq of **[**^**11**^**C]5c**.

**[**^**11**^**C]5c** exhibited high uptake in the liver, intestine, gallbladder, and kidneys, while displaying no uptake (<1.2% ID/cc) in both CHO xenografts (Figures 9A, 10, S10, and S11). Neither the radiotracer nor its potential radiometabolites accumulated in white adipose tissue (WAT) or the brain. Correspondingly, the *ex vivo* biodistribution results demonstrated substantial uptake in the liver, lung, and kidneys (Figure 9B).

The elevated kidney uptake may be attributed to a combination of perfusion, excretion, and reabsorption processes. As anticipated for this lipophilic tracer, the hepatobiliary system emerged as the principal pathway for tracer metabolism and excretion, as evidenced by the time-dependent increase observed in the intestines. Conversely, minimal radioactivity was discerned in the bladder, indicative of limited urinary excretion. The elevated lung uptake observed in our *ex vivo* study, along with the observed uptake in the liver and kidneys *ex vivo* and *in vivo*, is likely a consequence of perfusion effects rather than specific target binding. This phenomenon is expected due to the considerable blood volume and rapid perfusion rates in mice within these organs.^44^

In summary, **[**^**11**^**C]5c** exhibited an absence of specific binding to both PD-1 negative and PD-1 expressing xenografts. This observation could potentially be attributed to rapid radiotracer metabolism, constrained tissue penetration due to pronounced plasma protein binding, or heightened levels of nonspecific or off-target interactions. To gain deeper insights into the underlying factors contributing to the absence of specific binding observed in **[**^**11**^**C]5c**, further comprehensive investigations and experiments may be warranted. These endeavors could encompass conducting additional *in vivo* studies to elucidate metabolic pathways, refining the radiotracer formulation to enhance tissue penetration, and assessing potential off-target binding via competitive binding studies or other pertinent methodologies. These findings would provide valuable insights for the development and refinement of **[**^**11**^**C]5c** or similar radiotracers for PD-L1 imaging applications.

## Conclusions

A plethora of intermediates and a collection of 10 final compounds were effectively synthesized through a series of judiciously selected reactions, including Suzuki coupling, Mitsunobu reaction, nucleophilic substitution, and reductive amination. These synthesized compounds exhibited remarkable nanomolar affinities as ascertained through a well-established HTRF assay, enabling the elucidation of profound structure–activity relationships. Notably, our synthesis efforts culminated in the creation of a novel database housing potential PD-L1 ligands. Additionally, a highly promising candidate was efficiently radiolabeled via ^11^C-methylation, achieving a radiochemical yield of 9 ± 4%. Subsequent *in vivo* evaluation of **[**^**11**^**C]5c** revealed no discernible specific uptake in PD-L1 expressing CHO xenografts, while manifesting elevated uptake in excretory organs. The observed disparity between superior *in vitro* and comparatively inferior *in vivo* results necessitates further studies to unravel the underlying factors. This study underscores the critical importance of refined *in vitro* methodologies for predicting and comprehending *in vivo* pharmacokinetics more accurately.

## Experimental Section

### General Information

Solvents and chemicals were obtained from commercial suppliers and used without further purification unless otherwise stated. All synthesized compounds were ≥95% pure as assessed by high-performance liquid chromatography (HPLC).

### High-Performance Liquid Chromatography

#### Setup 1

Reaction progress, compound purity, and *in vitro* stability were measured with an Agilent 1200 series LC system (Agilent Technologies, Inc., Santa Clara, USA) paired with an Agilent 1100 series autosampler and an XBridge C18 HPLC column, 5 μm, 4.6 mm × 150 mm (Waters Corporation, Eschborn, Germany). GINA Star Software (Raytest Isotopenmessgeräte GmbH, Straubenhardt, Germany) was used for data acquisition. Solvent “A” consisted of a 10 mmol/L sodium phosphate (Merck KGaA, Darmstadt, Germany) buffer adjusted to pH 7.4 with 1 mol/L NaOH (Merck KGaA, Darmstadt, Germany) and solvent “B” of 90% v/v acetonitrile (MeCN) (Merck KGaA, Darmstadt, Germany) plus 10% v/v Milli-Q H_2_O (Merck KGaA, Darmstadt, Germany). The flow rate was set to 1.5 mL/min. A mobile phase gradient of 70% A: 30% B to 20% A: 80% B within 20 min and a hold until the end of the run was used.

#### Setup 2

For semipreparative purification, an Agilent 1200 series LC system was paired with a SUPELCOSIL ABZ+ HPLC Column, 5 μm, 25 cm × 10 mm (Merck KGaA, Darmstadt, Germany). Solvent “A” consisted of 90% v/v MeCN plus 10% v/v Milli-Q H_2_O and solvent “B” of 10 mmol/L sodium phosphate buffer adjusted to pH 7.4 with 1 mol/L NaOH. The flow rate was set to 5 mL/min. A mobile phase gradient of 30% A: 70% B to 60% A: 40% B within 20 min and a hold until the end of the run was used.

#### Setup 3

For log *D* measurements, an Agilent 1200 series was paired with an Agilent 1100 autosampler and Agilent 1100 UV detector, an apHera column, 5 μm, 10 mm × 6 mm (Merck KGaA, Darmstadt, Germany), GINA Star Software for data acquisition and a mobile phase gradient of 10% A and 90% B to 100% A within 9.4 min and back to starting conditions until minute 12. An equilibration time of 2 min before measurements has been set. Solvent “A” consisted of methanol (MeOH) (Merck KGaA, Darmstadt, Germany) and solvent “B” of 10 mmol/L sodium phosphate buffer pH 7.4. The flow rate was set to 1.5 mL/min.

#### Setup 4

For semipreparative purification after radiosynthesis the GE TRACERlab FX2 C synthesis module (General Electric Medical Systems, Sweden) was paired with a Sykam S1122 pump (Sykam, Eresing, Germany), a BlueShadow UV detector (KNAUER Wissenschaftliche Geräte GmbH, Berlin, Germany), and a SUPELCOSIL ABZ+ HPLC Column, 5 μm, 25 cm × 10 mm. The solvent consisted of 90% MeCN and 10% Milli-Q H_2_O. The flow rate was set to 5 mL/min.

For high-performance liquid chromatography measurements after radiosynthesis, an Agilent Technologies 1620 Infinity system was utilized with an XBridge BEH RP18 XP column, 2.5 μm, 3 cm × 5 cm (Waters Corporation, Eschborn, Germany), as stationary phase and GINA Star Software for data acquisition. “A” consisted of 90% v/v MeCN in Milli-Q H2O and “B” of 50 mmol/L ammonium dihydrogen phosphate (Honeywell International, Inc., Charlotte, USA) adjusted to pH 9.3 with 5 mol/L NaOH. For biocide purposes, a spatula tip’s worth of NaN3 was added to “B”.

#### Setup 5

A mobile phase gradient of 40% A: 60% B to 55.5% A: 44.5% B within 5 min and a hold until the end of the run was used. Flow rate was set to 1.0 mL/min.

#### Setup 6

An isocratic mobile phase of 50% A: 50% B and a flow rate of 1.5 mL/min was used.

### Compound Characterization

^1^H NMR, ^13^C NMR (DEPTQ), ^19^F-NMR, and 2D NMR spectra were recorded in CDCl_3_ or DMSO-*d*_6_ (Merck KGaA, Darmstadt, Germany) on Bruker AV NEO 400, AV NEO 500 WB, AV III 600 or AV III HD 700 spectrometers (Bruker, Mannheim, Germany). Spectra evaluation was performed using MestReNova 14.2 software (Mestrelab Research S.L., Santiago de Compostela, Spain).

Full-scan high-resolution mass spectra (*m*/*z* 50–1600) of the compounds dissolved in MeCN/MeOH and 1% H_2_O were obtained by direct infusion measurements on a maXis ESI-Qq-TOF mass spectrometer (Bruker, Mannheim, Germany). The sum formulas of the detected ions were determined using Compass DataAnalysis 4.0 (Bruker, Mannheim, Germany) based on the mass accuracy (Δ*m*/*z* ≤ 5 ppm) and isotopic pattern matching (SmartFormula algorithm).

Compound characterization data is provided in the Supporting Information (Figures S10–S19).

### Interference Compounds Test

Virtually and synthetically obtained structures were filtered for Pan Assay Interference Compounds (PAINS) using the ZINC online filter.^45^

### Ligand Docking Experiments

#### Pharmacophore Screening Study

A ChEMBL database^46^ containing bioactive compounds with molecular weights ranging from 4 to 200 g/mol was subjected to screening against a consensus feature-based pharmacophore derived within LigandScout 4.4 software (Inte:Ligand GmbH, Vienna, Austria). The pharmacophore was constructed based on X-ray crystallography data extracted from specific Protein Data Bank (PDB) entries,^47^ including codes 5J89, 5J8O, 5N2D, 5N2F, 6R3K, and 6NM8. Pharmacophore-Fit Scores were computed for each compound, resulting hits were transposed to the PDB entry 5J89, and Binding Affinity Scores were calculated. Hits were ranked according to their Pharmacophore-Fit Scores and Binding Affinity Scores, respectively, and assigned to a maximum of 10 points each score (max. 20 points total).

#### Ligand Docking Study

PDB 6R3K ligand was re-docked for validation of the docking procedure.^48^ A root-mean-square deviation (RMSD) of 0 Å was achieved between the docked and original pose, highlighting its reliability.

Newly synthesized and literature-known compound structures were protonated to pH 7.4 using MarvinSketch 22.13 software. Ligand docking was then performed with LigandScout 4.4 software using the AutoDock Vina 1.1 program and PDB code 6R3K (PD-L1 monomer C and D). The PD-L1 protein structure was maintained as a rigid entity, enabling flexible ligand docking. Water and ethylene glycol molecules were removed prior to docking. The grid box dimensions, approximately 30 × 30 × 30 Å, were automatically determined by LigandScout. Docking, performed in triplicates for enhanced consistency, adhered to default settings (Exhaustiveness: 8; Max. number of modes: 9; Max. energy difference: 3). Postdocking refinement of docking poses was not undertaken to maintain the integrity of the results.

### Syntheses

#### General Procedures

##### Mitsunobu Reaction—General Procedure 1

The alcohol (1 equiv), acid (1–2 equiv), and triphenylphosphine (1.5 equiv) were dissolved in an organic solvent on ice under N_2_ atmosphere, followed by slow addition of azodicarboxylate (1.5 equiv), and finally stirred at room temperature for several days. Products were purified by semipreparative silica gel chromatography.

##### Mitsunobu Reaction—General Procedure 2

General procedure 2 was used when general procedure 1 showed low conversion: Triphenylphosphine (2 equiv) and azodicarboxylate (2 equiv) were first mixed on ice—preforming the betaine—under N_2_ atmosphere, followed by the addition of the alcohol (1 equiv), the acid (1 equiv), and eventually stirred at room temperature for several days. Products were purified by semipreparative silica gel chromatography.

##### Nucleophilic Substitution—General Procedure 3

The electrophile (2 equiv) was premixed with catalytic amounts of iodide salt, dissolved in organic solvent, and added to the nucleophile (1 equiv) and base (2–3 equiv) under inert atmosphere, and stirred at room temperature for 1 day. Products were purified by semipreparative silica gel chromatography.

##### Reductive Amination—General Procedure 4

The aldehyde (1 equiv), amine (2–4 equiv), and acetic acid (excess) were dissolved in dichloromethane (DCM) under inert atmosphere and stirred at room temperature for 1 h. Sodium triacetoxyborohydride (1.5–4 equiv) was added and stirred for 1–4 days. Products were purified by semipreparative high-performance liquid chromatography.

##### Reductive Amination—General Procedure 5

General procedure 4 was adapted for hydrophilic amines (insoluble in DCM): The aldehyde (1 equiv), amine (2–4 equiv), and acetic acid (excess) were dissolved in *N*,*N*-dimethylformamide (DMF) and MeOH under inert atmosphere, and stirred at room temperature for 1 h. Sodium cyanoborohydride (3–6 equiv) was added and stirred for 1–4 days. Products were purified by semipreparative high-performance liquid chromatography.

##### Synthesis of (3-(2,3-Dihydrobenzo[*b*][1,4]dioxin-6-yl)-2-methylphenyl)methanol (**1b**)

1,4-Benzodioxane-6-boronic acid (202 mg, 1.12 mmol, 1.0 equiv) (Merck KGaA, Darmstadt, Germany), 3-bromo-2-methylbenzyl alcohol (228 mg, 1.13 mmol, 1.0 equiv) (Apollo Scientific Ltd., Stockport, U.K.), and XPhos Pd G3 catalyst (38.7 mg, 45.7 μmol, 0.04 equiv) were dissolved in anhydrous tetrahydrofuran (THF) (4.45 mL) (Merck KGaA, Darmstadt, Germany) under inert N_2_ atmosphere in a round-bottom flask. Aqueous 0.5 mol/L K_2_CO_3_ (4.45 mL, 2.22 mmol, 2.0 equiv) (Merck KGaA, Darmstadt, Germany) solution was deoxygenated with N_2_ and subsequently added. The reaction mixture was stirred at room temperature for 5 days protected from light. Ethyl acetate (EtOAc) (10 mL) (Honeywell International, Inc., Charlotte, USA) and saturated NaCl (brine) (10 mL) (Merck KGaA, Darmstadt, Germany) were added to the reaction solution. The yellow-brown organic phase was separated, washed with brine (5 mL), and dried over Na_2_SO_4_ (Merck KGaA, Darmstadt, Germany). After filtration, the organic solvent was removed *in vacuo* leaving a brown oil. The product was isolated by semipreparative silica gel 60 (Merck KGaA, Darmstadt, Germany) column chromatography using 3:1 hexane/EtOAc (Honeywell International, Inc., Charlotte, USA) and 2:1 hexane/EtOAc solvent mixtures consecutively. Organic solvents were removed *in vacuo* yielding a yellow-orange, highly viscous oil (183 mg, 64% yield).

^1^H NMR (400 MHz, CDCl_3_): δ 7.36 (d, *J =* 7.4 Hz, 1H), 7.23 (t, *J =* 7.5 Hz, 1H), 7.18 (d, *J =* 7.6 Hz, 1H), 6.90 (d, *J =* 8.2 Hz, 1H), 6.81 (d, *J =* 2 Hz), 6.76 (dd, *J =* 8.3 Hz, *J =* 2.1 Hz, 1H), 4.76 (s, 2H), 4.30 (s, 4H), 2.26 (s, 3H).

ESI-MS ([M – H]^−^): *m*/*z* calculated ([C_16_H_16_O_3_ – H]^−^) = 255.1021. Found = 255.1021.

##### Synthesis of (2-Methyl-3-(1*H*-pyrrol-1-yl)phenyl)methanol (**1c**)

2-Methyl-3-pyrrol-1-yl-benzoic acid (100 mg, 0.820 mmol, 1.0 equiv) (Matrix Scientific, Elgin, USA) was dissolved in anhydrous THF (2.8 mL) under N_2_ atmosphere and stirred on ice in a round-bottom flask. A 1 mol/L LiAlH_4_ solution in THF (1.23 mL, 1.23 mmol, 1.5 equiv) (Merck KGaA, Darmstadt, Germany) was slowly added. The reaction mixture was subsequently stirred on ice for 25 min and refluxed for 1 h. Diethyl ether (DEE) (5 mL) (Merck KGaA, Darmstadt, Germany) mixed with Milli-Q H_2_O (0.1 mL) was slowly added (H_2_ gas formation!). After complete quenching, the precipitate was filtered, and the remaining organic phase was washed with 1 mol/L NaOH and dried over Na_2_SO_4_. Thin-layer chromatography using precoated silica gel 60 F_254_ and 1:1 hexane/EtOAc showed only one product spot and no starting material. Organic solvents were removed *in vacuo* yielding a dark orange, highly viscous oil without further purification (68.6 mg, 74% yield).

^1^H NMR (500 MHz, CDCl_3_): δ 7.43 (d, *J* = 7.3 Hz, 1H), 7.29–7.22 (m, 2H), 6.76 (t, *J =* 2.1 Hz, 2H), 6.32 (t, *J =* 2.1 Hz, 2H), 4.77 (s, 2H), 2.13 (s, 3H).

^13^C NMR (126 MHz, CDCl_3_): δ 127.09, 126.62, 126.38, 122.48, 108.87, 63.81, 13.34.

ESI-MS ([M + H]^+^): *m*/*z* calculated ([C_12_H_13_NO + H]^+^) = 188.1070. Found = 188.1068.

##### Synthesis of 5-Chloro-6-((2-methyl-[1,1′-biphenyl]-3-yl)methoxy)nicotinaldehyde (**2a**)

Triphenylphosphine (PPh_3_) (53 mg, 202 μmol, 2.0 equiv) (Thermo Fisher Scientific, Inc., Waltham, USA) and diethyl azodicarboxylate (DEAD, 40% in toluene) (91.9 μL, 202 μmol, 2.0 equiv) (Merck KGaA, Darmstadt, Germany) were dissolved in anhydrous THF on ice in a round-bottom flask under N_2_ atmosphere. (2-Methyl-[1,1′-biphenyl]-3-yl)methanol (**1a**) (20 mg, 101 μmol, 1.0 equiv) (TCI Deutschland GmbH, Eschborn, Germany) and 5-chloro-6-hydroxynicotinaldehyde (15.9 mg, 101 μmol, 1.0 equiv) (Apollo Scientific Ltd., Stockport, U.K.) were separately dissolved in toluene (1 mL) (Merck KGaA, Darmstadt, Germany), as well as MeCN (1 mL) and *N*,*N*-dimethylformamide (DMF) (0.5 mL) (Merck KGaA, Darmstadt, Germany), respectively. These solutions were then introduced into the cooled reaction mixture. The mixture was stirred on ice for 1 h, followed by continuous stirring at room temperature for a duration of 3 days. Organic solvents were removed *in vacuo* and the product was purified by silica gel chromatography using a 9:1 DCM/DEE (Merck KGaA, Darmstadt, Germany) solvent mixture. DCM and DEE were removed *in vacuo* and subsequently dried overnight within a desiccator containing molecular sieves yielding off-white crystals (11.2 mg, 33% yield).

Purity: 96.45% as determined by HPLC setup 1, UV detector: 254 nm.

^1^H NMR (400 MHz, CDCl_3_): δ 9.51 (s, 1H), 8.04 (d, *J =* 2.2 Hz, 1H), 7.70 (d, *J =* 2.2 Hz, 1H), 7.44–7.28 (m, 7H), 7.14 (m, 1H), 5.32 (s, 2H), 2.15 (s, 1H).

ESI-MS ([M + H]^+^): *m*/*z* calculated ([C_20_H_16_ClNO_2_ + H]^+^) = 338.0942. Found = 338.0941.

##### Synthesis of 5-Chloro-2-hydroxy-4-((2-methyl-[1,1′-biphenyl]-3-yl)methoxy)benzaldehyde (**2b**)

(2-Methyl-[1,1′-biphenyl]-3-yl)methanol (**1a**) (27.0 mg, 136 μmol, 1.1 equiv), 5-chloro-2,4-dihydroxybenzaldehyde (22.3 mg, 129 μmol, 1.0 equiv) (abcr GmbH, Karlsruhe, Germany), and PPh_3_ (53 mg, 202 μmol, 1.6 equiv) were dissolved in DCM (3 mL) on ice in a round-bottom flask under N_2_ atmosphere. DEAD (40% in toluene) (93 μL, 204 μmol, 1.6 equiv) was diluted with DCM (1 mL) and added slowly to the cooled reaction mixture. The ice bath was removed, and the reaction mixture was continuously stirred at room temperature for 5 days. Organic solvents were removed *in vacuo* and the product was purified by silica gel chromatography using a 2:1 hexane/EtOAc mixture. Organic solvents were removed *in vacuo* yielding colorless crystals (15.9 mg, 34% yield).

Purity: 99.55% as determined by HPLC setup 1, UV detector: 254 nm.

^1^H NMR (400 MHz, CDCl_3_): δ 11.44 (s, 1H), 9.71 (s, 1H), 7.55 (s, 1H), 7.49–7.28 (m, 8H), 6.64 (s, 1H), 5.22 (s, 2H), 2.25 (s, 3H).

ESI-MS ([M – H]^−^): *m*/*z* calculated ([C_16_H_16_O_3_ – H]^−^) = 351.0788. Found = 351.0799.

##### Synthesis of 5-Chloro-4-((3-(2,3-dihydrobenzo[*b*][1,4]dioxin-6-yl)-2-methylbenzyl)oxy)-2-hydroxybenzaldehyde (**2c**)

(3-(2,3-Dihydrobenzo[*b*][1,4]dioxin-6-yl)-2-methylphenyl)methanol (**1a**) (500 mg, 1.95 mmol, 1.0 equiv), 5-chloro-2,4-dihydroxybenzaldehyde (673 mg, 3.90 mmol, 2.0 equiv), and PPh_3_ (768 mg, 2.93 mmol, 1.5 equiv) were dissolved in dry DCM (40 mL) on ice in a round-bottom flask under N_2_ atmosphere. DEAD (40% in toluene) (1.33 mL, 2.93 mmol, 1.5 equiv) was diluted with DCM (10 mL) and added slowly to the cooled reaction mixture. The ice bath was removed, and the reaction mixture was continuously stirred at room temperature for 3 days. Organic solvents were removed *in vacuo* and dry THF (5 mL) was added and filtered. The precipitate contained the product. Filtrate was concentrated *in vacuo*, cooled at −20 °C for precipitation, and filtered. Pooled precipitates were washed with ice-cold THF. The product was obtained as an off-white solid (420 mg, 52% yield).

Purity: 99.00% as determined by HPLC setup 1, UV detector: 254 nm.

^1^H NMR (400 MHz, CDCl_3_): δ 11.43 (s, 1H), 9.70 (s, 1H), 7.55 (s, 1H), 7.44 (dd, *J =* 6.2 Hz, *J* = 2.7 Hz, 1H), 7.28–7.23 (m, 2H), 6.91 (d, *J =* 8.2 Hz, 1H), 6.83 (d, *J =* 2.0 Hz), 6.78 (dd, *J =* 8.2 Hz, *J =* 2.0 Hz, 1H), 6.63 (s, 1H), 5.20 (s, 2H), 4.31 (s, 4H), 2.27 (s, 3H).

ESI-MS ([M – H]^−^): *m*/*z* calculated ([C_23_H_19_ClO_5_ – H]^−^) = 409.0843. Found = 409.0852.

##### Synthesis of 5-Chloro-2-hydroxy-4-((2-methyl-3-(1*H*-pyrrol-1-yl)benzyl)oxy)benzaldehyde (**2d**)

(2-Methyl-3-(1*H*-pyrrol-1-yl)phenyl)methanol (**1c**) (167 mg, 892 μmol, 1.0 equiv), 5-chloro-2,4-dihydroxybenzaldehyde (154 mg, 892 μmol, 1.0 equiv), and PPh_3_ (351 mg, 1.34 mmol, 1.5 equiv) were dissolved in dry THF (20 mL) on ice in a round-bottom flask under N_2_ atmosphere. DEAD (40% in toluene) (609 μL, 1.34 mmol, 1.5 equiv) was diluted with THF (10 mL) and added slowly to the cooled reaction mixture. The ice bath was removed, and the reaction mixture was continuously stirred at room temperature for 3 days. Organic solvents were removed *in vacuo*, and the product was purified by silica gel chromatography using a 2:1 hexane/EtOAc mixture. Organic solvents were removed *in vacuo* yielding a colorless solid (73.4 mg, 24% yield).

Purity: 96.95% as determined by HPLC setup 1, UV detector: 254 nm.

^1^H NMR (600 MHz, CDCl_3_): δ 11.44 (s, 1H), 9.71 (s, 1H), 7.56 (s, 1H), 7.51 (m, 1H), 7.31 (m, 2H), 6.79 (t, *J =* 2.1 Hz, 2H), 6.62 (s, 1H), 6.33 (t, *J* = 2.1 Hz, 2H), 5.20 (s, 2H), 2.16 (s, 3H).

ESI-MS ([M – H]^−^): *m*/*z* calculated ([C_19_H_16_ClNO_3_ – H]^−^) = 340.0740. Found = 340.0747.

##### Synthesis of 3-((4-Chloro-2-formyl-5-((2-methyl-[1,1′-biphenyl]-3-yl)methoxy)phenoxy)methyl)benzonitrile (**3a**)

5-Chloro-2-hydroxy-4-((2-methyl-[1,1′-biphenyl]-3-yl)methoxy)benzaldehyde (**2b**) (1.00 mg, 2.96 μmol, 1.0 equiv) was mixed with cesium carbonate (Cs_2_CO_3_, trace-metal basis) (1.93 mg, 5.92 μmol, 2.0 equiv) (Merck KGaA, Darmstadt, Germany) under N_2_ atmosphere in a round-bottom flask. 3-(Bromomethyl)benzonitrile (1.16 mg, 5.92 μmol, 2.0 equiv) (Merck KGaA, Darmstadt, Germany) and catalytic amounts of potassium iodide (KI) (0.04 mg, 0.24 μmol, 0.1 equiv) (Merck KGaA, Darmstadt, Germany) were dissolved in 0.5 mL of dry DMF, added to the flask, and continuously stirred at room temperature for 1 day. The reaction mixture was mixed with EtOAc, washed with Milli-Q H_2_O, and dried over Na_2_SO_4_. The product was purified by silica gel chromatography using a 1:1 hexane/EtOAc mixture. The organic solvents were removed *in vacuo* yielding a colorless solid (1.07 mg, 76% yield).

Purity: 96.49% as determined by HPLC setup 1, UV detector: 254 nm.

^1^H NMR (600 MHz, CDCl_3_): δ 10.32 (s, 1H), 7.92 (s, 1H), 7.73 (s, 1H), 7.69–7.67 (m, 2H), 7.55 (t, *J* = 7.8 Hz, 1H), 7.45–7.31 (m, 3H), 7.38–7.36 (m, 1H), 7.32–7.28 (m, 4H), 6.62 (s, 1H), 5.21 (s, 2H), 5.20 (s, 2H), 2.27 (s, 3H).

^13^C NMR (151 MHz, CDCl_3_): δ 186.80, 160.68, 160.08, 143.40, 141.67, 137.28, 134.31, 133.69, 132.37, 131.55, 130.77, 130.73, 130.46, 129.97, 129.50, 128.34, 127.71, 127.22, 125.91, 119.54, 118.40, 117.23, 113.35, 98.79, 70.69, 70.00, 16.47.

ESI-MS ([M + Cl]^−^): *m*/*z* calculated ([C_29_H_22_ClNO_3_ + Cl]^−^) = 502.0982 Found = 502.1029.

##### Synthesis of 4-((4-Chloro-5-((3-(2,3-dihydrobenzo[*b*][1,4]dioxin-6-yl)-2-methylbenzyl)oxy)-2-formylphenoxy)methyl)picolinonitrile (**3b**)

5-Chloro-4-((3-(2,3-dihydrobenzo[*b*][1,4]dioxin-6-yl)-2-methylbenzyl)oxy)-2-hydroxybenzaldehyde (**2c**) (50.0 mg, 122 μmol, 1.0 equiv) was mixed with Cs_2_CO_3_ (79.3 mg, 243 μmol, 2.0 equiv) under N_2_ atmosphere in a round-bottom flask. 4-(Bromomethyl)picolinonitrile (50.0 mg, 243 μmol, 2.0 equiv) (abcr GmbH, Karlsruhe, Germany) and cat. KI (0.4 mg, 2.4 μmol, 0.01 equiv) were dissolved in 0.5 mL of dry DMF, added to the flask, and continuously stirred at room temperature for 1 day. The product was purified by silica gel chromatography using a 1:1 hexane/EtOAc mixture. The organic solvents were removed *in vacuo* yielding a colorless solid (41.2 mg, 64% yield).

Purity: 92.80% as determined by HPLC setup 1, UV detector: 254 nm.

^1^H NMR (400 MHz, CDCl_3_): δ 10.33 (s, 1H), 8.77 (d, *J =* 5.0 Hz, 1H), 7.93 (s, 1H), 7.77 (s, 1H), 7.61 (d, *J =* 4.7 Hz, 1H), 7.36 (dd, *J =* 6.5 Hz, *J* = 2.2 Hz, 1H), 7.26–7.22 (m, 2H), 6.92 (d, *J =* 8.2 Hz, 1H), 6.81 (d, *J* = 2.0 Hz, 1H), 6.77 (dd, *J* = 8.2 Hz, *J =* 2.0 Hz, 1H), 6.55 (s, 1H), 5.22 (s, 2H), 5.21 (s, 2H), 4.32 (s, 4H), 2.28 (s, 3H).

ESI-MS ([M + Na]^+^): *m*/*z* calculated ([C_30_H_23_ClN_2_O_5_ + Na]^+^) = 549.1188. Found = 549.1176.

##### Synthesis of 3-((4-Chloro-5-((3-(2,3-dihydrobenzo[*b*][1,4]dioxin-6-yl)-2-methylbenzyl)oxy)-2-formylphenoxy)methyl)benzonitrile (**3c**)

5-Chloro-4-((3-(2,3-dihydrobenzo[*b*][1,4]dioxin-6-yl)-2-methylbenzyl)oxy)-2-hydroxybenzaldehyde (**2c**) (100 mg, 243 μmol, 1.0 equiv) was mixed with Cs_2_CO_3_ (159 mg, 487 μmol, 2.0 equiv) under N_2_ atmosphere in a round-bottom flask. 3-(Bromomethyl)benzonitrile (95.4 mg, 487 μmol, 2.0 equiv) and cat. KI (0.8 mg, 4.8 μmol, 0.02 equiv) were dissolved in 0.5 mL of dry DMF, added to the flask, and continuously stirred at room temperature for 1 day. The product was purified by silica gel chromatography using a 1:1 hexane/EtOAc mixture. Organic solvents were removed *in vacuo* yielding a colorless solid (104 mg, 81% yield).

Purity: 96.27% as determined by HPLC setup 1, UV detector: 254 nm.

^1^H NMR (400 MHz, CDCl_3_): δ 10.32 (s, 1H), 7.91 (s, 1H), 7.72 (s, 1H), 7.68 (d, *J =* 8.0 Hz, 2H), 7.54 (t, *J =* 7.8 Hz, 1H), 7.39 (m, 1H), 7.26–7.25 (m, 2H), 6.92 (d, *J =* 8.2 Hz, 1H), 6.81 (d, *J* = 2.0 Hz, 1H), 6.77 (dd, *J =* 8.2 Hz, *J =* 2.0 Hz, 1H), 6.60 (s, 1H), 5.20 (s, 2H), 5.18 (s, 2H), 4.31 (s, 4H), 2.28 (s, 3H).

ESI-MS ([M + Na]^+^): *m*/*z* calculated ([C_31_H_24_ClNO_5_ + Na]^+^) = 548.1235. Found = 548.1223.

##### Synthesis of 5-Chloro-4-((3-(2,3-dihydrobenzo[*b*][1,4]dioxin-6-yl)-2-methylbenzyl)oxy)-2-(oxazol-4-ylmethoxy)benzaldehyde (**3d**)

5-Chloro-4-((3-(2,3-dihydrobenzo[*b*][1,4]dioxin-6-yl)-2-methylbenzyl)oxy)-2-hydroxybenzaldehyde (**2c**) (50.0 mg, 122 μmol, 1.0 equiv) was mixed with Cs_2_CO_3_ (119 mg, 365 μmol, 3.0 equiv) under N_2_ atmosphere in a round-bottom flask. 4-(Chloromethyl)oxazole hydrochloride (37.5 mg, 243 μmol, 2.0 equiv) (BLDpharm, Kaiserslautern, Germany) and cat. KI (0.8 mg, 4.8 μmol, 0.04 equiv) were dissolved in 0.5 mL of dry DMF, added to the flask, and stirred at room temperature for 1 day. The product was purified by silica gel chromatography using a 1:1 hexane/EtOAc mixture. Organic solvents were removed *in vacuo* yielding a yellow-green solid (49.8 mg, 83% yield).

Purity: 97.90% as determined by HPLC setup 1, UV detector: 254 nm.

^1^H NMR (600 MHz, CDCl_3_): δ 10.27 (s, 1H), 7.90 (s, 1H), 7.88 (s, 1H), 7.45–7.44 (m, 1H), 7.26–7.25 (m, 2H), 6.91 (d, *J =* 8.2 Hz, 1H), 6.88 (s, 1H), 6.83 (d, *J* = 2.1 Hz, 1H), 6.77 (dd, *J* = 8.2 Hz, *J =* 2.1 Hz, 1H), 5.26 (s, 2H), 5.16 (s, 2H), 4.31 (s, 4H), 2.30 (s, 3H).

^13^C NMR (151 MHz, CDCl_3_): δ 187.17, 160.98, 160.02, 143.26, 142.88, 142.74, 137.62, 135.88, 135.16, 134.40, 133.88, 130.64, 130.06, 127.46, 125.81, 122.72, 119.56, 118.39, 117.08, 116.95, 99.40, 70.58, 64.61, 64.59, 63.66, 16.46.

ESI-MS ([M + H]^+^): *m*/*z* calculated ([C_27_H_22_ClNO_6_ + H]^+^) = 492.1208. Found = 492.1205.

##### Synthesis of 4-((4-Chloro-2-formyl-5-((2-methyl-3-(1*H*-pyrrol-1-yl)benzyl)oxy)phenoxy)methyl)picolinonitrile (**3e**)

5-Chloro-2-hydroxy-4-((2-methyl-3-(1*H*-pyrrol-1-yl)benzyl)oxy)benzaldehyde (**2d**) (46.0 mg, 135 μmol 1.0 equiv) was mixed with Cs_2_CO_3_ (87.1 mg, 269 μmol, 2.0 equiv) under N_2_ atmosphere in a round-bottom flask. 4-(Bromomethyl)picolinonitrile (52.9 mg, 268 μmol, 2.0 equiv) and cat. KI (0.4 mg, 2.4 μmol, 0.02 equiv) were dissolved in 0.5 mL of dry DMF, added to the flask, and stirred at room temperature for 1 day. The product was purified by silica gel chromatography using a 1:1 hexane/EtOAc mixture. Organic solvents were removed *in vacuo* yielding a colorless solid (49.9 mg, 81% yield).

Purity: 97.79% as determined by HPLC setup 1, UV detector: 254 nm.

^1^H NMR (700 MHz, CDCl_3_): δ 10.34 (s, 1H), 8.78 (d, *J* = 4.9 Hz, 1H), 7.94 (s, 1H), 7.78 (s, 1H), 7.64 (d, *J* = 4.9 Hz, 2H), 7.45 (d, *J* = 7.1 Hz, 1H), 7.32–7.30 (m, 2H), 6.78 (t, *J* = 2.0 Hz, 2H), 6.58 (s, 1H), 6.34 (t, *J* = 2.0 Hz, 2H), 5.25 (s, 2H), 5.20 (s, 2H), 2.17 (s, 3H).

ESI-MS ([M + H]^+^): *m*/*z* calculated ([C_26_H_20_ClN_3_O_3_ + H]^+^) = 458.1266. Found = 458.1260.

##### Synthesis of *N*-(2-(((5-Chloro-6-((2-methyl-[1,1′-biphenyl]-3-yl)methoxy)pyridin-3-yl)methyl)amino)ethyl)acetamide (**4a**)

5-Chloro-6-((2-methyl-[1,1′-biphenyl]-3-yl)methoxy)nicotinaldehyde (**2a**) (10.0 mg, 29.6 μmol, 1.0 equiv), *N*-(2-aminoethyl)acetamide (5.70 μL, 59.2 μmol, 2.0 equiv) (Merck KGaA, Darmstadt, Germany), and acetic acid (AcOH) (10.0 μL, 175 μmol, 5.9 equiv) (Merck KGaA, Darmstadt, Germany) were dissolved in DCM (1 mL) under N_2_ atmosphere and stirred at room temperature for 1 h. Sodium triacetoxyborohydride (NaBH(OAc)_3_) (9.41 mg, 44.4 μmol, 1.5 equiv) (Merck KGaA, Darmstadt, Germany) was added and stirred overnight. The organic solvent was removed *in vacuo*, MeCN (500 μL) and 10 mmol/L sodium phosphate buffer pH 7.4 (500 μL) were added, and the product was isolated by semiprep. HPLC setup 2. The organic solvent was removed *in vacuo*. The remaining solution was adjusted to pH ∼ 8.5 with sat. NaHCO_3_ (Merck KGaA, Darmstadt, Germany) and extracted twice with EtOAc. The combined organic phases were washed twice with Milli-Q H_2_O and dried over Na_2_SO_4_. EtOAc was removed *in vacuo* and dried overnight within a desiccator yielding a colorless solid (5.21 mg, 41% yield).

Purity: 98.05% as determined by HPLC setup 1, UV detector: 254 nm.

^1^H NMR (400 MHz, CDCl_3_): δ 7.62 (d, *J* = 2.0 Hz, 1H), 7.43–7.23 (m, 7H), 7.08 (d, *J* = 1.8 Hz, 1H), 7.01 (t, *J* = 4.8 Hz, 1H), 5.85 (br s, 1H), 5.24 (s, 2H), 3.50 (s, 2H), 3.32 (q, *J* = 5.8 Hz, 2H), 2.70 (t, *J* = 5.9 Hz, 2H), 2.16 (s, 3H), 1.97 (s, 3H).

^13^C NMR (101 MHz, CDCl_3_): δ 170.50, 158.79, 143.59, 141.72, 138.58, 134.25, 134.12, 133.63, 130.32, 129.43, 128.32, 127.67, 127.21, 126.74, 126.15, 117.53, 51.54, 49.79, 48.16, 39.21, 23.46, 16.60.

ESI-MS ([M + H]^+^): *m*/*z* calculated ([C_24_H_26_ClN_3_O_2_ + H]^+^) = 424.1786. Found = 424.1785.

##### Synthesis of (5-Chloro-2-((3-cyanobenzyl)oxy)-4-((2-methyl-[1,1′-biphenyl]-3-yl)methoxy)benzyl)-d-serine (**4b**)

3-((4-Chloro-2-formyl-5-((2-methyl-[1,1′-biphenyl]-3-yl)methoxy)phenoxy)methyl)benzonitrile (**3a**) (10.0 mg, 21.4 μmol, 1.0 equiv), d-serine (4.50 mg, 42.7 μmol, 2.0 equiv) (Merck KGaA, Darmstadt, Germany), and AcOH (14.2 μL, 248 μmol, 12 equiv) were dissolved in DMF (568 μL) and MeOH (142 μL) under N_2_ atmosphere and stirred at room temperature for 1 h. Sodium cyanoborohydride (NaBH_3_CN) (4.03 mg, 64.1 μmol, 3.0 equiv) (Merck KGaA, Darmstadt, Germany) was added and stirred overnight. The reaction mixture was diluted with 10 mmol/L sodium phosphate buffer pH 7.4 (500 μL), filtered through a 0.22 μm Millex-GV filter (Merck KGaA, Darmstadt, Germany), and the product was isolated by semiprep. HPLC setup 2. The organic solvent was removed *in vacuo*. The remaining suspension was centrifuged (21,380*g*, 4 °C), the precipitate was washed with Milli-Q H_2_O, centrifuged again, and dried overnight *in vacuo* within a desiccator yielding a colorless solid (2.06 mg, 18% yield).

Purity: 96.83% as determined by HPLC setup 1, UV detector: 254 nm.

^1^H NMR (600 MHz, DMSO-*d*_6_): δ 8.01 (s, 1H), 7.90 (d, *J* = 7.8 Hz, 1H), 7.89 (d, *J =* 5.1 Hz, 1H), 7.83 (s, *J =* 7.8 Hz, 1H), 7.62 (t, *J =* 7.8 Hz, 1H), 7.49–7.45 (m, 4H), 7.38 (tt, *J =* 7.5 Hz, *J* = 1.2 Hz, 1H), 7.32–7.30 (m, 2H), 7.28 (t, *J* = 7.5 Hz, 1H), 7.21 (dd, *J =* 7.6 Hz, *J =* 1.2 Hz, 1H), 7.12 (s, 1H), 5.30 (m, 2H), 5.26 (s, 2H), 3.95 (s, 2H), 3.67–3.59 (m, 2H), 3.13 (m, 1H), 2.23 (s, 3H).

^13^C NMR (151 MHz, DMSO-*d*_6_): δ 155.92, 154.09, 142.21, 141.27, 138.31, 134.99, 133.98, 132.49, 131.82, 131.16, 131.07, 129.80, 129.17, 128.27, 127.86, 126.99, 125.58, 118.68, 112.88, 111.53, 100.41, 69.64, 69.07, 62.27, 61.01, 44.58, 15.86.

ESI-MS ([M + H]^+^): *m*/*z* calculated ([C_32_H_29_ClN_2_O_5_ + H]^+^) = 557.1838. Found = 557.1836.

##### Synthesis of (5-Chloro-2-((2-cyanopyridin-4-yl)methoxy)-4-((3-(2,3-dihydrobenzo[*b*][1,4] dioxin-6-yl)-2-methylbenzyl)oxy)benzyl)-d-serine (**4c**)

4-((4-Chloro-5-((3-(2,3-dihydrobenzo[*b*][1,4]dioxin-6-yl)-2-methylbenzyl)oxy)-2-formylphenoxy)methyl)picolinonitrile (**3b**) (10 mg, 19.0 μmol, 1.0 equiv), d*-*serine (3.99 mg, 38.0 μmol, 2.0 equiv), and AcOH (14.2 μL, 248 μmol, 13 equiv) were dissolved in DMF (568 μL) and MeOH (142 μL) under N_2_ atmosphere and stirred at room temperature for 1 h. NaBH_3_CN (3.58 mg, 56.9 μmol, 3.0 equiv) was added and stirred overnight. The reaction mixture was diluted with 10 mmol/L sodium phosphate buffer pH 7.4 (500 μL), filtered through 0.22 μm Millex-GV filter, and the product was isolated by semiprep. HPLC setup 2. The organic solvent was removed *in vacuo*. EtOAc was added to the remaining turbid solution. The resulting crystals were filtered, washed with Milli-Q H_2_O, and dried overnight *in vacuo* within a desiccator yielding off-white crystals (1.61 mg, 14% yield).

Purity: 96.55% as determined by HPLC setup 1, UV detector: 254 nm. (DMSO was added to facilitate dissolution).

^1^H NMR (600 MHz, DMSO-*d*_6_): δ 8.76 (d, *J =* 5.1 Hz, 1H), 8.17 (s, 1H), 7.89 (d, *J =* 5.1 Hz, 1H), 7.52 (s, 1H), 7.40 (d, *J =* 7.5 Hz, 1H), 7.22 (t, *J =* 7.5 Hz, 1H), 7.17 (d, *J =* 7.6 Hz, 1H), 7.06 (s, 1H), 6.92 (d, *J* = 8.2 Hz, 1H), 6.77 (d, *J =* 2.1 Hz, 1H), 6.75 (dd, *J* = 8.2 Hz, *J =* 2.1 Hz, 1H), 5.39 (d, *J* = 5.4 Hz, 2H), 5.24 (s, 2H), 4.28 (s, 4H), 4.02 (s, 2H), 3.73–3.61 (m, 2H), 3.19 (m, 1H), 2.23 (s, 3H).

^13^C NMR (151 MHz, DMSO-*d*_6_): δ 155.62, 154.19, 151.35, 148.05, 142.97, 142.53, 141.68, 134.84, 134.34, 134.09, 132.81, 131.48, 129.84, 127.59, 126.84, 125.60, 125.45, 122.13, 117.72, 117.49, 116.81, 113.16, 100.25, 69.65, 67.75, 64.11, 64.09, 62.48, 60.95, 44.46, 15.88.

ESI-MS ([M + H]^+^): *m*/*z* calculated ([C_33_H_30_ClN_3_O_7_ + H]^+^) = 616.1845. Found = 616.1848.

##### Synthesis of *N*-(2-((5-Chloro-2-((2-cyanopyridin-4-yl)methoxy)-4-((3-(2,3-dihydrobenzo [*b*][1,4]dioxin-6-yl)-2-methylbenzyl)oxy)benzyl)amino)ethyl)acetamide (**4d**)

4-((4-Chloro-5-((3-(2,3-dihydrobenzo[*b*][1,4]dioxin-6-yl)-2-methylbenzyl)oxy)-2-formylphenoxy)methyl)picolinonitrile (**3b**) (10 mg, 19.0 μmol, 1.0 equiv), *N*-(2-aminoethyl)acetamide (3.64 μL, 38.0 μmol, 2.0 equiv), and AcOH (14.2 μL, 248 μmol, 13 equiv) were dissolved in DCM (1 mL) under N_2_ atmosphere and stirred at room temperature for 1 h. NaBH(OAc)_3_ (6.03 mg, 28.5 μmol, 1.5 equiv) was added and stirred overnight. The organic solvent was removed *in vacuo*, MeCN (500 μL) and 10 mmol/L sodium phosphate buffer pH 7.4 (500 μL) were added and the product was isolated by semiprep. HPLC setup 2. The organic solvent was removed *in vacuo*. The remaining solution was adjusted to pH ∼ 9 with sat. NaHCO_3_ and extracted twice with EtOAc. The combined organic phases were washed with Milli-Q H_2_O and dried over Na_2_SO_4_. The organic solvent was removed *in vacuo* and dried overnight within a desiccator yielding a colorless solid (4.05 mg, 35% yield).

Purity: 96.91% as determined by HPLC setup 1, UV detector: 254 nm.

^1^H NMR (600 MHz, CDCl_3_): δ 8.73 (d, *J =* 5 Hz, 1H), 7.78 (s, 1H), 7.52 (d, *J =* 5.0 Hz, 1H), 7.36 (dd, *J =* 6.3 Hz, *J* = 2.7 Hz, 1H), 7.34 (s, 1H), 7.22 (m, 2H), 6.92 (d, *J =* 8.2 Hz, 1H), 6.80 (d, *J* = 2.1 Hz, 1H), 6.77 (dd, *J* = 8.2 Hz, *J =* 2.1 Hz, 1H), 6.50 (s, 1H), 5.95 (br s, 1H), 5.12 (s, 2H), 5.11 (s, 2H), 4.31 (s, 4H), 3.80 (s, 2H), 3.36 (q, *J* = 5.8 Hz, 2H), 2.78 (t, *J =* 5.9 Hz, 2H), 2.28 (s, 3H), 1.97 (s, 3H).

^13^C NMR (151 MHz, CDCl_3_): δ 170.45, 154.84, 154.22, 151.57, 147.63, 143.24, 142.88, 142.61, 135.19, 134.65, 134.63, 134.26, 131.35, 130.46, 127.51, 126.15, 125.74, 124.33, 122.67, 118.35, 117.22, 117.11, 116.36, 100.56, 70.97, 68.01, 64.62, 64.59, 48.28, 47.61, 39.35, 23.49, 16.45.

ESI-MS ([M + H]^+^): *m*/*z* calculated ([C_34_H_33_ClN_4_O_5_ + H]^+^) = 613.2212. Found = 613.2216.

##### Synthesis of 4-((4-Chloro-5-((3-(2,3-dihydrobenzo[*b*][1,4]dioxin-6-yl)-2-methylbenzyl)oxy)-2-(((2-hydroxyethyl)amino)methyl)phenoxy)methyl)picolinonitrile (**4e**)

4-((4-Chloro-5-((3-(2,3-dihydrobenzo[*b*][1,4]dioxin-6-yl)-2-methylbenzyl)oxy)-2-formylphenoxy)methyl)picolinonitrile (**3b**) (10 mg, 19.0 μmol, 1.0 equiv), 2-aminoethan-1-ol (2.29 μL, 38.0 μmol, 2.0 equiv) (Merck KGaA, Darmstadt, Germany), and AcOH (10.0 μL, 175 μmol, 9.2 equiv) were dissolved in DCM (1 mL) under N_2_ atmosphere and stirred at room temperature for 1 h. NaBH(OAc)_3_ (6.03 mg, 28.5 μmol, 1.5 equiv) was added and stirred overnight. The organic solvent was removed *in vacuo*, MeCN (500 μL), 10 mmol/L sodium phosphate buffer pH 7.4 (500 μL), and DMSO (200 μL) (Merck KGaA, Darmstadt, Germany) were added, centrifuged (21,380*g*), and the product was isolated from the supernatant by semiprep. HPLC setup 2. The organic solvent was removed *in vacuo*. The remaining suspension was extracted with EtOAc. The organic phase was washed with Milli-Q H_2_O and brine and dried over Na_2_SO_4_. The organic solvent was removed *in vacuo* and dried overnight within a desiccator yielding a colorless solid (5.34 mg, 49% yield).

Purity: 97.61% as determined by HPLC setup 1, UV detector: 254 nm.

^1^H NMR (600 MHz, CDCl_3_): δ 8.73 (d, *J =* 5.0 Hz, 1H), 7.79 (s, 1H), 7.53 (d, *J =* 5.0 Hz, 1H), 7.37 (dd, *J =* 6.3 Hz, *J* = 2.9 Hz, 1H), 7.35 (s, 1H), 7.22–7.21 (m, 2H), 6.92 (d, *J =* 8.2 Hz, 1H), 6.81 (d, *J* = 2.1 Hz, 1H), 6.77 (dd, *J* = 8.2 Hz, *J =* 2.1 Hz, 1H), 6.50 (s, 1H), 5.12 (s, 2H), 5.10 (s, 2H), 4.31 (s, 4H), 3.82 (s, 2H), 3.70 (t, *J* = 5.1 Hz, 2H), 2.82 (t, *J =* 5.1 Hz, 2H), 2.28 (s, 3H).

^13^C NMR (151 MHz, CDCl_3_): δ 154.92, 154.23, 151.51, 147.59, 143.24, 142.86, 142.59, 135.20, 134.68, 134.62, 134.26, 131.51, 130.44, 127.52, 126.20, 125.73, 124.37, 122.68, 122.63, 118.35, 117.22, 117.10, 116.31, 100.57, 70.98, 68.02, 64.62, 64.59, 61.11, 50.61, 47.82, 16.44.

ESI-MS ([M + H]^+^): *m*/*z* calculated ([C_32_H_30_ClN_3_O_5_ + H]^+^) = 572.1947. Found = 572.1951.

##### Synthesis of (*S*)-1-(5-Chloro-2-((2-cyanopyridin-4-yl)methoxy)-4-((3-(2,3-dihydrobenzo [*b*][1,4]dioxin-6-yl)-2-methylbenzyl)oxy)benzyl)piperidine-2-carboxylic Acid (**4f**)

4-((4-Chloro-5-((3-(2,3-dihydrobenzo[*b*][1,4]dioxin-6-yl)-2-methylbenzyl)oxy)-2-formylphenoxy)methyl)picolinonitrile (**3b**) (10 mg, 19.0 μmol, 1.0 equiv), (*S*)-piperidine-2-carboxylic acid (9.80 mg, 75.9 μmol, 4.0 equiv) (Thermo Fisher Scientific, Inc., Waltham, USA), and AcOH (10.0 μL, 175 μmol, 9.2 equiv) were dissolved in DMF (568 μL) and MeOH (142 μL) under N_2_ atmosphere and stirred at room temperature for 1 h. NaBH_3_CN (4.77 mg, 75.9 μmol, 4.0 equiv) was added and stirred for 2 days. MeCN (500 μL) and 10 mmol/L sodium phosphate buffer pH 7.4 (500 μL) were added and the product was isolated by semiprep. HPLC setup 2. The organic solvent was removed *in vacuo*. The remaining suspension was extracted with EtOAc. The organic phase was washed with Milli-Q H_2_O and brine and dried over Na_2_SO_4_. The organic solvent was removed *in vacuo* and dried overnight within a desiccator yielding a colorless solid (4.73 mg, 39% yield).

Purity: 100.00% as determined by HPLC setup 1, UV detector: 254 nm.

^1^H NMR (600 MHz, CDCl_3_): δ 8.72 (d, *J =* 5.0 Hz, 1H), 7.80 (s, 1H), 7.53 (d, *J =* 5.0 Hz, 1H), 7.50 (s, 1H), 7.29 (dd, *J =* 7.6 Hz, *J* = 1.7 Hz, 1H), 7.22 (dd, *J =* 7.6 Hz, *J* = 1.7 Hz, 1H), 7.19 (t, *J =* 7.6 Hz, 1H), 6.91 (d, *J =* 8.2 Hz, 1H), 6.79 (d, *J* = 2.1 Hz, 1H), 6.75 (dd, *J* = 8.2 Hz, *J =* 2.1 Hz, 1H), 6.48 (s, 1H), 5.23 (d, *J =* 14 Hz, 1H), 5.13 (d, *J* = 14 Hz, 1H), 5.10 (s, 2H), 4.31 (s, 4H), 4.31–4.23 (m, 2H), 3.57 (s, 2H), 3.43 (m, 1H), 3.31 (m, 1H), 2.64 (m, 1H), 2.23 (s, 3H), 2.17 (m, 1H), 1.94–1.73 (m, 4H), 1.40 (m, 1H).

^13^C NMR (151 MHz, CDCl_3_): δ 156.35, 156.13, 151.63, 146.88, 143.26, 142.91, 142.69, 135.02, 134.57, 134.27, 134.19, 134.09, 130.60, 127.27, 126.61, 125.78, 124.81, 122.66, 118.34, 117.22, 117.12, 116.29, 99.73, 70.65, 68.64, 65.78, 64.61, 64.58, 53.09, 51.09, 27.50, 22.80, 21.93, 16.42.

ESI-MS ([M + H]^+^): *m*/*z* calculated ([C_36_H_34_ClN_3_O_6_ + H]^+^) = 640.2209. Found = 640.2205.

##### Synthesis of 1-(5-Chloro-2-((2-cyanopyridin-4-yl)methoxy)-4-((3-(2,3-dihydrobenzo [*b*][1,4]dioxin-6-yl)-2-methylbenzyl)oxy)phenyl)-5,8,11,14-tetraoxa-2-azaheptadecan-17-oic Acid (**4g**)

4-((4-Chloro-5-((3-(2,3-dihydrobenzo[*b*][1,4]dioxin-6-yl)-2-methylbenzyl)oxy)-2-formylphenoxy)methyl)picolinonitrile (**3b**) (10 mg, 19.0 μmol, 1.0 equiv), 1-amino-3,6,9,12-tetraoxapentadecan-15-oic acid (NH_2_–PEG_4_-COOH) (10.1 mg, 38.1 μmol, 2.0 equiv) (Quanta BioDesign, Ltd., Plain City, USA), and AcOH (10.0 μL, 175 μmol, 9.2 equiv) were dissolved in DMF (650 μL) and MeOH (500 μL) under N_2_ atmosphere and stirred at room temperature for 1 h. NaBH_3_CN (3.60 mg, 57.3 μmol, 3.0 equiv) was added and stirred for 3 days. AcOH was neutralized with 1 mol/L NaOH (140 μL). MeCN (2000 μL) and 10 mmol/L sodium phosphate buffer pH 7.4 (850 μL) were added and the product was isolated by semiprep. HPLC setup 2. The organic solvent was removed *in vacuo*. The remaining suspension was extracted twice with EtOAc. The organic phase was washed with Milli-Q H_2_O and dried over Na_2_SO_4_. The organic solvent was removed *in vacuo* and dried overnight within a desiccator yielding a colorless solid (6.43 mg, 44% yield).

Purity: 99.15% as determined by HPLC setup 1, UV detector: 254 nm.

^1^H NMR (700 MHz, CDCl_3_): δ 8.74 (d, *J =* 5.0 Hz, 1H), 7.78 (s, 1H), 7.62 (d, *J =* 5.0 Hz, 1H), 7.37 (s, 1H), 7.35–7.34 (m, 1H), 7.23–7.21 (m, 2H), 6.92 (d, *J =* 8.2 Hz, 1H), 6.81 (d, *J =* 2.0 Hz, 1H), 6.77 (dd, *J =* 8.2 Hz, *J =* 5.0 Hz, 1H), 6.49 (s, 1H), 5.13 (s, 1H), 5.11 (s, 1H), 4.31 (s, 4H), 3.96 (s, 2H), 3.74–3.72 (m, 4H), 3.64–3.56 (m, 12H), 2.90 (m, 2H), 2.45 (t, *J =* 5.6 Hz, 2H), 2.27 (s, 3H).

^13^C NMR (176 MHz, CDCl_3_): δ 176.09, 155.45, 155.16, 151.72, 147.32, 143.24, 142.88, 142.63, 135.15, 134.54, 134.45, 134.26, 132.76, 130.52, 127.45, 126.14, 125.76, 124.60, 122.68, 118.36, 117.16, 117.11, 116.20, 100.04, 70.83, 70.81, 70.77, 70.75, 70.66, 70.57, 70.40, 68.18, 67.68, 64.62, 64.59, 47.07, 46.58, 36.80, 16.44.

ESI-MS ([M + H]^+^): *m*/*z* calculated ([C_41_H_46_ClN_3_O_10_ + H]^+^) = 776.2944. Found = 776.2940.

##### Synthesis of (*S*)-2-((5-Chloro-2-((2-cyanopyridin-4-yl)methoxy)-4-((3-(2,3-dihydrobenzo [*b*][1,4]dioxin-6-yl)-2-methylbenzyl)oxy)benzyl)amino)hex-5-ynoic Acid (**4h**)

4-((4-Chloro-5-((3-(2,3-dihydrobenzo[*b*][1,4]dioxin-6-yl)-2-methylbenzyl)oxy)-2-formylphenoxy)methyl)picolinonitrile (**3b**) (10 mg, 19.0 μmol, 1.0 equiv), (*S*)-2-aminohex-5-ynoic acid hydrochloride (6.21 mg, 38.0 μmol, 2.0 equiv) (Jena Bioscience GmbH, Jena, Germany), and AcOH (14.2 μL, 248 μmol, 13 equiv) were dissolved in DMF (568 μL) and MeOH (142 μL) under N_2_ atmosphere and stirred at room temperature for 1 h. NaBH_3_CN (3.58 mg, 57.0 μmol, 3.0 equiv) was added and stirred for 3 days. MeCN (500 μL) and 10 mmol/L sodium phosphate buffer pH 7.4 (500 μL) were added and the product was isolated by semiprep. HPLC setup 2. The organic solvent was removed *in vacuo*. The remaining suspension was extracted twice with EtOAc. The organic phase was washed with Milli-Q H_2_O and dried over Na_2_SO_4_. The organic solvent was removed *in vacuo* and dried overnight within a desiccator yielding a colorless solid (7.24 mg, 60% yield).

Purity: 98.05% as determined by HPLC setup 1, UV detector: 254 nm.

^1^H NMR (700 MHz, DMSO-*d*_6_): δ 8.76 (d, *J =* 5.0 Hz, 1H), 8.14 (s, 1H), 7.86 (d, *J =* 5.0 Hz, 1H), 7.47 (s, 1H), 7.40 (d, *J =* 7.4 Hz, 1H), 7.21 (t, *J =* 7.4 Hz, 1H), 7.16 (d, *J =* 7.4 Hz, 1H), 7.03 (s, 1H), 6.92 (d, *J =* 8.2 Hz, 1H), 6.77 (d, *J =* 2.1 Hz, 1H), 6.74 (dd, *J =* 8.2 Hz, *J =* 2.1 Hz, 1H), 5.37 (m, 2H), 5.22 (s, 2H), 4.28 (s, 4H), 3.89 (d, *J =* 14 Hz, 1H), 3.80 (d, *J =* 14 Hz, 1H), 3.15 (t, *J =* 6.4 Hz, 1H), 2.71 (t, *J =* 2.6 Hz, 1H), 2.34–2.29 (m, 1H), 2.23–2.19 (m, 1H), 2.22 (s, 3H), 1.84–1.79 (m, 1H), 1.75–1.70 (m, 1H).

^13^C NMR (176 MHz, DMSO-*d*_6_): δ 155.35, 153.75, 151.42, 148.24, 143.00, 142.55, 141.69, 134.94, 134.39, 134.10, 132.80, 130.92, 129.83, 127.60, 126.84, 125.56, 125.47, 122.16, 117.75, 117.52, 116.85, 113.19, 100.31, 69.65, 67.95, 67.66, 64.14, 64.12, 59.82, 54.93, 44.58, 39.88, 30.80, 23.27, 22.43, 15.90, 14.85.

ESI-MS ([M + H]^+^): *m*/*z* calculated ([C_36_H_32_ClN_3_O_6_ + H]^+^) = 638.2052. Found = 638.2045.

##### Synthesis of *N*^2^-(5-Chloro-2-((2-cyanopyridin-4-yl)methoxy)-4-((3-(2,3-dihydrobenzo[*b*][1,4]dioxin-6-yl)-2-methylbenzyl)oxy)benzyl)-*N*^6^-((prop-2-yn-1-yloxy)carbonyl)-l-lysine (**4i**)

4-((4-Chloro-5-((3-(2,3-dihydrobenzo[*b*][1,4]dioxin-6-yl)-2-methylbenzyl)oxy)-2-formylphenoxy)methyl)picolinonitrile (**3b**) (10 mg, 19.0 μmol, 1.0 equiv), *N*-ε-propargyloxycarbonyl-l-lysine hydrochloride (10.0 mg, 38.0 μmol, 2.0 equiv) (MedChemExpress, Monmouth Junction, USA), and AcOH (14.2 μL, 248 μmol, 13 equiv) were dissolved in DMF (568 μL) and MeOH (142 μL) under N_2_ atmosphere and stirred at room temperature for 1 h. NaBH_3_CN (3.58 mg, 57.0 μmol, 3.0 equiv) was added and stirred for 3 days. MeCN (500 μL) and 10 mmol/L sodium phosphate buffer pH 7.4 (500 μL) were added and the product was isolated by semiprep. HPLC setup 2. The organic solvent was removed *in vacuo*. The remaining suspension was extracted twice with EtOAc. The organic phase was washed with Milli-Q H_2_O and dried over Na_2_SO_4_. The organic solvent was removed *in vacuo* and dried overnight within a desiccator yielding a colorless solid (9.04 mg, 64% yield).

Purity: 97.79% as determined by HPLC setup 1, UV detector: 254 nm.

^1^H NMR (700 MHz, DMSO-*d*_6_): δ 8.76 (d, *J =* 5.0 Hz, 1H), 8.15 (s, 1H), 7.86 (d, *J =* 5.0 Hz, 1H), 7.47 (s, 1H), 7.40 (d, *J =* 7.5 Hz, 1H), 7.21 (t, *J =* 7.5 Hz, 1H), 7.16 (d, *J =* 7.5 Hz, 1H), 7.04 (s, 1H), 6.92 (d, *J =* 8.2 Hz, 1H), 6.78 (d, *J =* 2.1 Hz, 1H), 6.74 (dd, *J =* 8.2 Hz, *J =* 2.1 Hz, 1H), 5.37 (m, 2H), 5.23 (s, 2H), 4.58 (d, *J =* 2.3 Hz, 2H), 4.28 (s, 4H), 3.90 (d, *J =* 14 Hz, 1H), 3.82 (d, *J =* 14 Hz, 1H), 3.44 (t, *J =* 2.3 Hz, 1H), 3.10 (t, *J =* 6.4 Hz, 1H), 2.94–2.91 (m, 2H), 2.34–2.29 (m, 1H), 2.23 (s, 3H), 1.64–1.59 (m, 1H), 1.58–1.55 (m, 1H), 1.35–1.30 (m, 4H).

^13^C NMR (176 MHz, DMSO-*d*_6_): δ 155.39, 155.20, 153.83, 151.35, 148.18, 142.96, 142.52, 141.66, 134.88, 134.34, 134.05, 132.78, 131.05, 129.80, 127.55, 126.80, 126.25, 125.54, 125.43, 124.10, 122.11, 117.72, 117.70, 117.46, 116.81, 116.80, 113.13, 100.29, 100.27, 76.97, 69.63, 67.65, 64.10, 64.08, 60.87, 51.31, 44.55, 40.19, 31.17, 30.37, 29.20, 28.96, 28.68, 22.63, 22.08, 15.86.

ESI-MS ([M + H]^+^): *m*/*z* calculated ([C_40_H_39_ClN_4_O_8_ + H]^+^) = 739.2529. Found = 739.2532.

##### Synthesis of *N*-(2-((5-Chloro-2-((3-cyanobenzyl)oxy)-4-((3-(2,3-dihydrobenzo[*b*][1,4]dioxin-6-yl)-2-methylbenzyl)oxy)benzyl)amino)ethyl)acetamide (**4j**)

3-((4-Chloro-5-((3-(2,3-dihydrobenzo[*b*][1,4]dioxin-6-yl)-2-methylbenzyl)oxy)-2-formylphenoxy)methyl)benzonitrile (**3c**) (10 mg, 19.0 μmol, 1.0 equiv), *N*-(2-aminoethyl)acetamide (3.64 μL, 38.0 μmol, 2.0 equiv), and AcOH (10.0 μL, 175 μmol, 9.2 equiv) were dissolved in DCM (1 mL) under N_2_ atmosphere and stirred at room temperature for 1 h. NaBH(OAc)_3_ (6.04 mg, 28.5 μmol, 1.5 equiv) was added and stirred overnight. The organic solvent was removed *in vacuo*, THF (100 μL), MeCN (800 μL), and 10 mmol/L sodium phosphate buffer pH 7.4 (500 μL) were added and the product was isolated by semiprep. HPLC setup 2. The organic solvent was removed *in vacuo*. The remaining turbid solution was extracted twice with EtOAc. The combined organic phases were washed with Milli-Q H_2_O and brine and dried over Na_2_SO_4_. The organic solvent was removed *in vacuo* and dried overnight within a desiccator yielding a colorless solid (4.99 mg, 43% yield).

Purity: 98.21% as determined by HPLC setup 1, UV detector: 254 nm.

^1^H NMR (600 MHz, CDCl_3_): δ 7.72 (s, 1H), 7.63 (m, 2H), 7.52 (t, *J =* 7.8 Hz, 1H), 7.40 (m, 1H), 7.30 (s, 1H), 7.23–7.22 (m, 2H), 6.91 (d, *J* = 8.2 Hz, 1H), 6.81 (d, *J* = 2.1 Hz, 1H), 6.77 (dd, *J =* 8.2 Hz, *J =* 2.1 Hz, 1H), 6.57 (s, 1H), 5.97 (br s, 1H), 5.11 (s, 2H), 5.07 (s, 2H), 4.31 (s, 4H), 3.76 (s, 2H), 3.33 (q, *J* = 5.9 Hz, 2H), 2.74 (t, *J =* 5.9 Hz, 2H), 2.28 (s, 3H), 1.96 (s, 3H).

^13^C NMR (151 MHz, CDCl_3_): δ 170.37, 155.45, 154.17, 143.22, 142.84, 142.56, 138.26, 135.28, 134.76, 134.29, 132.00, 131.36, 131.18, 130.65, 130.40, 129.81, 127.56, 125.72, 122.70, 122.52 118.64, 118.38, 117.07, 115.71, 113.15, 100.61, 70.86, 69.49, 64.61, 64.59, 48.05, 47.79, 39.27, 23.47, 16.42.

ESI-MS ([M + H]^+^): *m*/*z* calculated ([C_35_H_34_ClN_3_O_5_ + H]^+^) = 612.2260. Found = 612.2266.

##### Synthesis of *N*-(2-((5-Chloro-4-((3-(2,3-dihydrobenzo[*b*][1,4]dioxin-6-yl)-2-methylbenzyl)oxy)-2-(oxazol-4-ylmethoxy)benzyl)amino)ethyl)acetamide (**4k**)

5-Chloro-4-((3-(2,3-dihydrobenzo[*b*][1,4]dioxin-6-yl)-2-methylbenzyl)oxy)-2-(oxazol-4-ylmethoxy) benzaldehyde (**3d**) (10 mg, 20.3 μmol, 1.0 equiv), *N*-(2-aminoethyl)acetamide (3.90 μL, 40.7 μmol, 2.0 equiv), and AcOH (10.0 μL, 175 μmol, 8.6 equiv) were dissolved in DCM (1 mL) under N_2_ atmosphere and stirred at room temperature for 1 h. NaBH(OAc)_3_ (8.62 mg, 40.7 μmol, 2.0 equiv) was added and stirred overnight. The organic solvent was removed *in vacuo*, MeCN (500 μL) and 10 mmol/L sodium phosphate buffer pH 7.4 (500 μL) were added, filtered through a 0.22 μm Millex-GV filter, and the product was isolated by semiprep. HPLC setup 2. The organic solvent was removed *in vacuo*. The remaining turbid solution was extracted twice with EtOAc. The combined organic phases were washed with Milli-Q H_2_O and dried over Na_2_SO_4_. The organic solvent was removed *in vacuo* and dried overnight within a desiccator yielding an off-white solid (5.85 mg, 50% yield).

Purity: 98.62% as determined by HPLC setup 1, UV detector: 254 nm.

^1^H NMR (600 MHz, CDCl_3_): δ 7.90 (s, 1H), 7.62 (s, 1H), 7.45–7.43 (m, 1H), 7.24–7.22 (m, 3H), 6.91 (d, *J =* 8.2 Hz, 1H), 6.83 (d, *J* = 2.1 Hz, 1H), 6.78 (dd, *J* = 8.2 Hz, *J =* 2.1 Hz, 1H), 6.72 (s, 1H), 6.25 (br s, 1H), 5.16 (s, 2H), 5.02 (s, 2H), 4.31 (s, 4H), 3.70 (s, 2H), 3.33 (q, *J =* 5.7 Hz, 2H), 2.70 (t, *J =* 5.7 Hz), 2.30 (s, 3H), 1.97 (s, 1H).

^13^C NMR (151 MHz, CDCl_3_): δ 170.34, 155.68, 154.16, 143.23, 142.82, 142.55, 137.17, 135.35, 134.91, 134.28, 131.33, 130.32, 127.50, 125.73, 122.74, 122.64, 118.40, 117.05, 115.44, 100.84, 70.70, 64.61, 64.59, 62.99, 48.18, 47.86, 39.19, 23.44, 16.40.

ESI-MS ([M + H]^+^): *m*/*z* calculated ([C_31_H_32_ClN_3_O_6_ + H]^+^) = 578.2052. Found = 578.2055.

##### Synthesis of (*S*)-1-(5-Chloro-4-((3-(2,3-dihydrobenzo[*b*][1,4]dioxin-6-yl)-2-methylbenzyl)oxy)-2-(oxazol-4-ylmethoxy)benzyl)piperidine-2-carboxylic Acid (**4l**)

5-Chloro-4-((3-(2,3-dihydrobenzo[*b*][1,4]dioxin-6-yl)-2-methylbenzyl)oxy)-2-(oxazol-4-ylmethoxy) benzaldehyde (**3d**) (10 mg, 20.3 μmol, 1.0 equiv), (*S*)-piperidine-2-carboxylic acid (7.88 mg, 61.0 μmol, 3.0 equiv), and AcOH (10.0 μL, 175 μmol, 8.6 equiv) were dissolved in DMF (560 μL) and MeOH (150 μL) under N_2_ atmosphere and stirred at room temperature for 1 h. NaBH_3_CN (5.11 mg, 81.3 μmol, 4.0 equiv) was added and stirred for 4 days. MeCN (290 μL) and 10 mmol/L sodium phosphate buffer pH 7.4 (1000 μL) were added and the product was isolated by semiprep. HPLC setup 2. The organic solvent was removed *in vacuo*. The remaining suspension was extracted thrice with DEE and twice with EtOAc. The formed precipitate in the aqueous phase was filtered, washed with Milli-Q H_2_O, and dried overnight *in vacuo* within a desiccator yielding an off-white solid (4.12 mg, 34% yield).

Purity: 99.85% as determined by HPLC setup 1, UV detector: 254 nm.

^1^H NMR (600 MHz, DMSO-*d*_6_): δ 8.42 (s, 1H), 8.24 (s, 1H), 7.49 (dd, *J =* 7.6 Hz, *J =* 1.5 Hz, 1H), 7.43 (s, 1H), 7.27 (t, *J =* 7.6 Hz, 1H), 7.18 (dd, *J =* 7.6 Hz, *J =* 1.5 Hz, 1H), 7.18 (s, 1H), 6.92 (d, *J =* 8.2 Hz, 1H), 6.78 (d, *J* = 2.1 Hz, 1H), 6.76 (dd, *J* = 8.2 Hz, *J =* 2.1 Hz, 1H), 5.27 (s, 2H), 5.14 (s, 2H), 4.28 (s, 4H), 3.74 (d, *J =* 14 Hz, 1H), 3.60 (d, *J =* 14 Hz, 1H), 3.12 (m, 1H), 2.88 (m, 1H), 2.29 (m, 1H), 2.25 (s, 3H), 2.00 (m, 1H), 1.81 (m, 1H), 1.68 (m, 1H), 1.49 (m, 3H).

^13^C NMR (151 MHz, DMSO-*d*_6_): δ 155.94, 153.59, 151.74, 142.96, 142.51, 141.65, 137.92, 135.57, 135.10, 134.40, 134.07, 130.97, 129.75, 127.59, 125.49, 122.12, 117.71, 116.80, 112.98, 100.57, 69.61, 64.37, 64.10, 64.08, 62.71, 51.85, 49.26, 28.34, 24.00, 21.77, 15.89.

ESI-MS ([M + H]^+^): *m*/*z* calculated ([C_33_H_33_ClN_2_O_7_ + H]^+^) = 605.2049. Found = 605.2048.

##### Synthesis of (5-Chloro-2-((2-cyanopyridin-4-yl)methoxy)-4-((2-methyl-3-(1*H*-pyrrol-1-yl) benzyl)oxy)benzyl)-d-serine (**4m**)

4-((4-Chloro-2-formyl-5-((2-methyl-3-(1*H*-pyrrol-1-yl)benzyl)oxy)phenoxy)methyl)picolinonitrile (**3e**) (14.3 mg, 31.1 μmol, 1.0 equiv), d*-*serine (6.54 mg, 62.2 μmol, 2.0 equiv), and AcOH (14.2 μL, 248 μmol, 8.0 equiv) were dissolved in DMF (568 μL) and MeOH (142 μL) under N_2_ atmosphere and stirred at room temperature for 1 h. NaBH_3_CN (5.87 mg, 93.4 μmol, 3.0 equiv) was added and stirred for 2 days. The reaction mixture was diluted with 10 mmol/L sodium phosphate buffer pH 7.4 (500 μL), filtered through 0.22 μm Millex-GV filter, and the product was isolated by semiprep. HPLC setup 2. The solvents were removed *in vacuo*. The precipitate was washed thrice with Milli-Q H_2_O and dried overnight *in vacuo* within a desiccator yielding an off-white solid (3.58 mg, 21% yield).

Purity: 99.16% as determined by HPLC setup 1, UV detector: 254 nm.

^1^H NMR (700 MHz, DMSO-*d*_6_): δ 8.76 (d, *J =* 4.9 Hz, 1H), 8.17 (s, 1H), 7.89 (d, *J =* 4.9 Hz, 1H), 7.52 (s, 1H), 7.50 (d, *J =* 7.9 Hz, 1H), 7.31 (t, *J =* 7.9 Hz, 1H), 7.27 (d, *J =* 7.9 Hz, 1H), 7.07 (s, 1H), 6.90 (s, 2H), 6.24 (s, 2H), 5.39 (m, 2H), 5.27 (s, 2H), 4.00 (s, 2H), 3.71–3.61 (m, 2H), 3.17 (m, 1H), 2.12 (s, 3H).

ESI-MS ([M + H]^+^): *m*/*z* calculated ([C_29_H_27_ClN_4_O_5_ + H]^+^) = 547.1743. Found = 547.1745.

##### Synthesis of 4-((4-Chloro-2-(((2-hydroxyethyl)amino)methyl)-5-((2-methyl-3-(1*H*-pyrrol-1-yl)benzyl)oxy)phenoxy)methyl)picolinonitrile (**4n**)

4-((4-Chloro-2-formyl-5-((2-methyl-3-(1*H*-pyrrol-1-yl)benzyl)oxy)phenoxy)methyl)picolinonitrile (**3e**) (10.3 mg, 22.4 μmol, 1.0 equiv), 2-aminoethan-1-ol (3.65 μL, 59.8 μmol, 2.7 equiv), and AcOH (10.0 μL, 175 μmol, 7.8 equiv) were dissolved in DCM (1 mL) under N_2_ atmosphere and stirred at room temperature for 1 h. NaBH(OAc)_3_ (9.30 mg, 43.9 μmol, 2.0 equiv) was added and stirred overnight. The organic solvent was removed *in vacuo*, MeCN (1.5 mL) and 10 mmol/L sodium phosphate buffer pH 7.4 (500 μL) were added, filtered through a 0.22 μm Millex-GV filter, and the product was isolated by semiprep. HPLC setup 2. The organic solvent was removed *in vacuo*. The remaining turbid solution was extracted with EtOAc. The organic phase was washed with Milli-Q H_2_O and dried over Na_2_SO_4_. The organic solvent was removed *in vacuo* and dried overnight within a desiccator yielding an off-white solid (5.73 mg, 51% yield).

Purity: 95.19% as determined by HPLC setup 1, UV detector: 254 nm.

^1^H NMR (700 MHz, CDCl_3_): δ 8.74 (d, *J =* 5.0 Hz, 1H), 7.81 (s, 1H), 7.56 (d, *J =* 5.0 Hz, 1H), 7.46–7.45 (m, 2H), 7.37 (s, 1H), 7.28–7.27 (m, 2H), 6.78 (t, *J =* 2.1 Hz, 2H), 6.54 (s, 1H), 6.33 (t, *J =* 2.1 Hz, 2H), 5.14 (s, 2H), 5.10 (s, 2H), 3.83 (s, 2H), 3.71 (t, *J =* 5.2 Hz, 2H), 2.83 (t, *J =* 5.2 Hz, 2H), 2.17 (s, 3H).

^13^C NMR (176 MHz, CDCl_3_): δ 154.99, 154.06, 151.53, 147.54, 141.42, 135.75, 134.67, 133.15, 131.61, 127.96, 127.33, 126.47, 126.19, 124.35, 123.05, 122.48, 117.21, 116.36, 109.09, 100.40, 70.52, 68.08, 61.15, 50.64, 47.82, 13.76.

ESI-MS ([M + H]^+^): *m*/*z* calculated ([C_28_H_27_ClN_4_O_3_ + H]^+^) = 503.1844. Found = 503.1844.

##### Synthesis of (*S*)-1-(5-Chloro-2-((2-cyanopyridin-4-yl)methoxy)-4-((2-methyl-3-(1*H*-pyrrol-1-yl)benzyl)oxy)benzyl)piperidine-2-carboxylic Acid (**4o**)

4-((4-Chloro-2-formyl-5-((2-methyl-3-(1*H*-pyrrol-1-yl)benzyl)oxy)phenoxy)methyl)picolinonitrile (**3e**) (18.9 mg, 41.2 μmol, 1.0 equiv), (*S*)-piperidine-2-carboxylic acid (23.0 mg, 178 μmol, 4.3 equiv), and AcOH (10.0 μL, 175 μmol, 4.3 equiv) were dissolved in DCM (1 mL) under N_2_ atmosphere and stirred at room temperature for 1 h. NaBH(OAc)_3_ (37.3 mg, 176 μmol, 4.3 equiv) was added and stirred overnight. The organic solvent was removed *in vacuo*, MeCN (1.0 mL) and 10 mmol/L sodium phosphate buffer pH 7.4 (1.0 mL) were added, filtered through a 0.22 μm Millex-GV filter, and the product was isolated by semiprep. HPLC setup 2. The organic solvent was removed *in vacuo*. The remaining turbid solution was extracted with EtOAc. The organic phase was washed with Milli-Q H_2_O and dried over Na_2_SO_4_. The organic solvent was removed *in vacuo* and dried overnight within a desiccator yielding an off-white solid (4.26 mg, 18% yield).

Purity: 99.54% as determined by HPLC setup 1, UV detector: 254 nm.

^1^H NMR (600 MHz, CDCl_3_): δ 8.71 (d, *J =* 5.0 Hz, 1H), 7.84 (s, 1H), 7.57 (d, *J =* 5.0 Hz, 1H), 7.51 (s, 1H), 7.38 (dd, *J =* 7.6 Hz, *J =* 1.6 Hz 1H), 7.29 (dd, *J =* 7.6, *J =* 1.6 Hz, 1H), 7.26 (t, *J =* 7.6 Hz, 1H), 6.77 (t, *J =* 2.1 Hz, 1H), 6.52 (s, 1H), 6.33 (t, *J =* 2.1 Hz, 1H), 5.26 (d, *J =* 13 Hz, 1H), 5.16 (d, *J =* 13 Hz, 1H), 5.05 (s, 1H), 4.30 (d, *J =* 13 Hz, 1H), 4.22 (d, *J =* 13 Hz, 1H), 3.43 (d, *J =* 9.8 Hz, 1H), 3.30 (dd, *J =* 9.8 Hz, *J =* 3.9 Hz, 1H), 2.65 (t, *J =* 11 Hz, 1H), 2.19–2.17 (m, 1H), 2.12 (s, 3H), 1.93–1.87 (m, 1H), 1.83–1.76 (m, 3H), 1.42–1.41 (m, 1H).

^13^C NMR (151 MHz, CDCl_3_): δ 156.40, 155.96, 151.63, 146.83, 141.50, 135.15, 134.62, 134.32, 133.12, 127.79, 127.50, 126.65, 126.53, 124.81, 122.45, 117.20, 116.32, 109.17, 99.56, 70.18, 68.69, 65.61, 53.10, 51.10, 27.47, 22.80, 21.90, 13.77.

ESI-MS ([M + H]^+^): *m*/*z* calculated ([C_32_H_31_ClN_4_O_4_ + H]^+^) = 571.2107. Found = 571.2107.

##### Synthesis of *N*-(5-Chloro-2-((2-cyanopyridin-4-yl)methoxy)-4-((3-(2,3-dihydrobenzo[*b*][1,4] dioxin-6-yl)-2-methylbenzyl)oxy)benzyl)-*N*-methyl-d-serine (**5a**)

4-((4-Chloro-5-((3-(2,3-dihydrobenzo[*b*][1,4]dioxin-6-yl)-2-methylbenzyl)oxy)-2-formylphenoxy)methyl)picolinonitrile (**3b)** (10 mg, 19.0 μmol, 1.0 equiv), (*R*)-3-hydroxy-2-(methylamino)propanoic acid (3.99 mg, 38.0 μmol, 2.0 equiv) (BLDpharm, Kaiserslautern, Germany), and AcOH (21.3 μL, 372 μmol, 20 equiv) were dissolved in DMF (568 μL) and MeOH (142 μL) under N_2_ atmosphere and stirred at room temperature for 1 h. NaBH_3_CN (7.16 mg, 114 μmol, 6.0 equiv) was added and stirred for 3 days. The reaction mixture was diluted with 10 mmol/L sodium phosphate buffer pH 7.4 (1.0 mL), filtered through 0.22 μm Millex-GV filter, and the product was isolated by semiprep. HPLC setup 2. The organic solvent was removed *in vacuo*. The remaining turbid solution was extracted twice with EtOAc. The organic phase was washed with Milli-Q H_2_O and brine and dried over Na_2_SO_4_. The organic solvent was removed *in vacuo* and dried overnight within a desiccator yielding a colorless solid (2.07 mg, 17% yield).

Purity: 98.08% as determined by HPLC setup 1, UV detector: 254 nm.

^1^H NMR (600 MHz, DMSO-*d*_6_): δ 8.76 (d, *J =* 5.1 Hz, 1H), 8.12 (s, 1H), 7.82 (d, *J =* 5.1 Hz, 1H), 7.46 (s, 1H), 7.40 (d, *J =* 7.6 Hz, 1H), 7.22 (t, *J =* 7.6 Hz, 1H), 7.17 (d, *J =* 7.7 Hz, 1H), 7.03 (s, 1H), 6.92 (d, *J* = 8.2 Hz, 1H), 6.78 (d, *J =* 2.1 Hz, 1H), 6.75 (dd, *J* = 8.2 Hz, *J =* 2.1 Hz, 1H), 5.38 (s, 2H), 5.21 (s, 2H), 4.28 (s, 4H), 3.83 (d, *J* = 3.7 Hz, 2H), 3.77–3.62 (m, 2H), 2.34 (s, 3H), 2.23 (s, 3H).

^13^C NMR (151 MHz, DMSO-*d*_6_): δ 171.32, 155.42, 153.49, 151.38, 148.42, 142.97, 142.52, 141.65, 134.95, 134.36, 134.06, 132.76, 131.07, 129.79, 127.57, 126.81, 125.45, 125.44, 122.12, 119.95, 117.71, 117.47, 116.80, 113.32, 100.54, 69.61, 67.59, 67.33, 64.10, 64.08, 59.63, 52.11, 38.01, 15.87.

ESI-MS ([M + H]^+^): *m*/*z* calculated ([C_34_H_32_ClN_3_O_7_ + H]^+^) = 630.2002. Found = 630.1978.

##### Synthesis of Methyl (5-Chloro-2-((2-cyanopyridin-4-yl)methoxy)-4-((3-(2,3-dihydrobenzo[*b*][1,4]dioxin-6-yl)-2-methylbenzyl)oxy)benzyl)-d-serinate (**5b**)

4-((4-Chloro-5-((3-(2,3-dihydrobenzo[*b*][1,4]dioxin-6-yl)-2-methylbenzyl)oxy)-2-formylphenoxy)methyl)picolinonitrile (**3b)** (10 mg, 19.0 μmol, 1.0 equiv), d-serine methyl ester hydrochloride (5.90 mg, 38.0 μmol, 2.0 equiv) (Merck KGaA, Darmstadt, Germany), and AcOH (10.0 μL, 175 μmol, 9.2 equiv) were dissolved in DMF (568 μL) and MeOH (142 μL) under N_2_ atmosphere and stirred at room temperature for 1 h. NaBH_3_CN (2.38 mg, 38.0 μmol, 2.0 equiv) was added and stirred for 3 days. The reaction mixture was diluted with 10 mmol/L sodium phosphate buffer pH 7.4 (1.0 mL), filtered through 0.22 μm Millex-GV filter, and the product was isolated by semiprep. HPLC setup 2. The organic solvent was removed *in vacuo*. The remaining turbid solution was extracted twice with EtOAc. The organic phase was washed with Milli-Q H_2_O and brine and dried over Na_2_SO_4_. The organic solvent was removed *in vacuo* and dried overnight within a desiccator yielding a colorless solid (1.38 mg, 12% yield).

Purity: 99.43% as determined by HPLC setup 1, UV detector: 254 nm.

^1^H NMR (700 MHz, CDCl_3_): δ 8.73 (d, *J =* 5.1 Hz, 1H), 7.87 (s, 1H), 7.57 (d, *J =* 5.1 Hz, 1H), 7.37 (dd, *J =* 6.3 Hz, *J =* 2.8 Hz 1H), 7.33 (s, 1H), 7.23–7.22 (m, 2H), 6.92 (d, *J =* 8.2 Hz, 1H), 6.81 (d, *J =* 2.1 Hz, 1H), 6.77 (dd, *J =* 8.2 Hz, *J =* 2.1 Hz, 1H), 6.51 (s, 1H), 5.12 (s, 2H), 5.12 (d, *J =* 14 Hz, 1H), 5.09 (d, *J =* 14 Hz, 1H), 4.31 (s, 4H), 3.87 (d, *J* = 13 Hz, 1H), 3.81 (dd, *J =* 11 Hz, *J =* 4.5 Hz, 1H), 3.76 (d, *J =* 13 Hz, 1H), 3.71 (s, 3H), 3.63 (dd, *J =* 11 Hz, *J =* 6.5 Hz, 1H), 3.44 (dd, *J =* 6.5 Hz, *J =* 4.5 Hz, 1H), 2.28 (s, 3H).

^13^C NMR (176 MHz, CDCl_3_): δ 155.16, 154.49, 151.47, 147.42, 143.24, 142.86, 142.61, 135.18, 134.67, 134.59, 134.27, 131.86, 130.48, 127.52, 126.30, 125.74, 124.45, 122.68, 118.35, 117.22, 117.10, 116.18, 100.37, 70.93, 68.10, 64.62, 64.59, 62.49, 62.06, 52.50, 47.02, 16.44.

ESI-MS ([M + H]^+^): *m*/*z* calculated ([C_34_H_32_ClN_3_O_7_ + H]^+^) = 630.2002. Found = 630.2006.

##### Synthesis of 4-((4-Chloro-5-((3-(2,3-dihydrobenzo[*b*][1,4]dioxin-6-yl)-2-methylbenzyl)oxy)-2-(((2-hydroxyethyl)(methyl)amino)methyl)phenoxy)methyl)picolinonitrile (**5c**)

4-((4-Chloro-5-((3-(2,3-dihydrobenzo[*b*][1,4]dioxin-6-yl)-2-methylbenzyl)oxy)-2-formylphenoxy)methyl)picolinonitrile (**3b**) (10 mg, 19.0 μmol, 1.0 equiv), 2-(methylamino)ethanol (4.57 μL, 57.0 μmol, 3.0 equiv) (Merck KGaA, Darmstadt, Germany), and AcOH (10.0 μL, 175 μmol, 9.2 equiv) were dissolved in DCM (1 mL) under N_2_ atmosphere and stirred at room temperature for 1 h. NaBH(OAc)_3_ (6.03 mg, 28.5 μmol, 1.5 equiv) was added and stirred for 4 days. The organic solvent was removed *in vacuo*, MeCN (1.0 mL) and 10 mmol/L sodium phosphate buffer pH 7.4 (0.7 mL) were added, centrifuged (21,380 × g), and the product was isolated by semiprep. HPLC setup 2. The organic solvent was removed *in vacuo*. The remaining solution was adjusted to pH ∼ 7.5 with sat. NaHCO_3_. The suspension was centrifuged, and the precipitate was washed with Milli-Q H_2_O and dried *in vacuo* overnight within a desiccator yielding a colorless solid (1.73 mg, 16% yield).

Purity: 96.40% as determined by HPLC setup 1, UV detector: 254 nm.

^1^H NMR (600 MHz, CDCl_3_): δ 8.72 (d, *J =* 5.0 Hz, 1H), 7.80 (s, 1H), 7.53 (d, *J =* 5.0 Hz, 1H), 7.37 (dd, *J =* 6.2 Hz, *J* = 3.0 Hz, 1H), 7.33 (s, 1H), 7.23–7.22 (m, 2H), 6.92 (d, *J =* 8.2 Hz, 1H), 6.81 (d, *J* = 2.1 Hz, 1H), 6.77 (dd, *J* = 8.2 Hz, *J =* 2.1 Hz, 1H), 6.50 (s, 1H), 5.11 (s, 2H), 5.10 (s, 2H), 4.31 (s, 4H), 3.62 (t, *J* = 5.3 Hz, 2H), 3.57 (s, 2H), 2.64 (t, *J =* 5.3 Hz, 2H), 2.28 (s, 3H), 2.27 (s, 3H).

^13^C NMR (151 MHz, CDCl_3_): δ 155.28, 154.36, 151.47, 147.67, 143.24, 142.86, 142.59, 135.21, 134.67, 134.61, 134.25, 132.53, 130.44, 127.49, 126.21, 125.74, 124.38, 122.69, 118.36, 117.20, 117.10, 116.18, 100.56, 70.91, 68.10, 64.62, 64.59, 58.70, 58.58, 55.98, 41.86, 16.44.

ESI-MS ([M + H]^+^): *m*/*z* calculated ([C_33_H_32_ClN_3_O_5_ + H]^+^) = 586.2103. Found = 586.2095.

##### Synthesis of 4-((4-Chloro-5-((3-(2,3-dihydrobenzo[*b*][1,4]dioxin-6-yl)-2-methylbenzyl)oxy)-2-(((2-methoxyethyl)amino)methyl)phenoxy)methyl)picolinonitrile (**5d**)

4-((4-Chloro-5-((3-(2,3-dihydrobenzo[*b*][1,4]dioxin-6-yl)-2-methylbenzyl)oxy)-2-formylphenoxy)methyl)picolinonitrile (**3b)** (10 mg, 19.0 μmol, 1.0 equiv), 2-methoxyethylamine (3.28 μL, 38.0 μmol, 2.0 equiv) (Merck KGaA, Darmstadt, Germany), and AcOH (10.0 μL, 175 μmol, 9.2 equiv) were dissolved in DCM (1 mL) under N_2_ atmosphere and stirred at room temperature for 1 h. NaBH(OAc)_3_ (8.04 mg, 38.0 μmol, 2.0 equiv) was added and stirred overnight. The organic solvent was removed *in vacuo*, MeCN (750 μL) and 10 mmol/L sodium phosphate buffer pH 7.4 (750 μL) were added, and the product was isolated by semiprep. HPLC setup 2. The organic solvent was removed *in vacuo*. The remaining turbid solution was extracted twice with EtOAc. The organic phase was washed with Milli-Q H_2_O and dried over Na_2_SO_4_. The organic solvent was removed *in vacuo* and dried overnight within a desiccator yielding a colorless solid (1.82 mg, 16% yield).

Purity: 99.70% as determined by HPLC setup 1, UV detector: 254 nm.

^1^H NMR (700 MHz, CDCl_3_): δ 8.72 (d, *J =* 5.0 Hz, 1H), 7.79 (s, 1H), 7.56 (d, *J =* 5.0 Hz, 1H), 7.37 (dd, *J =* 6.2 Hz, *J* = 3.0 Hz, 1H), 7.36 (s, 1H), 7.22–7.21 (m, 2H), 6.92 (d, *J =* 8.2 Hz, 1H), 6.81 (d, *J* = 2.1 Hz, 1H), 6.77 (dd, *J* = 8.2 Hz, *J =* 2.1 Hz, 1H), 6.50 (s, 1H), 5.12 (s, 2H), 5.09 (s, 2H), 4.32 (s, 4H), 3.80 (s, 2H), 3.53 (t, *J* = 5.1 Hz, 2H), 3.34 (s, 3H), 2.81 (t, *J =* 5.1 Hz, 2H), 2.28 (s, 3H).

^13^C NMR (176 MHz, CDCl_3_): δ 154.94, 154.07, 151.48, 147.71, 143.23, 142.85, 142.56, 135.23, 134.75, 134.58, 134.24, 131.52, 130.41, 127.53, 126.17, 125.73, 124.43, 123.09, 122.69, 118.36, 117.18, 117.09, 116.28, 100.62, 72.18, 70.98, 68.02, 64.62, 64.59, 59.02, 49.08, 48.47, 16.43.

ESI-MS ([M + H]^+^): *m*/*z* calculated ([C_33_H_32_ClN_3_O_5_ + H]^+^) = 586.2103. Found = 586.2103.

##### Synthesis of 3-((2-((3-Acetyl-2-oxoimidazolidin-1-yl)methyl)-4-chloro-5-((3-(2,3-dihydrobenzo[*b*][1,4]dioxin-6-yl)-2-methylbenzyl)oxy)phenoxy)methyl)benzonitrile (**5e**)

**5e** was obtained as a byproduct in the synthesis of 2-fluoroethyl (2-acetamidoethyl)(5-chloro-2-((3-cyanobenzyl)oxy)-4-((3-(2,3-dihydrobenzo[*b*][1,4]dioxin-6-yl)-2-methylbenzyl)oxy)benzyl)carbamate (**5j)** from *N*-(2-((5-chloro-2-((3-cyanobenzyl)oxy)-4-((3-(2,3-dihydrobenzo[*b*][1,4]dioxin-6-yl)-2-methylbenzyl)oxy)benzyl)amino)ethyl)acetamide (**4j**) (3.00 mg, 4.90 μmol, 1.0 equiv) and Cs_2_CO_3_ (3.19 mg, 9.80 μmol, 2.0 equiv) by stirring at 50 °C in DMSO (0.5 mL) for 1 day. Product was isolated by semiprep. HPLC setup 2 (*vide infra*) and obtained as a colorless solid (1.26 mg, 40% yield).

Purity: 98.32% as determined by HPLC setup 1, UV detector: 254 nm.

^1^H NMR (600 MHz, CDCl_3_): δ 7.72 (s, 1H), 7.64 (m, 2H), 7.51 (t, *J =* 7.8 Hz, 1H), 7.40 (m, 1H), 7.29 (s, 1H), 7.24–7.23 (m, 2H), 6.91 (d, *J* = 8.2 Hz, 1H), 6.81 (d, *J* = 2.1 Hz, 1H), 6.77 (dd, *J =* 8.2 Hz, *J =* 2.1 Hz, 1H), 6.60 (s, 1H), 5.13 (s, 2H), 5.06 (s, 2H), 4.45 (s, 2H), 4.31 (s, 4H), 3.79 (t, *J* = 8.2 Hz, 2H), 3.29 (t, *J =* 8.2 Hz, 2H), 2.51 (s, 3H), 2.28 (s, 3H).

^13^C NMR (151 MHz, CDCl_3_): δ 170.99, 155.64, 154.92, 154.85, 143.24, 142.87, 142.64, 137.86, 135.21, 134.56, 134.34, 132.10, 131.48, 131.46, 130.72, 130.50, 129.75, 127.58, 125.76, 124.92, 122.68, 118.53, 118.37, 118.07, 117.09, 116.05, 113.20, 100.28, 70.82, 69.66, 64.62, 64.59, 42.11, 40.85, 39.57, 23.51, 16.45, 14.35.

ESI-MS ([M + H]^+^): *m*/*z* calculated ([C_36_H_32_ClN_3_O_6_ + H]^+^) = 638.2052. Found = 638.2055.

##### Synthesis of 4-((4-Chloro-2-(((2-hydroxyethyl)(methyl)amino)methyl)-5-((2-methyl-3-(1*H*-pyrrol-1-yl)benzyl)oxy)phenoxy)methyl)picolinonitrile (**5f**)

4-((4-Chloro-2-formyl-5-((2-methyl-3-(1*H*-pyrrol-1-yl)benzyl)oxy)phenoxy)methyl)picolinonitrile (**3e**) (10.1 mg, 22.1 μmol, 1.0 equiv), 2-(methylamino)ethanol (5.20 μL, 64.7 μmol, 2.9 equiv), and AcOH (10.0 μL, 175 μmol, 7.9 equiv) were dissolved in DCM (1 mL) under N_2_ atmosphere and stirred at room temperature for 1 h. NaBH(OAc)_3_ (9.30 mg, 43.9 μmol, 2.0 equiv) was added and stirred for 2 days. The organic solvent was removed *in vacuo*, MeCN (1.0 mL) and 10 mmol/L sodium phosphate buffer pH 7.4 (1.6 mL) were added, centrifuged (21,380*g*), filtered through a 0.22 μm Millex-GV filter, and the product was isolated by semiprep. HPLC setup 2. The organic solvent was removed *in vacuo*. The remaining turbid solution was extracted twice with EtOAc. The organic phase was washed with Milli-Q H_2_O and dried over Na_2_SO_4_. The organic solvent was removed *in vacuo* and dried overnight within a desiccator yielding an off-white solid (2.95 mg, 26% yield).

Purity: 95.01% as determined by HPLC setup 1, UV detector: 254 nm.

^1^H NMR (700 MHz, CDCl_3_): δ 8.73 (d, *J =* 5.0 Hz, 1H), 7.81 (s, 1H), 7.56 (d, *J =* 5.0 Hz, 1H), 7.46 (dd, *J =* 6.4 Hz, *J =* 2.6 Hz, 1H), 7.34 (s, 1H), 7.29–7.28 (m, 2H), 6.78 (t, *J =* 2.1 Hz, 2H), 6.53 (s, 1H), 6.33 (t, *J =* 2.1 Hz, 2H), 5.14 (s, 2H), 5.10 (s, 2H), 3.62 (t, *J =* 5.2 Hz, 2H), 3.58 (s, 2H), 2.64 (t, *J =* 5.2 Hz, 2H), 2.28 (s, 3H), 2.17 (s, 3H).

^13^C NMR (176 MHz, CDCl_3_): δ 155.36, 154.22, 151.49, 147.61, 141.42, 135.73, 134.65, 133.15, 132.64, 127.95, 127.33, 126.48, 126.21, 124.37, 122.48, 117.19, 116.23, 109.09, 100.39, 70.44, 68.17, 58.70, 58.56, 56.00, 41.88, 13.77.

ESI-MS ([M + H]^+^): *m*/*z* calculated ([C_29_H_29_ClN_4_O_3_ + H]^+^) = 517.2001. Found = 517.1999.

##### Synthesis of 4-((4-Chloro-5-((3-(2,3-dihydrobenzo[*b*][1,4]dioxin-6-yl)-2-methylbenzyl)oxy)-2-((2-(fluoromethyl)oxazolidin-3-yl)methyl)phenoxy)methyl)picolinonitrile (**5g**)

4-((4-Chloro-5-((3-(2,3-dihydrobenzo[*b*][1,4]dioxin-6-yl)-2-methylbenzyl)oxy)-2-(((2-hydroxyethyl) amino)methyl)phenoxy)methyl)picolinonitrile (**4e**) (2.00 mg, 3.50 μmol, 1.0 equiv), 2-fluoroethyl *p*-toluenesulfonate (1.77 μL, 10.5 μmol, 3.0 equiv) (TCI Deutschland GmbH, Eschborn, Germany), and *N*,*N*-diisopropylethylamine (DIPEA) (1.78 μL, 10.5 μmol, 3.0 equiv) (Merck KGaA, Darmstadt, Germany) were stirred at 50 °C in DMSO (0.5 mL) under N_2_ atmosphere for 4 days. The reaction mixture was diluted with 10 mmol/L sodium phosphate buffer pH 7.4 (500 μL) and the product was isolated by semiprep. HPLC setup 2. The organic solvent was removed *in vacuo*. The remaining turbid solution was extracted twice with EtOAc. The organic phase was washed with Milli-Q H_2_O and dried over Na_2_SO_4_. The organic solvent was removed *in vacuo* and dried overnight within a desiccator yielding a colorless solid (0.965 mg, 45% yield).

Purity: 96.57% as determined by HPLC setup 1, UV detector: 254 nm.

^1^H NMR (700 MHz, CDCl_3_): δ 8.72 (d, *J =* 5.0 Hz, 1H), 7.82 (s, 1H), 7.56 (d, *J =* 5.0 Hz, 1H), 7.43 (s, 1H), 7.37 (dd, *J =* 6.3 Hz, *J* = 2.8 Hz, 1H), 7.23–7.22 (m, 2H), 6.92 (d, *J =* 8.2 Hz, 1H), 6.81 (d, *J* = 2.1 Hz, 1H), 6.77 (dd, *J* = 8.2 Hz, *J =* 2.1 Hz, 1H), 6.51 (s, 1H), 5.12 (s, 2H), 5.12 (d, *J =* 14 Hz, 1H), 5.07 (d, *J =* 14 Hz, 1H), 4.59 (dt, *J =* 11 Hz, *J =* 4.8 Hz, 1H), 4.32 (s, 4H), 4.25 (ddd, *J =* 47 Hz, *J =* 9.8 Hz, *J =* 4.7 Hz, 1H), 4.22 (ddd, *J =* 47 Hz, *J =* 9.8 Hz, *J =* 4.7 Hz, 1H), 3.94–3.92 (m, 2H), 3.93 (d, *J =* 13 Hz, 1H), 3.69 (d, *J =* 13 Hz, 1H), 3.18 (dt, *J* = 10 Hz, *J =* 6.1 Hz, 1H), 2.83 (dt, *J =* 10 Hz, *J =* 6.1 Hz, 2H), 2.28 (s, 3H).

^19^F-{^1^H}-NMR (659 MHz, CDCl_3_): δ −226.70 (s, 1F).

ESI-MS ([M + H]^+^): *m*/*z* calculated ([C_34_H_31_ClFN_3_O_5_ + H]^+^) = 616.2009. Found = 616.2018.

##### Synthesis of 2-Fluoroethyl (*S*)-1-(5-Chloro-2-((2-cyanopyridin-4-yl)methoxy)-4-((3-(2,3-dihydrobenzo[*b*][1,4]dioxin-6-yl)-2-methylbenzyl)oxy)benzyl)piperidine-2-carboxylate (**5h**)

(*S*)-1-(5-Chloro-2-((2-cyanopyridin-4-yl)methoxy)-4-((3-(2,3-dihydrobenzo[*b*][1,4]dioxin-6-yl)-2-methylbenzyl)oxy)benzyl)piperidine-2-carboxylic acid (**4f**) (1.00 mg, 1.56 μmol, 1.0 equiv), 2-fluoroethyl *p*-toluenesulfonate (0.79 μL, 4.69 μmol, 3.0 equiv), and Cs_2_CO_3_ (1.53 mg, 4.69 μmol, 3.0 equiv) were stirred at 100 °C in DMSO (0.5 mL) under N_2_ atmosphere for 10 min. The reaction mixture was diluted with 10 mmol/L sodium phosphate buffer pH 7.4 (500 μL) and the product was isolated by semiprep. HPLC setup 2. The organic solvent was removed *in vacuo*. The remaining turbid solution was extracted with EtOAc. The organic phase was washed with Milli-Q H_2_O and dried over Na_2_SO_4_. The organic solvent was removed *in vacuo* and dried overnight within a desiccator yielding a colorless solid (0.443 mg, 40% yield).

Purity: 95.00% as determined by HPLC setup 1, UV detector: 254 nm.

^1^H NMR (600 MHz, DMSO-*d*_6_): δ 8.77 (d, *J =* 5.0 Hz, 1H), 8.11 (s, 1H), 7.80 (d, *J =* 5.0 Hz, 1H), 7.41 (d, *J =* 7.6 Hz, 1H), 7.35 (s, 1H), 7.22 (t, *J =* 7.6 Hz), 7.17 (d, *J =* 7.6 Hz), 7.03 (s, 1H), 6.92 (d, *J =* 8.2 Hz, 1H), 6.78 (d, *J* = 2.1 Hz, 1H), 6.75 (dd, *J* = 8.2 Hz, *J =* 2.1 Hz, 1H), 5.36 (s, *J =* 6.2 Hz, 1H), 5.21 (s, 1H), 4.60 (t, *J =* 48 Hz, *J* = 4.1 Hz, 1H), 4.34–4.26 (m, 2H), 4.28 (s, 4H), 3.68 (d, *J =* 14 Hz, 1H), 3.54 (d, *J =* 14 Hz, 1H), 2.86 (m, 1H), 2.27 (m, 1H), 2.23 (s, 3H), 1.76 (m, 2H), 1.47–1.41 (m, 4H).

^13^C NMR (151 MHz, DMSO-*d*_6_): δ 172.79, 155.30, 153.23, 151.34, 148.53, 142.97, 142.52, 141.65, 134.99, 134.37, 134.07, 132.77, 130.74, 129.79, 127.60, 126.67, 125.44, 125.39, 122.13, 120.56, 117.72, 117.47, 116.81, 113.24, 100.48, 81.15 (*J* = 165 Hz), 69.60, 67.45, 64.11, 64.09, 63.03 (*J* = 19 Hz), 62.86, 52.85, 48.82, 28.92, 25.00, 21.47, 15.88.

^19^F-{^1^H}-NMR (376 MHz, DMSO-*d*_6_): δ −223.34 (s, 1F).

ESI-MS ([M + H]^+^): *m*/*z* calculated ([C_38_H_37_ClFN_3_O_6_ + H]^+^) = 686.2428. Found = 686.2426.

##### Synthesis of 4-((4-Chloro-5-((3-(2,3-dihydrobenzo[*b*][1,4]dioxin-6-yl)-2-methylbenzyl)oxy)-2-(((2-fluoroethyl)amino)methyl)phenoxy)methyl)picolinonitrile (**5i**)

4-((4-Chloro-5-((3-(2,3-dihydrobenzo[*b*][1,4]dioxin-6-yl)-2-methylbenzyl)oxy)-2-formylphenoxy)methyl)picolinonitrile (**3b)** (10 mg, 19.0 μmol, 1.0 equiv), 2-fluoroethylamine hydrochloride (3.78 mg, 38.0 μmol, 2.0 equiv) (Merck KGaA, Darmstadt, Germany), and AcOH (10.0 μL, 175 μmol, 9.2 equiv) were dissolved in DMF (568 μL) and MeOH (142 μL) under N_2_ atmosphere and stirred at room temperature for 1 h. NaBH_3_CN (2.38 mg, 38.0 μmol, 2.0 equiv) was added and stirred for 3 days. The reaction mixture was diluted with 10 mmol/L sodium phosphate buffer pH 7.4 (1.0 mL) and the product was isolated by semiprep. HPLC setup 2. The organic solvent was removed *in vacuo*. The remaining turbid solution was extracted four times with EtOAc. The organic phase was washed with Milli-Q H_2_O and dried over Na_2_SO_4_. The organic solvent was removed *in vacuo* and dried overnight within a desiccator yielding a colorless solid (2.22 mg, 20% yield).

Purity: 96.82% as determined by HPLC setup 1, UV detector: 254 nm.

^1^H NMR (600 MHz, CDCl_3_): δ 8.72 (d, *J =* 5.1 Hz, 1H), 7.77 (s, 1H), 7.54 (d, *J =* 5.1 Hz, 1H), 7.37 (dd, *J =* 6.0 Hz, *J =* 2.9 Hz, 1H), 7.36 (s, 1H), 7.22–7.21 (m, 2H), 6.92 (d, *J =* 8.2 Hz, 1H), 6.81 (d, *J =* 2.1 Hz, 1H), 6.77 (dd, *J =* 8.2 Hz, *J =* 2.1 Hz, 1H), 6.51 (s, 1H), 5.12 (s, 2H), 5.10 (s, 2H), 4.58 (dt, *J =* 48 Hz, *J =* 4.7 Hz, 2H), 4.32 (s, 4H), 3.84 (s, 2H), 3.95 (dt, *J =* 29 Hz, *J =* 4.7 Hz, 2H), 2.28 (s, 3H).

^13^C NMR (151 MHz, CDCl_3_): δ 154.96, 154.23, 151.53, 147.59, 143.24, 142.86, 142.59, 135.22, 134.69, 134.61, 134.26, 131.50, 130.44, 127.53, 126.15, 125.74, 124.40, 122.68, 122.62, 118.36, 117.16, 117.10, 116.36, 100.65, 84.28, 83.18, 70.98, 68.07, 64.62, 64.59, 49.39, 49.26, 48.22, 16.44.

ESI-MS ([M + H]^+^): *m*/*z* calculated ([C_32_H_29_ClFN_3_O_4_ + H]^+^) = 574.1903. Found = 574.1902.

##### Synthesis of 2-Fluoroethyl (2-acetamidoethyl)(5-chloro-2-((3-cyanobenzyl)oxy)-4-((3-(2,3-dihydrobenzo[*b*][1,4]dioxin-6-yl)-2-methylbenzyl)oxy)benzyl)carbamate (**5j**)

*N*-(2-((5-chloro-2-((3-cyanobenzyl)oxy)-4-((3-(2,3-dihydrobenzo[*b*][1,4]dioxin-6-yl)-2-methylbenzyl)oxy)benzyl)amino)ethyl)acetamide (**4j**) (3.00 mg, 4.90 μmol, 1.0 equiv), 2-fluoroethyl *p*-toluenesulfonate (1.66 μL, 9.80 μmol, 2.0 equiv), and Cs_2_CO_3_ (3.19 mg, 9.80 μmol, 2.0 equiv) were stirring at 50 °C in DMSO (0.5 mL) for 1 day. The reaction mixture was diluted with 10 mmol/L sodium phosphate buffer pH 7.4 (1.0 mL) and the product was isolated by semiprep. HPLC setup 2. The organic solvent was removed *in vacuo*. The remaining turbid solution was extracted twice with EtOAc. The organic phase was washed with Milli-Q H_2_O and dried over Na_2_SO_4_. The organic solvent was removed *in vacuo* and dried overnight within a desiccator yielding a colorless solid (1.78 mg, 52% yield). Compound **5e** was obtained as a byproduct following the same procedure as delineated herein.

Purity: 99.50% as determined by HPLC setup 1, UV detector: 254 nm.

^1^H NMR (600 MHz, DMSO-*d*_6_): δ 7.95 (d, *J =* 11 Hz, 1H), 7.89 (br s, 1H), 7.82 (m, 2H), 7.62 (t, *J =* 7.7 Hz, 1H), 7.44 (d, *J =* 7.5 Hz, 1H), 7.24 (t, *J =* 7.5 Hz, 1H), 7.18 (dd, *J =* 7.7 Hz, *J =* 1.4 Hz, 1H), 7.14 (d, *J =* 5.8 Hz, 1H), 7.12 (s, 1H), 6.92 (d, *J =* 8.2 Hz, 1H), 6.78 (d, *J =* 2.1 Hz, 1H), 6.75 (dd, *J =* 8.2 Hz, *J =* 2.1 Hz, 1H), 5.28 (s, 2H), 5.23 (s, 2H), 4.56 (m, *J =* 45 Hz, 2H), 4.39 (s, 2H), 4.28 (s, 4H), 4.22 (m, *J =* 35 Hz, 2H), 3.21 (m, 2H), 3.13 (m, 2H), 2.24 (s, 3H), 1.74 (s, 3H).

^13^C NMR (151 MHz, DMSO-*d*_6_): δ 169.27, 155.46, 153.58, 142.97, 142.52, 141.66, 134.99, 134.37, 134.06, 132.42, 131.84 (*J =* 13 Hz), 131.15 (*J =* 25 Hz), 129.80, 129.76, 129.48, 129.30, 127.61, 125.49, 122.13, 119.25, 118.66, 117.71, 116.81, 112.92, 111.54, 100.53, 81.97 (*J =* 165 Hz), 81.47, 69.67, 68.91, 64.22 (*J =* 16 Hz), 64.11, 64.08, 45.87 (*J =* 82 Hz), 44.89 (*J =* 27 Hz), 36.76 (*J =* 64 Hz), 22.44, 15.88.

^19^F-NMR (565 MHz, DMSO-*d*_6_): δ −223.10 to −223.27 (m, 1F).

ESI-MS ([M + H]^+^): *m*/*z* calculated ([C_38_H_37_ClFN_3_O_7_ + H]^+^) = 702.2377. Found = 702.2363.

### Lipophilicity and Calculated Physicochemical Properties

The measurements of lipophilicity of precursors and products were based on the HPLC method of Donovan and Pescatore^36^ and performed according to and compared to the published database of Vraka et al.^37^ An internal standard mixture consisting of 1% v/v toluene (Merck KGaA, Darmstadt, Germany) and 0.438 mmol/L triphenylene (Merck KGaA, Darmstadt, Germany) in MeOH was added to sample solutions of approximately 1 mg/mL dissolved in DMSO.

After separation by HPLC setup 3 and determination of retention times by simultaneous detection at 254 and 280 nm in three technical replicates, the calculation of log*P*_OW_^pH7.4^ (log*D*) was performed as described before.^37^ Three log*P* values of the reference substances were taken from literature,^37^ resulting in a mean log*P* value of the analyte (μHPLC log*P*_OW analyte_^pH7.4^). Furthermore, the values were compared to calculated log*P* (clog*P*) and topological polar surface area (tPSA) values from ChemDraw 20.1 (PerkinElmer, Inc., Waltham, USA) as well as clog*D*_pH7.4_ values from MarvinSketch 22.13 (ChemAxon Ltd., Budapest, Hungary). Where applicable, p*K*_a_ values were calculated with MarvinSketch 22.13.

### Cell-Free Binding Affinity Measurements

A commercially available homogeneous time-resolved fluorescence (HTRF) PD-1/PD-L1 Binding Assay Kit (Cisbio Bioassays SAS, Codolet, France, part no. 64PD1PEG) was used to determine *in vitro* binding affinities toward human PD-L1. The assay was prepared and performed according to the binding assay kit protocol using white, flat-bottom, low-volume Greiner 384 well plates (Merck KGaA, Darmstadt, Germany) and an HTRF-compatible Flexstation 3 Multi-Mode Microplate Reader (Molecular Devices LLC., San Jose, USA) for read-out. Experiments were repeated for a total of three times. 10-fold dilution series of the small molecules were prepared at a constant final DMSO concentration of 0.2%, as it is recommended to keep DMSO below 0.5%.^31^ 10-fold dilution series with constant DMSO concentration was used for the PD-1/PD-L1 Inhibitor 1 (Selleck Chemicals Llc, Houston, USA) and PD-1/PD-L1 Inhibitor 2 (Selleck Chemicals Llc, Houston, USA). 3-fold dilution series without DMSO was used for the antibody atezolizumab (MedChemExpress, Monmouth Junction, Sweden) and 10-fold dilution series without DMSO for the peptide PD-1/PD-L1 Inhibitor 3 (Selleck Chemicals Llc, Houston, USA). Assay validation was monitored using the provided PD-1/PD-L1 antibody from the assay kit. Half-maximal inhibitory concentration (IC_50_) calculation was performed with GraphPad Prism 8 (GraphPad Software, Inc., Boston, USA) using the variable slope (four parameters) dose–response fit. Data normalization was performed for interassay comparison of multiple experiments according to the procedure advised by Cisbio Bioassays and has been described before.^31^

### Cell Culture

PD-L1 expressing Chinese hamster ovary cells (CHO-*h*PD-L1) (BPS Bioscience, San Diego, USA) and CHO-K1 cells (ATCC, Manassas, USA) were routinely cultured in Ham’s F-12 medium supplemented with 10% fetal bovine serum, 100 units/mL penicillin, and 100 μg/mL streptomycin at 37 °C, 5% CO_2_ under subconfluent conditions. CHO-*h*PD-L1 F-12 medium was additionally supplemented with 1 mg/mL Geneticin (G418). All cell culture reagents were purchased from Gibco Thermo Fisher Scientific, Inc., Waltham, USA.

### Cell Viability/Cytotoxicity

The impact of cell viability and therefore toxicity of the newly developed compounds was measured with CHO-*h*PD-L1 cells using an MTT cell viability assay. 10,000 CHO-*h*PD-L1 cells were seeded in triplicates in sterile, flat 96-well plates (Corning, Corning, USA) and incubated (37 °C, 5% CO_2_) for 24 h in 100 μL total volume with different concentrations of test compounds with a final concentration of 0.5% DMSO. A concentration range spanning from 0.977 to 125 μM was applied for compounds **5a** and **5c**, whereas a concentration range ranging from 0.977 to 250 μM was employed for PD-1/PD-L1 Inhibitor 1 and Inhibitor 2. 10 μL of 5 mg/mL 3-(4,5-dimethylthiazol-2-yl)-2,5-diphenyltetrazolium bromide (MTT) (Merck KGaA, Darmstadt, Germany) in Dulbecco’s phosphate-buffered saline (DPBS) (Thermo Fisher Scientific, Inc., Waltham, USA) was added into each well and incubated for 4 h. 0.5% DMSO was used as vehicle control. Supernatant was removed and formed formazan crystals were dissolved in 100 μL of DMSO. The absorbance at 550 nm was measured using 690 nm as a reference wavelength. Half-maximal effective concentration (EC_50_) calculation was performed with GraphPad Prism 8 using the variable slope (four parameters) dose–response fit.

### Competitive Radioligand Binding Assay

Nonspecific binding of MultiScreen plates (Merck KGaA, Darmstadt, Germany) was blocked with additive-free Ham’s F-12 medium for 30 min, followed by the addition of 2 × 10^5^ CHO-K1 (ATCC, Manassas, USA) or CHO-*h*PD-L1 cells in 150 μL of additive-free Ham’s F-12 medium per well. Blocking agent (**5a**, **5c**, or atezolizumab) was added in desired concentrations (>100-fold excess) and incubated for 30 min (37 °C, 5% CO_2_). [^89^Zr]Zr-DFO-atezolizumab ([^89^Zr]Zr-atezolizumab) was synthesized as described before from *N*-succinyl-desferrioxamine-conjugated atezolizumab (atezolizumab-*N*-suc-DFO) (University Medical Center Groningen, Groningen, Netherlands) and [^89^Zr]Zr-oxalate (PerkinElmer, Inc., Waltham, USA),^2^ and added for a final concentration of 1.2 nM into each well. Supernatant was removed after 60 min of incubation using the MultiScreenHTS vacuum filtration system (Merck KGaA, Darmstadt, Germany) and cells were washed twice with DPBS. After drying, filters were punched and measured in a Wizard^2^ γ counter (PerkinElmer, Waltham, USA).

### Radiosyntheses with Carbon-11

Radiosyntheses were performed using a GE TRACERlab FX2 C module (General Electric Medical Systems, Uppsala, Sweden). Radionuclide production and production of [^11^C]methylating agents was performed as described before.^49^ In short, [^11^C]CO_2_ was produced in a GE PETtrace cyclotron (General Electric Medical Systems, Uppsala, Sweden) by irradiation of a gas target containing N_2_ and 0.5% O_2_ using the ^14^N(p,α)^11^C nuclear reaction with up to 16.5 MeV protons. [^11^C]CO_2_ was reduced to [^11^C]CH_4_ by H_2_ gas and nanopowdered nickel as a catalyst at 400 °C. [^11^C]CH_4_ was converted into [^11^C]CH_3_I with I_2_ at 720–740 °C by a radical reaction. Subsequently, [^11^C]CH_3_I was trapped in the solvent (i.e., DMSO) or precursor solution for small-scale reactions or automated synthesis, respectively.

For small-scale reaction of isomers **[^11^C]5a** & **[^11^C]5b**, as well as isomers ****[^11^C]**5c** & **[^11^C]5d**, a 100 μL solution of [^11^C]CH_3_I (∼0.5 GBq) was added to a 400 μL precursor solution of **4c** or **4e**, respectively, dissolved in DMSO in a 2 mL screw top vial equipped with a septum and a cap (Merck KGaA, Darmstadt, Germany) for final precursor concentrations of 0.5–2 mg/mL and stirred at room temperature, 60 or 100 °C for 5 min with or without addition of base (i.e., DIPEA) on a heating block equipped with a contact thermometer. Subsequently, the reaction was quenched with 200 μL of Milli-Q H_2_O and radiochemical conversion was determined by HPLC setup 5 and setup 6 for ****[^11^C]**5a** & ****[^11^C]**5b**, and ****[^11^C]**5c** & ****[^11^C]**5d**, respectively.

For automated radiosynthesis of ****[^11^C]**5c**, [^11^C]CH_3_I (∼51 GBq) was trapped in the reactor of the synthesis module containing 4 mg/mL precursor **4e** in 250 μL of DMSO. The reaction mixture was heated for 5 min at 100 °C. After cooling, the product was purified by semiprep. HPLC setup 4. The product-containing fraction was diluted with 90 mL of H_2_O ad inj. (B. Braun, Maria Enzersdorf, Austria) and pushed through a preconditioned Sep-Pak C18 Plus Light cartridge (Waters Corporation, Eschborn, Germany). The cartridge was washed with 5 mL of H_2_O ad inj. The product was eluted with 1.4 mL of ethanol (Merck KGaA, Darmstadt, Germany) and concentrated for *in vivo* application by means of a SpeedVac vacuum concentrator (Thermo Fisher Scientific, Inc., Waltham, USA) at 60 °C for 30 min. The residue was reconstituted in a 0.9% NaCl solution (B. Braun, Maria Enzersdorf, Austria) and subjected to quality control assessments. Radiochemical and chemical purity evaluations were conducted utilizing HPLC setup 6. The osmolality and pH of a product sample were measured using an osmometer (Sanova Medical Systems, Vienna, Austria) and a pH meter (Metrohm, Herisau, Swiss), respectively.

### *In Vitro* Stability Tests

Plasma stability was tested against pooled mouse plasma (Merck KGaA, Darmstadt, Germany) and pooled human plasma (Merck KGaA, Darmstadt, Germany). 25 μL of formulated radiotracer were incubated with 1250 μL of plasma at 37 °C. The amount of intact tracer (%) was determined after 0, 15, 30, and 60 min. 100 μL aliquots were quenched with the same amount of MeCN, centrifuged for 4 min at 4 °C with 21,380*g*, and analyzed by HPLC setup 1.

Plasma protein binding was assessed using 10 kDa centrifugal filters (Merck KGaA, Darmstadt, Germany). After centrifugation for 30 min at 21,380*g*, filtrates, and filters were measured separately in a γ counter. In addition, water was used instead of plasma to assess the nonspecific binding to filters.

Metabolic stability was tested using pooled, mixed gender, 20 donor human liver microsomes (HLM) (Corning, Corning, USA, Cat. #452161) according to the supplied protocol. In short, 15 μL of HLM (20 mg/mL), 15 μL of nicotinamide adenine dinucleotide phosphate (NADPH) regenerating system solution A (Corning, Corning, USA), 3 μL of solution B (Corning, Corning, USA), and 257 μL of a 1:10 dilution of 10× phosphate-buffered saline (PBS) concentrate (MORPHISTO, Offenbach am Main, Germany) were preincubated at 37 °C for 10 min.

Aliquots were drawn 0, 15, 30, and 60 min after the addition of 5 μL radiotracer, subsequently quenched with the same amount of MeCN, centrifuged for 4 min at 21,380*g*, and analyzed by HPLC setup 1 for the amount of intact tracer (%).

### Animals

8- to 10-week-old male NOD.Cg-*Prkdc*^*scid*^*Il2rg*^*tm1Wjl*^/SzJ (“NSG”) mice (Center for Biomedical Research and Translational Surgery, Vienna, Austria) were kept under conventional housing conditions, with food and water supply ad libitum and a 12 h day/night cycle. All animals were treated according to the European Union rules on animal care. The corresponding animal experiments were approved by the Austrian Ministry of Sciences (2021-0.422.476).

### Flow Cytometry

Surface protein expression of *h*PD-L1 on CHO-K1 and CHO-*h*PD-L1 cell lines was determined by flow cytometry. 10^6^ cells per sample were stained in 100 μL of FACS staining solution (PBS, 0.5% BSA, 0.2% NaN_3_) for 30 min at 4 °C. PE antihuman CD274 (PD-L1) antibody (BioLegend, San Diego, USA, Cat. #329705) was used as primary antibody at a dilution of 1:200. Data were acquired with a BD FACSCantoII (BD, Franklin Lakes, USA) and analyzed with FlowJo X (BD, Franklin Lakes, USA).

### Immunohistochemistry

Immunohistochemistry was performed using primary antibodies against PD-L1 (Cell Signaling Technology, Danvers, USA, Cat. #13684) and CD31 (Cell Signaling Technology, Danvers, USA, Cat. #77699). In an autostainer (Lab Vision AS 360, Thermo Fisher Scientific, Waltham, USA), a polymer detection system with a secondary antibody conjugated to an enzyme-labeled polymer was applied. For details regarding antibodies, dilution, pretreatment, and chromogen used, see Table S1.

### Tumor Grafting

The optimization of engraftment in NSG mice (*n* = 8) involved consideration of the quantity of injected cells and the timing of inoculation to attain an appropriate tumor size of approximately 250 mm^3^ and ensure uniform tumor growth rates among experimental groups. Optimal outcomes were obtained by administering 1.5 × 10^6^ cells in a PBS/matrigel (1:1) matrix (Corning, Corning, USA) over a 9- to 12-day inoculation period.

For imaging studies, NSG mice (*n* = 4) were injected subcutaneously with 1.5 × 10^6^ CHO-K1 cells into one flank and 1.5 × 10^6^ CHO-*h*PD-L1 cells in the opposite flank. Body weight and tumor development were monitored every second day by caliper measurement. The respective tumor volume was calculated according to the following equation: tumor volume (mm^3^) = *d*^2^ × *D/2* (where *d* is the shortest diameter and *D* the longest diameter). The animals were subjected to μPET imaging, when tumors reached a volume of at least 200 mm^3^. Tumor volume never exceeded 1 cm^3^. There were no losses.

### *In Vivo* μPET/CT Protocol and Image Analysis

Eleven days after inoculation, xenograft-bearing male NSG mice (*n* = 4) received lateral tail vein injection of 31.91 ± 3.05 MBq radiotracer under anesthesia using isoflurane (2.5%) mixed with oxygen (1.5 L/min) to avoid movement during the imaging. Application volumes did not exceed 100 μL per application. The mice were placed in the μPET/CT scanner (Inveon, Siemens Medical Solution, Knoxville, USA), covering the total body, and dynamic imaging was performed for up to 60 min to follow tracer distribution. Images were recorded with frames of 4 × 2 s, 5 × 3 s, 3 × 4 s, 5 × 5 s, 6 × 10 s, 1 × 20 s, 6 × 10 s, 2 × 100 s, 1 × 145 s, 1 × 200 s, 1 × 240 s, 1 × 270 s, 1 × 300 s, 1 × 340 s, 1 × 380 s, 1 × 400 s, 1 × 423 s, and 1 × 500 s. During the whole imaging procedure, vital parameters (respiration, body temperature) were continuously monitored using a dedicated monitoring unit (bioVet; m2m imaging, Cleveland, USA) to ensure the depth of anesthesia and well-being of the animals.

The acquired PET data was reconstructed reconstructed with Inveon Acquisition Workplace (Siemens Preclinical Solutions, Knoxville, TN, USA) using the OSEM3D/MAP algorithm and 18 MAP iterations on a 256 × 256 × 159 grid with a voxel size of 0.388 × 0.388 × 0.796 mm. Volumes of interest (VOIs) were created semiautomatically based on fused μPET and μCT images using PMOD software (Version 3.807; Bruker, Mannheim, Germany). The tracer uptake in the VOIs is normalized to injected dose and volume and expressed as percentage injected dose per cubic centimeter (% ID/cc).

### *Ex Vivo* Biodistribution

*Ex vivo* biodistribution was assessed 30, 40, and 70 min after tracer application in NSG mice. Radioactivity was determined using a Wizard^2^ γ counter. Samples were measured for 30 s, CPM-corrected for background counts, and half-life corrected to time of tracer injection. Organs were wet-weighted, and the percentage of injected dose per gram of organ was calculated (% ID/g).

### Statistical Analysis

Values are depicted as mean ± standard deviation (SD), and experiments were performed in triplicates and repeated at least three times. Peak areas in the radioactivity channel were corrected for decay during HPLC measurements and radiochemical conversion (RCC) was calculated according to Formula 1.
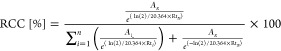
1where *A* is the peak area; Rt is the retention time (min); *x* is the substance of interest; and *i* denotes other entities.

Radioactive decay correction of RCC occurred during HPLC measurements. Spearman’s rank correlation was calculated in Microsoft Excel (Version 2307; Microsoft Corporation, Redmond, USA). Correlation categorization was adapted from Dancey and Reidy:^50^ weak: 0.1–0.39, moderate: 0.4–0.69, strong: 0.7–0.9. *t* tests were performed with GraphPad Prism 8. A confidence interval of 95% was applied.

## Data Availability

Data is contained within the article or Supporting Information.

## Supporting Information Available

Immunohistochemistry methodology details, PDB ligand information, calculated and measured physicochemical parameters and HTRF PD-L1 binding affinities, ligand docking experiments, MTT assay curves, radiosynthesis data for **[**^**11**^**C]5a** and **[**^**11**^**C]5c**, *in vivo* biodistribution and time–activity curves for **[**^**11**^**C]5c**; copies of ^1^H, ^13^C, ^19^F, and 2D NMR spectra, HR-MS spectra, as well as HPLC purity chromatograms for all compounds (PDF)
Molecular formula strings (SMILES) (CSV)

## Abbreviations Used

AcOH: acetic acid
BMS: Bristol Myers Squibb
CHO: Chinese hamster ovary
DCM: dichloromethane
DEAD: diethyl azodicarboxylate
DEE: diethyl ether
DIPEA: *N*,*N*-diisopropylethylamine
DMF: *N*,*N*-dimethylformamide
DMSO: dimethyl sulfoxide
EtOAc: ethyl acetate
HPLC: high-performance liquid chromatography
HTRF: homogeneous time-resolved fluorescence
MeCN: acetonitrile
MeOH: methanol
PAINS: pan assay interference compounds
PD-1: programmed cell death protein 1
PD-L1: programmed cell death 1 ligand 1
PET: positron emission tomography
RCC: radiochemical conversion
RT: room temperature
THF: tetrahydrofuran
