# Supplementary material for: Development and In Vivo Evaluation of Small-Molecule Ligands for Positron Emission Tomography of Immune Checkpoint Modulation Targeting Programmed Cell Death 1 Ligand 1
Source: J Med Chem. 2024 Mar 5;67(5):4036–62. doi: 10.1021/acs.jmedchem.3c02342 (PMC10945501; doi:10.1021/acs.jmedchem.3c02342)

Supporting information

Development and *In Vivo* Evaluation of Small Molecule Ligands for Positron Emission Tomography of Immune Checkpoint Modulation Targeting Programmed Cell Death 1 Ligand 1

Karsten Bamminger[1,2], Verena Pichler[1,3,*], Chrysoula Vraka[2], Tanja Limberger[1,4], Boryana Moneva[2], Katharina Pallitsch[5], Barbara Lieder[6,7], Anna Sophia Zacher[2], Stefanie Marie Ponti[2], Katarína Benčurová[2], Jiaye Yang[4], Sandra Högler[8], Petra Kodajova[8], Lukas Kenner[1,4,8], Marcus Hacker[2], Wolfgang Wadsak[1,2,*]

(1) CBmed GmbH - Center for Biomarker Research in Medicine, 8010 Graz, Austria
(2) Department of Biomedical Imaging and Image-guided Therapy, Division of Nuclear Medicine, Medical University of Vienna, 1090 Vienna, Austria
(3) Department of Pharmaceutical Sciences, Division of Pharmaceutical Chemistry, University of Vienna, 1090 Vienna, Austria
(4) Institute of Clinical Pathology, Medical University of Vienna, 1090 Vienna, Austria
(5) Institute of Organic Chemistry, University of Vienna, 1090 Vienna, Austria
(6) Institute of Physiological Chemistry, University of Vienna, 1090 Vienna, Austria
(7) Institute of Clinical Nutrition, University of Hohenheim, 70599 Stuttgart, Germany
(8) Unit of Laboratory Animal Pathology, University of Veterinary Medicine Vienna, 1210 Vienna, Austria

*Corresponding authors: wolfgang.wadsak@cbmed.at, verena.pichler@univie.ac.at

Table of contents

Immunohistochemistry S2
PDB ligands S2
Physicochemical parameters S3
Ligand docking experiments S5
MTT assay S6
Radiolabeling S7
In vivo biodistribution over time S10
Time-activity curves S11
Substance purity S12
Nuclear magnetic resonance spectroscopy S46
Mass spectrometry S134

Immunohistochemistry

Table S1: Immunohistochemistry procedure for PD-L1 and CD31 staining.

Antibody	Dilution	Antigen retrieval	Detection System	Immunostaining reagent	Counterstain
**PDL1 CST#13684**	1: 500	Heating in TRIS-EDTA buffer pH 9	Bright Vision Rabbit HRP (Medac, Wedel, Germany, Cat. #DPVR110 HRP)	DAB Quanto (Thermo Fisher Scientific Inc., Waltham, USA, Cat. #TA-125-QHDX)	Hematoxylin
**CD31 CST#77699**	1: 100	Heating in citrate buffer pH 6	Bright Vision Rabbit HRP (Medac, Wedel, Germany, Cat. #DPVR110 HRP)	Bright DAB (Medac, Wedel, Germany, Cat. #BS04-110)	Hematoxylin

PDB ligands

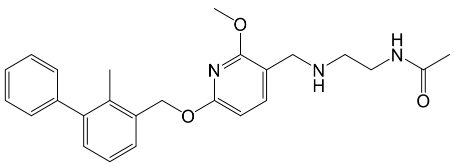

BMS-202

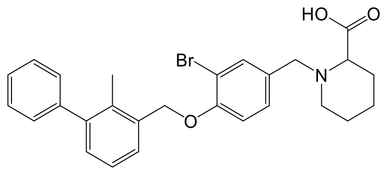

BMS-8

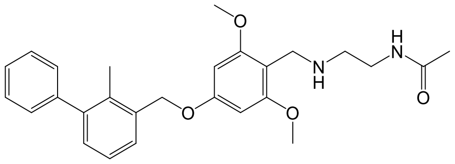

BMS-37

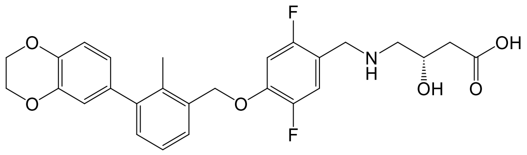

BMS-200

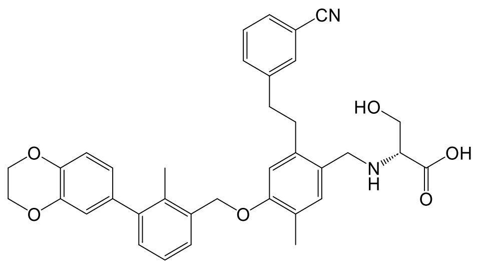

"BMS-1001" *

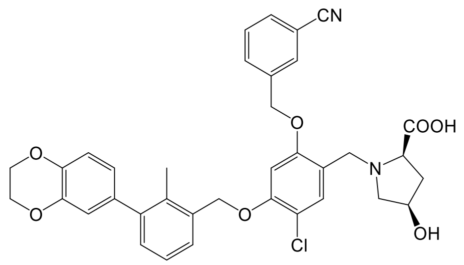

BMS-1166

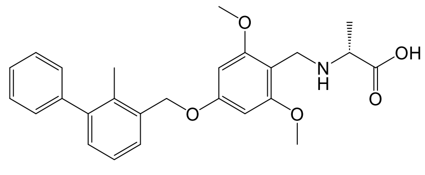

BMS-105

Figure S1: Representation of small molecule PD-L1 ligands from the PDB datasets. * The structural configuration of the ligand differs from the structure showcased in the original patent.

Physicochemical parameters

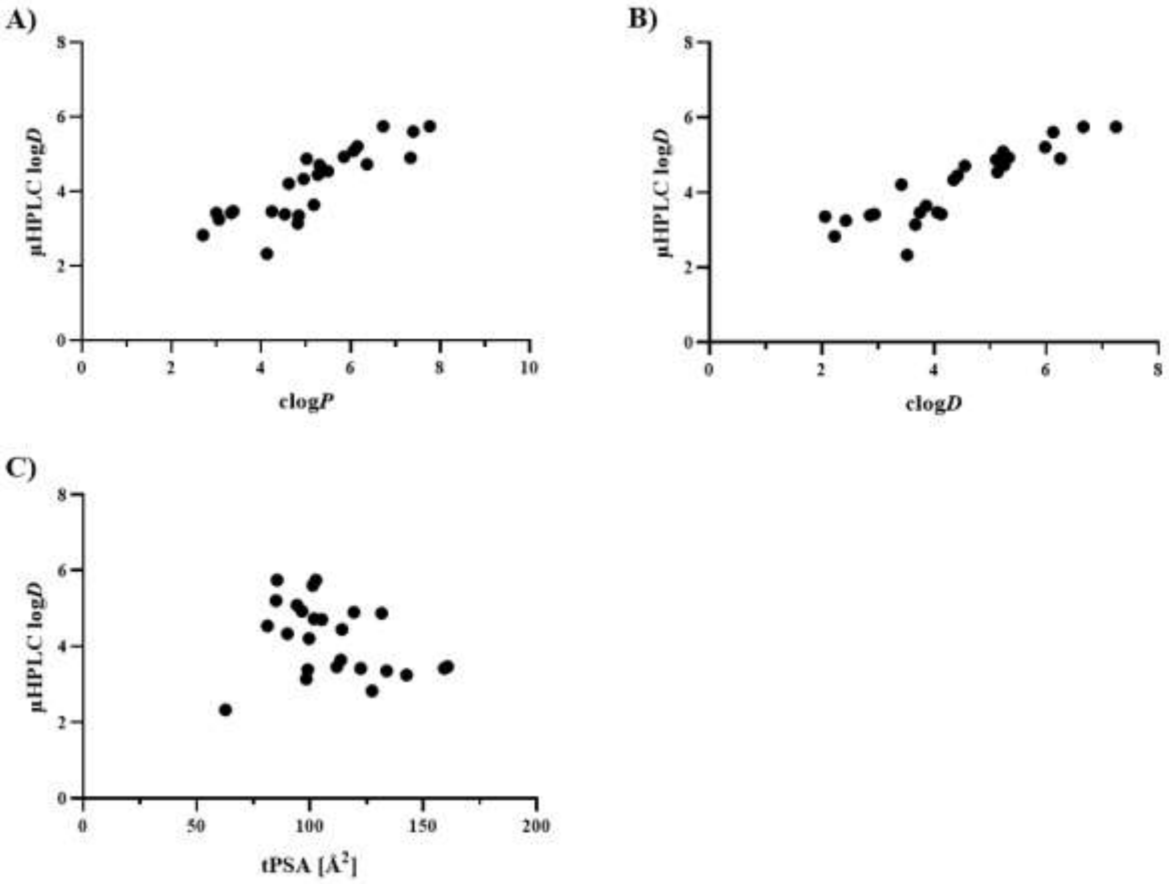

Figure S2: Correlation analysis of calculated parameters with measured µHPLC log*D*. Correlation of (**A**) clog*P*, (**B**) clog*D*, and (**C**) tPSA.

Table S2: Calculated and measured physicochemical parameters as well as HTRF PD-L1 binding affinities of intermediates **2**, **3**, and **4** and final products **5**. Antibody atezolizumab and small molecule compounds PD-1/PD-L1 Inhibitor 1 and Inhibitor 2, as well as macrocyclic peptide Inhibitor 3 were used as reference compounds. The parameters clog*P* and tPSA were calculated using ChemDraw, while clog$D_{pH\ 7.4}$ was calculated using MarvinSketch. ND = not determined.

Compound	clog*P*	clog$D_{pH\ 7.4}$	tPSA [Å2]	µHPLC log$D_{pH\ 7.4}$	IC_{50} [nM]
**2a**	5.25	5.39	38.66	ND	ND
**2b**	6.27	6.33	46.53	ND	>100,000
**2c**	6.19	5.84	64.99	ND	>100,000
**2d**	5.83	5.64	49.77	ND	ND
**3a**	7.31	7.44	59.32	ND	1,300 ± 305
**3b**	6.13	6.12	90.14	ND	207 ± 340
**3c**	7.23	6.95	77.78	ND	>5,000
**3d**	5.48	5.12	75.58	ND	>5,000
**3e**	5.77	5.92	74.92	ND	>5,000
**4a**	4.13	3.52	62.72	2.33 ± 0.14	>50,000
**4b**	4.24	3.75	111.81	3.46 ± 0.05	6.7 ± 0.4
**4c**	3.06	2.43	142.63	3.25 ± 0.03	6.1 ± 0.3
**4d**	5.27	4.42	114.20	4.45 ± 0.20	9.2 ± 0.6
**4e**	5.31	4.55	105.33	4.71 ± 0.24	7.7 ± 0.6
**4f**	5.18	3.86	113.61	3.64 ± 0.09	4.9 ± 0.3
**4g**	3.00	2.94	159.32	3.42 ± 0.05	46 ± 5
**4h**	3.33	4.13	122.40	3.42 ± 0.05	8.2 ± 1.8
**4i**	3.38	4.06	160.73	3.47 ± 0.06	3.7 ± 0.5
**4j**	6.37	5.25	101.84	4.73 ± 0.24	11 ± 1
**4k**	4.62	3.42	99.64	4.21 ± 0.16	50 ± 7
**4l**	4.53	2.86	99.05	3.39 ± 0.04	16 ± 3
**4m**	2.70	2.23	127.41	2.83 ± 0.04	6 ± 1
**4n**	4.95	4.35	90.11	4.34 ± 0.18	50 ± 3
**4o**	4.82	3.67	98.39	3.14 ± 0.01	29 ± 3
**5a**	4.84	2.06	133.84	3.36 ± 0.04	6.2 ± 0.6
**5b**	5.02	5.11	131.63	4.88 ± 0.26	30 ± 8
**5c**	5.85	5.33	96.54	4.93 ± 0.27	10.2 ± 0.2
**5d**	6.07	5.23	94.33	5.09 ± 0.29	19 ± 4
**5e**	7.40	6.12	101.33	5.61 ± 0.37	589 ± 178
**5f**	5.49	5.13	81.32	4.54 ± 0.21	16 ± 4
**5g**	6.73	6.66	85.54	>5.75	12 ± 2
**5h**	7.77	7.24	102.61	>5.75	1,290 ± 440
**5i**	6.15	5.98	85.10	5.21 ± 0.31	12 ± 1
**5j**	7.34	6.25	119.35	4.90 ± 0.27	1,700 ± 540
**Atezolizumab**	ND	ND	ND	ND	4.1 ± 0.4
**Inhibitor 1 (BMS-1)**	4.60	2.73	68.23	3.16 ± 0.16	202 ± 27
**Inhibitor 2 (BMS-202)**	4.41	3.64	71.95	3.88 ± 0.12	101 ± 10
**Inhibitor 3**	ND	ND	ND	ND	113 ± 45

Ligand docking experiments

Table S3*:* Calculated ligand docking parameters as determined by three independent runs. Lower Binding Affinity Score and Affinity values indicate better affinity. Lit. IC_{50} = literature IC_{50} as stated in the original patent.

Compound	Binding Affinity Score	Affinity [kcal/mol]	Lit. IC_{50} [nM]	Measured IC_{50} [nM]
**BMS-8**	-39.37 ± 9.14	-10.30 ± 0.79	146	--
**BMS-16**	-36.16 ± 4.04	-9.87 ± 0.15	1,945	--
**BMS-39**	-34.27 ± 3.26	-10.63 ± 0.58	4,184	--
**BMS-49**	-36.43 ± 3.95	-10.73 ± 0.15	9,492	--
**BMS-75**	-37.31 ± 3.36	-10.77 ± 0.21	953	--
**BMS-82**	-38.94 ± 3.26	-10.47 ± 0.32	3,186	--
**BMS-101**	-31.91 ± 4.33	-11.03 ± 0.06	1,076	--
**BMS-107**	-34.36 ± 2.14	-10.00 ± 0.20	329	--
**BMS-114**	-33.15 ± 1.26	-11.43 ± 0.25	43	--
**BMS-163**	-39.35 ± 1.03	-8.20 ± 0.35	93	--
**BMS-172**	-34.79 ± 1.16	-9.40 ± 0.36	107	--
**BMS-174**	-32.62 ± 2.09	-10.13 ± 0.15	22	--
**BMS-200**	-37.65 ± 2.39	-11.20 ± 0.17	80	--
**BMS-202**	-29.35 ± 3.11	-9.57 ± 0.12	18	101 ± 10
**BMS-1001**	-38.70 ± 1.13	-10.17 ± 0.25	2.25	--
**BMS-1016**	-35.24 ± 3.73	-10.73 ± 0.40	4.55	--
**BMS-1043**	-38.06 ± 4.23	-10.57 ± 0.06	239.2	--
**BMS-1057**	-37.23 ± 2.20	-9.93 ± 0.06	985.8	--
**BMS-1082**	-47.04 ± 9.38	-10.43 ± 0.38	828.4	--
**BMS-1095**	-37.43 ± 8.00	-10.57 ± 0.06	81.25	--
**BMS-1108**	-42.08 ± 3.55	-10.40 ± 0.26	624.2	--
**BMS-1119**	-41.00 ± 2.72	-10.30 ± 0.17	14,250	--
**BMS-1166**	-32.98 ± 3.57	-11.20 ± 1.91	1.4	--
**BMS-1197**	-39.64 ± 5.77	-12.27 ± 0.12	1.85	--
**BMS-1205**	-40.23 ± 4.92	-10.77 ± 0.23	2.71	--
**BMS-1210**	-39.19 ± 0.48	-11.10 ± 0.20	12.74	--
**BMS-1218**	-40.05 ± 3.35	-10.77 ± 0.25	10.11	--
**BMS-1220**	-34.45 ± 0.66	-11.13 ± 0.06	6.07	--
**BMS-1239**	-33.67 ± 4.04	-10.93 ± 0.06	148.9	--
**BMS-1250**	-42.12 ± 4.89	-10.87 ± 0.25	1.19	--
**BMS-1288**	-35.19 ± 1.19	-11.70 ± 0.10	1.88	--
**BMS-1306**	-33.99 ± 0.92	-9.80 ± 1.30	0.92	--
**BMS-2002**	-34.08 ± 1.31	-10.57 ± 0.31	10	--
**BMS-2010**	-40.73 ± 5.32	-10.70 ± 0.17	50	--
**BMS-3013**	-34.64 ± 1.84	-12.07 ± 0.06	80	--
**BMS-3024**	-34.16 ± 1.95	-11.00 ± 0.20	5.54	--
**BMS-3029**	-35.24 ± 6.64	-11.43 ± 0.12	2,350	--
**4a**	-32.60 ± 1.38	-9.97 ± 0.15	--	>50,000
**4b**	-36.41 ± 5.24	-10.50 ± 0.17	--	6.7 ± 0.4
**4c**	-35.23 ± 0.89	-11.03 ± 0.15	--	6.1 ± 0.3
**4d**	-36.92 ± 3.44	-10.43 ± 0.15	--	9.2 ± 0.6
**4e**	-32.67 ± 1.98	-10.70 ± 0.89	--	7.7 ± 0.6
**4f**	-36.39 ± 2.36	-11.67 ± 0.12	--	4.9 ± 0.3
**4g**	-37.24 ± 11.72	-8.57 ± 1.33	--	46 ± 5
**4h**	-39.42 ± 1.81	-10.30 ± 0.00	--	8.2 ± 1.8
**4i**	-35.21 ± 4.78	-9.10 ± 0.36	--	3.7 ± 0.5
**4j**	-41.88 ± 7.53	-10.63 ± 0.25	--	11 ± 1
**4k**	-30.15 ± 3.33	-9.77 ± 1.12	--	50 ± 7
**4l**	-37.39 ± 1.94	-11.37 ± 0.12	--	16 ± 3
**4m**	-29.59 ± 5.63	-9.40 ± 1.04	--	6 ± 1
**4n**	-37.70 ± 2.38	-9.10 ± 0.95	--	50 ± 3
**4o**	-38.51 ± 0.85	-10.17 ± 0.06	--	29 ± 3
**5a**	-30.63 ± 0.37	-11.23 ± 0.31	--	6.2 ± 0.6
**5b**	-31.89 ± 11.50	-10.40 ± 0.87	--	30 ± 8
**5c**	-36.29 ± 5.86	-11.27 ± 0.15	--	10.2 ± 0.2
**5d**	-36.20 ± 4.18	-10.70 ± 0.26	--	19 ± 4
**5e**	-34.55 ± 1.94	-11.03 ± 0.40	--	589 ± 178
**5f**	-41.18 ± 4.47	-9.40 ± 0.72	--	16 ± 4
**5g**	-30.67 ± 5.13	-10.20 ± 1.92	--	12 ± 2
**5h**	-34.13 ± 4.77	-10.97 ± 0.21	--	1,290 ± 440
**5i**	-36.59 ± 2.88	-11.10 ± 0.10	--	12 ± 1
**5j**	-35.28 ± 9.65	-9.10 ± 0.44	--	1,700 ± 540

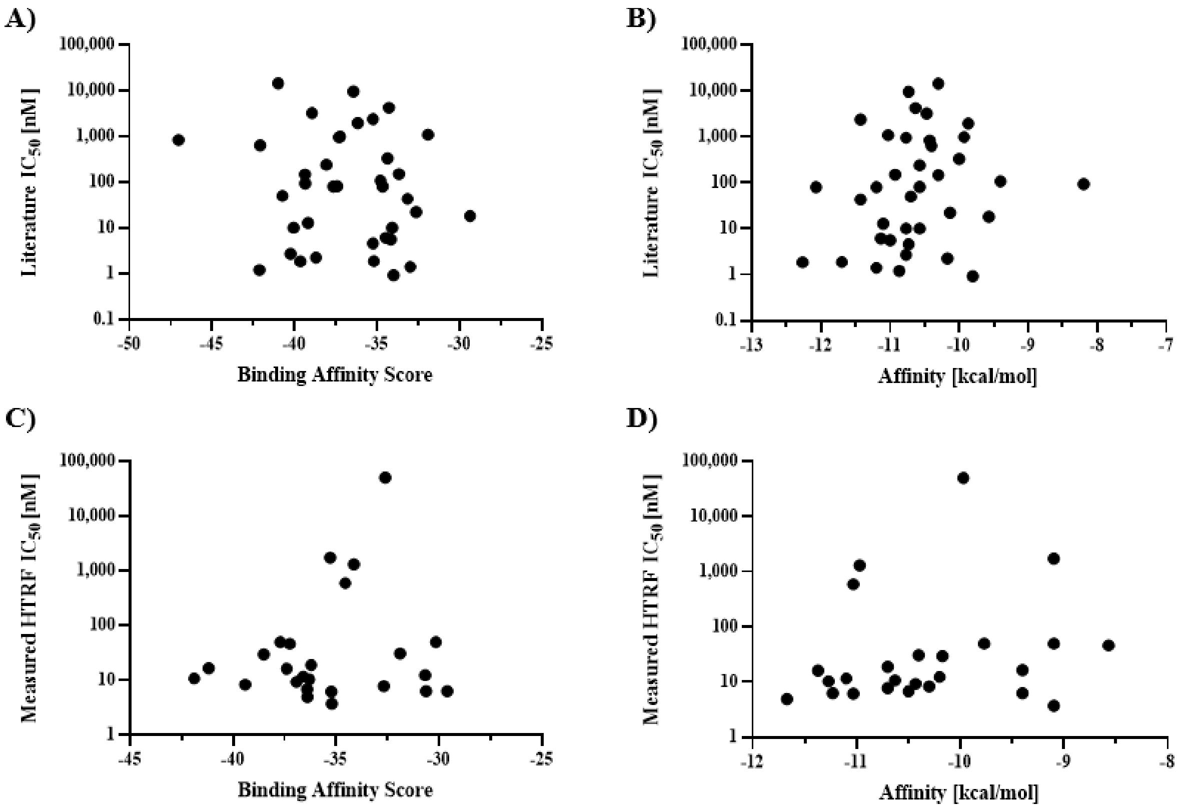

Figure S3: Correlation analysis of ligand docking parameters with reported and measured PD-L1 binding affinities. Correlation of (**A**) Binding Affinity Score and (**B**) Affinity with literature IC_{50}, as well as (**C**) Binding Affinity Score and (**D**) Affinity with measured HTRF IC_{50}.

MTT assay

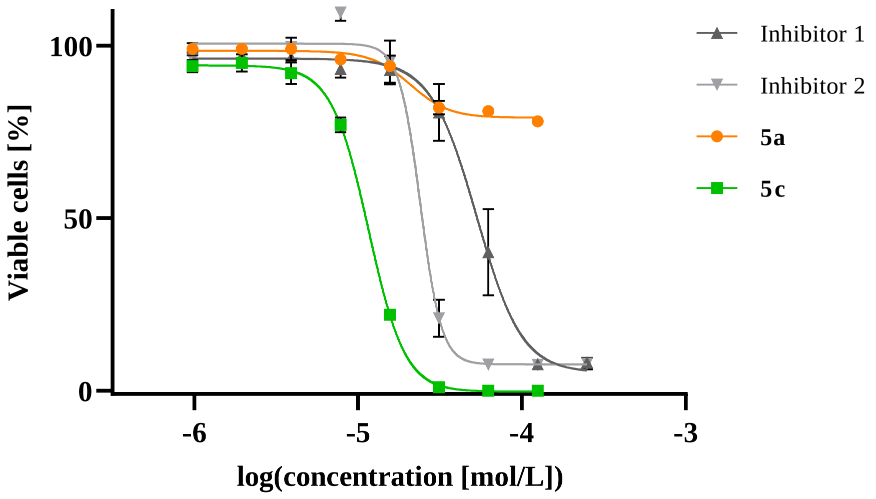

Figure S4: Representative MTT assay dose-response curves of small molecules evaluated on CHO-*h*PD-L1 cells.

Radiolabeling

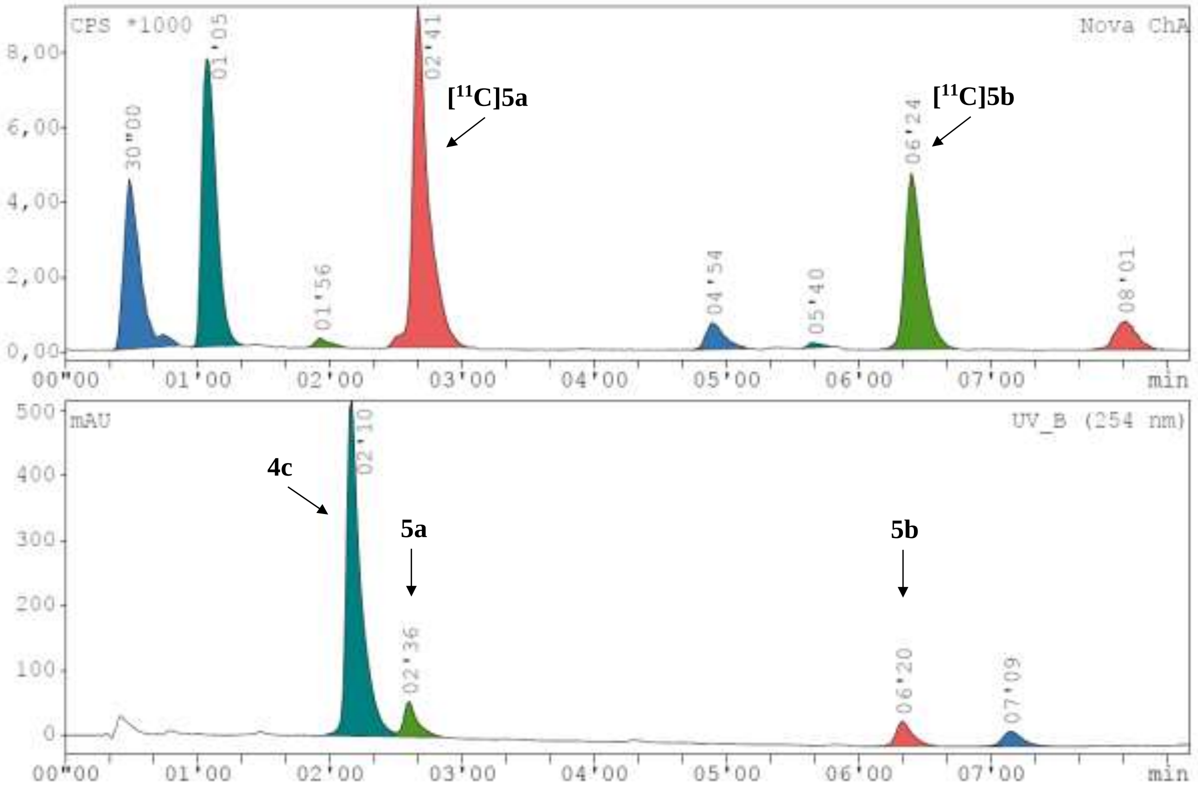

Figure S5: Representative radio-HPLC chromatogram (radioactivity channel top, UV channel bottom) for the synthesis of **[^{11}C]5a** with a RCC of 32.8% (corrected for decay during HPLC measurement). The identity of the product peaks **5a** and **5b** was validated through co-injection of reference standards.

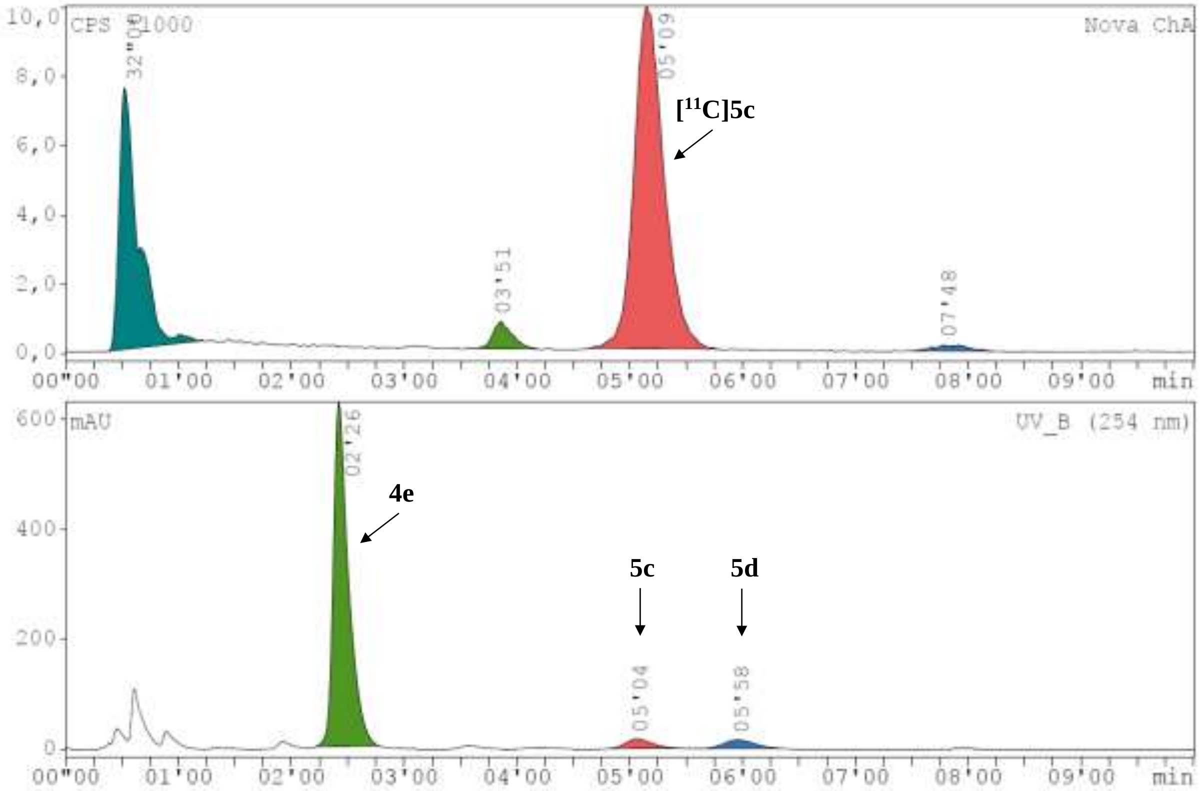

Figure S6: Representative radio-HPLC chromatogram (radioactivity channel top, UV channel bottom) for the synthesis of **[11C]5c** with a RCC of 67.0% (corrected for decay during HPLC measurement). The identity of the product peaks **5c** and **5d** was validated through co-injection of reference standards.

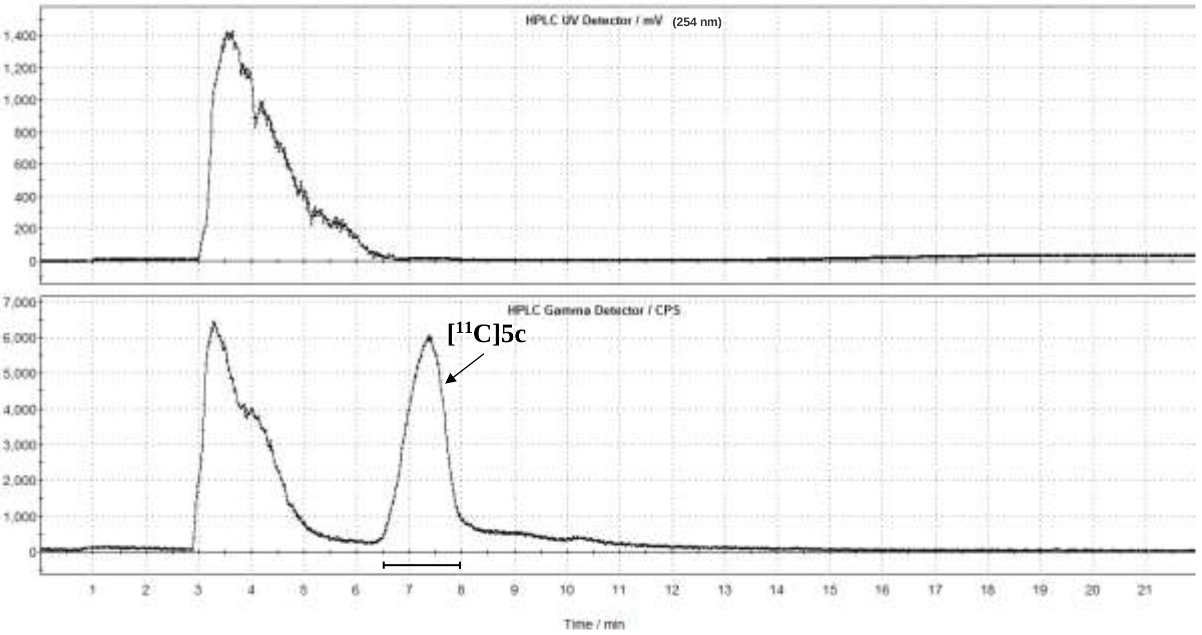

Figure S7: Representative semi-preparative HPLC chromatogram (UV channel top, radioactivity channel bottom) of **[11C]5c** radiosynthesis.

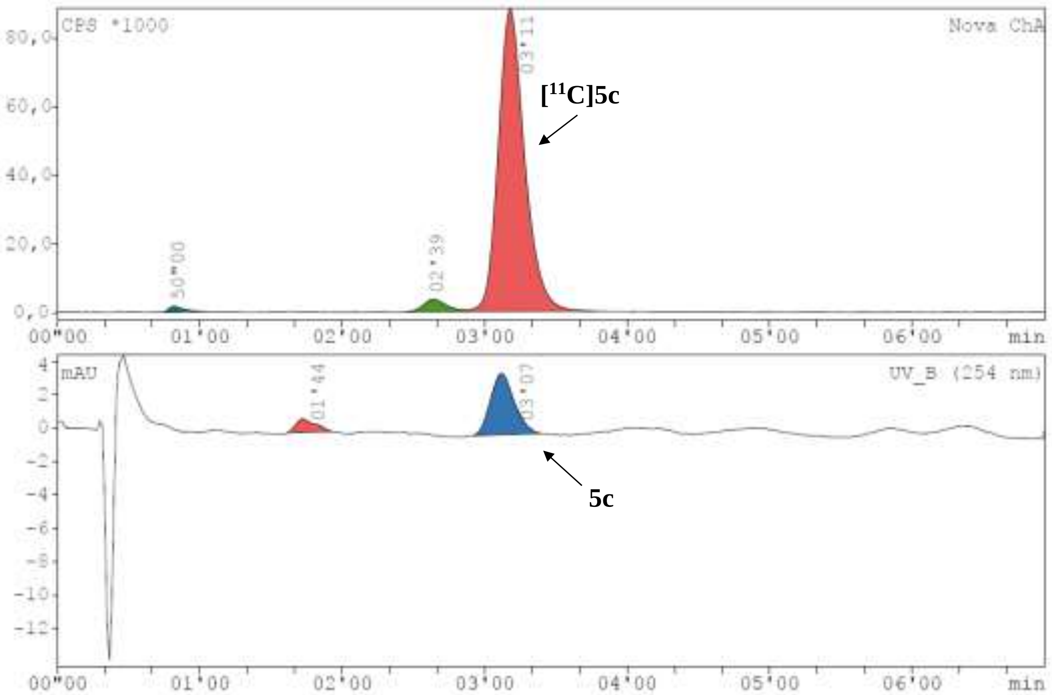

Figure S8: Representative radio-HPLC chromatogram (radioactivity channel top, UV channel bottom) of **[11C]5c** radiosynthesis with 95.5% radiochemical purity. The identity of the product peak was validated through a prior injection of a reference standard.

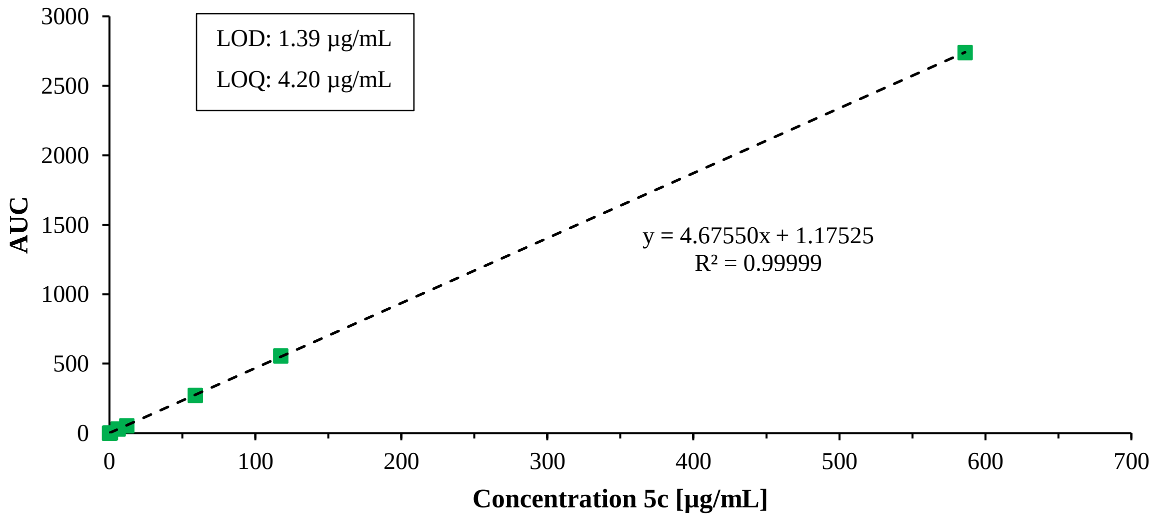

Figure S9: Standard curve of **5c** for determination of molar activity after radiosynthesis. AUC = area under the curve.

In vivo biodistribution over time

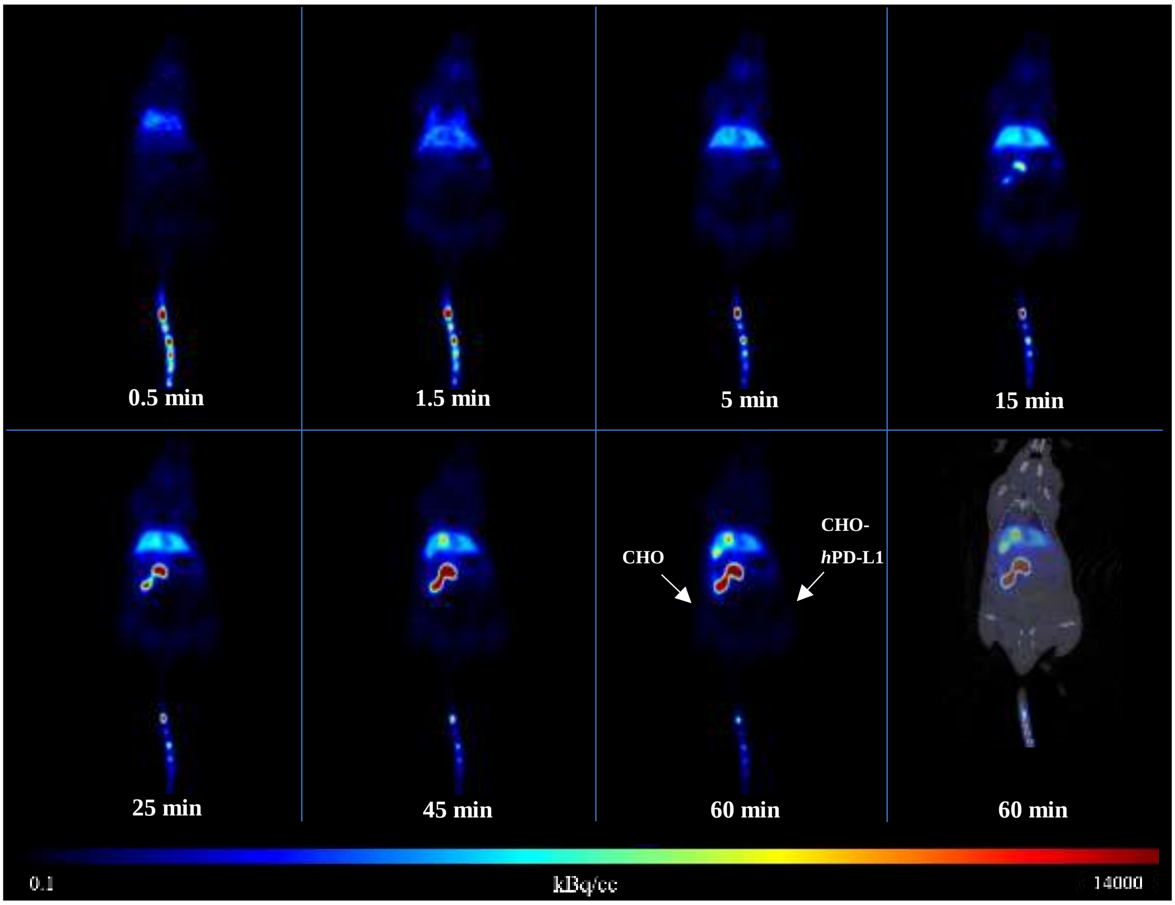

Figure S10: PET images capturing the *in vivo* distribution of **[^{11}C]5c** are presented at specified time points during a dynamic 60-minute µPET/CT scan. Images were obtained following the i.v. injection of 31 MBq of **[^{11}C]5c**.

Time-activity curves

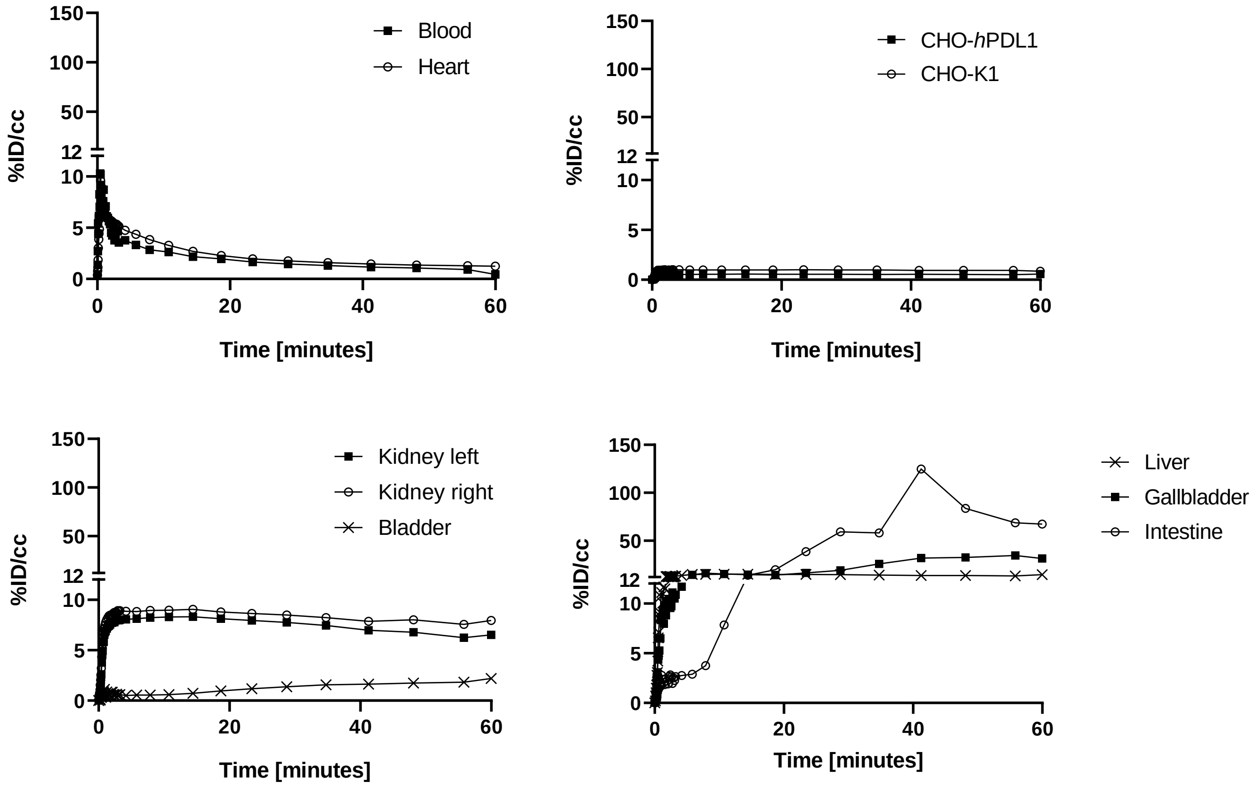

Figure S11: Time-activity curves of **[^{11}C]5c** for different regions of interest.

Substance purity

Figure S12: 5-Chloro-6-((2-methyl-[1,1'-biphenyl]-3-yl)methoxy)nicotinaldehyde (**2a**):

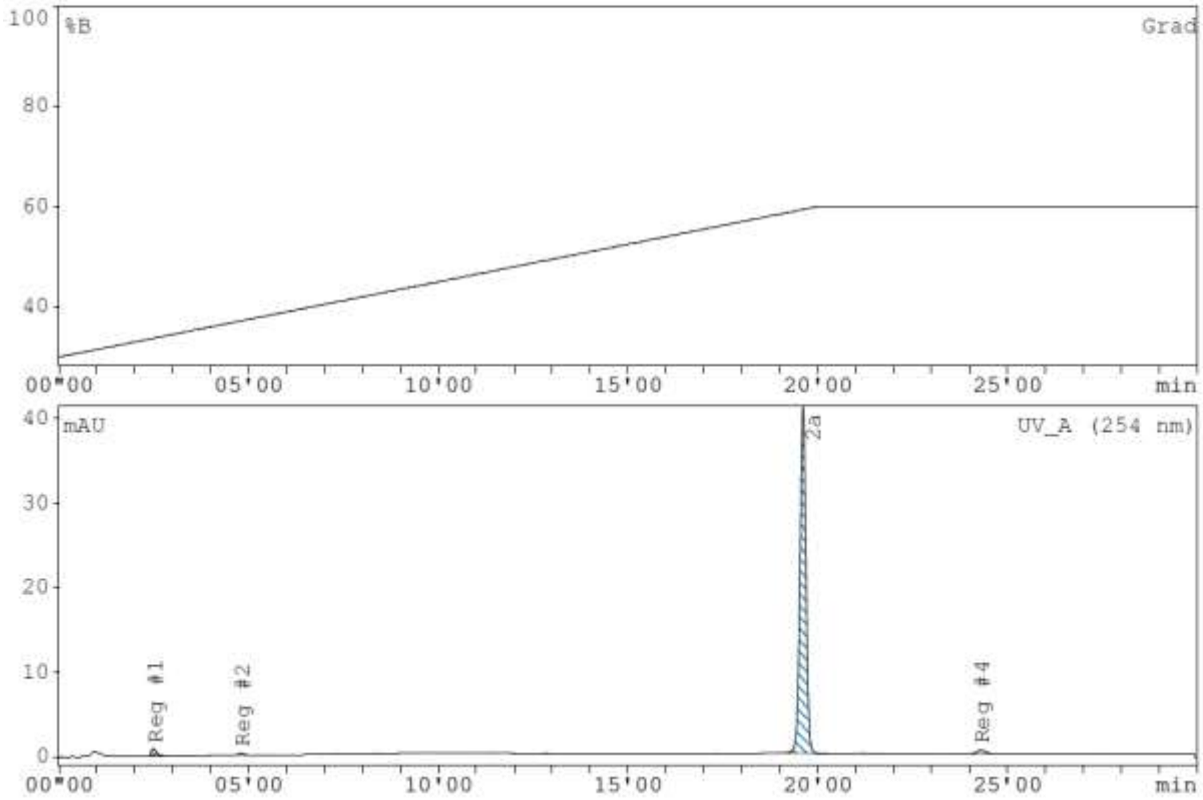

Integration UV_A (254 nm)

Substance	R/T s	Type	Area mAU*s	%Area %
Reg #1	02'29	BB	9,1679	1,99
Reg #2	04'48	BB	1,4947	0,32
2a	19'37	BB	444,9343	96,45
Reg #4	24'18	BB	5,7181	1,24
Sum in ROI			461,3151	100,00

Figure S13: 5-Chloro-2-hydroxy-4-((2-methyl-[1,1'-biphenyl]-3-yl)methoxy)benzaldehyde (**2b**):

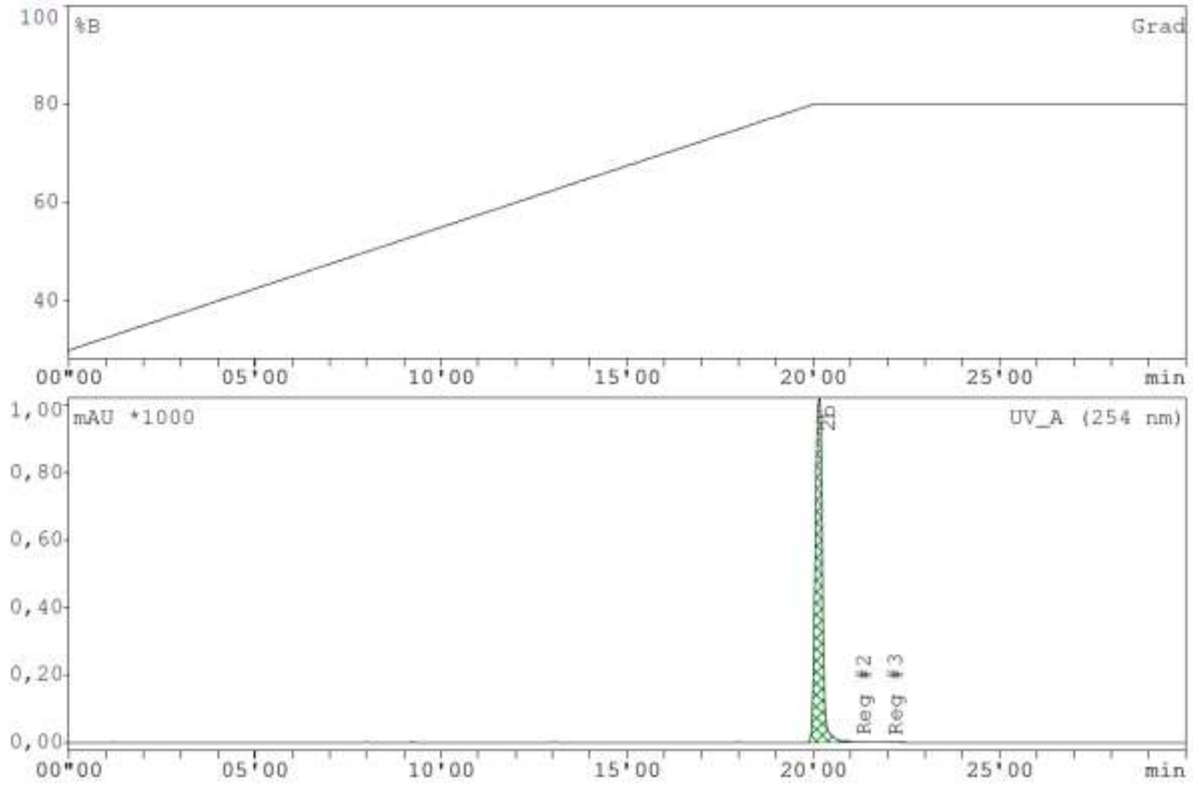

Integration UV_A (254 nm)

Substance	R/T s	Type	Area mAU*s	%Area %
2b	20'09	BD	13544,83	99,55
Reg #2	21'19	DB	49,46	0,36
Reg #3	22'09	BB	11,69	0,09
Sum in ROI			13605,98	100,00

Figure S14: 5-Chloro-4-((3-(2,3-dihydrobenzo[*b*][1,4]dioxin-6-yl)-2-methylbenzyl)oxy)-2-hydroxybenzaldehyde (**2c**):

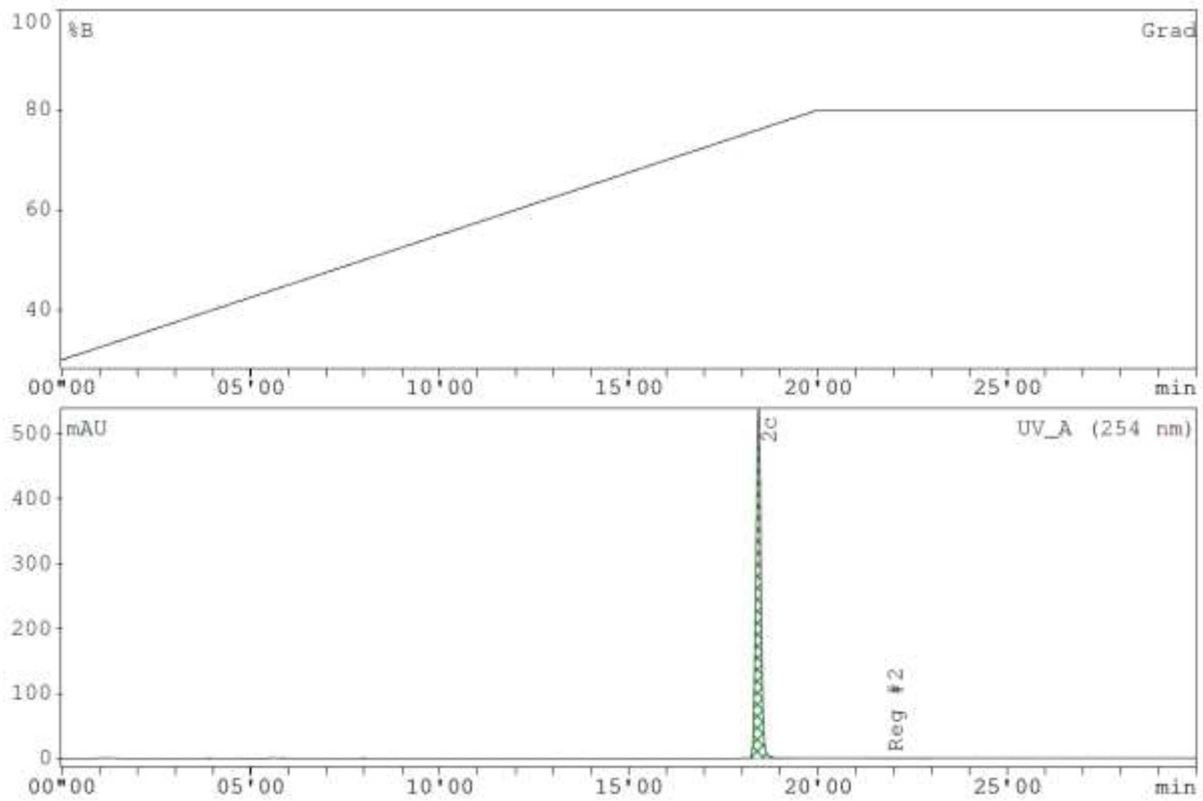

Integration UV_A (254 nm)

Substance	R/T s	Type	Area mAU*s	%Area %
2c	18'26	BB	4873,643	99,00
Reg #2	22'00	BB	32,377	0,66
Reg #3	30'47	BB	16,831	0,34
Sum in ROI			4922,851	100,00

Figure S15: 5-Chloro-2-hydroxy-4-((2-methyl-3-(1*H*-pyrrol-1-yl)benzyl)oxy)benzaldehyde (**2d**):

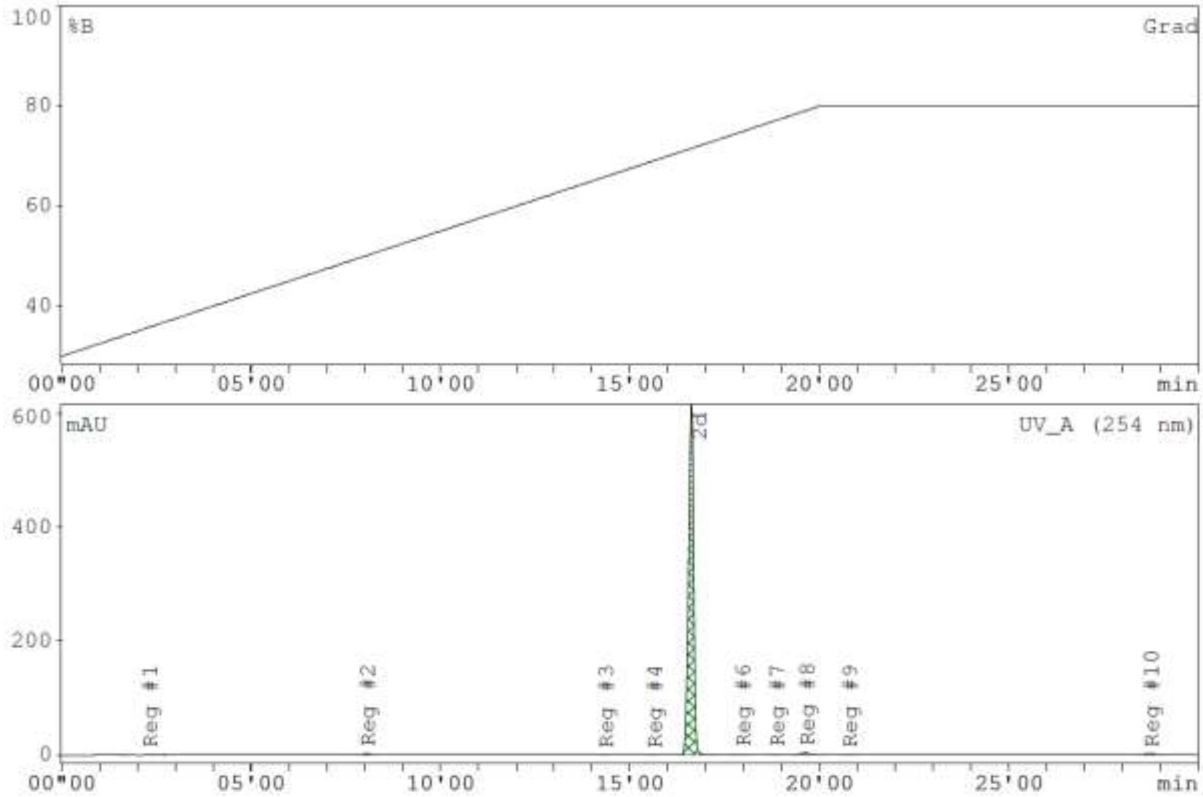

Integration UV_A (254 nm)

Substance	R/T s	Type	Area mAU*s	%Area %
Reg #1	02'18	BB	8,123	0,14
Reg #2	08'02	BB	24,264	0,42
Reg #3	14'19	BB	4,093	0,07
Reg #4	15'36	BB	4,096	0,07
2d	16'37	BB	5649,665	96,95
Reg #6	17'57	BB	14,779	0,25
Reg #7	18'51	BB	7,752	0,13
Reg #8	19'38	BB	60,277	1,03
Reg #9	20'46	BB	8,131	0,14
Reg #10	28'43	BB	46,036	0,79
Sum in ROI			5827,216	100,00

Figure S16: 3-((4-Chloro-2-formyl-5-((2-methyl-[1,1'-biphenyl]-3-yl)methoxy)phenoxy)methyl)benzonitrile (**3a**):

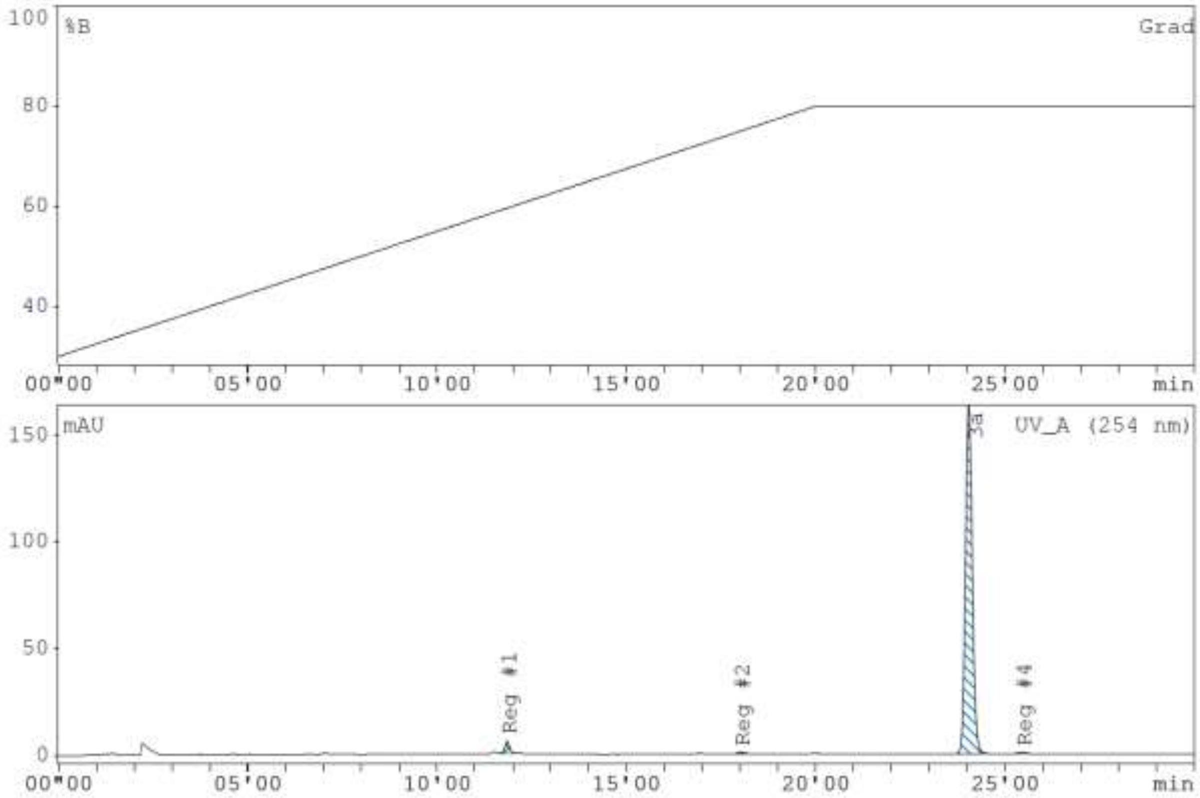

Integration UV_A (254 nm)

Substance	R/T s	Type	Area mAU*s	%Area %
Reg #1	11'51	BB	48,260	2,29
Reg #2	18'01	BB	9,165	0,43
3a	24'03	BB	2033,331	96,49
Reg #4	25'28	BB	6,656	0,32
Reg #5	33'07	BB	9,860	0,47
Sum in ROI			2107,272	100,00

Figure S17: 4-((4-Chloro-5-((3-(2,3-dihydrobenzo[*b*][1,4]dioxin-6-yl)-2-methylbenzyl)oxy)-2-formylphenoxy)methyl)picolinonitrile (**3b**):

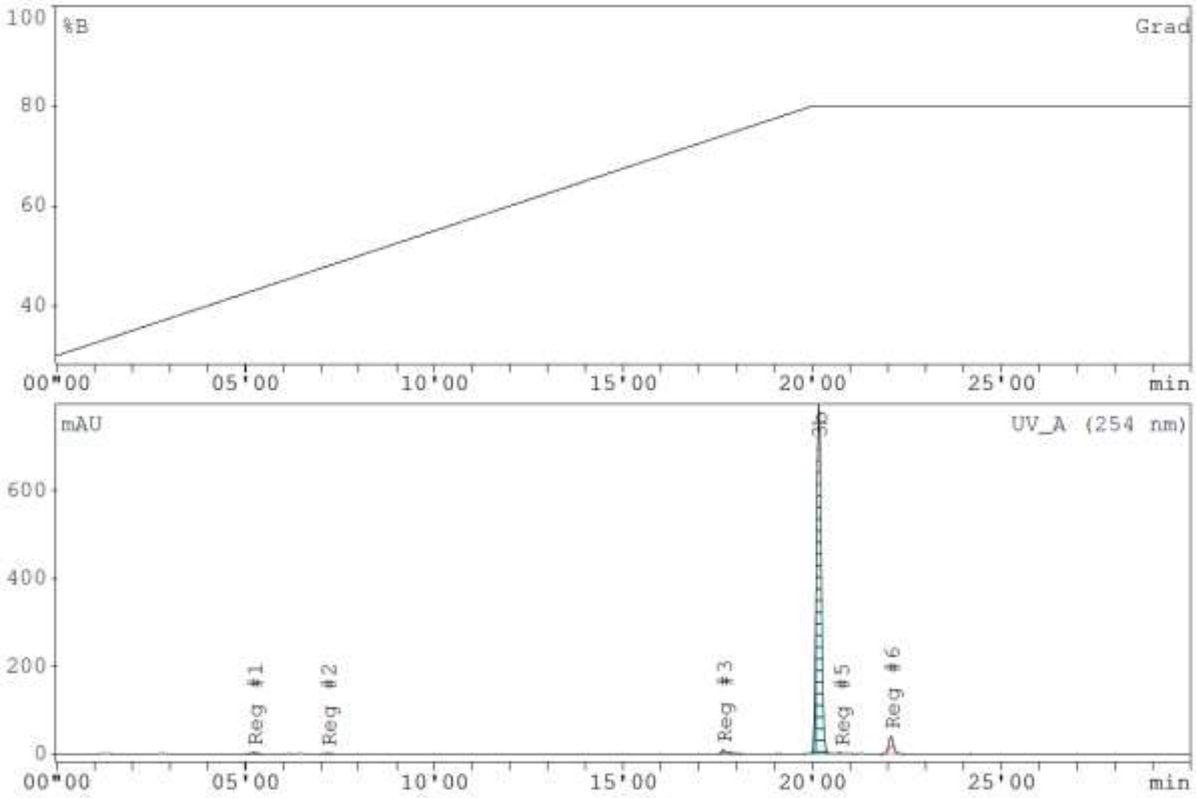

Integration UV_A (254 nm)

Substance	R/T s	Type	Area mAU*s	%Area %
Reg #1	05'13	BB	31,440	0,42
Reg #2	07'10	BB	16,566	0,22
Reg #3	17'39	BB	95,650	1,29
3b	20'10	BD	6883,311	92,80
Reg #5	20'44	DB	21,779	0,29
Reg #6	22'05	BB	368,246	4,96
Sum in ROI			7416,992	100,00

Figure S18: 3-((4-Chloro-5-((3-(2,3-dihydrobenzo[*b*][1,4]dioxin-6-yl)-2-methylbenzyl)oxy)-2-formylphenoxy)methyl)benzonitrile (**3c**):

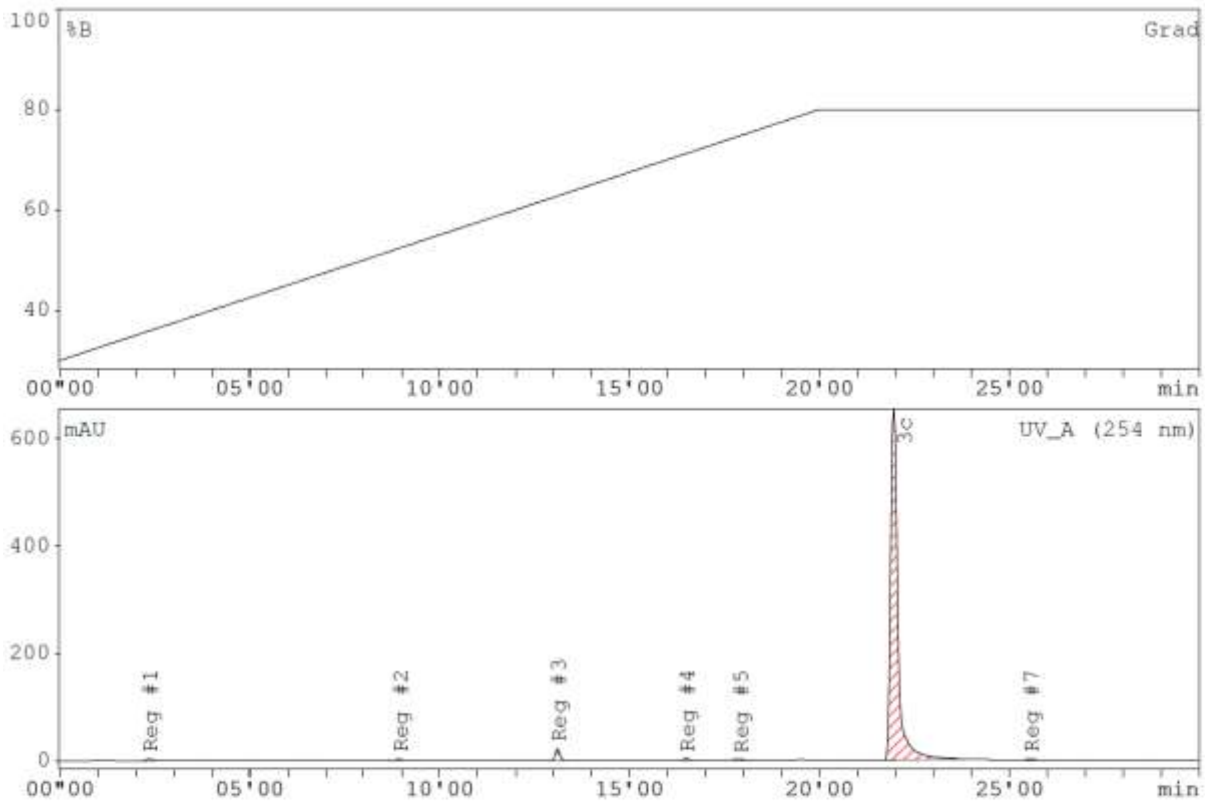

Integration UV_A (254 nm)

Substance	R/T s	Type	Area mAU*s	%Area %
Reg #1	02'21	BB	26,775	0,30
Reg #2	08'56	BB	25,017	0,28
Reg #3	13'06	BB	174,567	1,93
Reg #4	16'29	BB	31,920	0,35
Reg #5	17'53	BB	33,228	0,37
3c	21'57	BB	8706,522	96,27
Reg #7	25'33	BB	30,937	0,34
Reg #8	31'06	BB	14,448	0,16
Sum in ROI			9043,414	100,00

Figure S19: 5-Chloro-4-((3-(2,3-dihydrobenzo[*b*][1,4]dioxin-6-yl)-2-methylbenzyl)oxy)-2-(oxazol-4-ylmethoxy)benzaldehyde (**3d**):

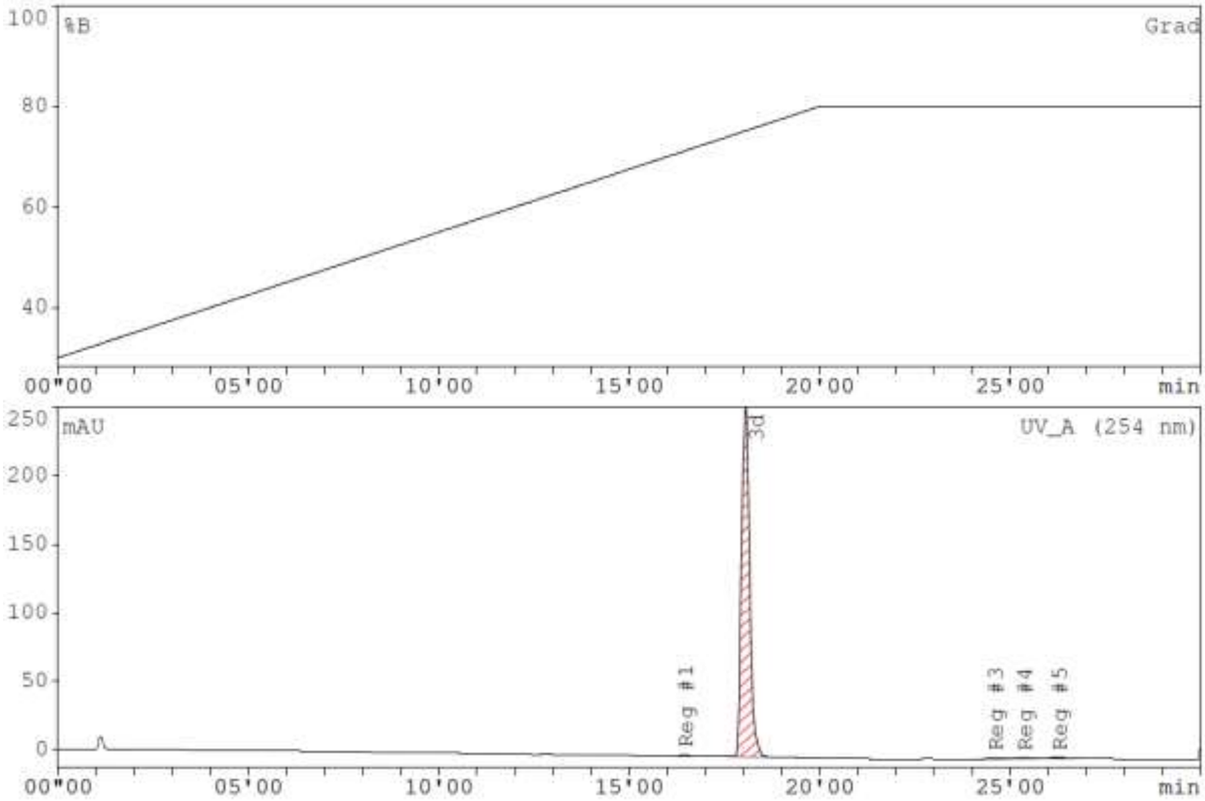

Integration UV_A (254 nm)

Substance	R/T s	Type	Area mAU*s	%Area %
Reg #1	16'27	BB	14,092	0,36
3d	18'04	BB	3807,188	97,90
Reg #3	24'36	BD	14,819	0,38
Reg #4	25'21	DD	25,418	0,65
Reg #5	26'15	DB	27,503	0,71
Sum in ROI			3889,019	100,00

Figure S20: 4-((4-Chloro-2-formyl-5-((2-methyl-3-(1*H*-pyrrol-1-yl)benzyl)oxy)phenoxy)methyl)picolinonitrile (**3e**):

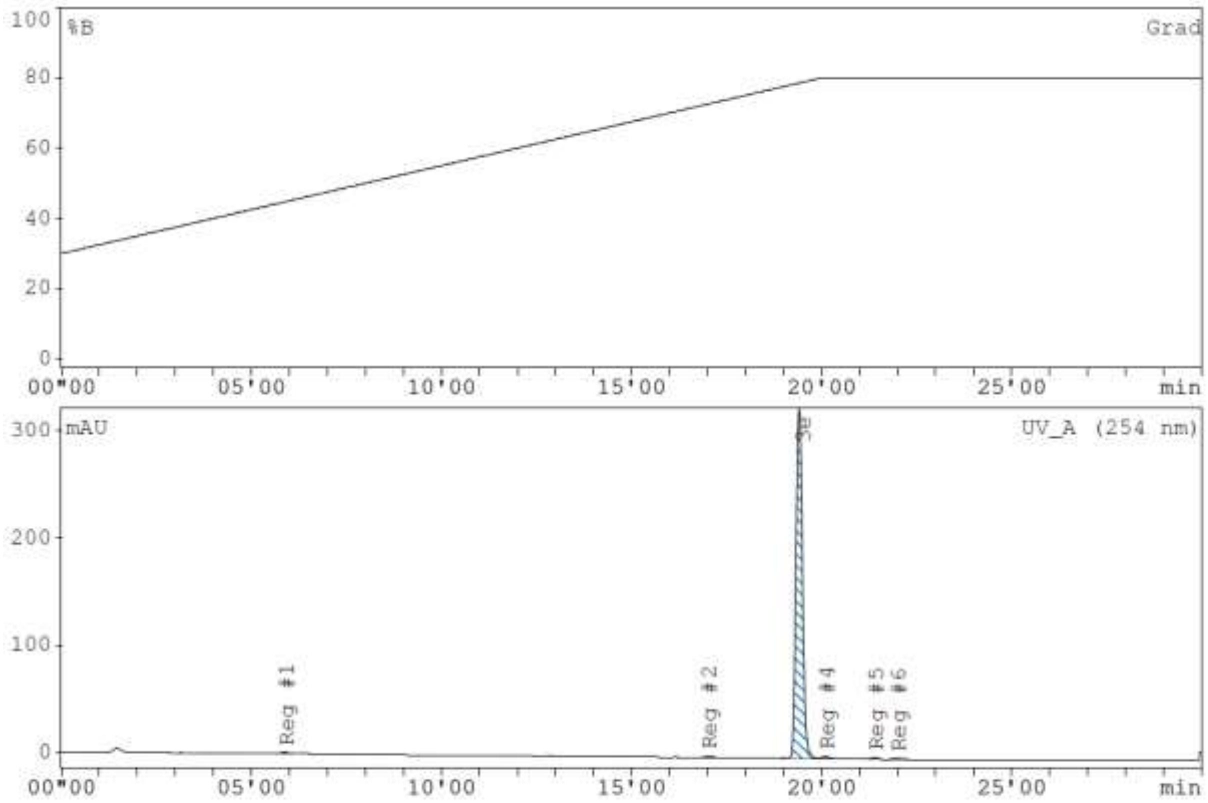

Integration UV_A (254 nm)

Substance	R/T s	Type	Area mAU*s	%Area %
Reg #1	05'53	BB	11,721	0,29
Reg #2	17'01	BB	16,413	0,41
3e	19'25	BD	3921,691	97,79
Reg #4	20'06	DB	24,067	0,60
Reg #5	21'24	BB	19,769	0,49
Reg #6	21'59	BB	16,573	0,41
Sum in ROI			4010,233	100,00

Figure S21: *N*-(2-(((5-chloro-6-((2-methyl-[1,1'-biphenyl]-3-yl)methoxy)pyridin-3-yl)methyl)amino)ethyl)acetamide (**4a**):

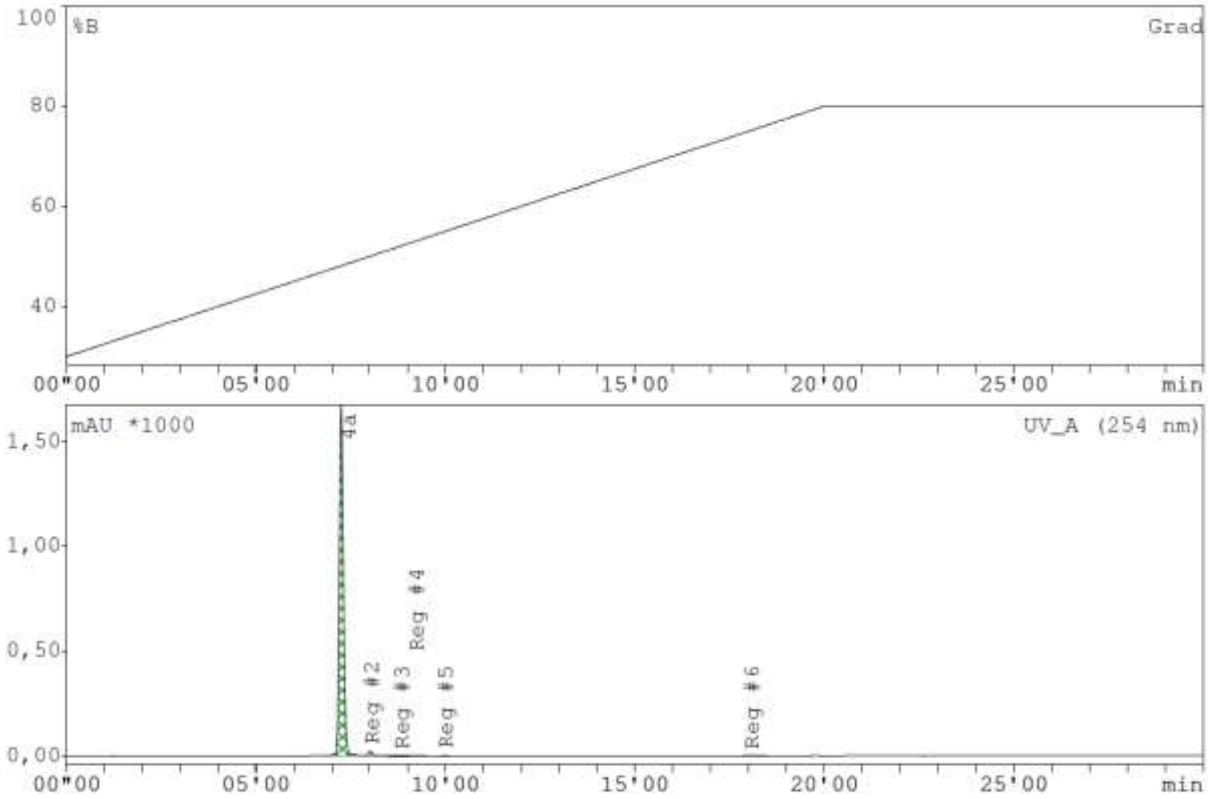

Integration UV_A (254 nm)

Substance	R/T s	Type	Area mAU*s	%Area %
4a	07'15	BD	12560,04	98,05
Reg #2	08'01	DD	185,05	1,44
Reg #3	08'48	DD	25,62	0,20
Reg #4	09'12	DB	14,89	0,12
Reg #5	09'58	BB	2,46	0,02
Reg #6	18'03	BB	21,62	0,17
Sum in ROI			12809,68	100,00

Figure S22: (5-Chloro-2-((3-cyanobenzyl)oxy)-4-((2-methyl-[1,1'-biphenyl]-3-yl)methoxy)benzyl)-*D*-serine (**4b**):

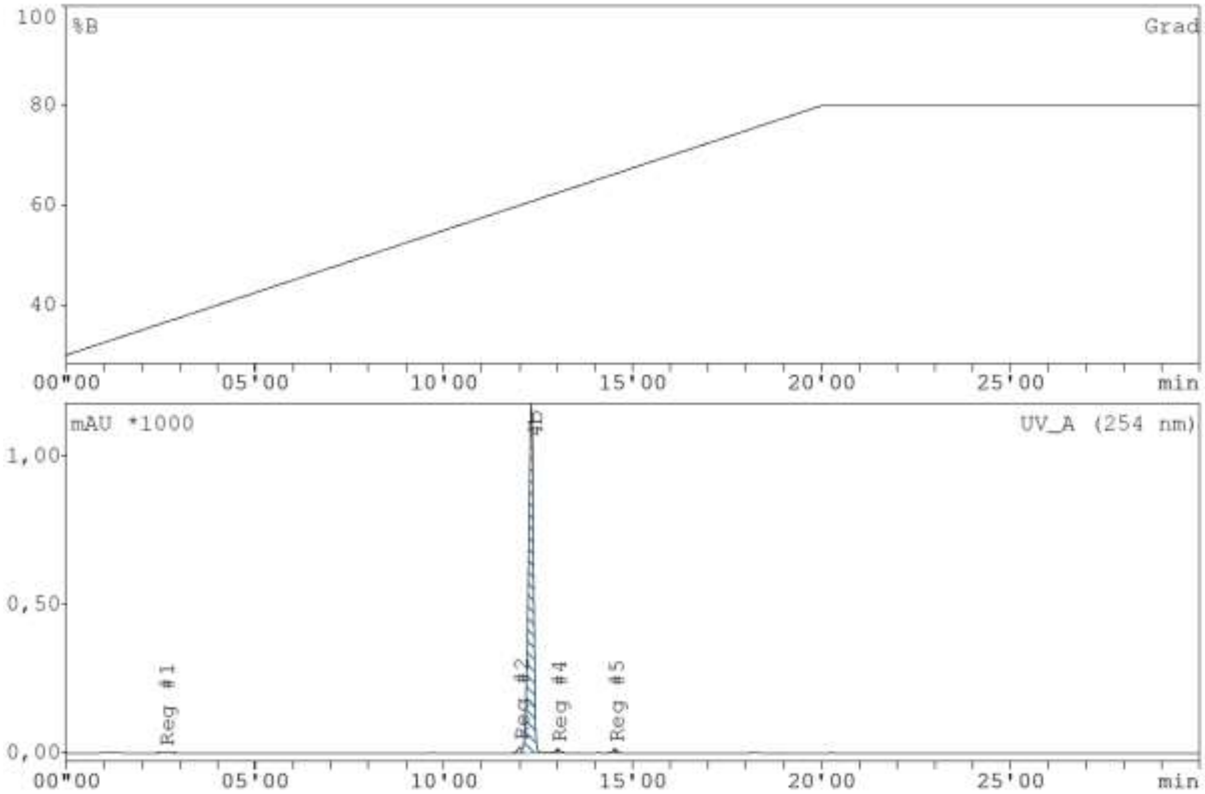

Integration UV_A (254 nm)

Substance	R/T s	Type	Area mAU*s	%Area %
Reg #1	02'37	BB	20,74	0,19
Reg #2	12'01	BD	128,26	1,16
4b	12'19	DB	10664,66	96,83
Reg #4	13'01	BB	101,29	0,92
Reg #5	14'32	BB	99,12	0,90
Sum in ROI			11014,07	100,00

Figure S23: (5-Chloro-2-((2-cyanopyridin-4-yl)methoxy)-4-((3-(2,3-dihydrobenzo[*b*][1,4]dioxin-6-yl)-2-methylbenzyl)oxy)benzyl)-*D*-serine (**4c**):

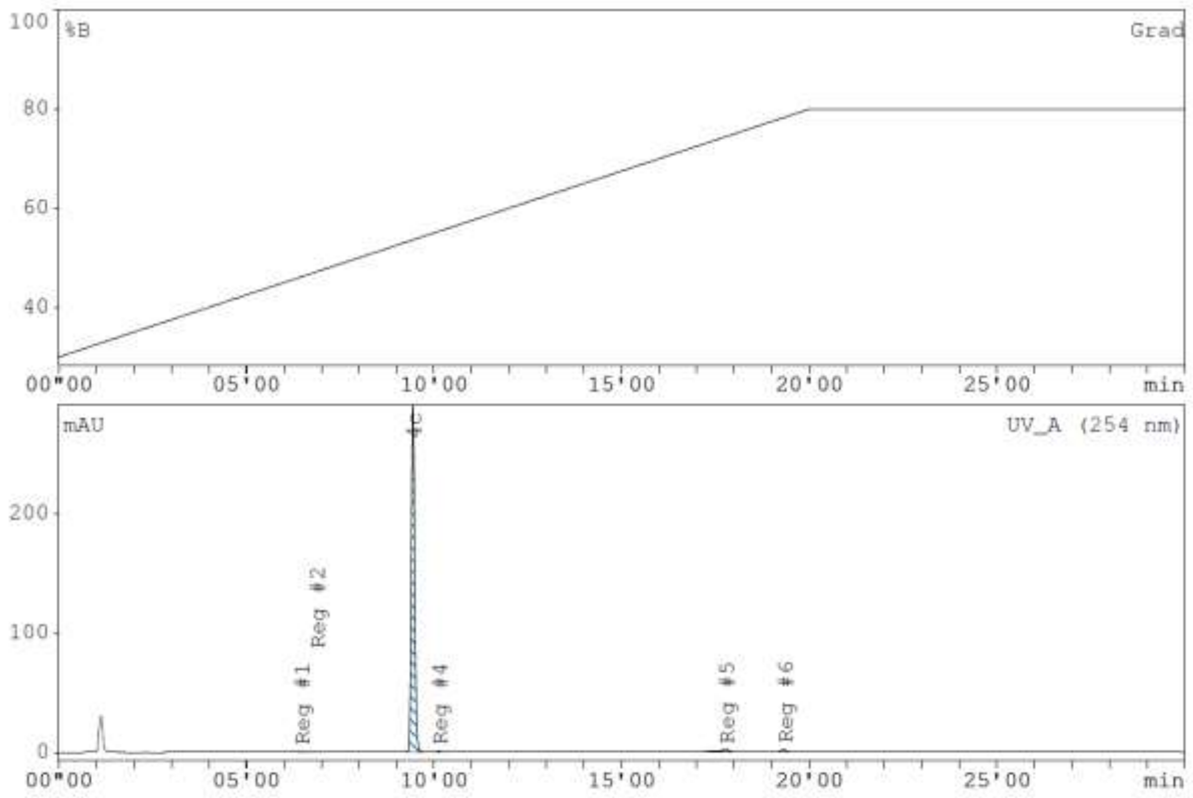

Integration UV_A (254 nm)

Substance	R/T s	Type	Area mAU*s	%Area %
Reg #1	06'27	BD	4,159	0,22
Reg #2	06'51	DB	3,314	0,18
4c	09'26	BB	1813,080	96,55
Reg #4	10'07	BB	4,428	0,24
Reg #5	17'46	BB	35,515	1,89
Reg #6	19'19	BB	17,412	0,93
Sum in ROI			1877,908	100,00

Figure S24: *N*-(2-((5-chloro-2-((2-cyanopyridin-4-yl)methoxy)-4-((3-(2,3-dihydrobenzo[*b*][1,4]dioxin-6-yl)-2-methylbenzyl)oxy)benzyl)amino)ethyl)acetamide (**4d**):

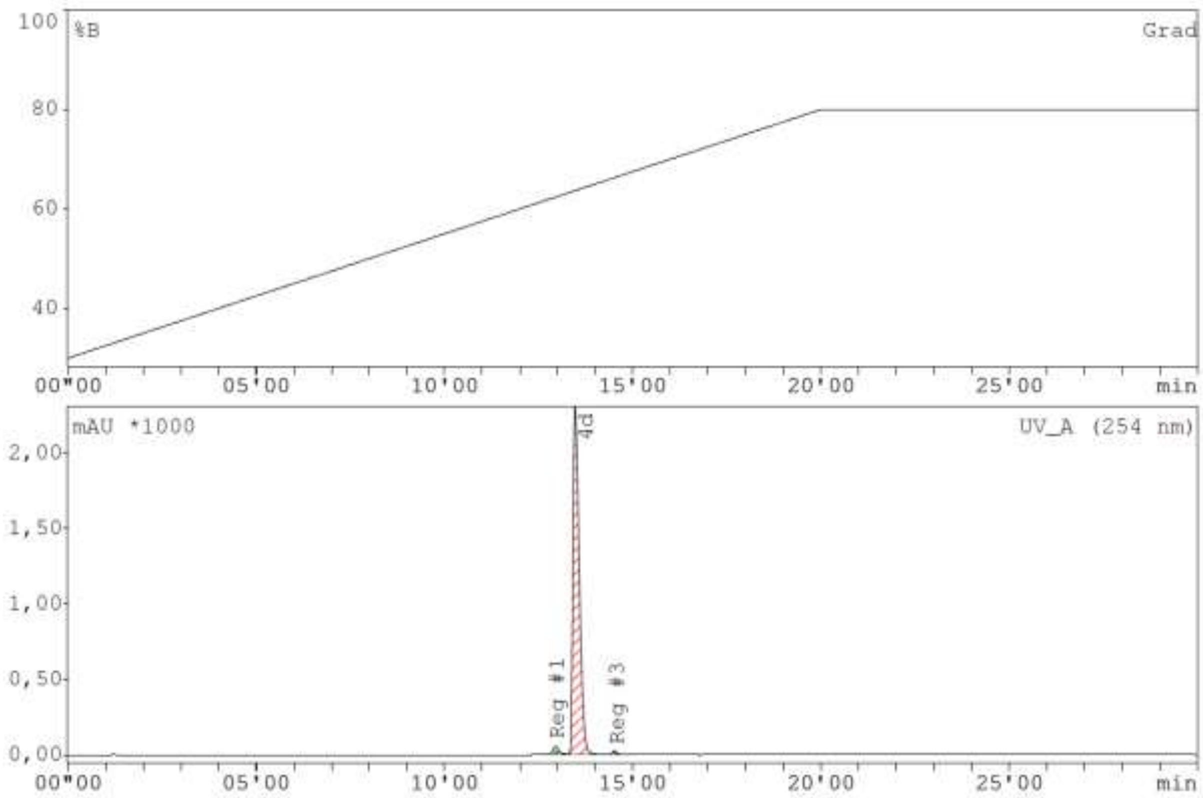

Integration UV_A (254 nm)

Substance	R/T s	Type	Area mAU*s	%Area %
Reg #1	12'57	BD	623,53	2,27
4d	13'29	DD	26642,41	96,91
Reg #3	14'31	DB	226,05	0,82
Sum in ROI			27491,98	100,00

Figure S25: 4-((4-Chloro-5-((3-(2,3-dihydrobenzo[*b*][1,4]dioxin-6-yl)-2-methylbenzyl)oxy)-2-(((2-hydroxyethyl)amino)methyl)phenoxy)methyl)picolinonitrile (**4e**):

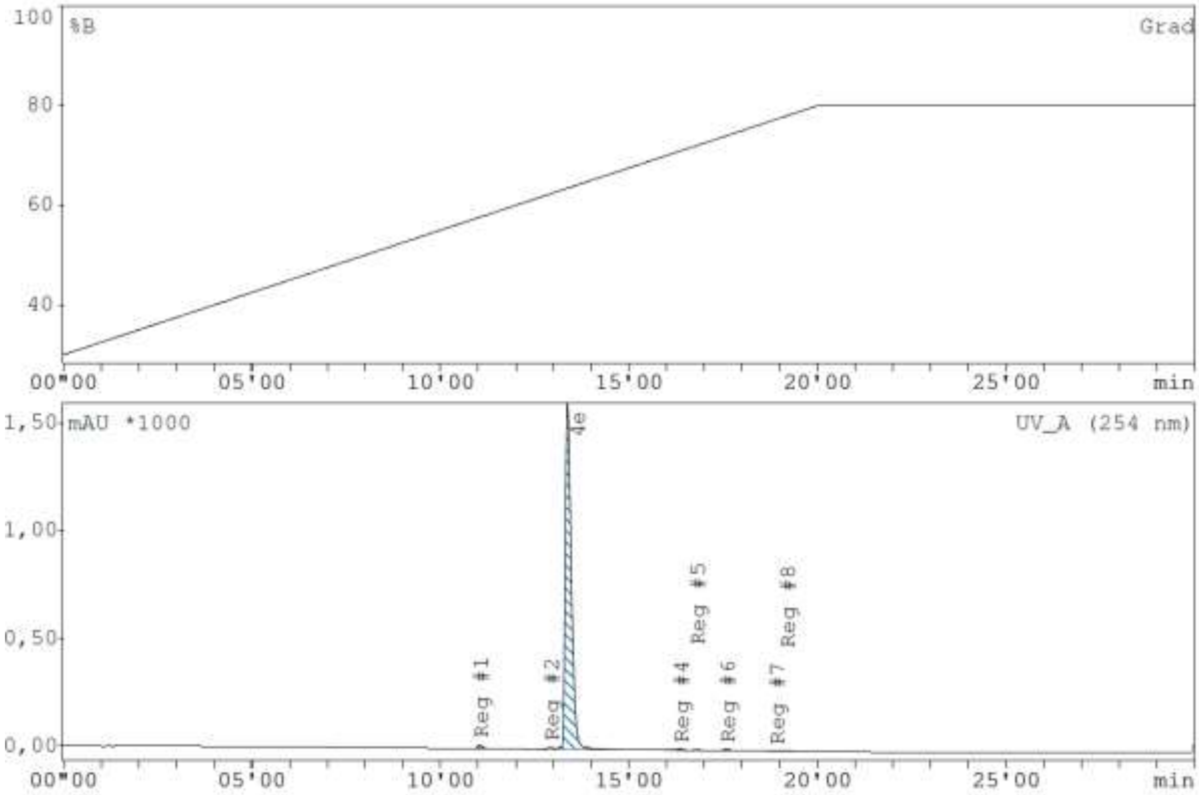

Integration UV_A (254 nm)

Substance	R/T s	Type	Area mAU*s	%Area %
Reg #1	11'03	BB	138,14	0,74
Reg #2	12'55	BD	65,96	0,35
4e	13'23	DB	18239,61	97,61
Reg #4	16'21	BB	83,64	0,45
Reg #5	16'48	DB	23,24	0,12
Reg #6	17'35	BB	81,10	0,43
Reg #7	18'53	BD	20,88	0,11
Reg #8	19'10	DB	34,04	0,18
Sum in ROI			18686,61	100,00

Figure S26: (*S*)-1-(5-chloro-2-((2-cyanopyridin-4-yl)methoxy)-4-((3-(2,3-dihydrobenzo[*b*][1,4]dioxin-6-yl)-2-methylbenzyl)oxy)benzyl)piperidine-2-carboxylic acid (**4f**):

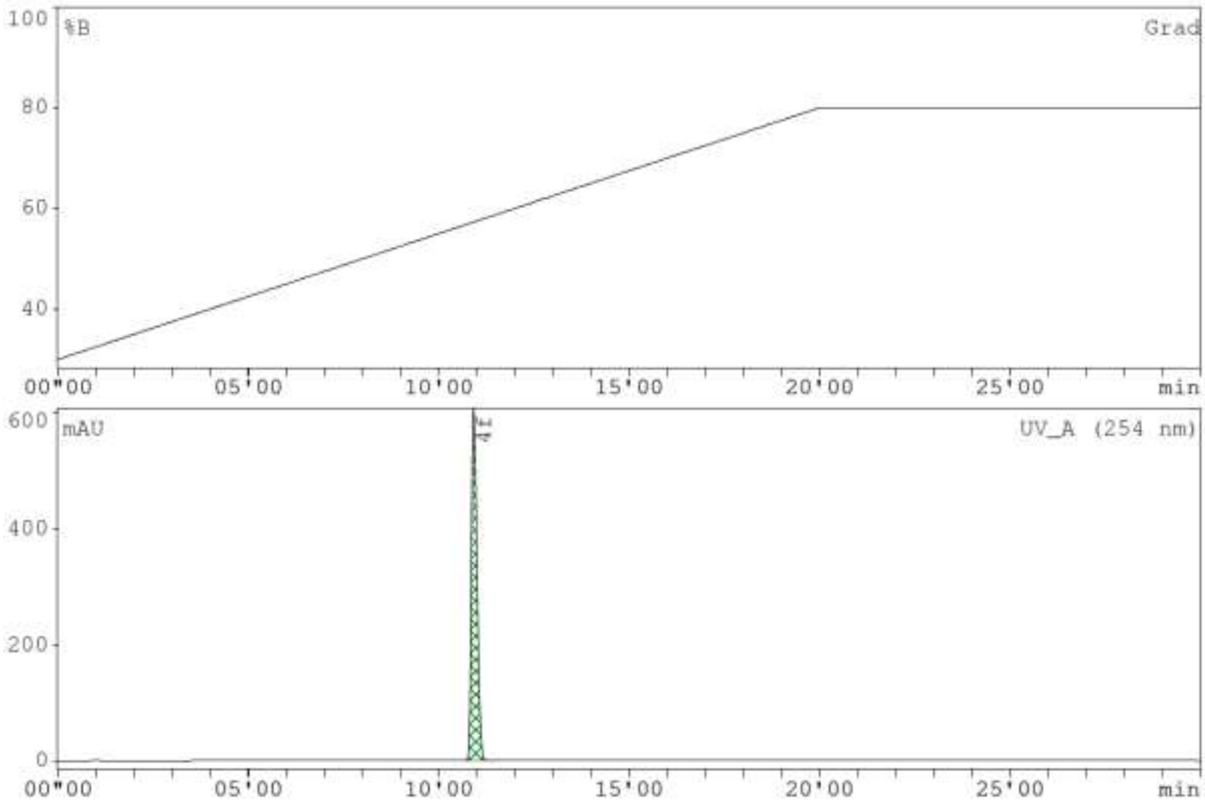

Integration UV_A (254 nm)

Substance	R/T s	Type	Area mAU*s	%Area %
4f	10'55	BB	6321,001	100,00
Sum in ROI			6321,001	100,00

Figure S27: 1-(5-Chloro-2-((2-cyanopyridin-4-yl)methoxy)-4-((3-(2,3-dihydrobenzo[*b*][1,4]dioxin-6-yl)-2-methylbenzyl)oxy)phenyl)-5,8,11,14-tetraoxa-2-azaheptadecan-17-oic acid (**4g**):

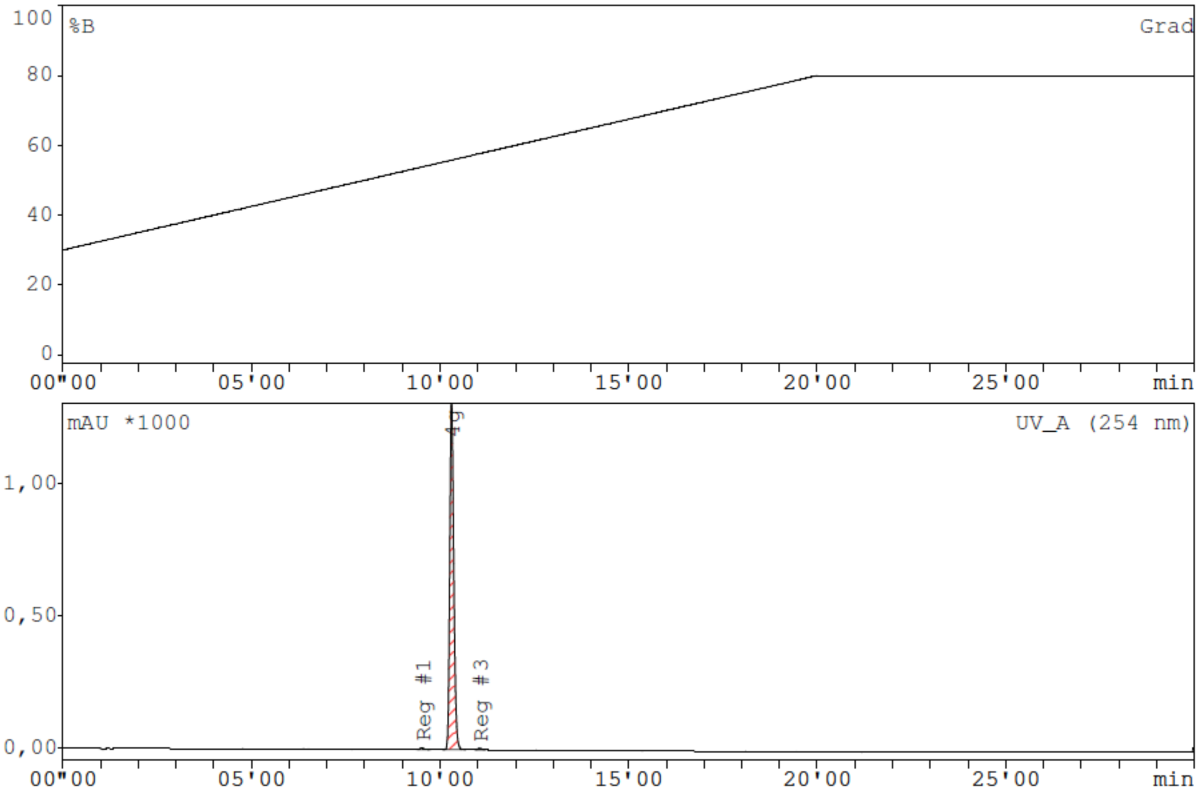

Integration UV_A (254 nm)

Substance	R/T s	Type	Area mAU*s	%Area %
Reg #1	09'31	BB	46,216	0,46
4g	10'19	BB	9945,091	99,15
Reg #3	11'03	BB	39,119	0,39
Sum in ROI			10030,426	100,00

Figure S28: (*S*)-2-((5-chloro-2-((2-cyanopyridin-4-yl)methoxy)-4-((3-(2,3-dihydrobenzo[*b*][1,4]dioxin-6-yl)-2-methylbenzyl)oxy)benzyl)amino)hex-5-ynoic acid (**4h**):

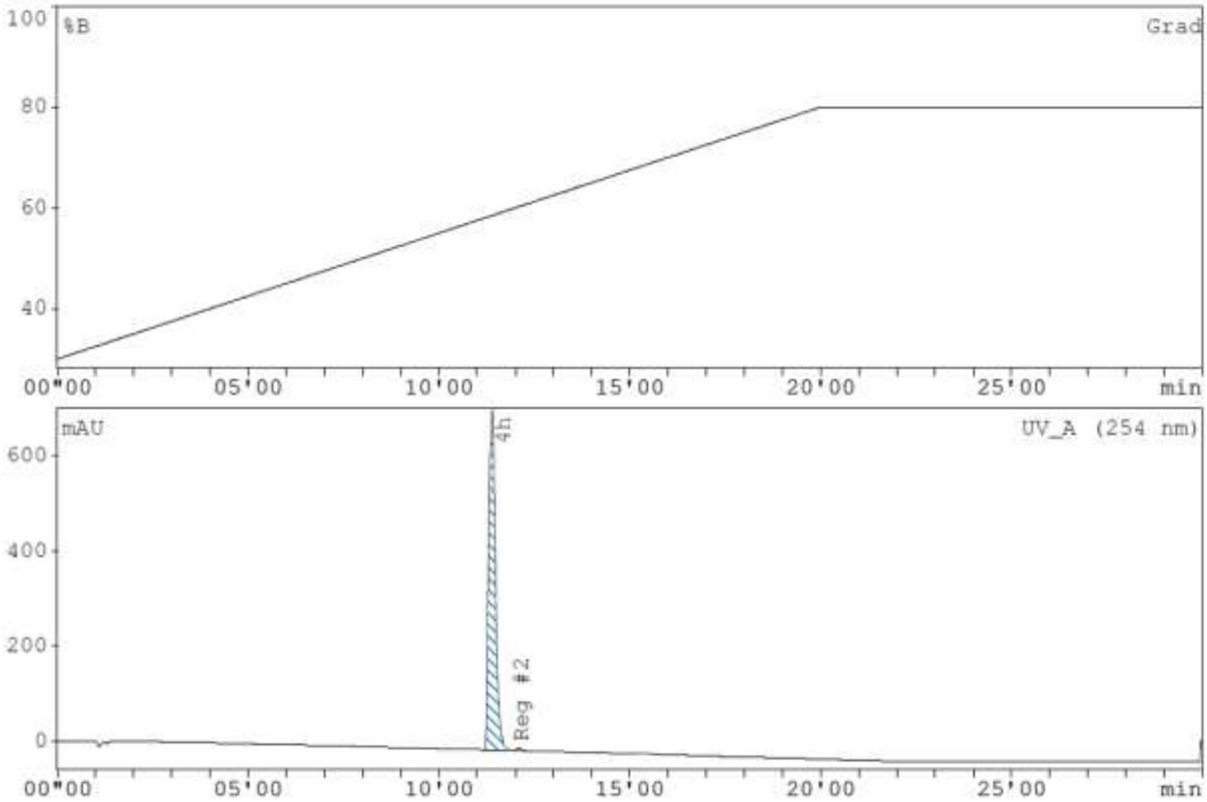

Integration UV_A (254 nm)

Substance	R/T s	Type	Area mAU*s	%Area %
4h	10'51	BD	8148,679	98,05
Reg #2	11'48	DD	162,05	1,45

Figure S29: N^2-(5-chloro-2-((2-cyanopyridin-4-yl)methoxy)-4-((3-(2,3-dihydrobenzo[*b*][1,4]dioxin-6-yl)-2-methylbenzyl)oxy)benzyl)-N^6-((prop-2-yn-1-yloxy)carbonyl)-L-lysine (**4i**):

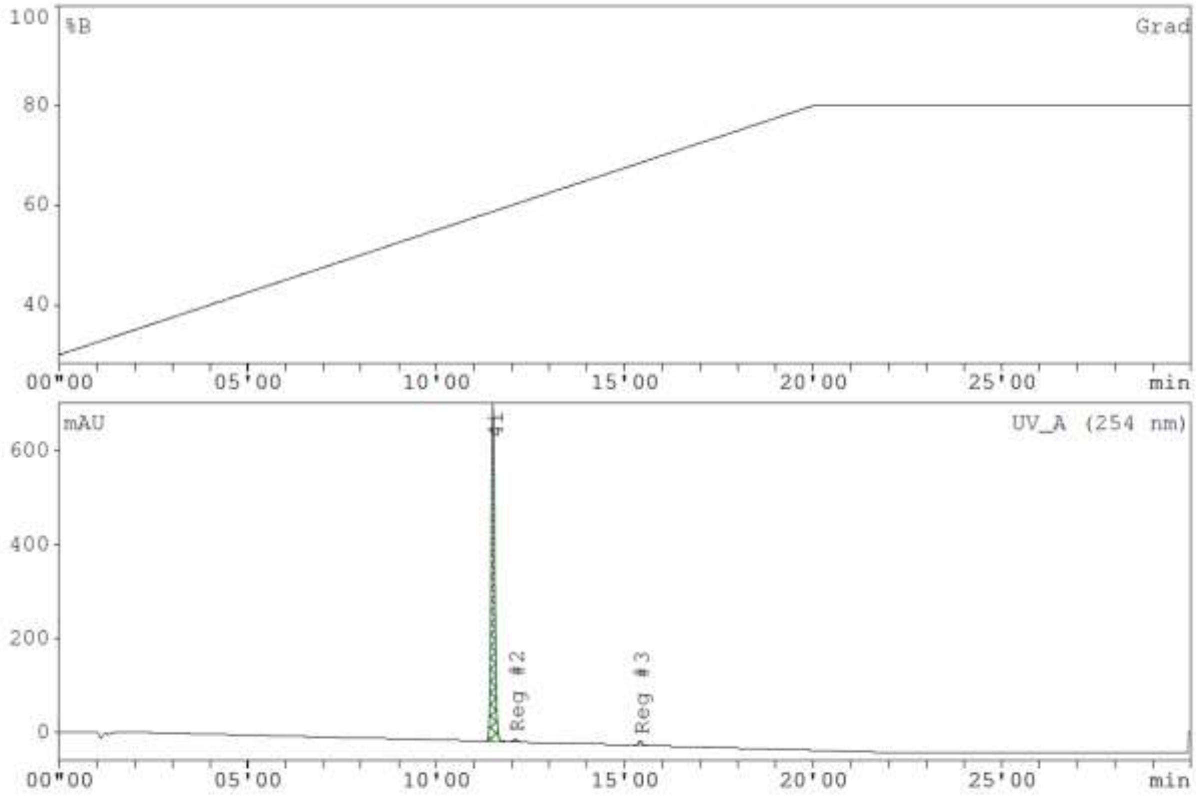

Integration UV_A (254 nm)

Substance	R/T s	Type	Area mAU*s	%Area %
4i	11'30	BD	4750,757	97,79
Reg #2	12'06	DB	42,868	0,88
Reg #3	15'24	BB	64,427	1,33
Sum in ROI			4858,052	100,00

Figure S30: *N*-(2-((5-chloro-2-((3-cyanobenzyl)oxy)-4-((3-(2,3-dihydrobenzo[*b*][1,4]dioxin-6-yl)-2-methylbenzyl)oxy)benzyl)amino)ethyl)acetamide (**4j**):

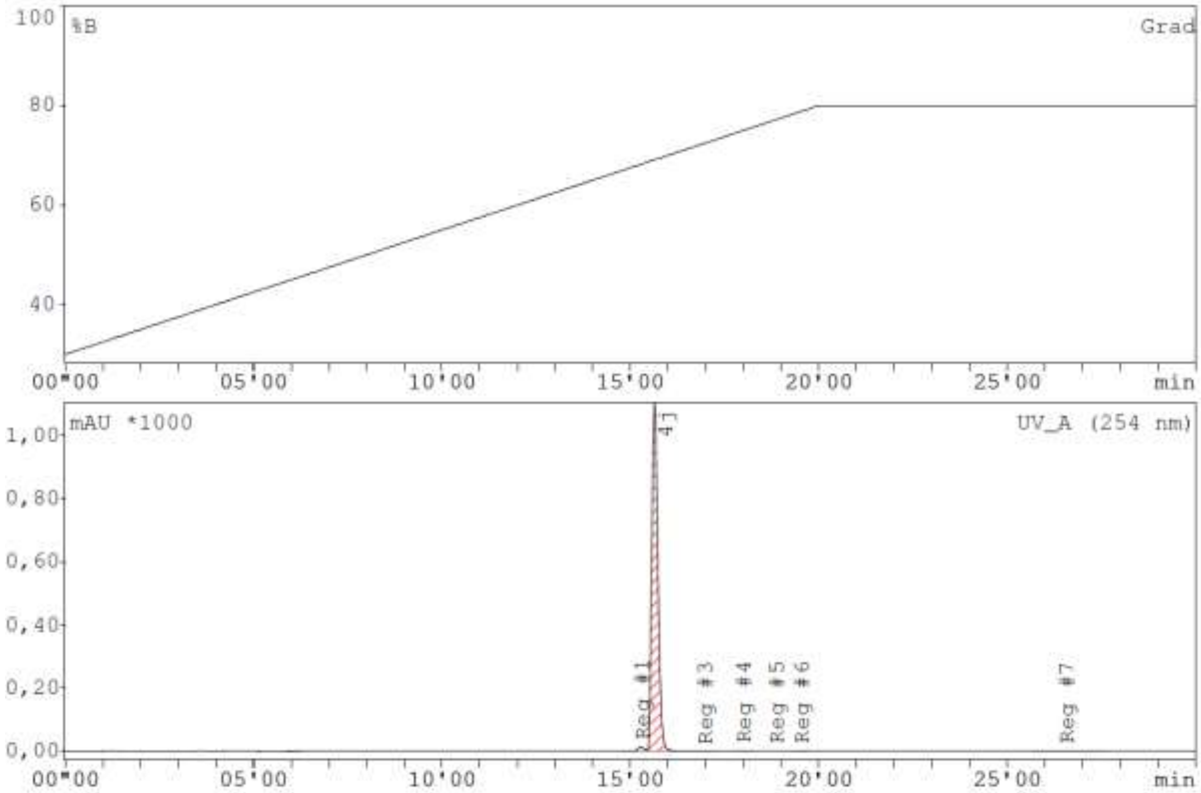

Integration UV_A (254 nm)

Substance	R/T s	Type	Area mAU*s	%Area %
Reg #1	15'17	BD	140,82	1,12
4j	15'39	DD	12390,87	98,21
Reg #3	16'56	DB	10,52	0,08
Reg #4	17'59	BB	13,93	0,11
Reg #5	18'51	BB	2,79	0,02
Reg #6	19'31	BB	8,04	0,06
Reg #7	26'32	BB	50,26	0,40
Sum in ROI			12617,23	100,00

Figure S31: *N*-(2-((5-chloro-4-((3-(2,3-dihydrobenzo[*b*][1,4]dioxin-6-yl)-2-methylbenzyl)oxy)-2-(oxazol-4-ylmethoxy)benzyl)amino)ethyl)acetamide (**4k**):

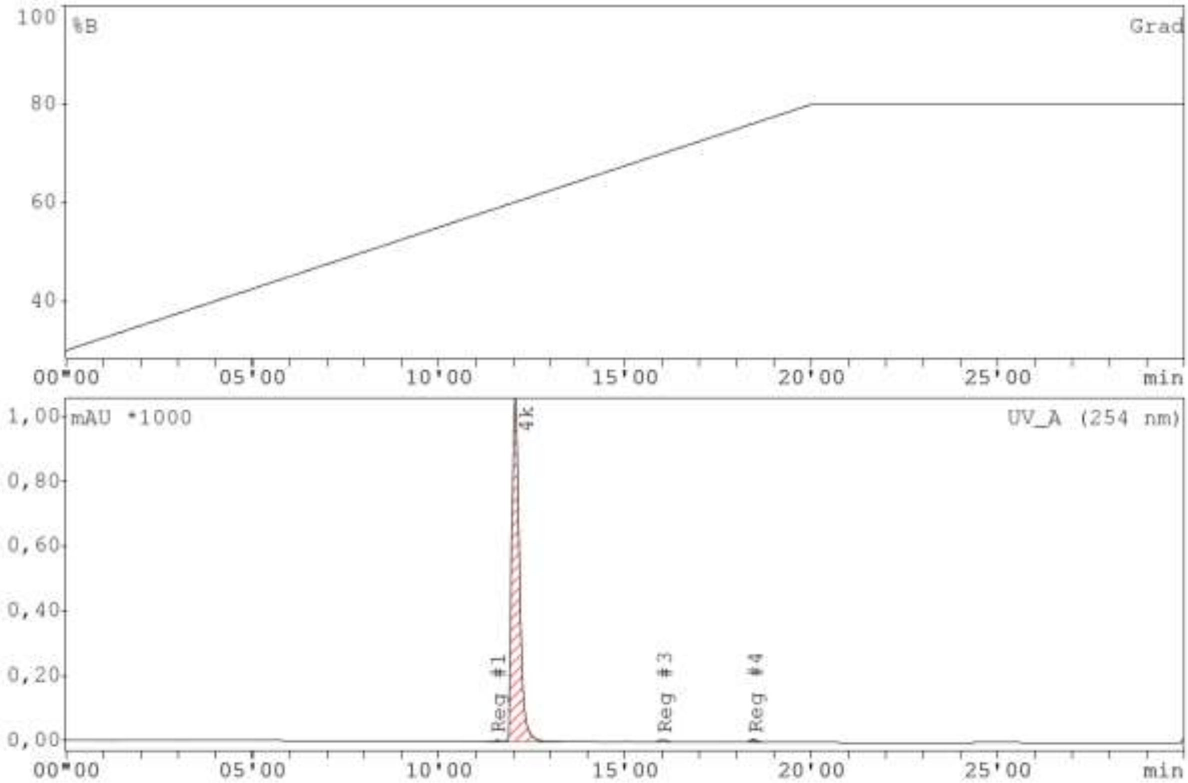

Integration UV_A (254 nm)

Substance	R/T s	Type	Area mAU*s	%Area %
Reg #1	11'34	BD	31,18	0,20
4k	12'03	DB	15030,49	98,62
Reg #3	16'01	BB	69,92	0,46
Reg #4	18'27	BB	109,16	0,72
Sum in ROI			15240,75	100,00

Figure S32: (*S*)-1-(5-chloro-4-((3-(2,3-dihydrobenzo[*b*][1,4]dioxin-6-yl)-2-methylbenzyl)oxy)-2-(oxazol-4-ylmethoxy)benzyl)piperidine-2-carboxylic acid (**4l**):

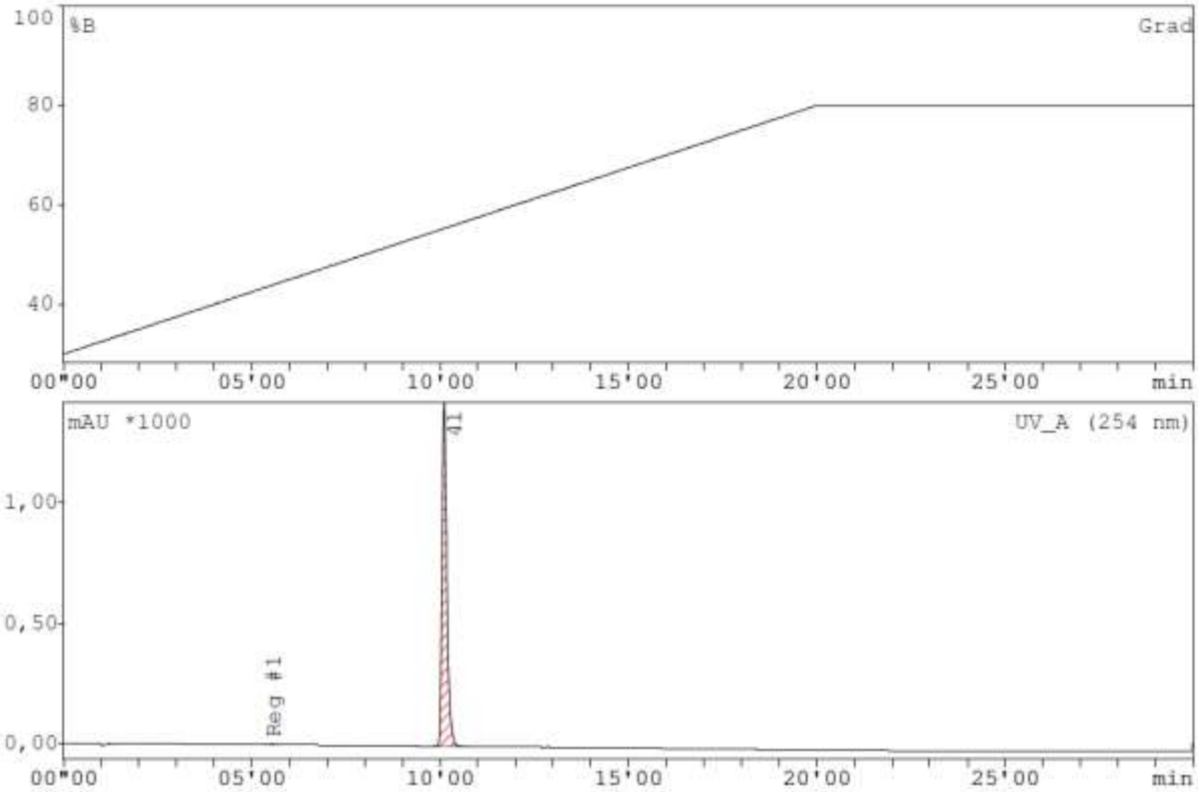

Integration UV_A (254 nm)

Substance	R/T s	Type	Area mAU*s	%Area %
Reg #1	05'33	BB	19,39	0,15
4l	10'07	BB	12657,23	99,85
Sum in ROI			12676,62	100,00

Figure S33: (5-Chloro-2-((2-cyanopyridin-4-yl)methoxy)-4-((2-methyl-3-(1*H*-pyrrol-1-yl)benzyl)oxy)benzyl)-*D*-serine (**4m**):

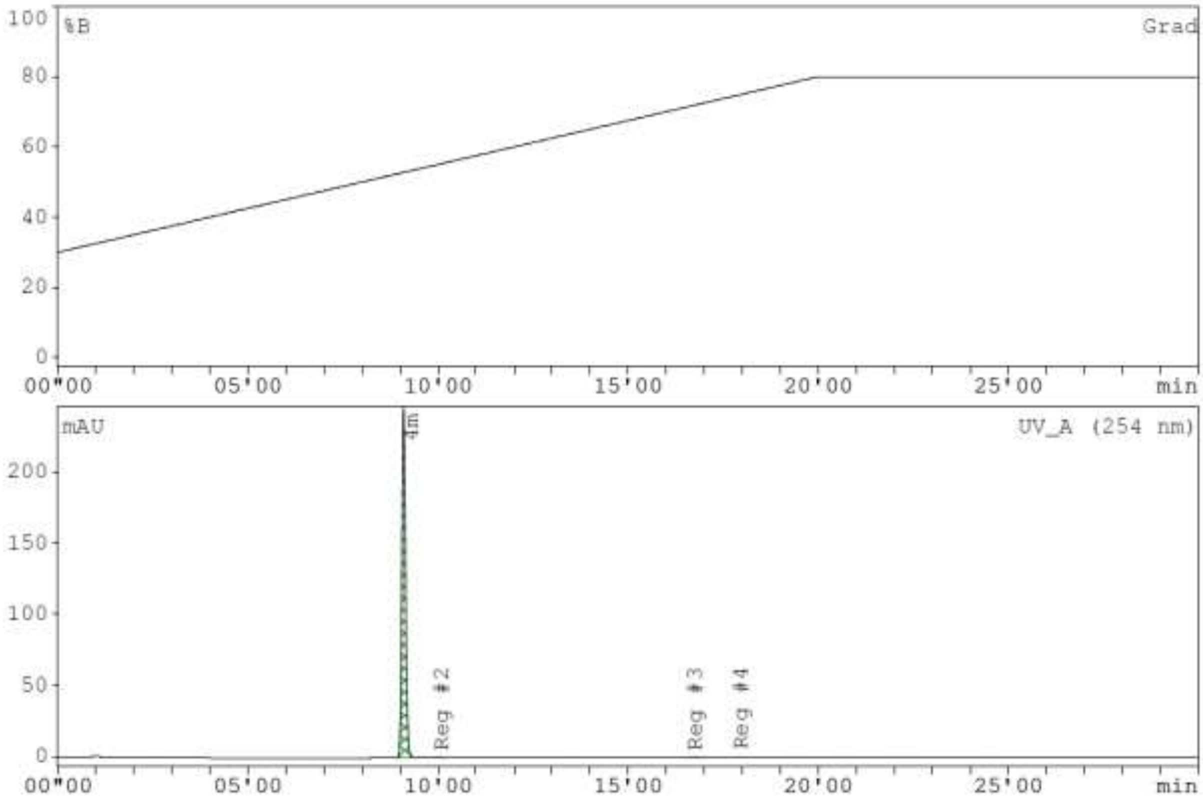

Integration UV_A (254 nm)

Substance	R/T s	Type	Area mAU*s	%Area %
4m	09'06	BB	1690,147	99,16
Reg #2	10'02	BB	4,115	0,24
Reg #3	16'42	BB	4,887	0,29
Reg #4	17'56	BB	5,307	0,31
Sum in ROI			1704,455	100,00

Figure S34: 4-((4-Chloro-2-(((2-hydroxyethyl)amino)methyl)-5-((2-methyl-3-(1*H*-pyrrol-1-yl)benzyl)oxy)phenoxy)methyl)picolinonitrile (**4n**):

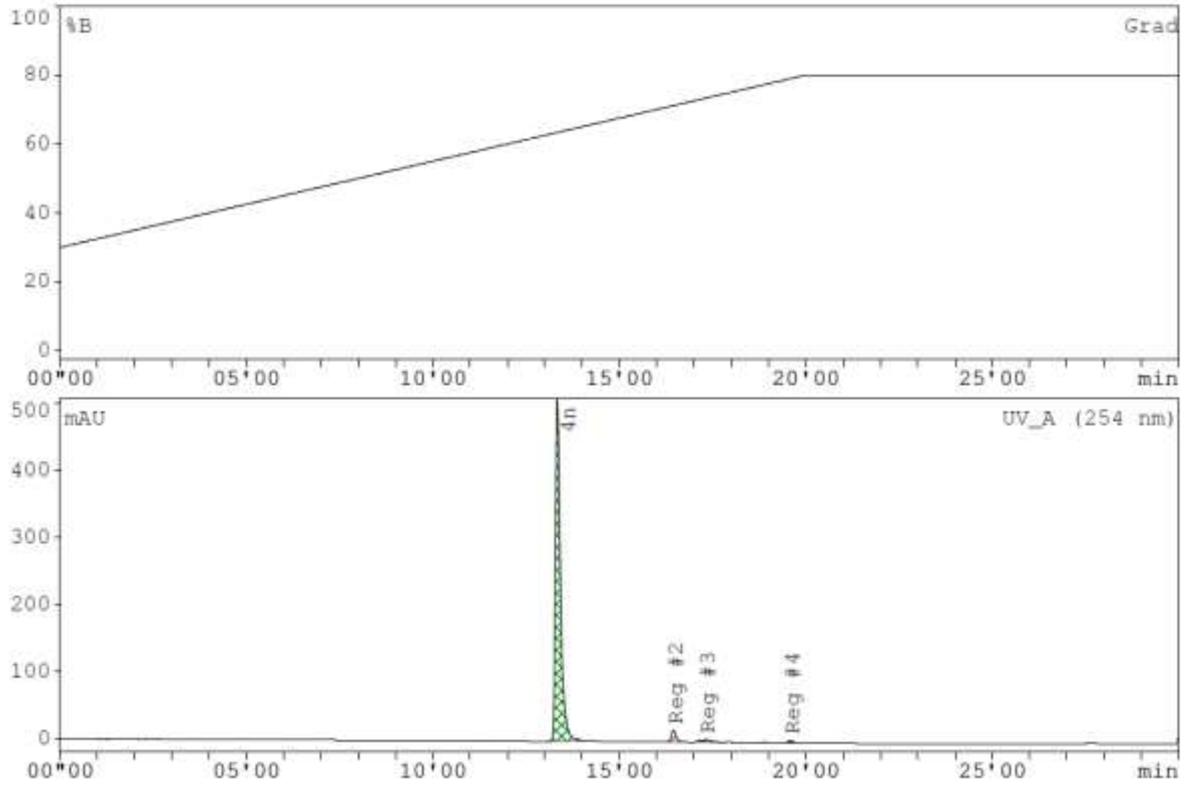

Integration UV_A (254 nm)

Substance	R/T s	Type	Area mAU*s	%Area %
4n	13'21	BB	4961,846	95,19
Reg #2	16'28	BB	148,744	2,85
Reg #3	17'20	BB	71,479	1,37
Reg #4	19'36	BB	30,666	0,59
Sum in ROI			5212,734	100,00

Figure S35: (*S*)-1-(5-chloro-2-((2-cyanopyridin-4-yl)methoxy)-4-((2-methyl-3-(1*H*-pyrrol-1-yl)benzyl)oxy)benzyl)piperidine-2-carboxylic acid (**4o**):

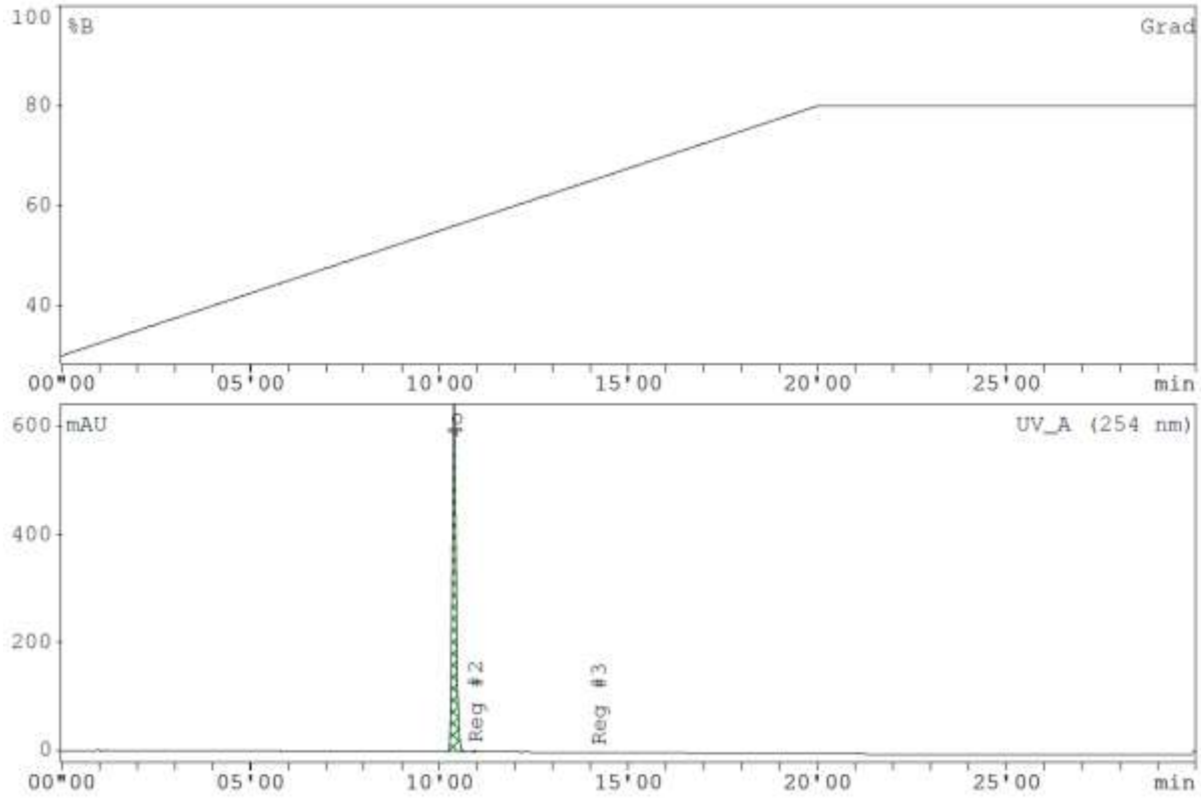

Integration UV_A (254 nm)

Substance	R/T s	Type	Area mAU*s	%Area %
4o	10'23	BD	4602,464	99,54
Reg #2	10'55	DB	16,123	0,35
Reg #3	14'10	BB	5,101	0,11
Sum in ROI			4623,688	100,00

Figure S36: *N*-(5-chloro-2-((2-cyanopyridin-4-yl)methoxy)-4-((3-(2,3-dihydrobenzo[*b*][1,4]dioxin-6-yl)-2-methylbenzyl)oxy)benzyl)-*N*-methyl-*D*-serine (**5a**):

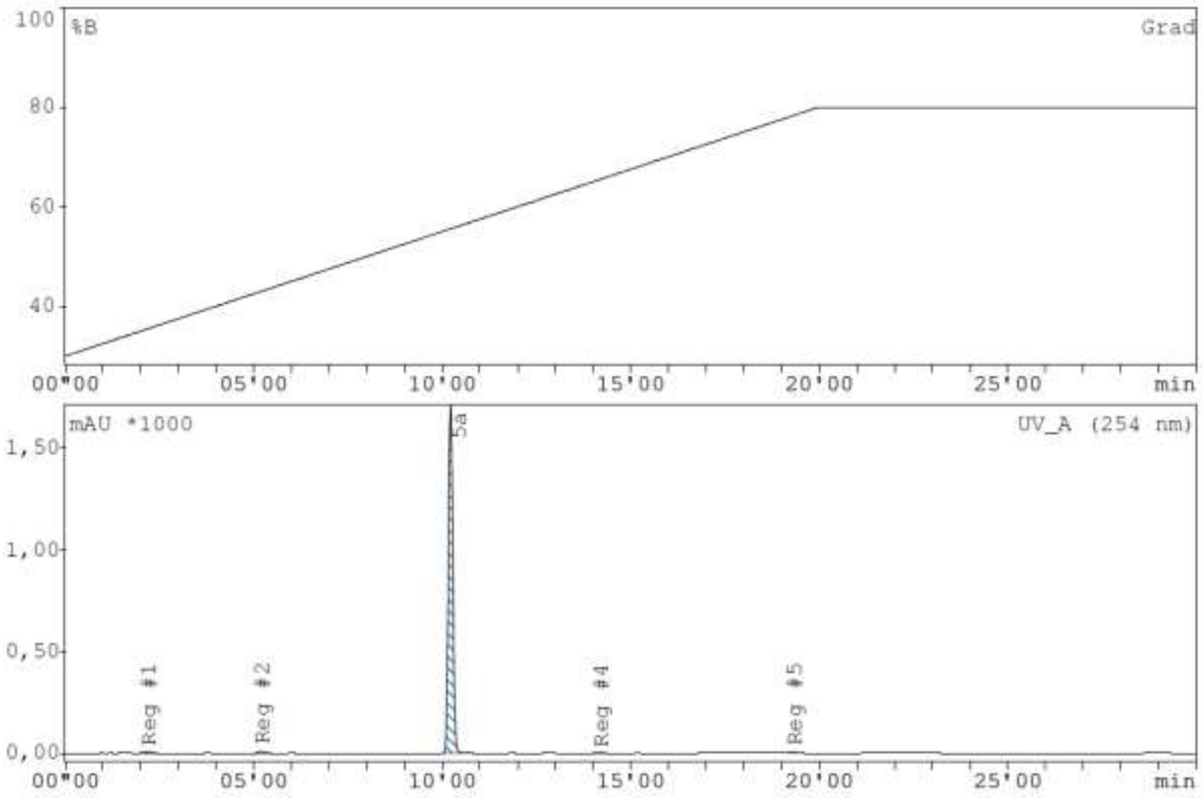

Integration UV_A (254 nm)

Substance	R/T s	Type	Area mAU*s	%Area %
Reg #1	02'10	BB	90,27	0,64
Reg #2	05'11	BB	95,87	0,68
5a	10'13	BB	13791,13	98,08
Reg #4	14'10	BB	23,40	0,17
Reg #5	19'18	BB	59,80	0,43
Sum in ROI			14060,48	100,00

Figure S37: Methyl (5-chloro-2-((2-cyanopyridin-4-yl)methoxy)-4-((3-(2,3-dihydrobenzo[*b*][1,4]dioxin-6-yl)-2-methylbenzyl)oxy)benzyl)-*D*-serinate (**5b**):

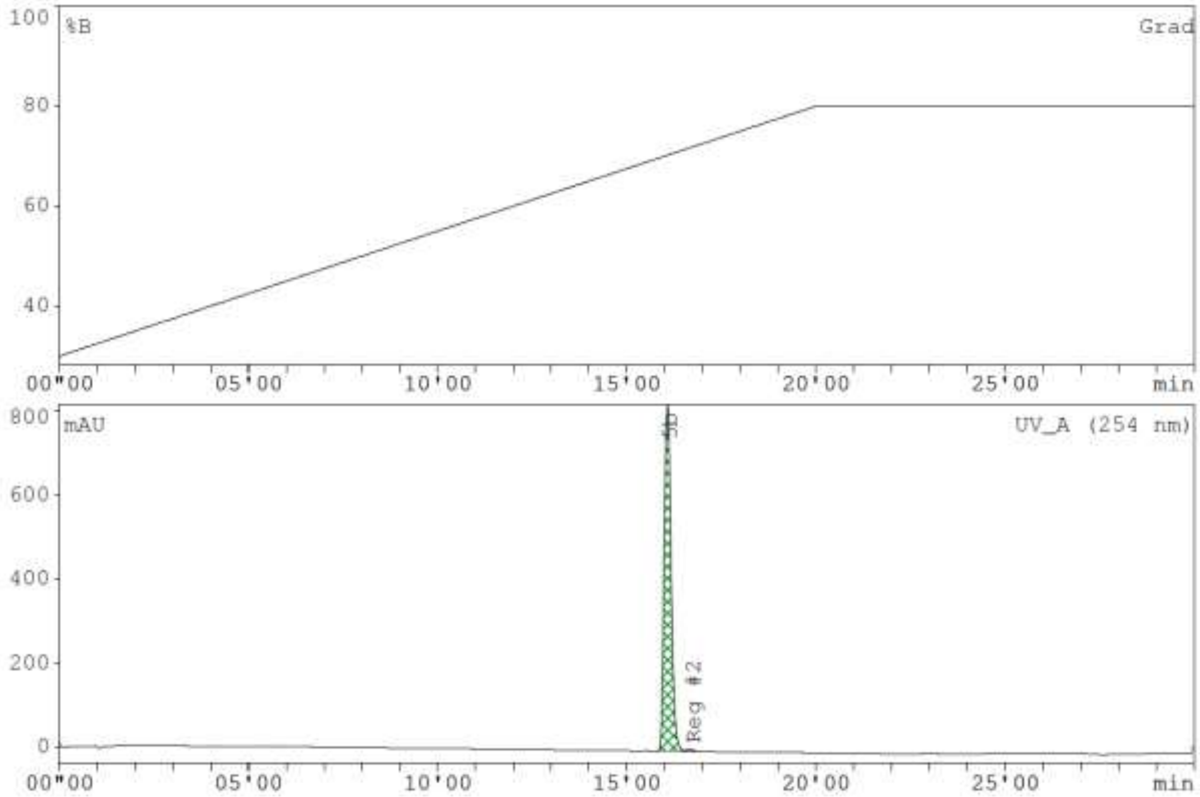

Integration UV_A (254 nm)

Substance	R/T s	Type	Area mAU*s	%Area %
5b	16'05	BD	10543,91	99,43
Reg #2	16'43	DB	60,88	0,57
Sum in ROI			10604,79	100,00

Figure S38: 4-((4-Chloro-5-((3-(2,3-dihydrobenzo[*b*][1,4]dioxin-6-yl)-2-methylbenzyl)oxy)-2-(((2-hydroxyethyl)(methyl)amino)methyl)phenoxy)methyl)picolinonitrile (**5c**):

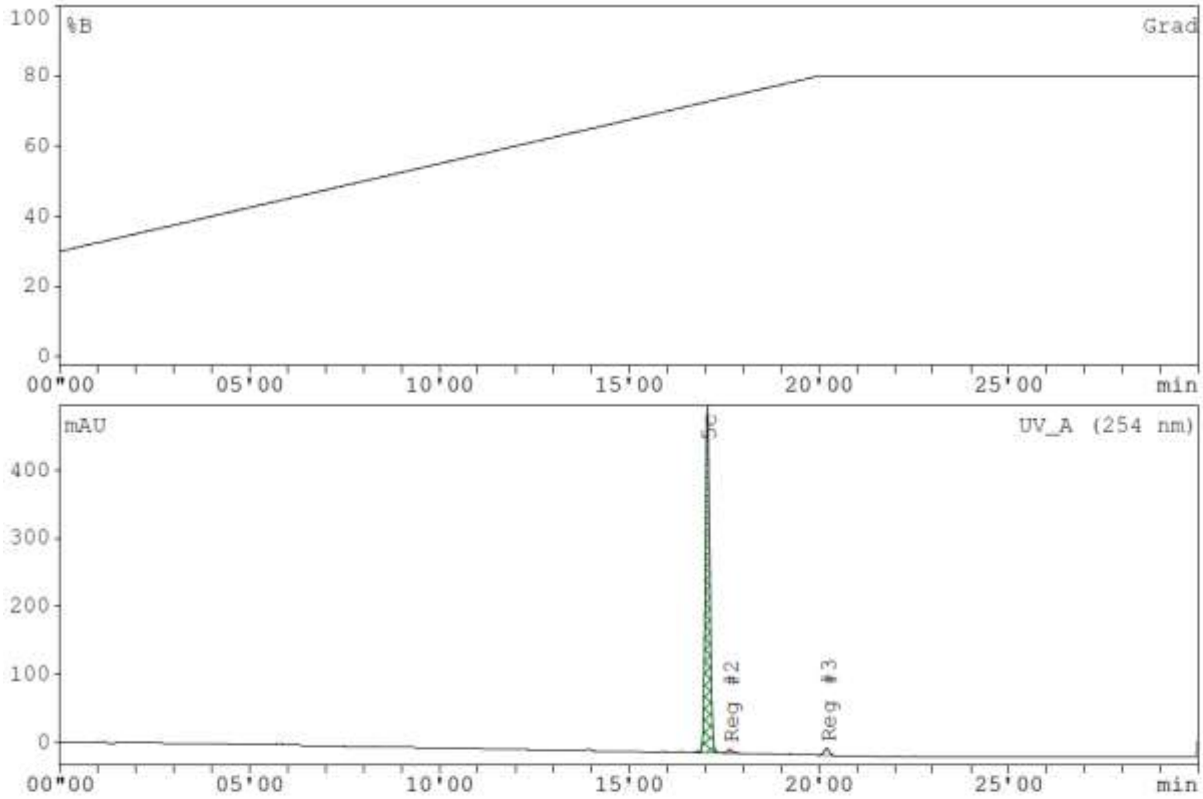

Integration UV_A (254 nm)

Substance	R/T s	Type	Area mAU*s	%Area %
5c	17'04	BB	4185,128	96,40
Reg #2	17'39	DB	57,954	1,33
Reg #3	20'13	BB	98,511	2,27
Sum in ROI			4341,592	100,00

Figure S39: 4-((4-Chloro-5-((3-(2,3-dihydrobenzo[*b*][1,4]dioxin-6-yl)-2-methylbenzyl)oxy)-2-(((2-methoxyethyl)amino)methyl)phenoxy)methyl)picolinonitrile (**5d**):

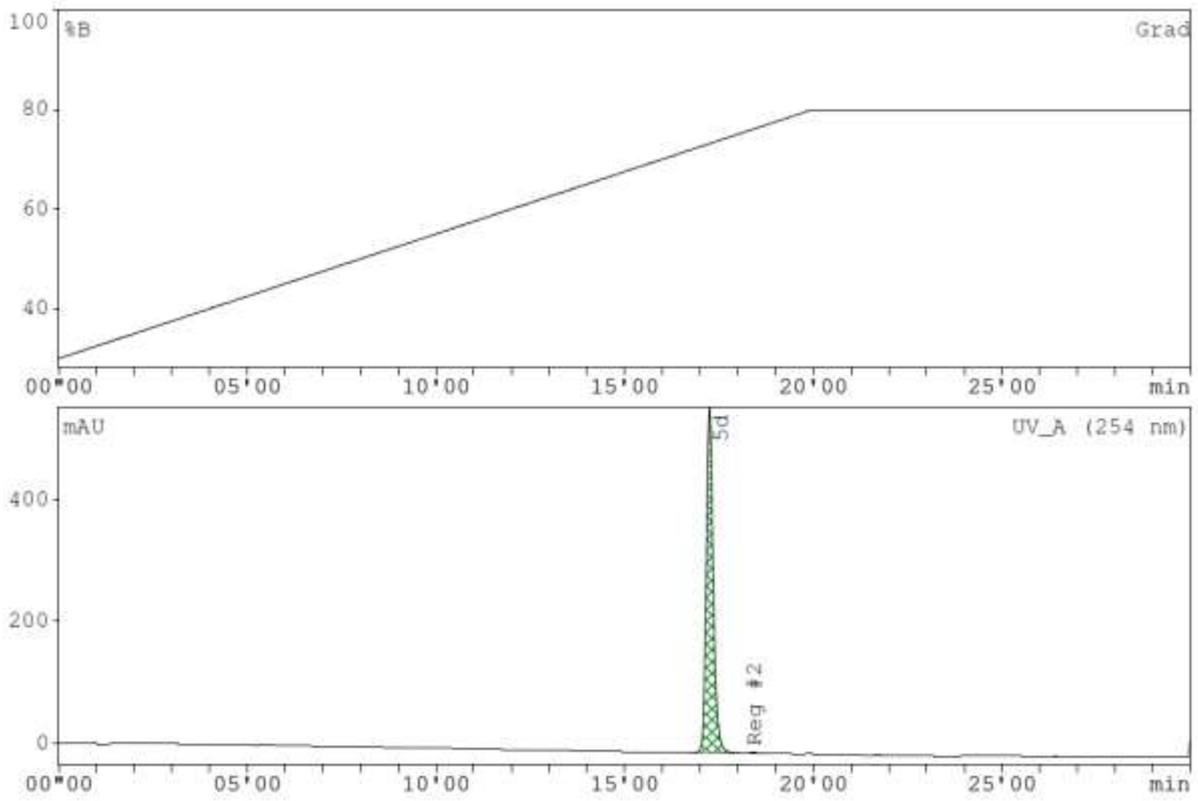

Integration UV_A (254 nm)

Substance	R/T s	Type	Area mAU*s	%Area %
5d	17'15	BD	7374,939	99,70
Reg #2	18'24	DB	22,335	0,30
Sum in ROI			7397,274	100,00

Figure S40: 3-((2-((3-Acetyl-2-oxoimidazolidin-1-yl)methyl)-4-chloro-5-((3-(2,3-dihydrobenzo[*b*][1,4]dioxin-6-yl)-2-methylbenzyl)oxy)phenoxy)methyl)benzonitrile (**5e**):

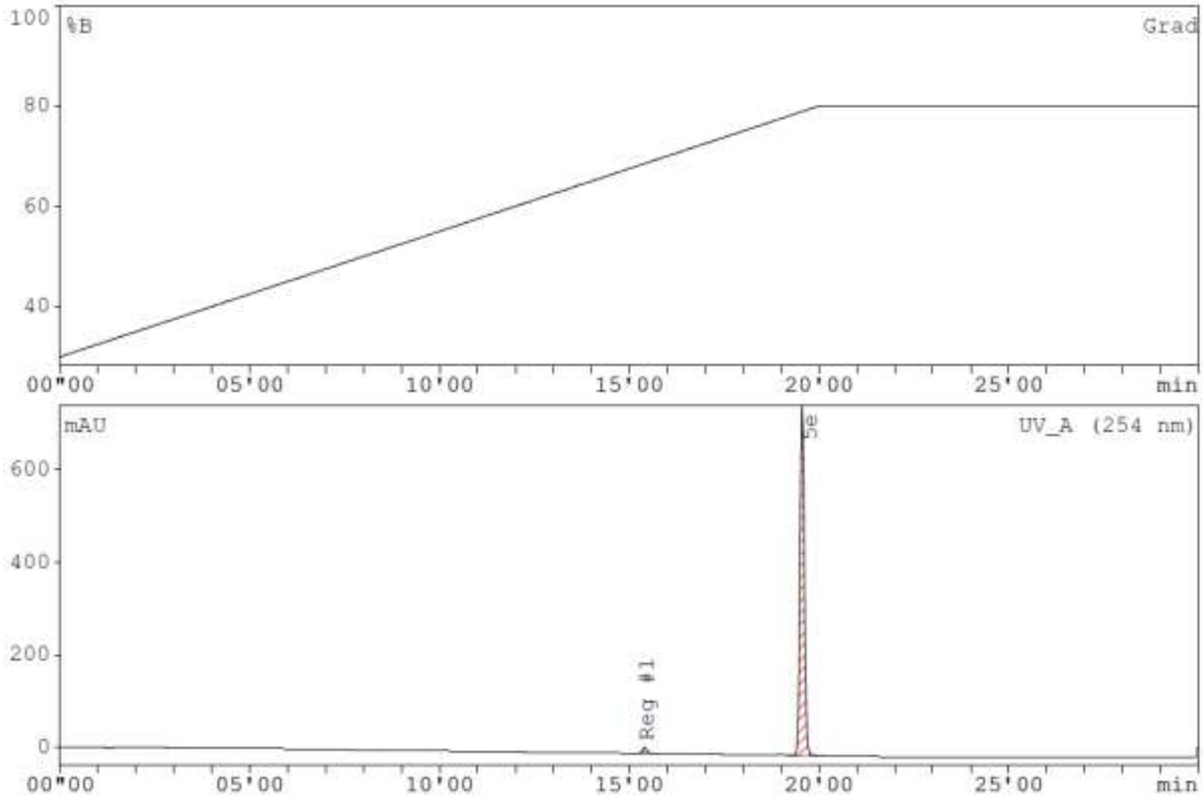

Integration UV_A (254 nm)

Substance	R/T s	Type	Area mAU*s	%Area %
Reg #1	15'25	BB	101,482	1,68
5e	19'34	DB	5924,896	98,32
Sum in ROI			6026,378	100,00

Figure S41: 4-((4-Chloro-2-(((2-hydroxyethyl)(methyl)amino)methyl)-5-((2-methyl-3-(1*H*-pyrrol-1-yl)benzyl)oxy)phenoxy)methyl)picolinonitrile (**5f**):

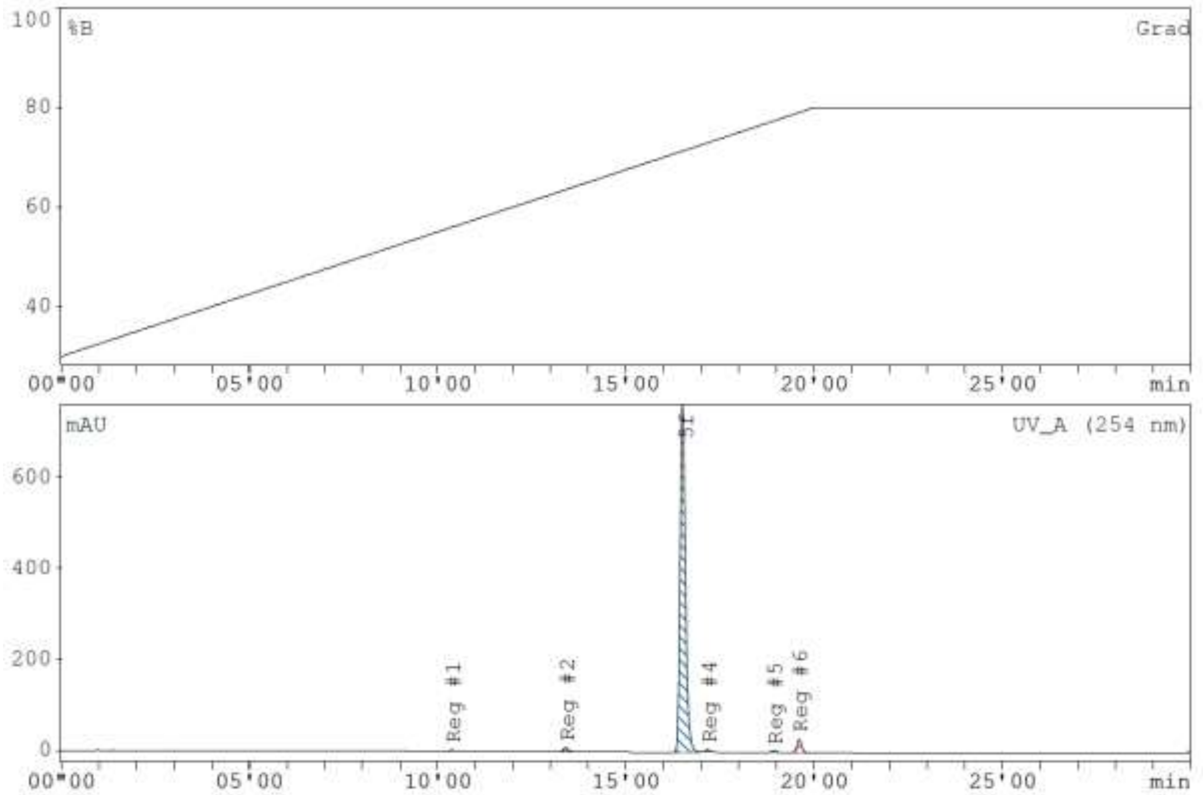

Integration UV_A (254 nm)

Substance	R/T s	Type	Area mAU*s	%Area %
Reg #1	10'23	BB	10,607	0,14
Reg #2	13'24	BB	79,957	1,02
5f	16'30	BD	7433,598	95,01
Reg #4	17'10	BB	22,753	0,29
Reg #5	18'55	BB	18,828	0,24
Reg #6	19'36	BB	258,126	3,30
Sum in ROI			7823,870	100,00

Figure S42: 4-((4-Chloro-5-((3-(2,3-dihydrobenzo[*b*][1,4]dioxin-6-yl)-2-methylbenzyl)oxy)-2-((2-(fluoromethyl)oxazolidin-3-yl)methyl)phenoxy)methyl)picolinonitrile (**5g**):

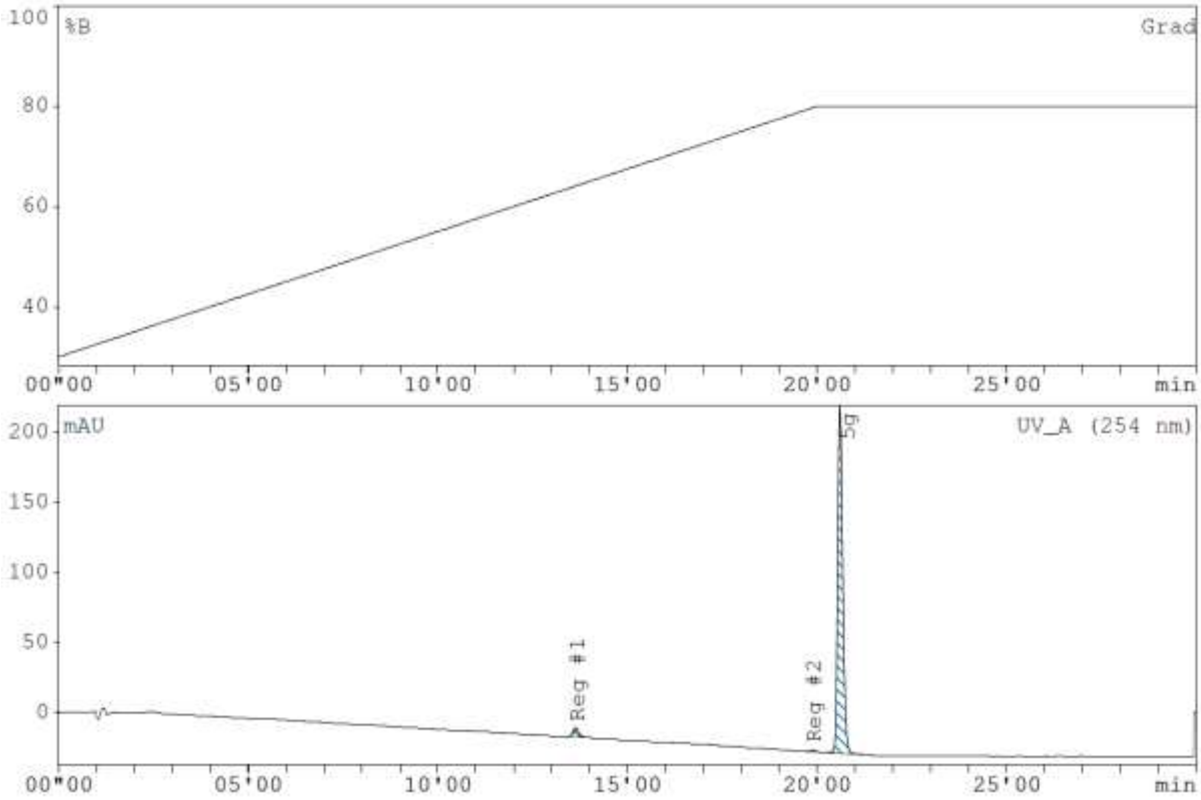

Integration UV_A (254 nm)

Substance	R/T s	Type	Area mAU*s	%Area %
Reg #1	13'38	BB	69,248	2,90
Reg #2	19'53	BB	12,799	0,54
5g	20'36	BB	2309,132	96,57
Sum in ROI			2391,179	100,00

Figure S43: 2-Fluoroethyl (*S*)-1-(5-chloro-2-((2-cyanopyridin-4-yl)methoxy)-4-((3-(2,3-dihydrobenzo[*b*][1,4]dioxin-6-yl)-2-methylbenzyl)oxy)benzyl)piperidine-2-carboxylate (**5h**):

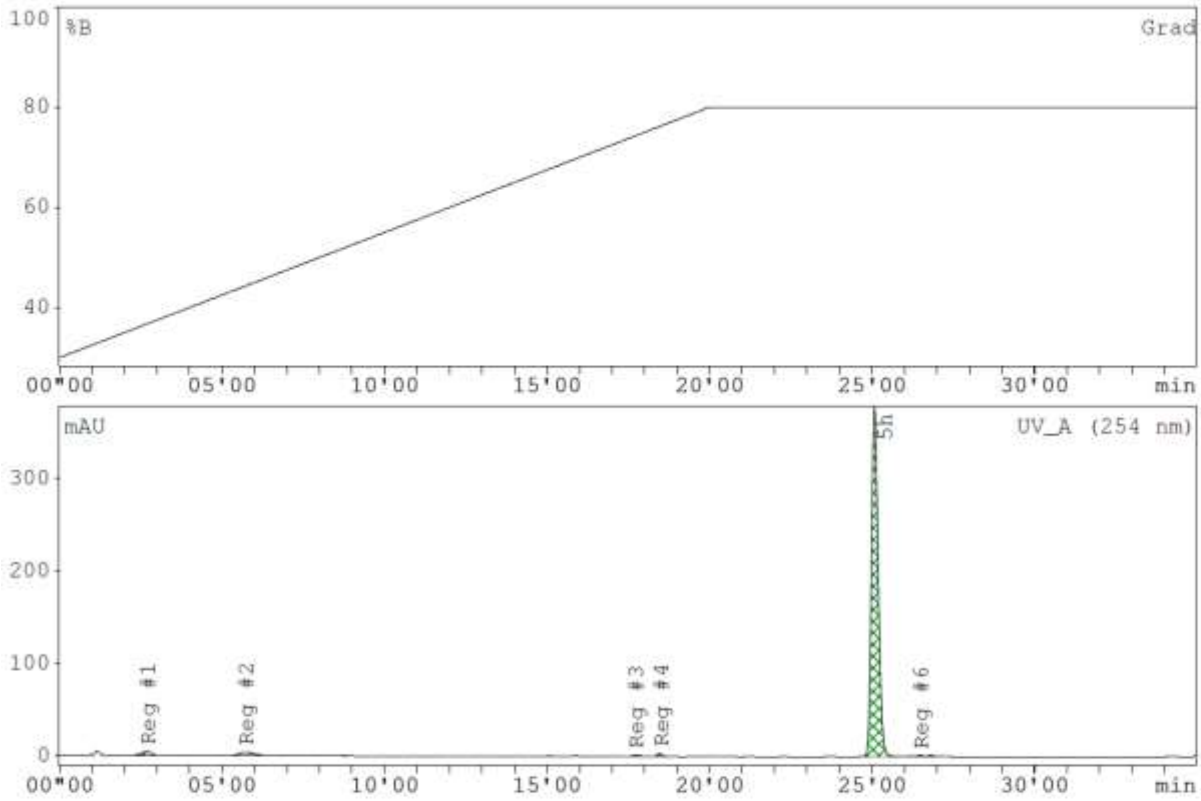

Integration UV_A (254 nm)

Substance	R/T<br>s	Type	Area<br>mAU*s	%Area<br>%
Reg #1	02'41	BB	91,333	1,59
Reg #2	05'42	BB	107,280	1,86
Reg #3	17'42	BB	18,178	0,32
Reg #4	18'28	BB	31,738	0,55
5h	25'05	BB	5464,992	95,00
Reg #6	26'28	BB	39,351	0,68
Sum in ROI			5752,872	100,00

Figure S44: 4-((4-Chloro-5-((3-(2,3-dihydrobenzo[*b*][1,4]dioxin-6-yl)-2-methylbenzyl)oxy)-2-(((2-fluoroethyl)amino)methyl)phenoxy)methyl)picolinonitrile (**5i**):

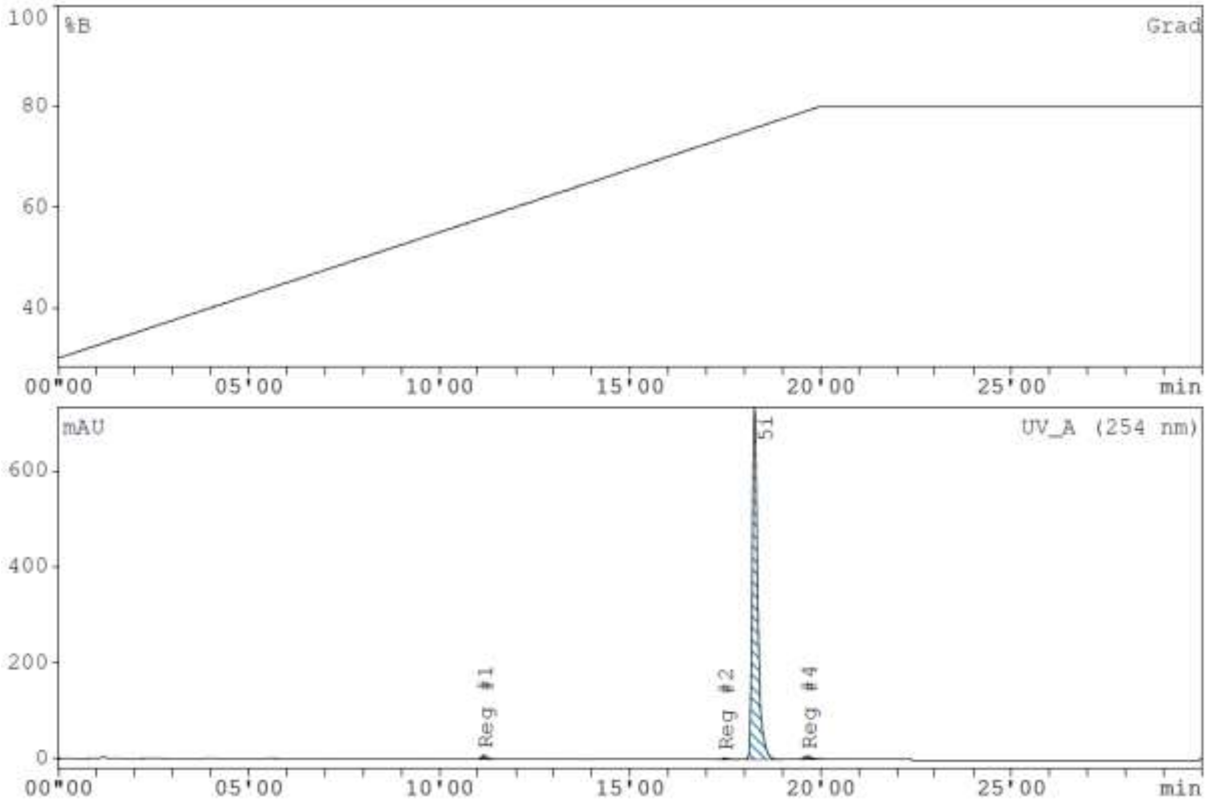

Integration UV_A (254 nm)

Substance	R/T s	Type	Area mAU*s	%Area %
Reg #1	11'10	BB	91,350	1,20
Reg #2	17'29	BB	35,811	0,47
5i	18'16	BB	7352,070	96,82
Reg #4	19'39	BB	114,220	1,50
Sum in ROI			7593,451	100,00

Figure S45: 2-Fluoroethyl (2-acetamidoethyl)(5-chloro-2-((3-cyanobenzyl)oxy)-4-((3-(2,3-dihydrobenzo[*b*][1,4]dioxin-6-yl)-2-methylbenzyl)oxy)benzyl)carbamate (**5j**):

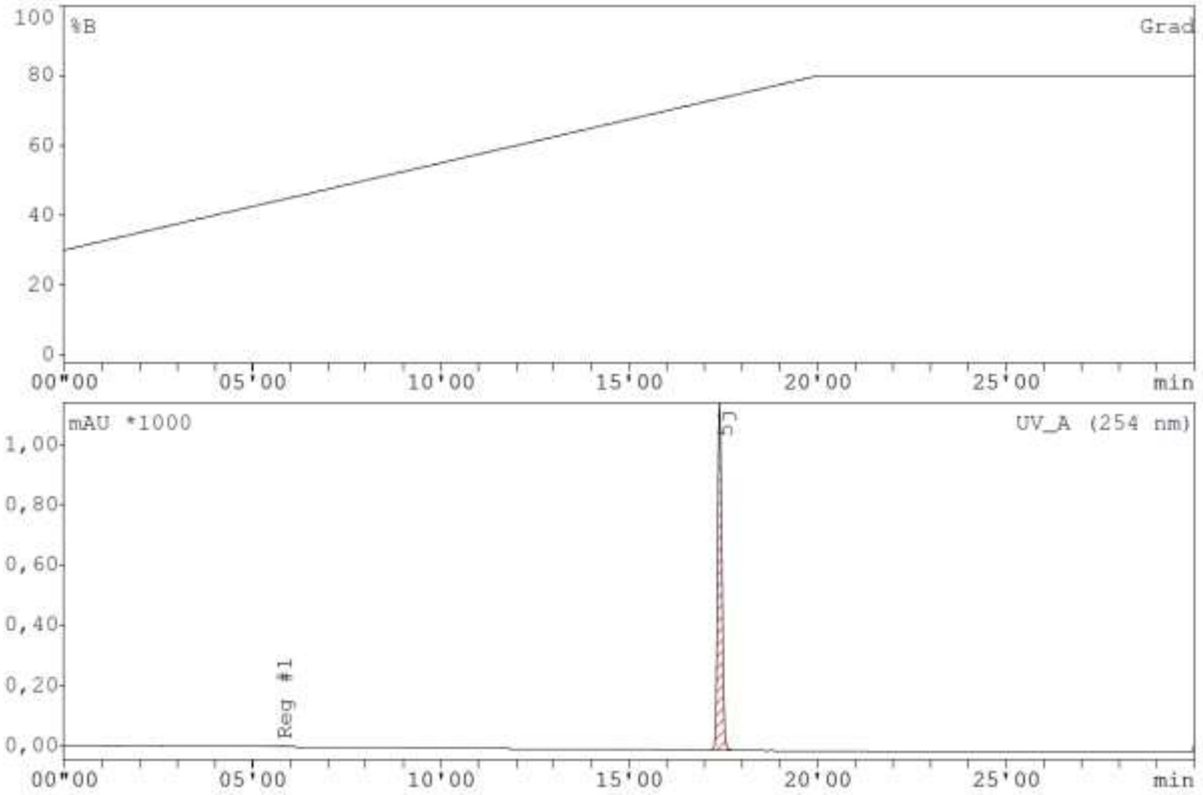

Integration UV_A (254 nm)

Substance	R/T s	Type	Area mAU*s	%Area %
Reg #1	05'48	BB	44,491	0,50
5j	17'24	BB	8852,504	99,50
Sum in ROI			8896,995	100,00

Nuclear magnetic resonance spectroscopy

Figure S46: (2-Methyl-[1,1'-biphenyl]-3-yl)methanol (**1a**):

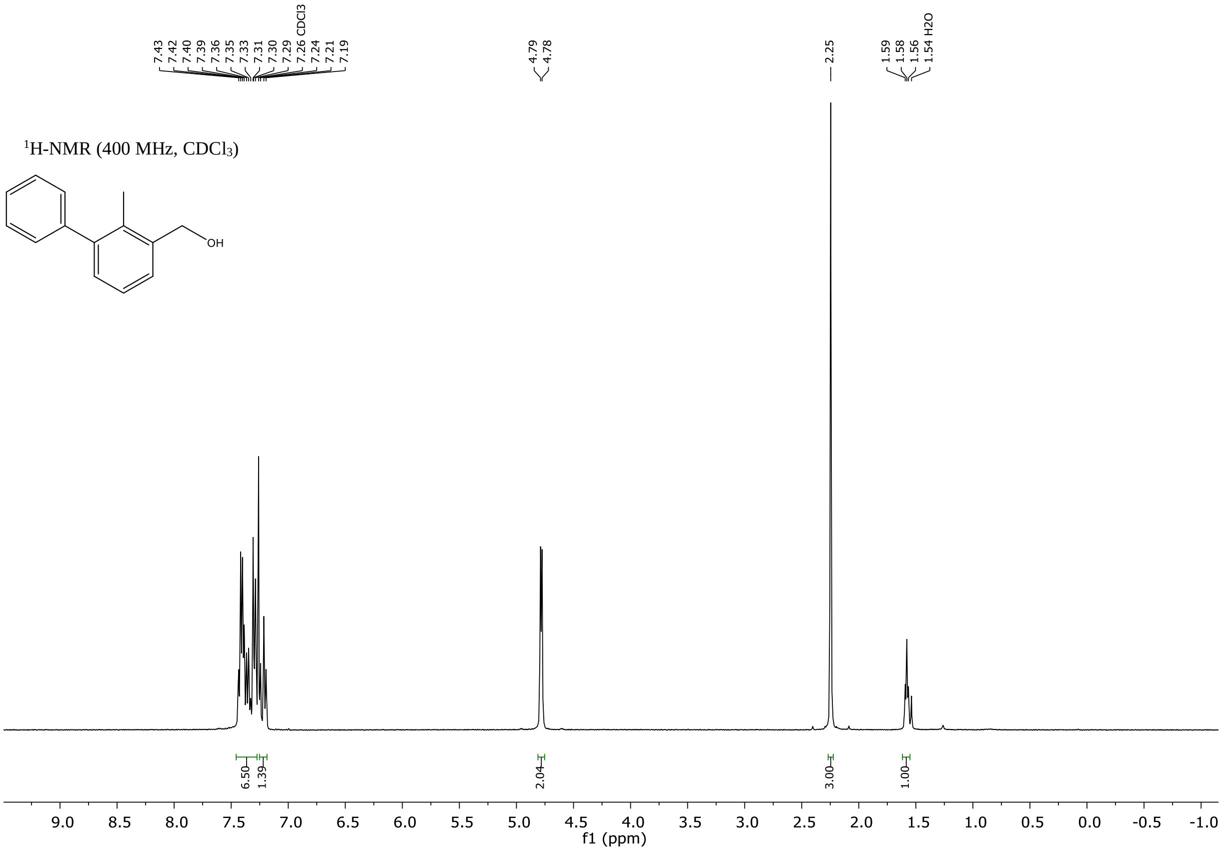

^{1}H-NMR (400 MHz, $CDCl_3$): δ 7.43-7.19 (m, 8H), 4.78 (d, *J* = 5.4 Hz, 2H), 2.25 (s, 3H), 1.59-1.56 (m, 1H).

Figure S47: (3-(2,3-Dihydrobenzo[*b*][1,4]dioxin-6-yl)-2-methylphenyl)methanol (**1b**):

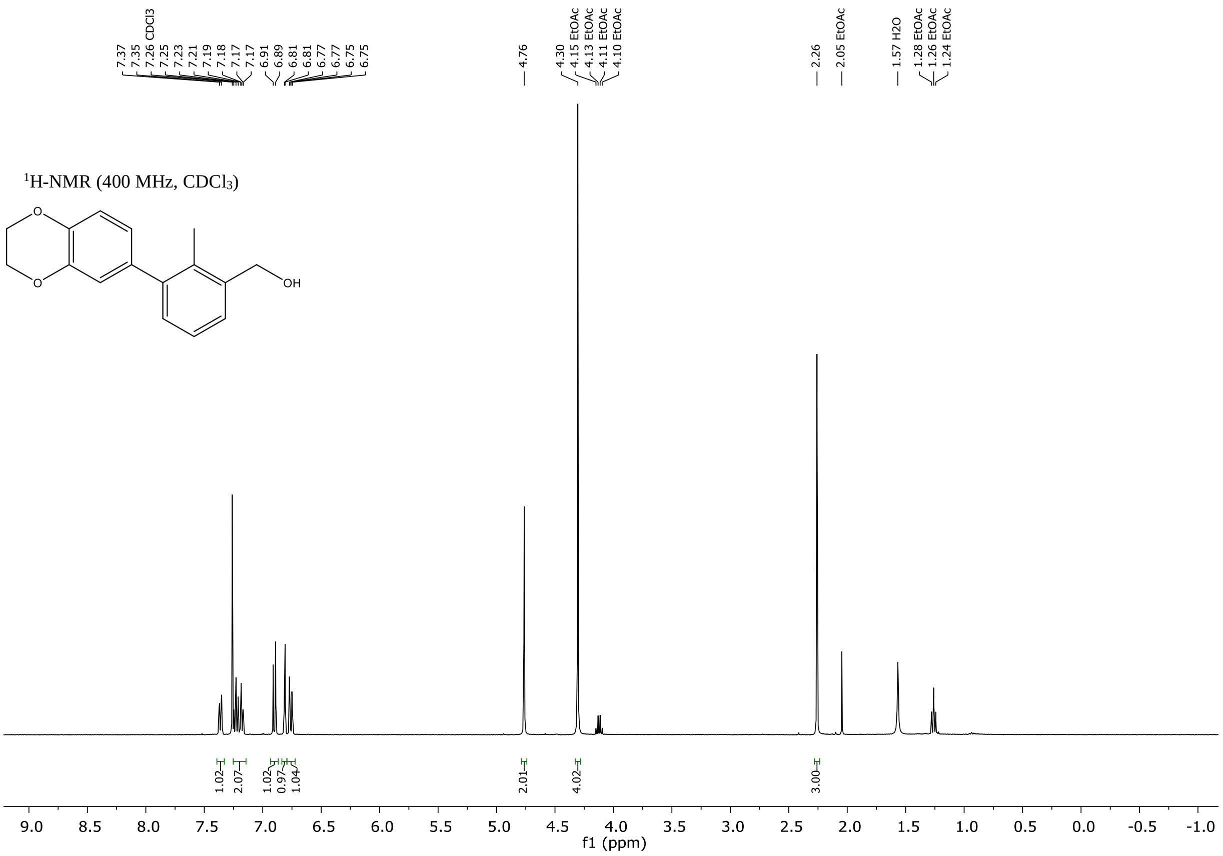

^{1}H-NMR (400 MHz, $CDCl_3$): δ 7.36 (d, *J* = 7.4 Hz, 1H), 7.23 (t, *J* = 7.5 Hz, 1H), 7.18 (d, *J* = 7.6 Hz, 1H), 6.90 (d, *J* = 8.2 Hz, 1H), 6.81 (d, *J* = 2 Hz), 6.76 (dd, *J* = 8.3 Hz, *J* = 2.1 Hz, 1H), 4.76 (s, 2H), 4.30 (s, 4H), 2.26 (s, 3H).

Figure S48: (2-Methyl-3-(1*H*-pyrrol-1-yl)phenyl)methanol (**1c**):

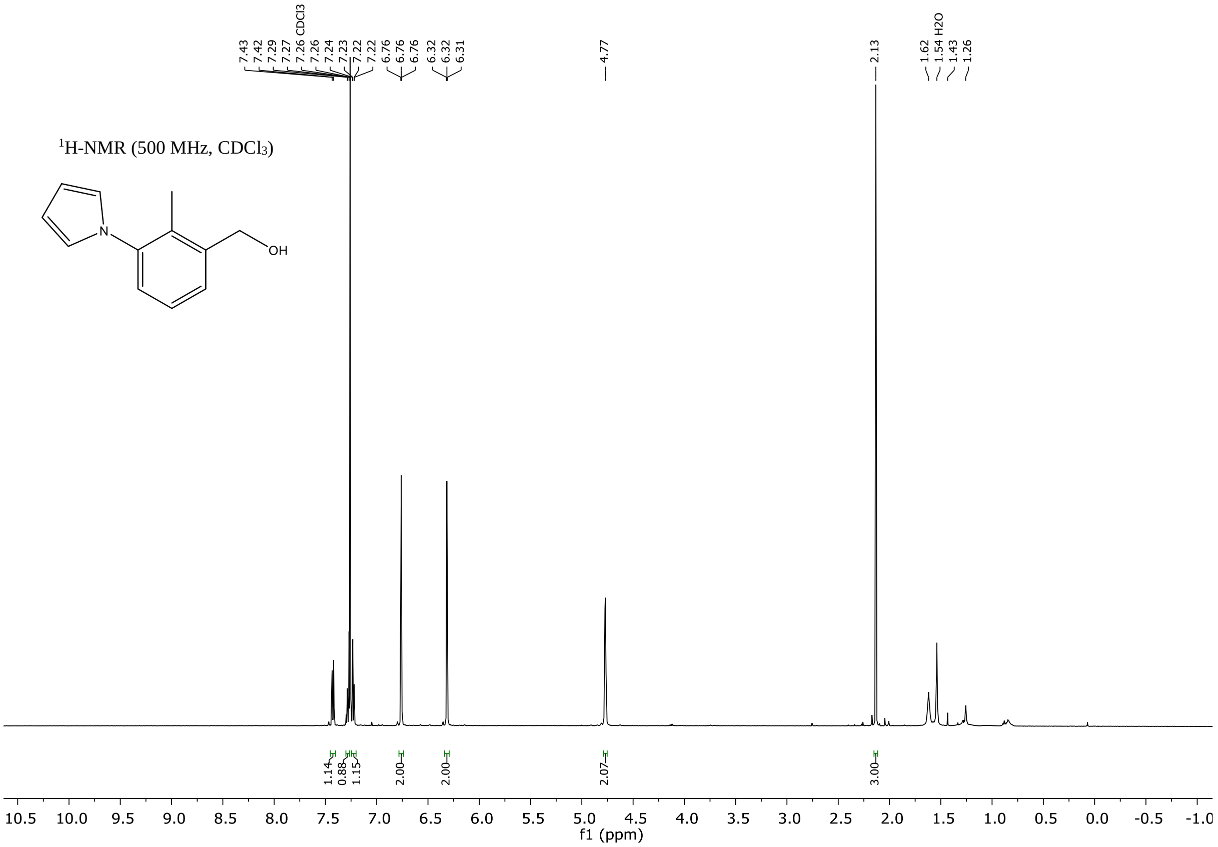

^{1}H-NMR (500 MHz, $CDCl_3$): δ 7.43 (d, *J* = 7.3 Hz, 1H), 7.29 – 7.22 (m, 2H), 6.76 (t, *J* = 2.1 Hz, 2H), 6.32 (t, *J* = 2.1 Hz, 2H), 4.77 (s, 2H), 2.13 (s, 3H).

Figure S49: (2-Methyl-3-(1*H*-pyrrol-1-yl)phenyl)methanol (**1c**):

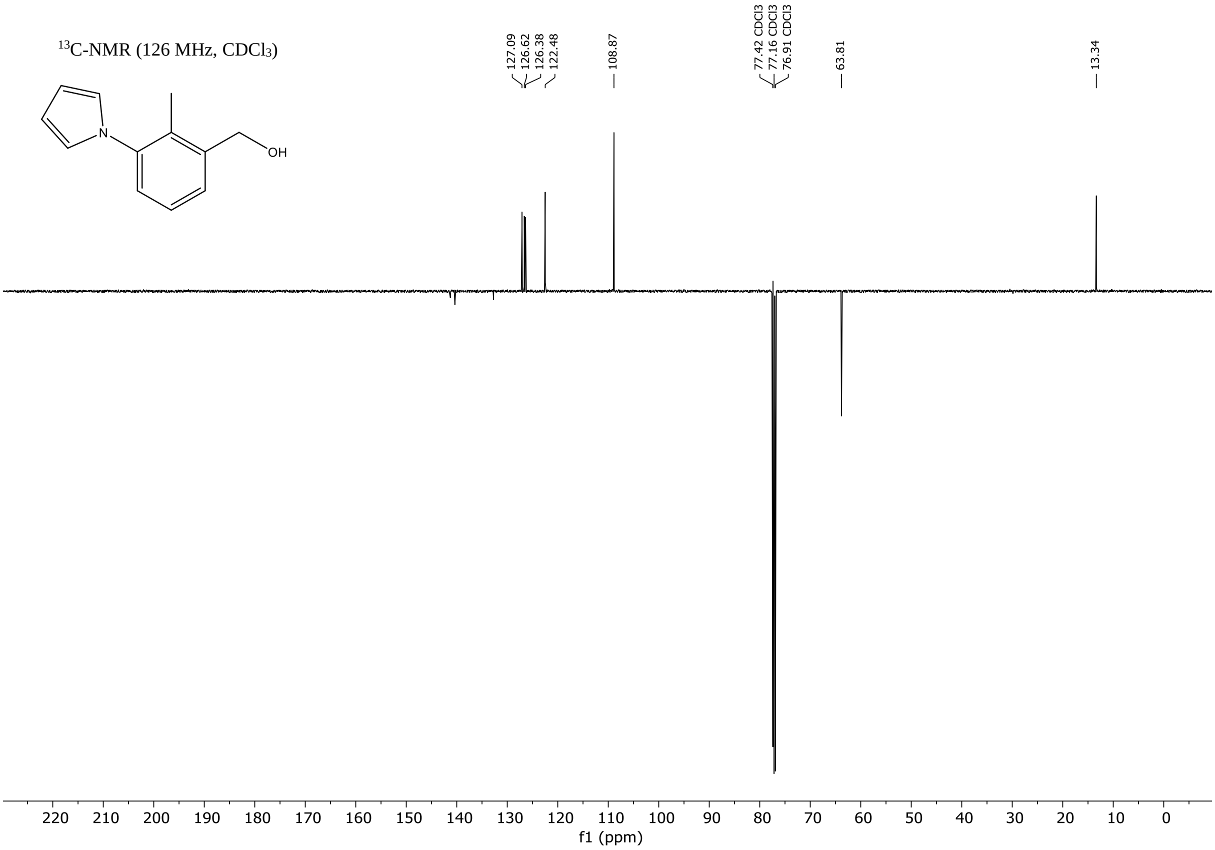

^{13}C-NMR (126 MHz, $CDCl_3$): δ 127.09, 126.62, 126.38, 122.48, 108.87, 63.81, 13.34.

Figure S50: 5-Chloro-6-((2-methyl-[1,1'-biphenyl]-3-yl)methoxy)nicotinaldehyde (**2a**):

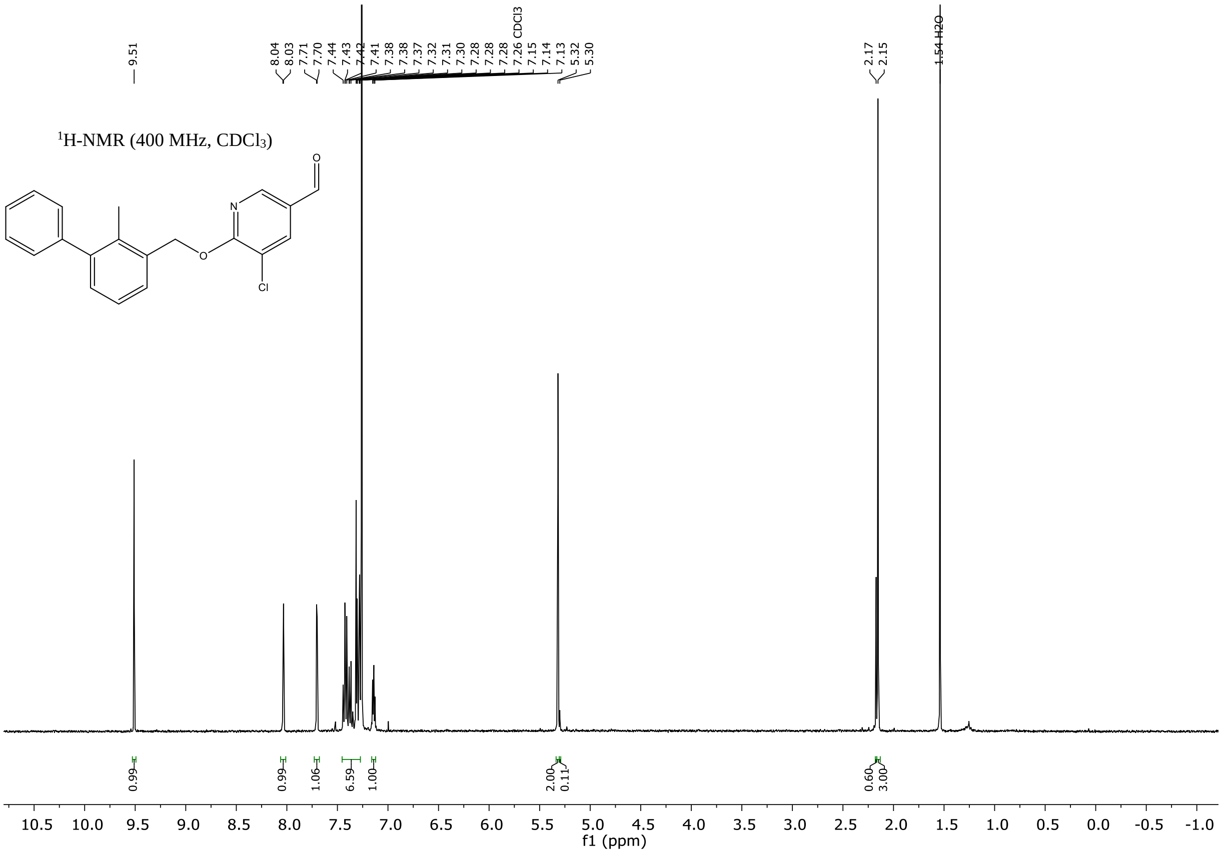

^{1}H-NMR (400 MHz, $CDCl_3$): δ 9.51 (s, 1H), 8.04 (d, *J* = 2.2 Hz, 1H), 7.70 (d, *J* = 2.2 Hz, 1H), 7.44-7.28 (m, 7H), 7.14 (m, 1H), 5.32 (s, 2H), 2.15 (s, 1H).

Figure S51: 5-Chloro-2-hydroxy-4-((2-methyl-[1,1'-biphenyl]-3-yl)methoxy)benzaldehyde (**2b**):

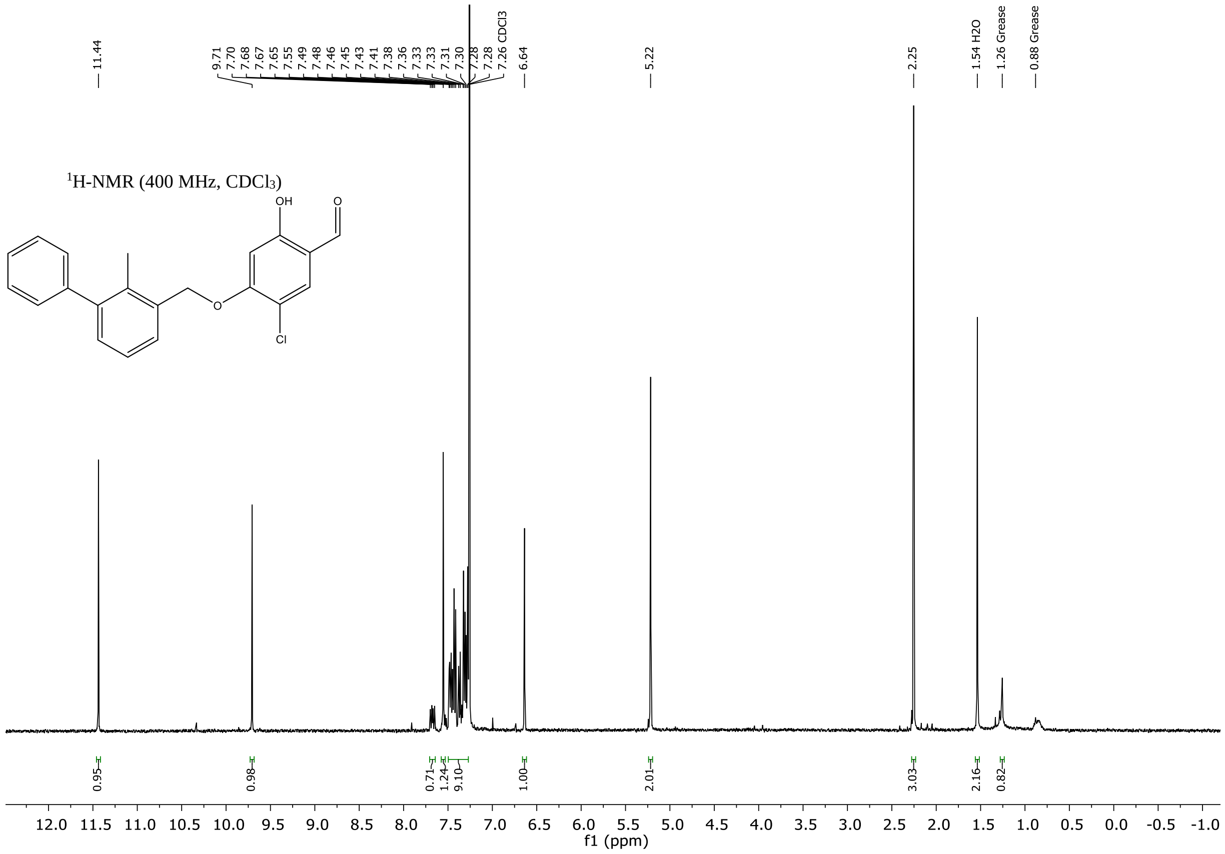

^{1}H-NMR (400 MHz, $CDCl_3$): δ 11.44 (s, 1H), 9.7 (s, 1H), 7.55 (s, 1H), 7.49-7.28 (m, 8H), 6.64 (s, 1H), 5.22 (s, 2H), 2.25 (s, 3H).

Figure S52: 5-Chloro-4-((3-(2,3-dihydrobenzo[*b*][1,4]dioxin-6-yl)-2-methylbenzyl)oxy)-2-hydroxybenzaldehyde (**2c**):

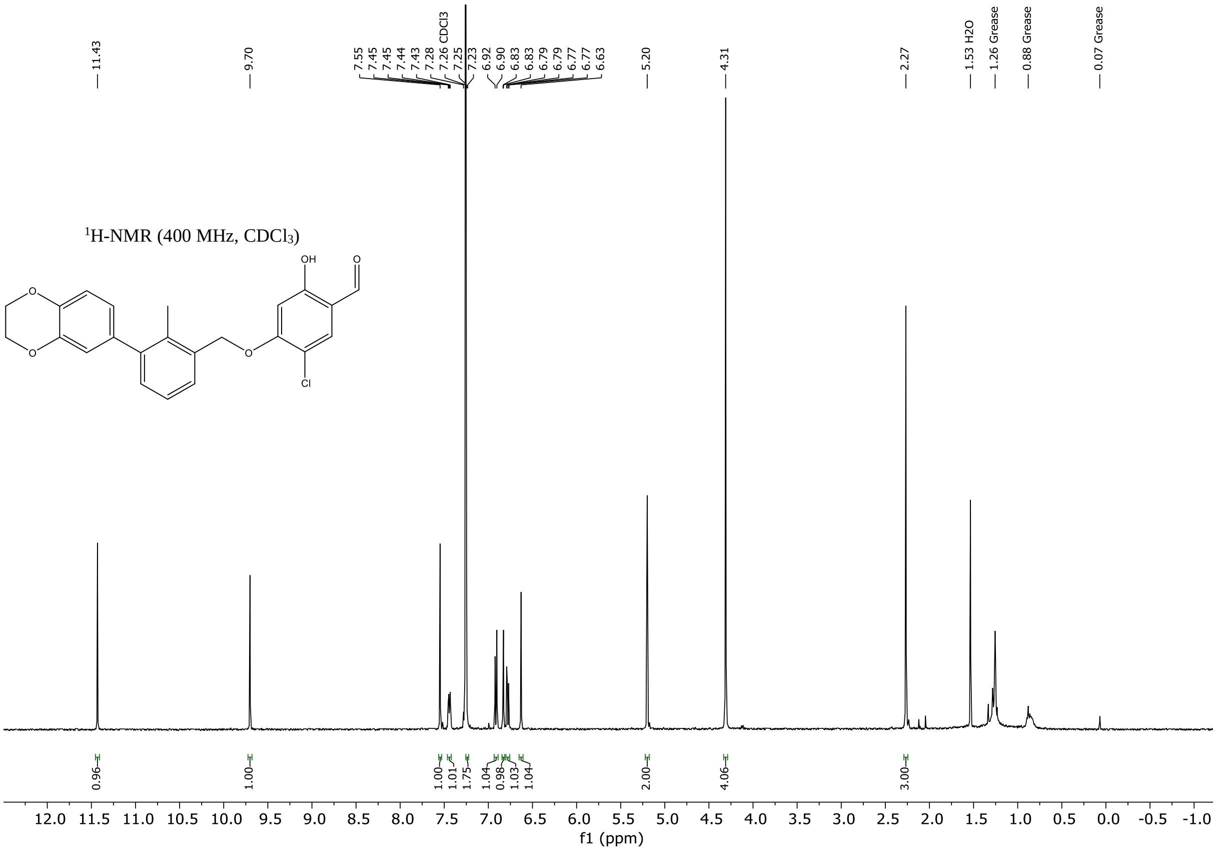

^{1}H-NMR (400 MHz, CDCl$_3$): δ 11.43 (s, 1H), 9.70 (s, 1H), 7.55 (s, 1H), 7.44 (dd, *J* = 6.2 Hz, *J* = 2.7 Hz, 1H), 7.28-7.23 (m, 2H), 6.91 (d, *J* = 8.2 Hz, 1H), 6.83 (d, *J* = 2.0 Hz), 6.78 (dd, *J* = 8.2 Hz, *J* = 2.0 Hz, 1H), 6.63 (s, 1H), 5.20 (s, 2H), 4.31 (s, 4H), 2.27 (s, 3H).

Figure S53: 5-Chloro-2-hydroxy-4-((2-methyl-3-(1*H*-pyrrol-1-yl)benzyl)oxy)benzaldehyde (**2d**):

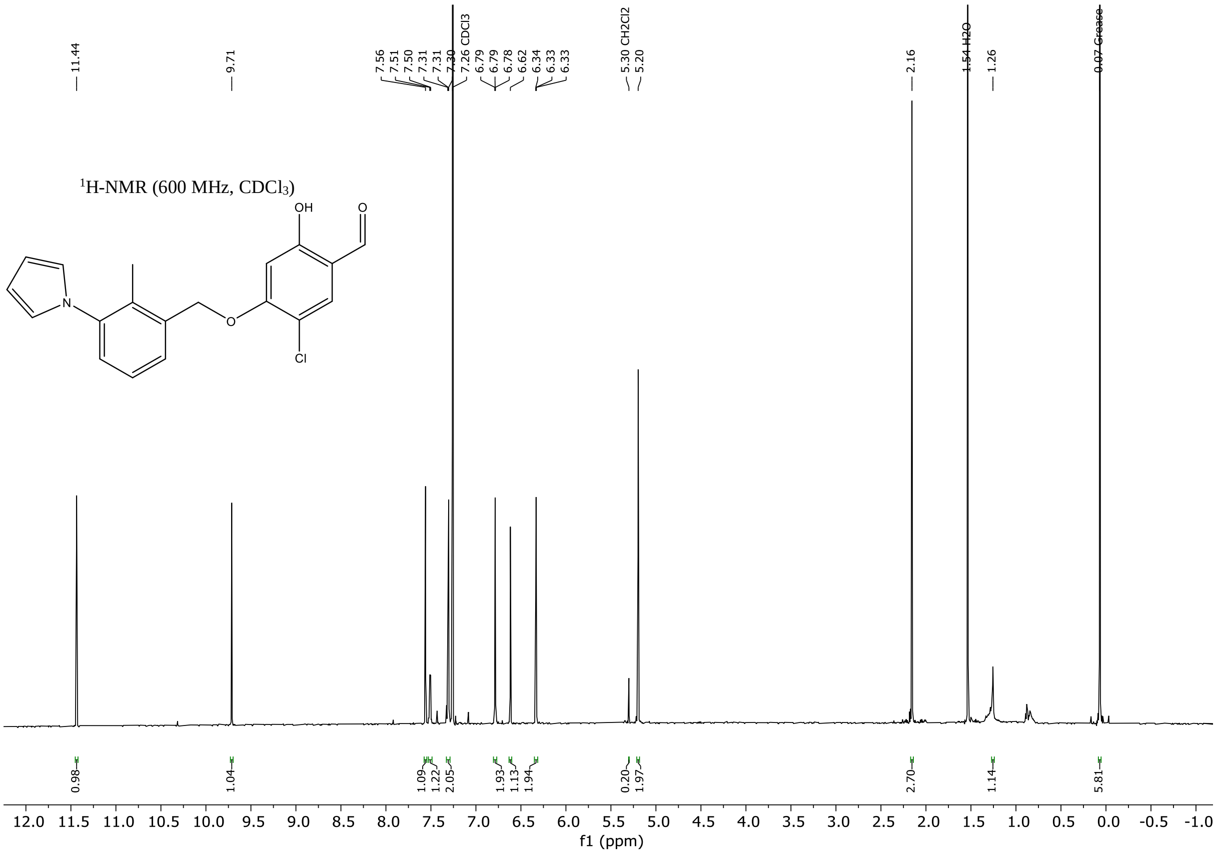

^{1}H-NMR (600 MHz, $CDCl_3$): δ 11.44 (s, 1H), 9.71 (s, 1H), 7.56 (s, 1H), 7.51 (m, 1H), 7.31 (m, 2H), 6.79 (t, *J* = 2.1 Hz, 2H), 6.62 (s, 1H), 6.33 (t, *J* = 2.1 Hz, 2H), 5.20 (s, 2H), 2.16 (s, 3H).

Figure S54: 3-((4-Chloro-2-formyl-5-((2-methyl-[1,1'-biphenyl]-3-yl)methoxy)phenoxy)methyl)benzonitrile (**3a**):

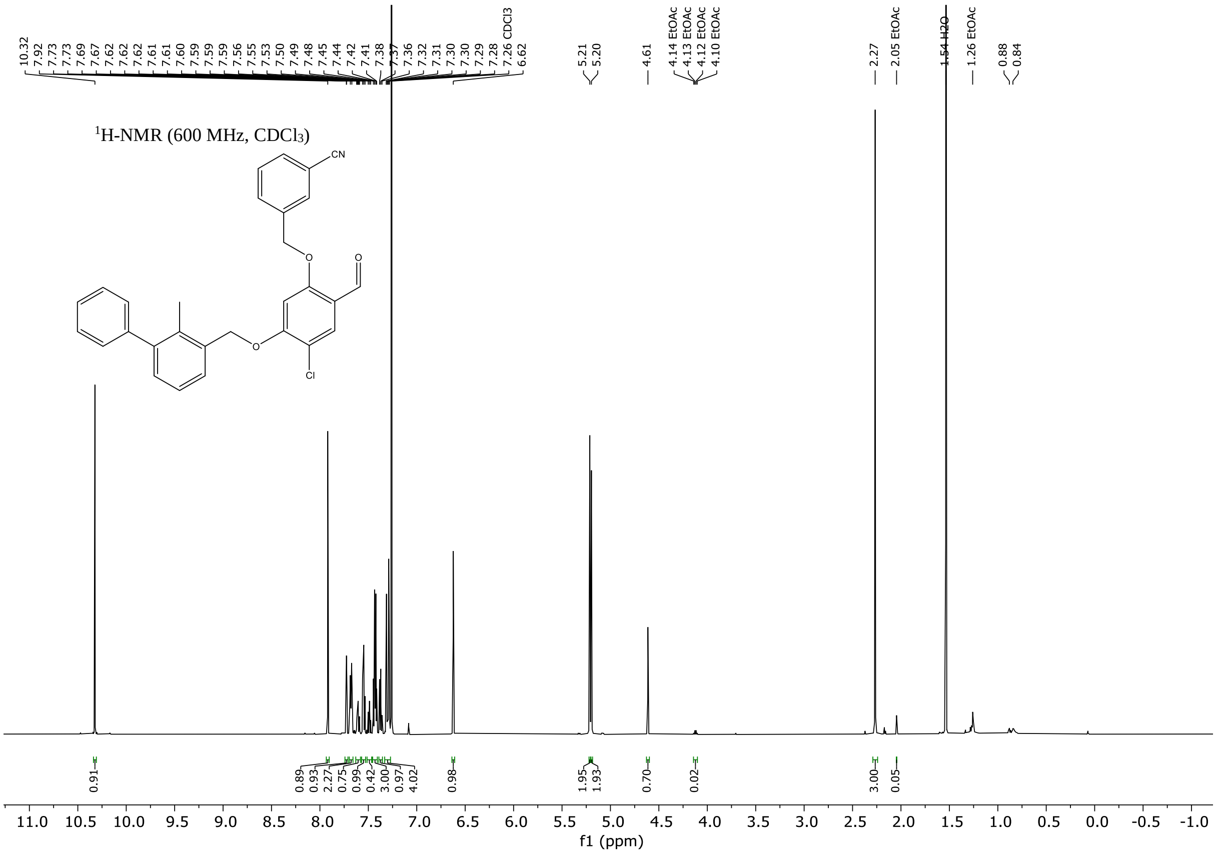

^{1}H-NMR (600 MHz, $CDCl_3$): δ 10.32 (s, 1H), 7.92 (s, 1H), 7.73 (s, 1H), 7.69-7.67 (m, 2H), 7.55 (t, *J* = 7.8 Hz, 1H), 7.45-7.31 (m, 3H), 7.38-7.36 (m, 1H), 7.32 – 7.28 (m, 4H), 6.62 (s, 1H), 5.21 (s, 2H), 5.20 (s, 2H), 2.27 (s, 3H).

Figure S55: 3-((4-Chloro-2-formyl-5-((2-methyl-[1,1'-biphenyl]-3-yl)methoxy)phenoxy)methyl)benzonitrile (**3a**):

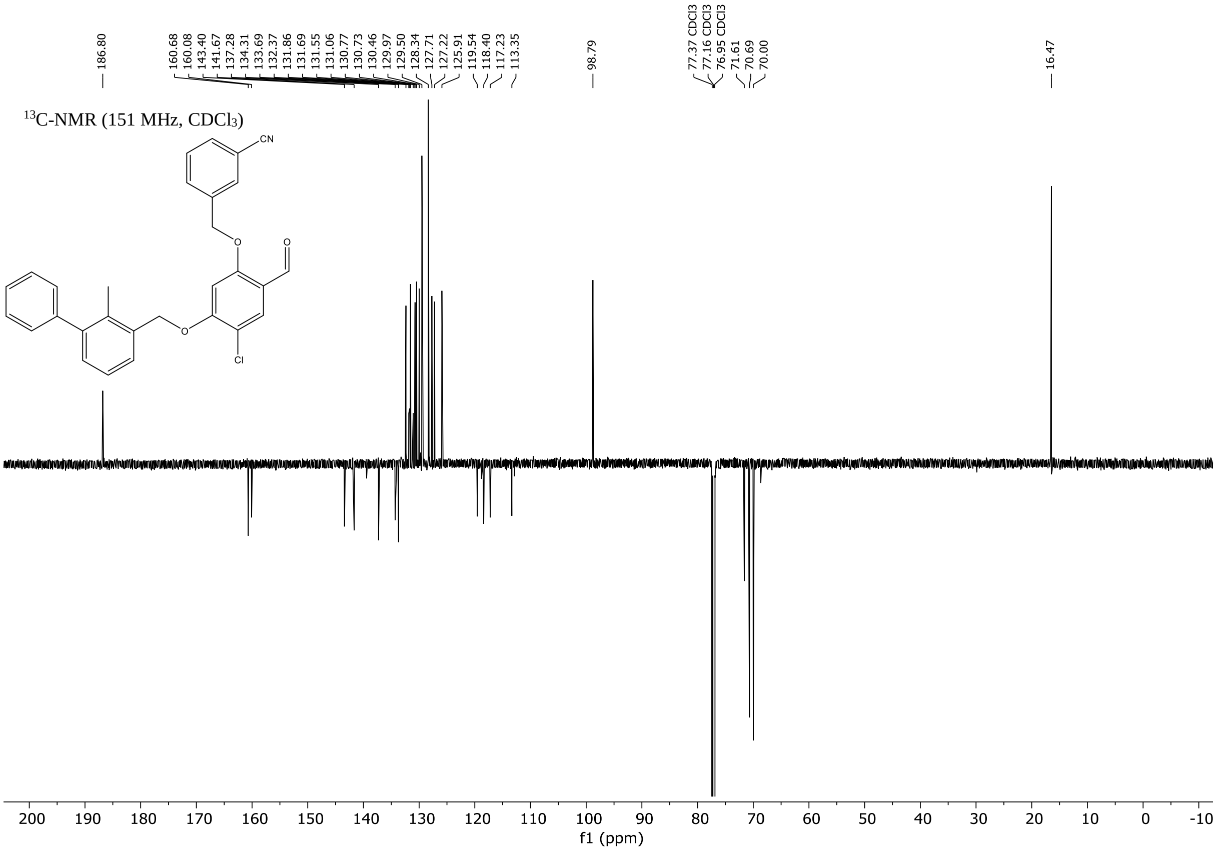

^{13}C-NMR (151 MHz, $CDCl_3$): δ 186.80, 160.68, 160.08, 143.40, 141.67, 137.28, 134.31, 133.69, 132.37, 131.55, 130.77, 130.73, 130.46, 129.97, 129.50, 128.34, 127.71, 127.22, 125.91, 119.54, 118.40, 117.23, 113.35, 98.79, 70.69, 70.00, 16.47.

Figure S56: 4-((4-Chloro-5-((3-(2,3-dihydrobenzo[*b*][1,4]dioxin-6-yl)-2-methylbenzyl)oxy)-2-formylphenoxy)methyl)picolinonitrile (**3b**):

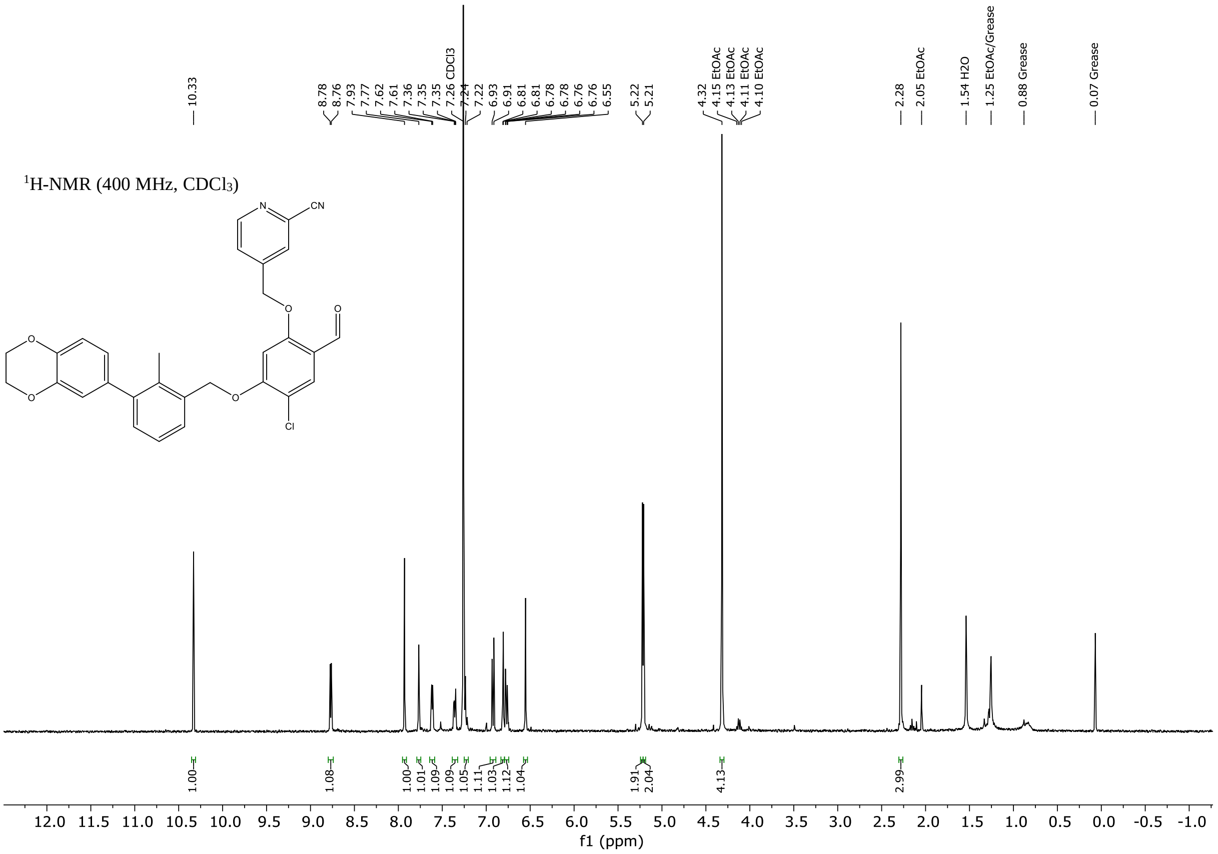

^{1}H-NMR (400 MHz, $CDCl_3$): δ 10.33 (s, 1H), 8.77 (d, *J* = 5.0 Hz, 1H), 7.93 (s, 1H), 7.77 (s, 1H), 7.61 (d, *J* = 4.7 Hz, 1H), 7.36 (dd, *J* = 6.5 Hz, *J* = 2.2 Hz, 1H), 7.26 – 7.22 (m, 2H), 6.92 (d, *J* = 8.2 Hz, 1H), 6.81 (d, *J* = 2.0 Hz, 1H), 6.77 (dd, *J* = 8.2 Hz, *J* = 2.0 Hz, 1H), 6.55 (s, 1H), 5.22 (s, 2H), 5.21 (s, 2H), 4.32 (s, 4H), 2.28 (s, 3H).

Figure S57: 3-((4-Chloro-5-((3-(2,3-dihydrobenzo[*b*][1,4]dioxin-6-yl)-2-methylbenzyl)oxy)-2-formylphenoxy)methyl)benzonitrile (**3c**):

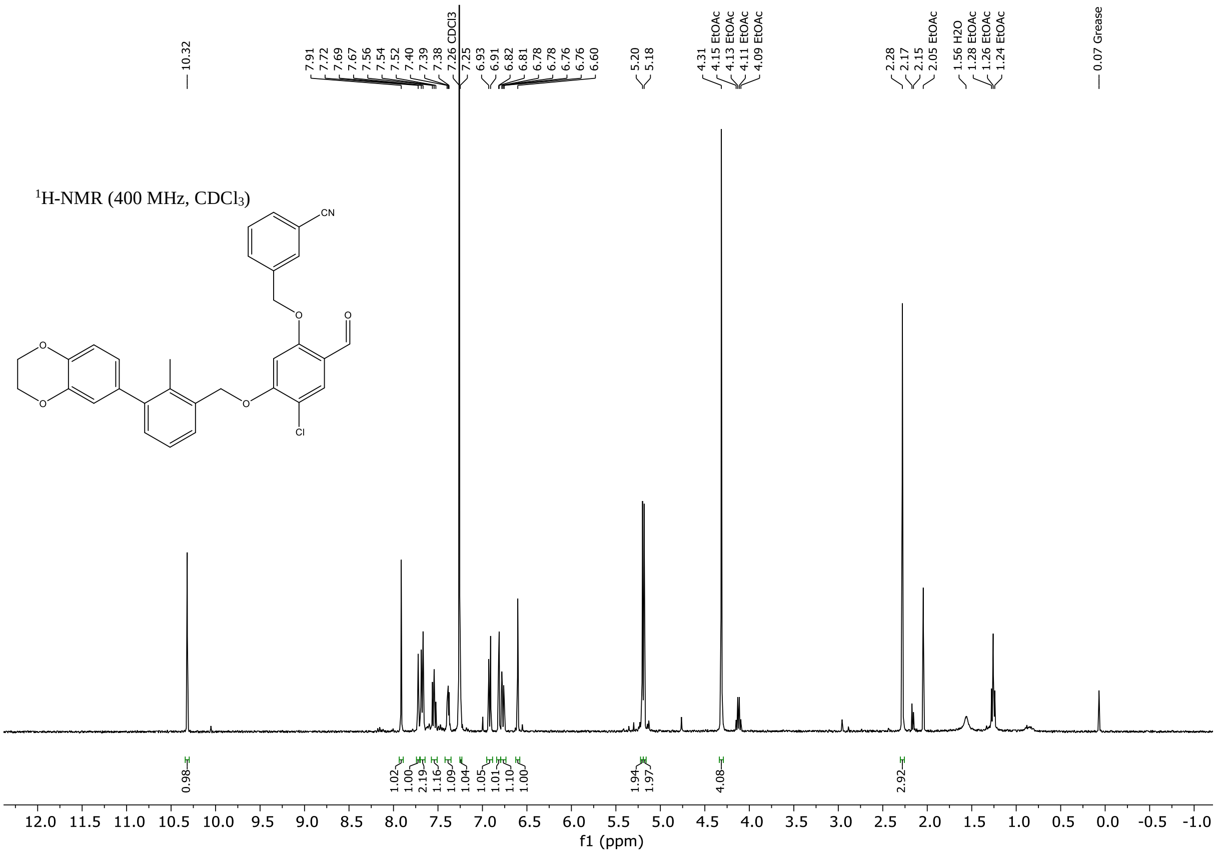

^{1}H-NMR (400 MHz, CDCl$_3$): δ 10.32 (s, 1H), 7.91 (s, 1H), 7.72 (s, 1H), 7.68 (d, *J* = 8.0 Hz, 2H), 7.54 (t, *J* = 7.8 Hz, 1H), 7.39 (m, 1H), 7.26 – 7.25 (m, 2H), 6.92 (d, *J* = 8.2 Hz, 1H), 6.81 (d, *J* = 2.0 Hz, 1H), 6.77 (dd, *J* = 8.2 Hz, *J* = 2.0 Hz, 1H), 6.60 (s, 1H), 5.20 (s, 2H), 5.18 (s, 2H), 4.31 (s, 4H), 2.28 (s, 3H).

Figure S58: 5-Chloro-4-((3-(2,3-dihydrobenzo[*b*][1,4]dioxin-6-yl)-2-methylbenzyl)oxy)-2-(oxazol-4-ylmethoxy)benzaldehyde (**3d**):

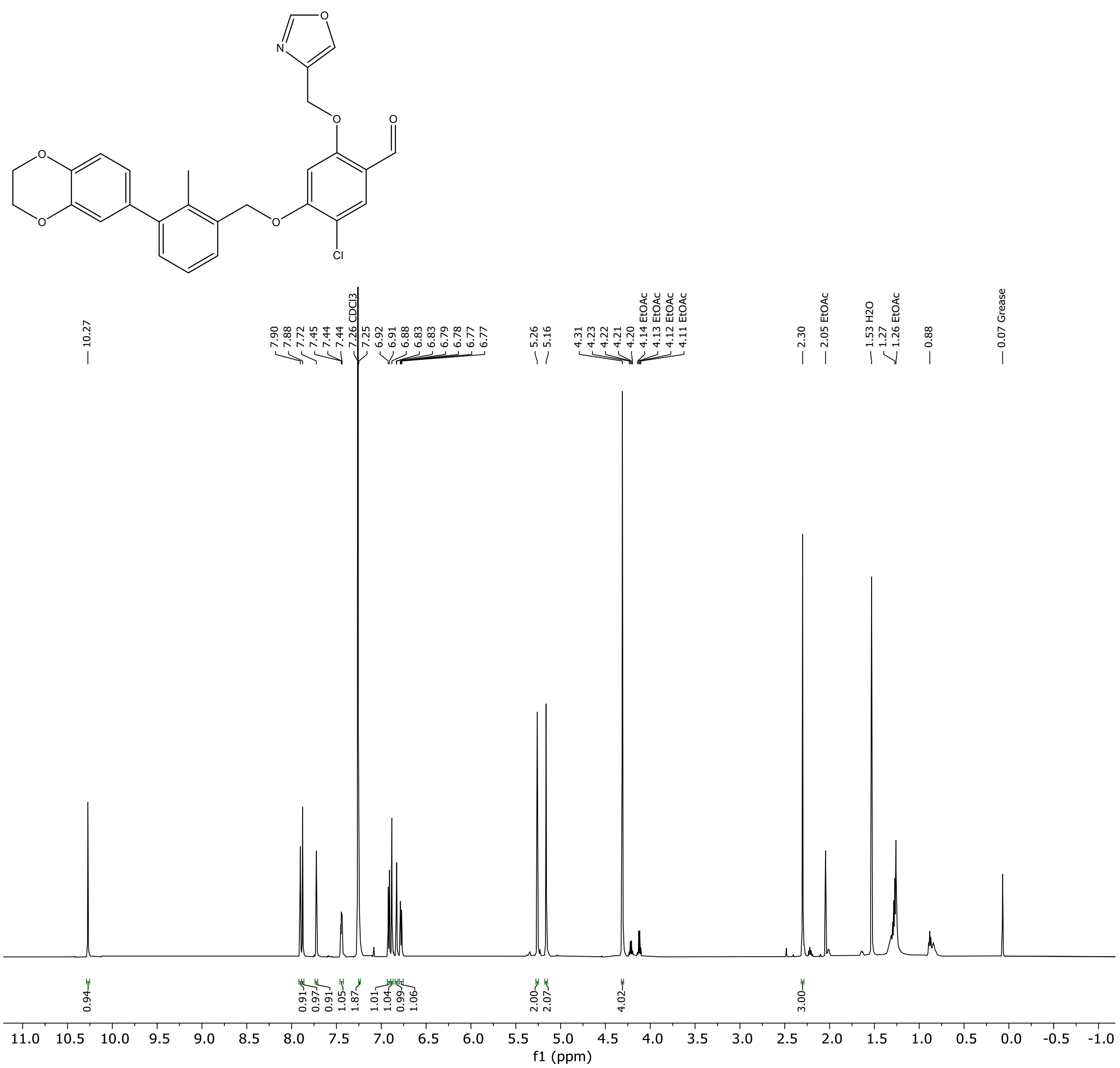

^{1}H-NMR (600 MHz, $CDCl_3$): δ 10.27 (s, 1H), 7.90 (s, 1H), 7.88 (s, 1H), 7.45 – 7.44 (m, 1H), 7.26 – 7.25 (m, 2H), 6.91 (d, *J* = 8.2 Hz, 1H), 6.88 (s, 1H), 6.83 (d, *J* = 2.1 Hz, 1H), 6.77 (dd, *J* = 8.2 Hz, *J* = 2.1 Hz, 1H), 5.26 (s, 2H), 5.16 (s, 2H), 4.31 (s, 4H), 2.30 (s, 3H).

Figure S59: 5-Chloro-4-((3-(2,3-dihydrobenzo[*b*][1,4]dioxin-6-yl)-2-methylbenzyl)oxy)-2-(oxazol-4-ylmethoxy)benzaldehyde (**3d**):

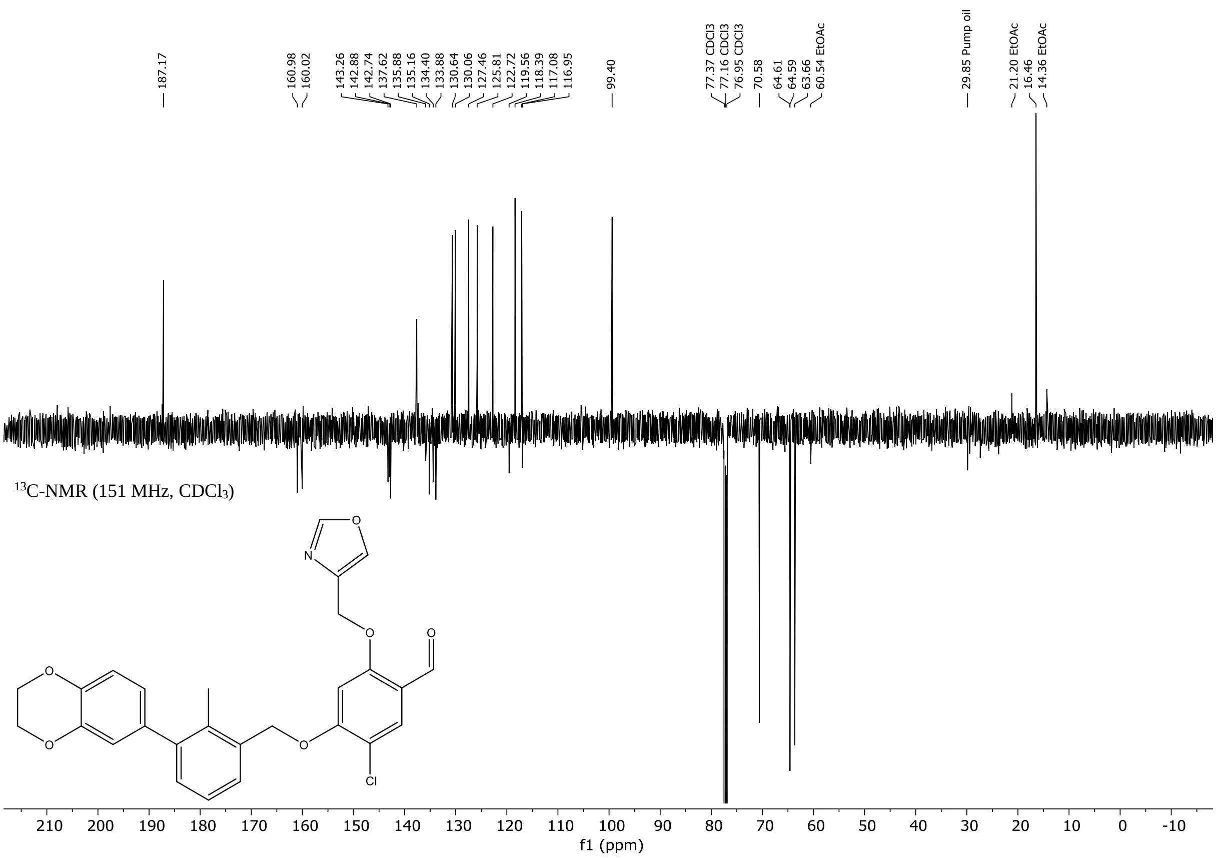

^{13}C-NMR (151 MHz, $CDCl_3$): δ 187.17, 160.98, 160.02, 143.26, 142.88, 142.74, 137.62, 135.88, 135.16, 134.40, 133.88, 130.64, 130.06, 127.46, 125.81, 122.72, 119.56, 118.39, 117.08, 116.95, 99.40, 70.58, 64.61, 64.59, 63.66, 16.46.

Figure S60: 4-((4-Chloro-2-formyl-5-((2-methyl-3-(1*H*-pyrrol-1-yl)benzyl)oxy)phenoxy)methyl)picolinonitrile (**3e**):

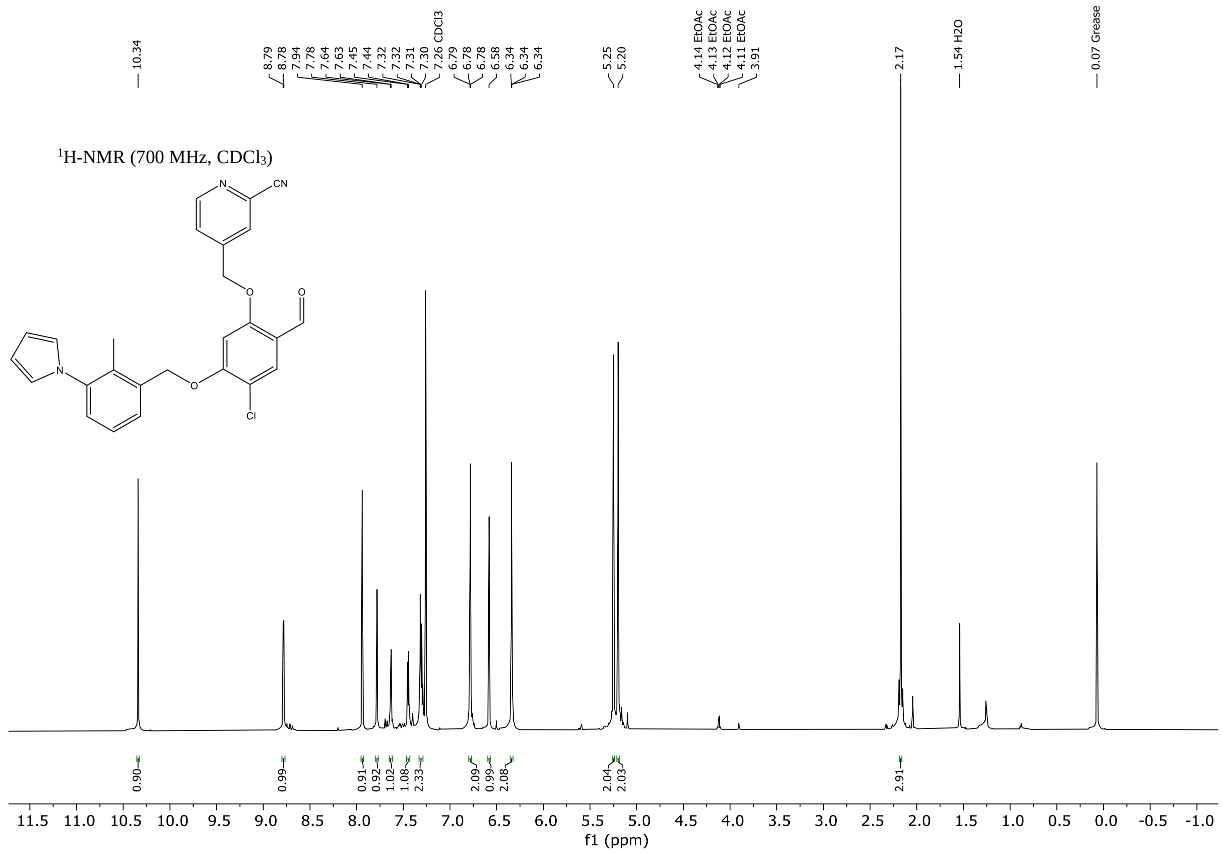

^{1}H-NMR (700 MHz, $CDCl_3$): δ 10.34 (s, 1H), 8.78 (d, *J* = 4.9 Hz, 1H), 7.94 (s, 1H), 7.78 (s, 1H), 7.64 (d, *J* = 4.9 Hz, 2H), 7.45 (d, *J* = 7.1 Hz, 1H), 7.32-7.30 (m, 2H), 6.78 (t, *J* = 2.0 Hz, 2H), 6.58 (s, 1H), 6.34 (t, *J* = 2.0 Hz, 2H), 5.25 (s, 2H), 5.20 (s, 2H), 2.17 (s, 3H).

Figure S61: *N*-(2-(((5-chloro-6-((2-methyl-[1,1'-biphenyl]-3-yl)methoxy)pyridin-3-yl)methyl)amino)ethyl)acetamide (**4a**):

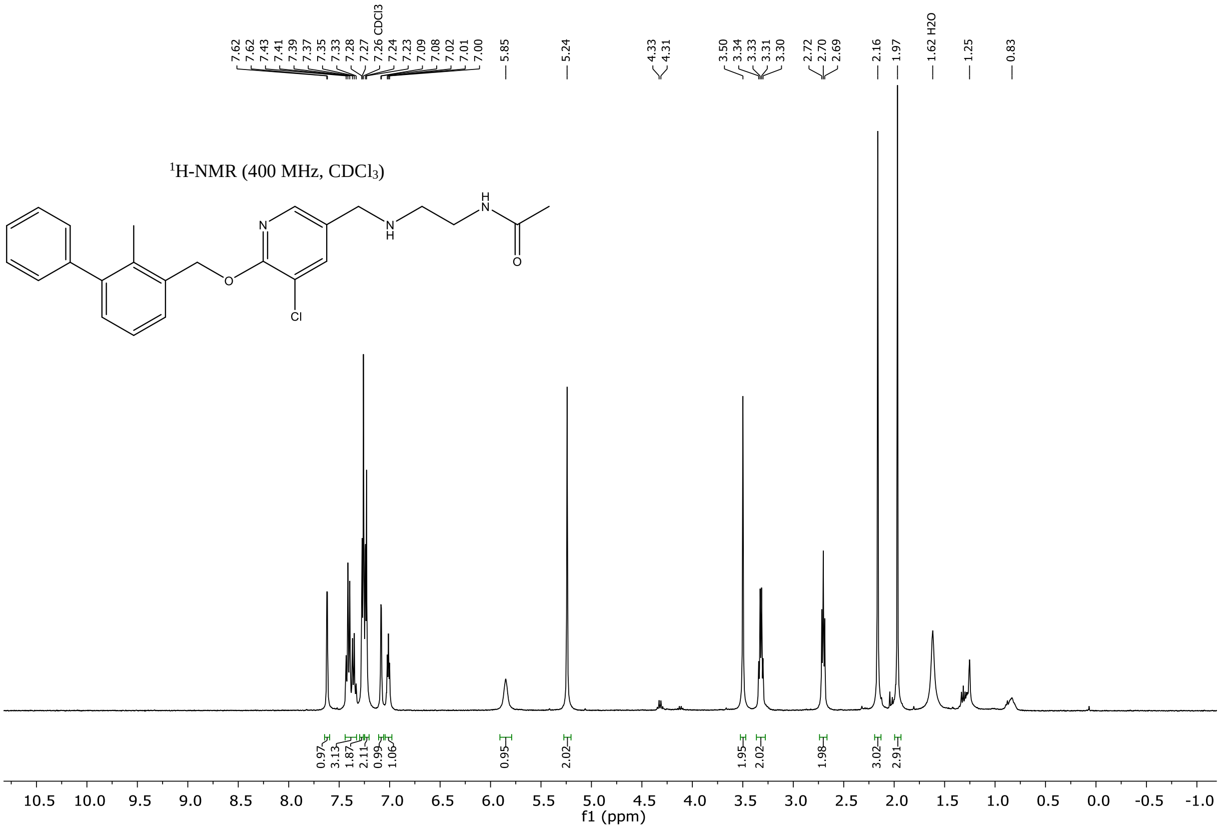

^{1}H-NMR (400 MHz, $CDCl_3$): δ 7.62 (d, *J* = 2.0 Hz, 1H), 7.43-7.23 (m, 7H), 7.08 (d, *J* = 1.8 Hz, 1H), 7.01 (t, *J* = 4.8 Hz, 1H), 5.85 (br s, 1H), 5.24 (s, 2H), 3.50 (s, 2H), 3.32 (q, *J* = 5.8 Hz, 2H), 2.70 (t, *J* = 5.9 Hz, 2H), 2.16 (s, 3H), 1.97 (s, 3H).

Figure S62: *N*-(2-(((5-chloro-6-((2-methyl-[1,1'-biphenyl]-3-yl)methoxy)pyridin-3-yl)methyl)amino)ethyl)acetamide (**4a**):

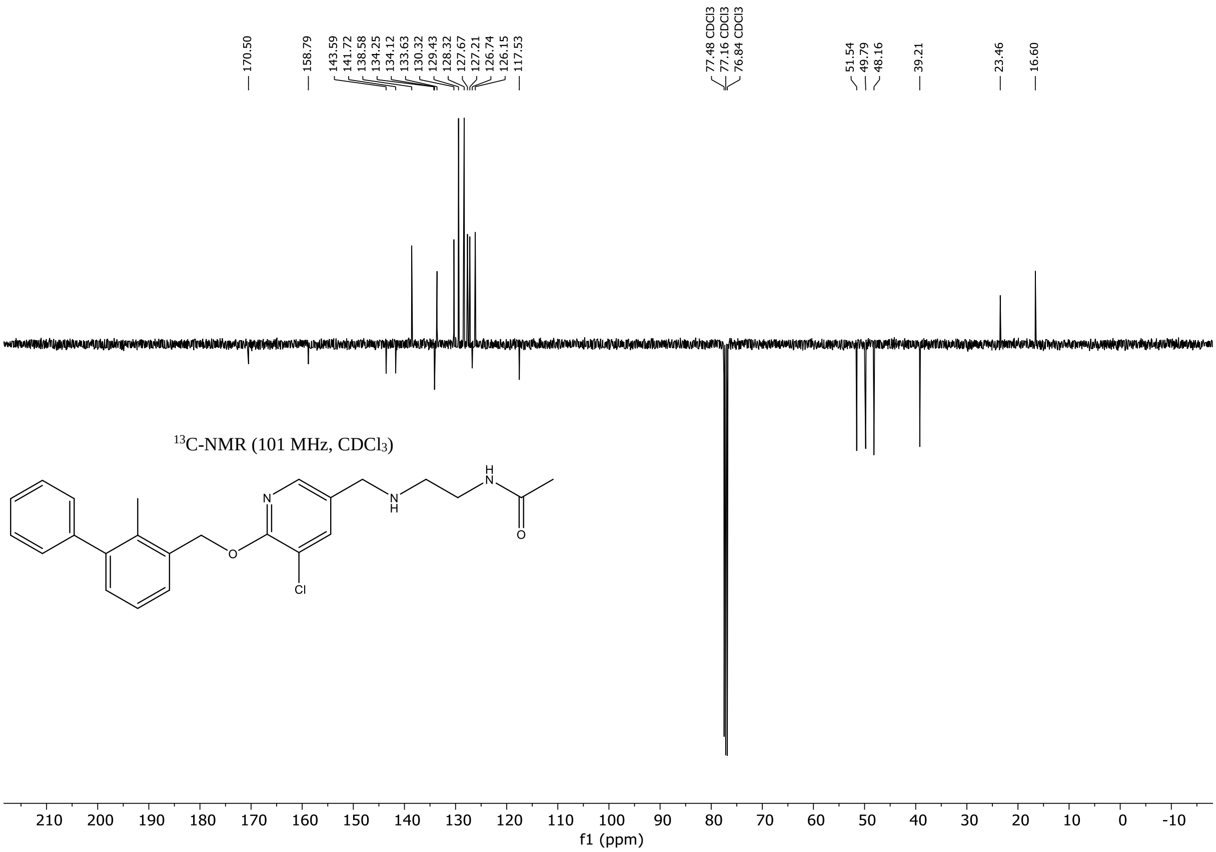

^{13}C-NMR (101 MHz, $CDCl_3$): δ 170.50, 158.79, 143.59, 141.72, 138.58, 134.25, 134.12, 133.63, 130.32, 129.43, 128.32, 127.67, 127.21, 126.74, 126.15, 117.53, 51.54, 49.79, 48.16, 39.21, 23.46, 16.60.

Figure S63: (5-Chloro-2-((3-cyanobenzyl)oxy)-4-((2-methyl-[1,1'-biphenyl]-3-yl)methoxy)benzyl)-*D*-serine (**4b**):

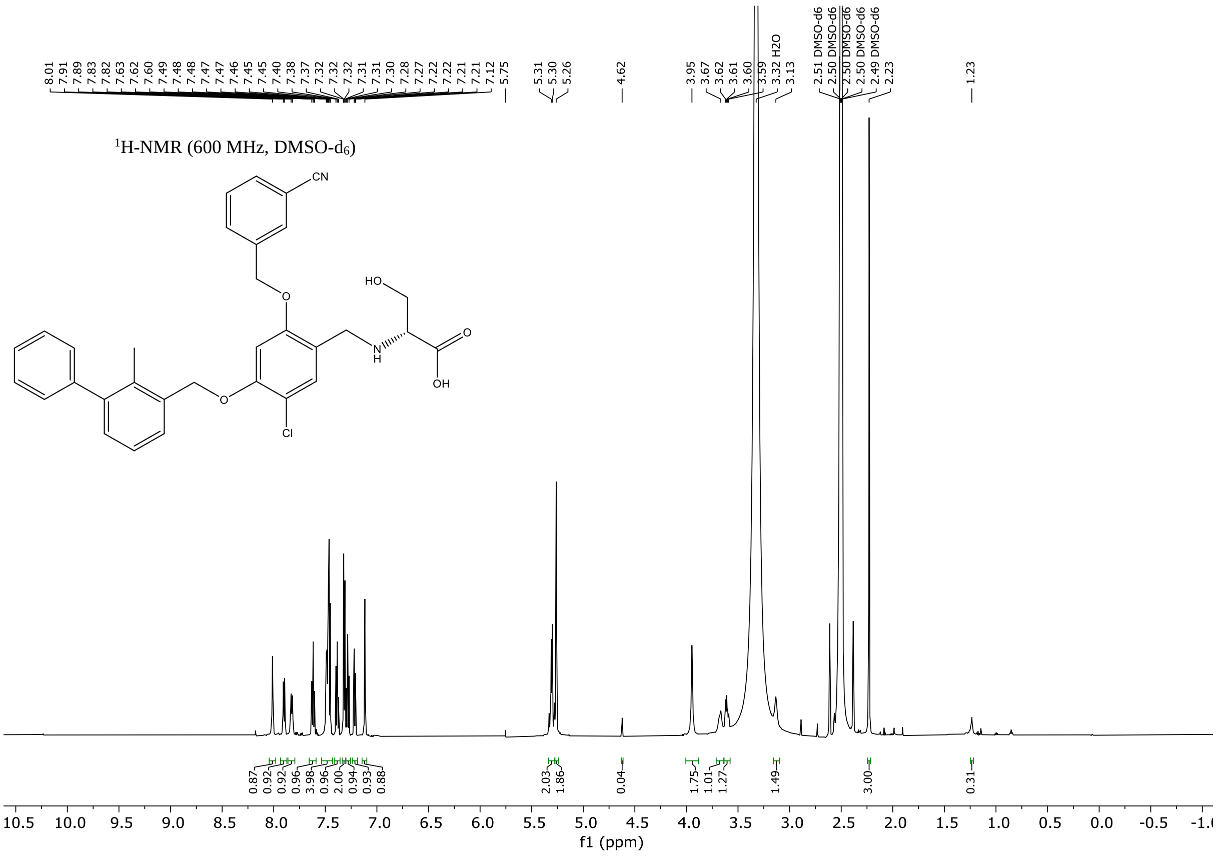

^{1}H-NMR (600 MHz, DMSO-d$_6$): δ 8.01 (s, 1H), 7.90 (d, *J* = 7.8 Hz, 1H), 7.89 (d, *J* = 5.1 Hz, 1H), 7.83 (s, *J* = 7.8 Hz, 1H), 7.62 (t, *J* = 7.8 Hz, 1H), 7.49 – 7.45 (m, 4H), 7.38 (tt, *J* = 7.5 Hz, *J* = 1.2 Hz, 1H), 7.32 – 7.30 (m, 2H), 7.28 (t, *J* = 7.5 Hz, 1H), 7.21 (dd, *J* = 7.6 Hz, *J* = 1.2 Hz, 1H), 7.12 (s, 1H), 5.30 (m, 2H), 5.26 (s, 2H), 3.95 (s, 2H), 3.67 – 3.59 (m, 2H), 3.13 (m, 1H), 2.23 (s, 3H).

Figure S64: (5-Chloro-2-((3-cyanobenzyl)oxy)-4-((2-methyl-[1,1'-biphenyl]-3-yl)methoxy)benzyl)-*D*-serine (**4b**):

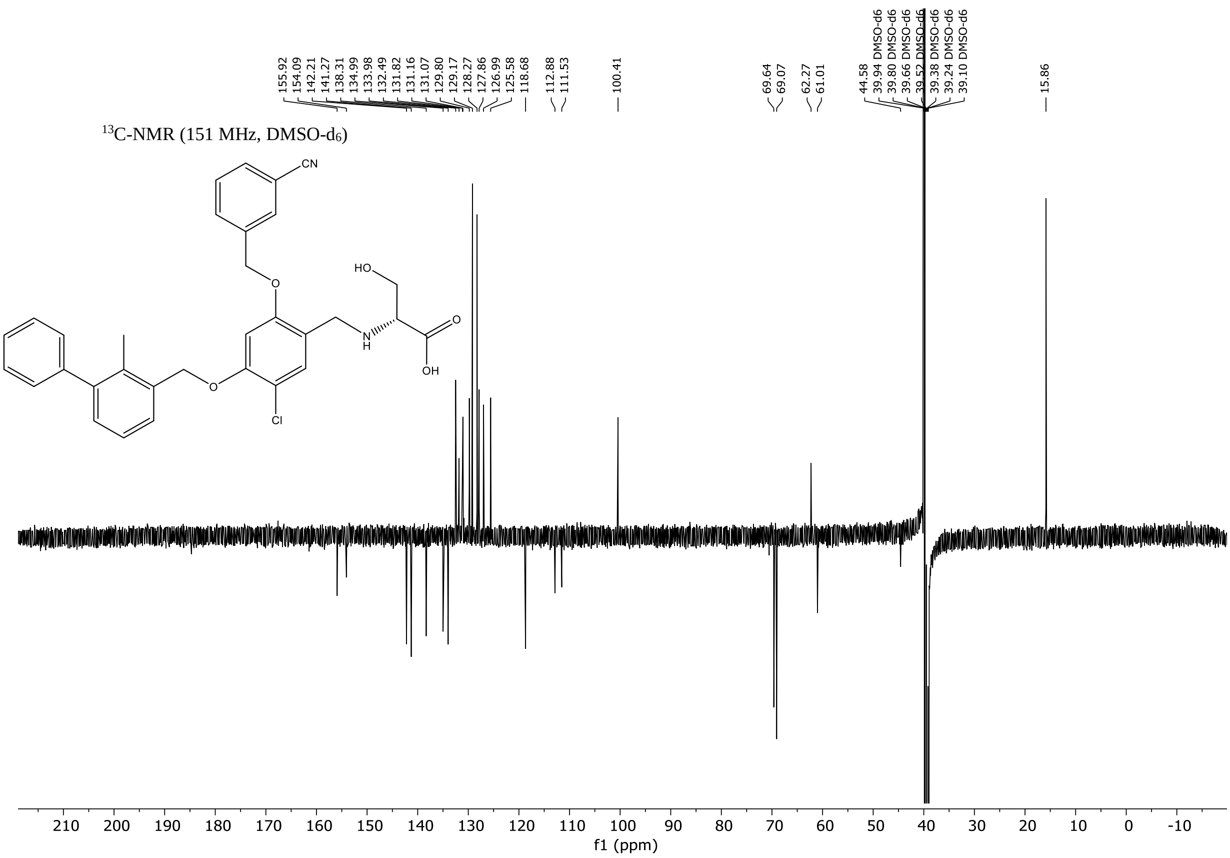

^{13}C-NMR (151 MHz, DMSO-d_6): δ 155.92, 154.09, 142.21, 141.27, 138.31, 134.99, 133.98, 132.49, 131.82, 131.16, 131.07, 129.80, 129.17, 128.27, 127.86, 126.99, 125.58, 118.68, 112.88, 111.53, 100.41, 69.64, 69.07, 62.27, 61.01, 44.58, 15.86.

Figure S65: (5-Chloro-2-((2-cyanopyridin-4-yl)methoxy)-4-((3-(2,3-dihydrobenzo[*b*][1,4]dioxin-6-yl)-2-methylbenzyl)oxy)benzyl)-*D*-serine (**4c**):

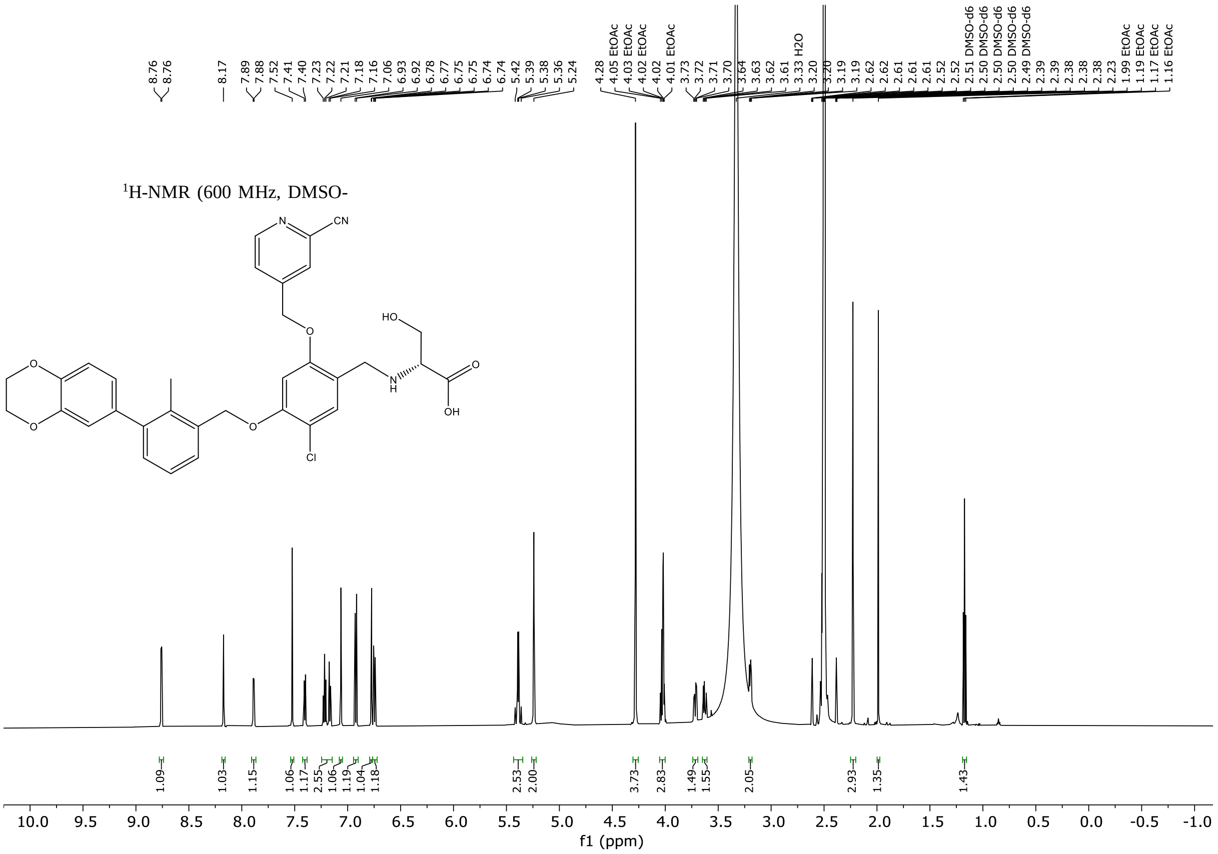

^{1}H-NMR (600 MHz, DMSO-d_6): δ 8.76 (d, *J* = 5.1 Hz, 1H), 8.17 (s, 1H), 7.89 (d, *J* = 5.1 Hz, 1H), 7.52 (s, 1H), 7.40 (d, *J* = 7.5 Hz, 1H), 7.22 (t, *J* = 7.5 Hz, 1H), 7.17 (d, *J* = 7.6 Hz, 1H), 7.06 (s, 1H), 6.92 (d, *J* = 8.2 Hz, 1H), 6.77 (d, *J* = 2.1 Hz, 1H), 6.75 (dd, *J* = 8.2 Hz, *J* = 2.1 Hz, 1H), 5.39 (d, *J* = 5.4 Hz, 2H), 5.24 (s, 2H), 4.28 (s, 4H), 4.02 (s, 2H), 3.73 – 3.61 (m, 2H), 3.19 (m, 1H), 2.23 (s, 3H).

Figure S66: (5-Chloro-2-((2-cyanopyridin-4-yl)methoxy)-4-((3-(2,3-dihydrobenzo[*b*][1,4]dioxin-6-yl)-2-methylbenzyl)oxy)benzyl)-*D*-serine (**4c**):

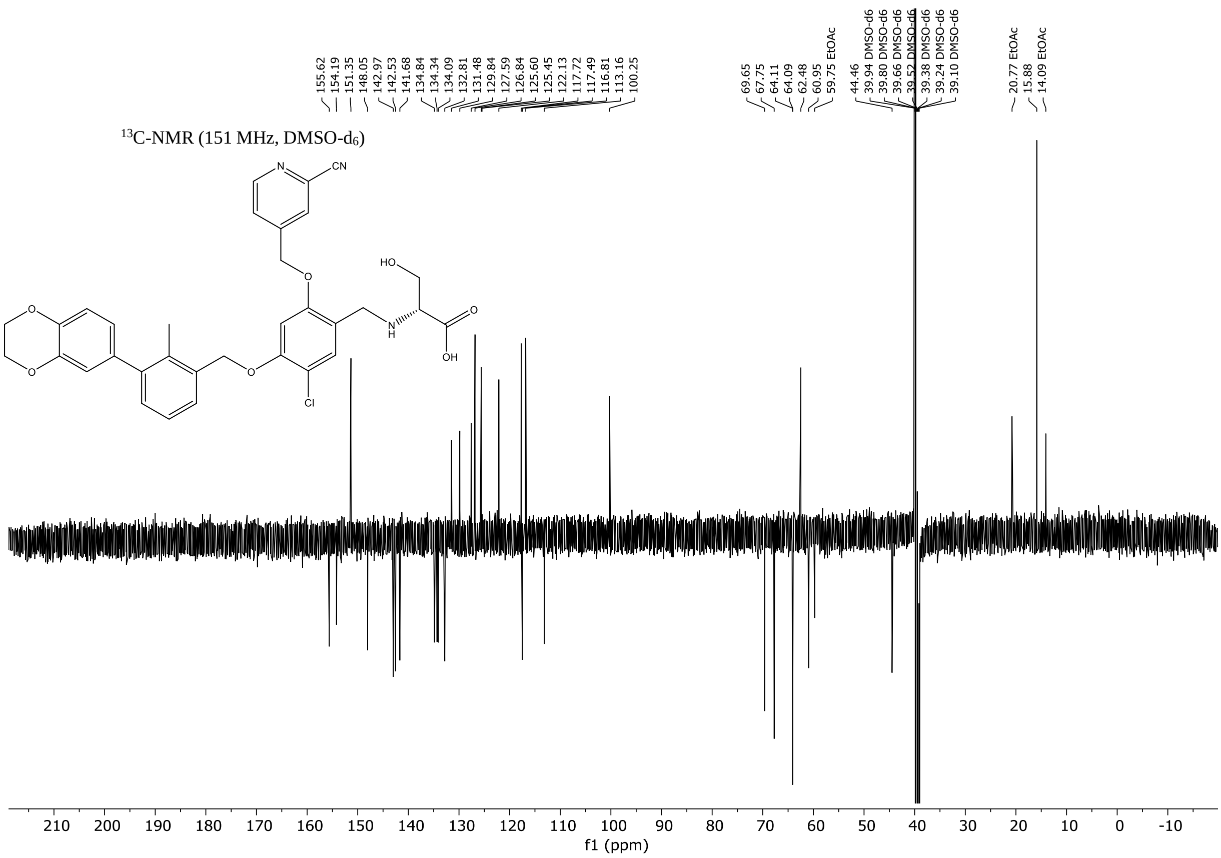

^{13}C-NMR (151 MHz, DMSO-d$_6$): δ 155.62, 154.19, 151.35, 148.05, 142.97, 142.53, 141.68, 134.84, 134.34, 134.09, 132.81, 131.48, 129.84, 127.59, 126.84, 125.60, 125.45, 122.13, 117.72, 117.49, 116.81, 113.16, 100.25, 69.65, 67.75, 64.11, 64.09, 62.48, 60.95, 44.46, 15.88.

Figure S67: *N*-(2-((5-chloro-2-((2-cyanopyridin-4-yl)methoxy)-4-((3-(2,3-dihydrobenzo[*b*][1,4]dioxin-6-yl)-2-methylbenzyl)oxy)benzyl)amino)ethyl)acetamide (**4d**):

^{1}H-NMR (600 MHz, $CDCl_3$)

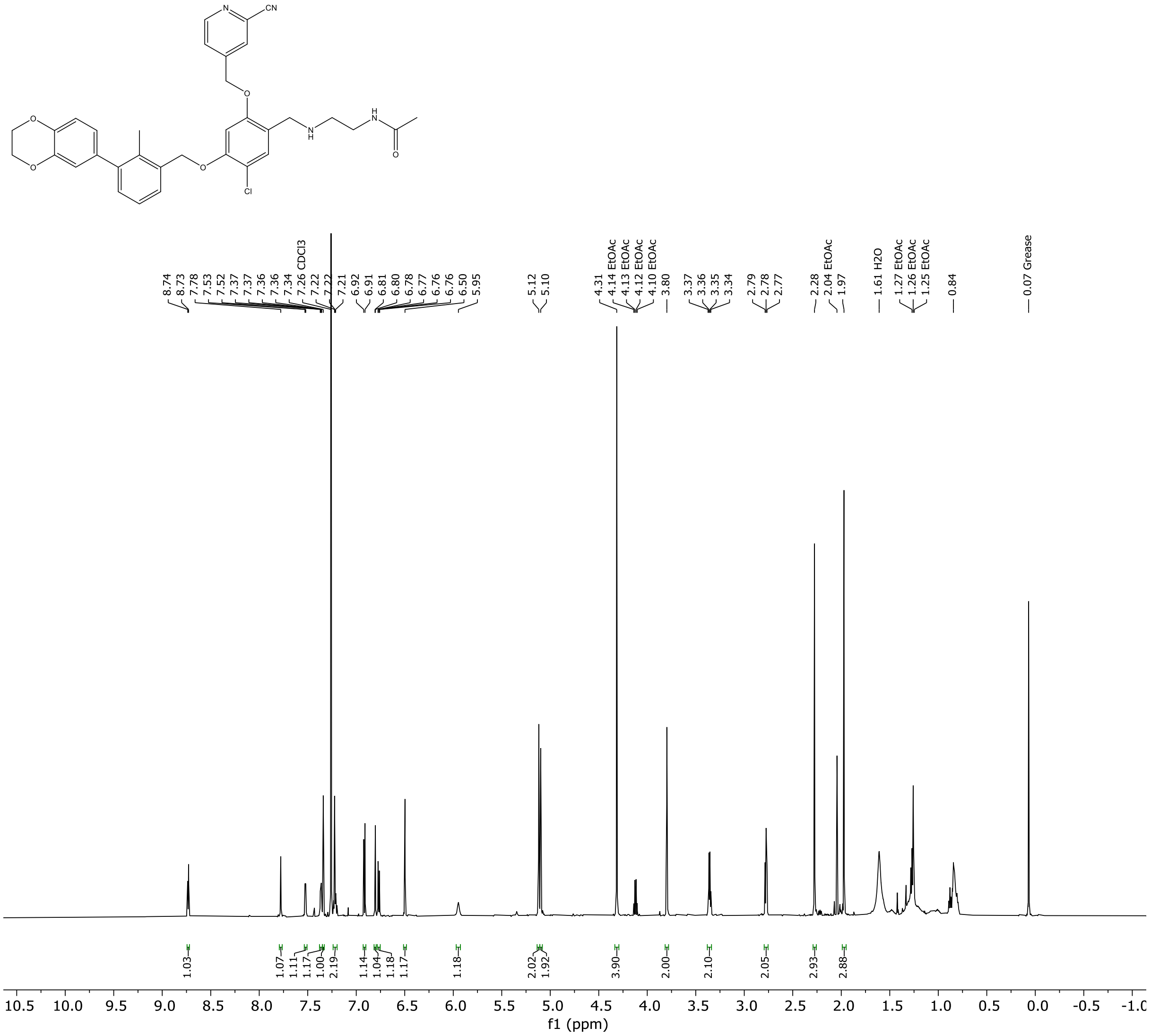

^{1}H-NMR (600 MHz, $CDCl_3$): δ 8.73 (d, *J* = 5 Hz, 1H), 7.78 (s, 1H), 7.52 (d, *J* = 5.0 Hz, 1H), 7.36 (dd, *J* = 6.3 Hz, *J* = 2.7 Hz, 1H), 7.34 (s, 1H), 7.22 (m, 2H), 6.92 (d, *J* = 8.2 Hz, 1H), 6.80 (d, *J* = 2.1 Hz, 1H), 6.77 (dd, *J* = 8.2 Hz, *J* = 2.1 Hz, 1H), 6.50 (s, 1H), 5.95 (br s, 1H), 5.12 (s, 2H), 5.11 (s, 2H), 4.31 (s, 4H), 3.80 (s, 2H), 3.36 (q, *J* = 5.8 Hz, 2H), 2.78 (t, *J* = 5.9 Hz, 2H), 2.28 (s, 3H), 1.97 (s, 3H).

Figure S68: *N*-(2-((5-chloro-2-((2-cyanopyridin-4-yl)methoxy)-4-((3-(2,3-dihydrobenzo[*b*][1,4]dioxin-6-yl)-2-methylbenzyl)oxy)benzyl)amino)ethyl)acetamide (**4d**):

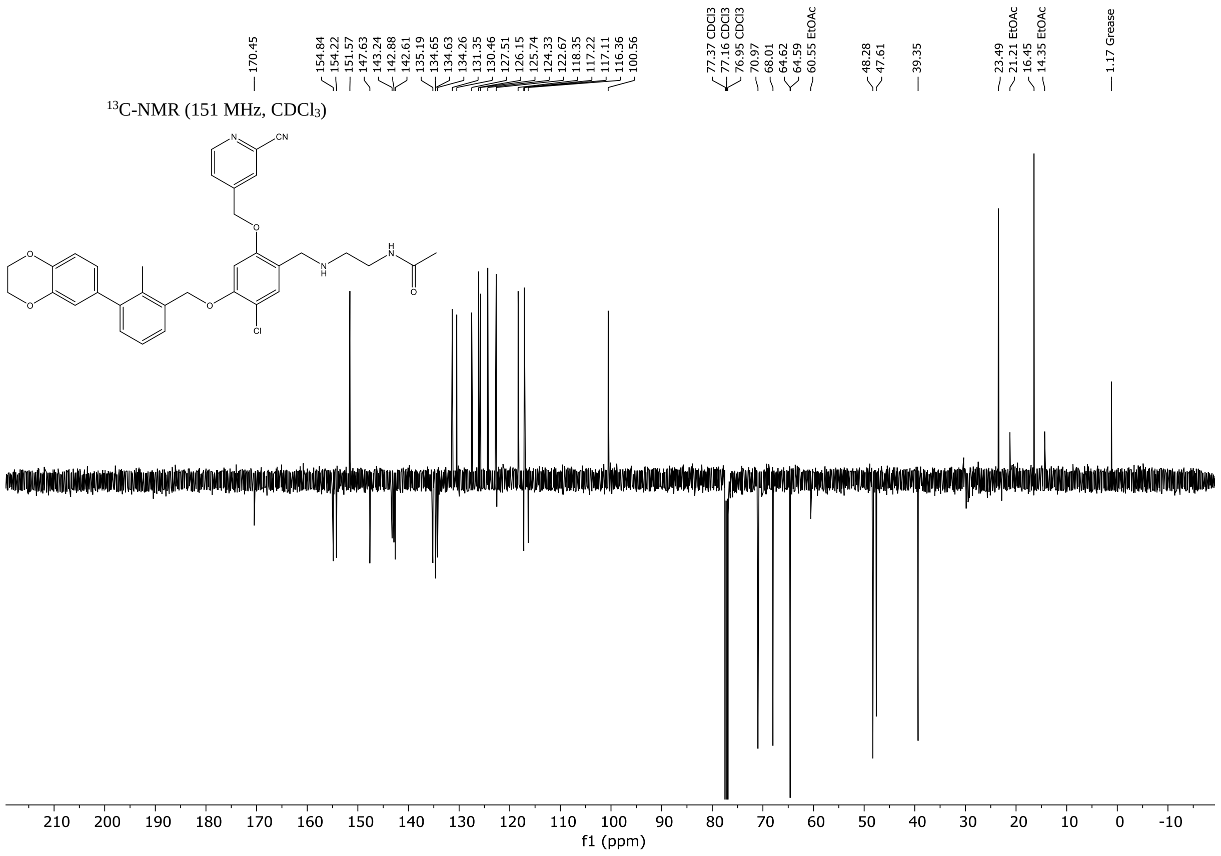

^{13}C-NMR (151 MHz, $CDCl_3$): δ 170.45, 154.84, 154.22, 151.57, 147.63, 143.24, 142.88, 142.61, 135.19, 134.65, 134.63, 134.26, 131.35, 130.46, 127.51, 126.15, 125.74, 124.33, 122.67, 118.35, 117.22, 117.11, 116.36, 100.56, 70.97, 68.01, 64.62, 64.59, 48.28, 47.61, 39.35, 23.49, 16.45.

Figure S69: 4-((4-Chloro-5-((3-(2,3-dihydrobenzo[*b*][1,4]dioxin-6-yl)-2-methylbenzyl)oxy)-2-(((2-hydroxyethyl)amino)methyl)phenoxy)methyl)picolinonitrile (**4e**):

^{1}H-NMR (600 MHz, $CDCl_3$)

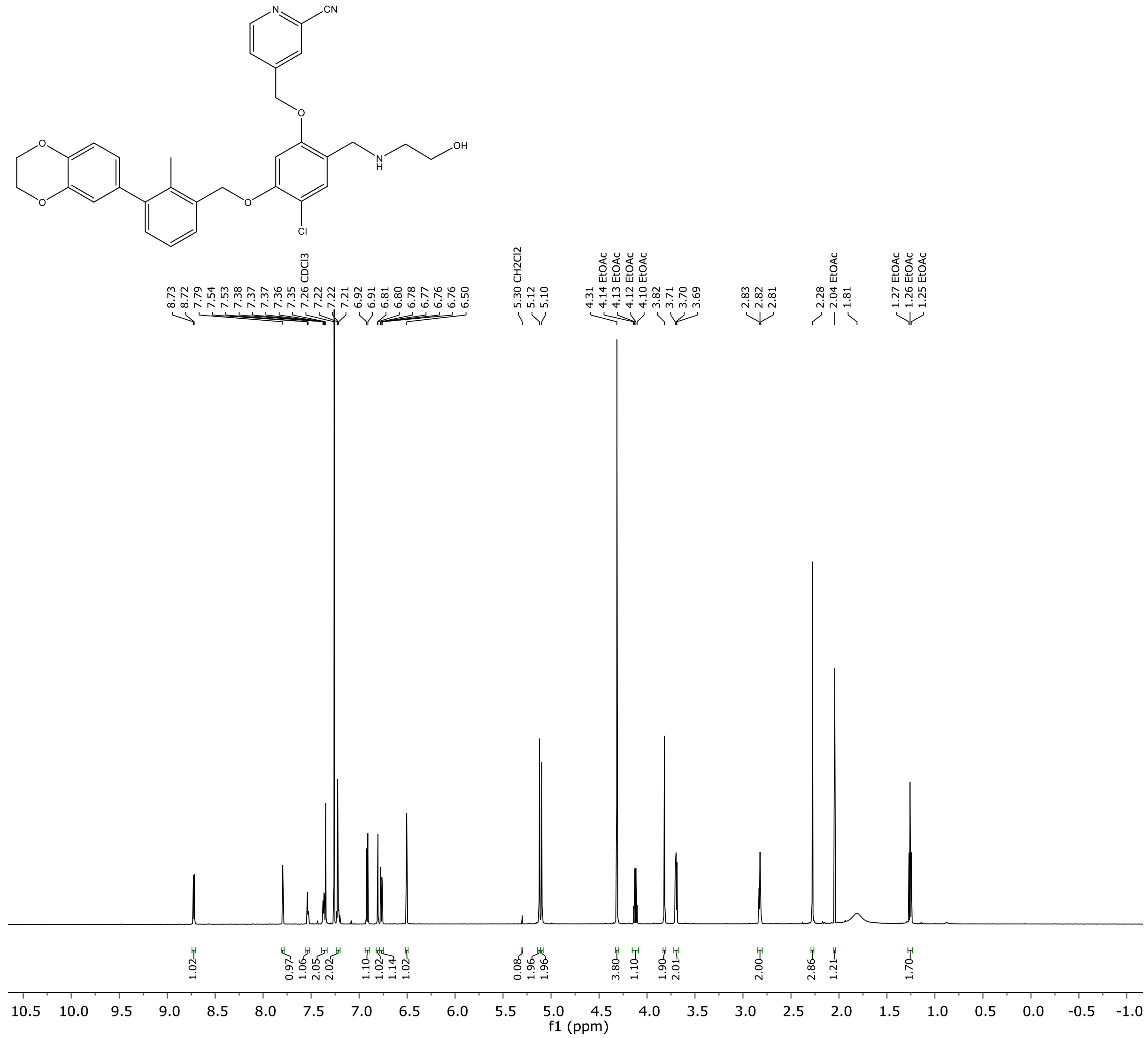

^{1}H-NMR (600 MHz, $CDCl_3$): δ 8.73 (d, *J* = 5.0 Hz, 1H), 7.79 (s, 1H), 7.53 (d, *J* = 5.0 Hz, 1H), 7.37 (dd, *J* = 6.3 Hz, *J* = 2.9 Hz, 1H), 7.35 (s, 1H), 7.22 – 7.21 (m, 2H), 6.92 (d, *J* = 8.2 Hz, 1H), 6.81 (d, *J* = 2.1 Hz, 1H), 6.77 (dd, *J* = 8.2 Hz, *J* = 2.1 Hz, 1H), 6.50 (s, 1H), 5.12 (s, 2H), 5.10 (s, 2H), 4.31 (s, 4H), 3.82 (s, 2H), 3.70 (t, *J* = 5.1 Hz, 2H), 2.82 (t, *J* = 5.1 Hz, 2H), 2.28 (s, 3H).

Figure S70: 4-((4-Chloro-5-((3-(2,3-dihydrobenzo[*b*][1,4]dioxin-6-yl)-2-methylbenzyl)oxy)-2-(((2-hydroxyethyl)amino)methyl)phenoxy)methyl)picolinonitrile (**4e**):

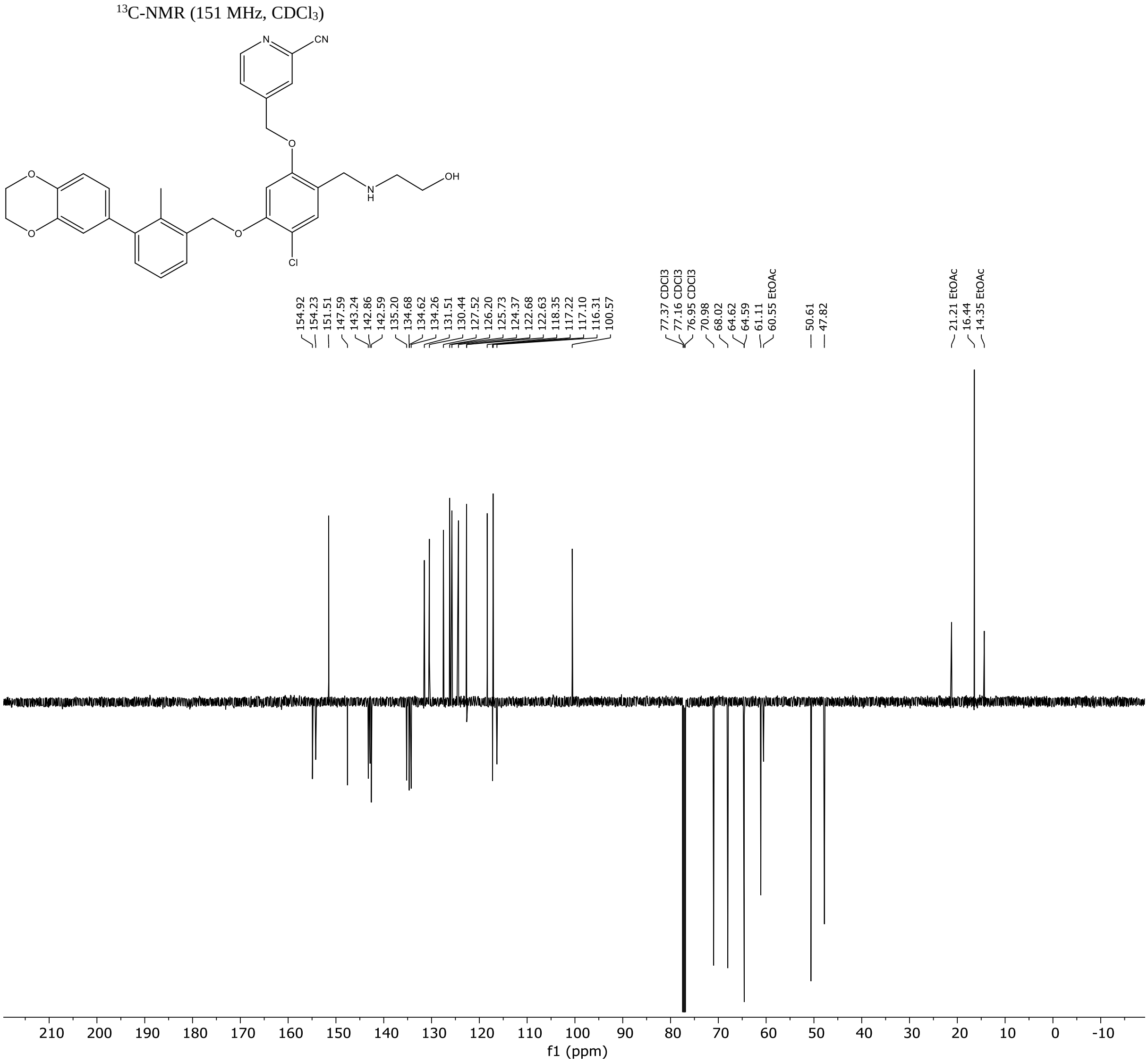

^{13}C-NMR (151 MHz, CDCl$_3$): δ 154.92, 154.23, 151.51, 147.59, 143.24, 142.86, 142.59, 135.20, 134.68, 134.62, 134.26, 131.51, 130.44, 127.52, 126.20, 125.73, 124.37, 122.68, 122.63, 118.35, 117.22, 117.10, 116.31, 100.57, 70.98, 68.02, 64.62, 64.59, 61.11, 50.61, 47.82, 16.44.

Figure S71: (*S*)-1-(5-chloro-2-((2-cyanopyridin-4-yl)methoxy)-4-((3-(2,3-dihydrobenzo[*b*][1,4]dioxin-6-yl)-2-methylbenzyl)oxy)benzyl)piperidine-2-carboxylic acid (**4f**):

^{1}H-NMR (600 MHz, CDCl$_3$)

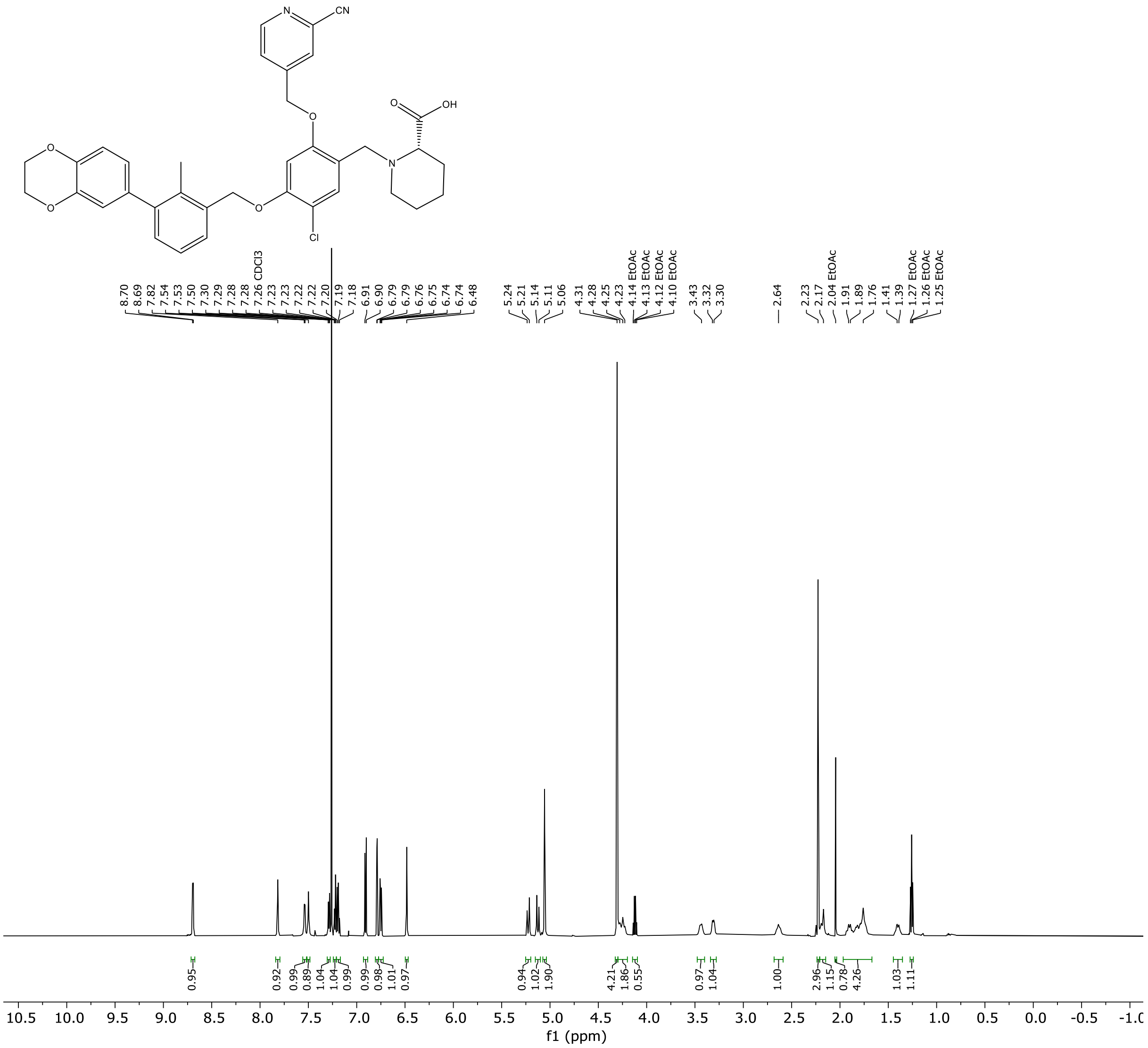

^{1}H-NMR (600 MHz, CDCl$_3$): δ 8.72 (d, *J* = 5.0 Hz, 1H), 7.80 (s, 1H), 7.53 (d, *J* = 5.0 Hz, 1H), 7.50 (s, 1H), 7.29 (dd, *J* = 7.6 Hz, *J* = 1.7 Hz, 1H), 7.22 (dd, *J* = 7.6 Hz, *J* = 1.7 Hz, 1H), 7.19 (t, *J* = 7.6 Hz, 1H), 6.91 (d, *J* = 8.2 Hz, 1H), 6.79 (d, *J* = 2.1 Hz, 1H), 6.75 (dd, *J* = 8.2 Hz, *J* = 2.1 Hz, 1H), 6.48 (s, 1H), 5.23 (d, *J* = 14 Hz, 1H), 5.13 (d, *J* = 14 Hz, 1H), 5.10 (s, 2H), 4.31 (s, 4H), 4.31 – 4.23 (m, 2H), 3.57 (s, 2H), 3.43 (m, 1H), 3.31 (m, 1H), 2.64 (m, 1H), 2.23 (s, 3H), 2.17 (m, 1H), 1.94 – 1.73 (m, 4H), 1.40 (m, 1H).

Figure S72: (*S*)-1-(5-chloro-2-((2-cyanopyridin-4-yl)methoxy)-4-((3-(2,3-dihydrobenzo[*b*][1,4]dioxin-6-yl)-2-methylbenzyl)oxy)benzyl)piperidine-2-carboxylic acid (**4f**):

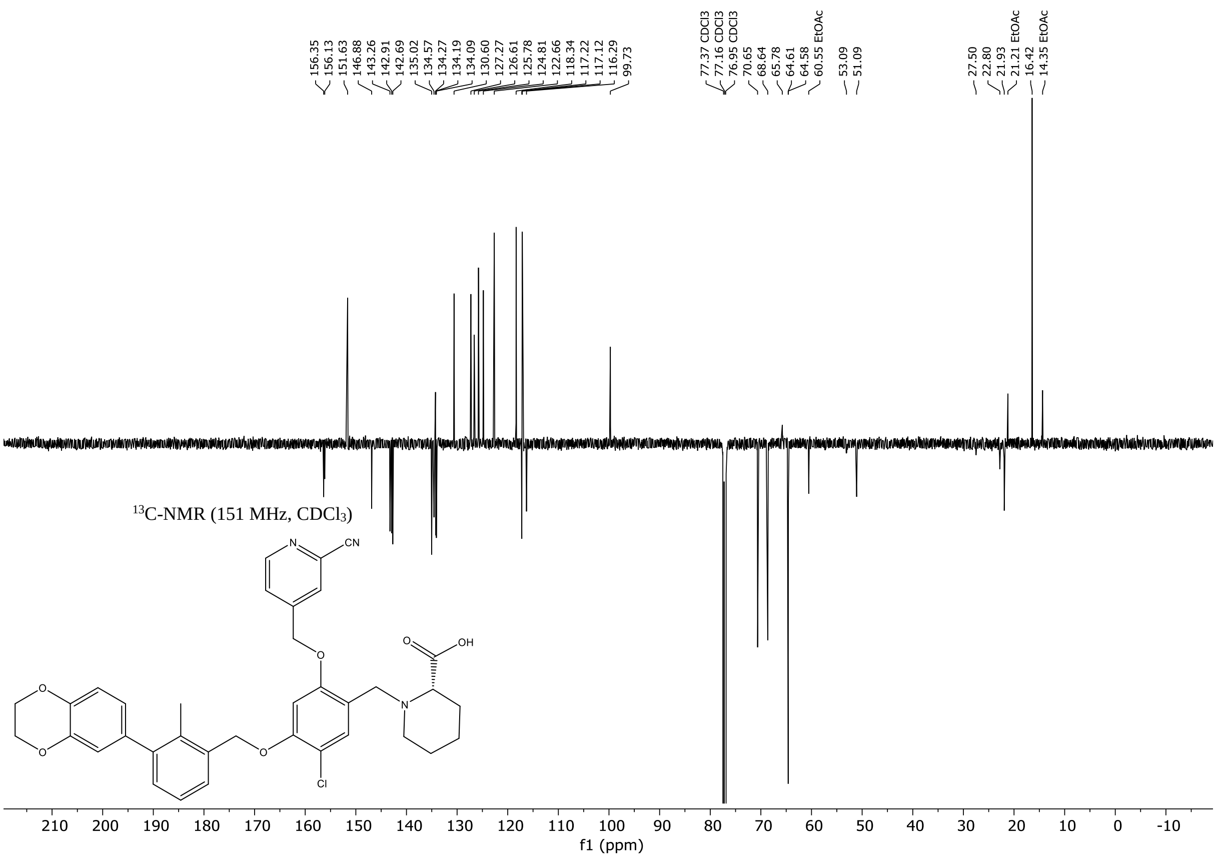

^{13}C-NMR (151 MHz, $CDCl_3$): δ 156.35, 156.13, 151.63, 146.88, 143.26, 142.91, 142.69, 135.02, 134.57, 134.27, 134.19, 134.09, 130.60, 127.27, 126.61, 125.78, 124.81, 122.66, 118.34, 117.22, 117.12, 116.29, 99.73, 70.65, 68.64, 65.78, 64.61, 64.58, 53.09, 51.09, 27.50, 22.80, 21.93, 16.42.

Figure S73: 1-(5-Chloro-2-((2-cyanopyridin-4-yl)methoxy)-4-((3-(2,3-dihydrobenzo[*b*][1,4]dioxin-6-yl)-2-methylbenzyl)oxy)phenyl)-5,8,11,14-tetraoxa-2-azaheptadecan-17-oic acid (**4g**):

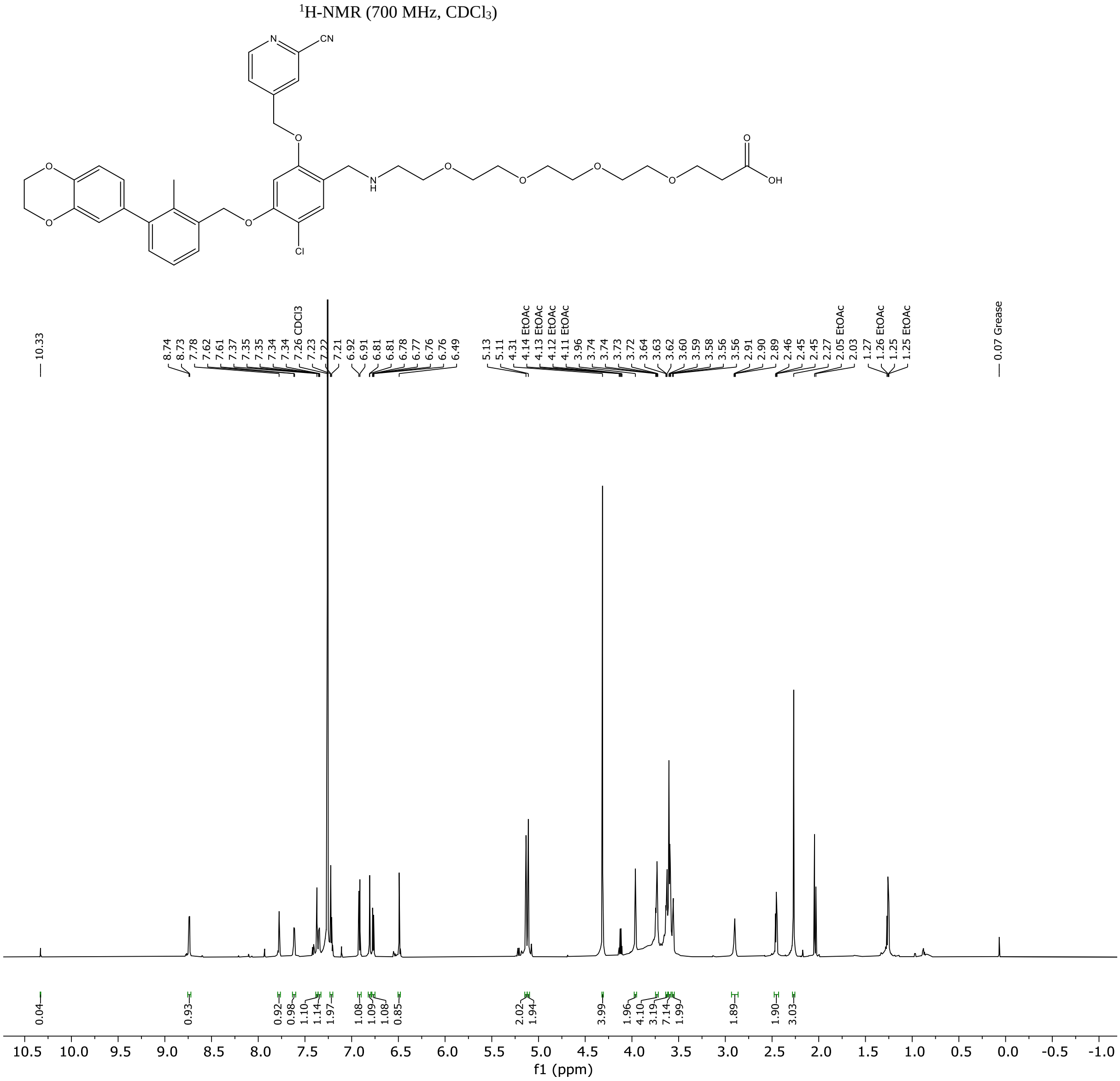

^{1}H-NMR (700 MHz, $CDCl_3$): δ 8.74 (d, *J* = 5.0 Hz, 1H), 7.78 (s, 1H), 7.62 (d, *J* = 5.0 Hz, 1H), 7.37 (s, 1H), 7.35 – 7.34 (m, 1H), 7.23 – 7.21 (m, 2H), 6.92 (d, *J* = 8.2 Hz, 1H), 6.81 (d, *J* = 2.0 Hz, 1H), 6.77 (dd, *J* = 8.2 Hz, *J* = 5.0 Hz, 1H), 6.49 (s, 1H), 5.13 (s, 1H), 5.11 (s, 1H), 4.31 (s, 4H), 3.96 (s, 2H), 3.74 – 3.72 (m, 4H), 3.64 – 3.56 (m, 12H), 2.90 (m, 2H), 2.45 (t, *J* = 5.6 Hz, 2H), 2.27 (s, 3H).

Figure S74: 1-(5-Chloro-2-((2-cyanopyridin-4-yl)methoxy)-4-((3-(2,3-dihydrobenzo[*b*][1,4]dioxin-6-yl)-2-methylbenzyl)oxy)phenyl)-5,8,11,14-tetraoxa-2-azaheptadecan-17-oic acid (**4g**):

^{13}C-NMR (176 MHz, $CDCl_3$)

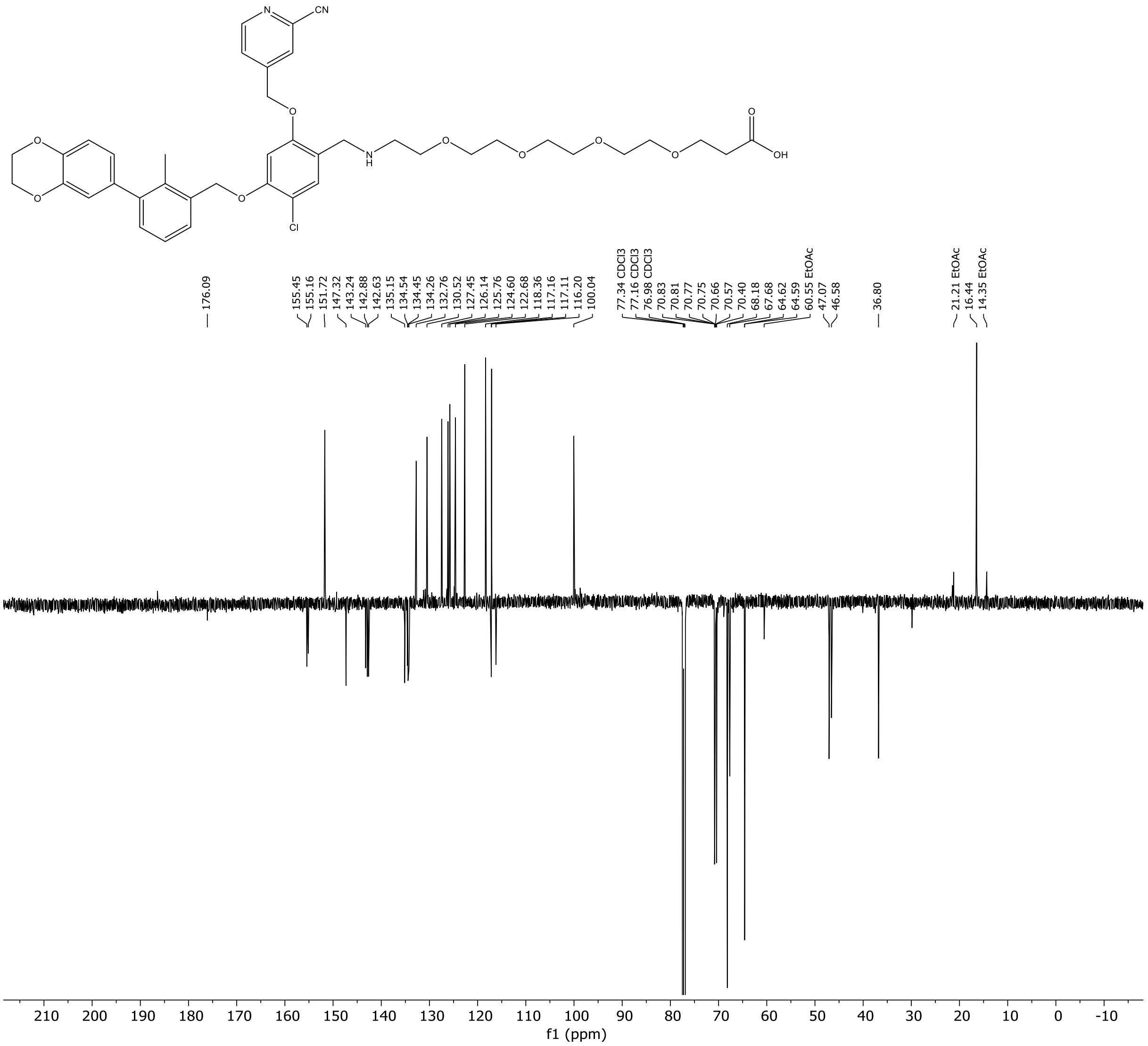

^{13}C-NMR (176 MHz, $CDCl_3$): δ 176.09, 155.45, 155.16, 151.72, 147.32, 143.24, 142.88, 142.63, 135.15, 134.54, 134.45, 134.26, 132.76, 130.52, 127.45, 126.14, 125.76, 124.60, 122.68, 118.36, 117.16, 117.11, 116.20, 100.04, 70.83, 70.81, 70.77, 70.75, 70.66, 70.57, 70.40, 68.18, 67.68, 64.62, 64.59, 47.07, 46.58, 36.80, 16.44.

Figure S75: 1-(5-Chloro-2-((2-cyanopyridin-4-yl)methoxy)-4-((3-(2,3-dihydrobenzo[*b*][1,4]dioxin-6-yl)-2-methylbenzyl)oxy)phenyl)-5,8,11,14-tetraoxa-2-azaheptadecan-17-oic acid (**4g**):

^{1}H-^{1}H COSY NMR (700 MHz, $CDCl_3$)

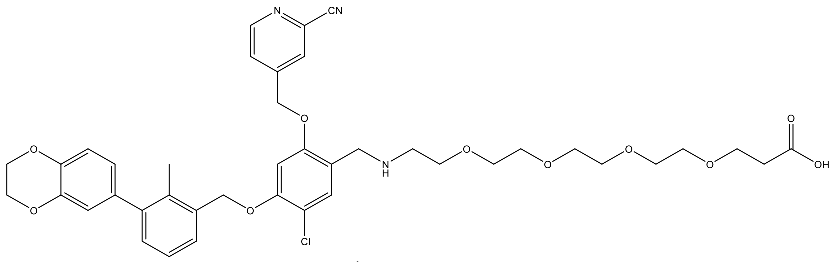

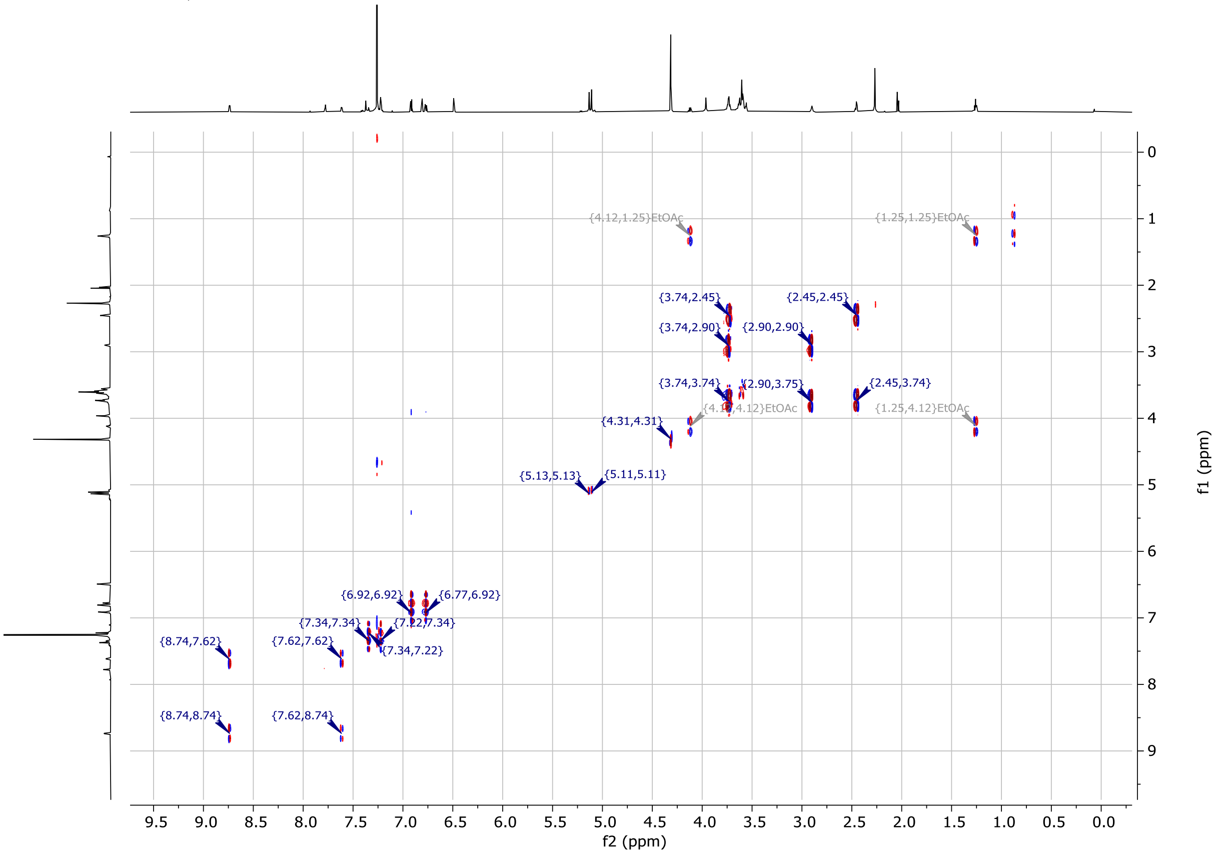

^{1}H-NMR (700 MHz, $CDCl_3$): δ 8.74, 8.74, 7.62, 7.62, 7.34, 7.34, 7.22, 7.22, 6.92, 6.92, 6.77, 6.77, 5.13, 5.11, 4.31, 3.75, 3.74, 3.74, 3.60, 2.90, 2.90, 2.45, 2.45.

^{1}H-NMR (700 MHz, $CDCl_3$): δ 8.74, 8.74, 7.62, 7.62, 7.34, 7.34, 7.22, 7.22, 6.92, 6.92, 6.77, 6.77, 5.13, 5.11, 4.31, 3.75, 3.74, 3.74, 3.60, 2.90, 2.90, 2.45, 2.45.

Figure S76: (*S*)-2-((5-chloro-2-((2-cyanopyridin-4-yl)methoxy)-4-((3-(2,3-dihydrobenzo[*b*][1,4]dioxin-6-yl)-2-methylbenzyl)oxy)benzyl)amino)hex-5-ynoic acid (**4h**):

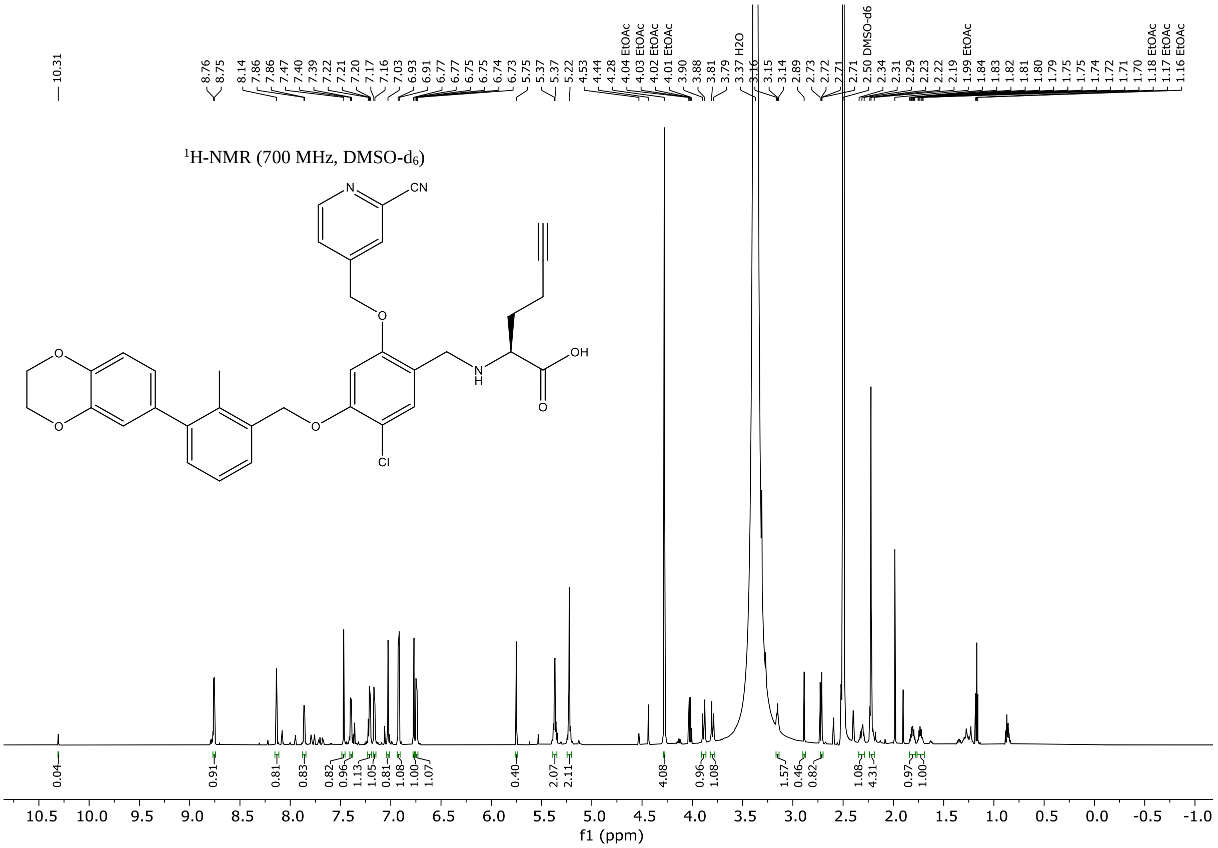

^{1}H-NMR (700 MHz, DMSO-d$_6$): δ 8.76 (d, *J* = 5.0 Hz, 1H), 8.14 (s, 1H), 7.86 (d, *J* = 5.0 Hz, 1H), 7.47 (s, 1H), 7.40 (d, *J* = 7.4 Hz, 1H), 7.21 (t, *J* = 7.4 Hz, 1H), 7.16 (d, *J* = 7.4 Hz, 1H), 7.03 (s, 1H), 6.92 (d, *J* = 8.2 Hz, 1H), 6.77 (d, *J* = 2.1 Hz, 1H), 6.74 (dd, *J* = 8.2 Hz, *J* = 2.1 Hz, 1H), 5.37 (m, 2H), 5.22 (s, 2H), 4.28 (s, 4H), 3.89 (d, *J* = 14 Hz, 1H), 3.80 (d, *J* = 14 Hz, 1H), 3.15 (t, *J* = 6.4 Hz, 1H), 2.71 (t, *J* = 2.6 Hz, 1H), 2.34 – 2.29 (m, 1H), 2.23 – 2.19 (m, 1H), 2.22 (s, 3H), 1.84 – 1.79 (m, 1H), 1.75 – 1.70 (m, 1H).

Figure S77: (*S*)-2-((5-chloro-2-((2-cyanopyridin-4-yl)methoxy)-4-((3-(2,3-dihydrobenzo[*b*][1,4]dioxin-6-yl)-2-methylbenzyl)oxy)benzyl)amino)hex-5-ynoic acid (**4h**):

^{13}C-NMR (176 MHz, DMSO-d$_6$)

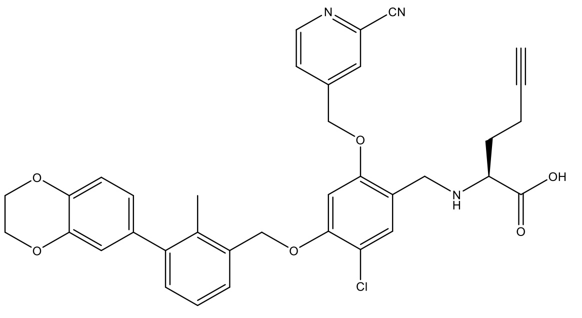

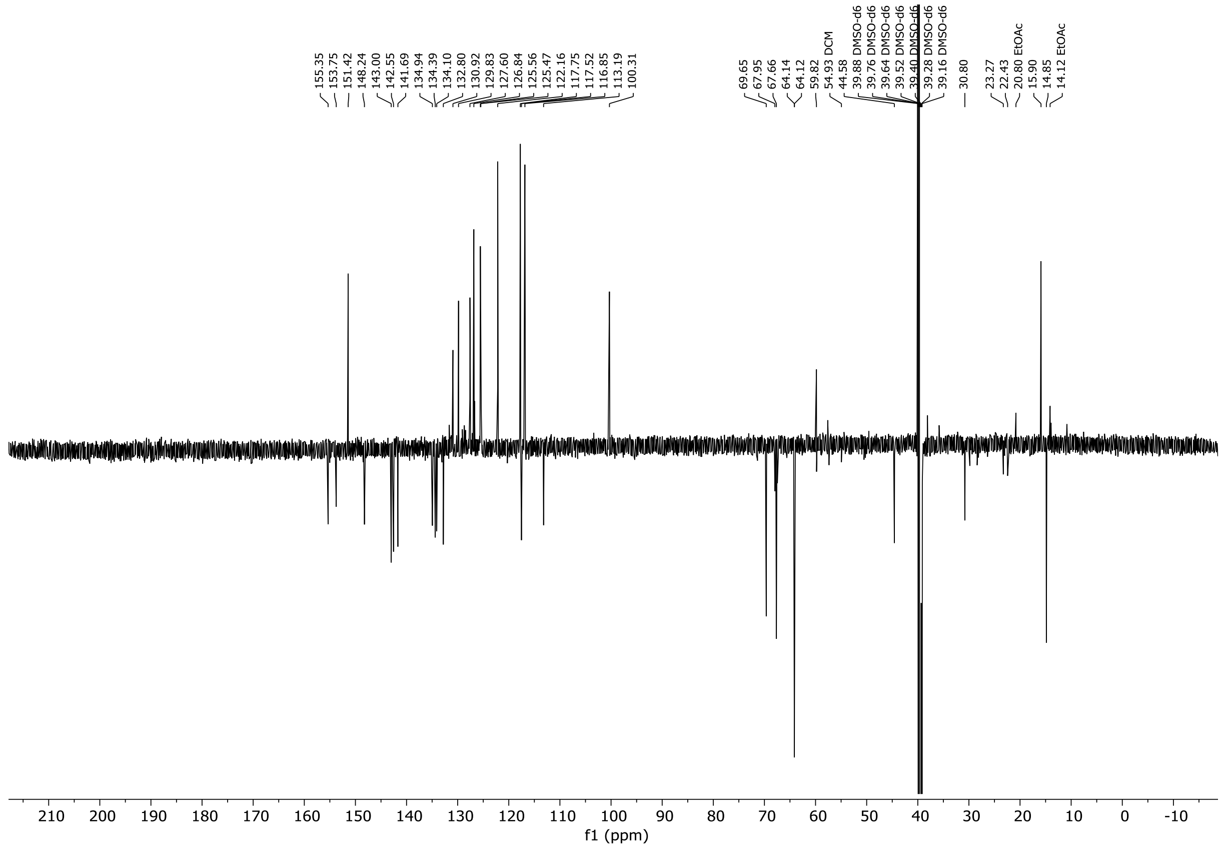

^{13}C-NMR (176 MHz, DMSO-d$_6$): δ 155.35, 153.75, 151.42, 148.24, 143.00, 142.55, 141.69, 134.94, 134.39, 134.10, 132.80, 130.92, 129.83, 127.60, 126.84, 125.56, 125.47, 122.16, 117.75, 117.52, 116.85, 113.19, 100.31, 69.65, 67.95, 67.66, 64.14, 64.12, 59.82, 54.93, 44.58, 39.88, 30.80, 23.27, 22.43, 15.90, 14.85.

Figure S78: (*S*)-2-((5-chloro-2-((2-cyanopyridin-4-yl)methoxy)-4-((3-(2,3-dihydrobenzo[*b*][1,4]dioxin-6-yl)-2-methylbenzyl)oxy)benzyl)amino)hex-5-ynoic acid (**4h**):

^{1}H-^{1}H COSY NMR (700 MHz, DMSO-d$_6$)

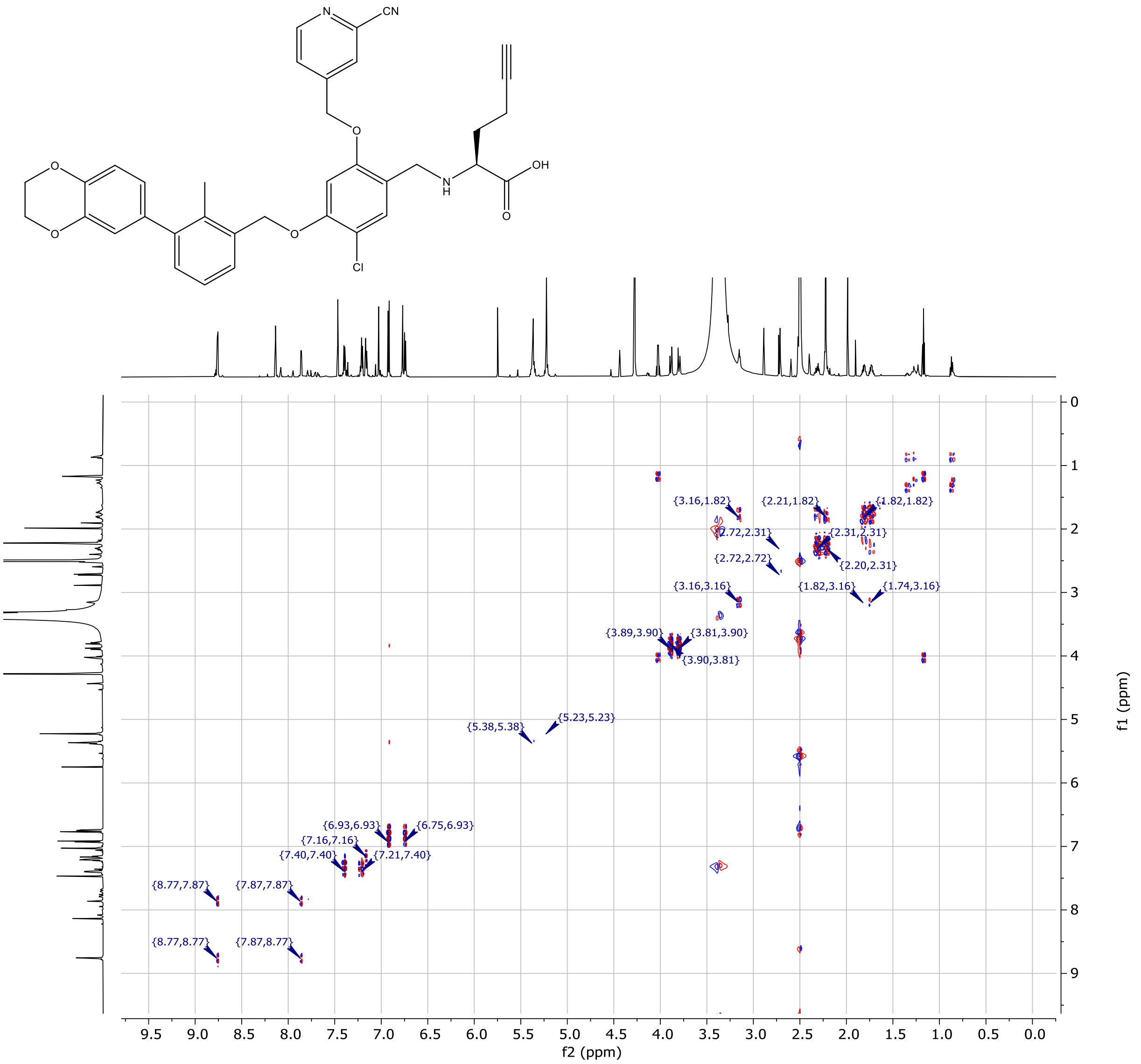

^{1}H-NMR (700 MHz, DMSO-d$_6$): δ 8.77, 8.77, 8.14, 7.87, 7.87, 7.40, 7.40, 7.21, 7.16, 6.93, 6.93, 6.78, 6.78, 6.75, 6.75, 5.38, 5.23, 3.90, 3.90, 3.81, 3.81, 3.16, 3.16, 3.16, 2.72, 2.31, 2.31, 2.31, 2.31, 2.20, 2.20, 2.20, 2.20, 1.82, 1.82, 1.82, 1.82, 1.74, 1.74, 1.74, 1.74.

^{1}H-NMR (700 MHz, DMSO-d$_6$): δ 8.77, 8.77, 8.14, 7.87, 7.87, 7.40, 7.40, 7.21, 7.16, 6.93, 6.93, 6.93, 6.75, 6.75, 6.75, 5.38, 5.23, 3.90, 3.89, 3.81, 3.81, 3.16, 3.16, 3.16, 2.72, 2.72, 2.72, 2.31, 2.31, 2.31, 2.21, 2.21, 2.20, 1.82, 1.82, 1.82, 1.82, 1.74, 1.74, 1.74, 1.74.

Figure S79: (*S*)-2-((5-chloro-2-((2-cyanopyridin-4-yl)methoxy)-4-((3-(2,3-dihydrobenzo[*b*][1,4]dioxin-6-yl)-2-methylbenzyl)oxy)benzyl)amino)hex-5-ynoic acid (**4h**):

^{1}H-^{13}C HSQC NMR (700 MHz, 176 MHz, DMSO-d$_6$)

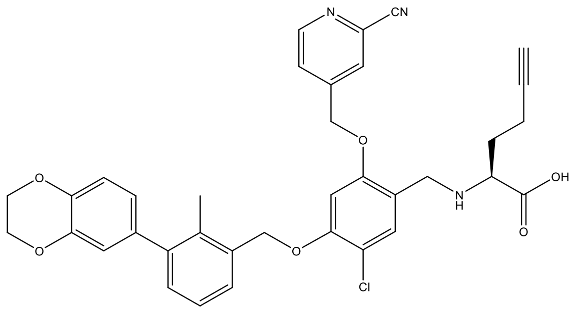

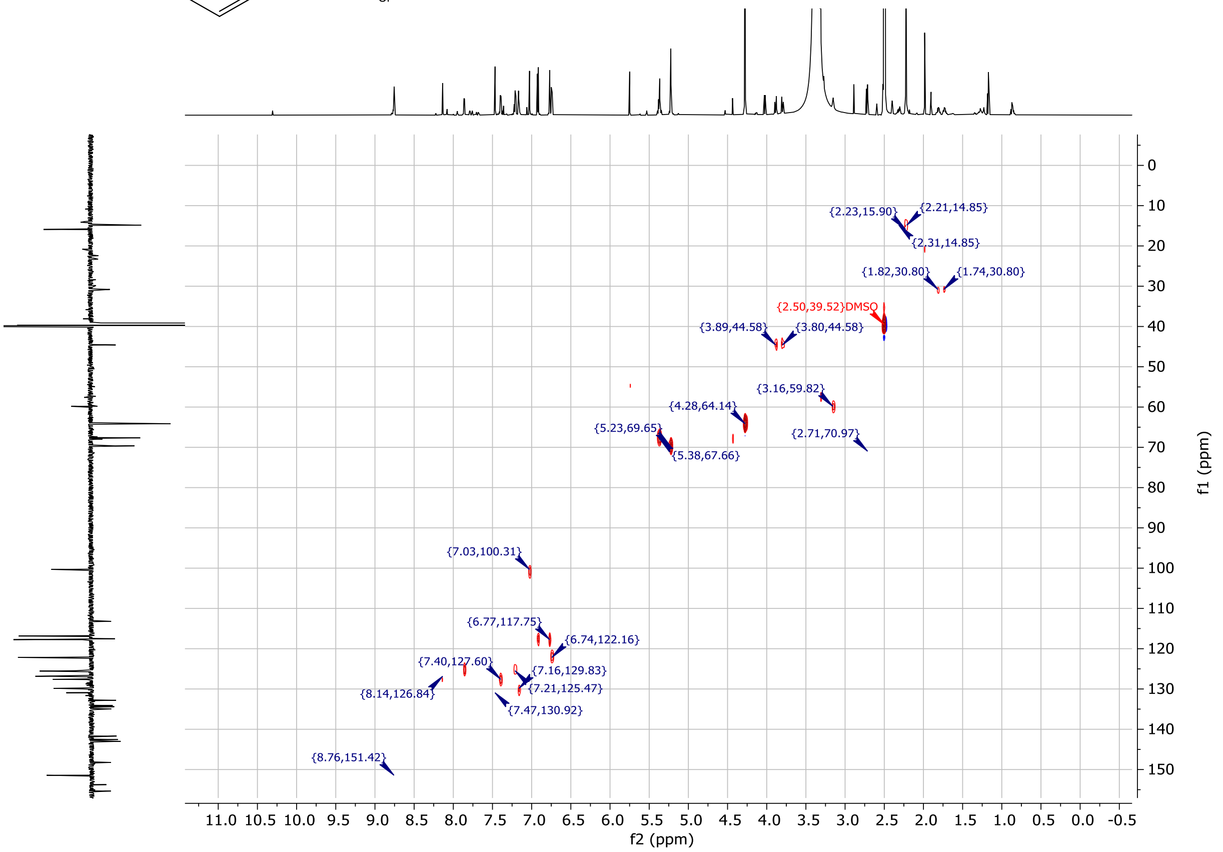

^{13}C-NMR (176 MHz, DMSO-d$_6$): δ 151.42, 130.92, 129.83, 127.60, 126.84, 125.57, 125.47, 122.16, 117.75, 116.85, 100.31, 70.97, 69.65, 67.66, 64.14, 59.82, 44.58, 44.58, 30.80, 30.80, 15.90, 14.85, 14.85

^{1}H-NMR (700 MHz, DMSO-d$_6$): δ 8.76, 8.14, 7.86, 7.47, 7.40, 7.21, 7.16, 7.03, 6.92, 6.77, 6.74, 5.38, 5.23, 4.28, 3.89, 3.80, 3.16, 2.71, 2.31, 2.23, 2.21, 1.82, 1.74.

Figure S80: N^2-(5-chloro-2-((2-cyanopyridin-4-yl)methoxy)-4-((3-(2,3-dihydrobenzo[*b*][1,4]dioxin-6-yl)-2-methylbenzyl)oxy)benzyl)-N^6-((prop-2-yn-1-yloxy)carbonyl)-L-lysine (**4i**):

^{1}H-NMR (700 MHz, DMSO-d$_6$)

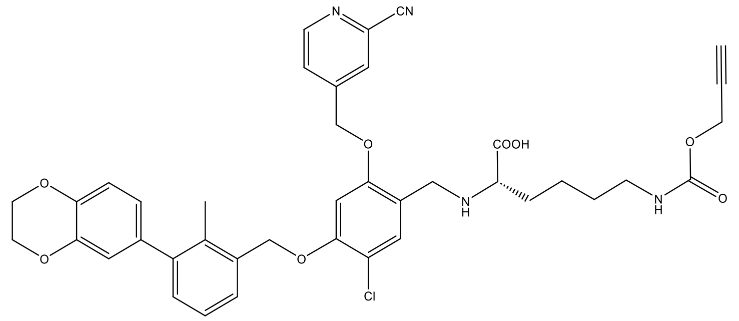

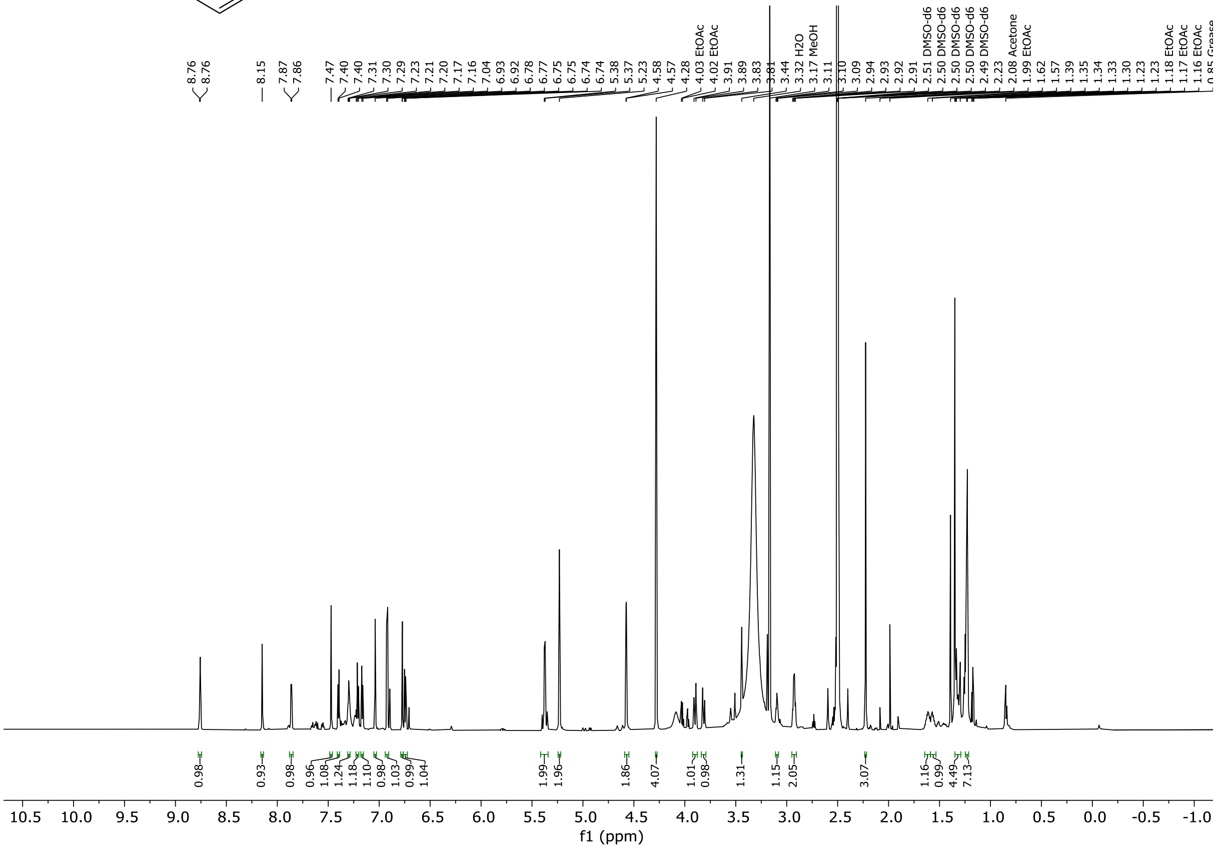

^{1}H-NMR (700 MHz, DMSO-d$_6$): δ 8.76 (d, J = 5.0 Hz, 1H), 8.15 (s, 1H), 7.86 (d, J = 5.0 Hz, 1H), 7.47 (s, 1H), 7.40 (d, J = 7.5 Hz, 1H), 7.21 (t, J = 7.5 Hz, 1H), 7.16 (d, J = 7.5 Hz, 1H), 7.04 (s, 1H), 6.92 (d, J = 8.2 Hz, 1H), 6.78 (d, J = 2.1 Hz, 1H), 6.74 (dd, J = 8.2 Hz, J = 2.1 Hz, 1H), 5.37 (m, 2H), 5.23 (s, 2H), 4.58 (d, J = 2.3 Hz, 2H), 4.28 (s, 4H), 3.90 (d, J = 14 Hz, 1H), 3.82 (d, J = 14 Hz, 1H), 3.44 (t, J = 2.3 Hz, 1H), 3.10 (t, J = 6.4 Hz, 1H), 2.94 – 2.91 (m, 2H), 2.34 – 2.29 (m, 1H), 2.23 (s, 3H), 1.64 – 1.59 (m, 1H), 1.58 – 1.55 (m, 1H), 1.35 – 1.30 (m, 4H).

Figure S81: *N*[2]-(5-chloro-2-((2-cyanopyridin-4-yl)methoxy)-4-((3-(2,3-dihydrobenzo[*b*][1,4]dioxin-6-yl)-2-methylbenzyl)oxy)benzyl)-*N*[6]-((prop-2-yn-1-yloxy)carbonyl)-L-lysine (**4i**):

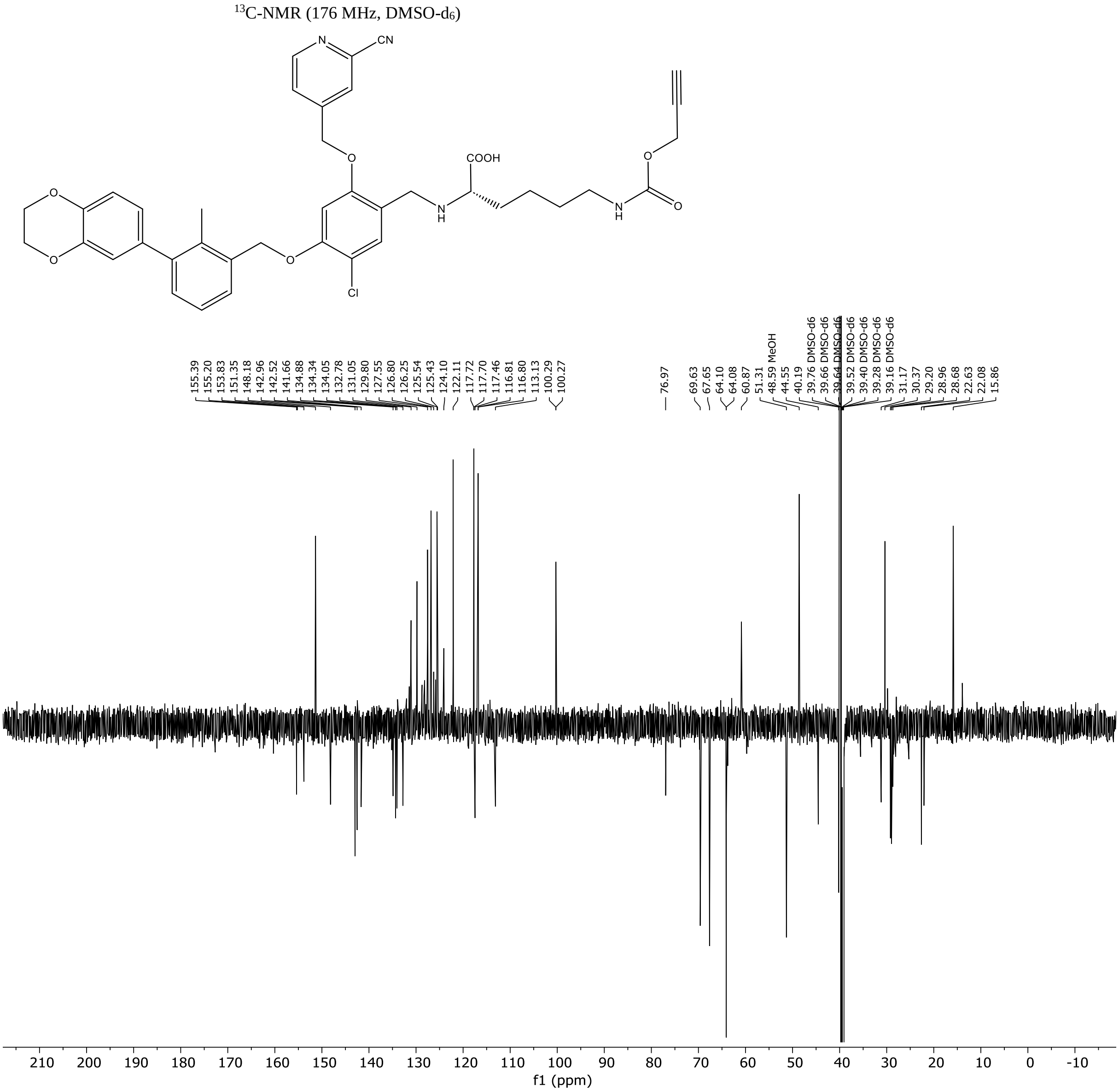

^{13}C-NMR (176 MHz, DMSO-d$_6$): δ 155.39, 155.20, 153.83, 151.35, 148.18, 142.96, 142.52, 141.66, 134.88, 134.34, 134.05, 132.78, 131.05, 129.80, 127.55, 126.80, 126.25, 125.54, 125.43, 124.10, 122.11, 117.72, 117.70, 117.46, 116.81, 116.80, 113.13, 100.29, 100.27, 76.97, 69.63, 67.65, 64.10, 64.08, 60.87, 51.31, 44.55, 40.19, 31.17, 30.37, 29.20, 28.96, 28.68, 22.63, 22.08, 15.86.

Figure S82: N^2-(5-chloro-2-((2-cyanopyridin-4-yl)methoxy)-4-((3-(2,3-dihydrobenzo[*b*][1,4]dioxin-6-yl)-2-methylbenzyl)oxy)benzyl)-N^6-((prop-2-yn-1-yloxy)carbonyl)-L-lysine (**4i**):

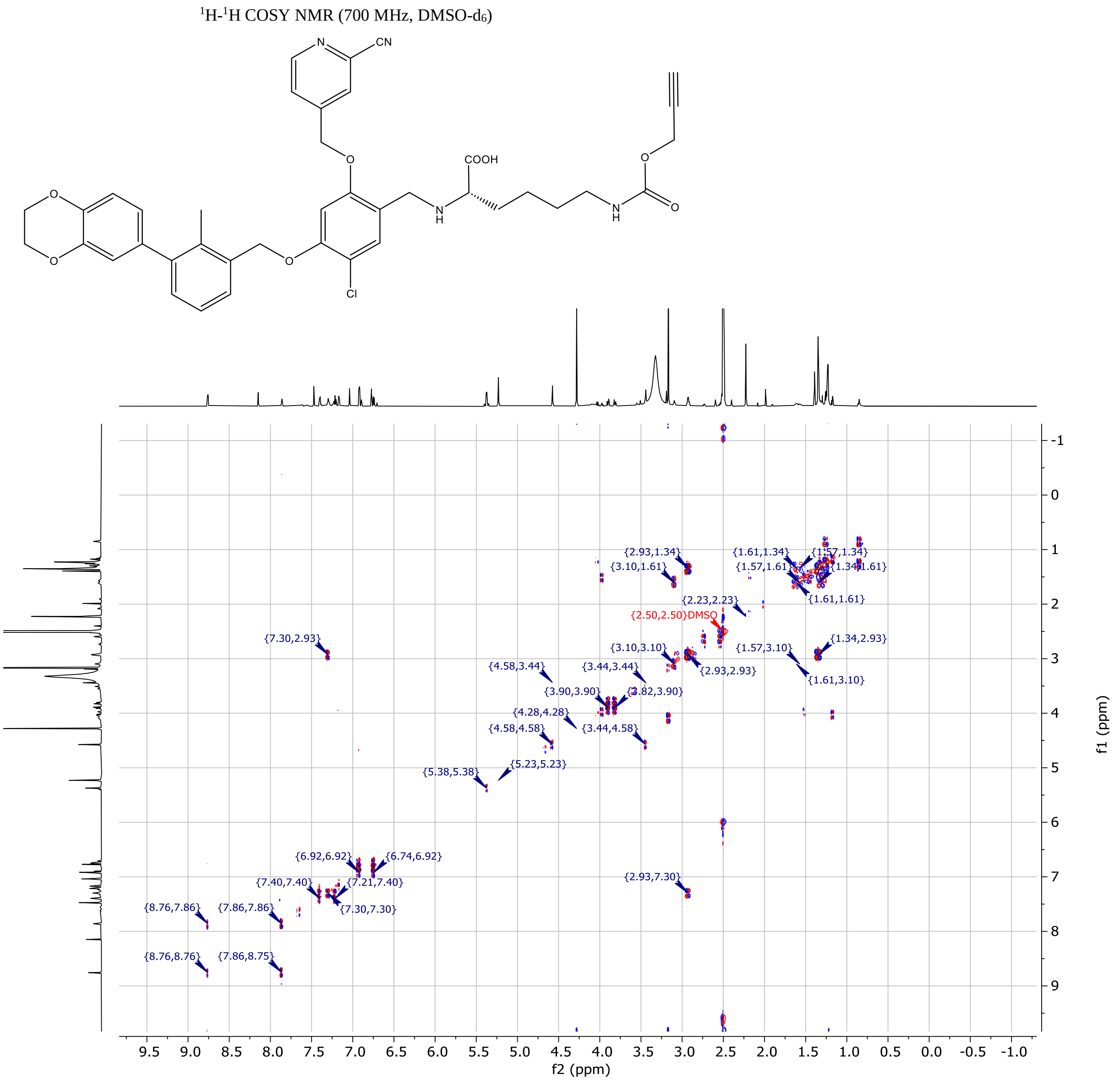

^{1}H-NMR (700 MHz, DMSO-d$_6$): δ 8.76, 8.75, 7.86, 7.86, 7.40, 7.40, 7.30, 7.30, 7.21, 7.17, 6.92, 6.92, 6.77, 6.77, 6.74, 6.74, 5.38, 5.23, 4.58, 4.58, 4.28, 3.90, 3.90, 3.82, 3.82, 3.44, 3.44, 3.10, 3.10, 3.10, 2.93, 2.93, 2.93, 2.23, 1.61, 1.61, 1.61, 1.61, 1.57, 1.57, 1.57, 1.57, 1.34, 1.34, 1.34, 1.34

^{1}H-NMR (700 MHz, DMSO-d$_6$): δ 8.76, 8.76, 7.86, 7.86, 7.40, 7.30, 7.30, 7.21, 7.21, 7.17, 6.92, 6.92, 6.92, 6.74, 6.74, 6.74, 5.38, 5.23, 4.58, 4.58, 4.28, 3.90, 3.90, 3.82, 3.82, 3.44, 3.44, 3.10, 3.10, 3.10, 2.93, 2.93, 2.93, 2.23, 1.61, 1.61, 1.61, 1.61, 1.57, 1.57, 1.57, 1.57, 1.34, 1.34, 1.34, 1.34.

Figure S83: N^2-(5-chloro-2-((2-cyanopyridin-4-yl)methoxy)-4-((3-(2,3-dihydrobenzo[*b*][1,4]dioxin-6-yl)-2-methylbenzyl)oxy)benzyl)-N^6-((prop-2-yn-1-yloxy)carbonyl)-L-lysine (**4i**):

^{1}H-^{13}C HSQC NMR (700 MHz, 176 MHz, DMSO-d_6)

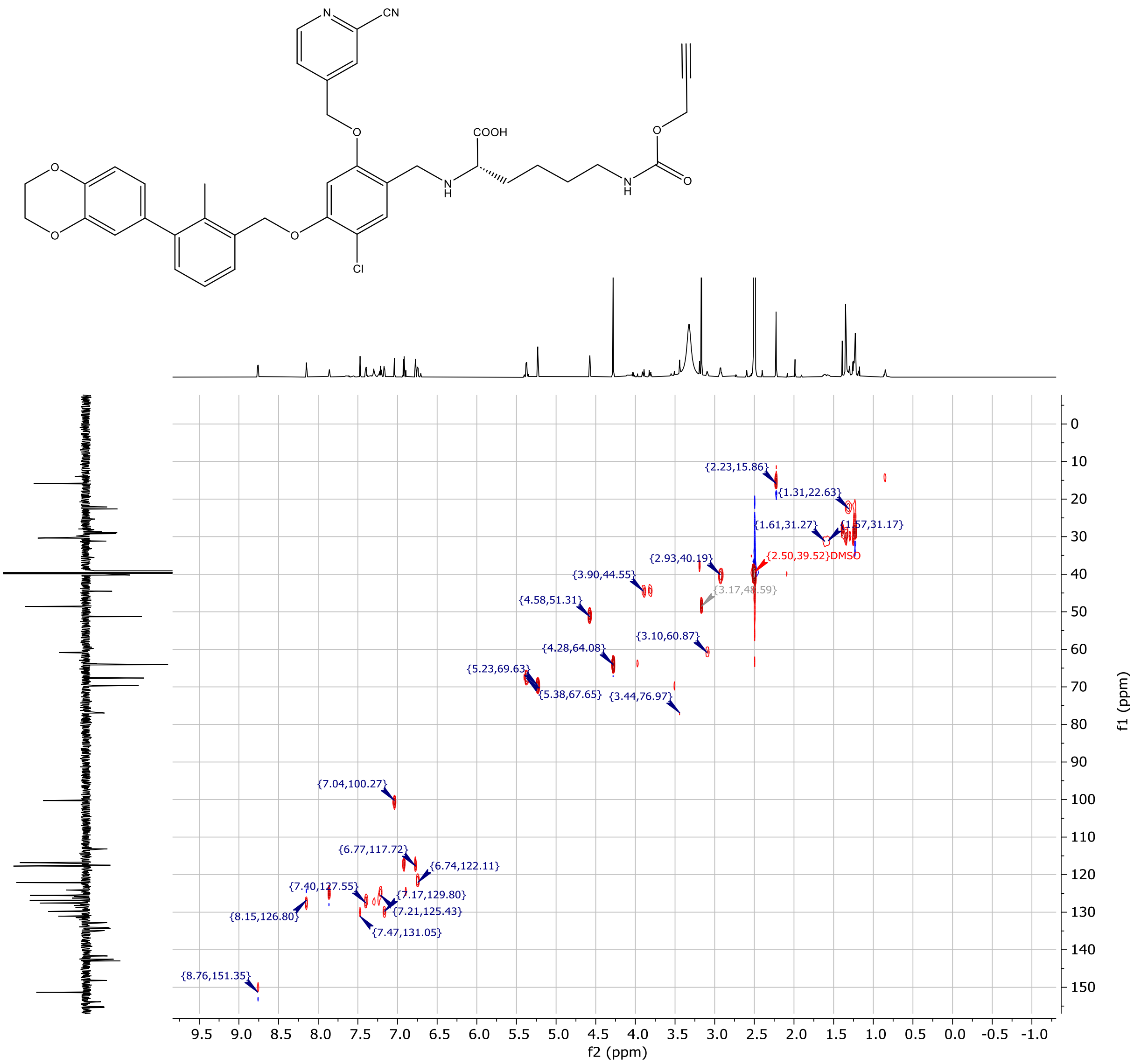

^{13}C-NMR (176 MHz, DMSO-d_6): δ 151.35, 131.05, 129.80, 127.55, 126.80, 125.54, 125.43, 122.11, 117.72, 116.81, 100.27, 76.97, 69.63, 67.65, 64.08, 60.87, 51.31, 44.55, 44.55, 40.19, 31.27, 31.17, 28.96, 22.63, 15.86.

^{1}H-NMR (700 MHz, DMSO-d_6): δ 8.76, 8.15, 7.86, 7.47, 7.40, 7.21, 7.17, 7.04, 6.92, 6.77, 6.74, 5.38, 5.23, 4.58, 4.28, 3.90, 3.82, 3.44, 3.10, 2.93, 2.23, 1.61, 1.57, 1.34, 1.31.

Figure S84: *N*-(2-((5-chloro-2-((3-cyanobenzyl)oxy)-4-((3-(2,3-dihydrobenzo[*b*][1,4]dioxin-6-yl)-2-methylbenzyl)oxy)benzyl)amino)ethyl)acetamide (**4j**):

^{1}H-NMR (600 MHz, $CDCl_3$)

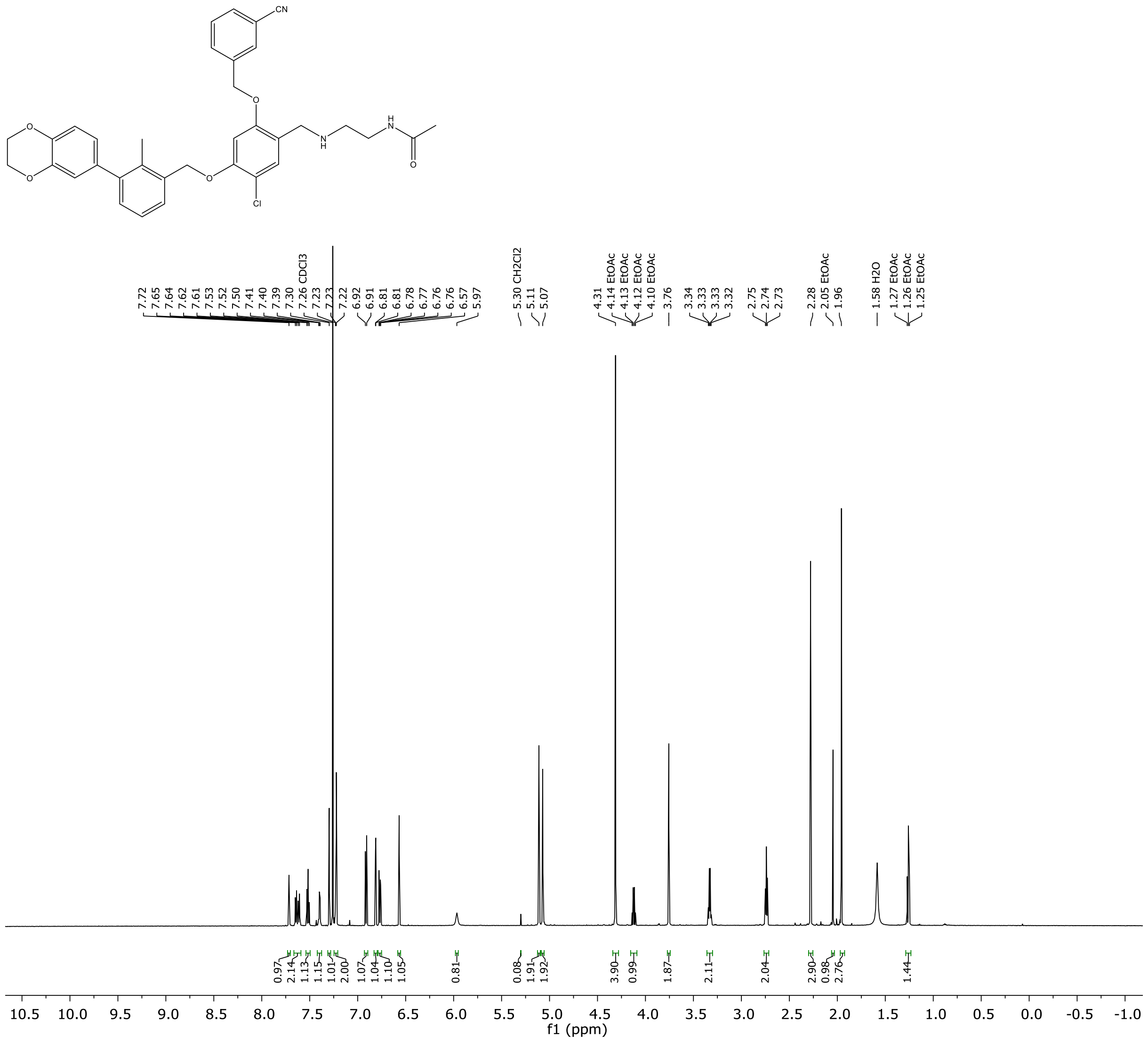

^{1}H-NMR (600 MHz, $CDCl_3$): δ 7.72 (s, 1H), 7.63 (m, 2H), 7.52 (t, *J* = 7.8 Hz, 1H), 7.40 (m, 1H), 7.30 (s, 1H), 7.23-7.22 (m, 2H), 6.91 (d, *J* = 8.2 Hz, 1H), 6.81 (d, *J* = 2.1 Hz, 1H), 6.77 (dd, *J* = 8.2 Hz, *J* = 2.1 Hz, 1H), 6.57 (s, 1H), 5.97 (br s, 1H), 5.11 (s, 2H), 5.07 (s, 2H), 4.31 (s, 4H), 3.76 (s, 2H), 3.33 (q, *J* = 5.9 Hz, 2H), 2.74 (t, *J* = 5.9 Hz, 2H), 2.28 (s, 3H), 1.96 (s, 3H).

Figure S85: *N*-(2-((5-chloro-2-((3-cyanobenzyl)oxy)-4-((3-(2,3-dihydrobenzo[*b*][1,4]dioxin-6-yl)-2-methylbenzyl)oxy)benzyl)amino)ethyl)acetamide (**4j**):

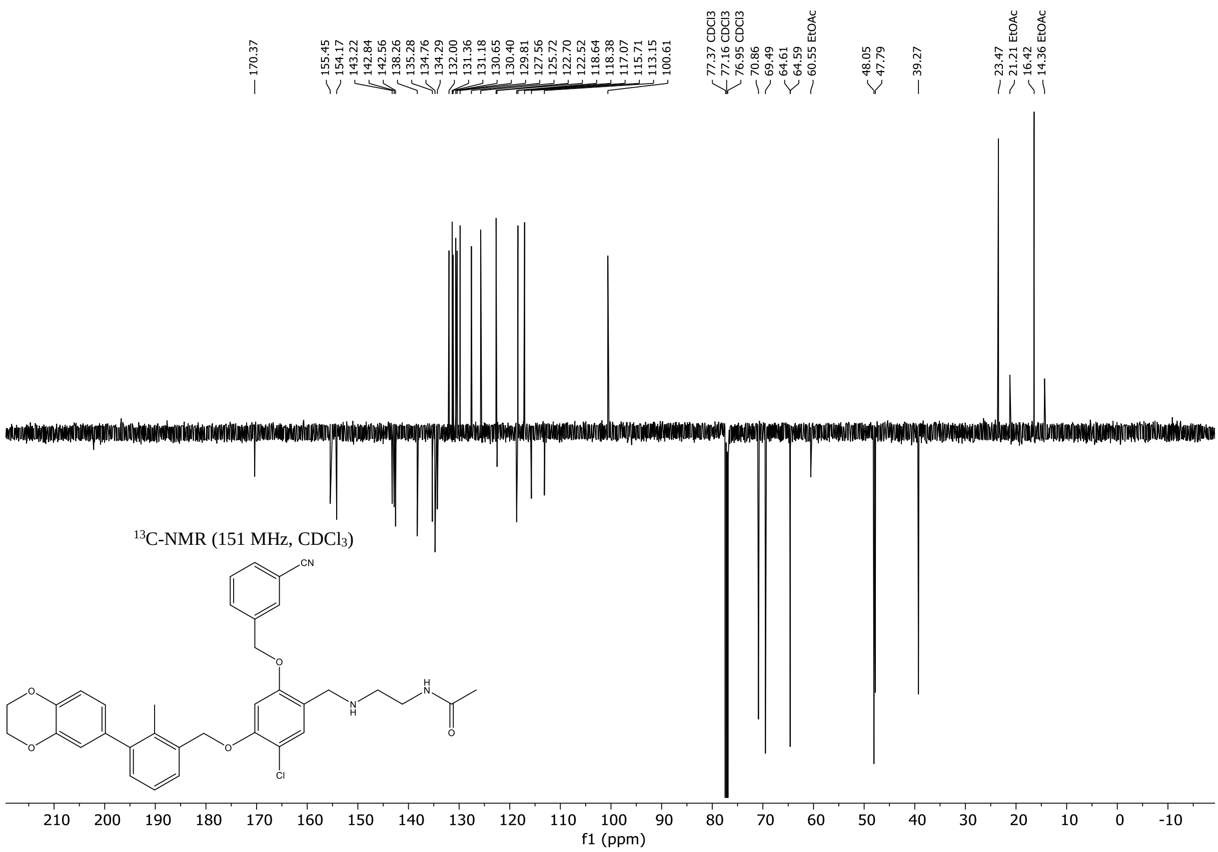

^{13}C-NMR (151 MHz, $CDCl_3$): δ 170.37, 155.45, 154.17, 143.22, 142.84, 142.56, 138.26, 135.28, 134.76, 134.29, 132.00, 131.36, 131.18, 130.65, 130.40, 129.81, 127.56, 125.72, 122.70, 122.52 118.64, 118.38, 117.07, 115.71, 113.15, 100.61, 70.86, 69.49, 64.61, 64.59, 48.05, 47.79, 39.27, 23.47, 16.42.

Figure S86: *N*-(2-((5-chloro-4-((3-(2,3-dihydrobenzo[*b*][1,4]dioxin-6-yl)-2-methylbenzyl)oxy)-2-(oxazol-4-ylmethoxy)benzyl)amino)ethyl)acetamide (**4k**):

^{1}H-NMR (600 MHz, $CDCl_3$)

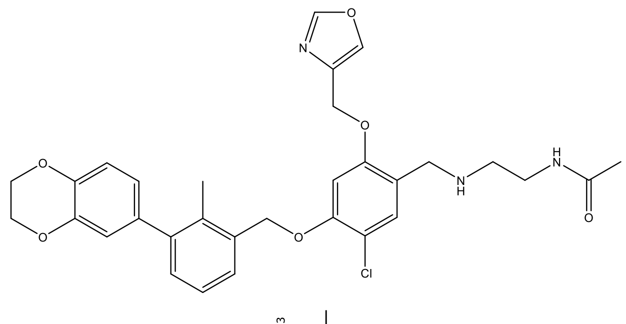

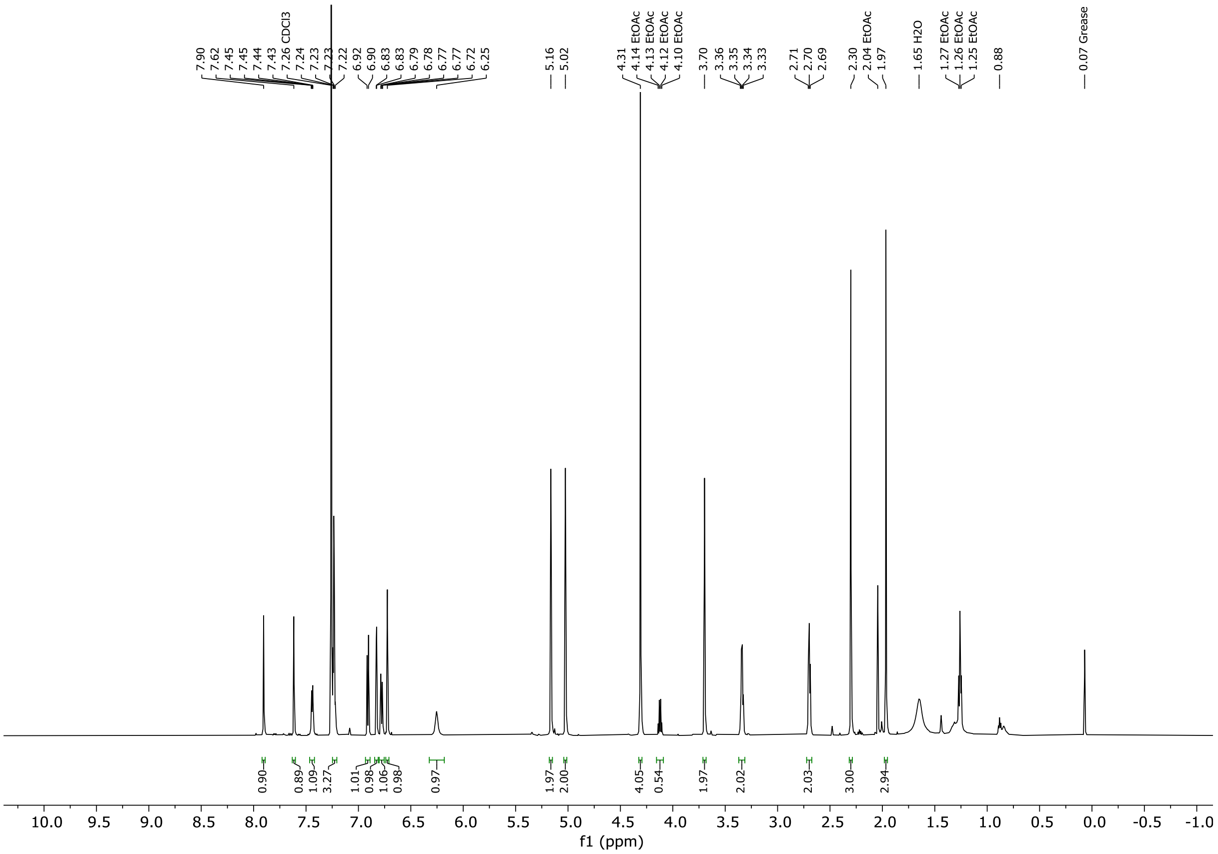

^{1}H-NMR (600 MHz, $CDCl_3$): δ 7.90 (s, 1H), 7.62 (s, 1H), 7.45 – 7.43 (m, 1H), 7.24 – 7.22 (m, 3H), 6.91 (d, *J* = 8.2 Hz, 1H), 6.83 (d, *J* = 2.1 Hz, 1H), 6.78 (dd, *J* = 8.2 Hz, *J* = 2.1 Hz, 1H), 6.72 (s, 1H), 6.25 (br s, 1H), 5.16 (s, 2H), 5.02 (s, 2H), 4.31 (s, 4H), 3.70 (s, 2H), 3.33 (q, *J* = 5.7 Hz, 2H), 2.70 (t, *J* = 5.7 Hz), 2.30 (s, 3H), 1.97 (s, 1H).

Figure S87: *N*-(2-((5-chloro-4-((3-(2,3-dihydrobenzo[*b*][1,4]dioxin-6-yl)-2-methylbenzyl)oxy)-2-(oxazol-4-ylmethoxy)benzyl)amino)ethyl)acetamide (**4k**):

^{1}H-NMR (151 MHz, $CDCl_3$)

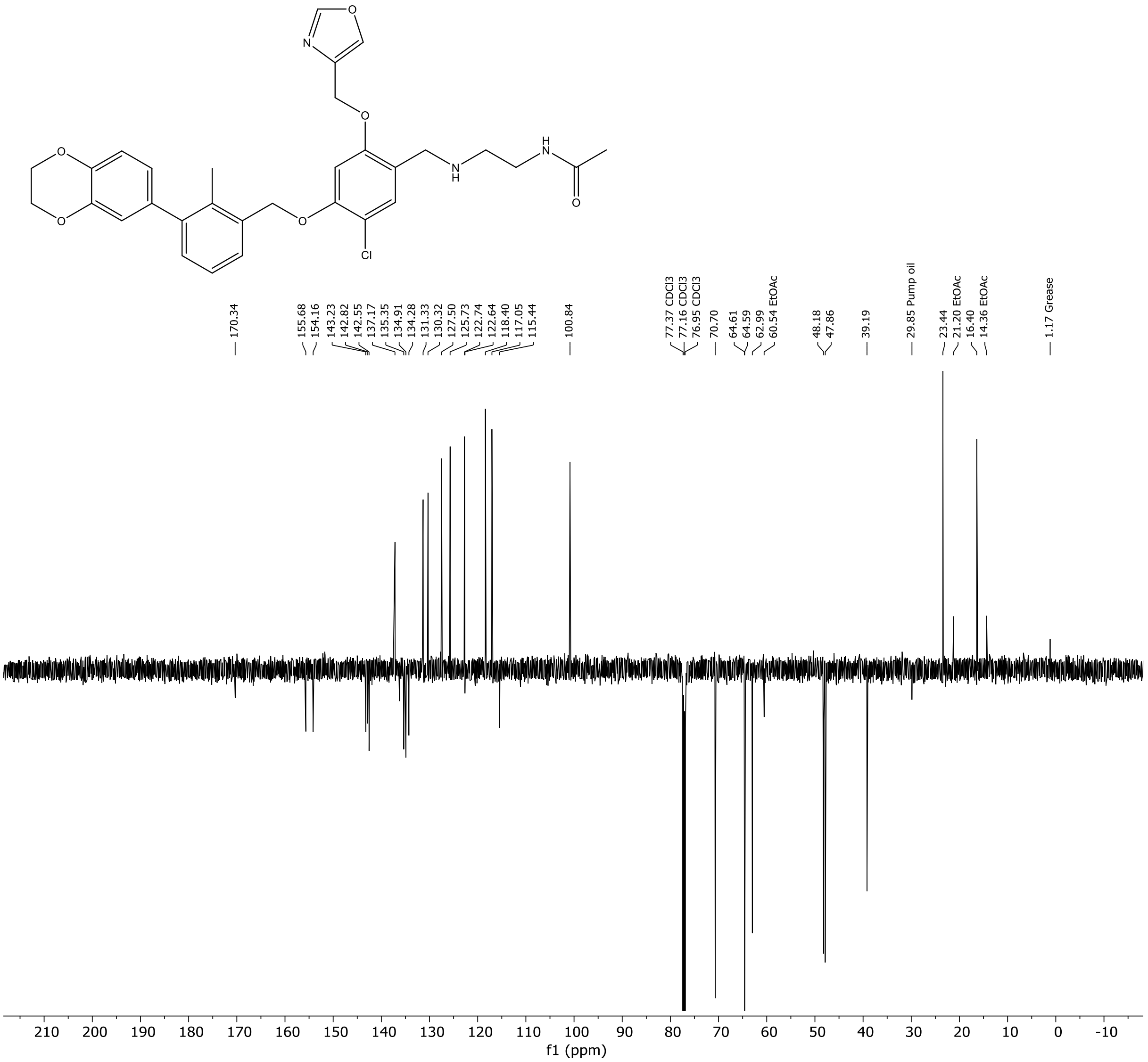

^{13}C-NMR (151 MHz, $CDCl_3$): δ 170.34, 155.68, 154.16, 143.23, 142.82, 142.55, 137.17, 135.35, 134.91, 134.28, 131.33, 130.32, 127.50, 125.73, 122.74, 122.64, 118.40, 117.05, 115.44, 100.84, 70.70, 64.61, 64.59, 62.99, 48.18, 47.86, 39.19, 23.44, 16.40.

Figure S88: (*S*)-1-(5-chloro-4-((3-(2,3-dihydrobenzo[*b*][1,4]dioxin-6-yl)-2-methylbenzyl)oxy)-2-(oxazol-4-ylmethoxy)benzyl)piperidine-2-carboxylic acid (**4l**):

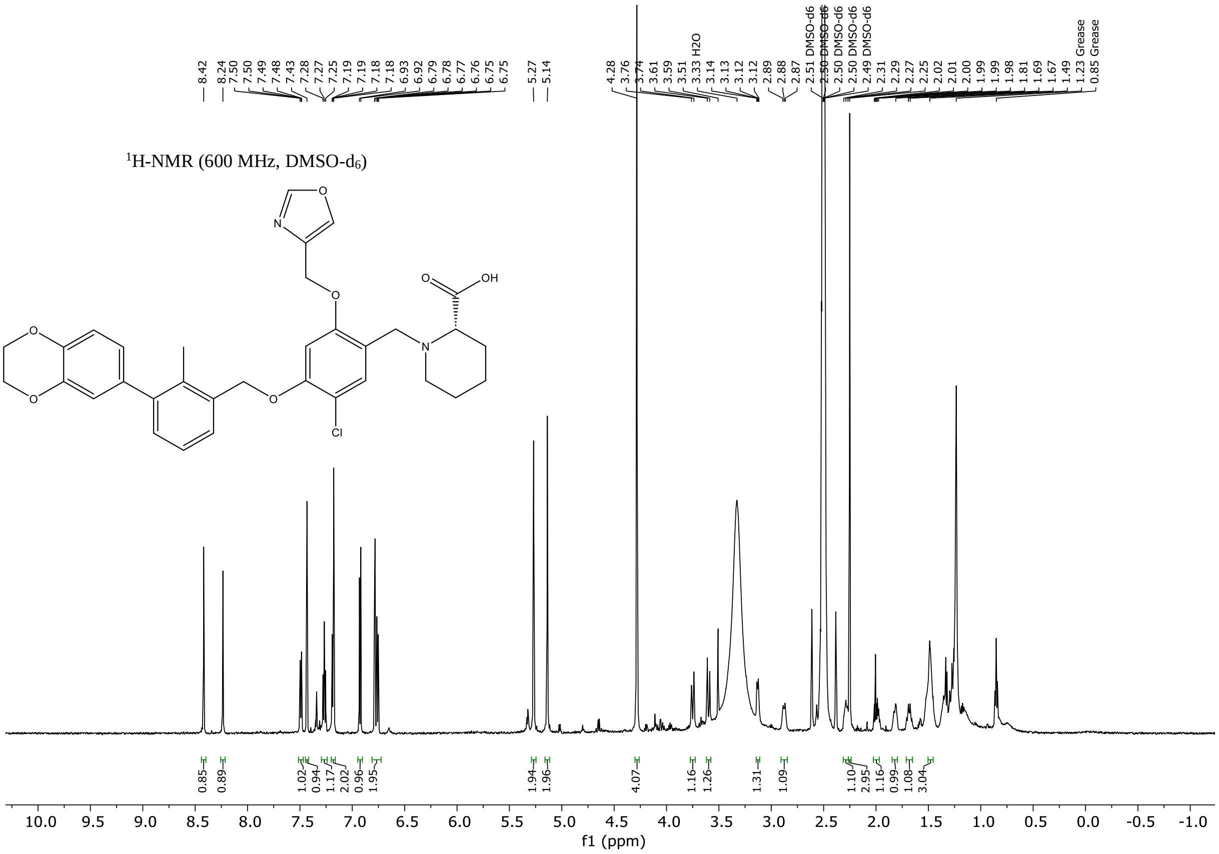

^{1}H-NMR (600 MHz, DMSO-d$_6$): δ 8.42 (s, 1H), 8.24 (s, 1H), 7.49 (dd, *J* = 7.6 Hz, *J* = 1.5 Hz, 1H), 7.43 (s, 1H), 7.27 (t, *J* = 7.6 Hz, 1H), 7.18 (dd, *J* = 7.6 Hz, *J* = 1.5 Hz, 1H), 7.18 (s, 1H), 6.92 (d, *J* = 8.2 Hz, 1H), 6.78 (d, *J* = 2.1 Hz, 1H), 6.76 (dd, *J* = 8.2 Hz, *J* = 2.1 Hz, 1H), 5.27 (s, 2H), 5.14 (s, 2H), 4.28 (s, 4H), 3.74 (d, *J* = 14 Hz, 1H), 3.60 (d, *J* = 14 Hz, 1H), 3.12 (m, 1H), 2.88 (m, 1H), 2.29 (m, 1H), 2.25 (s, 3H), 2.00 (m, 1H), 1.81 (m, 1H), 1.68 (m, 1H), 1.49 (m, 3H).

Figure S89: (*S*)-1-(5-chloro-4-((3-(2,3-dihydrobenzo[*b*][1,4]dioxin-6-yl)-2-methylbenzyl)oxy)-2-(oxazol-4-ylmethoxy)benzyl)piperidine-2-carboxylic acid (**4l**):

^{13}C-NMR (151 MHz, DMSO-d$_6$)

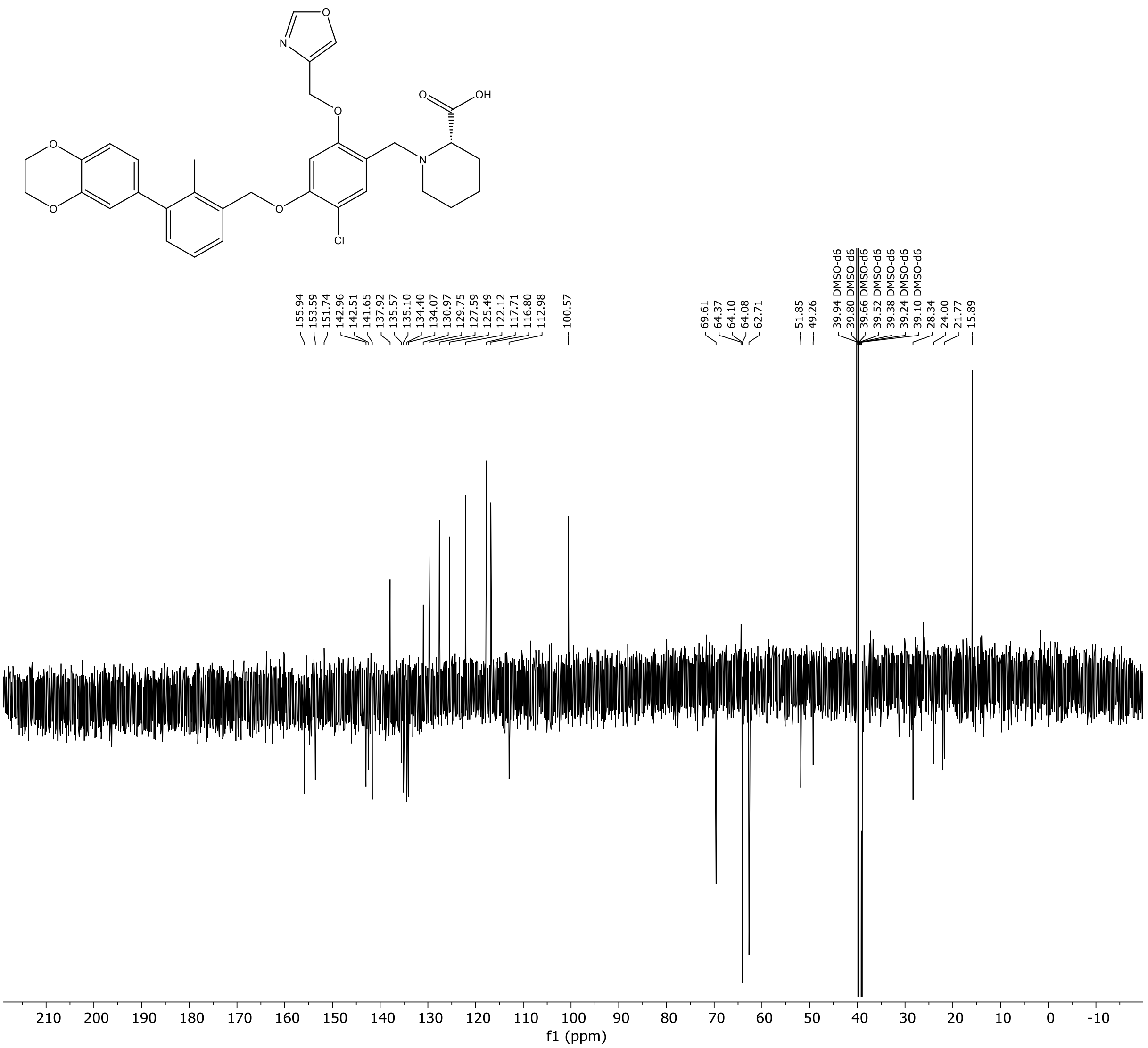

^{13}C-NMR (151 MHz, DMSO-d$_6$): δ 155.94, 153.59, 151.74, 142.96, 142.51, 141.65, 137.92, 135.57, 135.10, 134.40, 134.07, 130.97, 129.75, 127.59, 125.49, 122.12, 117.71, 116.80, 112.98, 100.57, 69.61, 64.37, 64.10, 64.08, 62.71, 51.85, 49.26, 28.34, 24.00, 21.77, 15.89.

Figure S90: (*S*)-1-(5-chloro-4-((3-(2,3-dihydrobenzo[*b*][1,4]dioxin-6-yl)-2-methylbenzyl)oxy)-2-(oxazol-4-ylmethoxy)benzyl)piperidine-2-carboxylic acid (**4l**):

^{1}H-^{1}H COSY NMR (600 MHz, DMSO-d_6)

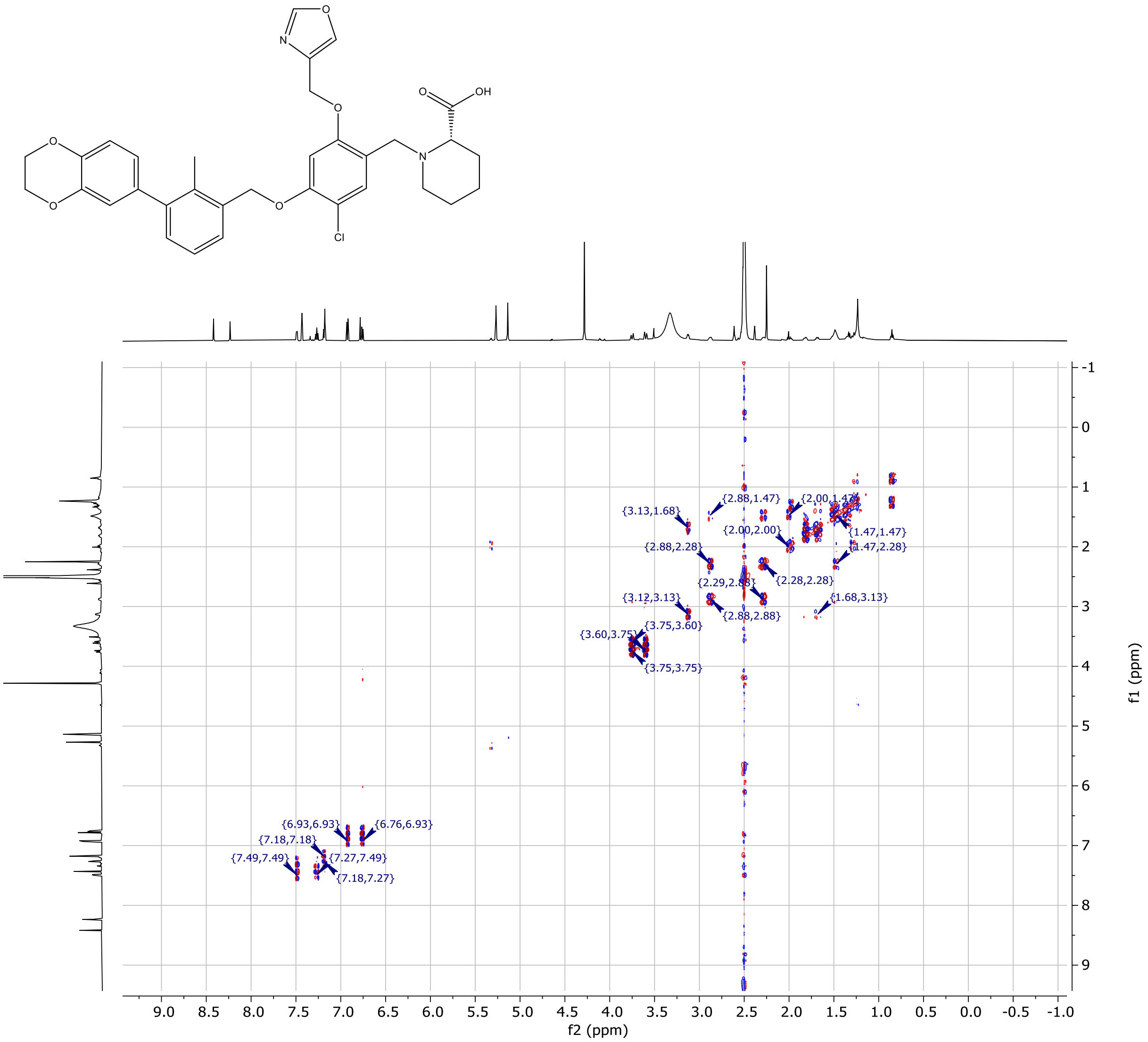

^{1}H-NMR (600 MHz, DMSO-d_6): δ 7.49, 7.49, 7.27, 7.27, 7.27, 7.18, 7.18, 6.93, 6.93, 6.76, 6.76, 3.75, 3.75, 3.60, 3.60, 3.13, 3.13, 2.88, 2.88, 2.28, 2.28, 2.28, 2.00, 1.82, 1.81, 1.68, 1.68, 1.68, 1.47, 1.47, 1.47, 1.47.

^{1}H-NMR (600 MHz, DMSO-d_6): δ 7.49, 7.49, 7.27, 7.27, 7.27, 7.18, 7.18, 6.93, 6.93, 6.76, 6.76, 3.75, 3.75, 3.60, 3.60, 3.13, 3.12, 2.88, 2.88, 2.88, 2.29, 2.29, 2.28, 2.00, 2.00, 1.81, 1.81, 1.68, 1.68, 1.68, 1.47, 1.47.

Figure S91: (*S*)-1-(5-chloro-4-((3-(2,3-dihydrobenzo[*b*][1,4]dioxin-6-yl)-2-methylbenzyl)oxy)-2-(oxazol-4-ylmethoxy)benzyl)piperidine-2-carboxylic acid (**4l**):

^{1}H-^{13}C HSQC NMR (600 MHz, 151 MHz, DMSO-d$_6$)

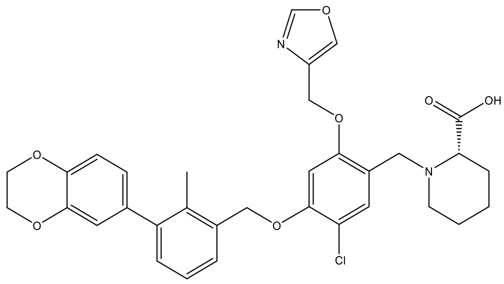

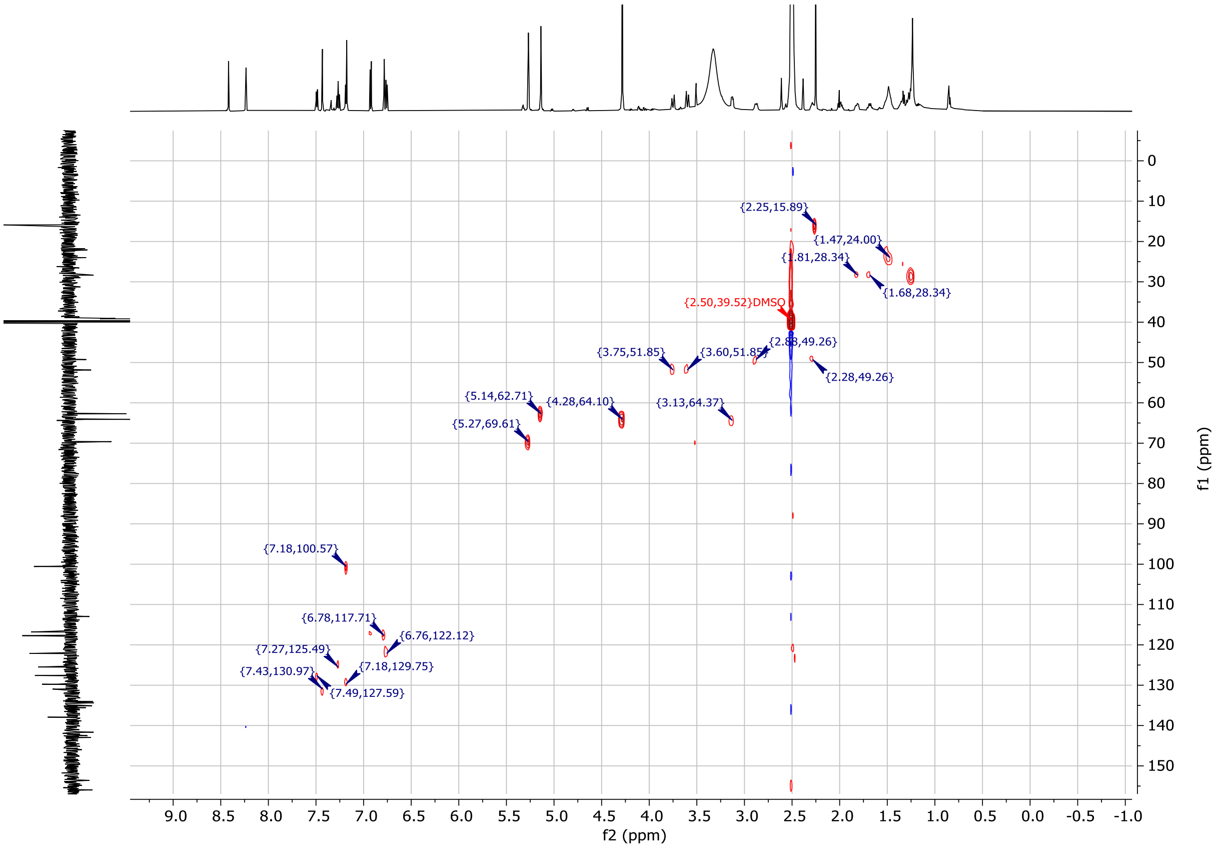

^{13}C-NMR (151 MHz, DMSO-d$_6$): δ 130.97, 129.75, 127.59, 125.49, 122.12, 117.71, 116.80, 100.57, 69.61, 64.37, 64.10, 62.71, 51.85, 51.85, 49.26, 49.26, 28.34, 28.34, 24.00, 15.89.

^{1}H-NMR (600 MHz, DMSO-d$_6$): δ 7.49, 7.43, 7.27, 7.18, 7.18, 6.92, 6.78, 6.76, 5.27, 5.14, 4.28, 3.75, 3.60, 3.13, 2.88, 2.28, 2.25, 1.81, 1.68, 1.47.

Figure S92: (5-Chloro-2-((2-cyanopyridin-4-yl)methoxy)-4-((2-methyl-3-(1*H*-pyrrol-1-yl)benzyl)oxy)benzyl)-*D*-serine (**4m**):

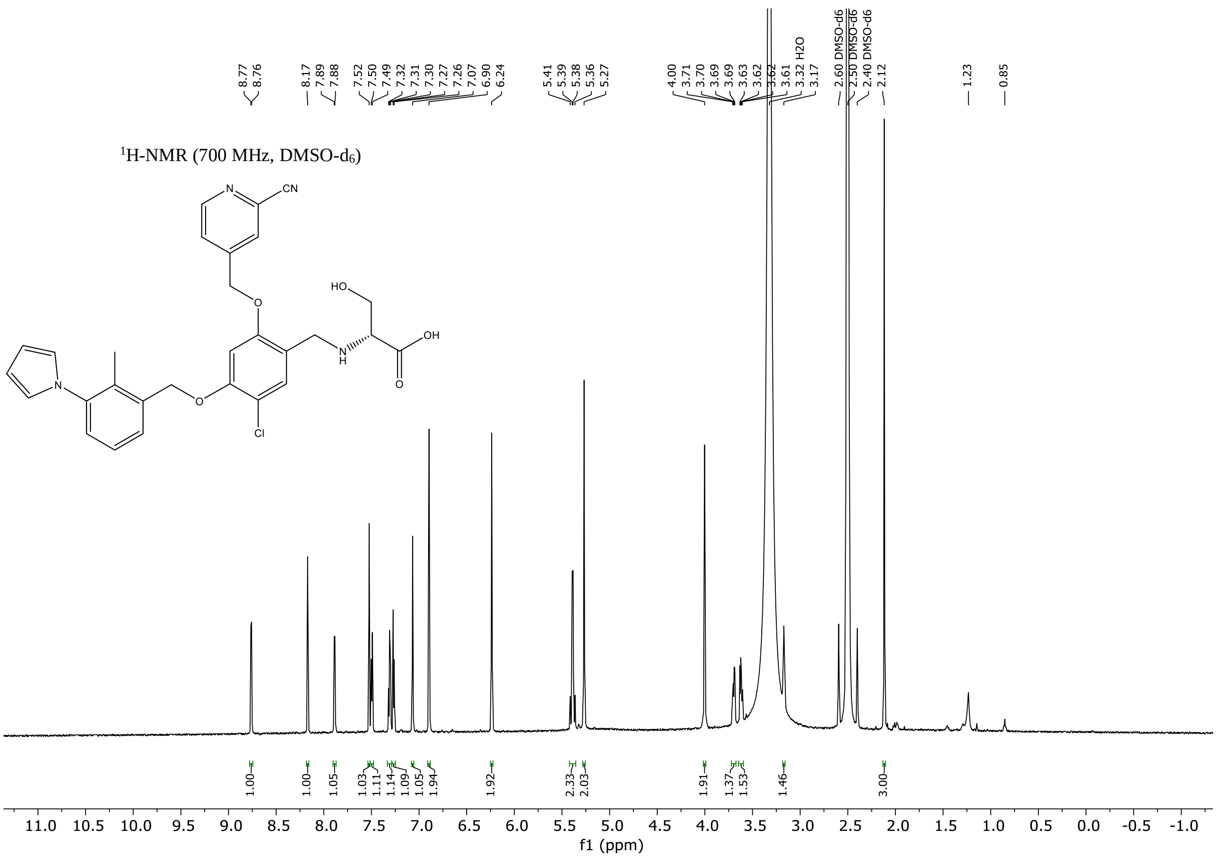

^{1}H-NMR (700 MHz, DMSO-d$_6$): δ 8.76 (d, J = 4.9 Hz, 1H), 8.17 (s, 1H), 7.89 (d, J = 4.9 Hz, 1H), 7.52 (s, 1H), 7.50 (d, J = 7.9 Hz, 1H), 7.31 (t, J = 7.9 Hz, 1H), 7.27 (d, J = 7.9 Hz, 1H), 7.07 (s, 1H), 6.90 (s, 2H), 6.24 (s, 2H), 5.39 (m, 2H), 5.27 (s, 2H), 4.00 (s, 2H), 3.71 – 3.61 (m, 2H), 3.17 (m, 1H), 2.12 (s, 3H).

Figure S93: 4-((4-Chloro-2-(((2-hydroxyethyl)amino)methyl)-5-((2-methyl-3-(1*H*-pyrrol-1-yl)benzyl)oxy)phenoxy)methyl)picolinonitrile (**4n**):

^{1}H-NMR (700 MHz, $CDCl_3$)

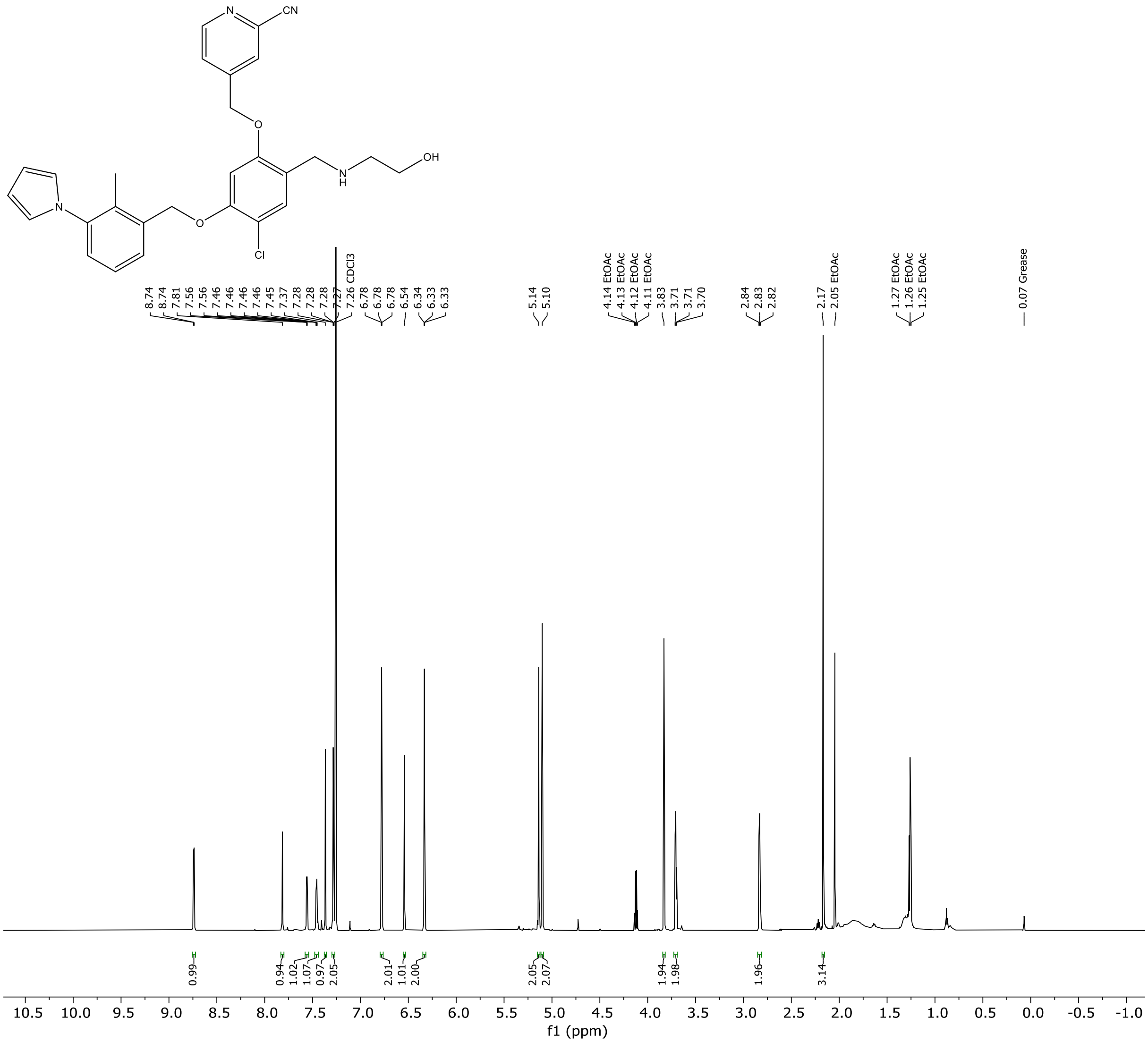

^{1}H-NMR (700 MHz, $CDCl_3$): δ 8.74 (d, *J* = 5.0 Hz, 1H), 7.81 (s, 1H), 7.56 (d, *J* = 5.0 Hz, 1H), 7.46 – 7.45 (m, 2H), 7.37 (s, 1H), 7.28 – 7.27 (m, 2H), 6.78 (t, *J* = 2.1 Hz, 2H), 6.54 (s, 1H), 6.33 (t, *J* = 2.1 Hz, 2H), 5.14 (s, 2H), 5.10 (s, 2H), 3.83 (s, 2H), 3.71 (t, *J* = 5.2 Hz, 2H), 2.83 (t, *J* = 5.2 Hz, 2H), 2.17 (s, 3H).

Figure S94: 4-((4-Chloro-2-(((2-hydroxyethyl)amino)methyl)-5-((2-methyl-3-(1*H*-pyrrol-1-yl)benzyl)oxy)phenoxy)methyl)picolinonitrile (**4n**):

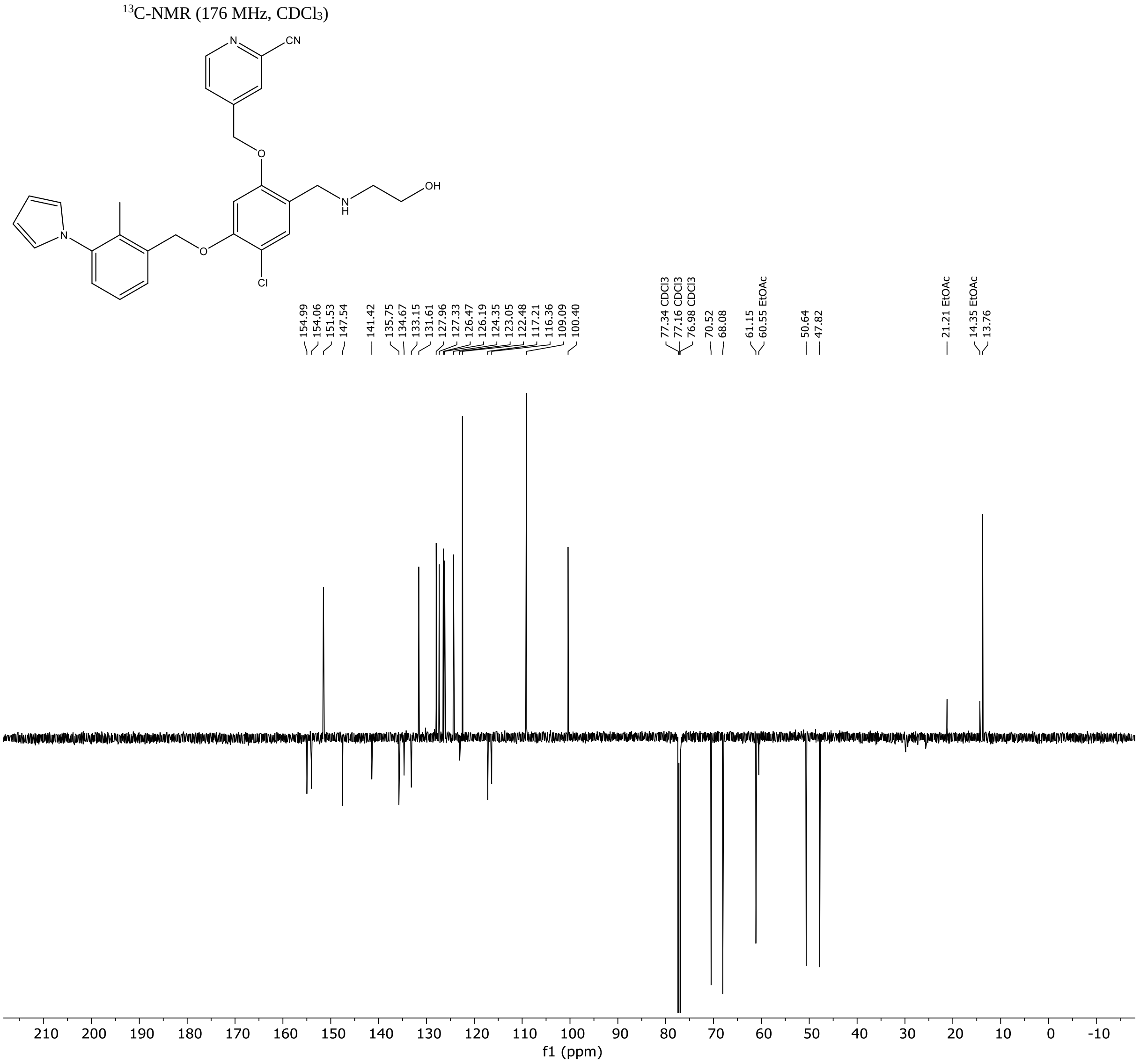

^{13}C-NMR (176 MHz, $CDCl_3$): δ 154.99, 154.06, 151.53, 147.54, 141.42, 135.75, 134.67, 133.15, 131.61, 127.96, 127.33, 126.47, 126.19, 124.35, 123.05, 122.48, 117.21, 116.36, 109.09, 100.40, 70.52, 68.08, 61.15, 50.64, 47.82, 13.76.

Figure S95: 4-((4-Chloro-2-(((2-hydroxyethyl)amino)methyl)-5-((2-methyl-3-(1*H*-pyrrol-1-yl)benzyl)oxy)phenoxy)methyl)picolinonitrile (**4n**):

^{1}H-^{1}H COSY NMR (700 MHz, $CDCl_3$)

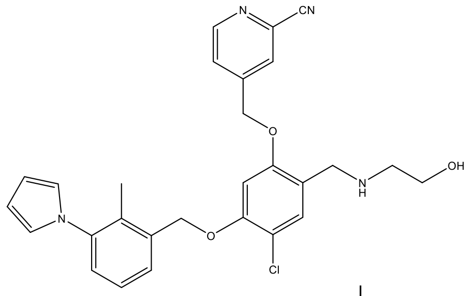

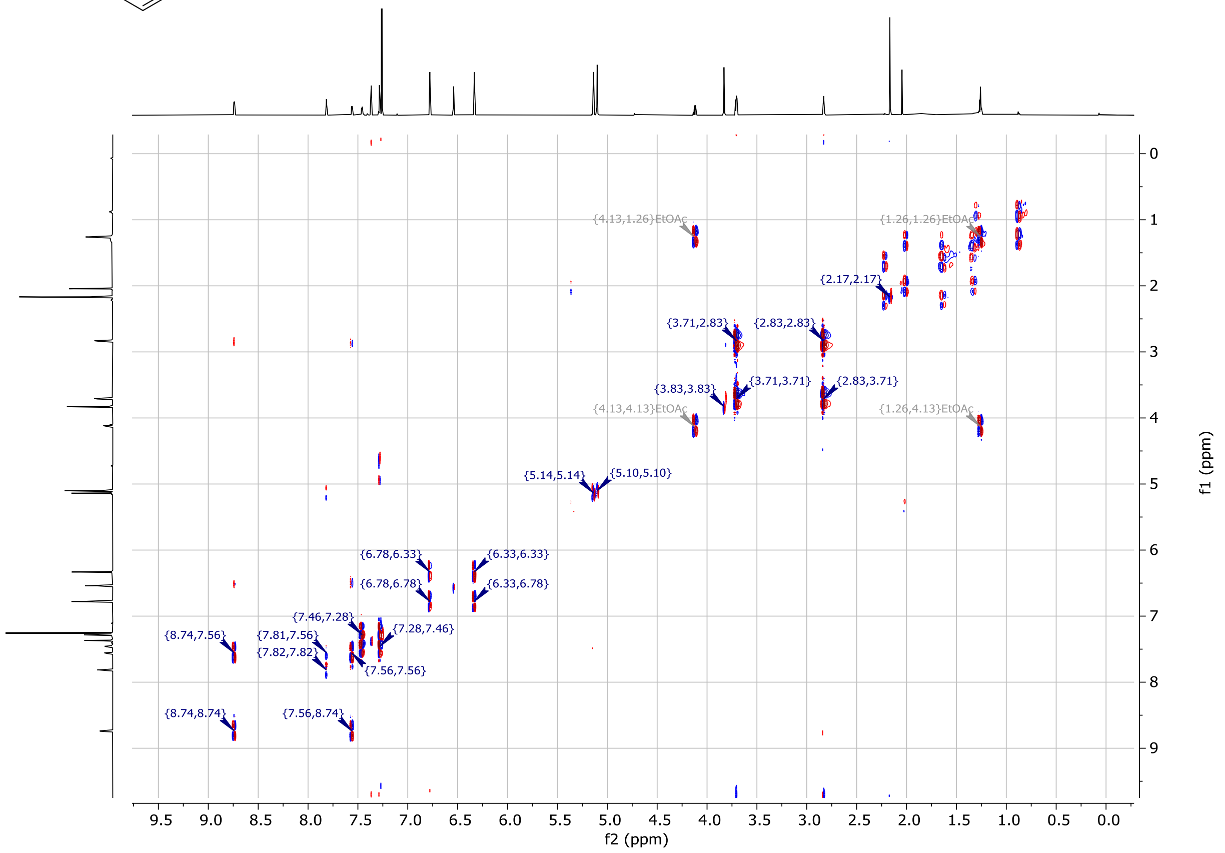

^{1}H-NMR (700 MHz, $CDCl_3$): δ 8.74, 8.74, 7.82, 7.56, 7.56, 7.56, 7.46, 7.46, 7.37, 7.28, 7.28, 6.78, 6.78, 6.54, 6.33, 6.33, 5.14, 5.10, 3.83, 3.71, 3.71, 2.83, 2.83, 2.17.

^{1}H-NMR (700 MHz, $CDCl_3$): δ 8.74, 8.74, 7.82, 7.81, 7.56, 7.56, 7.46, 7.46, 7.37, 7.28, 7.28, 6.78, 6.78, 6.54, 6.33, 6.33, 5.14, 5.10, 3.83, 3.71, 3.71, 2.83, 2.83, 2.17.

Figure S96: 4-((4-Chloro-2-(((2-hydroxyethyl)amino)methyl)-5-((2-methyl-3-(1*H*-pyrrol-1-yl)benzyl)oxy)phenoxy)methyl)picolinonitrile (**4n**):

^{1}H-^{13}C HSQC NMR (700 MHz, 176 MHz, $CDCl_3$)

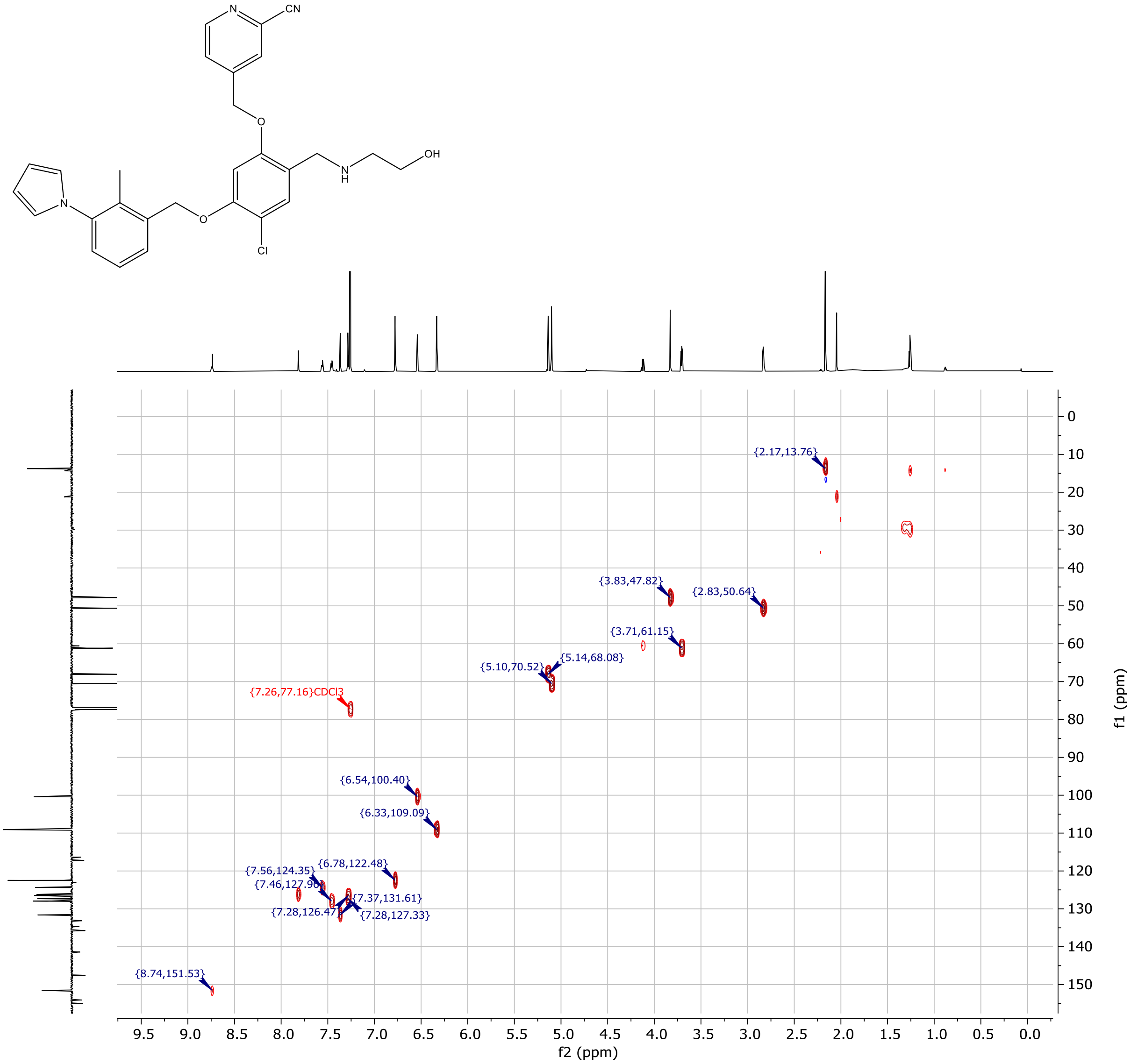

^{13}C-NMR (176 MHz, $CDCl_3$): δ 151.53, 131.61, 127.96, 127.33, 126.47, 126.19, 124.35, 122.48, 109.09, 100.40, 70.52, 68.08, 61.15, 50.64, 47.82, 13.76.

^{1}H-NMR (700 MHz, $CDCl_3$): δ 8.74, 7.81, 7.56, 7.46, 7.37, 7.28, 7.28, 6.78, 6.54, 6.33, 5.14, 5.10, 3.83, 3.71, 2.83, 2.17.

Figure S97: (*S*)-1-(5-chloro-2-((2-cyanopyridin-4-yl)methoxy)-4-((2-methyl-3-(1*H*-pyrrol-1-yl)benzyl)oxy)benzyl)piperidine-2-carboxylic acid (**4o**):

^{1}H-NMR (600 MHz, $CDCl_3$)

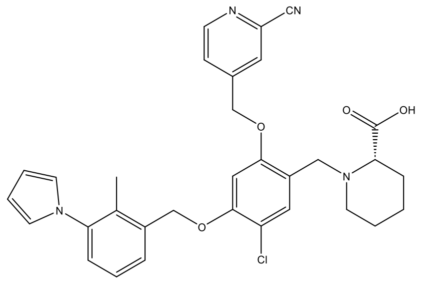

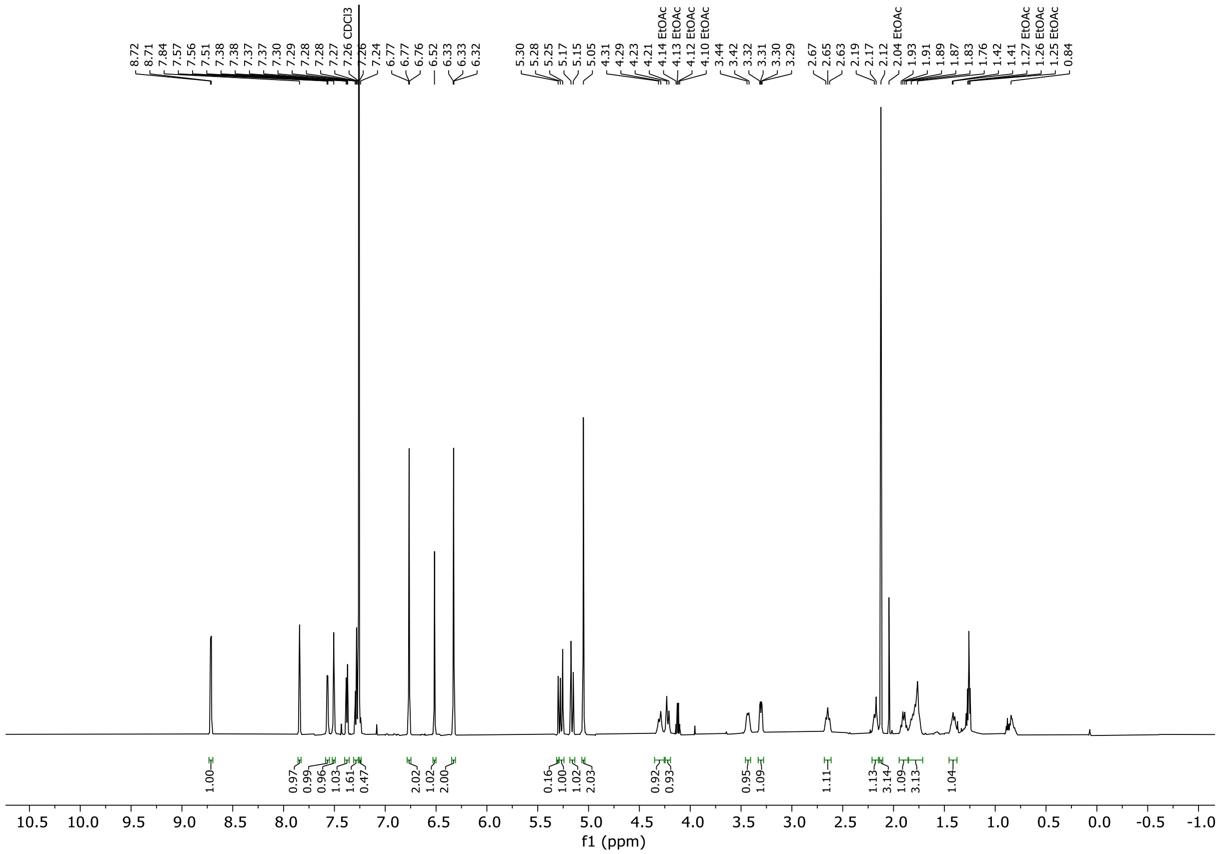

^{1}H-NMR (600 MHz, $CDCl_3$): δ 8.71 (d, *J* = 5.0 Hz, 1H), 7.84 (s, 1H), 7.57 (d, *J* = 5.0 Hz, 1H), 7.51 (s, 1H), 7.38 (dd, *J* = 7.6 Hz, *J* = 1.6 Hz 1H), 7.29 (dd, *J* = 7.6, *J* = 1.6 Hz, 1H), 7.26 (t, *J* = 7.6 Hz, 1H), 6.77 (t, *J* = 2.1 Hz, 1H), 6.52 (s, 1H), 6.33 (t, *J* = 2.1 Hz, 1H), 5.26 (d, *J* = 13 Hz, 1H), 5.16 (d, *J* = 13 Hz, 1H), 5.05 (s, 1H), 4.30 (d, *J* = 13 Hz, 1H), 4.22 (d, *J* = 13 Hz, 1H), 3.43 (d, *J* = 9.8 Hz, 1H), 3.30 (dd, *J* = 9.8 Hz, *J* = 3.9 Hz, 1H), 2.65 (t, *J* = 11 Hz, 1H), 2.19 – 2.17 (m, 1H), 2.12 (s, 3H), 1.93 – 1.87 (m, 1H), 1.83 – 1.76 (m, 3H), 1.42 – 1.41 (m, 1H).

Figure S98: (*S*)-1-(5-chloro-2-((2-cyanopyridin-4-yl)methoxy)-4-((2-methyl-3-(1*H*-pyrrol-1-yl)benzyl)oxy)benzyl)piperidine-2-carboxylic acid (**4o**):

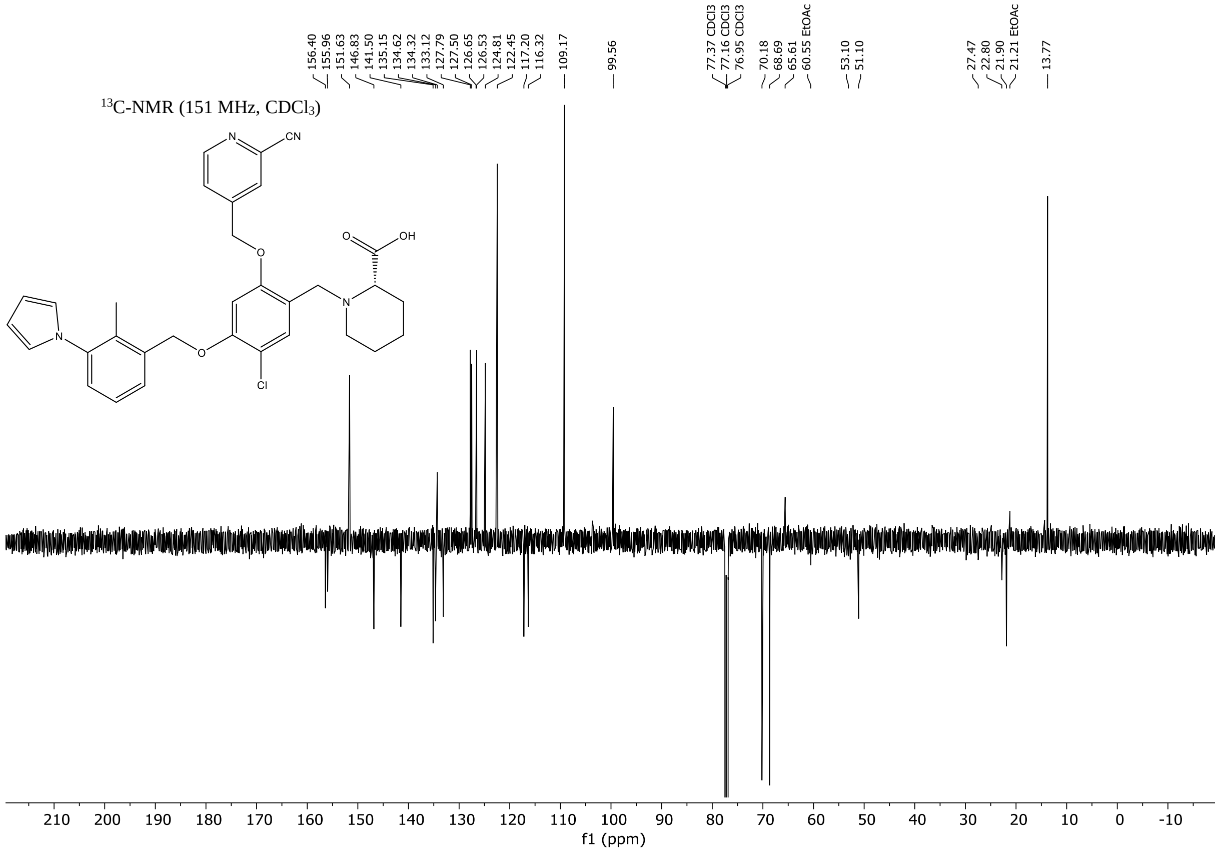

^{13}C-NMR (151 MHz, CDCl$_3$): δ 156.40, 155.96, 151.63, 146.83, 141.50, 135.15, 134.62, 134.32, 133.12, 127.79, 127.50, 126.65, 126.53, 124.81, 122.45, 117.20, 116.32, 109.17, 99.56, 70.18, 68.69, 65.61, 53.10, 51.10, 27.47, 22.80, 21.90, 13.77.

Figure S99: (*S*)-1-(5-chloro-2-((2-cyanopyridin-4-yl)methoxy)-4-((2-methyl-3-(1*H*-pyrrol-1-yl)benzyl)oxy)benzyl)piperidine-2-carboxylic acid (**4o**):

^{1}H-^{1}H COSY NMR (600 MHz, $CDCl_3$)

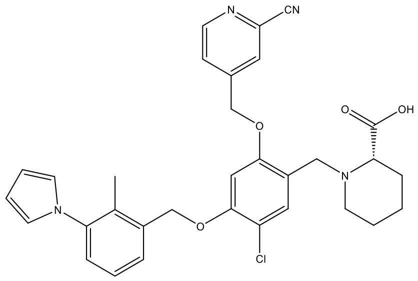

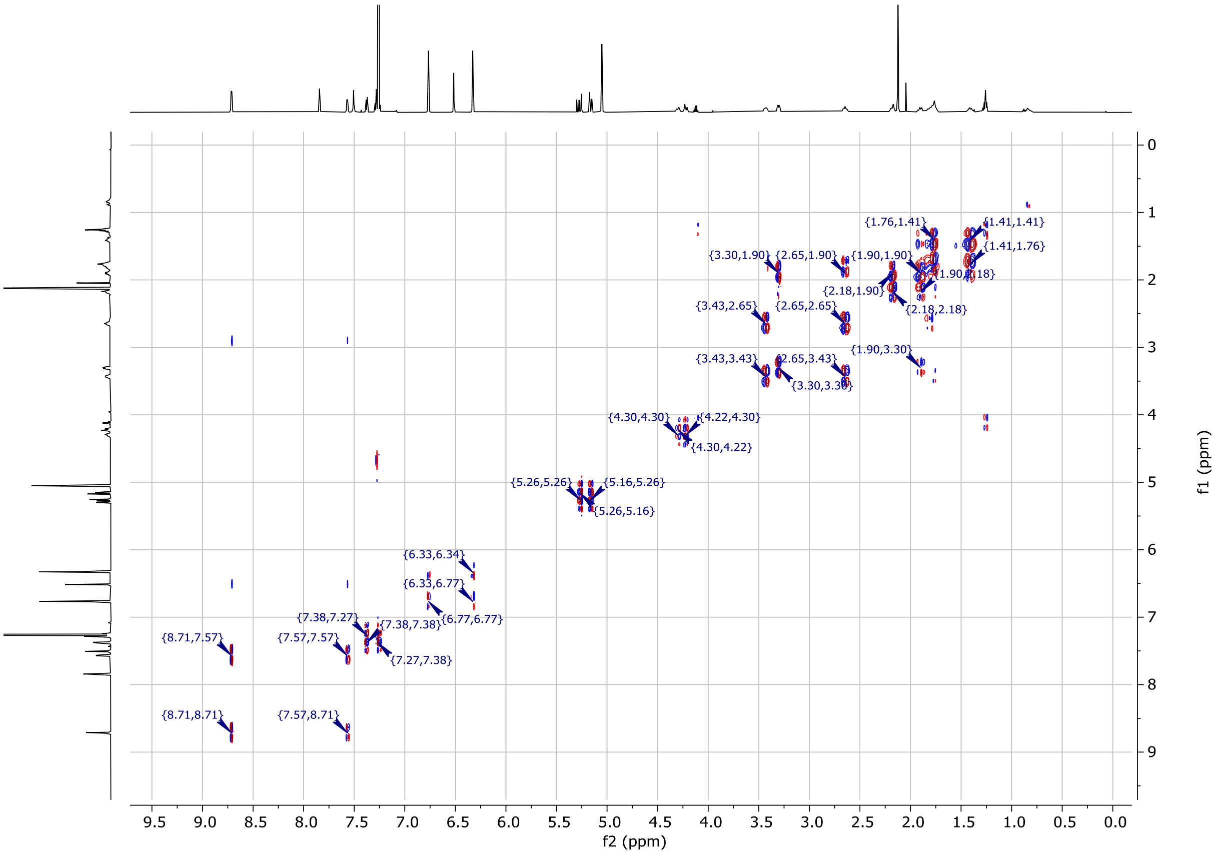

^{1}H-NMR (600 MHz, $CDCl_3$): δ 8.71, 8.71, 7.57, 7.57, 7.38, 7.38, 7.27, 7.27, 6.77, 6.77, 6.34, 6.33, 5.26, 5.26, 5.16, 5.16, 4.30, 4.30, 4.22, 4.22, 3.43, 3.43, 3.30, 3.30, 2.65, 2.65, 2.18, 2.18, 1.90, 1.90, 1.90, 1.90, 1.76, 1.76, 1.41, 1.41

^{1}H-NMR (600 MHz, $CDCl_3$): δ 8.71, 8.71, 7.57, 7.57, 7.38, 7.38, 7.27, 7.27, 6.77, 6.77, 6.33, 6.33, 5.26, 5.26, 5.16, 5.16, 4.30, 4.30, 4.22, 4.22, 3.43, 3.43, 3.30, 3.30, 2.65, 2.65, 2.65, 2.18, 2.18, 1.90, 1.90, 1.90, 1.76, 1.76, 1.41, 1.41.

Figure S100: (*S*)-1-(5-chloro-2-((2-cyanopyridin-4-yl)methoxy)-4-((2-methyl-3-(1*H*-pyrrol-1-yl)benzyl)oxy)benzyl)piperidine-2-carboxylic acid (**4o**):

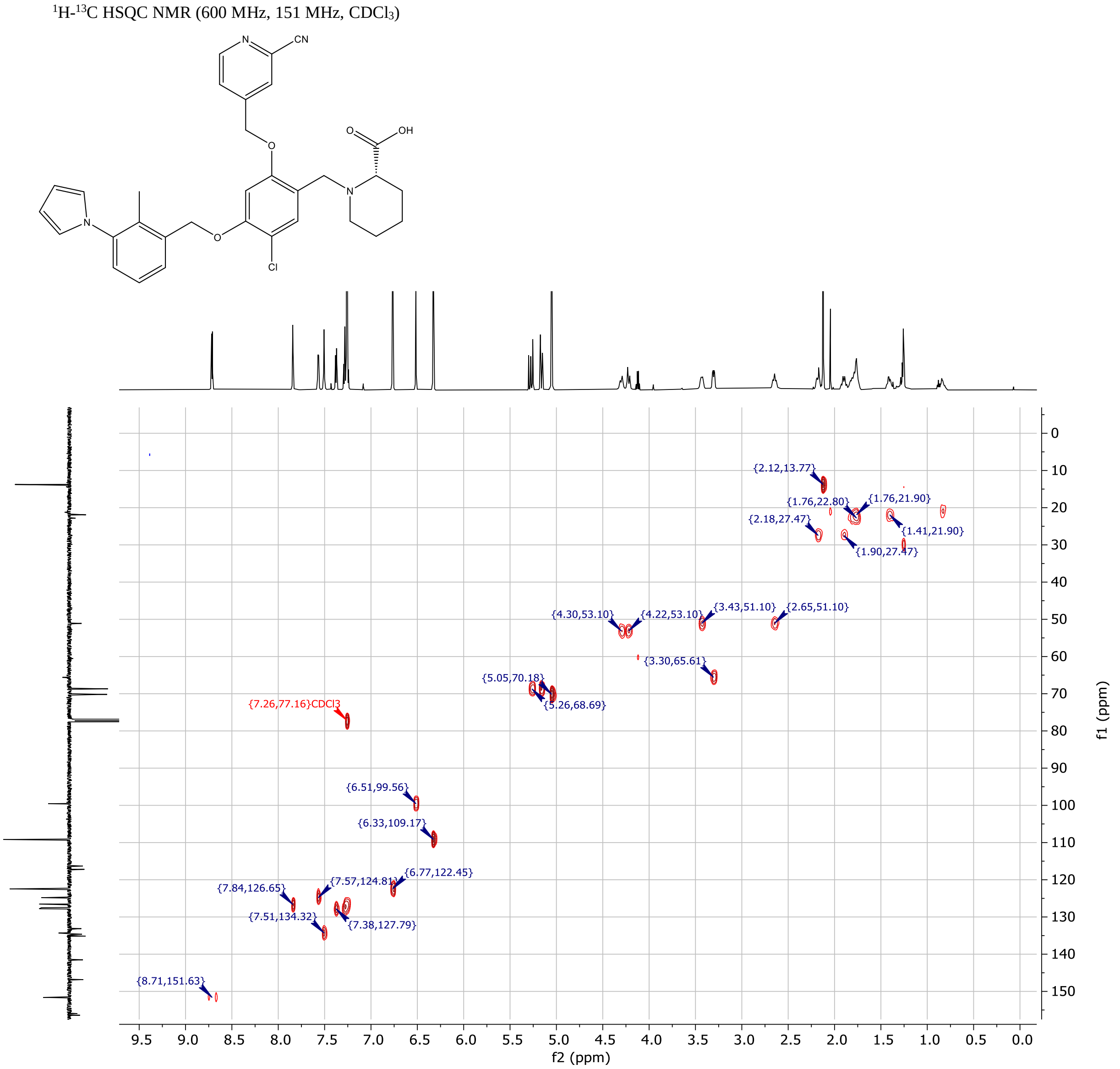

^{13}C-NMR (151 MHz, $CDCl_3$): δ 151.63, 134.32, 127.79, 127.50, 126.65, 126.53, 124.81, 122.45, 109.17, 99.56, 70.18, 68.69, 68.69, 65.61, 53.10, 53.10, 51.10, 51.10, 27.47, 27.47, 22.80, 21.90, 21.90, 13.77.
^1H-NMR (600 MHz, $CDCl_3$): δ 8.71, 7.84, 7.57, 7.51, 7.38, 7.27, 7.27, 6.77, 6.51, 6.33, 5.26, 5.16, 5.05, 4.30, 4.22, 3.43, 3.30, 2.65, 2.18, 2.12, 1.90, 1.76, 1.76, 1.41.

Figure S101: *N*-(5-chloro-2-((2-cyanopyridin-4-yl)methoxy)-4-((3-(2,3-dihydrobenzo[*b*][1,4]dioxin-6-yl)-2-methylbenzyl)oxy)benzyl)-*N*-methyl-*D*-serine (**5a**):

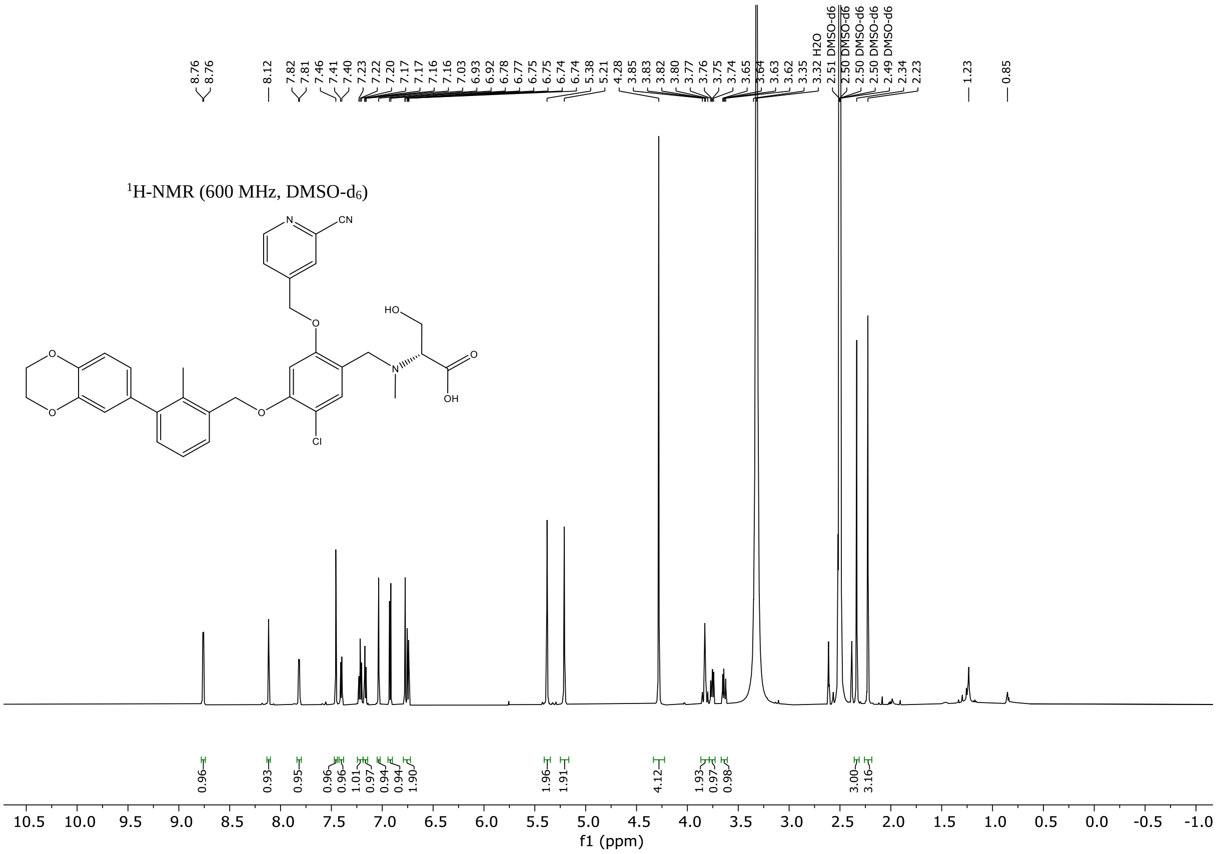

^{1}H-NMR (600 MHz, DMSO-d$_6$): δ 8.76 (d, *J* = 5.1 Hz, 1H), 8.12 (s, 1H), 7.82 (d, *J* = 5.1 Hz, 1H), 7.46 (s, 1H), 7.40 (d, *J* = 7.6 Hz, 1H), 7.22 (t, *J* = 7.6 Hz, 1H), 7.17 (d, *J* = 7.7 Hz, 1H), 7.03 (s, 1H), 6.92 (d, *J* = 8.2 Hz, 1H), 6.78 (d, *J* = 2.1 Hz, 1H), 6.75 (dd, *J* = 8.2 Hz, *J* = 2.1 Hz, 1H), 5.38 (s, 2H), 5.21 (s, 2H), 4.28 (s, 4H), 3.83 (d, *J* = 3.7 Hz, 2H), 3.77 – 3.62 (m, 2H), 2.34 (s, 3H), 2.23 (s, 3H).

Figure S102: *N*-(5-chloro-2-((2-cyanopyridin-4-yl)methoxy)-4-((3-(2,3-dihydrobenzo[*b*][1,4]dioxin-6-yl)-2-methylbenzyl)oxy)benzyl)-*N*-methyl-*D*-serine (**5a**):

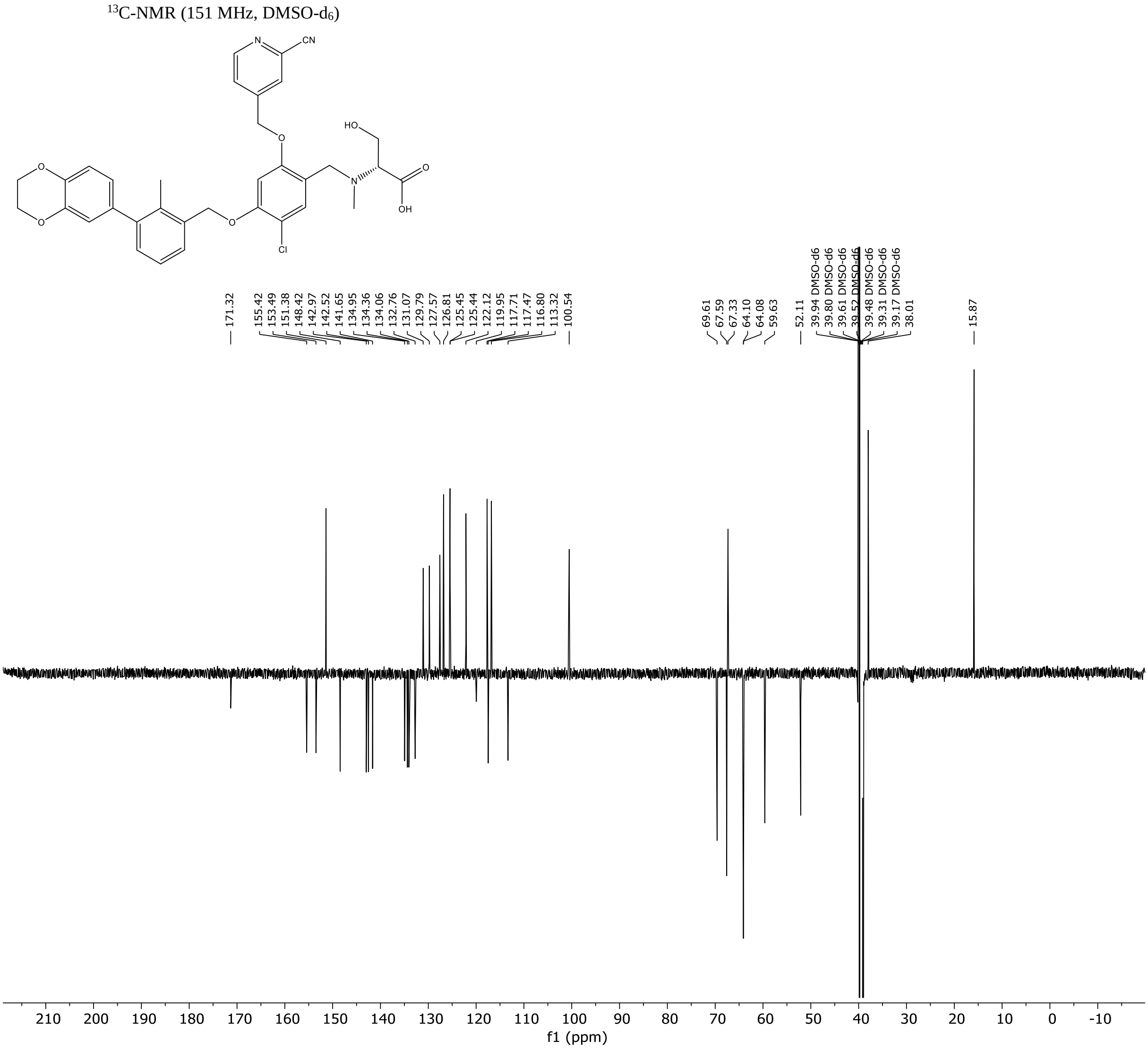

^{13}C-NMR (151 MHz, DMSO-d$_6$): δ 171.32, 155.42, 153.49, 151.38, 148.42, 142.97, 142.52, 141.65, 134.95, 134.36, 134.06, 132.76, 131.07, 129.79, 127.57, 126.81, 125.45, 125.44, 122.12, 119.95, 117.71, 117.47, 116.80, 113.32, 100.54, 69.61, 67.59, 67.33, 64.10, 64.08, 59.63, 52.11, 38.01, 15.87.

Figure S103: Methyl (5-chloro-2-((2-cyanopyridin-4-yl)methoxy)-4-((3-(2,3-dihydrobenzo[*b*][1,4]dioxin-6-yl)-2-methylbenzyl)oxy)benzyl)-*D*-serinate (**5b**):

^{1}H-NMR (700 MHz, $CDCl_3$)

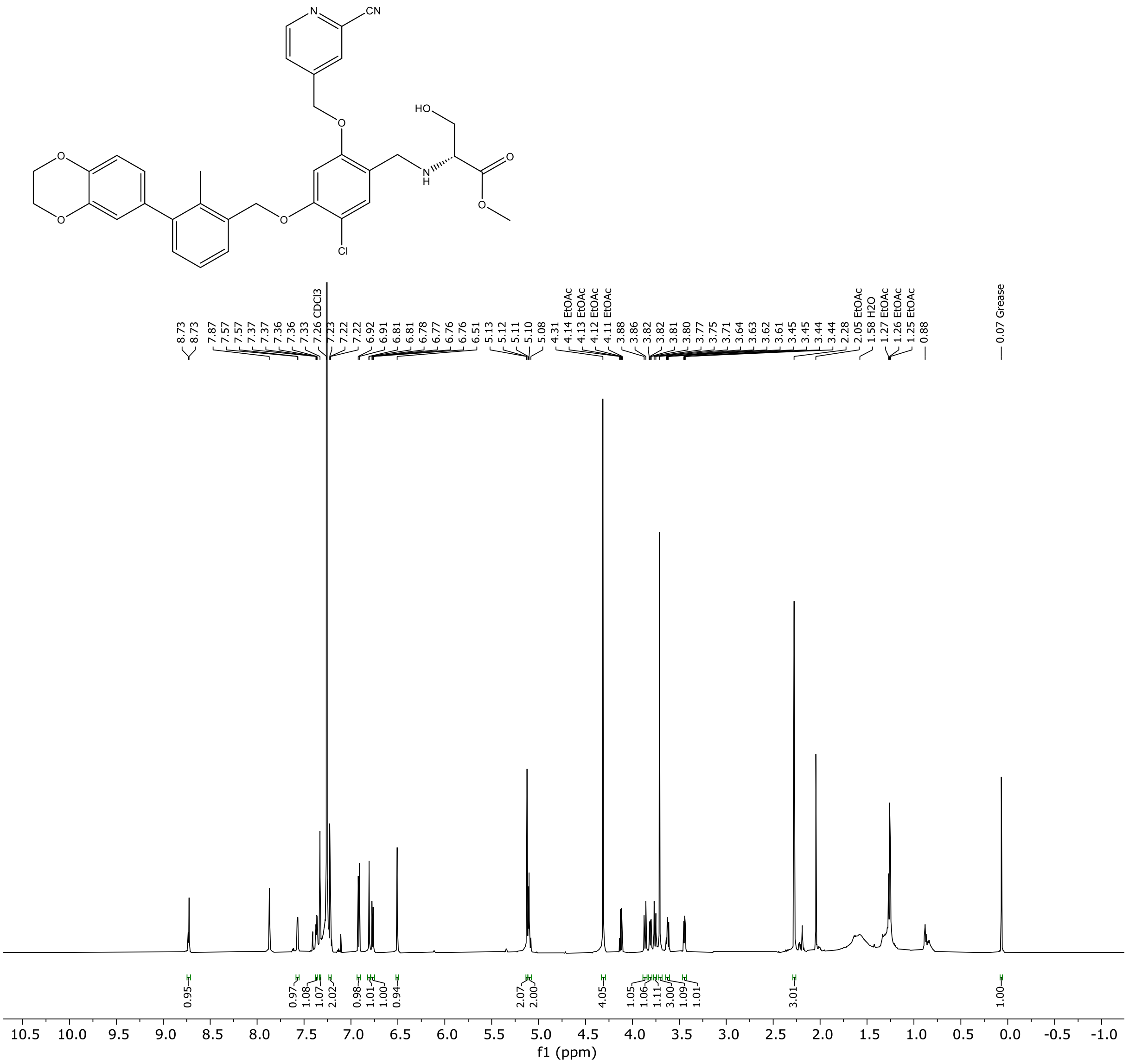

^{1}H-NMR (700 MHz, $CDCl_3$): δ 8.73 (d, *J* = 5.1 Hz, 1H), 7.87 (s, 1H), 7.57 (d, *J* = 5.1 Hz, 1H), 7.37 (dd, *J* = 6.3 Hz, *J* = 2.8 Hz 1H), 7.33 (s, 1H), 7.23 – 7.22 (m, 2H), 6.92 (d, *J* = 8.2 Hz, 1H), 6.81 (d, *J* = 2.1 Hz, 1H), 6.77 (dd, *J* = 8.2 Hz, *J* = 2.1 Hz, 1H), 6.51 (s, 1H), 5.12 (s, 2H), 5.12 (d, *J* = 14 Hz, 1H), 5.09 (d, *J* = 14 Hz, 1H), 4.31 (s, 4H), 3.87 (d, *J* = 13 Hz, 1H), 3.81 (dd, *J* = 11 Hz, *J* = 4.5 Hz, 1H), 3.76 (d, *J* = 13 Hz, 1H), 3.71 (s, 3H), 3.63 (dd, *J* = 11 Hz, *J* = 6.5 Hz, 1H), 3.44 (dd, *J* = 6.5 Hz, *J* = 4.5 Hz, 1H), 2.28 (s, 3H).

Figure S104: Methyl (5-chloro-2-((2-cyanopyridin-4-yl)methoxy)-4-((3-(2,3-dihydrobenzo[*b*][1,4]dioxin-6-yl)-2-methylbenzyl)oxy)benzyl)-*D*-serinate (**5b**):

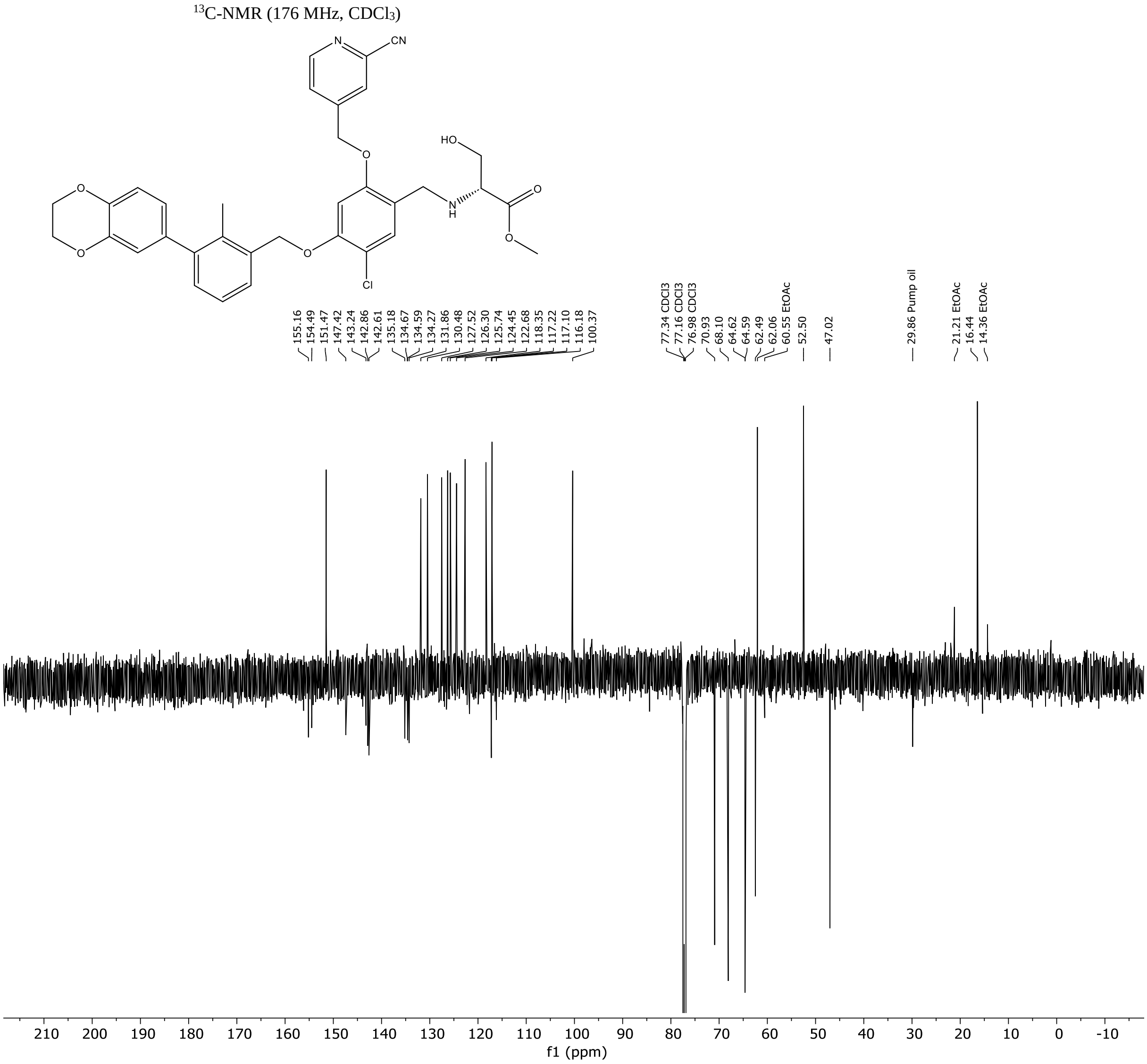

^{13}C-NMR (176 MHz, $CDCl_3$): δ 155.16, 154.49, 151.47, 147.42, 143.24, 142.86, 142.61, 135.18, 134.67, 134.59, 134.27, 131.86, 130.48, 127.52, 126.30, 125.74, 124.45, 122.68, 118.35, 117.22, 117.10, 116.18, 100.37, 70.93, 68.10, 64.62, 64.59, 62.49, 62.06, 52.50, 47.02, 16.44.

Figure S105: 4-((4-Chloro-5-((3-(2,3-dihydrobenzo[*b*][1,4]dioxin-6-yl)-2-methylbenzyl)oxy)-2-(((2-hydroxyethyl)(methyl)amino)methyl)phenoxy)methyl)picolinonitrile (**5c**):

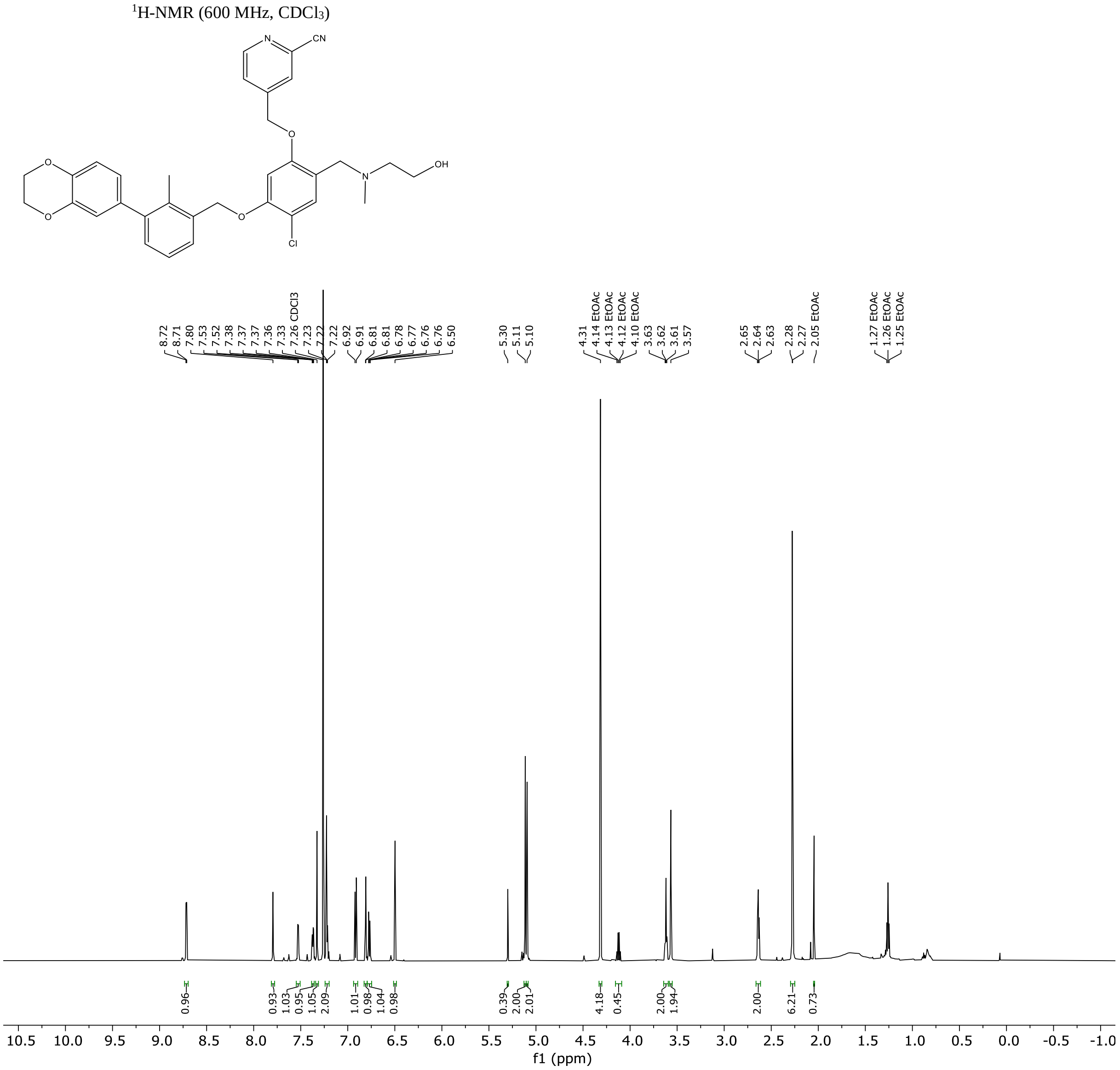

^{1}H-NMR (600 MHz, $CDCl_3$): δ 8.72 (d, *J* = 5.0 Hz, 1H), 7.80 (s, 1H), 7.53 (d, *J* = 5.0 Hz, 1H), 7.37 (dd, *J* = 6.2 Hz, *J* = 3.0 Hz, 1H), 7.33 (s, 1H), 7.23 – 7.22 (m, 2H), 6.92 (d, *J* = 8.2 Hz, 1H), 6.81 (d, *J* = 2.1 Hz, 1H), 6.77 (dd, *J* = 8.2 Hz, *J* = 2.1 Hz, 1H), 6.50 (s, 1H), 5.11 (s, 2H), 5.10 (s, 2H), 4.31 (s, 4H), 3.62 (t, *J* = 5.3 Hz, 2H), 3.57 (s, 2H), 2.64 (t, *J* = 5.3 Hz, 2H), 2.28 (s, 3H), 2.27 (s, 3H).

Figure S106: 4-((4-Chloro-5-((3-(2,3-dihydrobenzo[*b*][1,4]dioxin-6-yl)-2-methylbenzyl)oxy)-2-(((2-hydroxyethyl)(methyl)amino)methyl)phenoxy)methyl)picolinonitrile (**5c**):

^{13}C-NMR (151 MHz, $CDCl_3$)

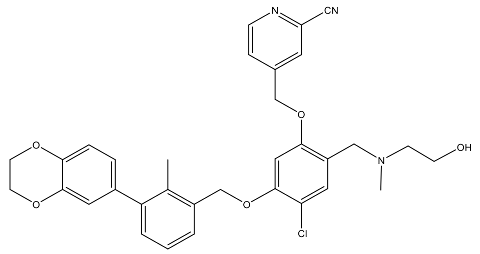

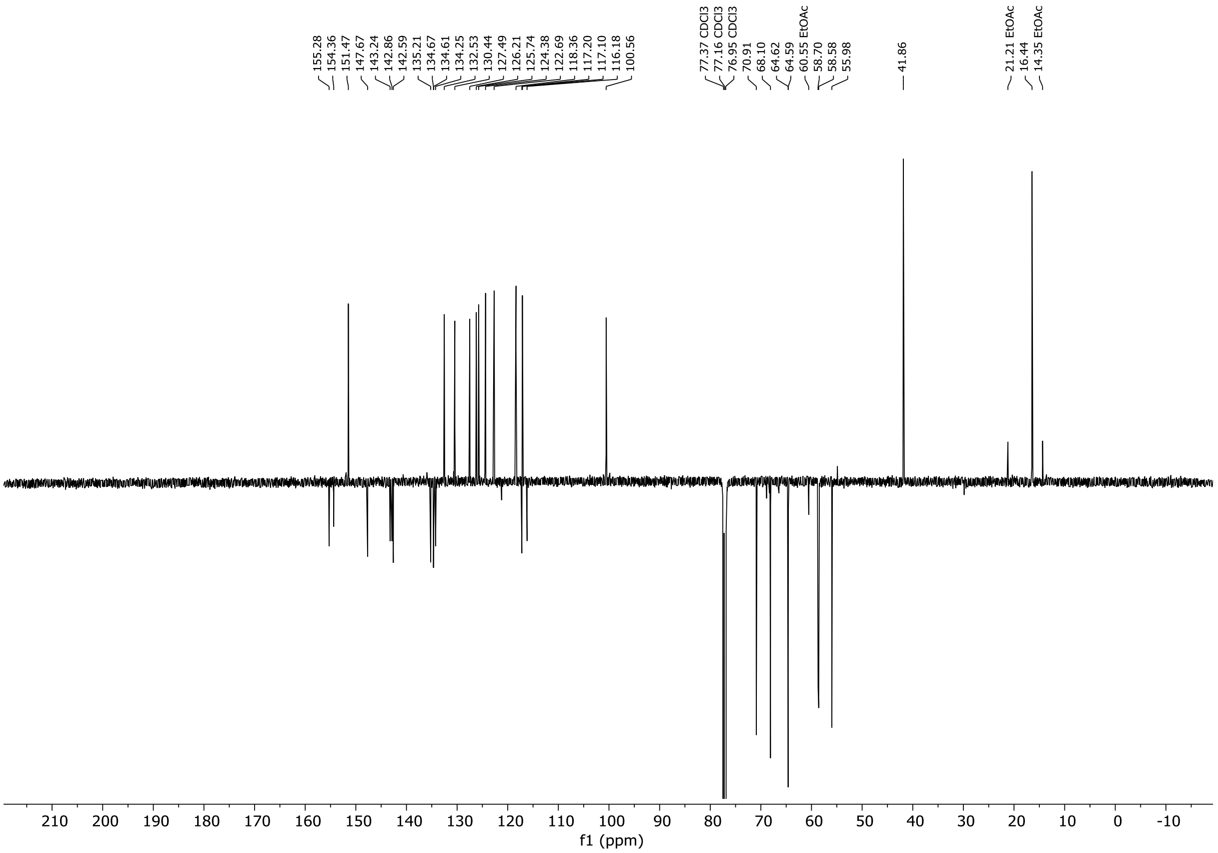

^{13}C-NMR (151 MHz, $CDCl_3$): δ 155.28, 154.36, 151.47, 147.67, 143.24, 142.86, 142.59, 135.21, 134.67, 134.61, 134.25, 132.53, 130.44, 127.49, 126.21, 125.74, 124.38, 122.69, 118.36, 117.20, 117.10, 116.18, 100.56, 70.91, 68.10, 64.62, 64.59, 58.70, 58.58, 55.98, 41.86, 16.44.

Figure S107: 4-((4-Chloro-5-((3-(2,3-dihydrobenzo[*b*][1,4]dioxin-6-yl)-2-methylbenzyl)oxy)-2-(((2-hydroxyethyl)(methyl)amino)methyl)phenoxy)methyl)picolinonitrile (**5c**):

^{1}H-^{1}H COSY NMR (600 MHz, $CDCl_3$)

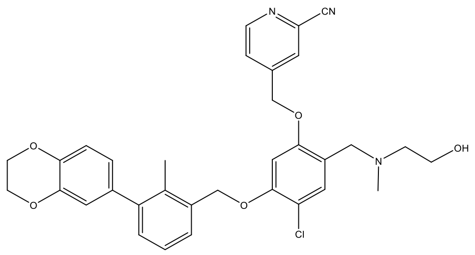

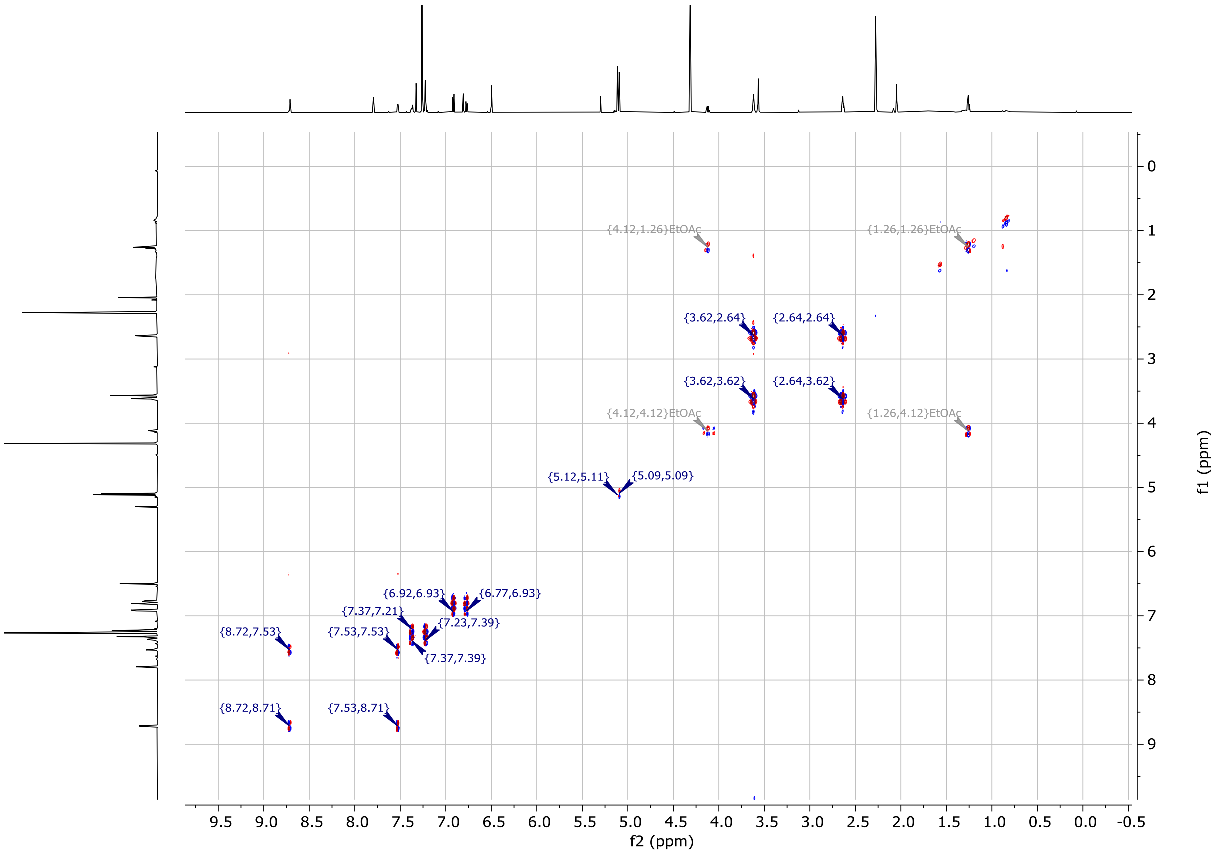

^{1}H-NMR (600 MHz, $CDCl_3$): δ 8.71, 8.71, 7.53, 7.53, 7.39, 7.39, 7.21, 7.20, 6.93, 6.93, 6.76, 6.76, 5.11, 5.09, 3.62, 3.62, 2.64, 2.64.

^{1}H-NMR (600 MHz, $CDCl_3$): δ 8.72, 8.72, 7.53, 7.53, 7.37, 7.37, 7.23, 7.22, 6.92, 6.92, 6.77, 6.77, 5.12, 5.09, 3.62, 3.62, 2.64, 2.64.

Figure S108: 4-((4-Chloro-5-((3-(2,3-dihydrobenzo[*b*][1,4]dioxin-6-yl)-2-methylbenzyl)oxy)-2-(((2-hydroxyethyl)(methyl)amino)methyl)phenoxy)methyl)picolinonitrile (**5c**):

^{1}H-^{13}C HSQC NMR (600 MHz, 151 MHz, $CDCl_3$)

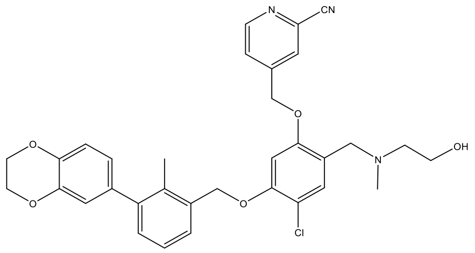

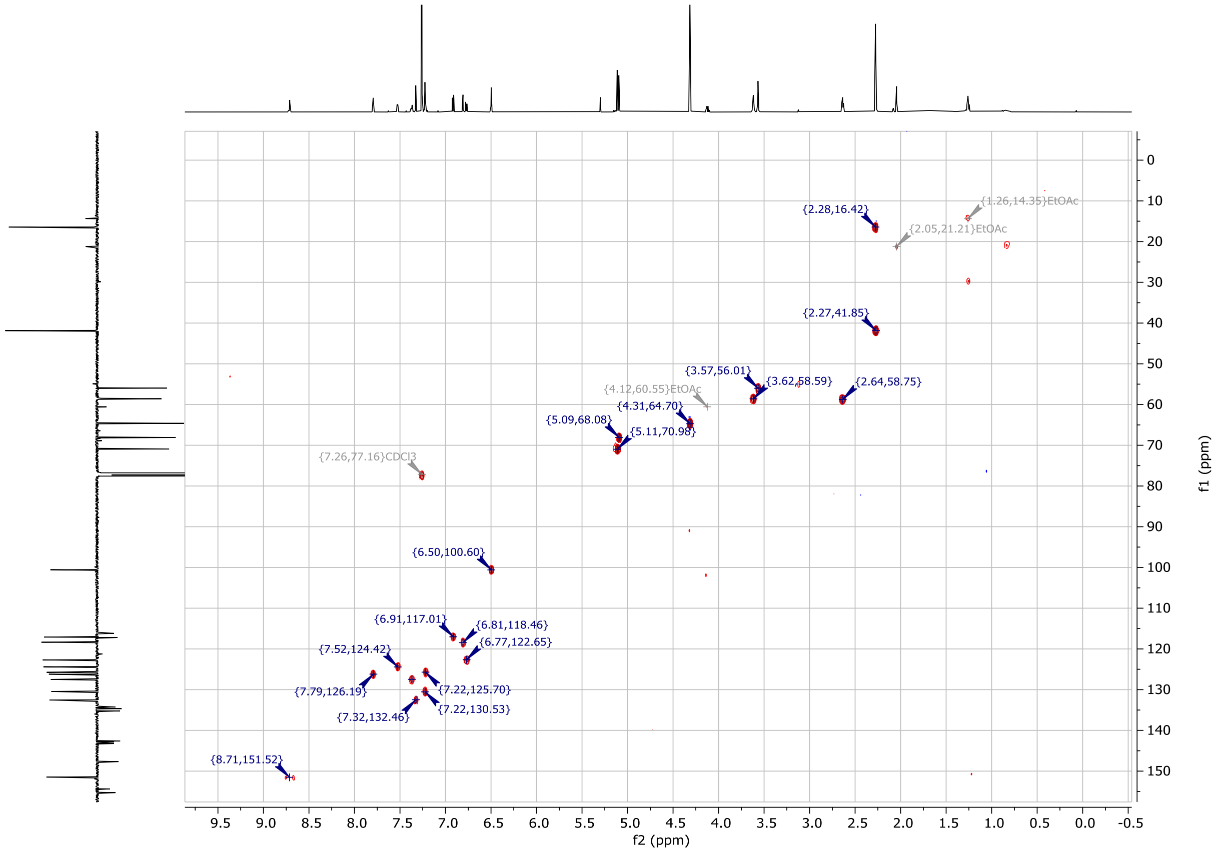

^{13}C-NMR (151 MHz, $CDCl_3$): δ 151.52, 132.46, 130.53, 127.47, 126.19, 125.70, 124.42, 122.65, 118.46, 117.01, 100.60, 70.98, 68.08, 64.70, 58.75, 58.59, 56.01, 55.05, 41.85, 16.42.

^{1}H-NMR (600 MHz, $CDCl_3$): δ 8.71, 7.79, 7.52, 7.37, 7.32, 7.22, 7.22, 6.91, 6.81, 6.77, 6.50, 5.11, 5.09, 4.31, 3.62, 3.57, 3.12, 2.64, 2.28, 2.27.

Figure S109: 4-((4-Chloro-5-((3-(2,3-dihydrobenzo[*b*][1,4]dioxin-6-yl)-2-methylbenzyl)oxy)-2-(((2-methoxyethyl)amino)methyl)phenoxy)methyl)picolinonitrile (**5d**):

^{1}H-NMR (700 MHz, $CDCl_3$)

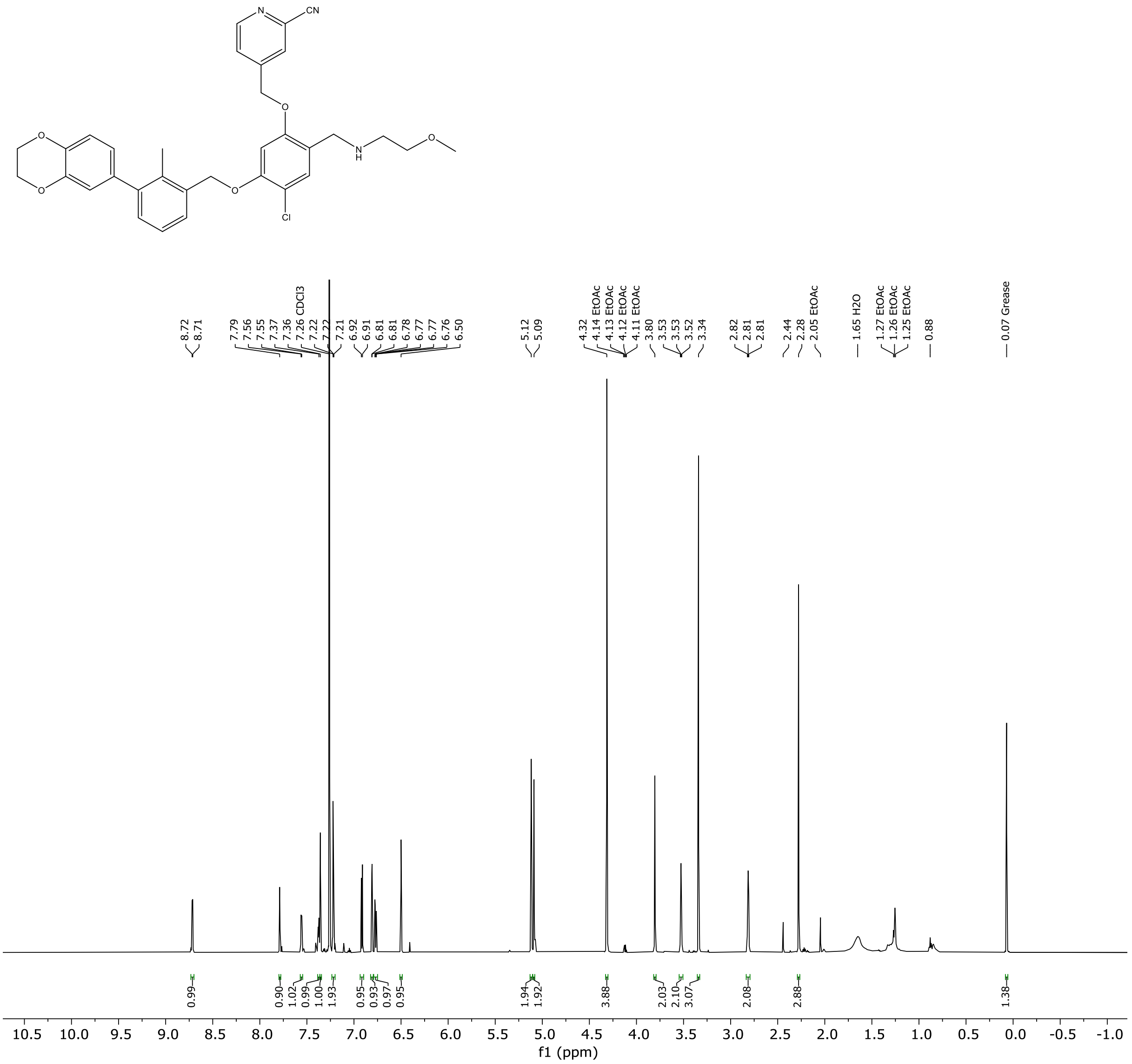

^{1}H-NMR (700 MHz, $CDCl_3$): δ 8.72 (d, *J* = 5.0 Hz, 1H), 7.79 (s, 1H), 7.56 (d, *J* = 5.0 Hz, 1H), 7.37 (dd, *J* = 6.2 Hz, *J* = 3.0 Hz, 1H), 7.36 (s, 1H), 7.22 – 7.21 (m, 2H), 6.92 (d, *J* = 8.2 Hz, 1H), 6.81 (d, *J* = 2.1 Hz, 1H), 6.77 (dd, *J* = 8.2 Hz, *J* = 2.1 Hz, 1H), 6.50 (s, 1H), 5.12 (s, 2H), 5.09 (s, 2H), 4.32 (s, 4H), 3.80 (s, 2H), 3.53 (t, *J* = 5.1 Hz, 2H), 3.34 (s, 3H), 2.81 (t, *J* = 5.1 Hz, 2H), 2.28 (s, 3H).

Figure S110: 4-((4-Chloro-5-((3-(2,3-dihydrobenzo[*b*][1,4]dioxin-6-yl)-2-methylbenzyl)oxy)-2-(((2-methoxyethyl)amino)methyl)phenoxy)methyl)picolinonitrile (**5d**):

^{13}C-NMR (176 MHz, $CDCl_3$)

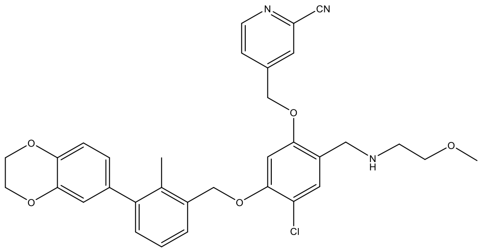

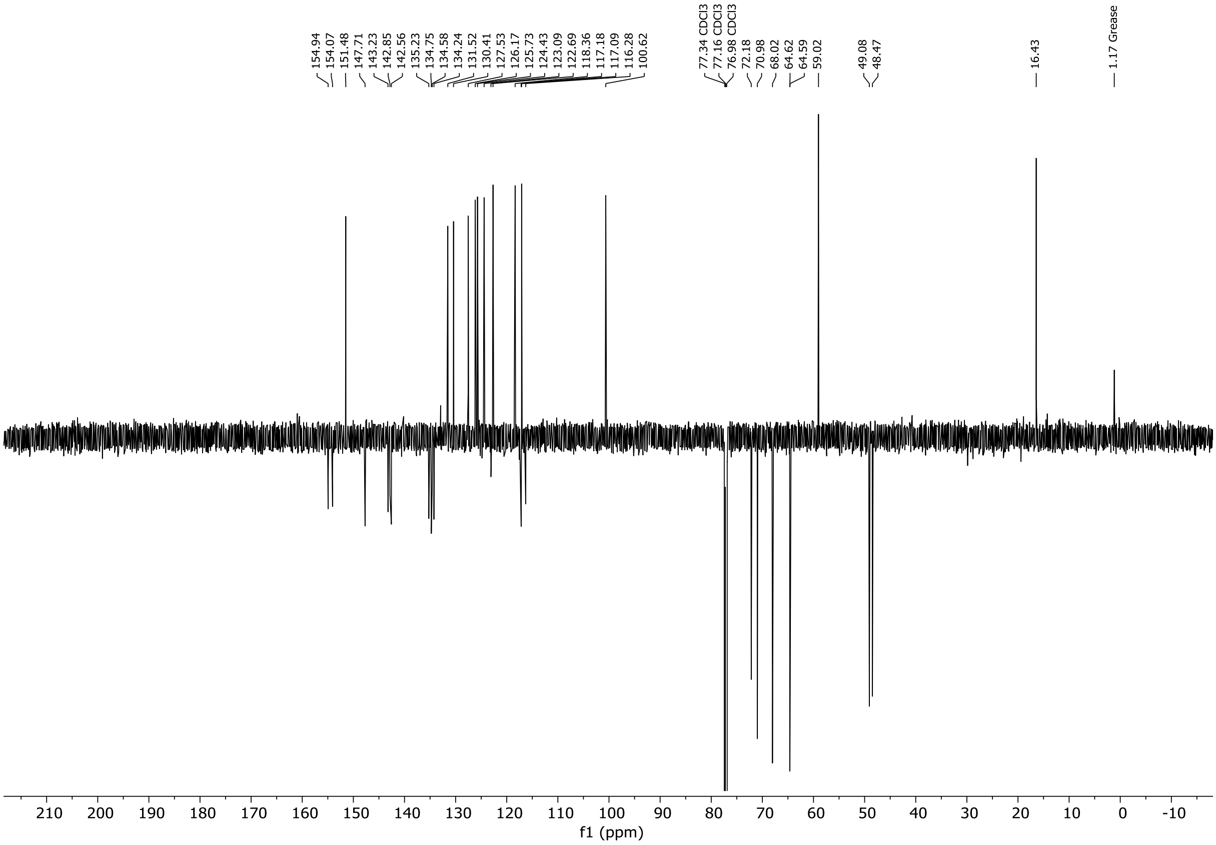

^{13}C-NMR (176 MHz, $CDCl_3$): δ 154.94, 154.07, 151.48, 147.71, 143.23, 142.85, 142.56, 135.23, 134.75, 134.58, 134.24, 131.52, 130.41, 127.53, 126.17, 125.73, 124.43, 123.09, 122.69, 118.36, 117.18, 117.09, 116.28, 100.62, 72.18, 70.98, 68.02, 64.62, 64.59, 59.02, 49.08, 48.47, 16.43.

Figure S111: 3-((2-((3-Acetyl-2-oxoimidazolidin-1-yl)methyl)-4-chloro-5-((3-(2,3-dihydrobenzo[*b*][1,4]dioxin-6-yl)-2-methylbenzyl)oxy)phenoxy)methyl)benzonitrile (**5e**):

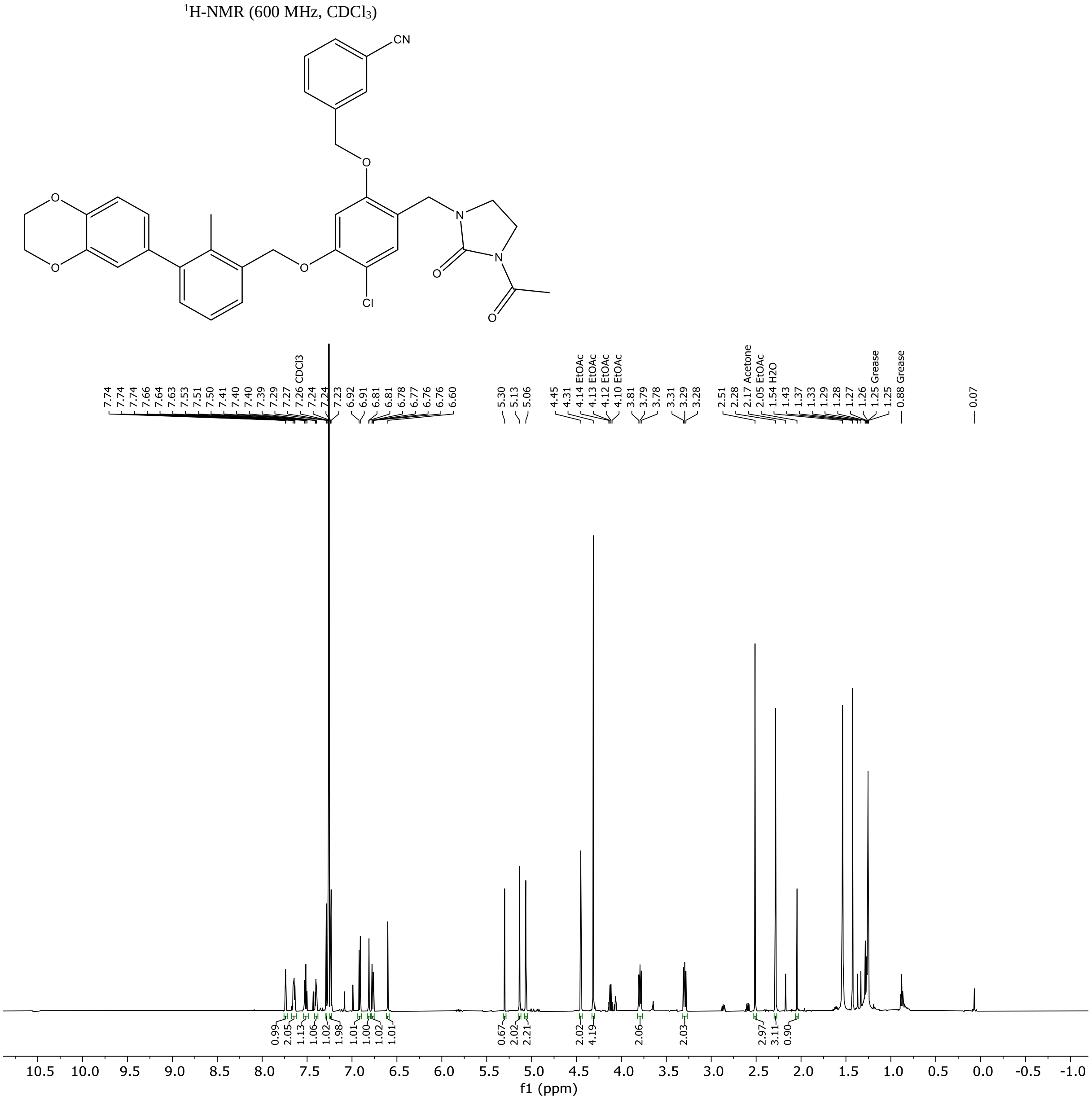

^{1}H-NMR (600 MHz, $CDCl_3$): δ 7.72 (s, 1H), 7.64 (m, 2H), 7.51 (t, *J* = 7.8 Hz, 1H), 7.40 (m, 1H), 7.29 (s, 1H), 7.24-7.23 (m, 2H), 6.91 (d, *J* = 8.2 Hz, 1H), 6.81 (d, *J* = 2.1 Hz, 1H), 6.77 (dd, *J* = 8.2 Hz, *J* = 2.1 Hz, 1H), 6.60 (s, 1H), 5.13 (s, 2H), 5.06 (s, 2H), 4.45 (s, 2H), 4.31 (s, 4H), 3.79 (t, *J* = 8.2 Hz, 2H), 3.29 (t, *J* = 8.2 Hz, 2H), 2.51 (s, 3H), 2.28 (s, 3H).

Figure S112: 3-((2-((3-Acetyl-2-oxoimidazolidin-1-yl)methyl)-4-chloro-5-((3-(2,3-dihydrobenzo[*b*][1,4]dioxin-6-yl)-2-methylbenzyl)oxy)phenoxy)methyl)benzonitrile (**5e**):

^{13}C-NMR (600 MHz, $CDCl_3$)

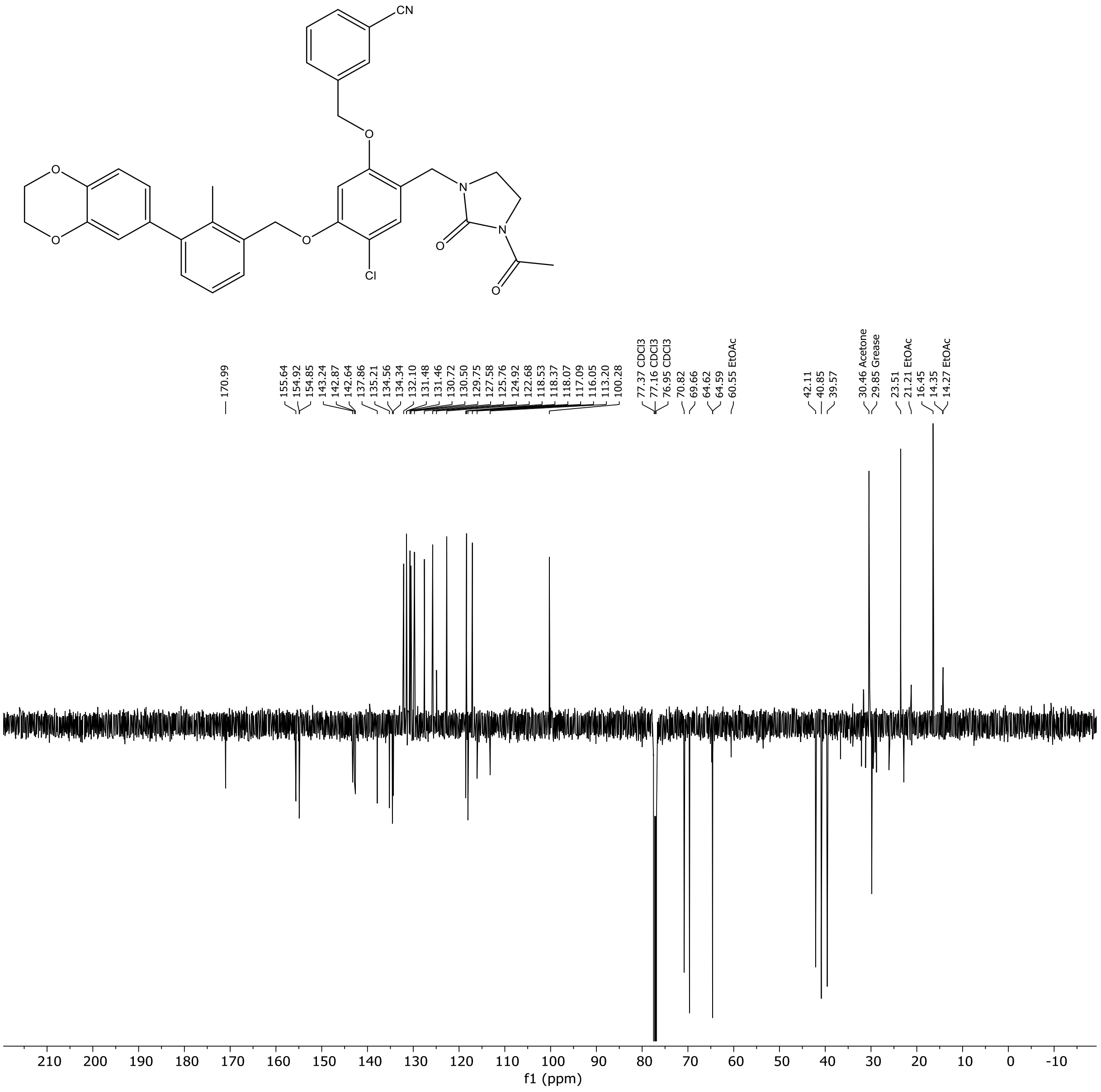

^{13}C-NMR (151 MHz, $CDCl_3$): δ 170.99, 155.64, 154.92, 154.85, 143.24, 142.87, 142.64, 137.86, 135.21, 134.56, 134.34, 132.10, 131.48, 131.46, 130.72, 130.50, 129.75, 127.58, 125.76, 124.92, 122.68, 118.53, 118.37, 118.07, 117.09, 116.05, 113.20, 100.28, 70.82, 69.66, 64.62, 64.59, 42.11, 40.85, 39.57, 23.51, 16.45, 14.35.

Figure S113: 4-((4-Chloro-2-(((2-hydroxyethyl)(methyl)amino)methyl)-5-((2-methyl-3-(1*H*-pyrrol-1-yl)benzyl)oxy)phenoxy)methyl)picolinonitrile (**5f**):

^{1}H-NMR (700 MHz, $CDCl_3$)

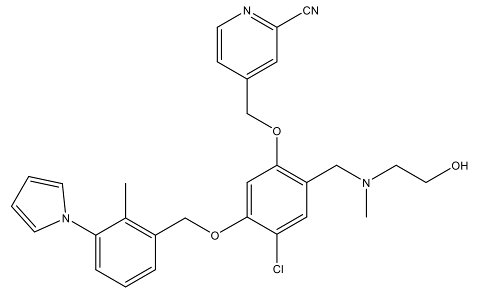

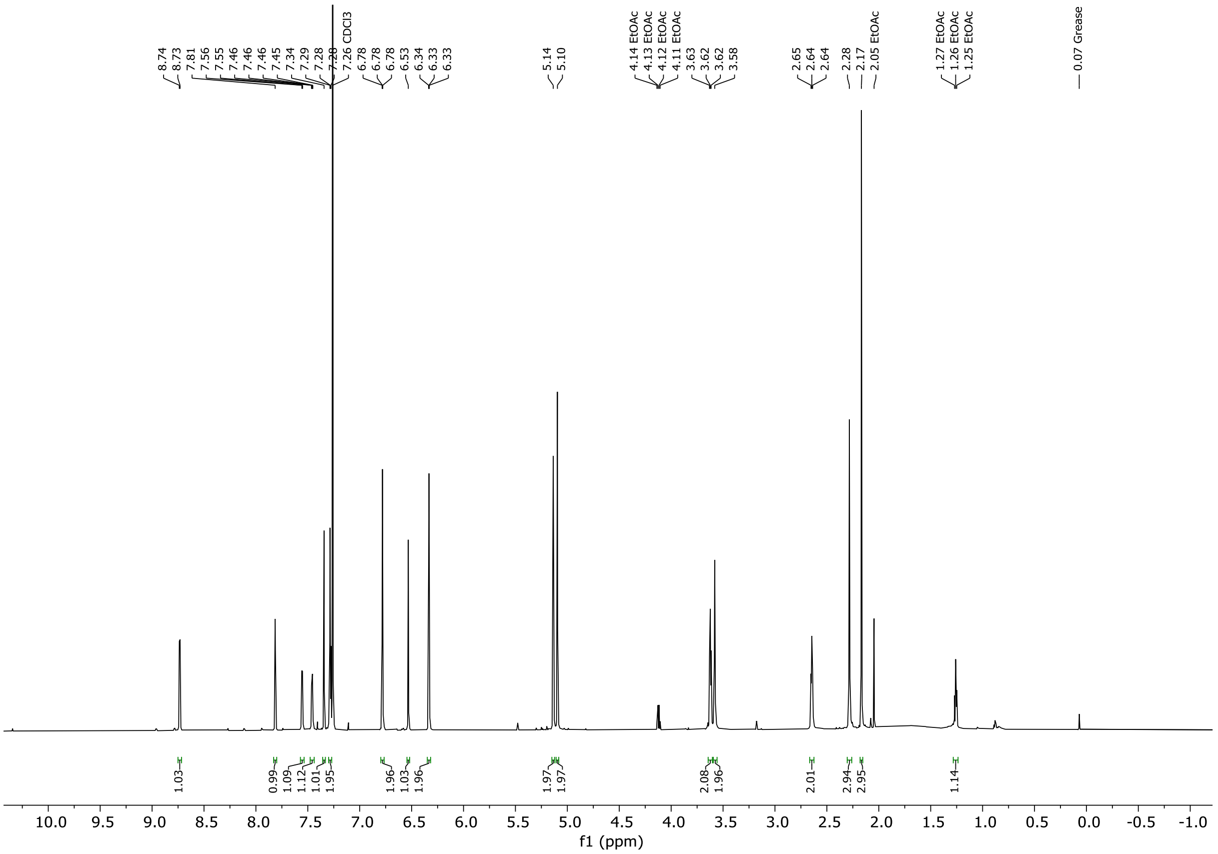

^{1}H-NMR (700 MHz, $CDCl_3$): δ 8.73 (d, *J* = 5.0 Hz, 1H), 7.81 (s, 1H), 7.56 (d, *J* = 5.0 Hz, 1H), 7.46 (dd, *J* = 6.4 Hz, *J* = 2.6 Hz, 1H), 7.34 (s, 1H), 7.29 – 7.28 (m, 2H), 6.78 (t, *J* = 2.1 Hz, 2H), 6.53 (s, 1H), 6.33 (t, *J* = 2.1 Hz, 2H), 5.14 (s, 2H), 5.10 (s, 2H), 3.62 (t, *J* = 5.2 Hz, 2H), 3.58 (s, 2H), 2.64 (t, *J* = 5.2 Hz, 2H), 2.28 (s, 3H), 2.17 (s, 3H).

Figure S114: 4-((4-Chloro-2-(((2-hydroxyethyl)(methyl)amino)methyl)-5-((2-methyl-3-(1*H*-pyrrol-1-yl)benzyl)oxy)phenoxy)methyl)picolinonitrile (**5f**):

^{13}C-NMR (176 MHz, $CDCl_3$)

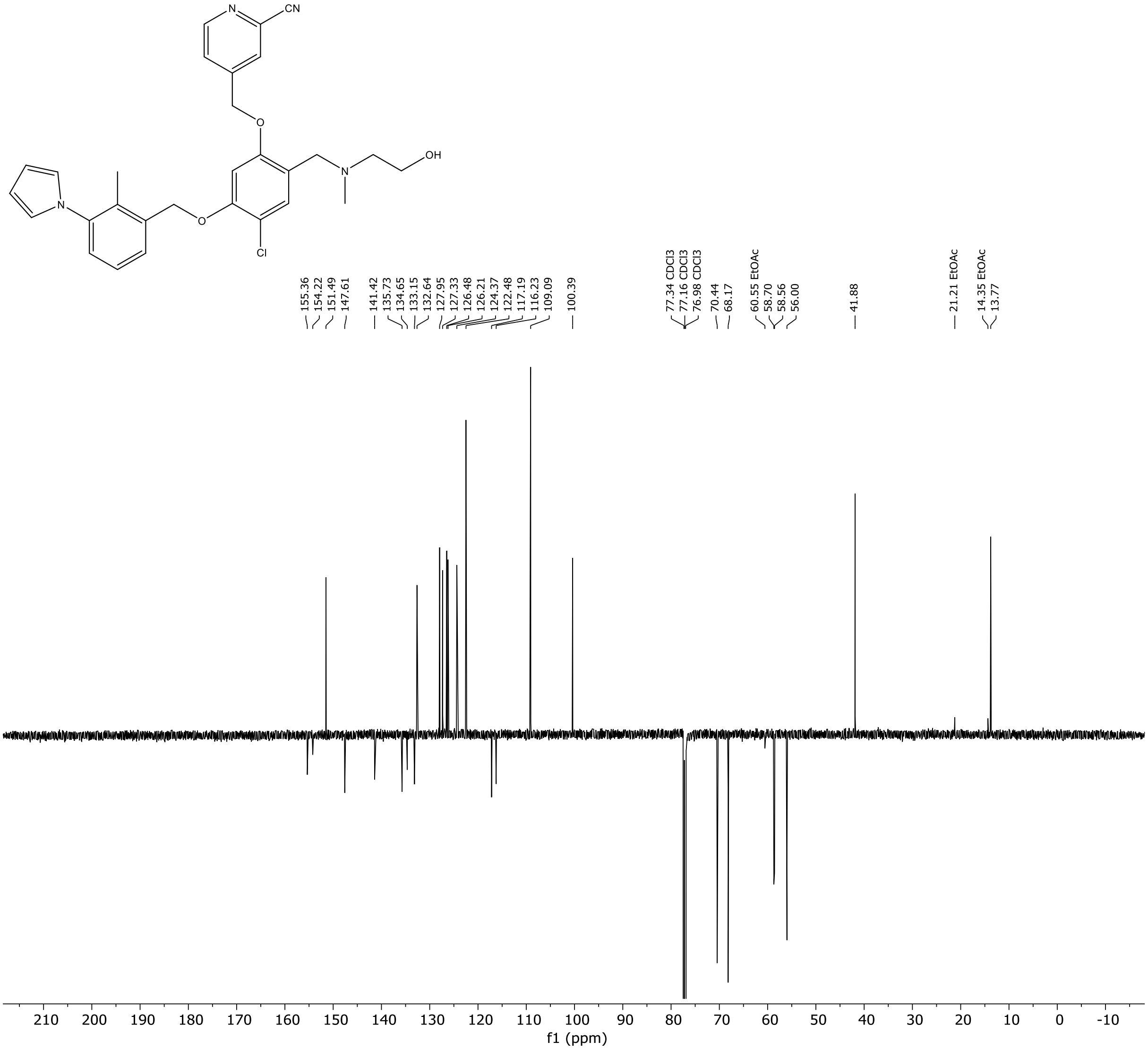

^{13}C-NMR (176 MHz, $CDCl_3$): δ 155.36, 154.22, 151.49, 147.61, 141.42, 135.73, 134.65, 133.15, 132.64, 127.95, 127.33, 126.48, 126.21, 124.37, 122.48, 117.19, 116.23, 109.09, 100.39, 70.44, 68.17, 58.70, 58.56, 56.00, 41.88, 13.77.

Figure S115: 4-((4-Chloro-2-(((2-hydroxyethyl)(methyl)amino)methyl)-5-((2-methyl-3-(1*H*-pyrrol-1-yl)benzyl)oxy)phenoxy)methyl)picolinonitrile (**5f**):

^{1}H-^{1}H COSY NMR (700 MHz, $CDCl_3$)

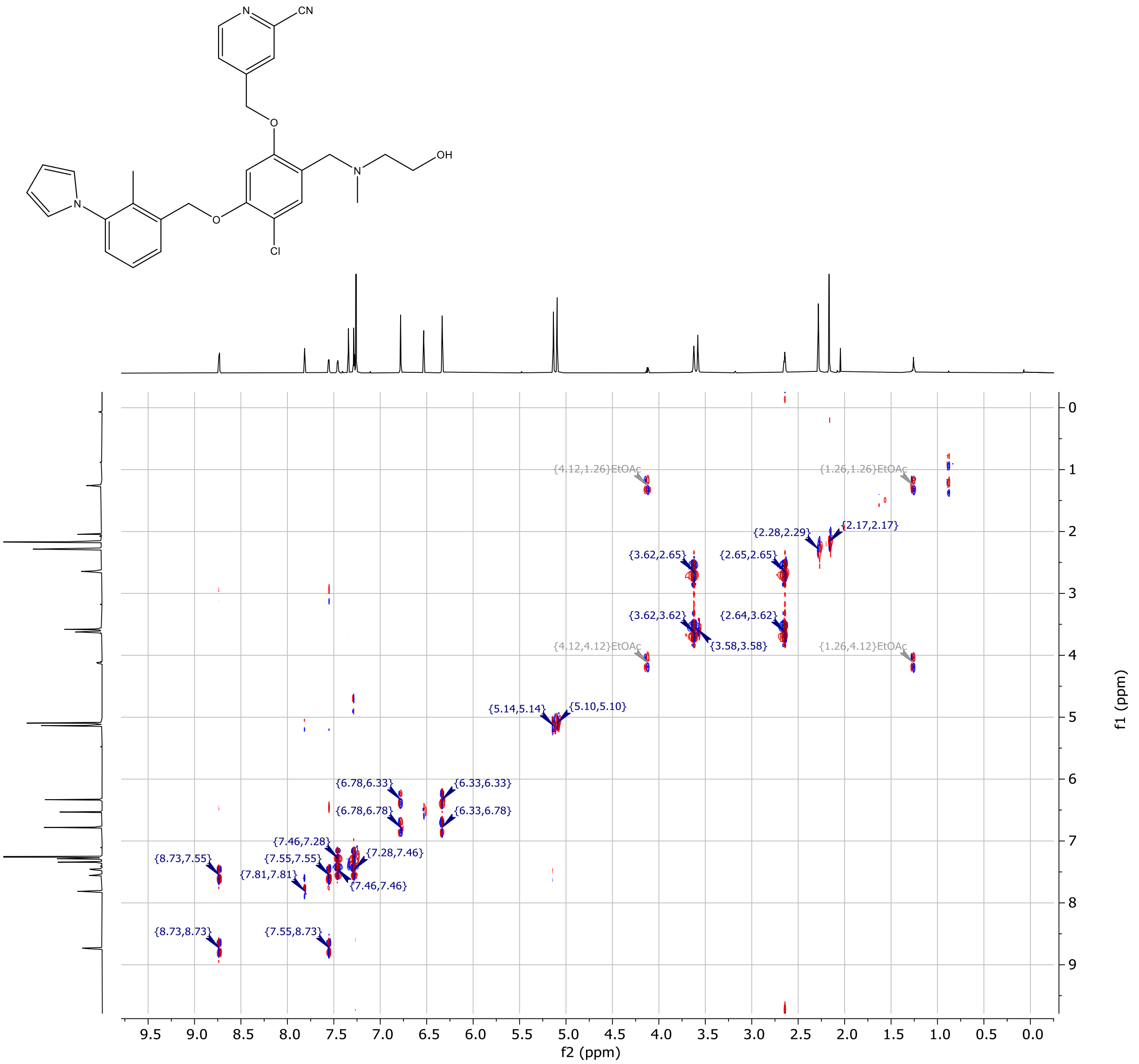

^{1}H-NMR (700 MHz, $CDCl_3$): δ 8.73, 8.73, 7.81, 7.55, 7.55, 7.46, 7.46, 7.28, 7.28, 6.78, 6.78, 6.53, 6.33, 6.33, 5.14, 5.10, 3.62, 3.62, 3.58, 2.65, 2.65, 2.29, 2.17.

^{1}H-NMR (700 MHz, $CDCl_3$): δ 8.73, 8.73, 7.81, 7.55, 7.55, 7.46, 7.46, 7.28, 7.28, 6.78, 6.78, 6.53, 6.33, 6.33, 5.14, 5.10, 3.62, 3.62, 3.58, 2.65, 2.64, 2.28, 2.17.

Figure S116: 4-((4-Chloro-2-(((2-hydroxyethyl)(methyl)amino)methyl)-5-((2-methyl-3-(1*H*-pyrrol-1-yl)benzyl)oxy)phenoxy)methyl)picolinonitrile (**5f**):

^{1}H-^{13}C HSQC NMR (700 MHz, 176 MHz, $CDCl_3$)

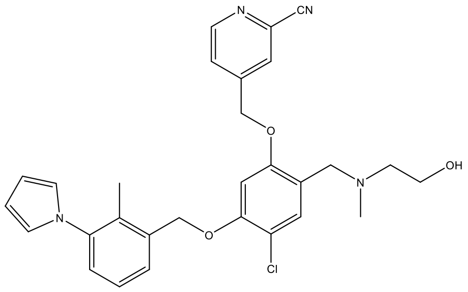

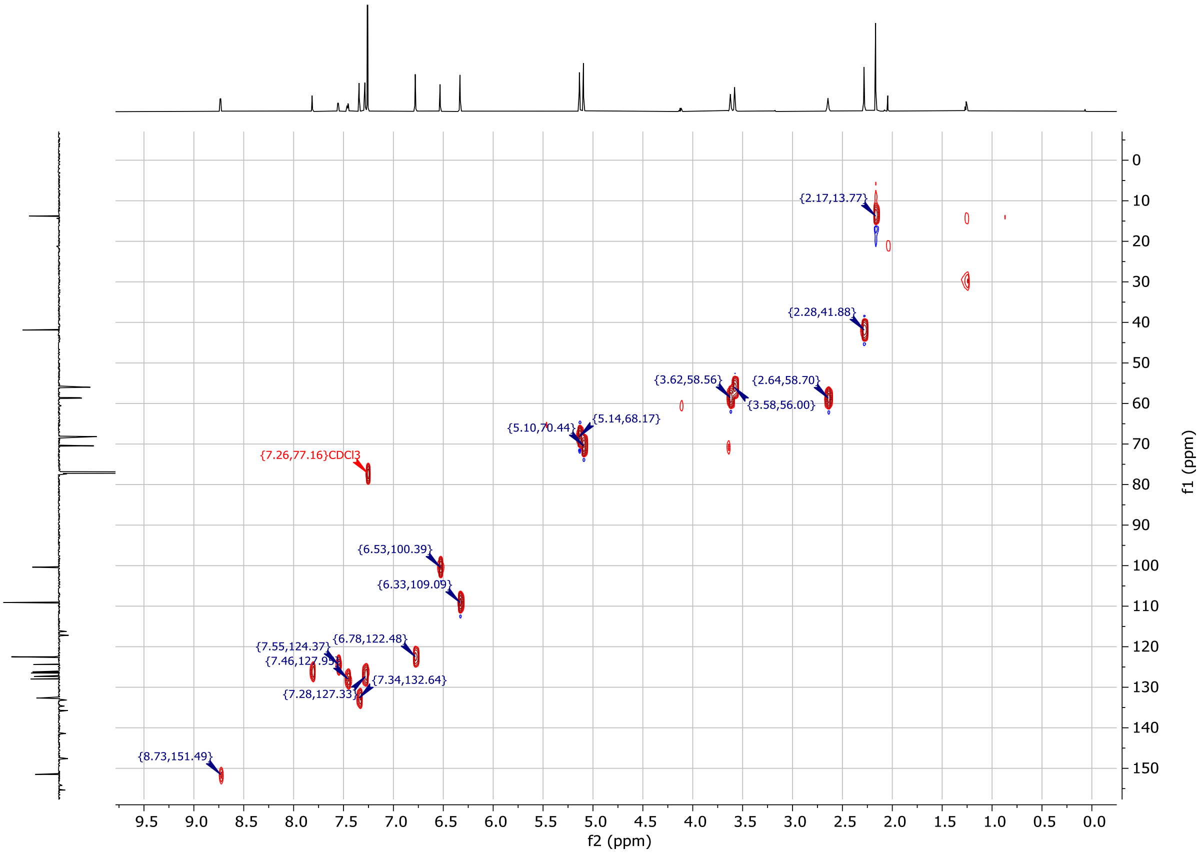

^{13}C-NMR (176 MHz, $CDCl_3$): δ 151.49, 132.64, 127.95, 127.33, 126.48, 126.21, 124.37, 122.48, 109.09, 100.39, 70.44, 68.17, 58.70, 58.56, 56.00, 41.88, 13.77

^{1}H-NMR (700 MHz, $CDCl_3$): δ 8.73, 7.81, 7.55, 7.46, 7.34, 7.28, 7.28, 6.78, 6.53, 6.33, 5.14, 5.10, 3.62, 3.58, 2.64, 2.28, 2.17.

Figure S117: 4-((4-Chloro-5-((3-(2,3-dihydrobenzo[*b*][1,4]dioxin-6-yl)-2-methylbenzyl)oxy)-2-((2-(fluoromethyl)oxazolidin-3-yl)methyl)phenoxy)methyl)picolinonitrile (**5g**):

^{1}H-NMR (700 MHz, $CDCl_3$)

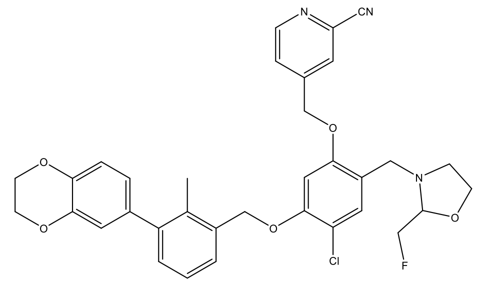

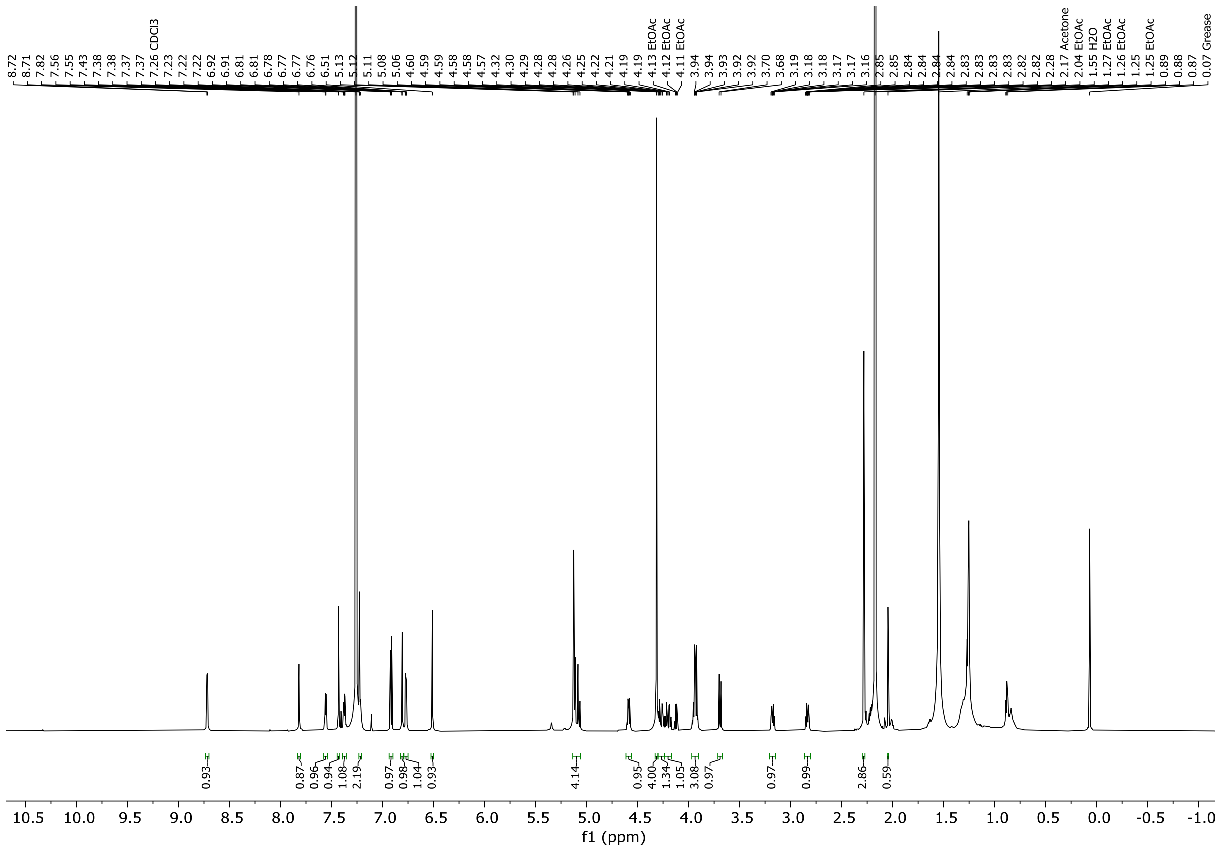

^{1}H-NMR (700 MHz, $CDCl_3$): δ 8.72 (d, *J* = 5.0 Hz, 1H), 7.82 (s, 1H), 7.56 (d, *J* = 5.0 Hz, 1H), 7.43 (s, 1H), 7.37 (dd, *J* = 6.3 Hz, *J* = 2.8 Hz, 1H), 7.23 – 7.22 (m, 2H), 6.92 (d, *J* = 8.2 Hz, 1H), 6.81 (d, *J* = 2.1 Hz, 1H), 6.77 (dd, *J* = 8.2 Hz, *J* = 2.1 Hz, 1H), 6.51 (s, 1H), 5.12 (s, 2H), 5.12 (d, *J* = 14 Hz, 1H), 5.07 (d, *J* = 14 Hz, 1H), 4.59 (dt, *J* = 11 Hz, *J* = 4.8 Hz, 1H), 4.32 (s, 4H), 4.25 (ddd, *J* = 47 Hz, *J* = 9.8 Hz, *J* = 4.7 Hz, 1H), 4.22 (ddd, *J* = 47 Hz, *J* = 9.8 Hz, *J* = 4.7 Hz, 1H), 3.94 – 3.92 (m, 2H), 3.93 (d, *J* = 13 Hz, 1H), 3.69 (d, *J* = 13 Hz, 1H), 3.18 (dt, *J* = 10 Hz, *J* = 6.1 Hz, 1H), 2.83 (dt, *J* = 10 Hz, *J* = 6.1 Hz, 2H), 2.28 (s, 3H).

Figure S118: 4-((4-Chloro-5-((3-(2,3-dihydrobenzo[*b*][1,4]dioxin-6-yl)-2-methylbenzyl)oxy)-2-((2-(fluoromethyl)oxazolidin-3-yl)methyl)phenoxy)methyl)picolinonitrile (**5g**):

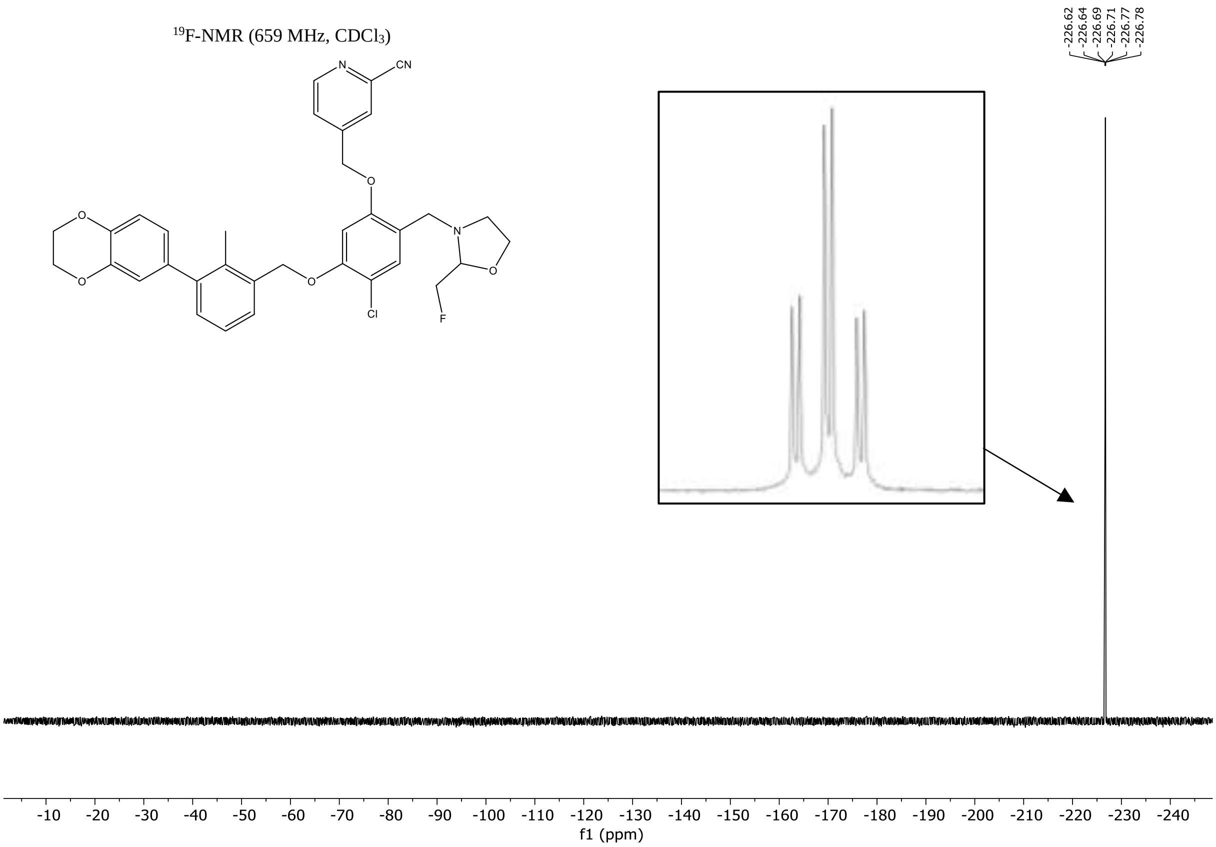

^{19}F-NMR (659 MHz, $CDCl_3$): δ -226.70 (td, *J* = 47 Hz, *J* = 11 Hz, 1F).

Figure S119: 4-((4-Chloro-5-((3-(2,3-dihydrobenzo[*b*][1,4]dioxin-6-yl)-2-methylbenzyl)oxy)-2-((2-(fluoromethyl)oxazolidin-3-yl)methyl)phenoxy)methyl)picolinonitrile (**5g**):

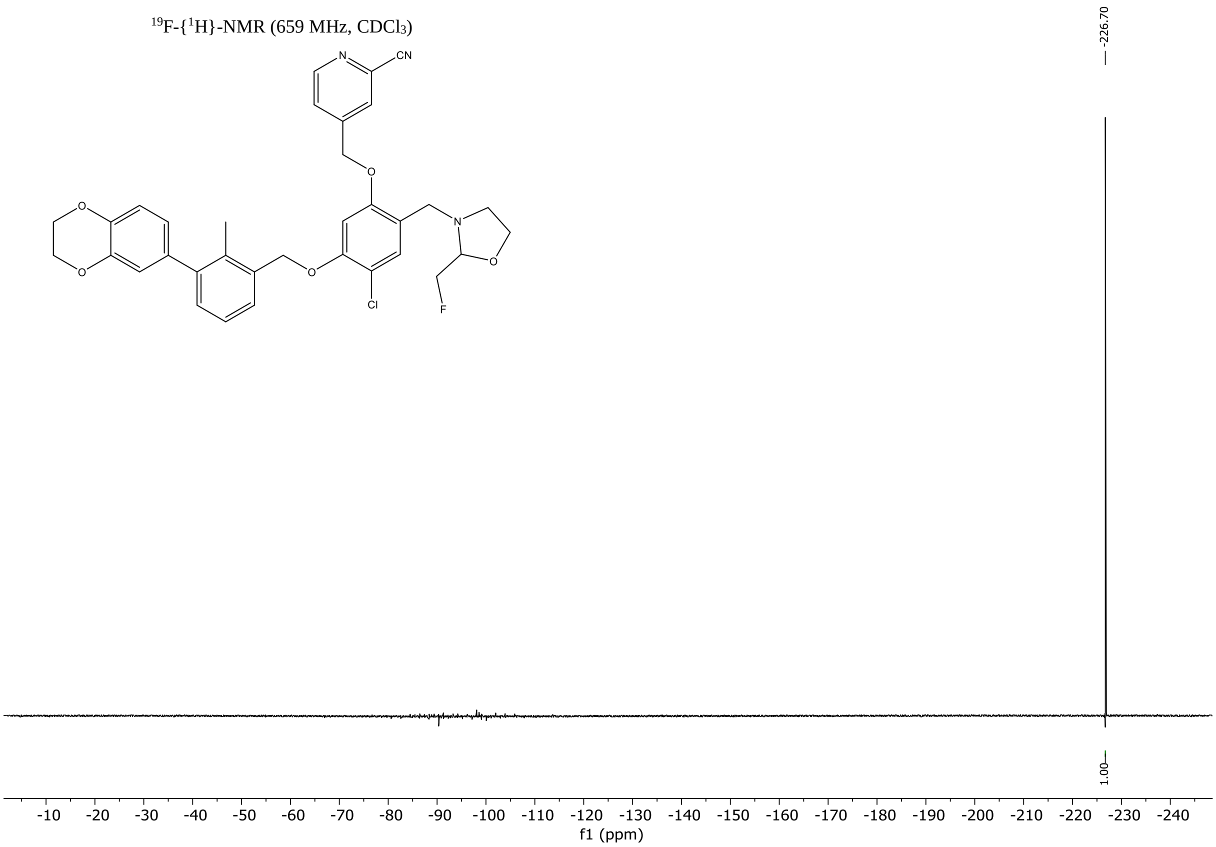

^{19}F-{^{1}H}-NMR (659 MHz, $CDCl_3$): δ -226.70 (s, 1F).

Figure S120: 2-Fluoroethyl (*S*)-1-(5-chloro-2-((2-cyanopyridin-4-yl)methoxy)-4-((3-(2,3-dihydrobenzo[*b*][1,4]dioxin-6-yl)-2-methylbenzyl)oxy)benzyl)piperidine-2-carboxylate (**5h**):

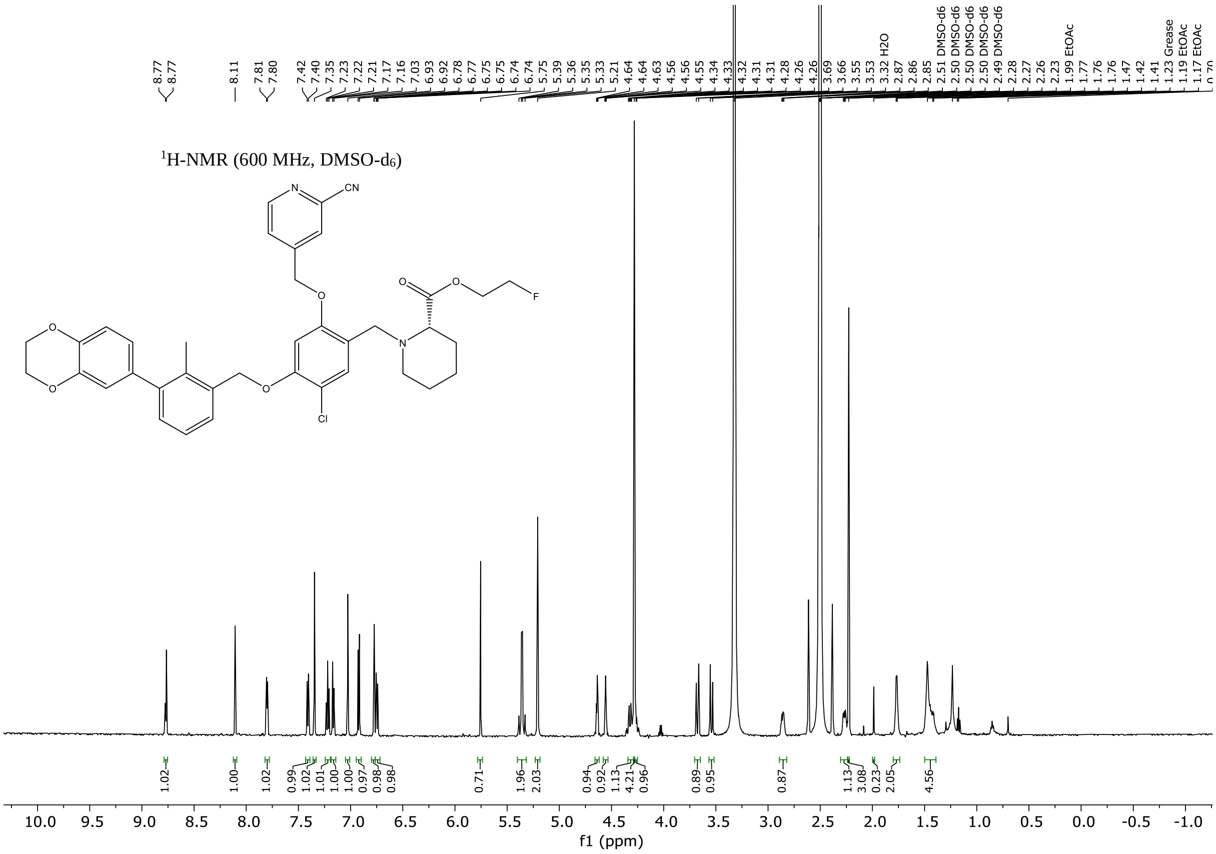

^{1}H-NMR (600 MHz, DMSO-d$_6$): δ 8.77 (d, *J* = 5.0 Hz, 1H), 8.11 (s, 1H), 7.80 (d, *J* = 5.0 Hz, 1H), 7.41 (d, *J* = 7.6 Hz, 1H), 7.35 (s, 1H), 7.22 (t, *J* = 7.6 Hz), 7.17 (d, *J* = 7.6 Hz), 7.03 (s, 1H), 6.92 (d, *J* = 8.2 Hz, 1H), 6.78 (d, *J* = 2.1 Hz, 1H), 6.75 (dd, *J* = 8.2 Hz, *J* = 2.1 Hz, 1H), 5.36 (s, *J* = 6.2 Hz, 1H), 5.21 (s, 1H), 4.60 (t, *J* = 48 Hz, *J* = 4.1 Hz, 1H), 4.34 – 4.26 (m, 2H), 4.28 (s, 4H), 3.68 (d, *J* = 14 Hz, 1H), 3.54 (d, *J* = 14 Hz, 1H), 2.86 (m, 1H), 2.27 (m, 1H), 2.23 (s, 3H), 1.76 (m, 2H), 1.47 – 1.41 (m, 4H).

Figure S121: 2-Fluoroethyl (*S*)-1-(5-chloro-2-((2-cyanopyridin-4-yl)methoxy)-4-((3-(2,3-dihydrobenzo[*b*][1,4]dioxin-6-yl)-2-methylbenzyl)oxy)benzyl)piperidine-2-carboxylate (**5h**):

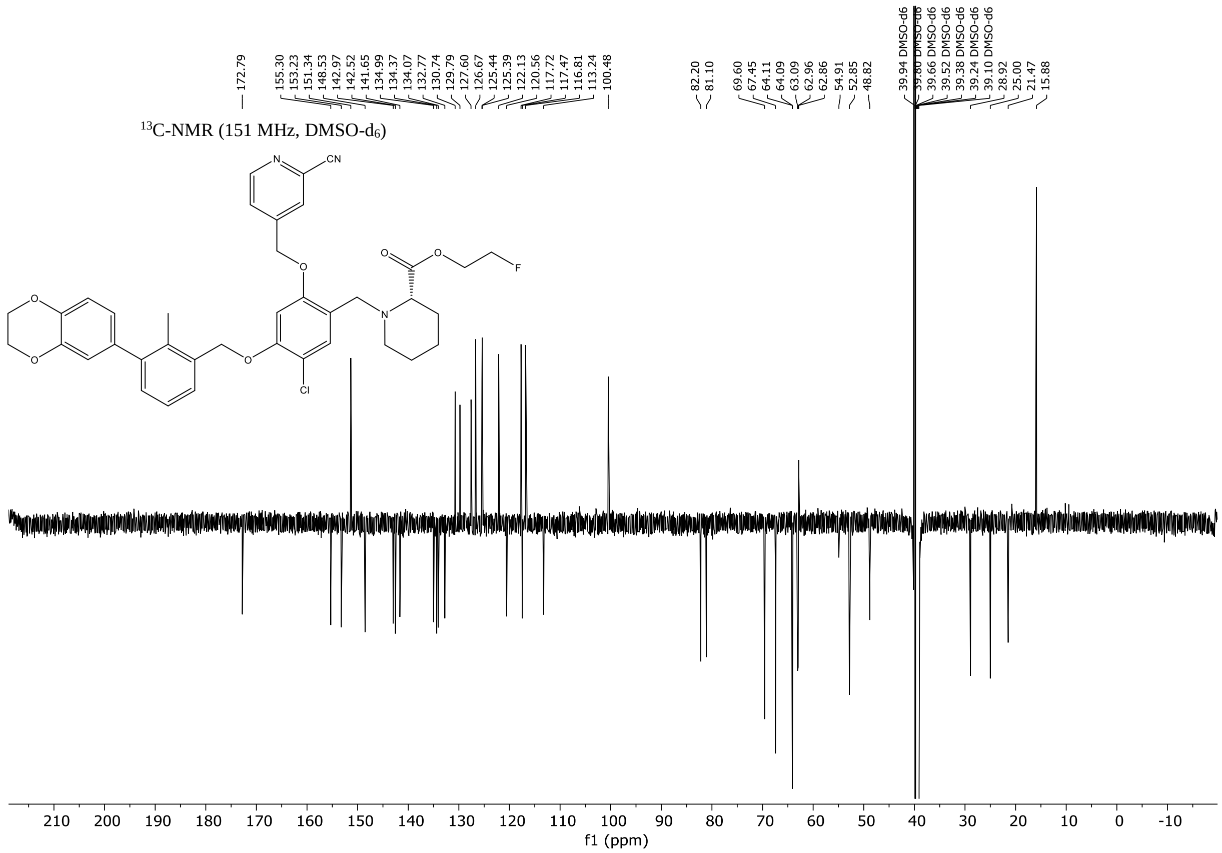

^{13}C-NMR (151 MHz, DMSO-d$_6$): δ 172.79, 155.30, 153.23, 151.34, 148.53, 142.97, 142.52, 141.65, 134.99, 134.37, 134.07, 132.77, 130.74, 129.79, 127.60, 126.67, 125.44, 125.39, 122.13, 120.56, 117.72, 117.47, 116.81, 113.24, 100.48, 81.15 (*J* = 165 Hz), 69.60, 67.45, 64.11, 64.09, 63.03 (*J* = 19 Hz), 62.86, 52.85, 48.82, 28.92, 25.00, 21.47, 15.88.

Figure S122: 2-Fluoroethyl (*S*)-1-(5-chloro-2-((2-cyanopyridin-4-yl)methoxy)-4-((3-(2,3-dihydrobenzo[*b*][1,4]dioxin-6-yl)-2-methylbenzyl)oxy)benzyl)piperidine-2-carboxylate (**5h**):

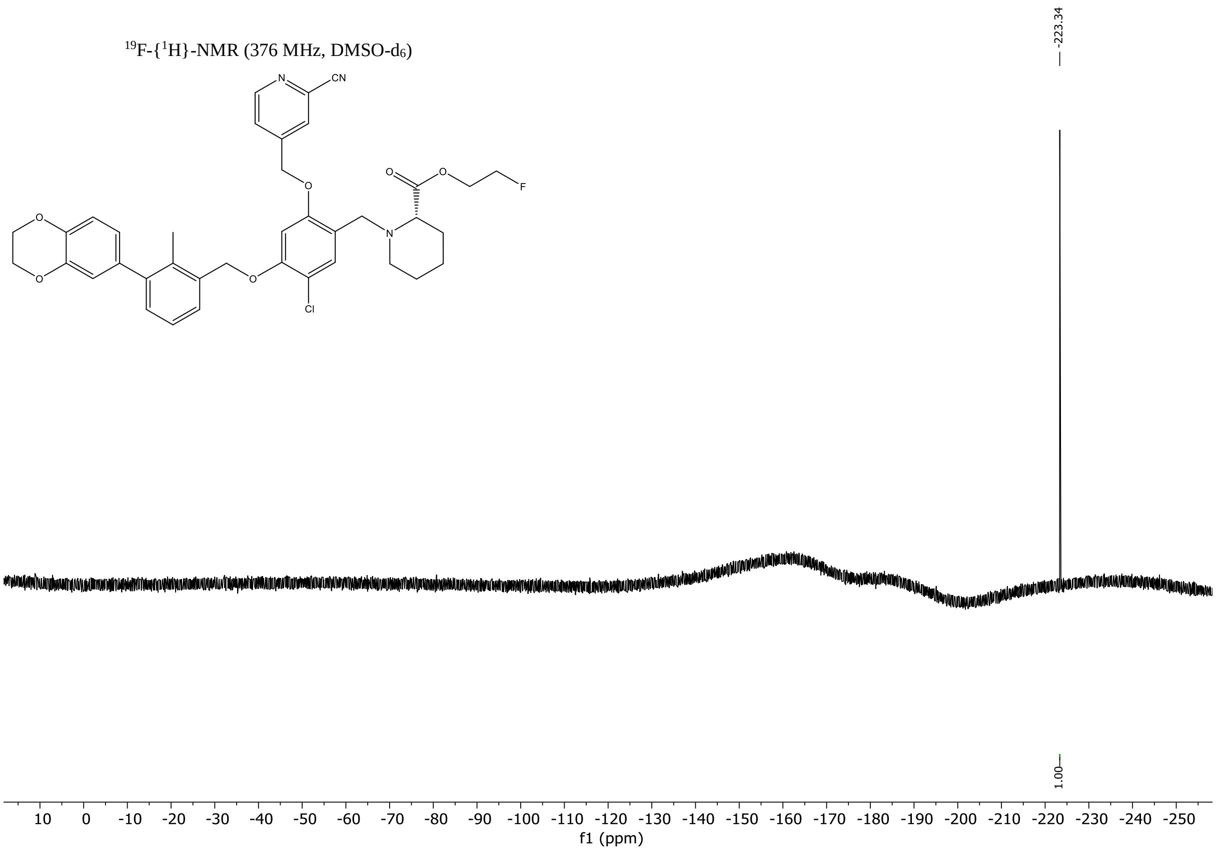

^{19}F-{^{1}H}-NMR (376 MHz, DMSO-d_6): δ -223.34 (s, 1F).

Figure S123: 2-Fluoroethyl (*S*)-1-(5-chloro-2-((2-cyanopyridin-4-yl)methoxy)-4-((3-(2,3-dihydrobenzo[*b*][1,4]dioxin-6-yl)-2-methylbenzyl)oxy)benzyl)piperidine-2-carboxylate (**5h**):

^{1}H-^{1}H COSY NMR (600 MHz, DMSO-d_6)

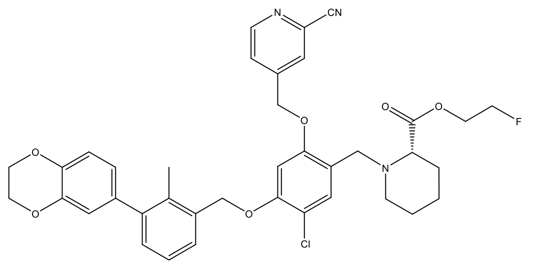

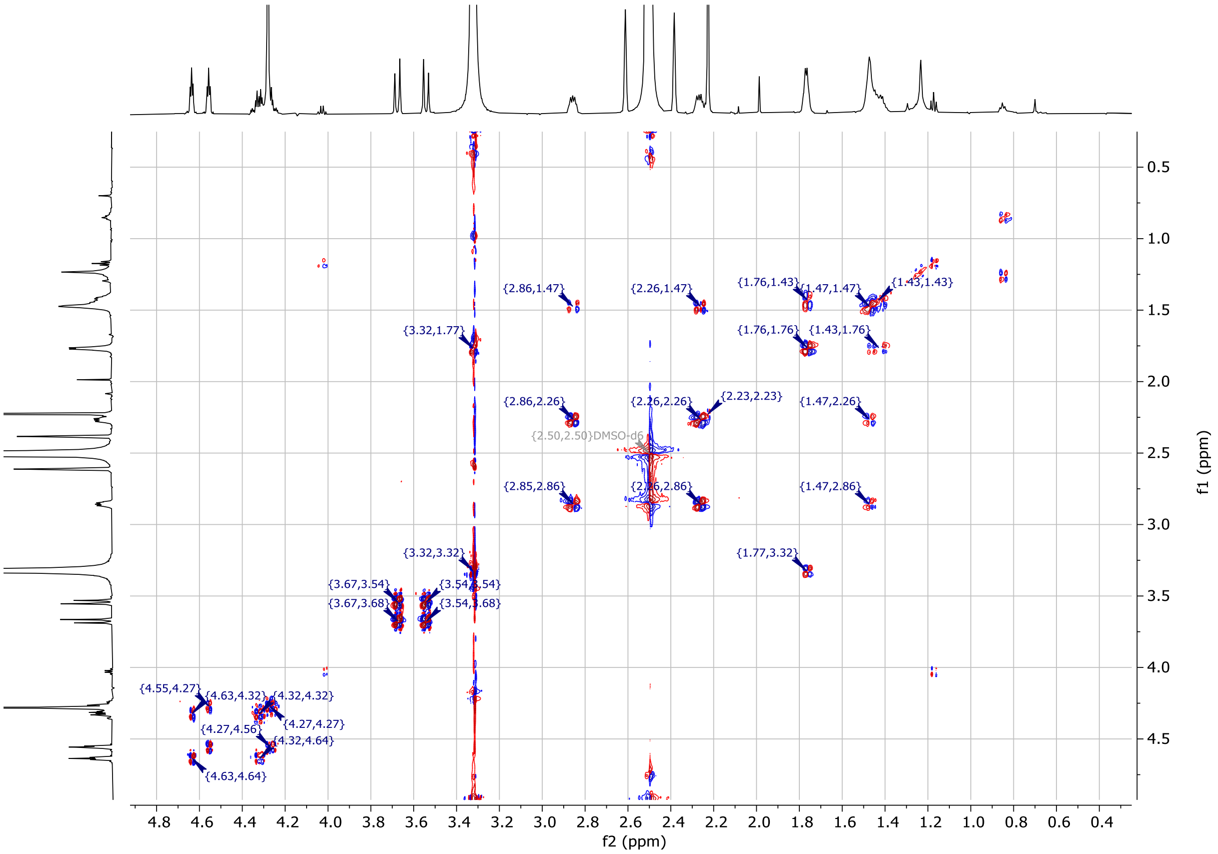

^{1}H-NMR (600 MHz, DMSO-d_6): δ 4.64, 4.64, 4.56, 4.56, 4.32, 4.32, 4.27, 4.27, 3.68, 3.68, 3.54, 3.54, 3.32, 3.32, 2.86, 2.86, 2.86, 2.26, 2.26, 2.26, 2.23, 1.77, 1.76, 1.76, 1.47, 1.47, 1.47, 1.43, 1.43.

^{1}H-NMR (600 MHz, DMSO-d_6): δ 4.64, 4.64, 4.56, 4.56, 4.32, 4.32, 4.27, 4.27, 3.68, 3.68, 3.54, 3.54, 3.32, 3.32, 2.86, 2.86, 2.86, 2.26, 2.26, 2.26, 2.23, 1.77, 1.76, 1.76, 1.47, 1.47, 1.47, 1.43, 1.43.

Figure S124: 2-Fluoroethyl (*S*)-1-(5-chloro-2-((2-cyanopyridin-4-yl)methoxy)-4-((3-(2,3-dihydrobenzo[*b*][1,4]dioxin-6-yl)-2-methylbenzyl)oxy)benzyl)piperidine-2-carboxylate (**5h**):

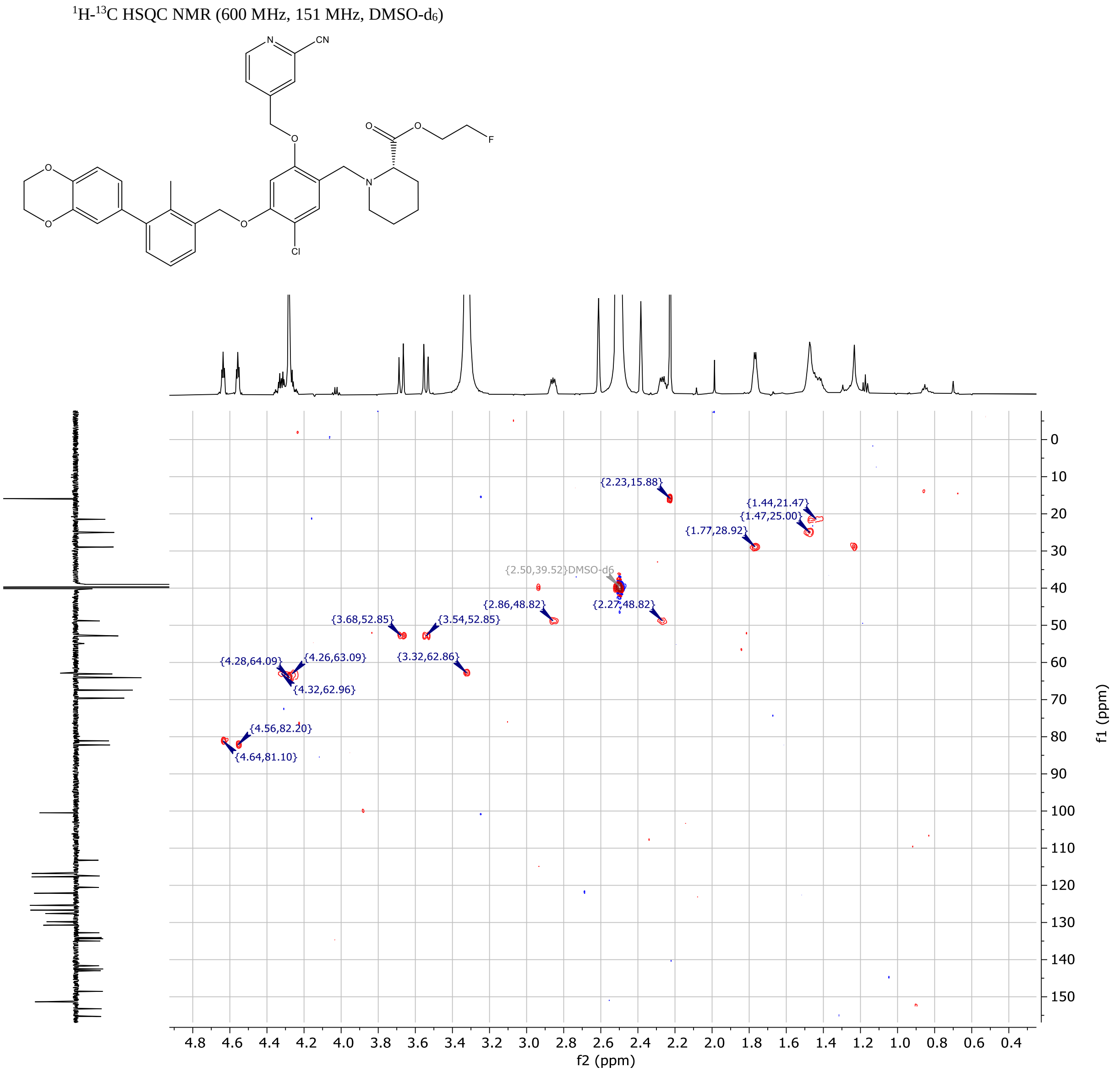

^{13}C-NMR (151 MHz, DMSO-d$_6$): δ 82.20, 81.10, 64.09, 63.09, 62.96, 62.86, 52.85, 52.85, 48.82, 48.82, 39.52, 28.92, 25.00, 21.47, 15.88.

^{1}H-NMR (600 MHz, DMSO-d$_6$): δ 4.64, 4.56, 4.32, 4.28, 4.26, 3.68, 3.54, 3.32, 2.86, 2.50, 2.27, 2.23, 1.77, 1.47, 1.44.

Figure S125: 4-((4-Chloro-5-((3-(2,3-dihydrobenzo[*b*][1,4]dioxin-6-yl)-2-methylbenzyl)oxy)-2-(((2-fluoroethyl)amino)methyl)phenoxy)methyl)picolinonitrile (**5i**):

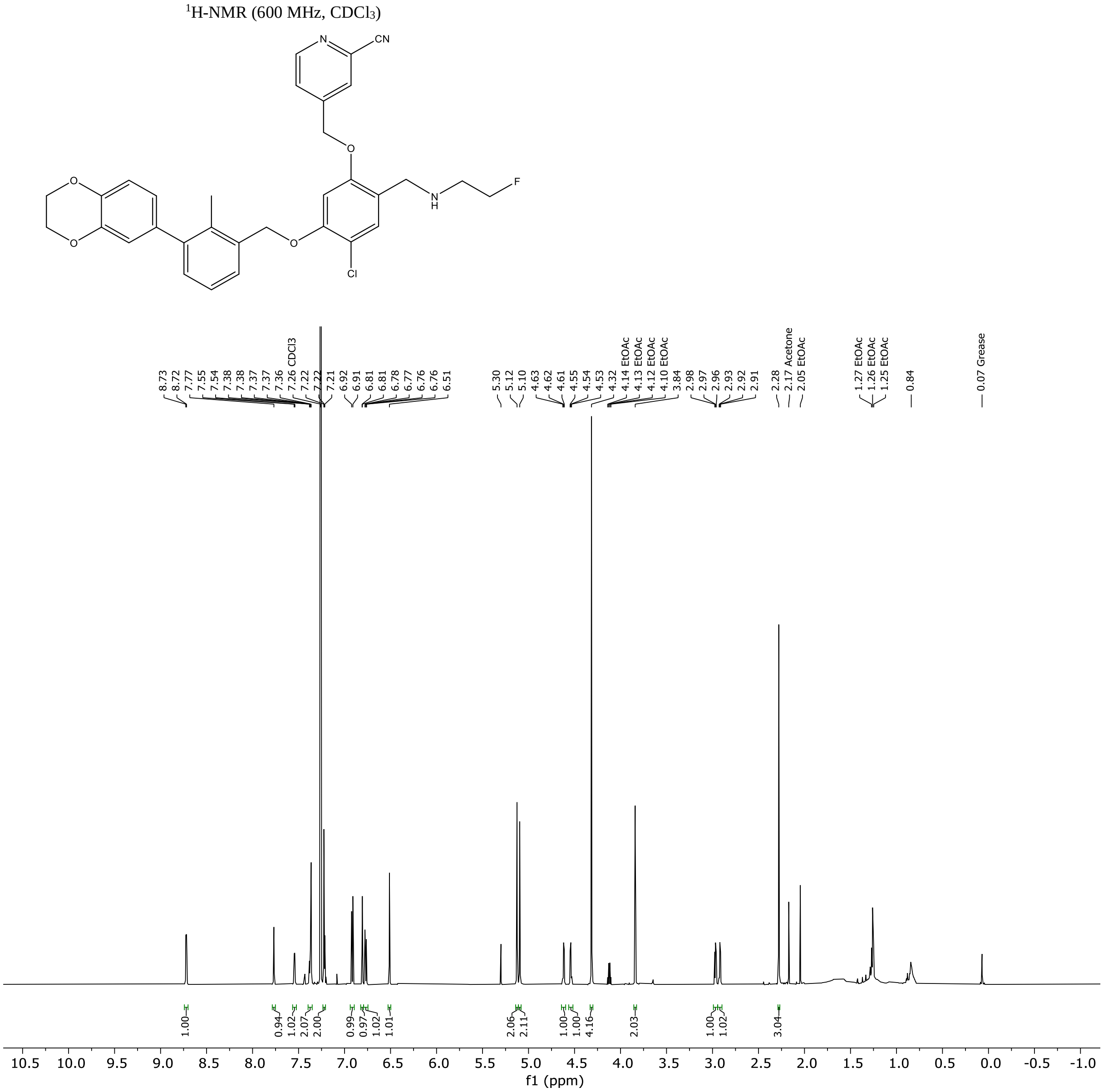

^{1}H-NMR (600 MHz, $CDCl_3$): δ 8.72 (d, *J* = 5.1 Hz, 1H), 7.77 (s, 1H), 7.54 (d, *J* = 5.1 Hz, 1H), 7.37 (dd, *J* = 6.0 Hz, *J* = 2.9 Hz, 1H), 7.36 (s, 1H), 7.22 – 7.21 (m, 2H), 6.92 (d, *J* = 8.2 Hz, 1H), 6.81 (d, *J* = 2.1 Hz, 1H), 6.77 (dd, *J* = 8.2 Hz, *J* = 2.1 Hz, 1H), 6.51 (s, 1H), 5.12 (s, 2H), 5.10 (s, 2H), 4.58 (dt, *J* = 48 Hz, *J* = 4.7 Hz, 2H), 4.32 (s, 4H), 3.84 (s, 2H), 3.95 (dt, *J* = 29 Hz, *J* = 4.7 Hz, 2H), 2.28 (s, 3H).

Figure S126: 4-((4-Chloro-5-((3-(2,3-dihydrobenzo[*b*][1,4]dioxin-6-yl)-2-methylbenzyl)oxy)-2-(((2-fluoroethyl)amino)methyl)phenoxy)methyl)picolinonitrile (**5i**):

^{13}C-NMR (151 MHz, $CDCl_3$)

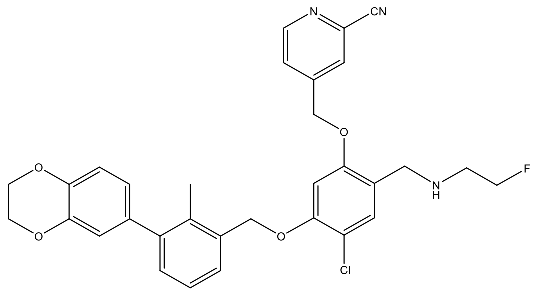

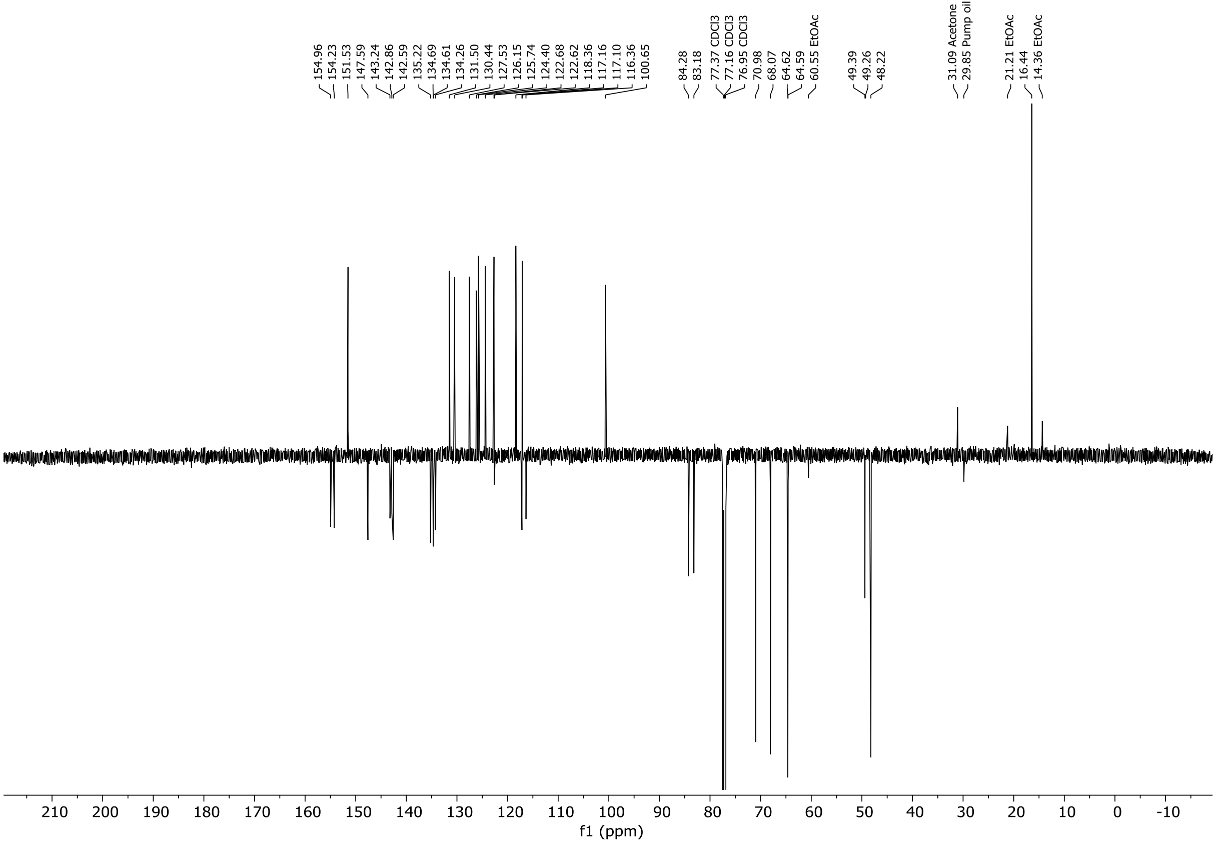

^{13}C-NMR (151 MHz, $CDCl_3$): δ 154.96, 154.23, 151.53, 147.59, 143.24, 142.86, 142.59, 135.22, 134.69, 134.61, 134.26, 131.50, 130.44, 127.53, 126.15, 125.74, 124.40, 122.68, 122.62, 118.36, 117.16, 117.10, 116.36, 100.65, 84.28, 83.18, 70.98, 68.07, 64.62, 64.59, 49.39, 49.26, 48.22, 16.44.

Figure S127: 4-((4-Chloro-5-((3-(2,3-dihydrobenzo[*b*][1,4]dioxin-6-yl)-2-methylbenzyl)oxy)-2-(((2-fluoroethyl)amino)methyl)phenoxy)methyl)picolinonitrile (**5i**):

^{1}H-^{1}H COSY NMR (600 MHz, $CDCl_3$)

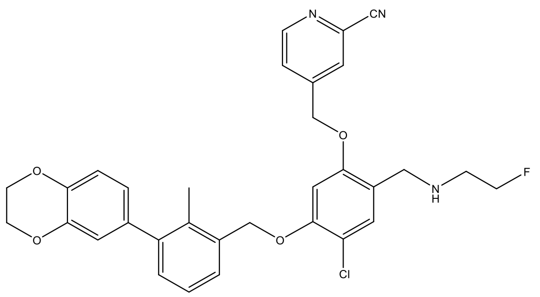

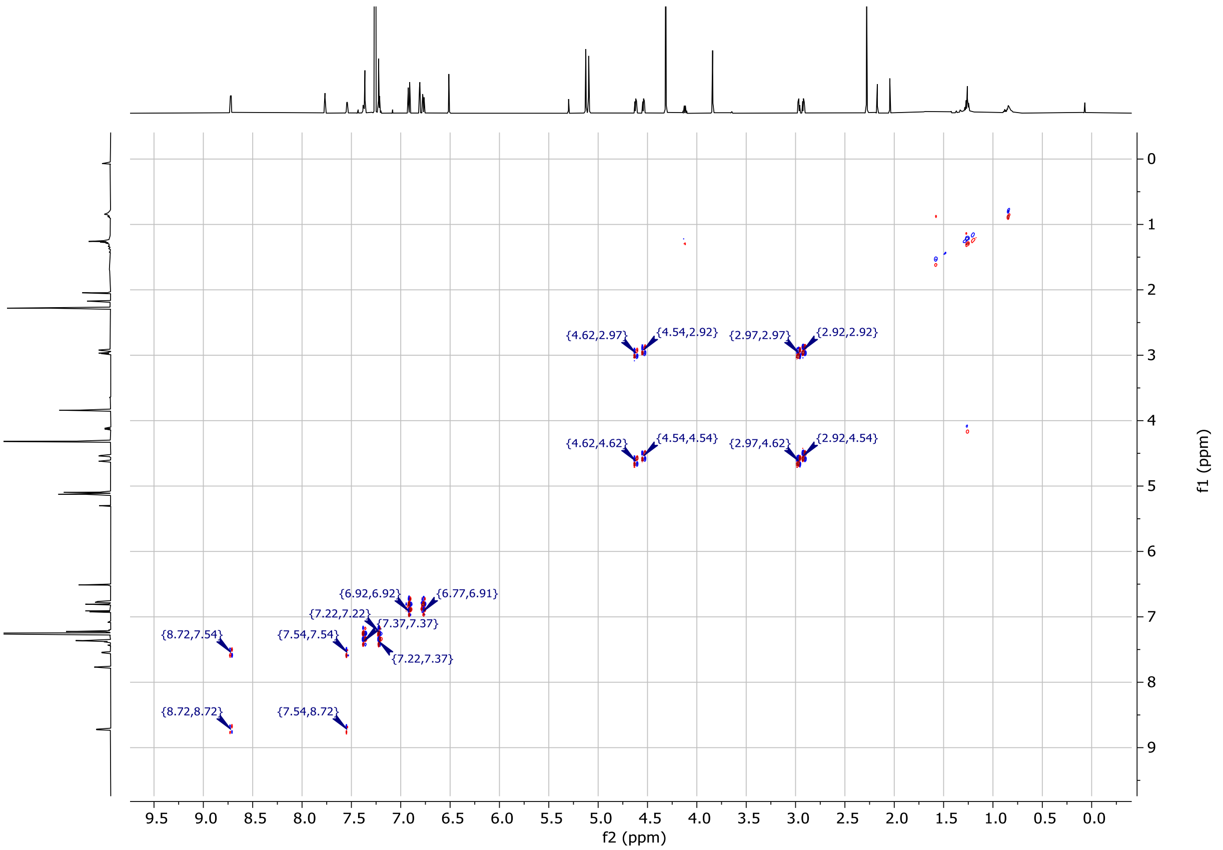

^{1}H-NMR (600 MHz, $CDCl_3$): δ 8.72, 8.72, 7.54, 7.54, 7.37, 7.37, 7.22, 7.22, 6.92, 6.91, 6.77, 6.77, 4.62, 4.62, 4.54, 4.54, 2.97, 2.97, 2.92, 2.92.

^{1}H-NMR (600 MHz, $CDCl_3$): δ 8.72, 8.72, 7.54, 7.54, 7.37, 7.37, 7.22, 7.22, 6.92, 6.91, 6.77, 6.77, 4.62, 4.62, 4.54, 4.54, 2.97, 2.97, 2.92, 2.92.

Figure S128: 4-((4-Chloro-5-((3-(2,3-dihydrobenzo[*b*][1,4]dioxin-6-yl)-2-methylbenzyl)oxy)-2-(((2-fluoroethyl)amino)methyl)phenoxy)methyl)picolinonitrile (**5i**):

^{1}H-^{13}C HSQC NMR (600 MHz, 151 MHz, $CDCl_3$)

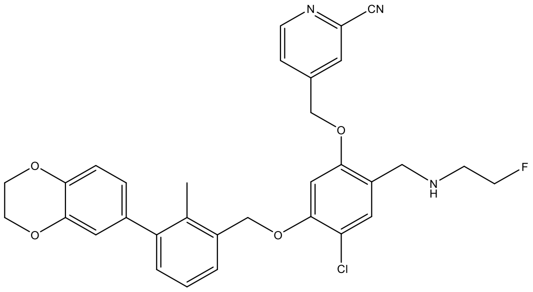

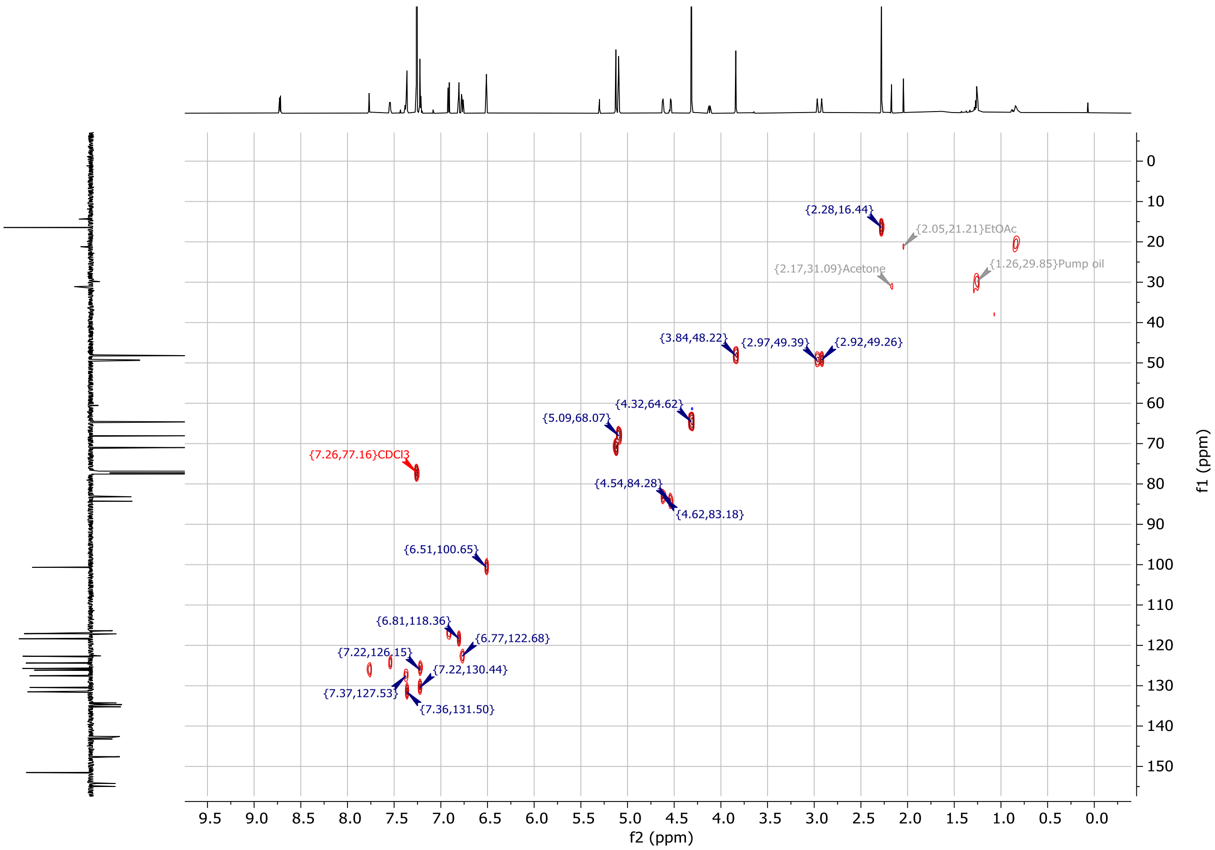

^{13}C-NMR (151 MHz, $CDCl_3$): δ 131.50, 130.44, 127.53, 126.15, 125.74, 124.40, 122.68, 118.36, 117.10, 100.65, 84.28, 83.18, 68.07, 64.62, 49.39, 49.26, 48.22, 16.44.

^{1}H-NMR (600 MHz, $CDCl_3$): δ 7.77, 7.54, 7.37, 7.36, 7.22, 7.22, 6.92, 6.81, 6.77, 6.51, 5.09, 4.62, 4.54, 4.32, 3.84, 2.97, 2.92, 2.28.

Figure S129: 2-Fluoroethyl (2-acetamidoethyl)(5-chloro-2-((3-cyanobenzyl)oxy)-4-((3-(2,3-dihydrobenzo[*b*][1,4]dioxin-6-yl)-2-methylbenzyl)oxy)benzyl)carbamate (**5j**):

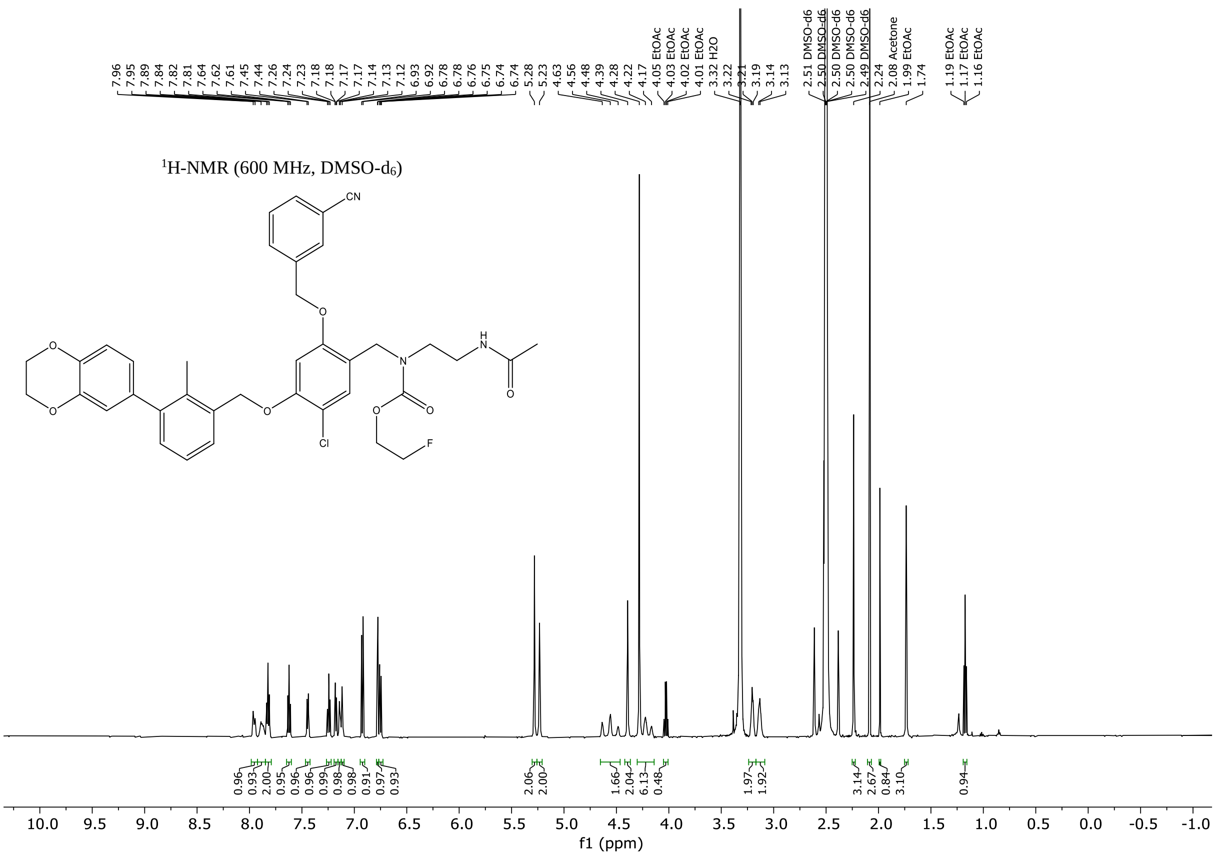

^{1}H-NMR (600 MHz, DMSO-d_6): δ 7.95 (d, *J* = 11 Hz, 1H), 7.89 (br s, 1H), 7.82 (m, 2H), 7.62 (t, *J* = 7.7 Hz, 1H), 7.44 (d, *J* = 7.5 Hz, 1H), 7.24 (t, *J* = 7.5 Hz, 1H), 7.18 (dd, *J* = 7.7 Hz, *J* = 1.4 Hz, 1H), 7.14 (d, *J* = 5.8 Hz, 1H), 7.12 (s, 1H), 6.92 (d, *J* = 8.2 Hz, 1H), 6.78 (d, *J* = 2.1 Hz, 1H), 6.75 (dd, *J* = 8.2 Hz, *J* = 2.1 Hz, 1H), 5.28 (s, 2H), 5.23 (s, 2H), 4.56 (m, *J* = 45 Hz, 2H), 4.39 (s, 2H), 4.28 (s, 4H), 4.22 (m, *J* = 35 Hz, 2H), 3.21 (m, 2H), 3.13 (m, 2H), 2.24 (s, 3H), 1.74 (s, 3H).

Figure S130: 2-Fluoroethyl (2-acetamidoethyl)(5-chloro-2-((3-cyanobenzyl)oxy)-4-((3-(2,3-dihydrobenzo[*b*][1,4]dioxin-6-yl)-2-methylbenzyl)oxy)benzyl)carbamate (**5j**):

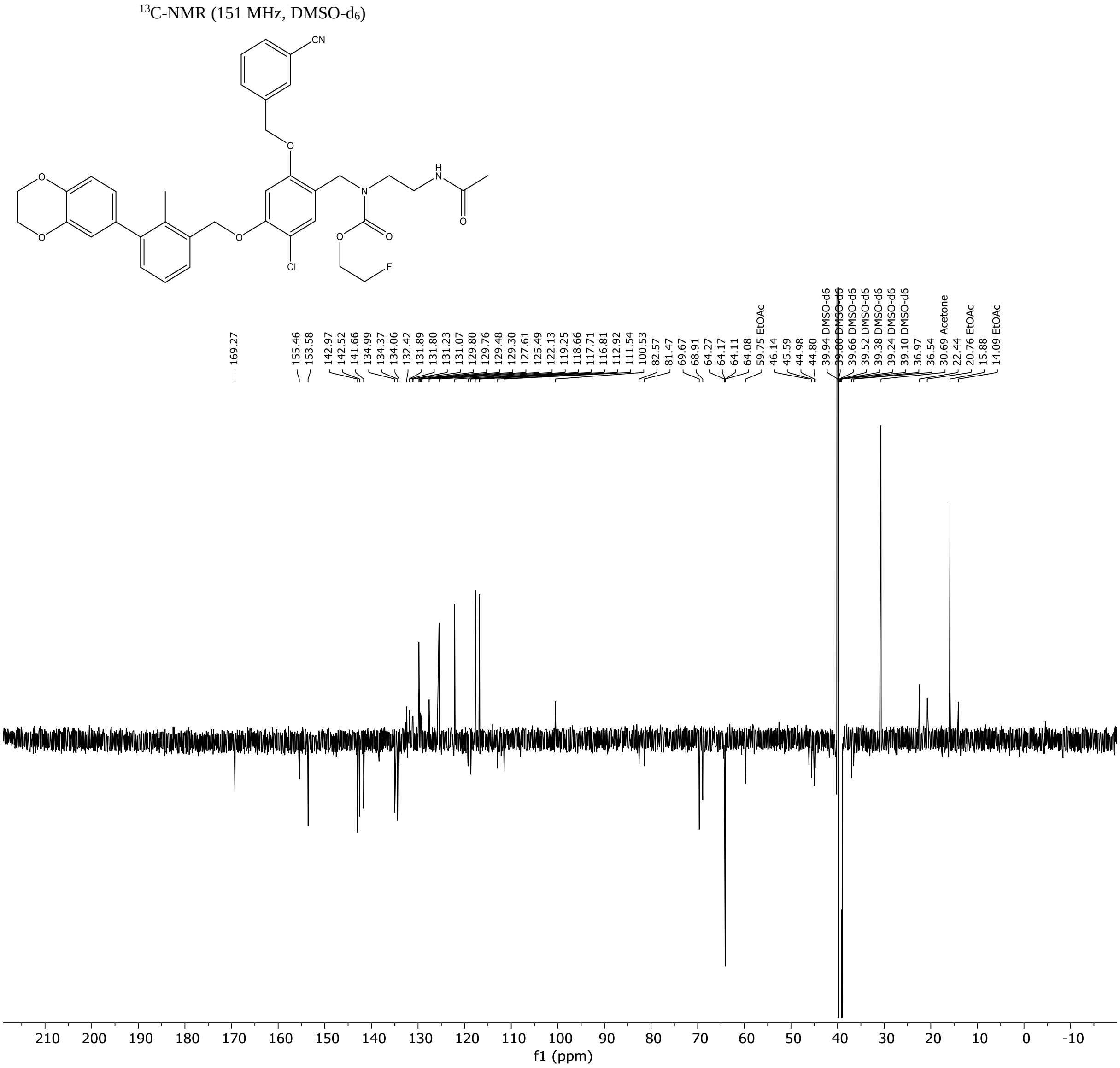

^{13}C-NMR (151 MHz, DMSO-d$_6$): δ 169.27, 155.46, 153.58, 142.97, 142.52, 141.66, 134.99, 134.37, 134.06, 132.42, 131.84 (J = 13 Hz), 131.15 (J = 25 Hz), 129.80, 129.76, 129.48, 129.30, 127.61, 125.49, 122.13, 119.25, 118.66, 117.71, 116.81, 112.92, 111.54, 100.53, 81.97 (J = 165 Hz), 81.47, 69.67, 68.91, 64.22 (J = 16 Hz), 64.11, 64.08, 45.87 (J = 82 Hz), 44.89 (J = 27 Hz), 36.76 (J = 64 Hz), 22.44, 15.88.

Figure S131: 2-Fluoroethyl (2-acetamidoethyl)(5-chloro-2-((3-cyanobenzyl)oxy)-4-((3-(2,3-dihydrobenzo[*b*][1,4]dioxin-6-yl)-2-methylbenzyl)oxy)benzyl)carbamate (**5j**):

^{1}H-^{1}H COSY NMR (600 MHz, DMSO-d$_6$)

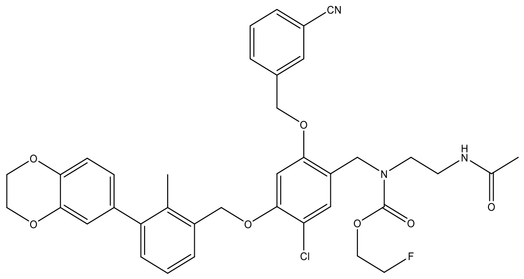

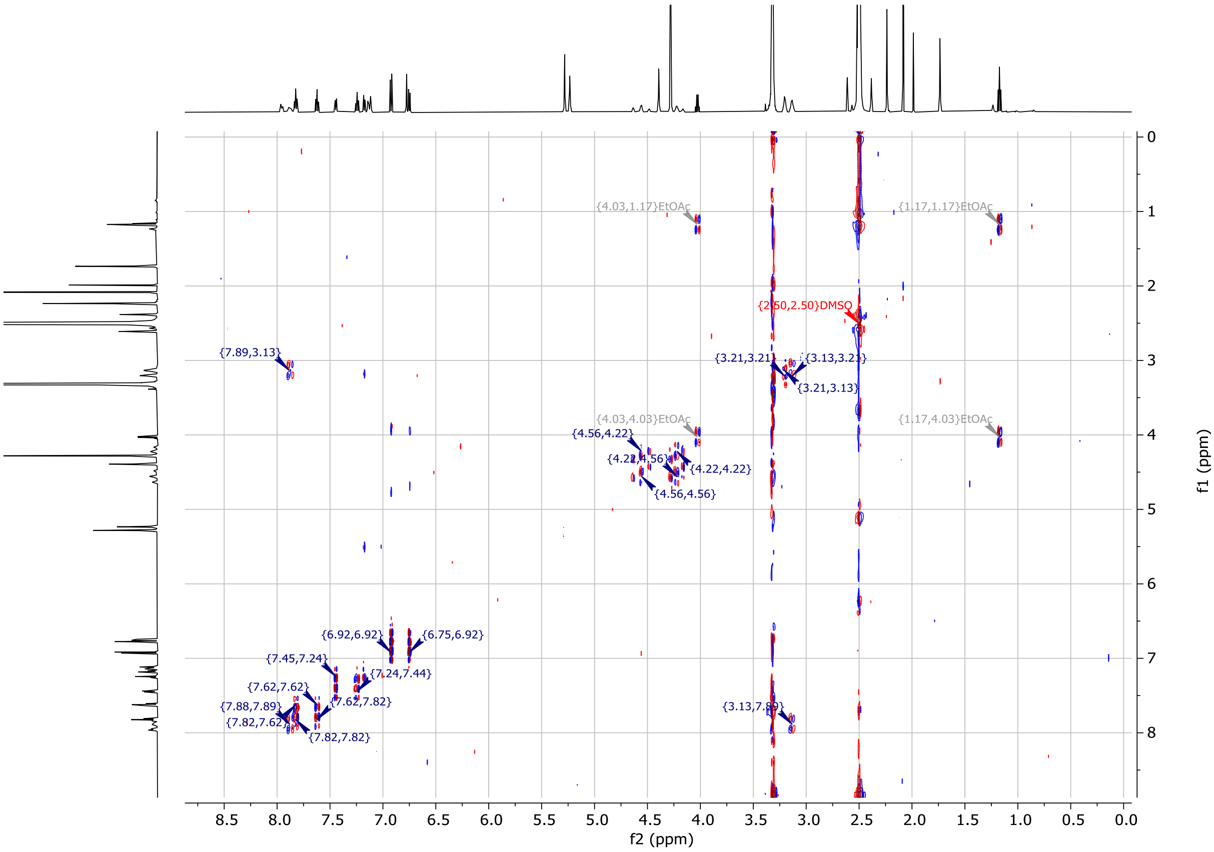

^{1}H-NMR (600 MHz, DMSO-d$_6$): δ 7.89, 7.89, 7.82, 7.82, 7.82, 7.62, 7.62, 7.45, 7.44, 7.24, 7.24, 7.18, 6.92, 6.92, 6.75, 6.75, 4.56, 4.56, 4.22, 4.22, 3.21, 3.21, 3.20, 3.13, 3.13, 3.13.

^{1}H-NMR (600 MHz, DMSO-d$_6$): δ 7.90, 7.90, 7.89, 7.88, 7.82, 7.82, 7.62, 7.62, 7.45, 7.45, 7.24, 7.24, 7.18, 6.92, 6.92, 6.75, 6.75, 4.56, 4.56, 4.22, 4.22, 3.21, 3.21, 3.13, 3.13, 3.13.

Figure S132: 2-Fluoroethyl (2-acetamidoethyl)(5-chloro-2-((3-cyanobenzyl)oxy)-4-((3-(2,3-dihydrobenzo[*b*][1,4]dioxin-6-yl)-2-methylbenzyl)oxy)benzyl)carbamate (**5j**):

^{1}H-^{13}C HSQC NMR (600 MHz, 151 MHz, DMSO-d_6)

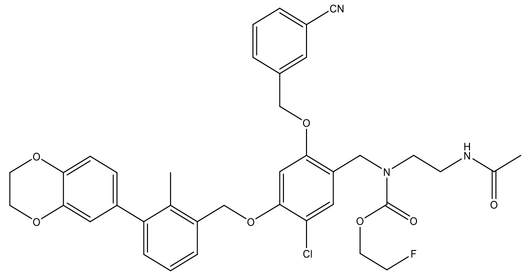

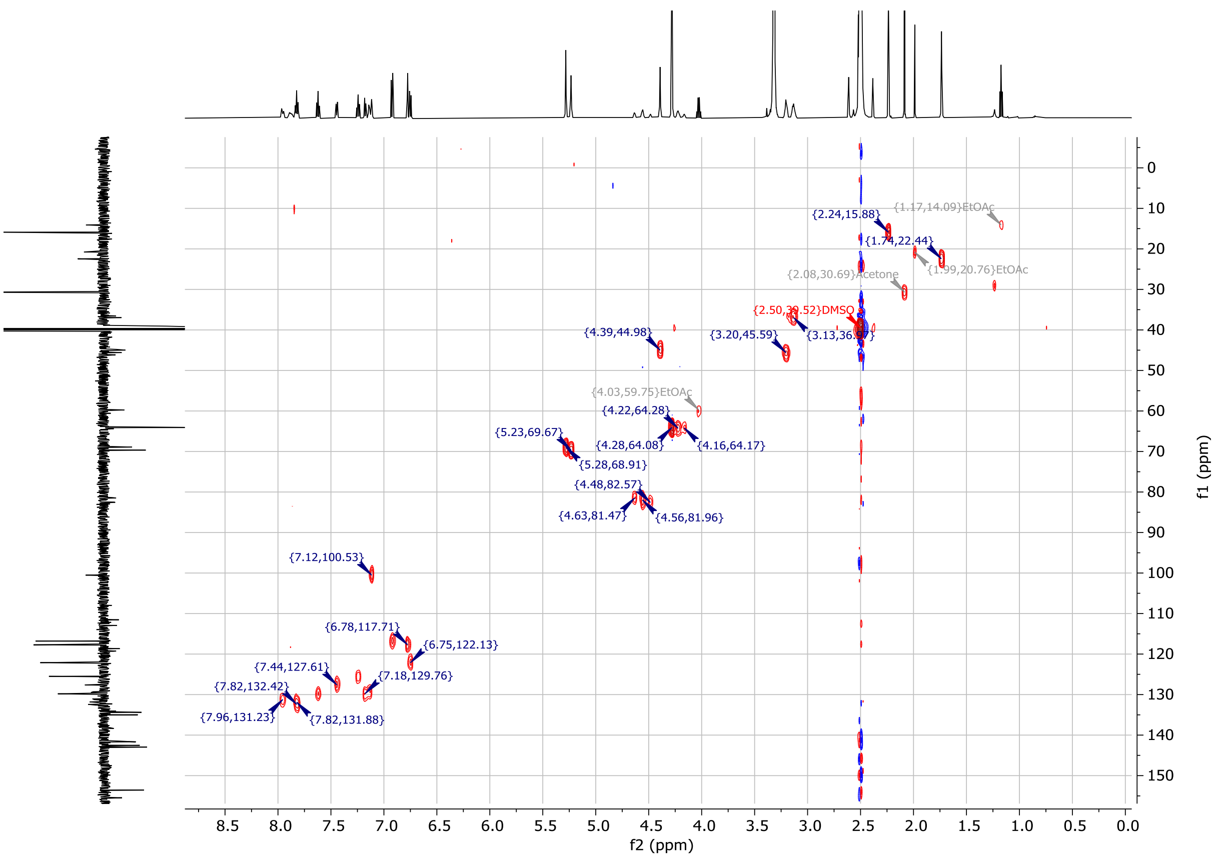

^{13}C-NMR (151 MHz, DMSO-d_6): δ 132.42, 131.88, 131.23, 129.80, 129.76, 129.48, 129.30, 127.61, 125.49, 122.13, 117.71, 116.81, 100.53, 82.57, 81.96, 81.47, 69.67, 68.91, 68.91, 64.28, 64.17, 64.17, 64.08, 59.75, 45.59, 44.98, 44.80, 39.52, 36.97, 36.97, 30.69, 22.44, 20.76, 15.88, 14.09.

^{1}H-NMR (600 MHz, DMSO-d_6): δ 7.96, 7.82, 7.82, 7.62, 7.44, 7.24, 7.18, 7.15, 7.14, 7.12, 6.92, 6.78, 6.75, 5.28, 5.26, 5.23, 4.63, 4.56, 4.48, 4.39, 4.38, 4.28, 4.27, 4.22, 4.16, 4.03, 3.20, 3.14, 3.13, 2.50, 2.24, 2.08, 1.99, 1.74, 1.17.

Figure S133: 2-Fluoroethyl (2-acetamidoethyl)(5-chloro-2-((3-cyanobenzyl)oxy)-4-((3-(2,3-dihydrobenzo[*b*][1,4]dioxin-6-yl)-2-methylbenzyl)oxy)benzyl)carbamate (**5j**):

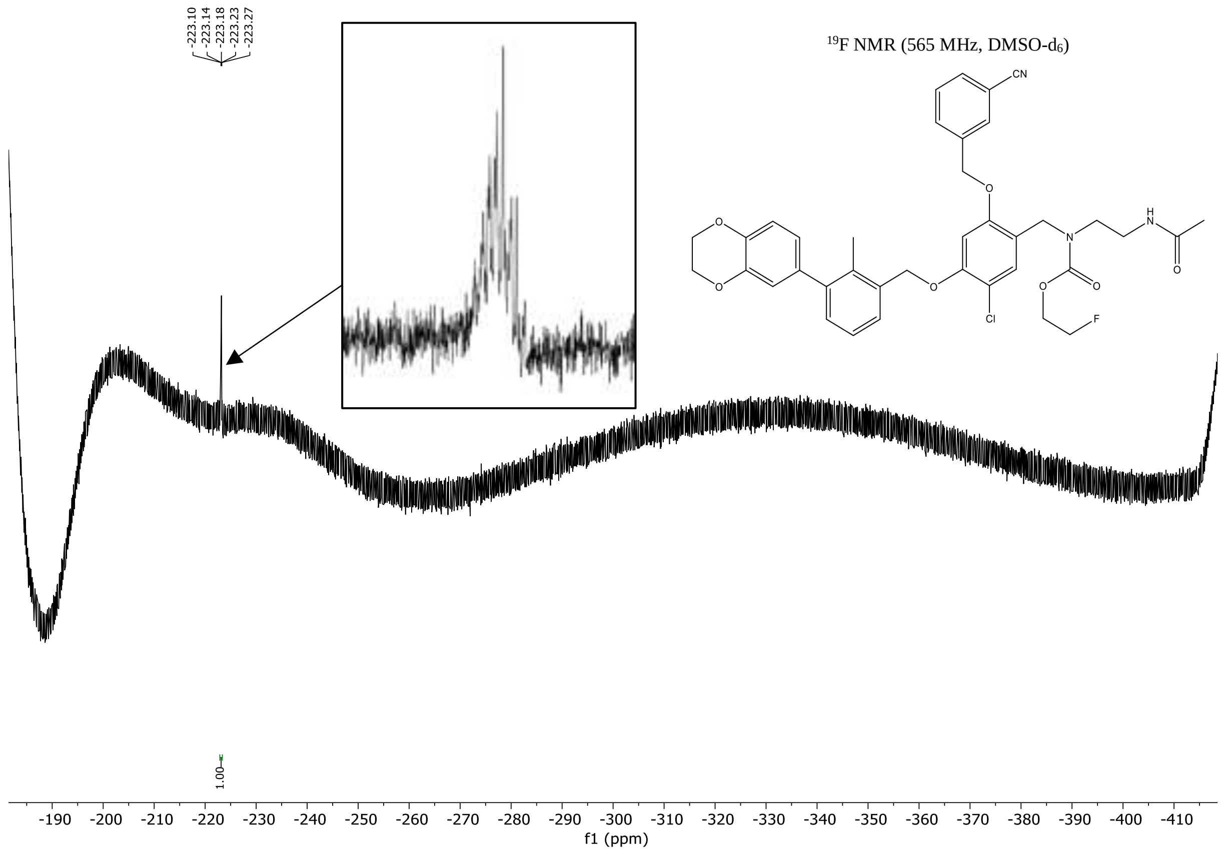

^{19}F-NMR (565 MHz, DMSO-d$_6$): δ -223.10 – -223.27 (m, 1F).

Mass spectrometry

Figure S134: (3-(2,3-Dihydrobenzo[*b*][1,4]dioxin-6-yl)-2-methylphenyl)methanol (**1b**):

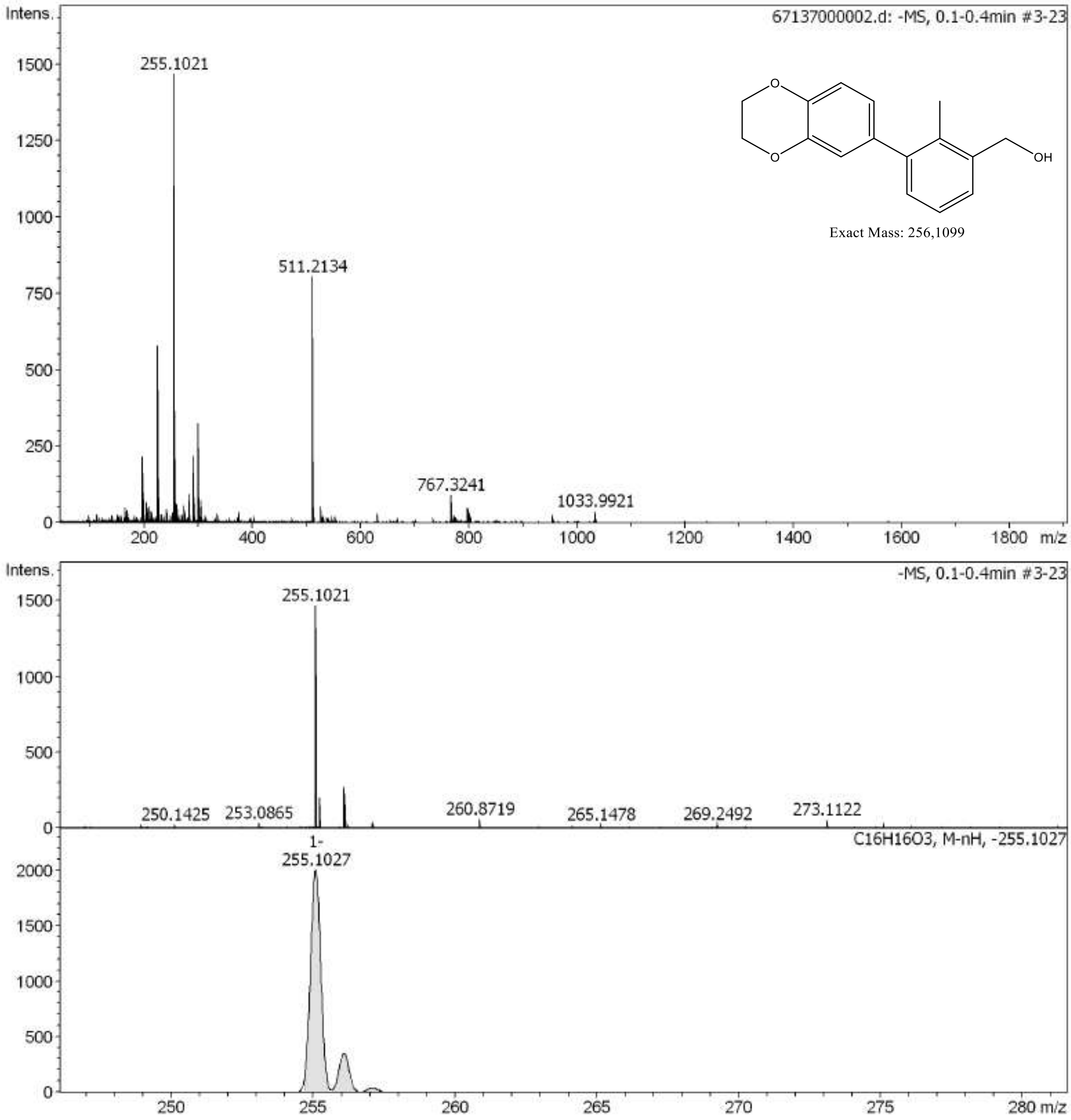

Figure S135: (2-Methyl-3-(1*H*-pyrrol-1-yl)phenyl)methanol (**1c**):

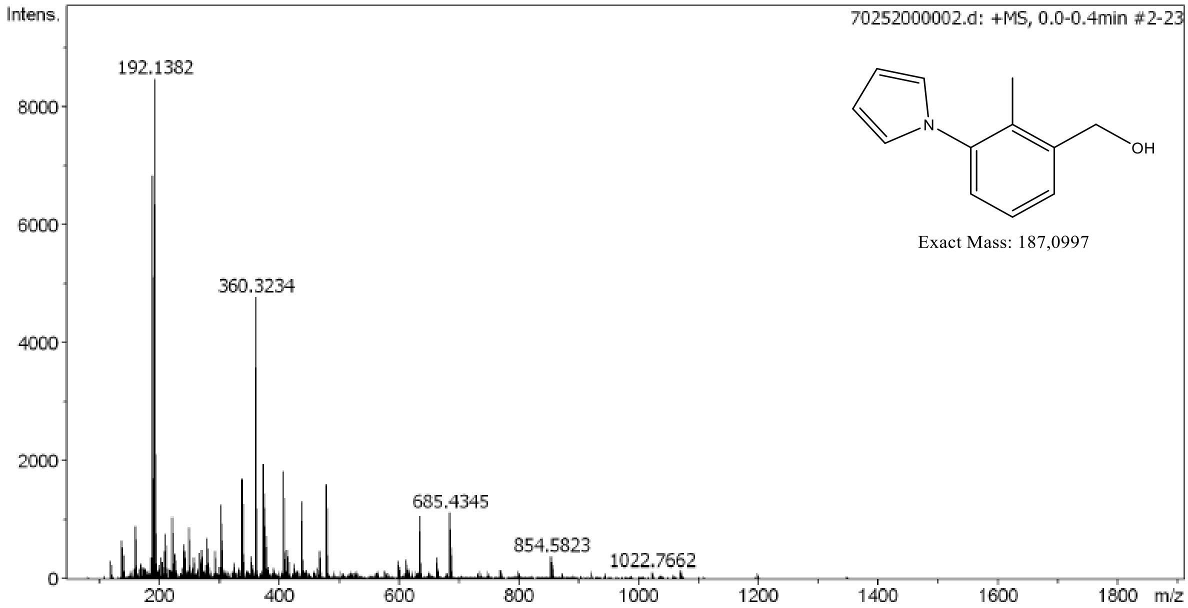

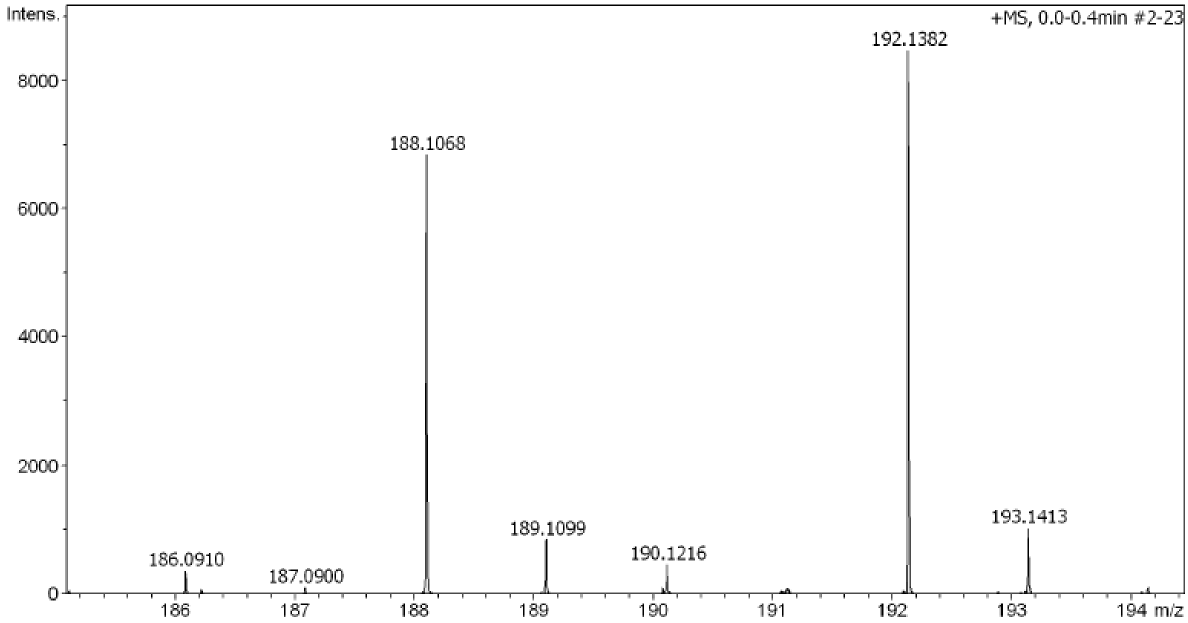

Figure S136: 5-Chloro-6-((2-methyl-[1,1'-biphenyl]-3-yl)methoxy)nicotinaldehyde (**2a**):

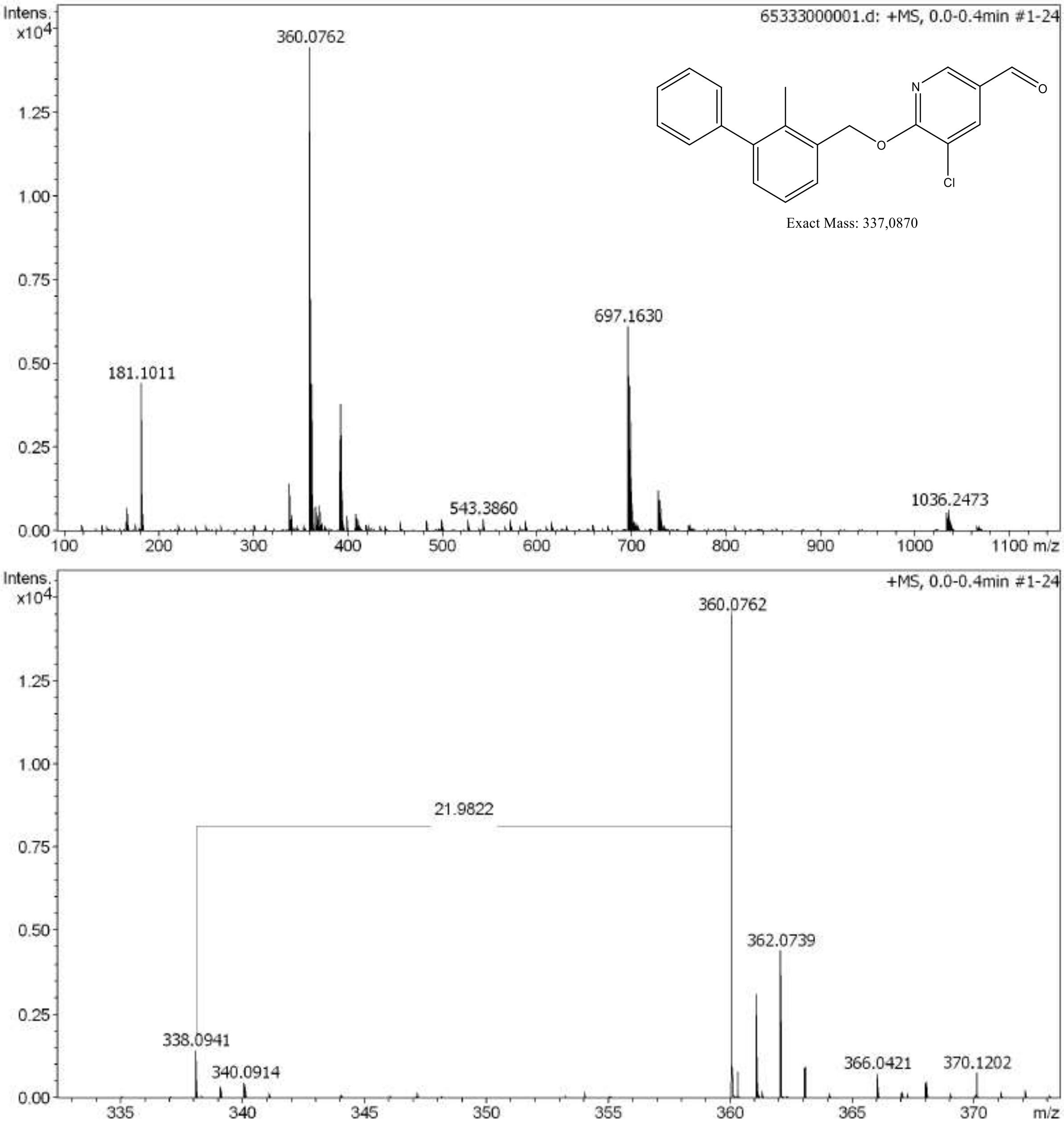

Figure S137: 5-Chloro-2-hydroxy-4-((2-methyl-[1,1'-biphenyl]-3-yl)methoxy)benzaldehyde (**2b**):

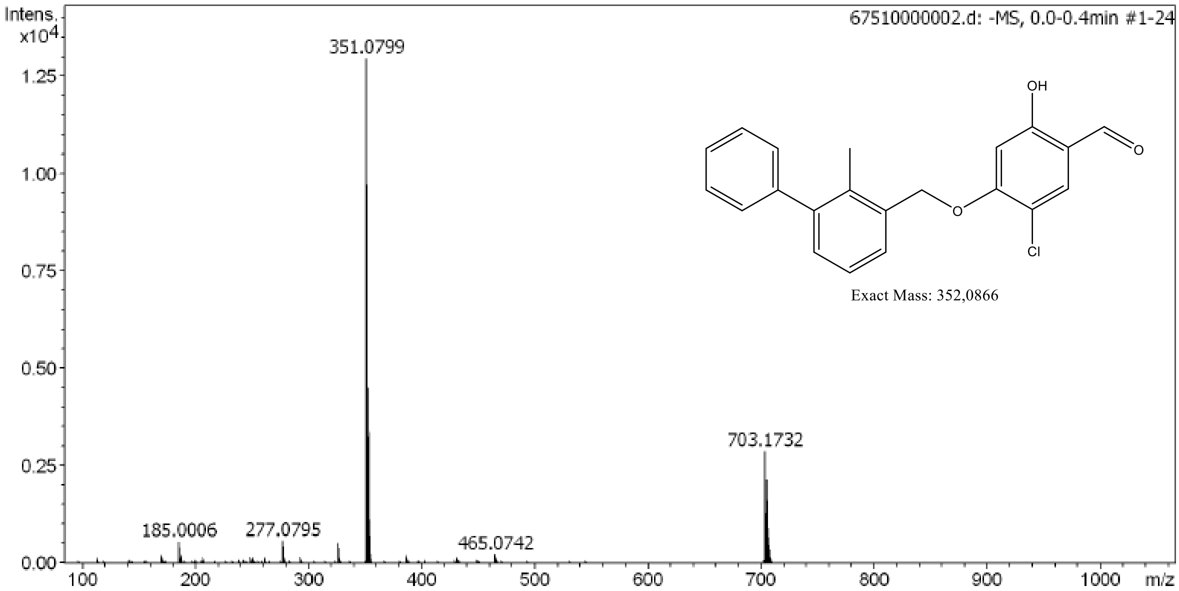

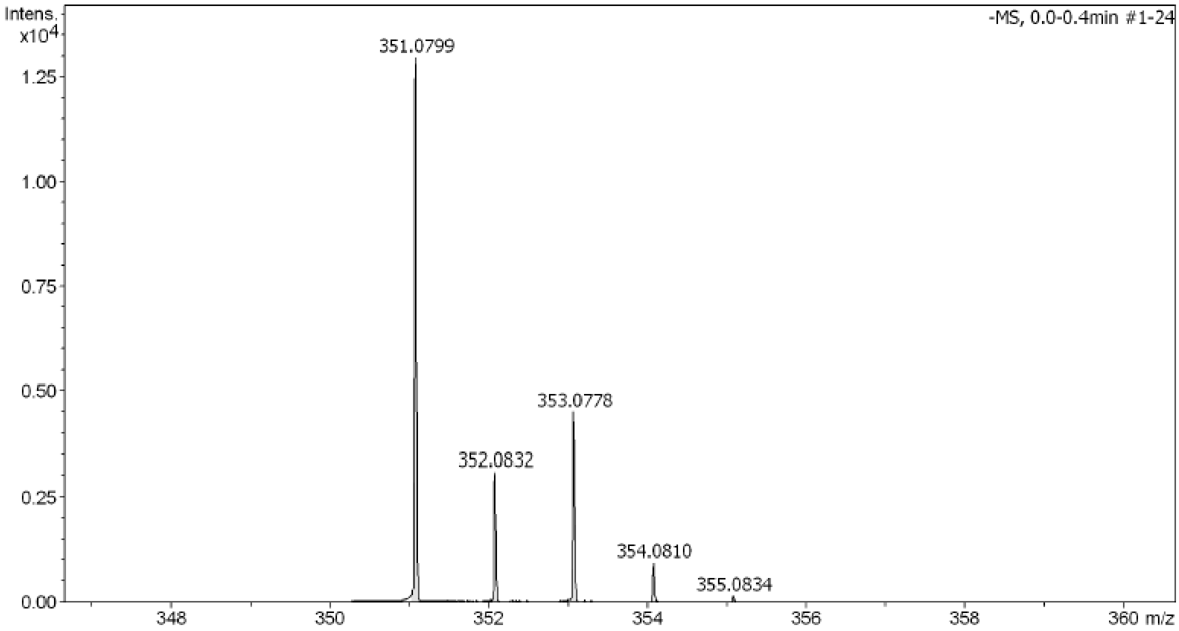

Figure S138: 5-Chloro-4-((3-(2,3-dihydrobenzo[*b*][1,4]dioxin-6-yl)-2-methylbenzyl)oxy)-2-hydroxybenzaldehyde (**2c**):

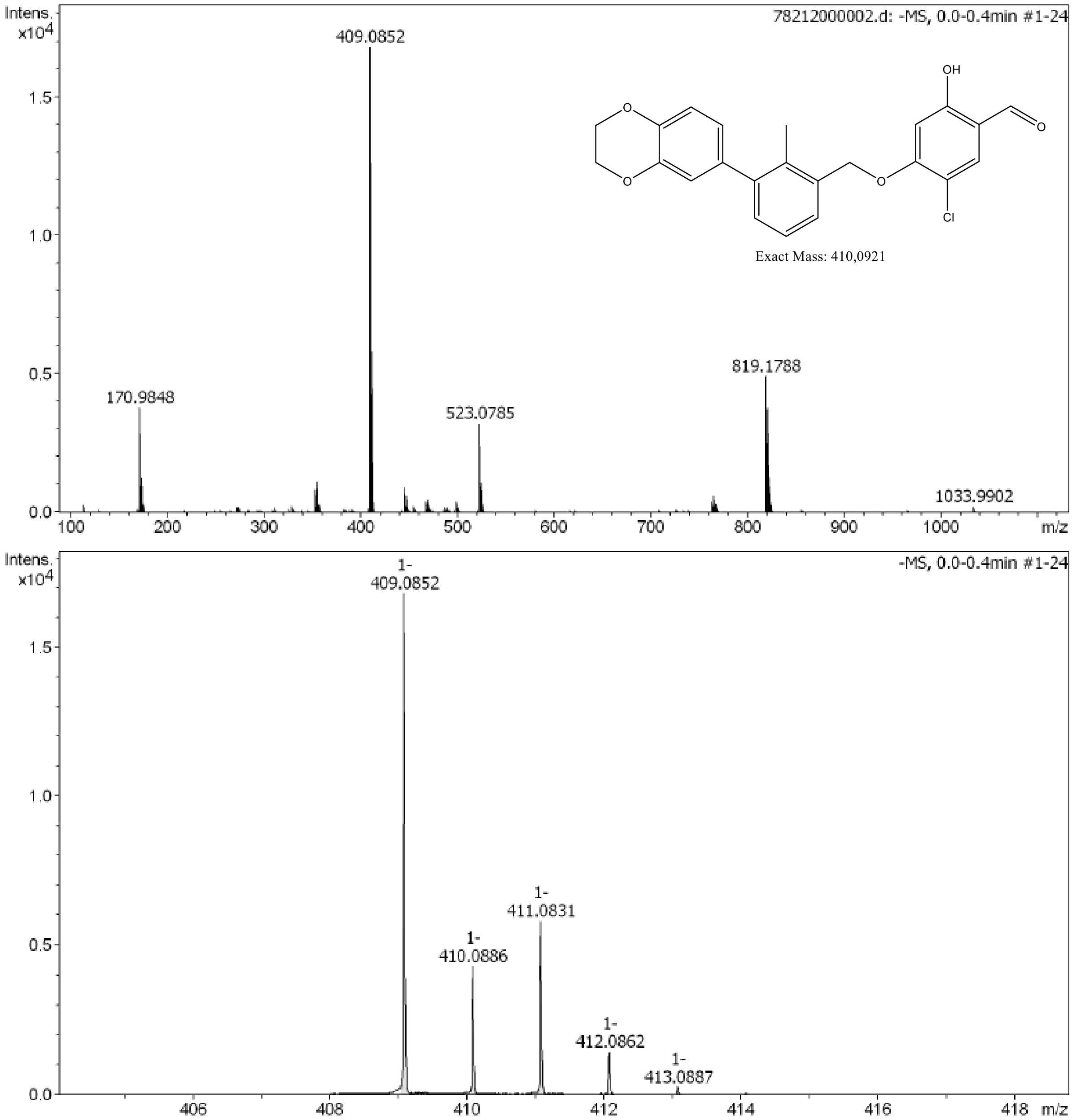

Figure S139: 5-Chloro-2-hydroxy-4-((2-methyl-3-(1*H*-pyrrol-1-yl)benzyl)oxy)benzaldehyde (**2d**):

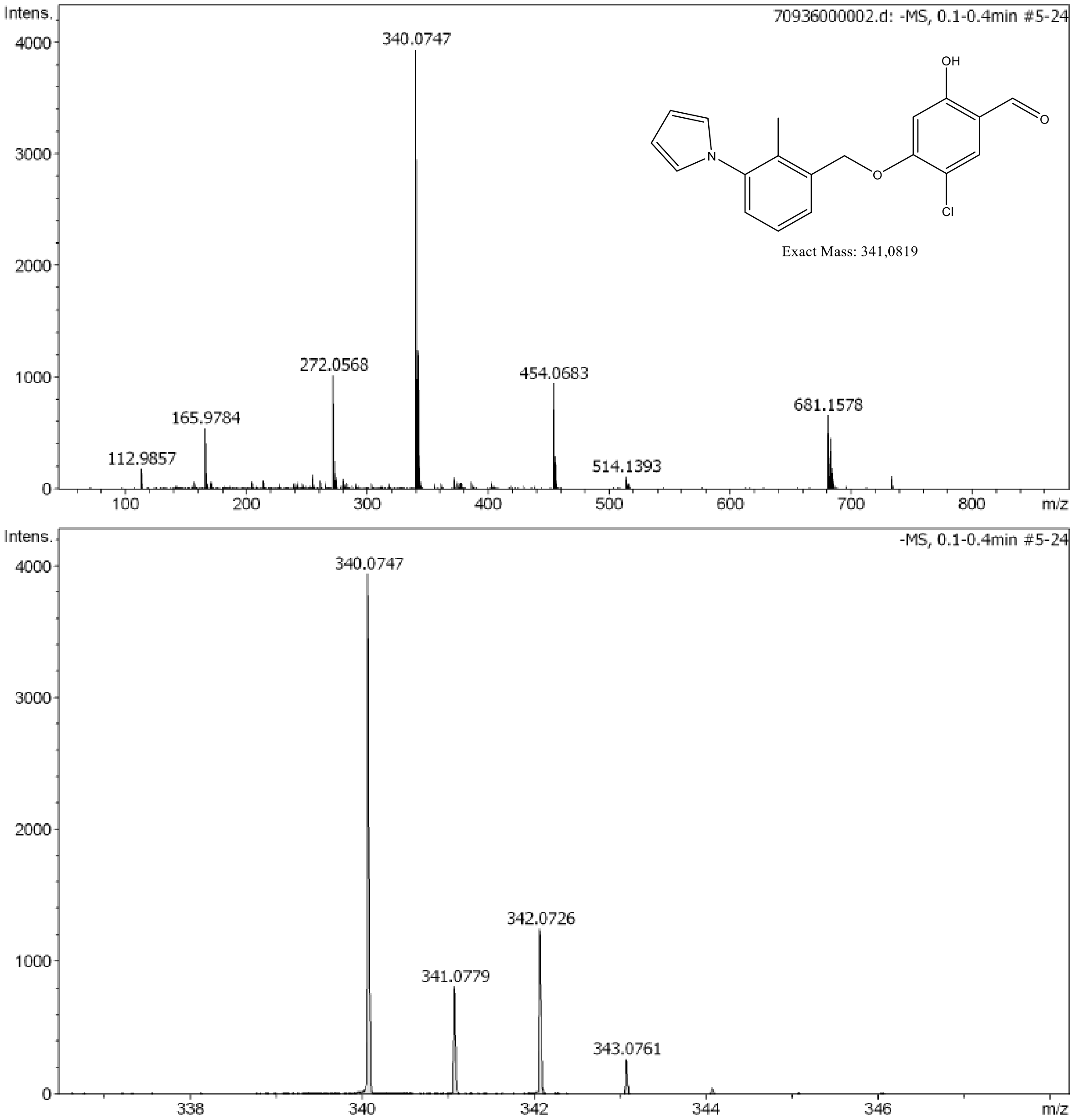

Figure S140: 3-((4-Chloro-2-formyl-5-((2-methyl-[1,1'-biphenyl]-3-yl)methoxy)phenoxy)methyl)benzonitrile (**3a**):

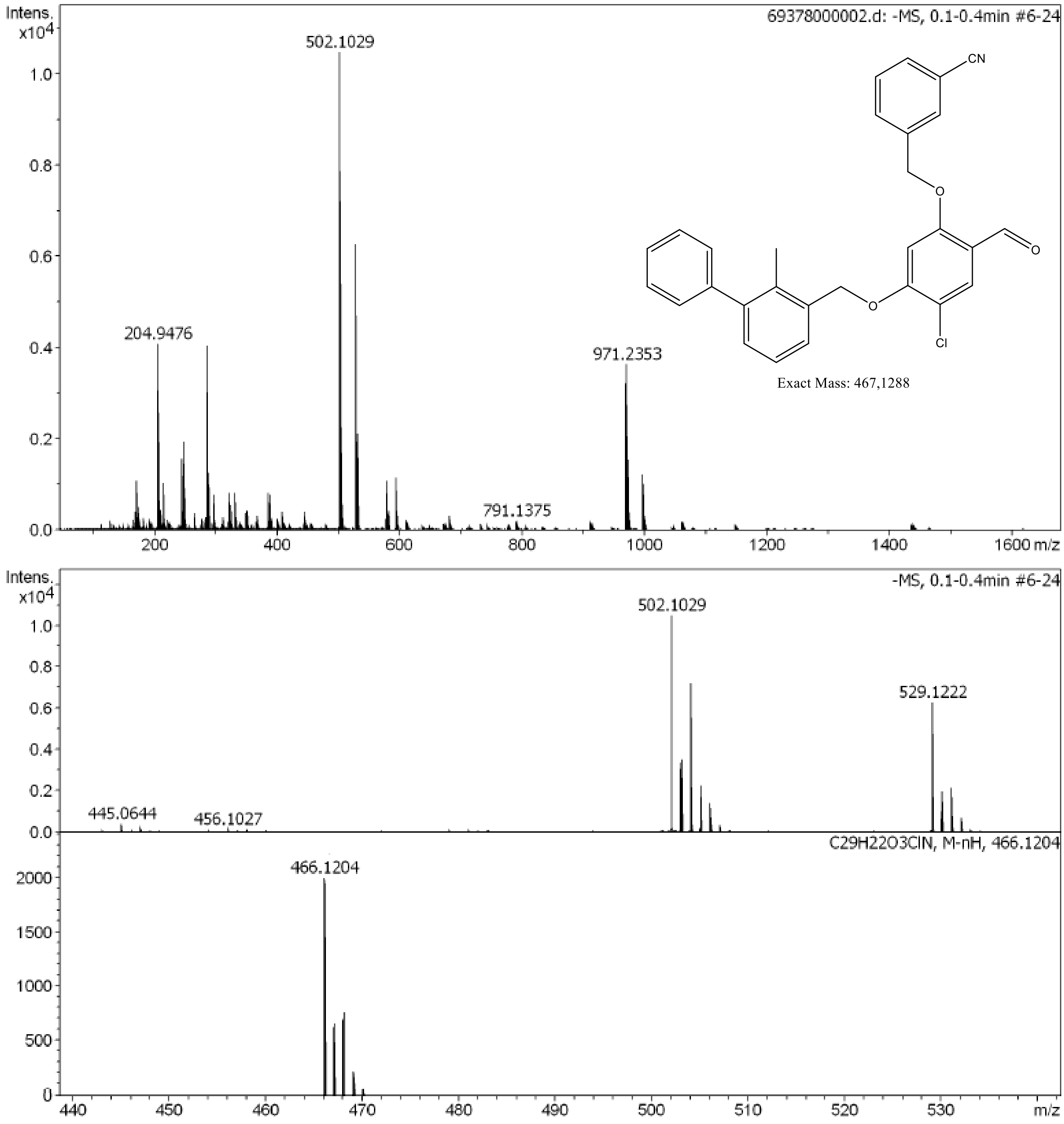

Figure S141: 4-((4-Chloro-5-((3-(2,3-dihydrobenzo[*b*][1,4]dioxin-6-yl)-2-methylbenzyl)oxy)-2-formylphenoxy)methyl)picolinonitrile (**3b**):

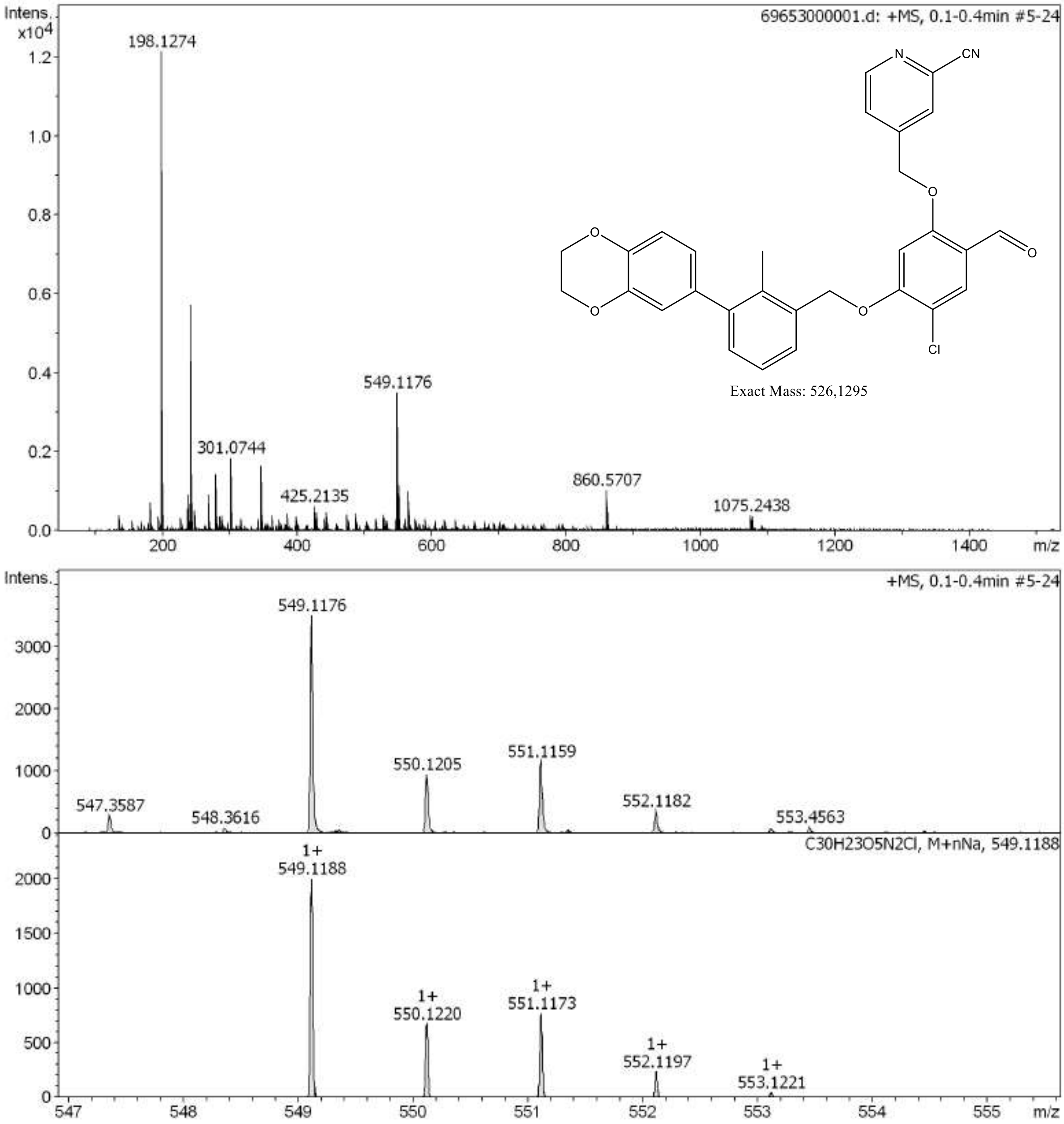

Figure S142: 3-((4-Chloro-5-((3-(2,3-dihydrobenzo[*b*][1,4]dioxin-6-yl)-2-methylbenzyl)oxy)-2-formylphenoxy)methyl)benzonitrile (**3c**):

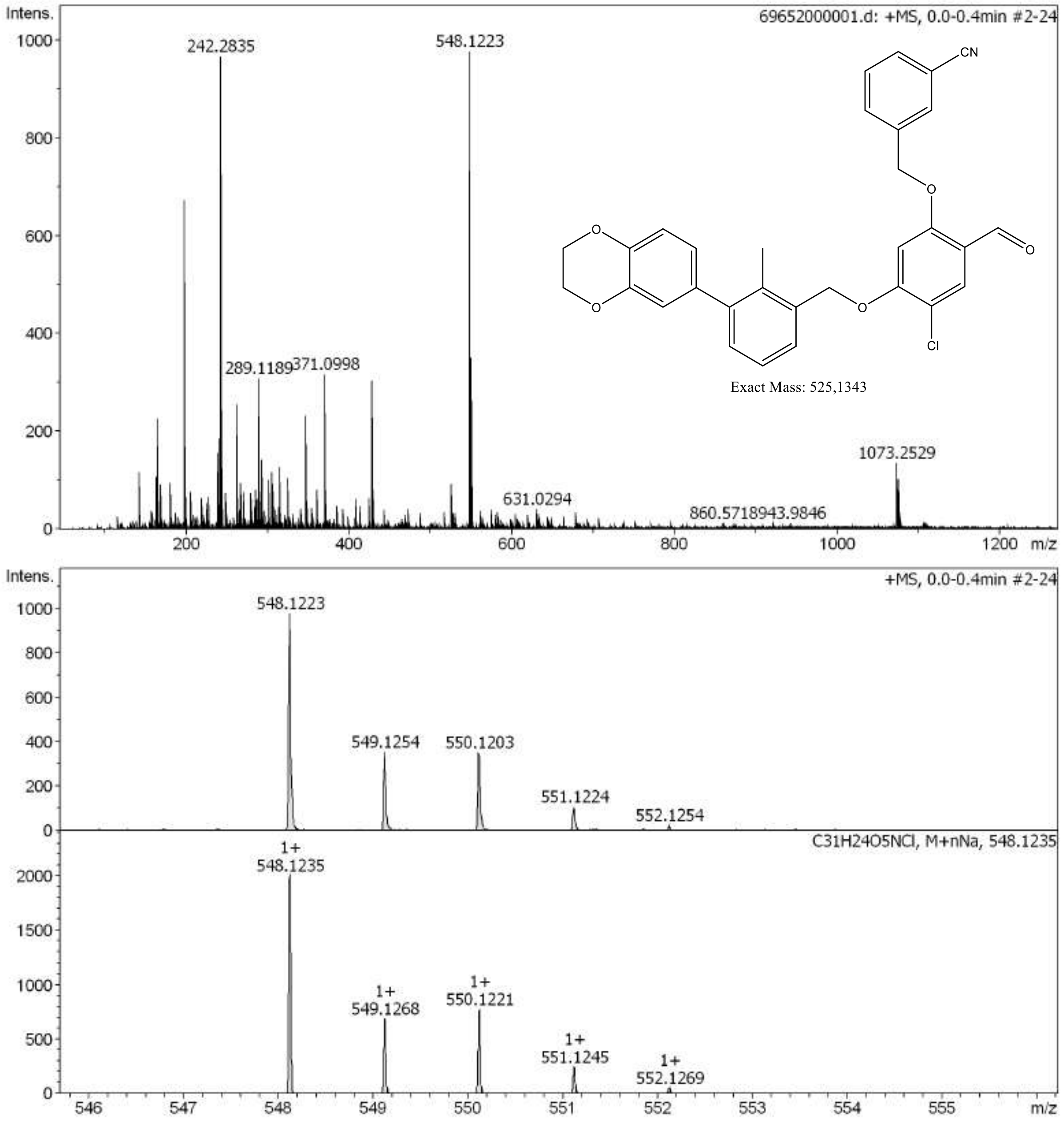

Figure S143: 5-Chloro-4-((3-(2,3-dihydrobenzo[*b*][1,4]dioxin-6-yl)-2-methylbenzyl)oxy)-2-(oxazol-4-ylmethoxy)benzaldehyde (**3d**):

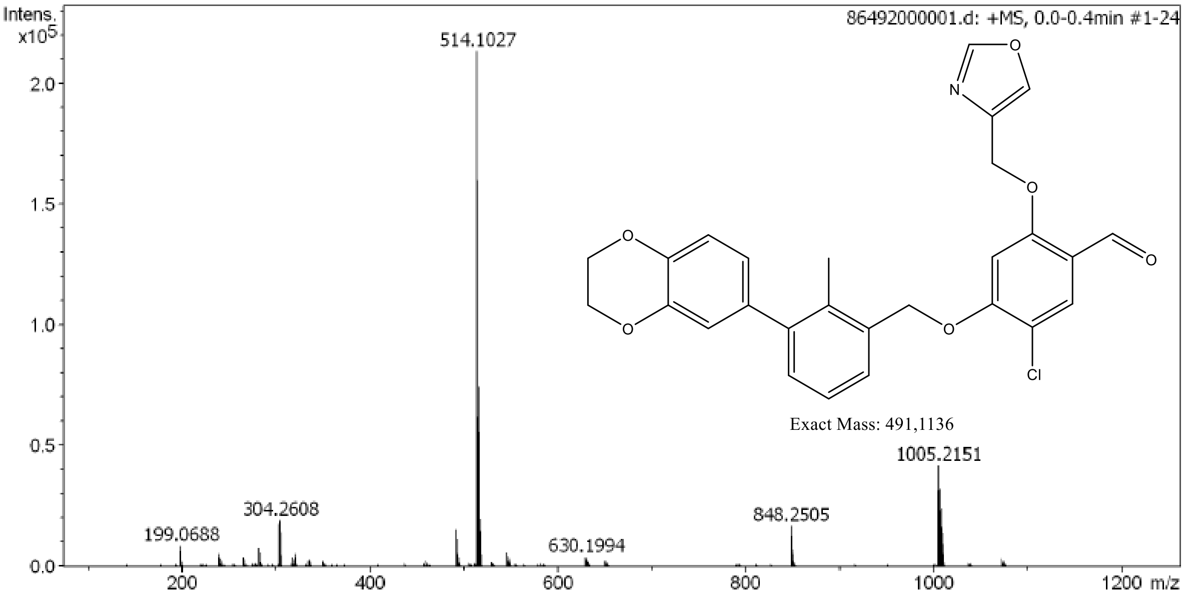

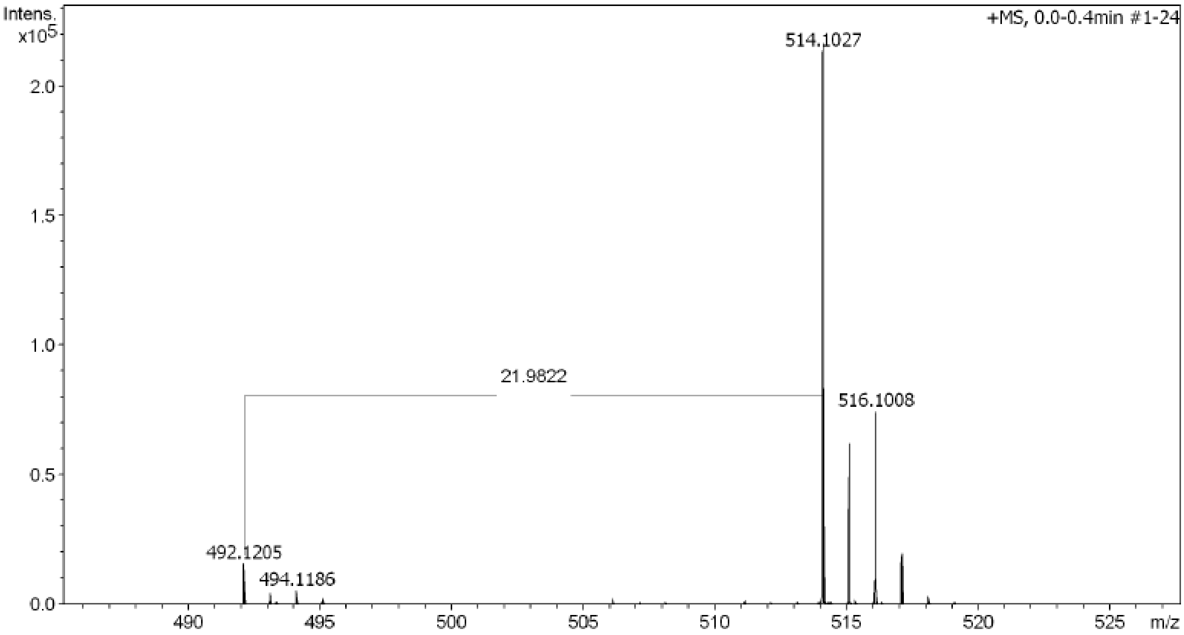

Figure S144: 4-((4-Chloro-2-formyl-5-((2-methyl-3-(1*H*-pyrrol-1-yl)benzyl)oxy)phenoxy)methyl)picolinonitrile (**3e**):

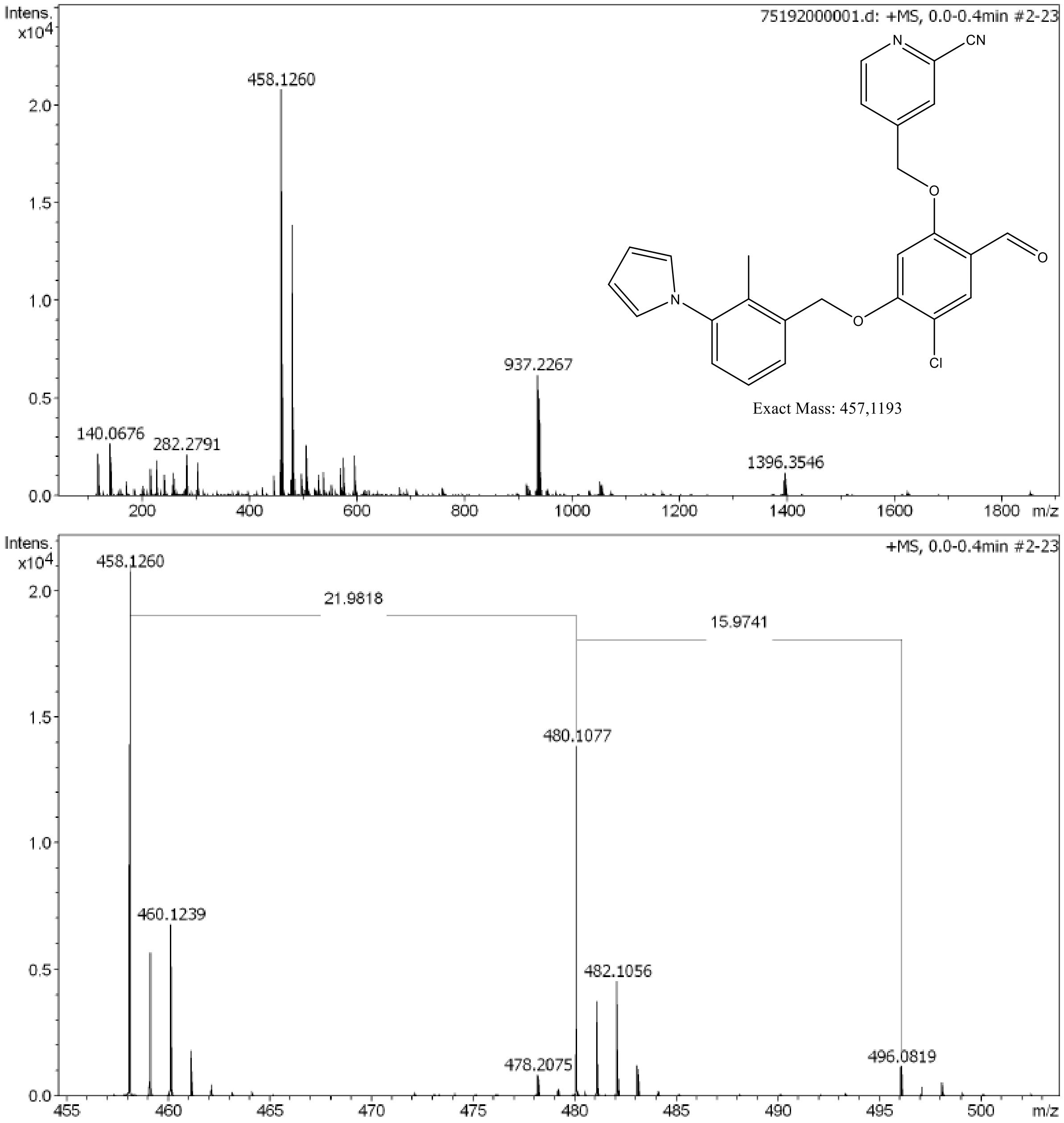

Figure S145: *N*-(2-(((5-chloro-6-((2-methyl-[1,1'-biphenyl]-3-yl)methoxy)pyridin-3-yl)methyl)amino)ethyl)acetamide (**4a**):

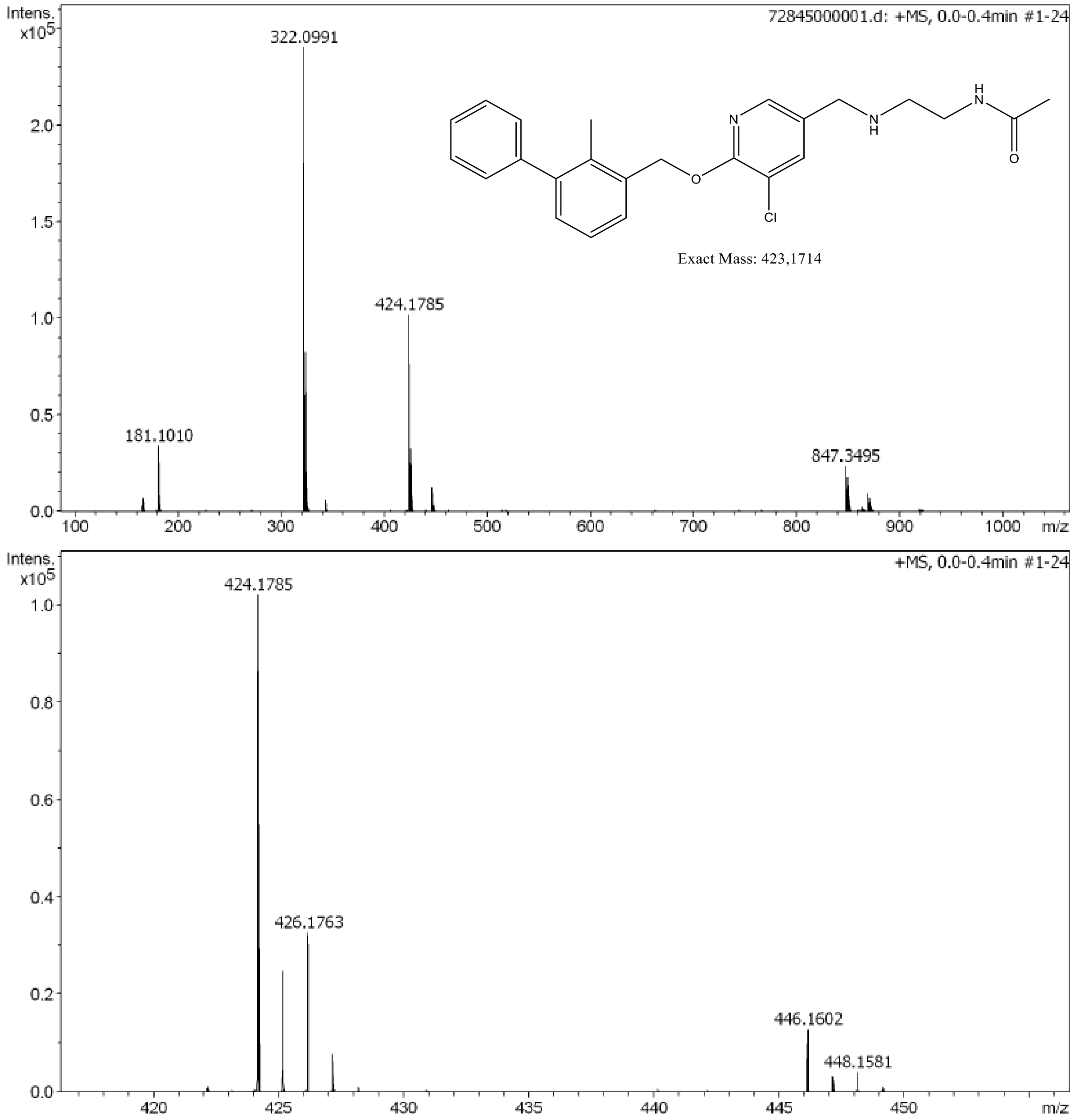

Figure S146: (5-Chloro-2-((3-cyanobenzyl)oxy)-4-((2-methyl-[1,1'-biphenyl]-3-yl)methoxy)benzyl)-*D*-serine (**4b**):

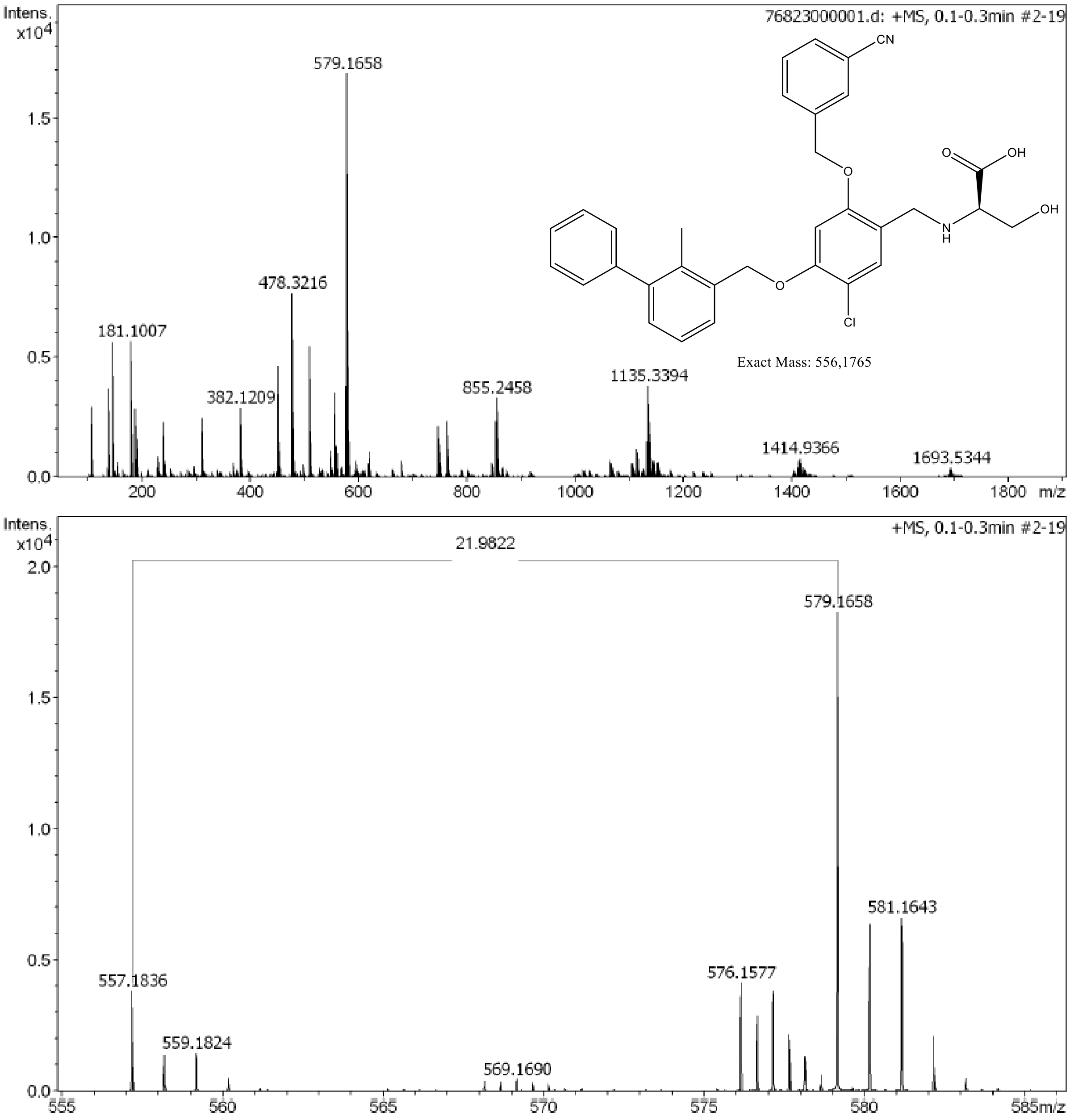

Figure S147: (5-Chloro-2-((2-cyanopyridin-4-yl)methoxy)-4-((3-(2,3-dihydrobenzo[*b*][1,4]dioxin-6-yl)-2-methylbenzyl)oxy)benzyl)-*D*-serine (**4c**):

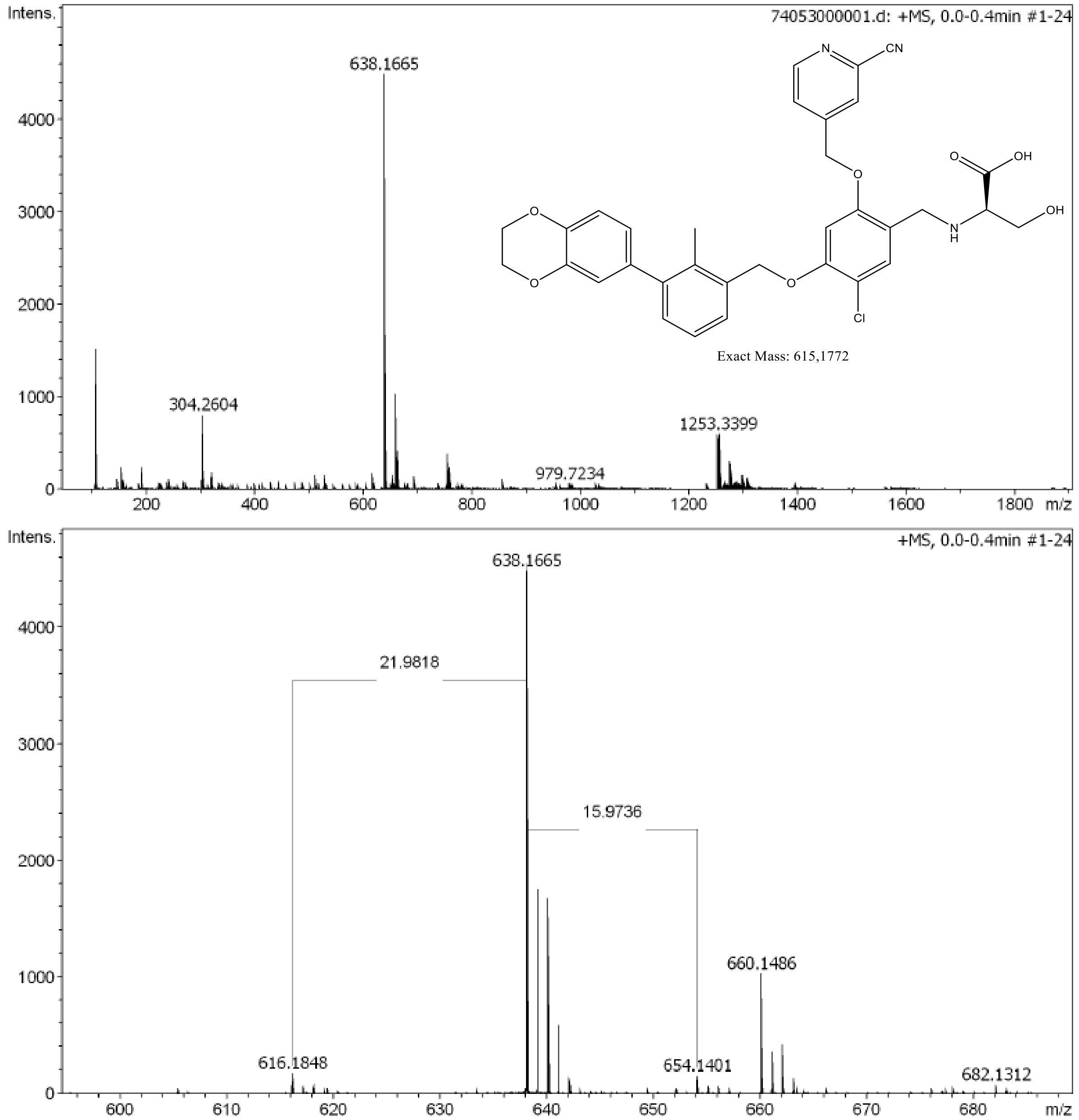

Figure S148: *N*-(2-((5-chloro-2-((2-cyanopyridin-4-yl)methoxy)-4-((3-(2,3-dihydrobenzo[*b*][1,4]dioxin-6-yl)-2-methylbenzyl)oxy)benzyl)amino)ethyl)acetamide (**4d**):

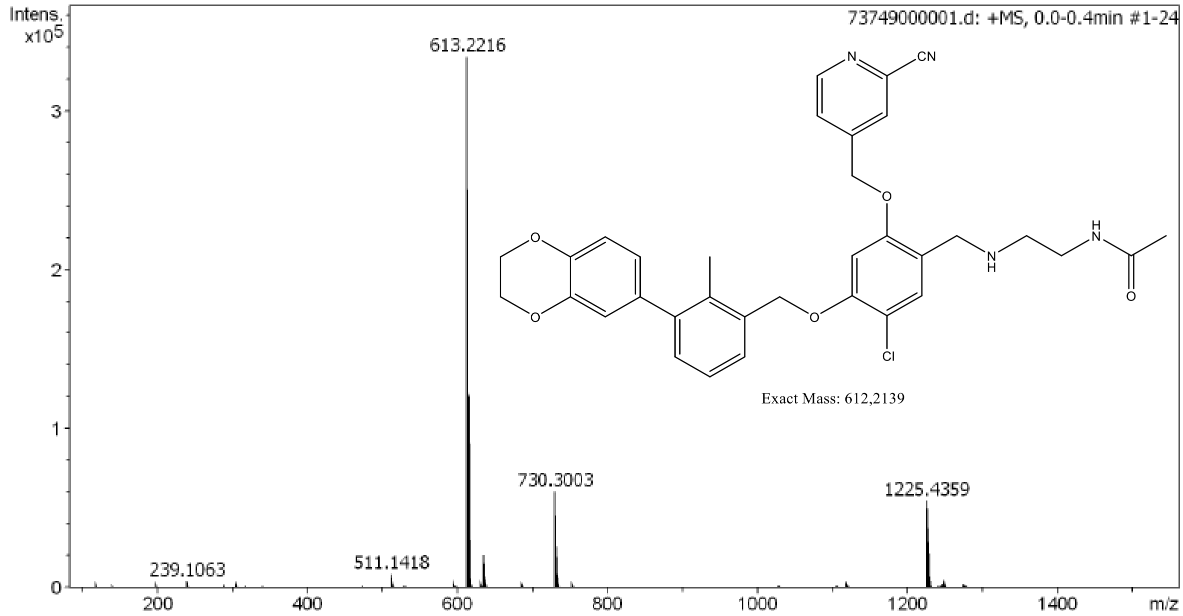

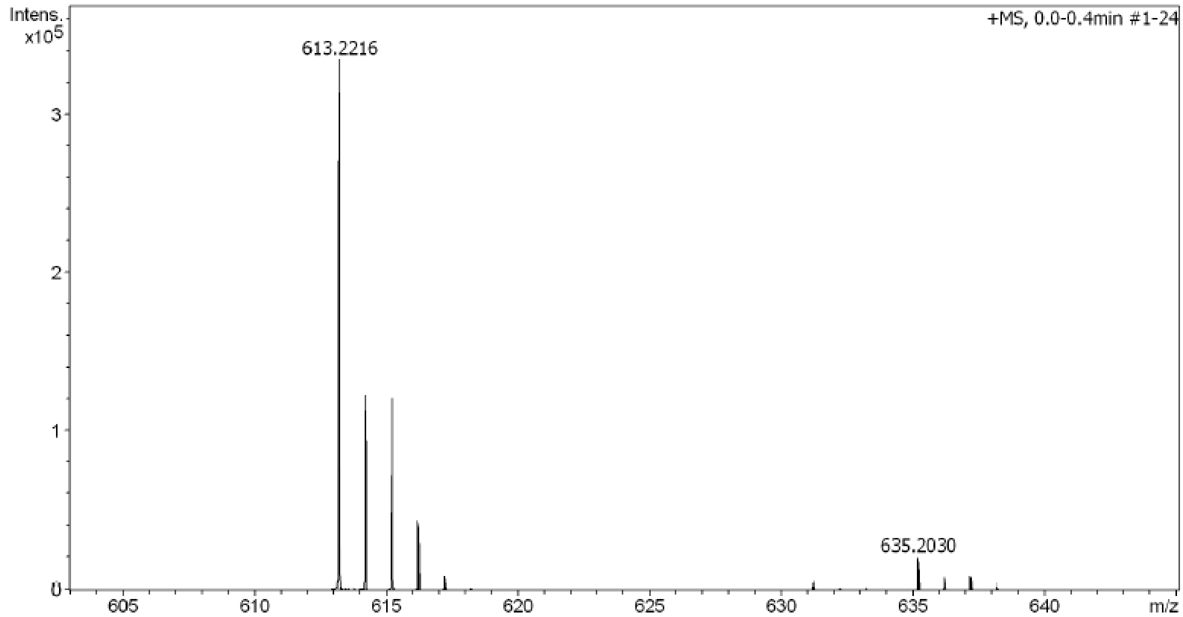

Figure S149: 4-((4-Chloro-5-((3-(2,3-dihydrobenzo[*b*][1,4]dioxin-6-yl)-2-methylbenzyl)oxy)-2-(((2-hydroxyethyl)amino)methyl)phenoxy)methyl)picolinonitrile (**4e**):

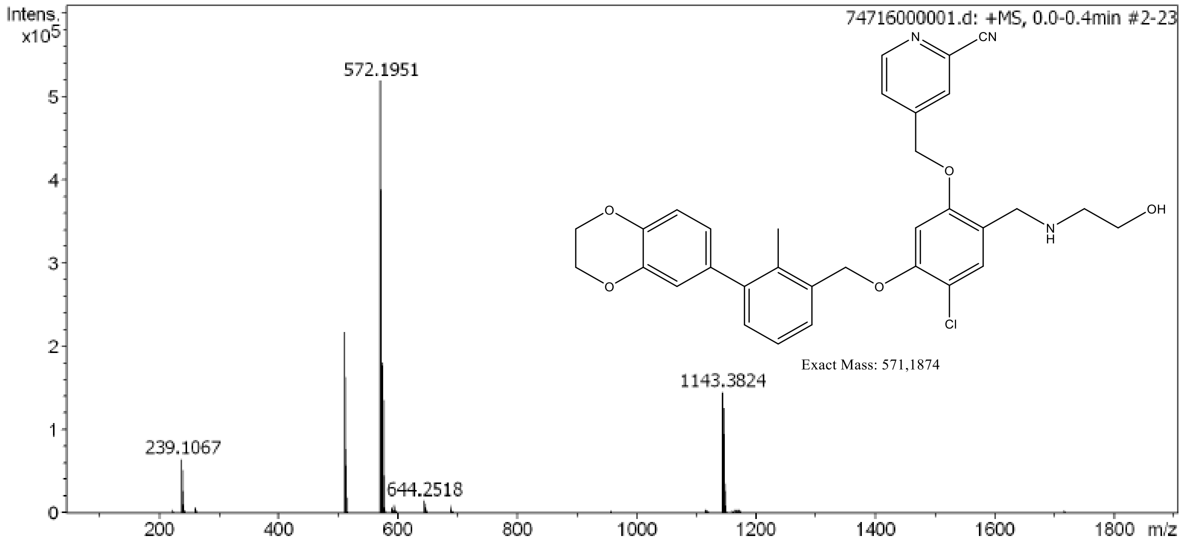

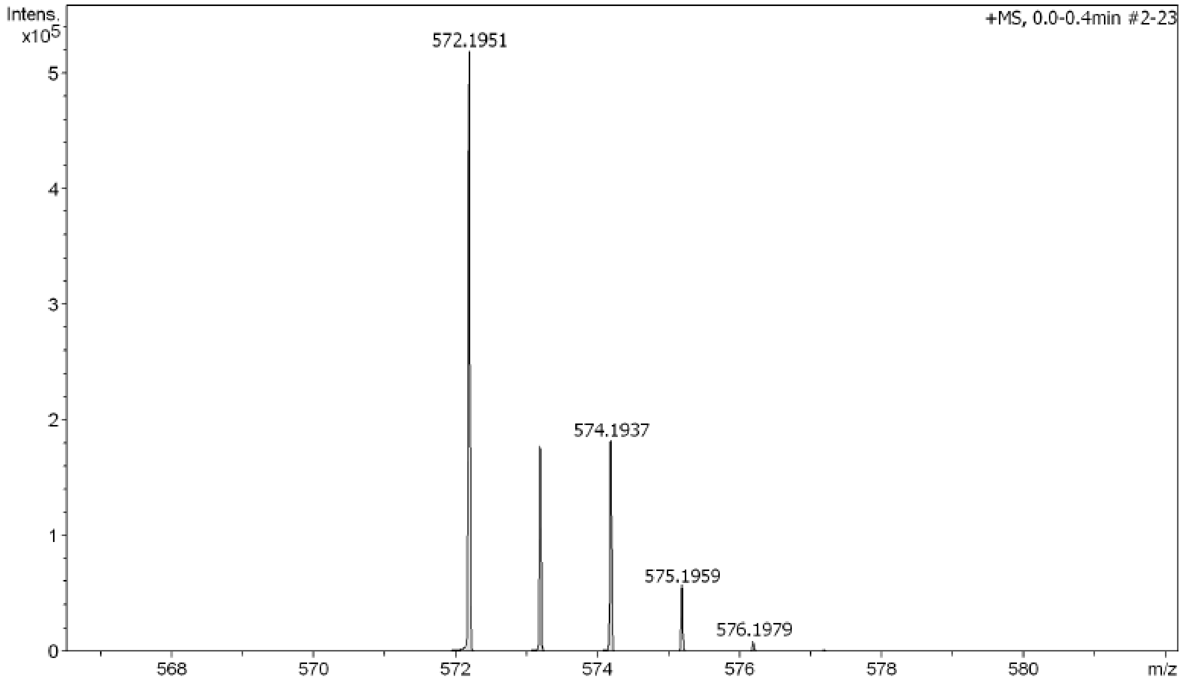

Figure S150: (*S*)-1-(5-chloro-2-((2-cyanopyridin-4-yl)methoxy)-4-((3-(2,3-dihydrobenzo[*b*][1,4]dioxin-6-yl)-2-methylbenzyl)oxy)benzyl)piperidine-2-carboxylic acid (**4f**):

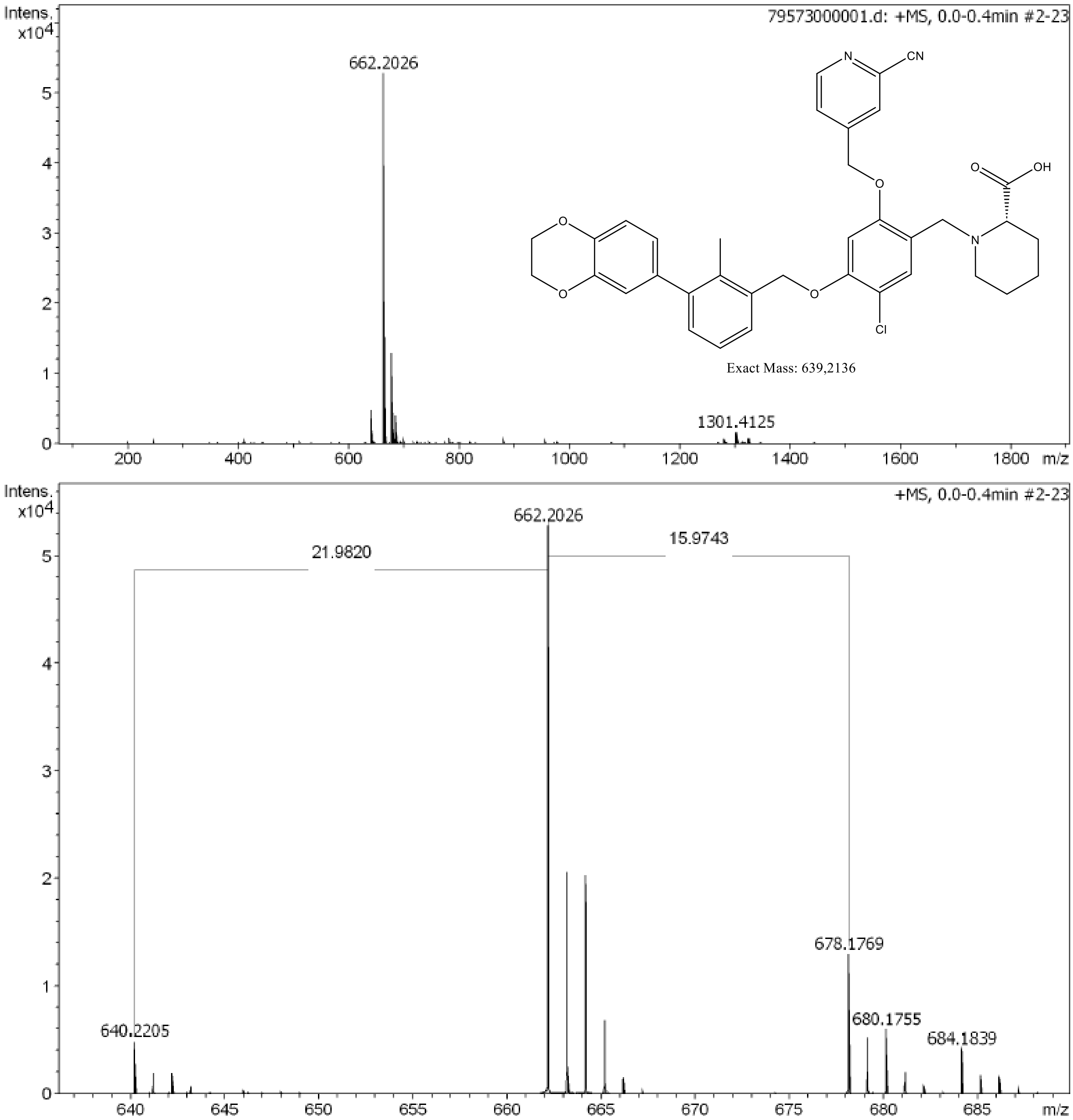

Figure S151: 1-(5-Chloro-2-((2-cyanopyridin-4-yl)methoxy)-4-((3-(2,3-dihydrobenzo[*b*][1,4]dioxin-6-yl)-2-methylbenzyl)oxy)phenyl)-5,8,11,14-tetraoxa-2-azaheptadecan-17-oic acid (**4g**):

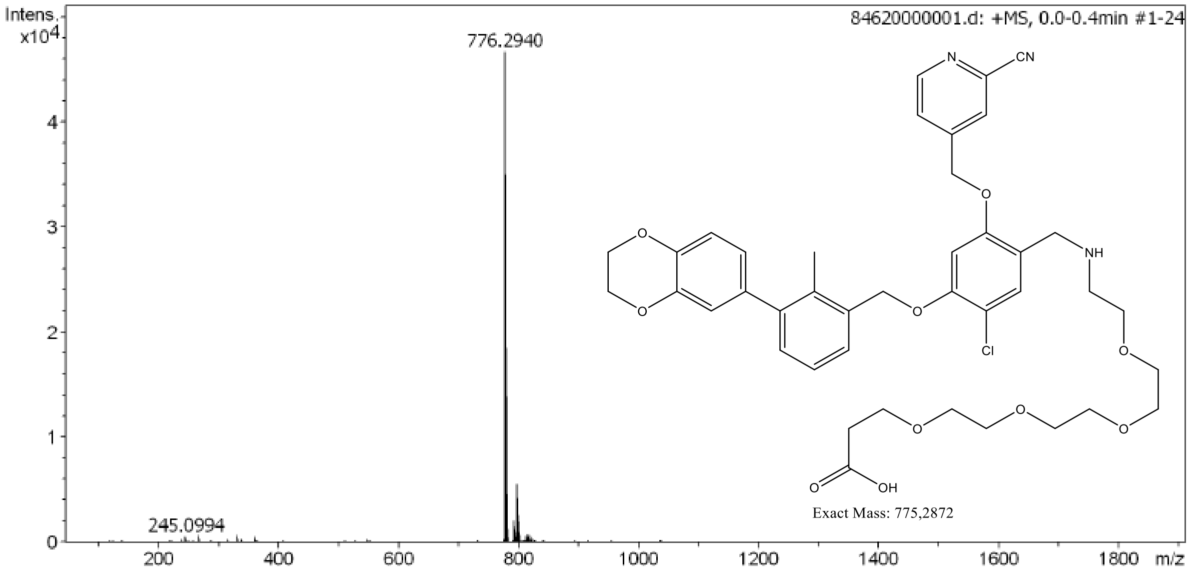

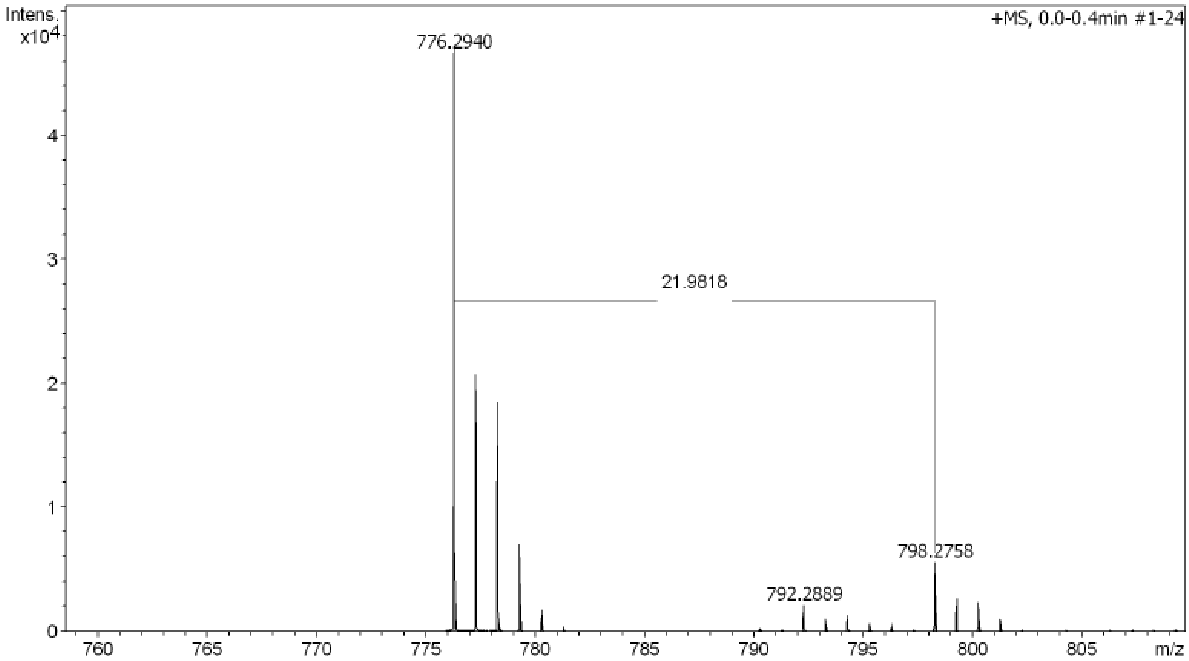

Figure S152: (*S*)-2-((5-chloro-2-((2-cyanopyridin-4-yl)methoxy)-4-((3-(2,3-dihydrobenzo[*b*][1,4]dioxin-6-yl)-2-methylbenzyl)oxy)benzyl)amino)hex-5-ynoic acid (**4h**):

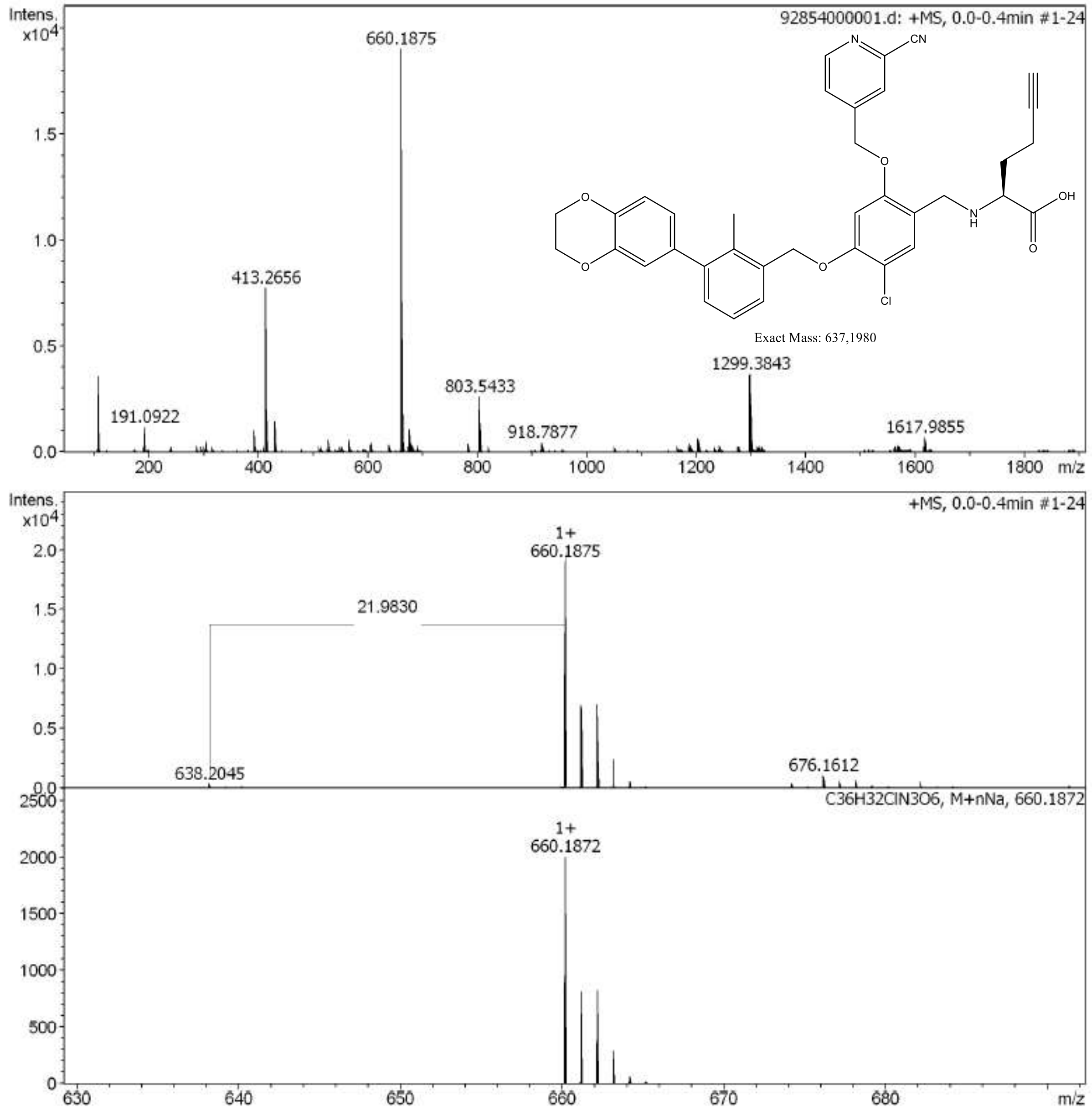

Figure S153: N^2-(5-chloro-2-((2-cyanopyridin-4-yl)methoxy)-4-((3-(2,3-dihydrobenzo[*b*][1,4]dioxin-6-yl)-2-methylbenzyl)oxy)benzyl)-N^6-((prop-2-yn-1-yloxy)carbonyl)-L-lysine (**4i**):

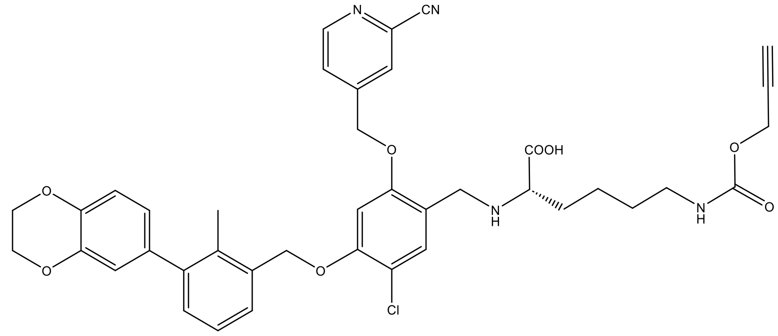

Exact Mass: 738,2456

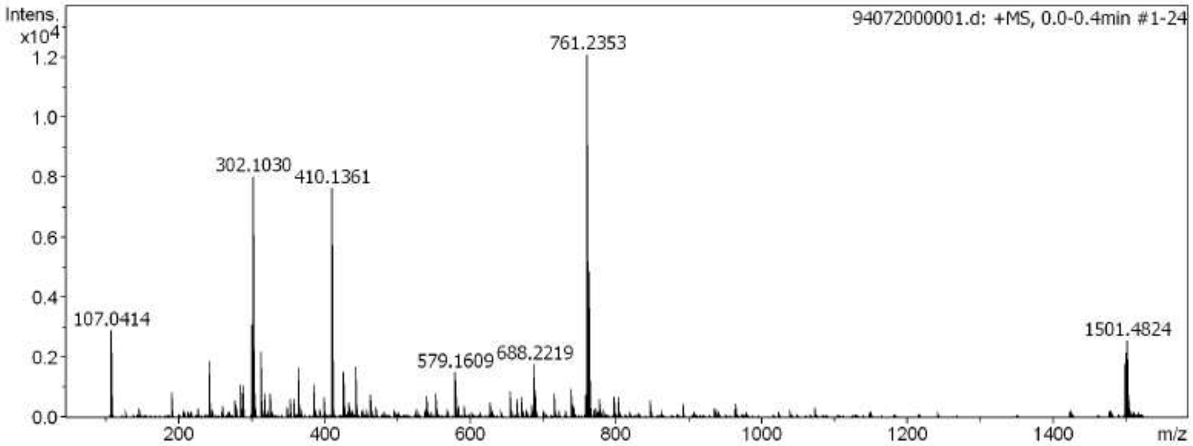

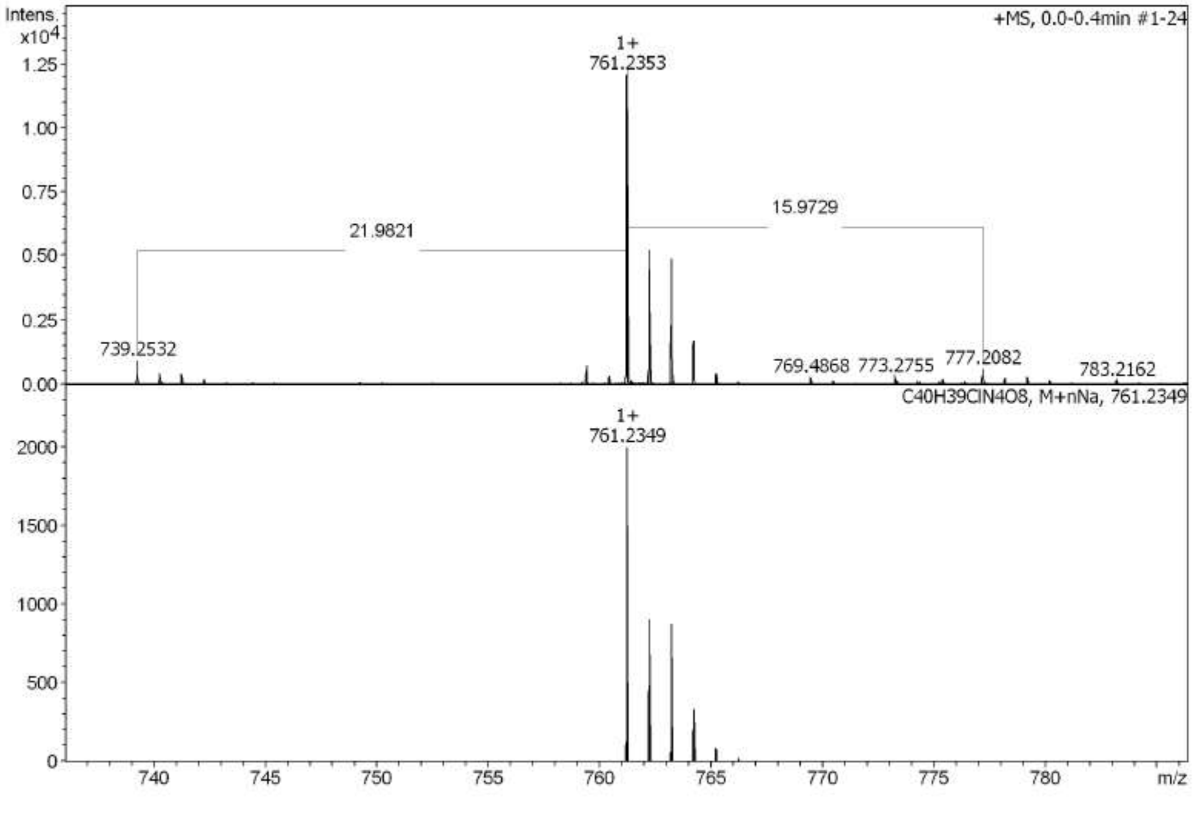

Figure S154: *N*-(2-((5-chloro-2-((3-cyanobenzyl)oxy)-4-((3-(2,3-dihydrobenzo[*b*][1,4]dioxin-6-yl)-2-methylbenzyl)oxy)benzyl)amino)ethyl)acetamide (**4j**):

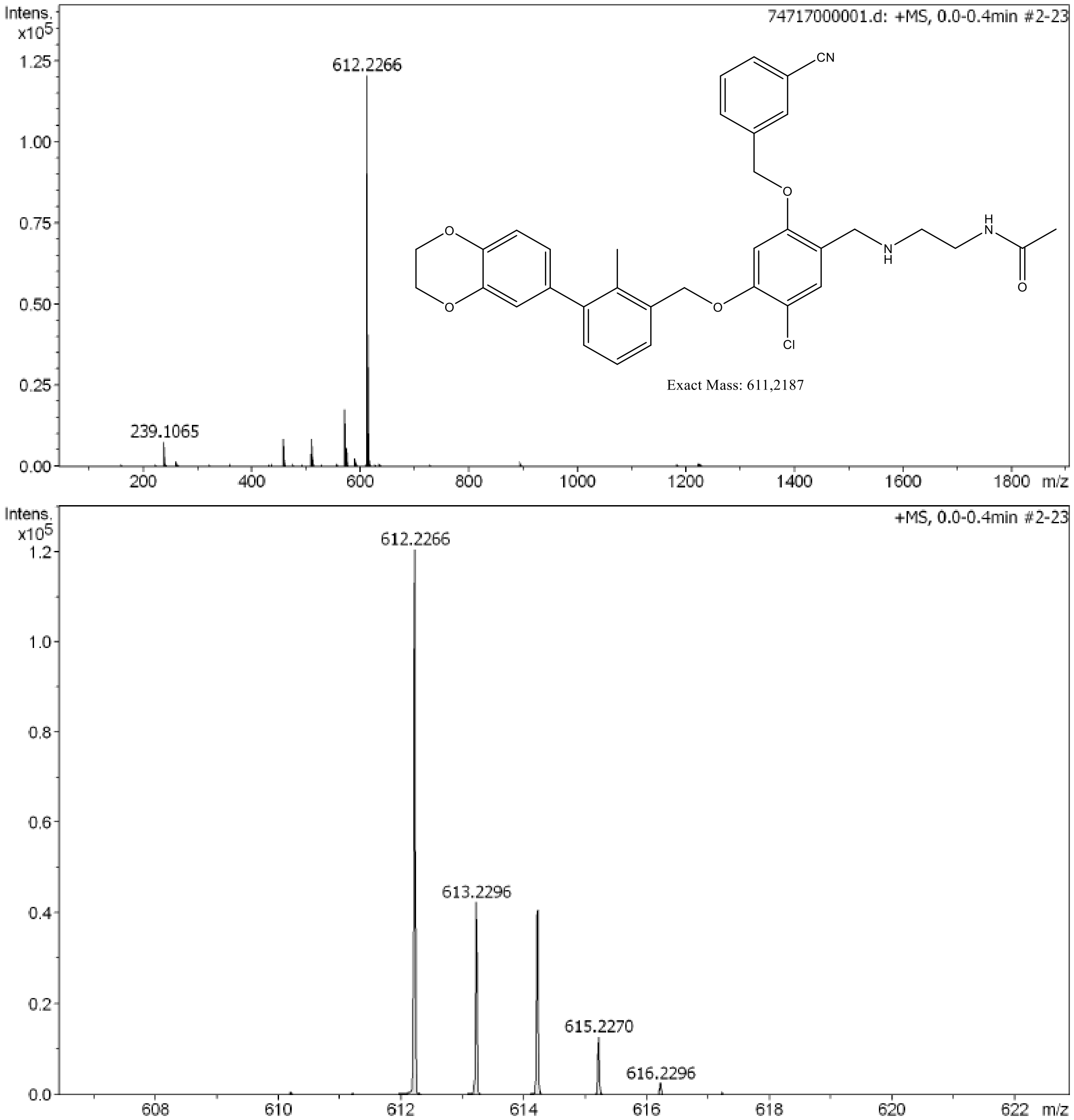

Figure S155: *N*-(2-((5-chloro-4-((3-(2,3-dihydrobenzo[*b*][1,4]dioxin-6-yl)-2-methylbenzyl)oxy)-2-(oxazol-4-ylmethoxy)benzyl)amino)ethyl)acetamide (**4k**):

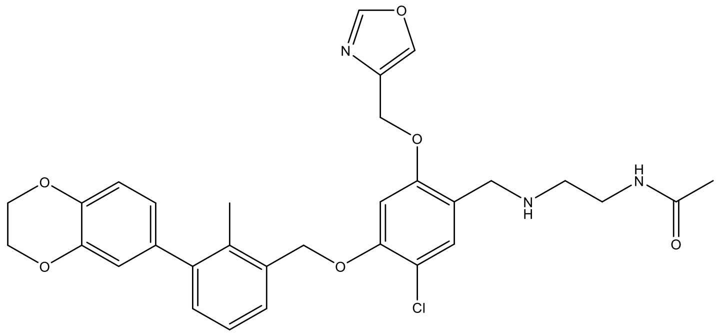

Exact Mass: 577,1980

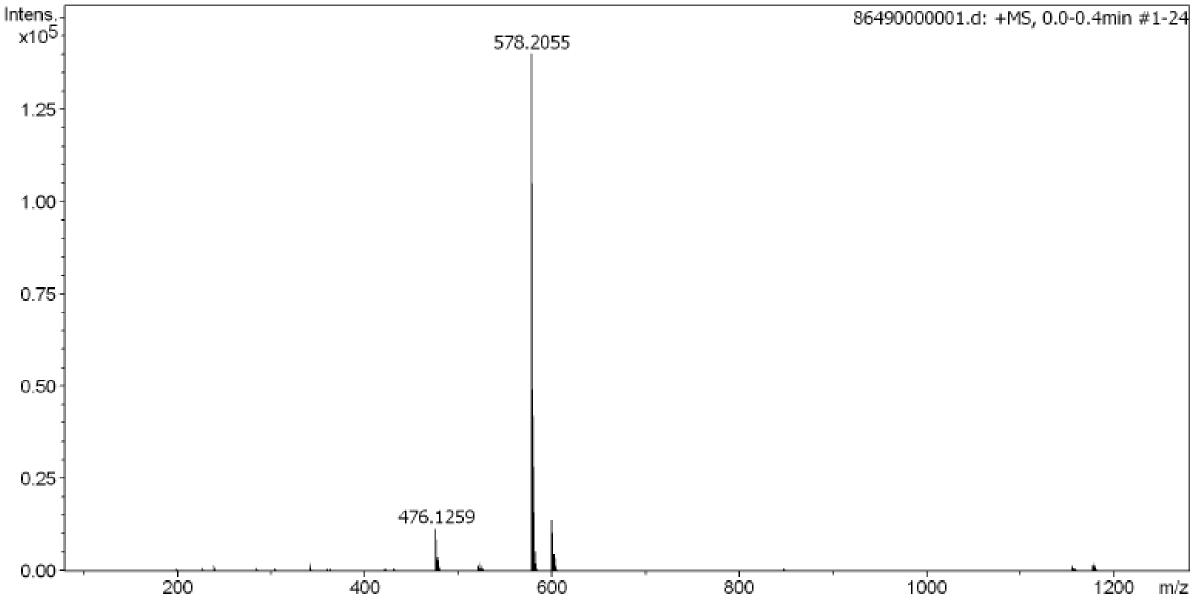

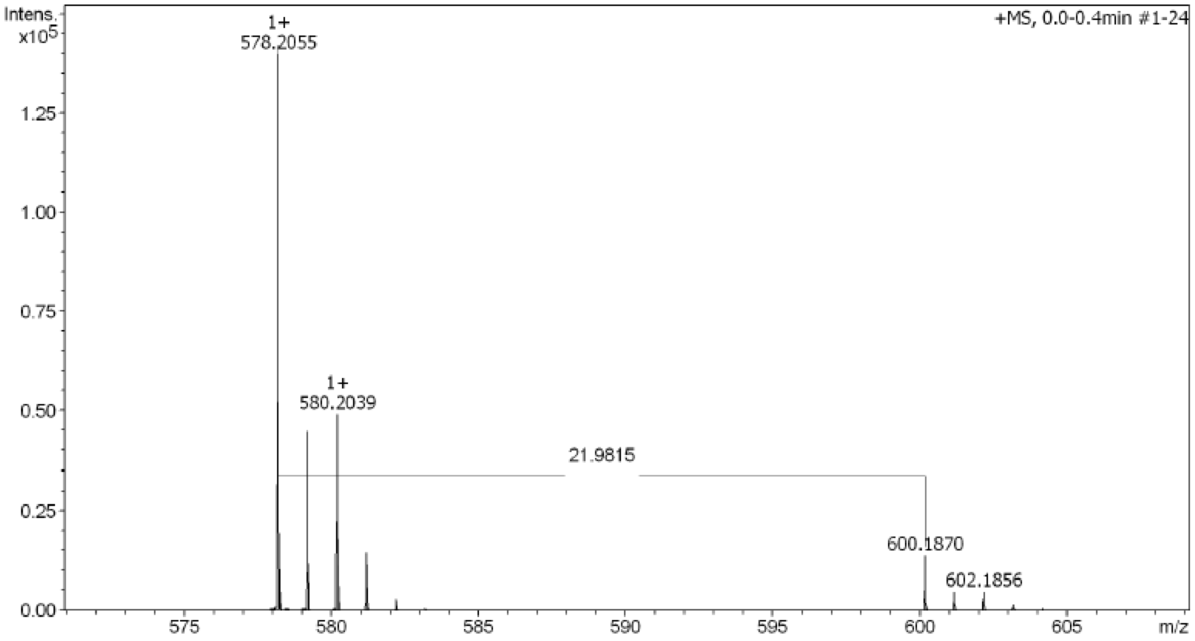

Figure S156: (*S*)-1-(5-chloro-4-((3-(2,3-dihydrobenzo[*b*][1,4]dioxin-6-yl)-2-methylbenzyl)oxy)-2-(oxazol-4-ylmethoxy)benzyl)piperidine-2-carboxylic acid (**4l**):

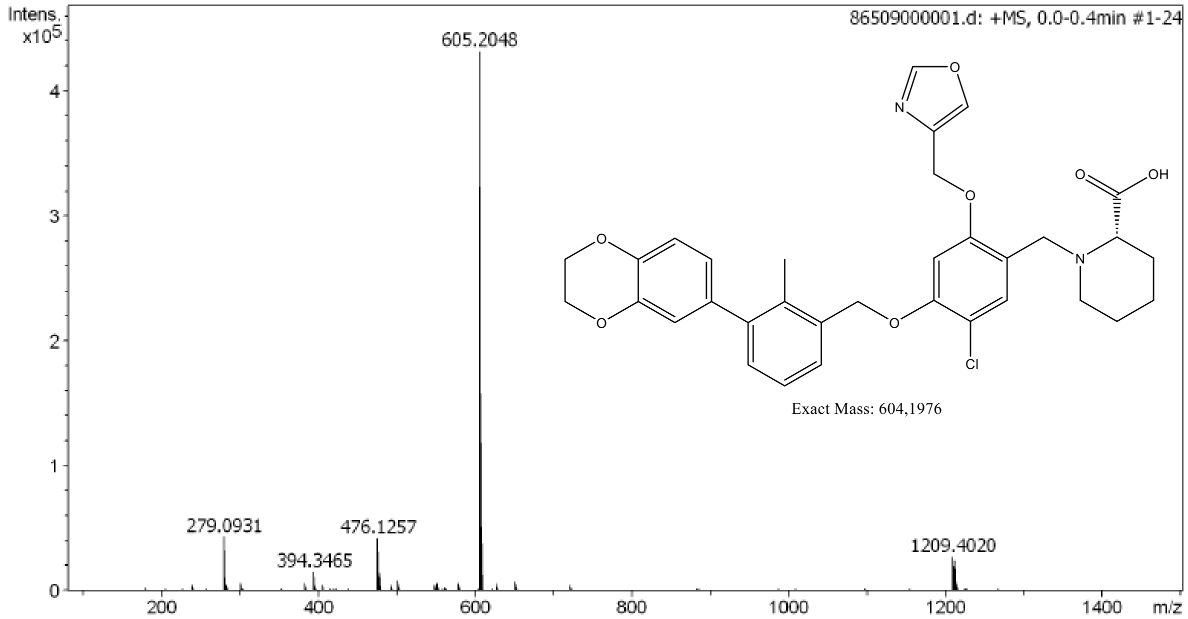

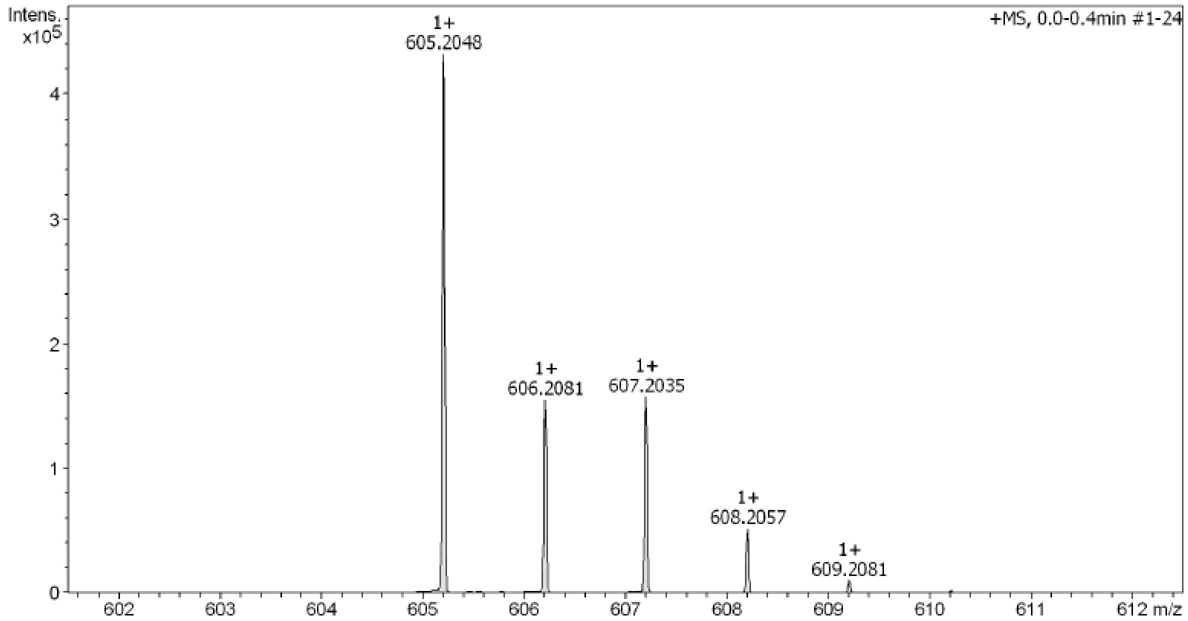

Figure S157: (5-Chloro-2-((2-cyanopyridin-4-yl)methoxy)-4-((2-methyl-3-(1*H*-pyrrol-1-yl)benzyl)oxy)benzyl)-*D*-serine (**4m**):

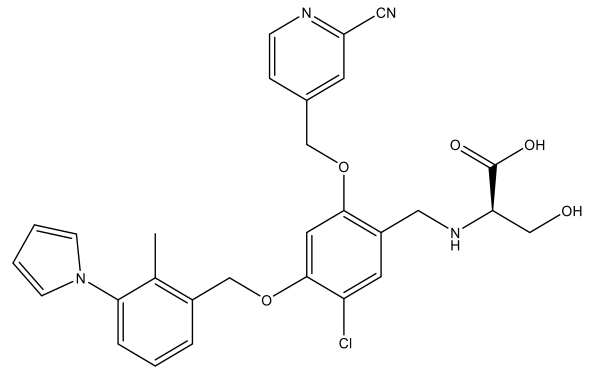

Exact Mass: 546,1670

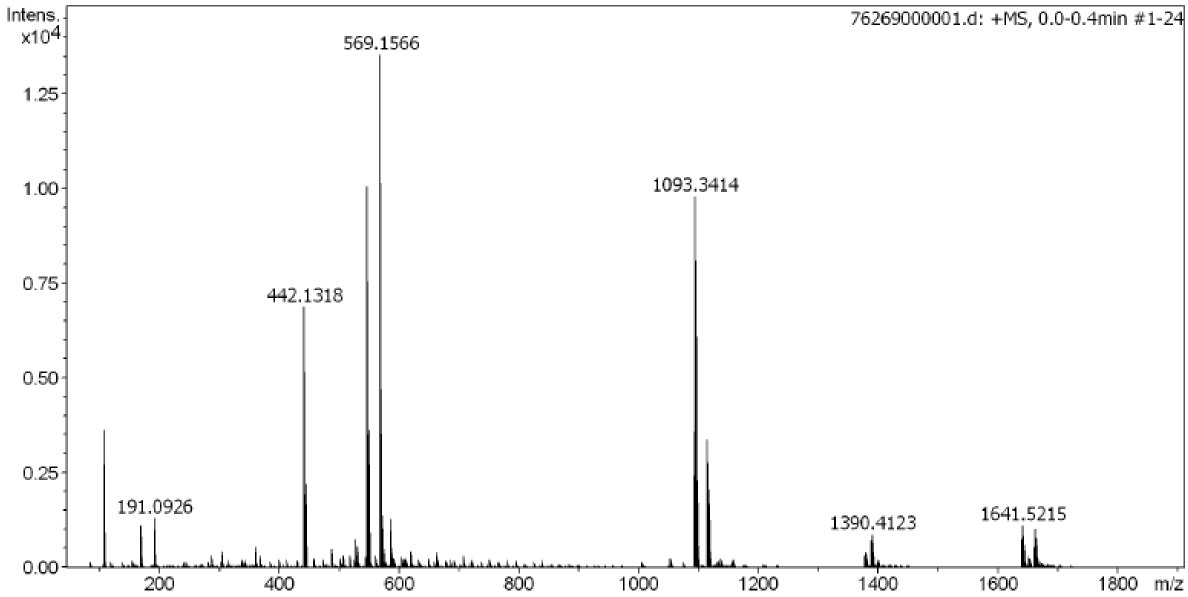

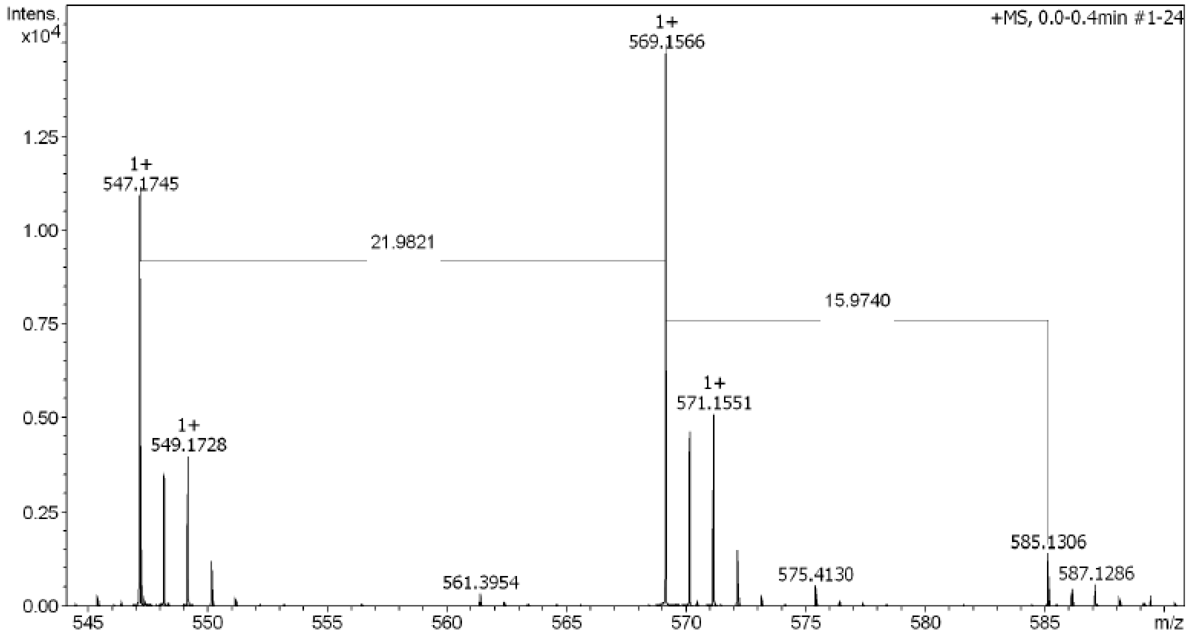

Figure S158: 4-((4-Chloro-2-(((2-hydroxyethyl)amino)methyl)-5-((2-methyl-3-(1*H*-pyrrol-1-yl)benzyl)oxy)phenoxy)methyl)picolinonitrile (**4n**):

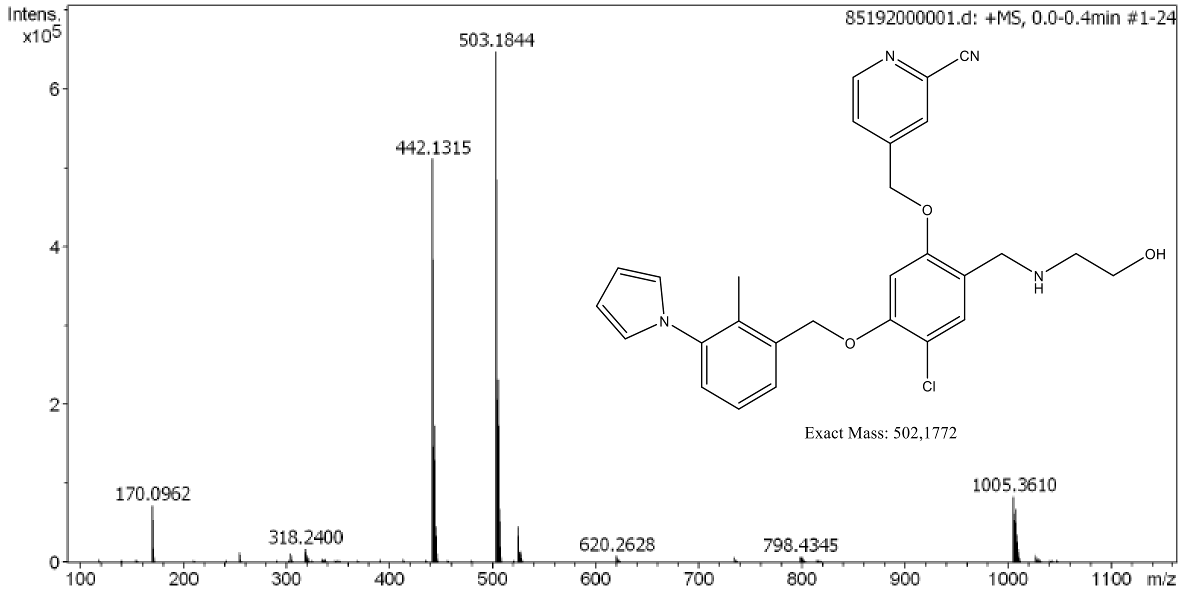

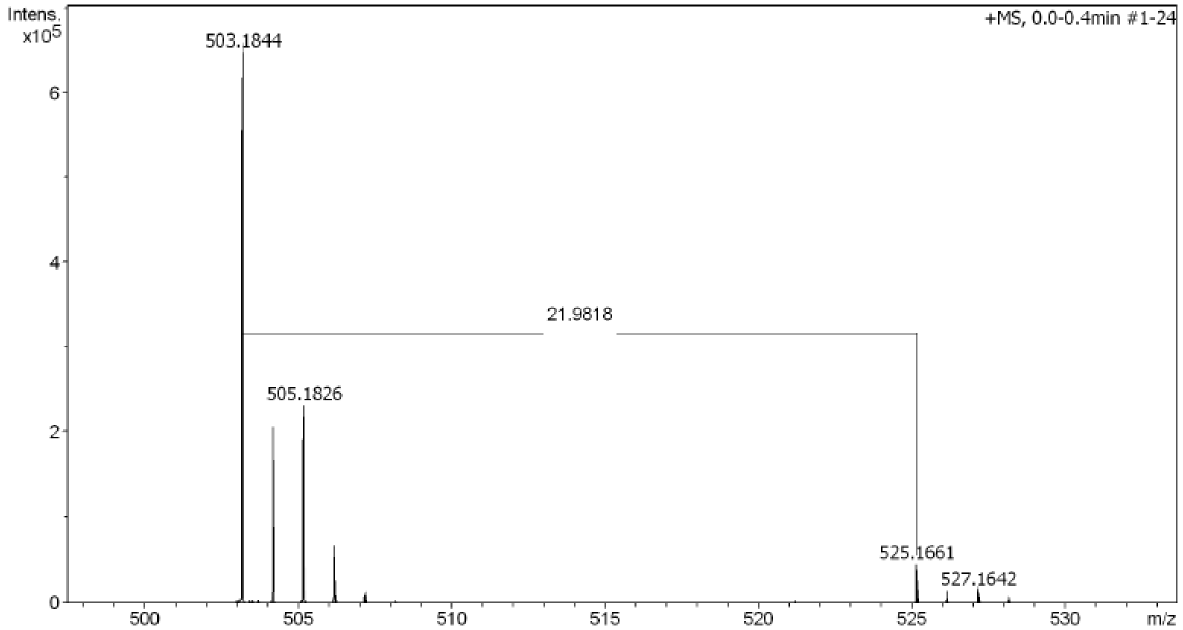

Figure S159: (*S*)-1-(5-chloro-2-((2-cyanopyridin-4-yl)methoxy)-4-((2-methyl-3-(1*H*-pyrrol-1-yl)benzyl)oxy)benzyl)piperidine-2-carboxylic acid (**4o**):

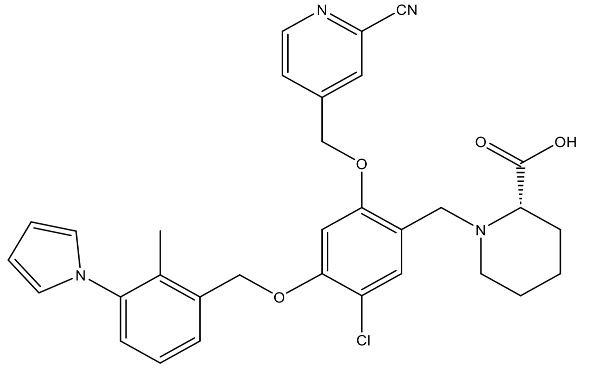

Exact Mass: 570,2034

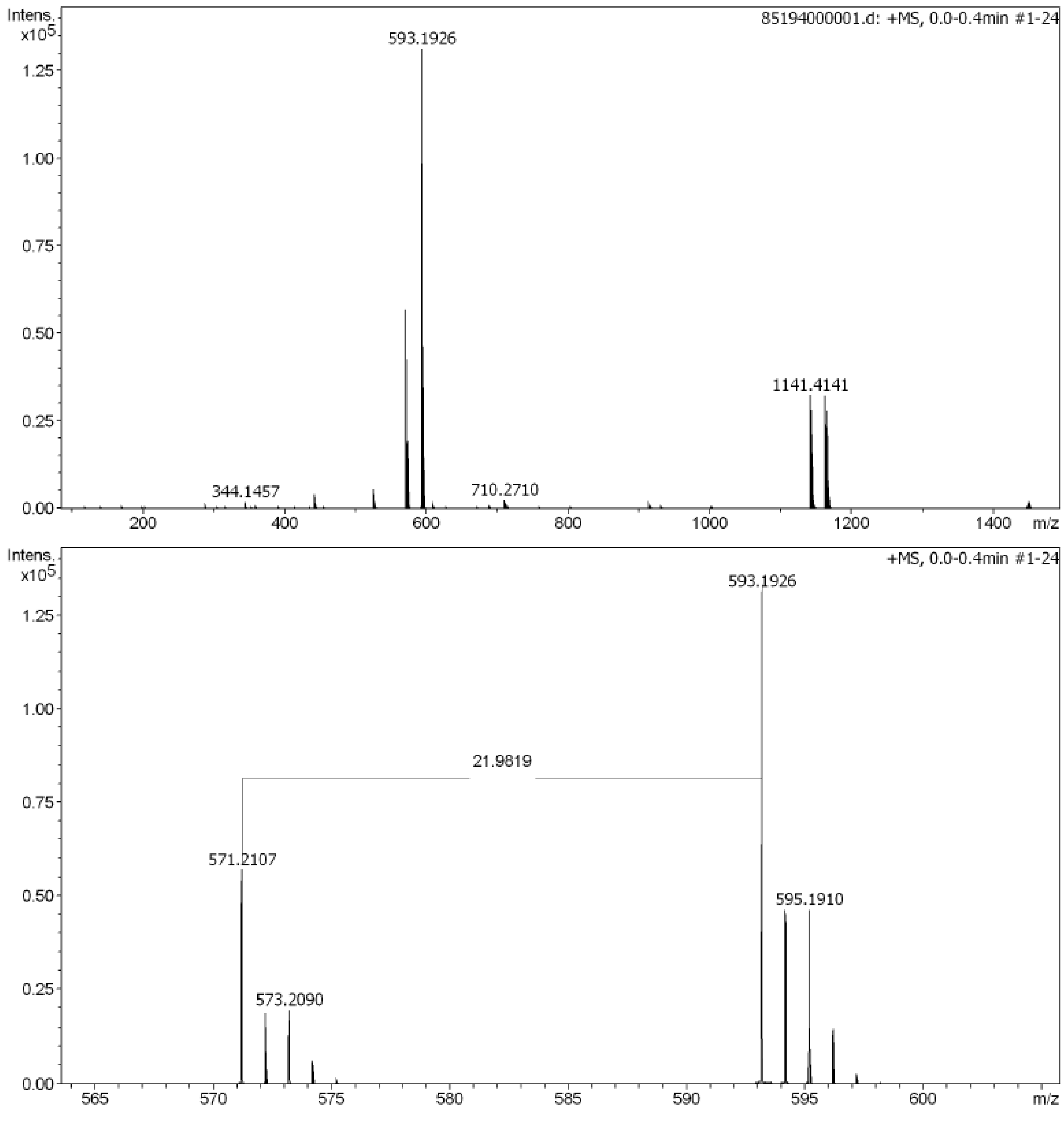

Figure S160: *N*-(5-chloro-2-((2-cyanopyridin-4-yl)methoxy)-4-((3-(2,3-dihydrobenzo[*b*][1,4]dioxin-6-yl)-2-methylbenzyl)oxy)benzyl)-*N*-methyl-*D*-serine (**5a**):

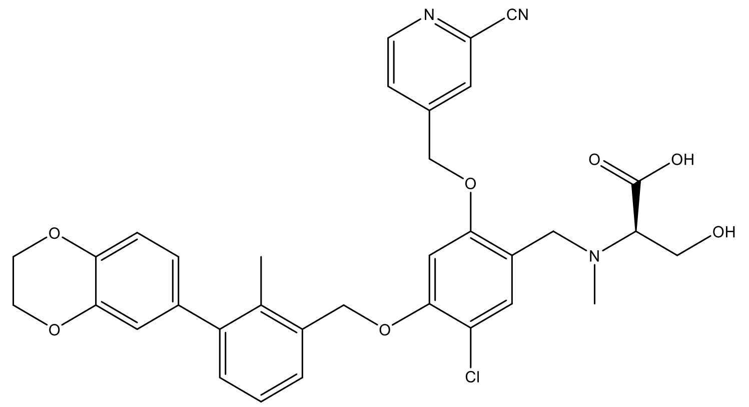

Exact Mass: 629,1929

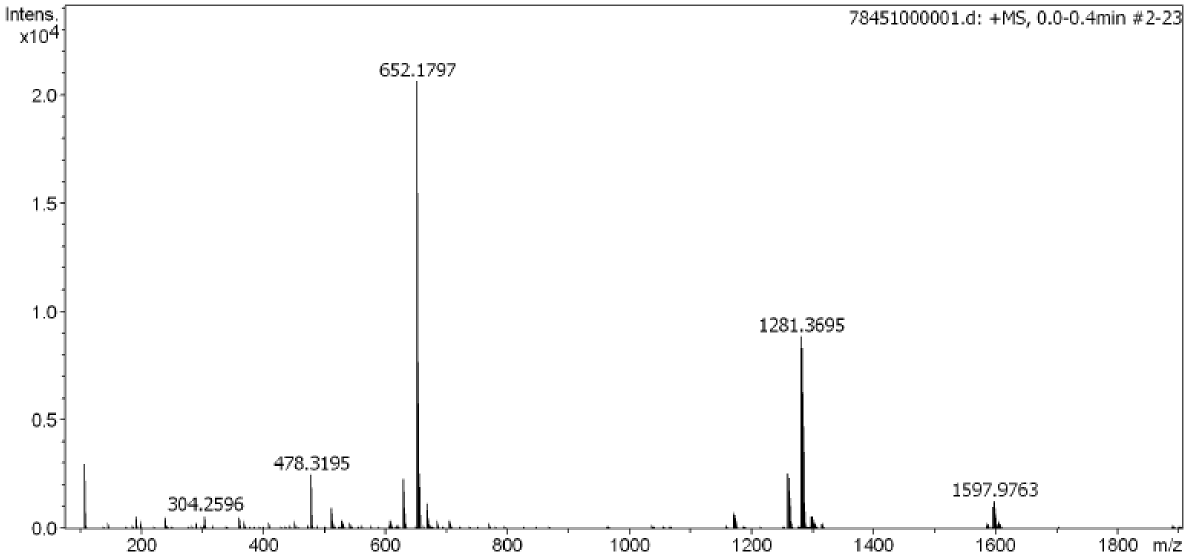

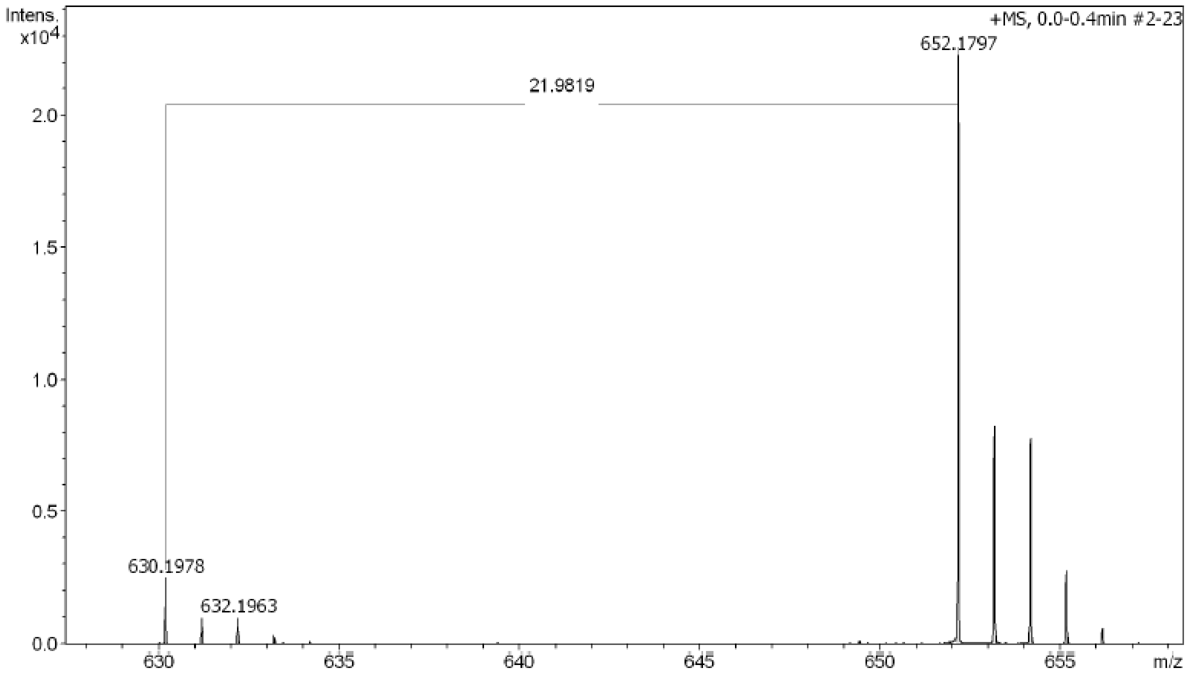

Figure S161: Methyl (5-chloro-2-((2-cyanopyridin-4-yl)methoxy)-4-((3-(2,3-dihydrobenzo[*b*][1,4]dioxin-6-yl)-2-methylbenzyl)oxy)benzyl)-*D*-serinate (**5b**):

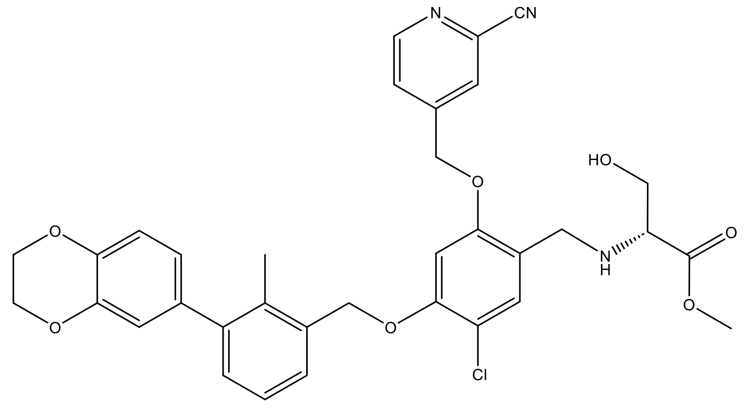

Exact Mass: 629,1929

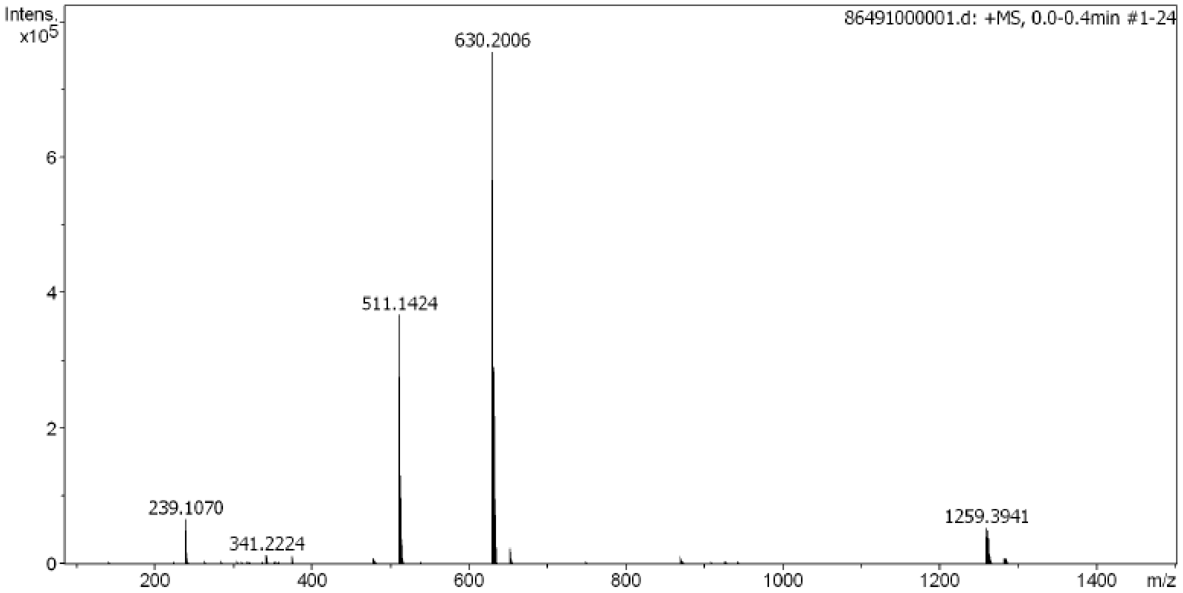

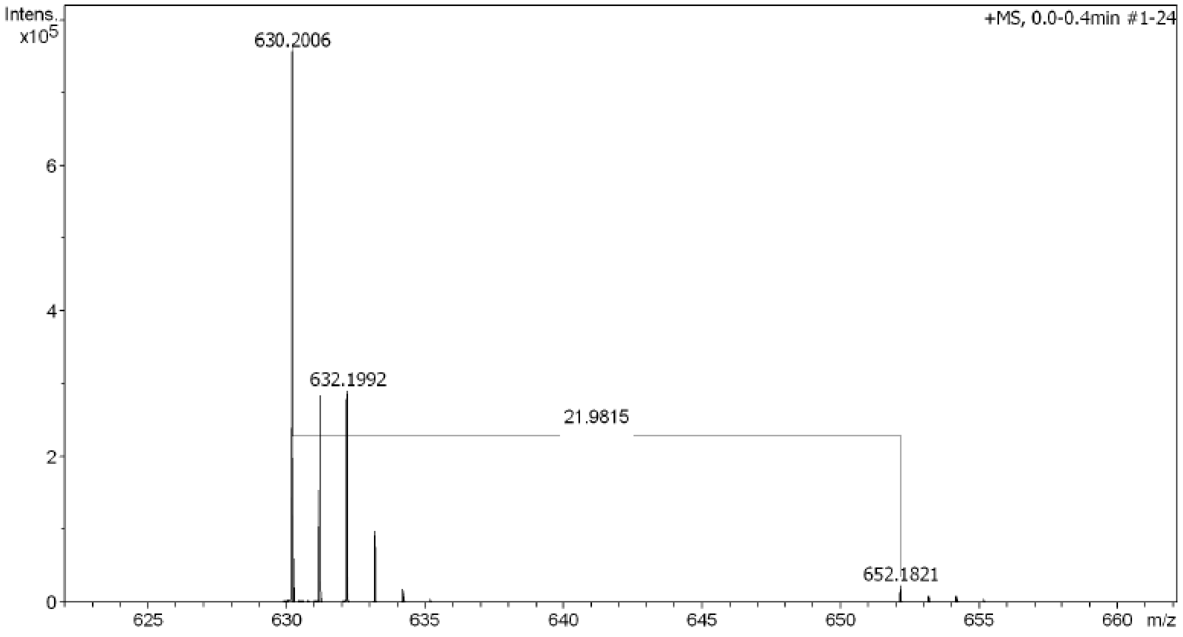

Figure S162: 4-((4-Chloro-5-((3-(2,3-dihydrobenzo[*b*][1,4]dioxin-6-yl)-2-methylbenzyl)oxy)-2-(((2-hydroxyethyl)(methyl)amino)methyl)phenoxy)methyl)picolinonitrile (**5c**):

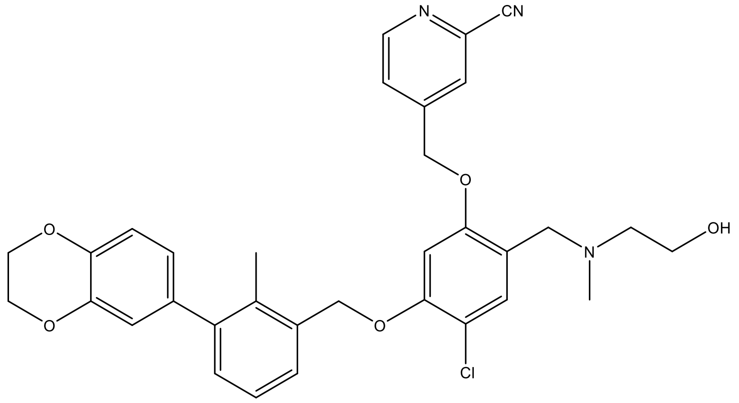

Exact Mass: 585,2030

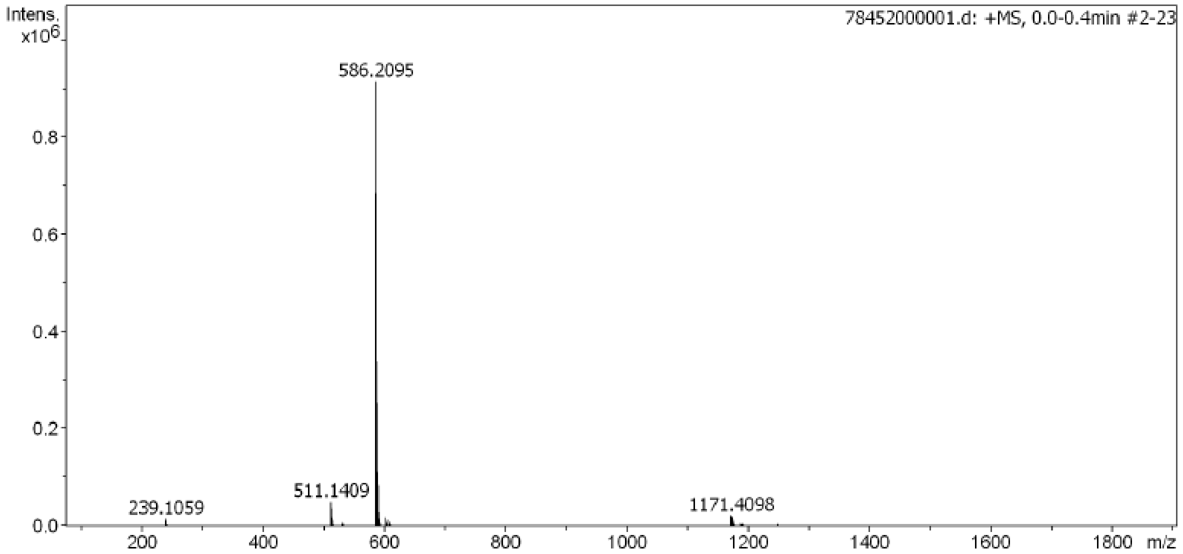

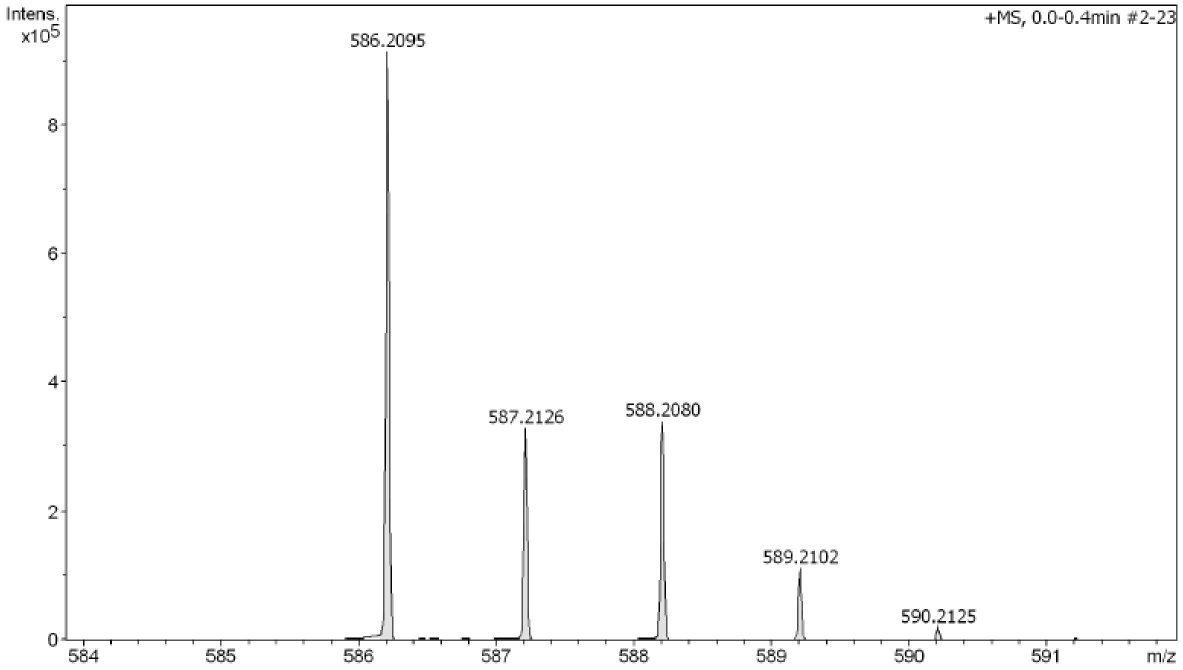

Figure S163: 4-((4-Chloro-5-((3-(2,3-dihydrobenzo[*b*][1,4]dioxin-6-yl)-2-methylbenzyl)oxy)-2-(((2-methoxyethyl)amino)methyl)phenoxy)methyl)picolinonitrile (**5d**):

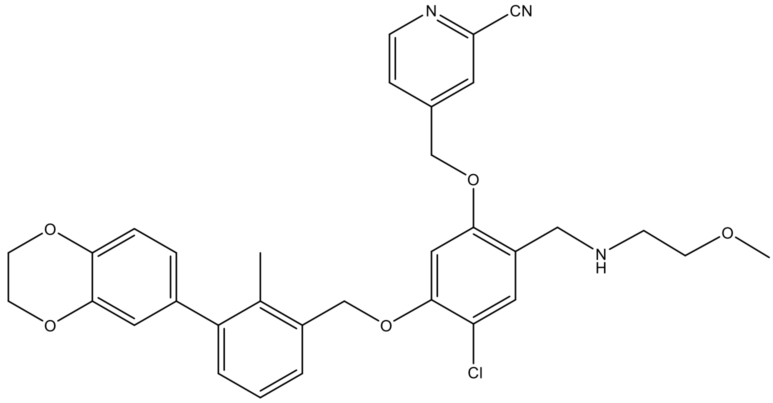

Exact Mass: 585,2030

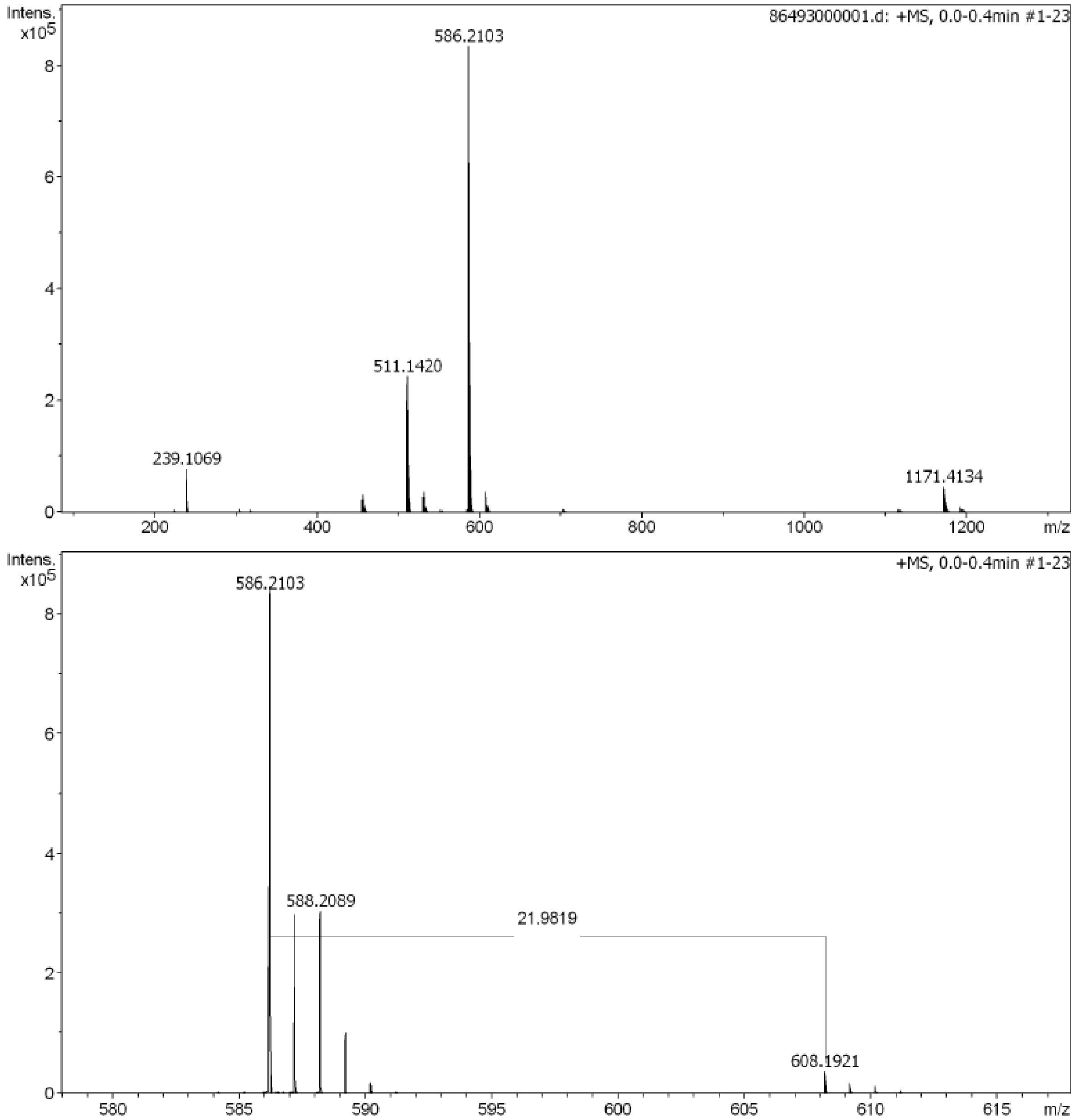

Figure S164: 3-((2-((3-Acetyl-2-oxoimidazolidin-1-yl)methyl)-4-chloro-5-((3-(2,3-dihydrobenzo[*b*][1,4]dioxin-6-yl)-2-methylbenzyl)oxy)phenoxy)methyl)benzonitrile (**5e**):

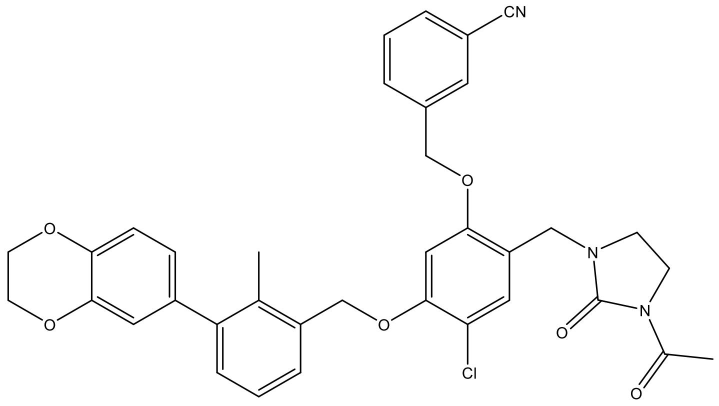

Exact Mass: 637,1980

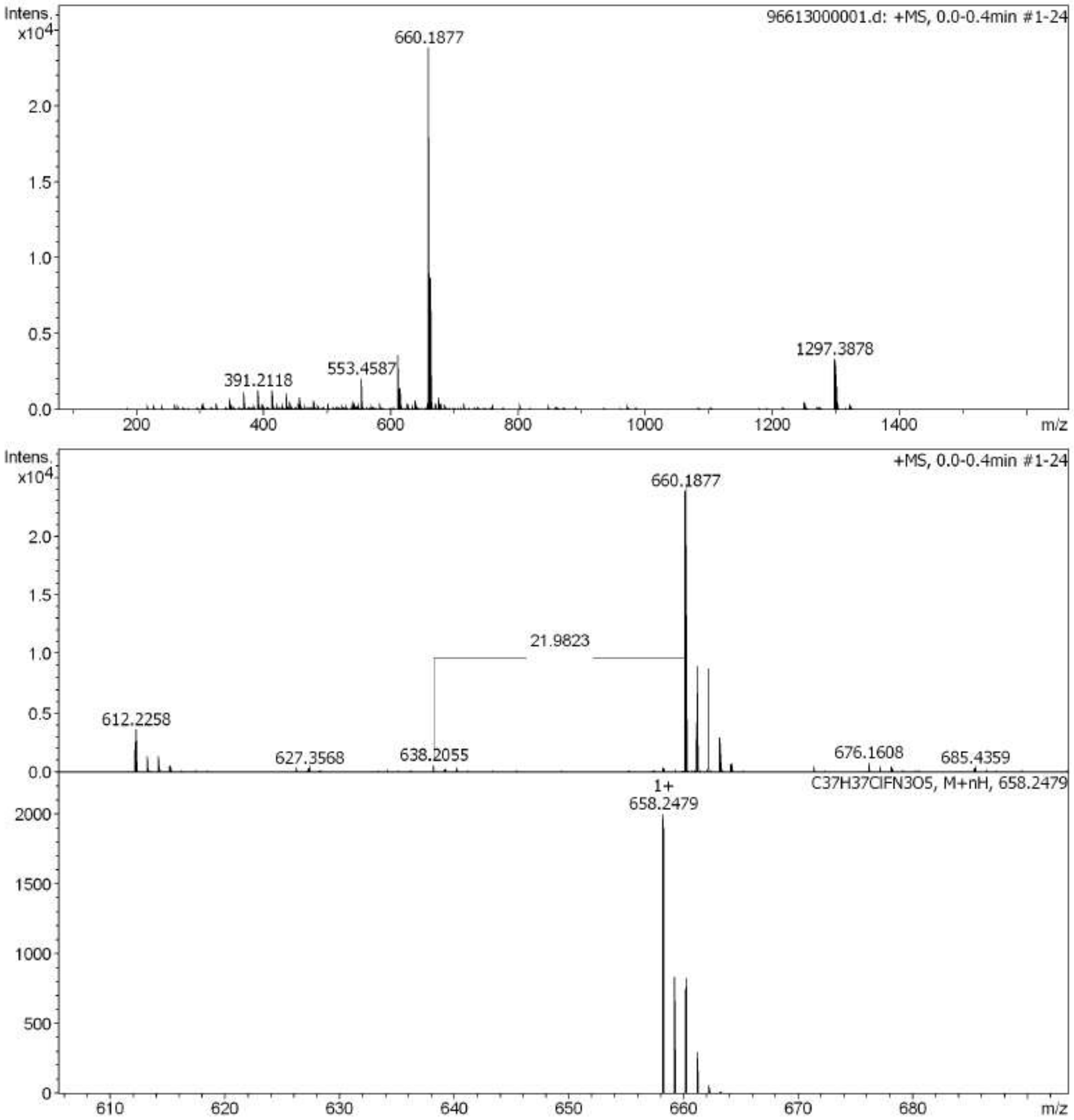

Figure S165: 4-((4-Chloro-2-(((2-hydroxyethyl)(methyl)amino)methyl)-5-((2-methyl-3-(1*H*-pyrrol-1-yl)benzyl)oxy)phenoxy)methyl)picolinonitrile (**5f**):

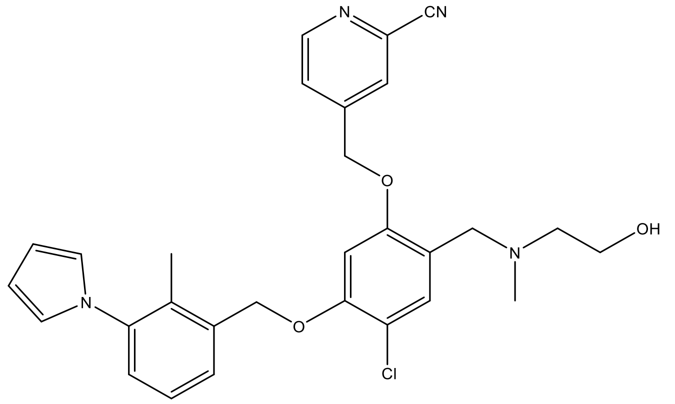

Exact Mass: 516,1928

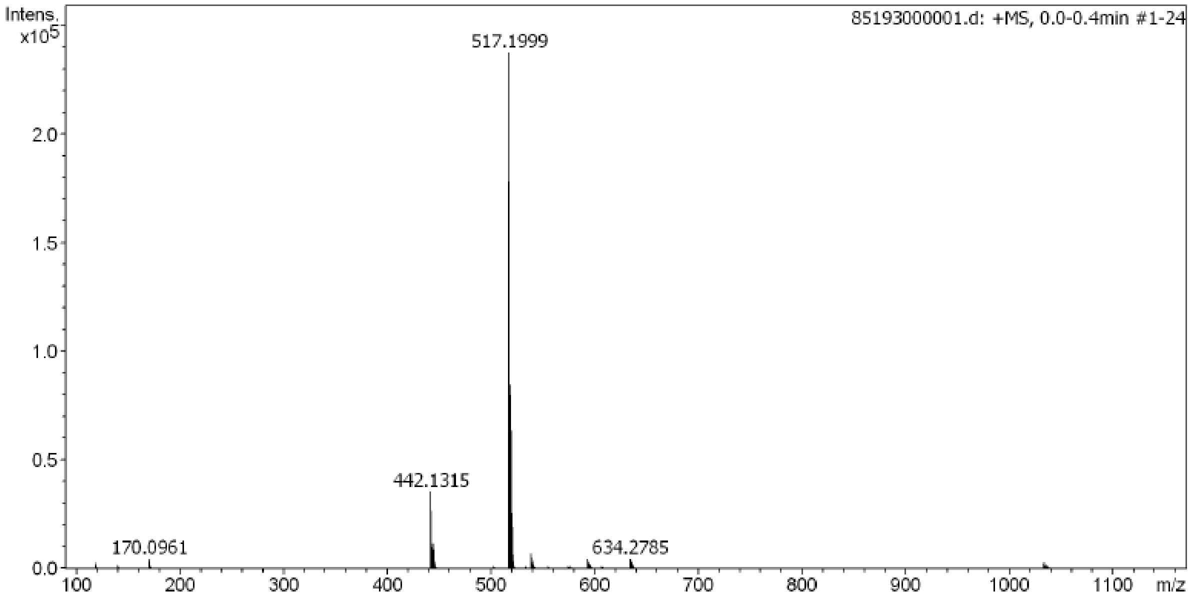

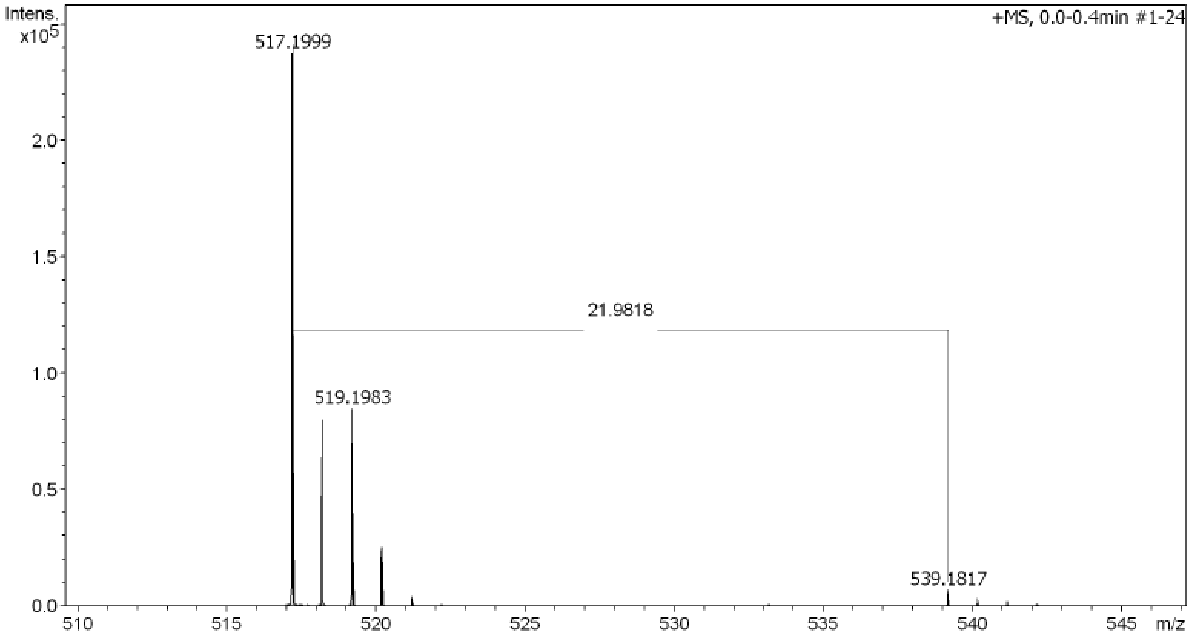

Figure S166: 4-((4-Chloro-5-((3-(2,3-dihydrobenzo[*b*][1,4]dioxin-6-yl)-2-methylbenzyl)oxy)-2-((2-(fluoromethyl)oxazolidin-3-yl)methyl)phenoxy)methyl)picolinonitrile (**5g**):

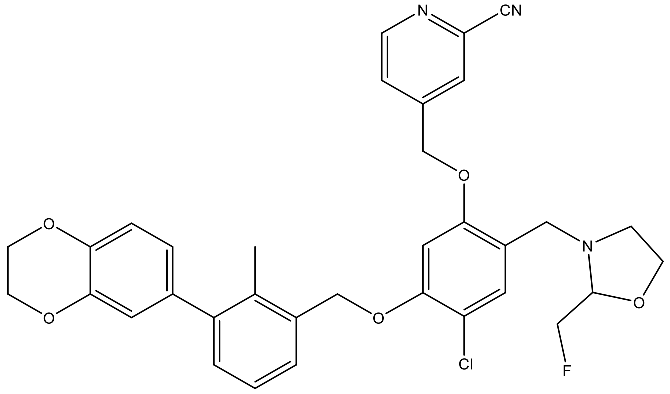

Exact Mass: 615,1936

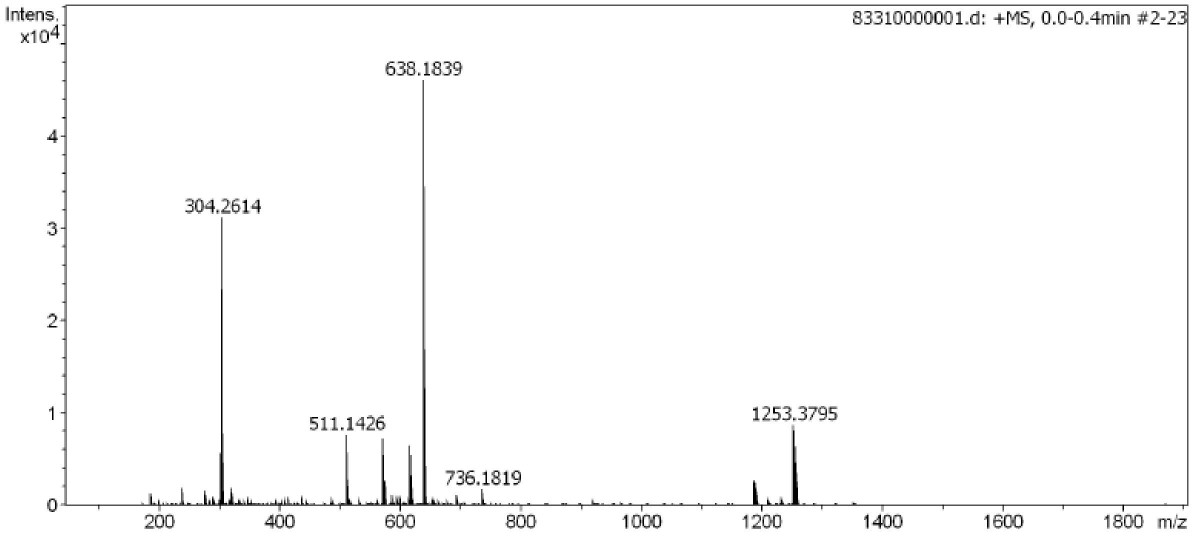

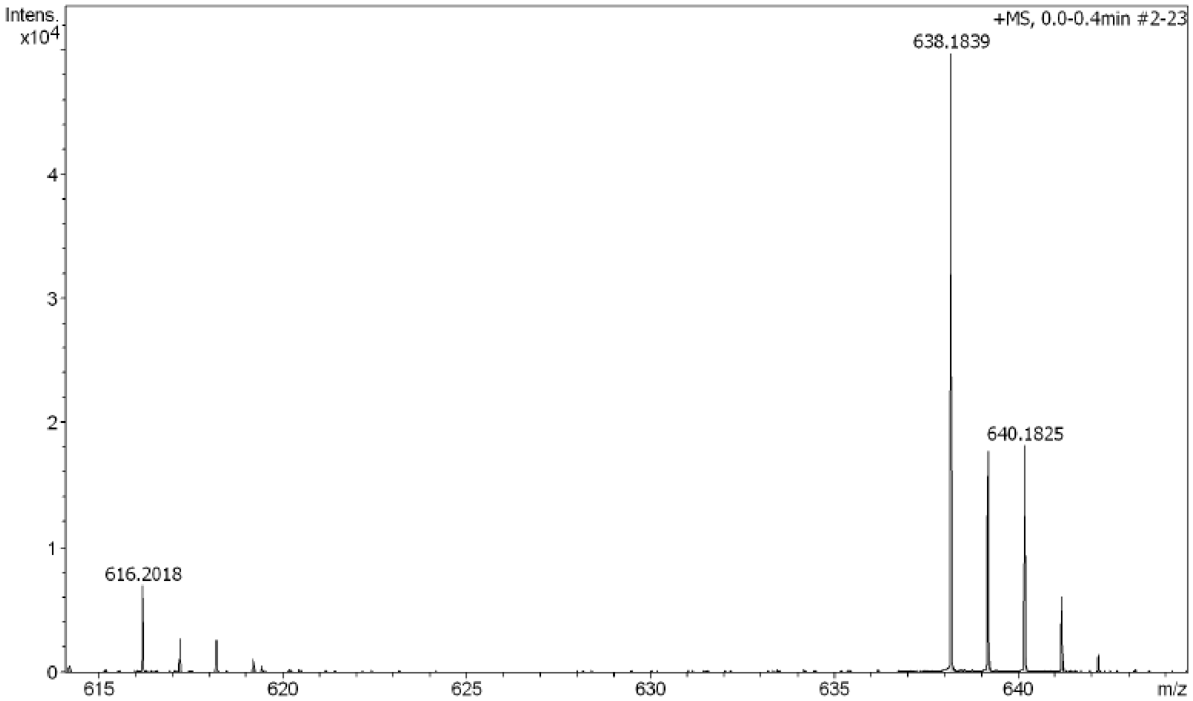

Figure S167: 2-Fluoroethyl (*S*)-1-(5-chloro-2-((2-cyanopyridin-4-yl)methoxy)-4-((3-(2,3-dihydrobenzo[*b*][1,4]dioxin-6-yl)-2-methylbenzyl)oxy)benzyl)piperidine-2-carboxylate (**5h**):

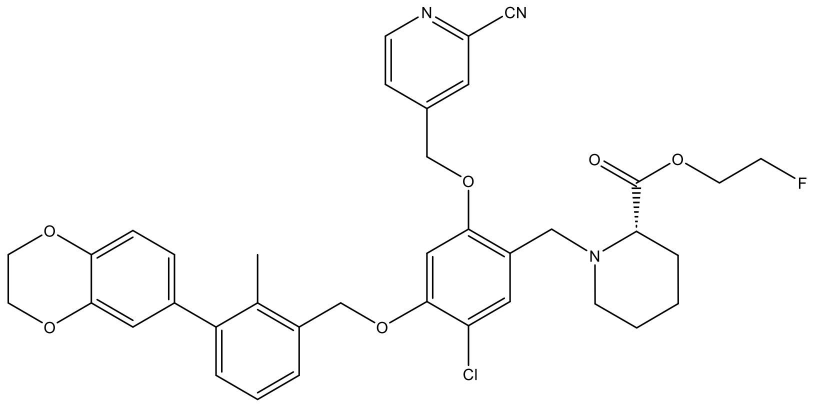

Exact Mass: 685,2355

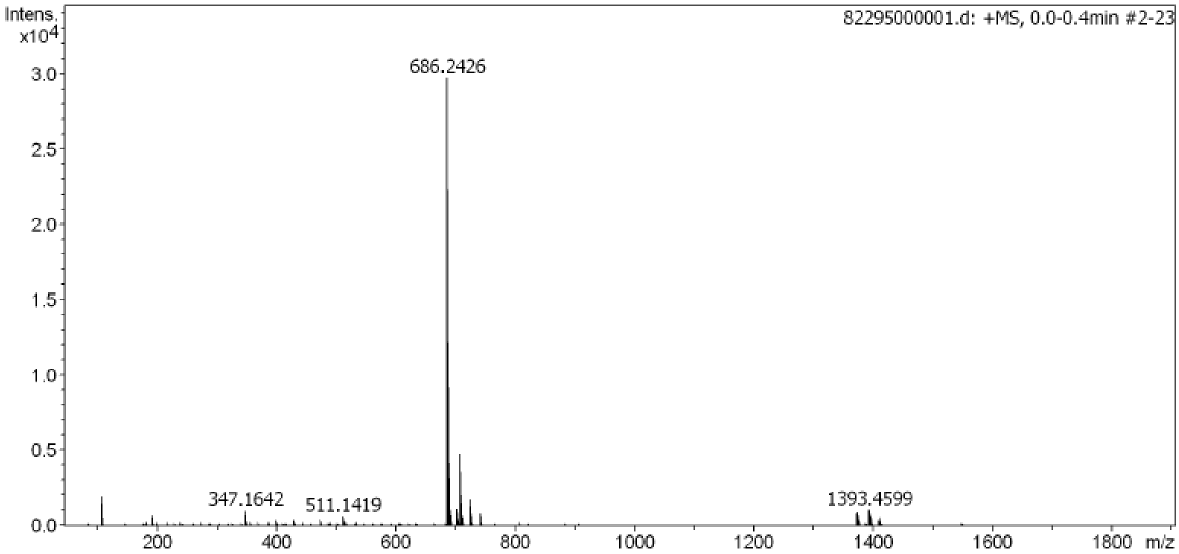

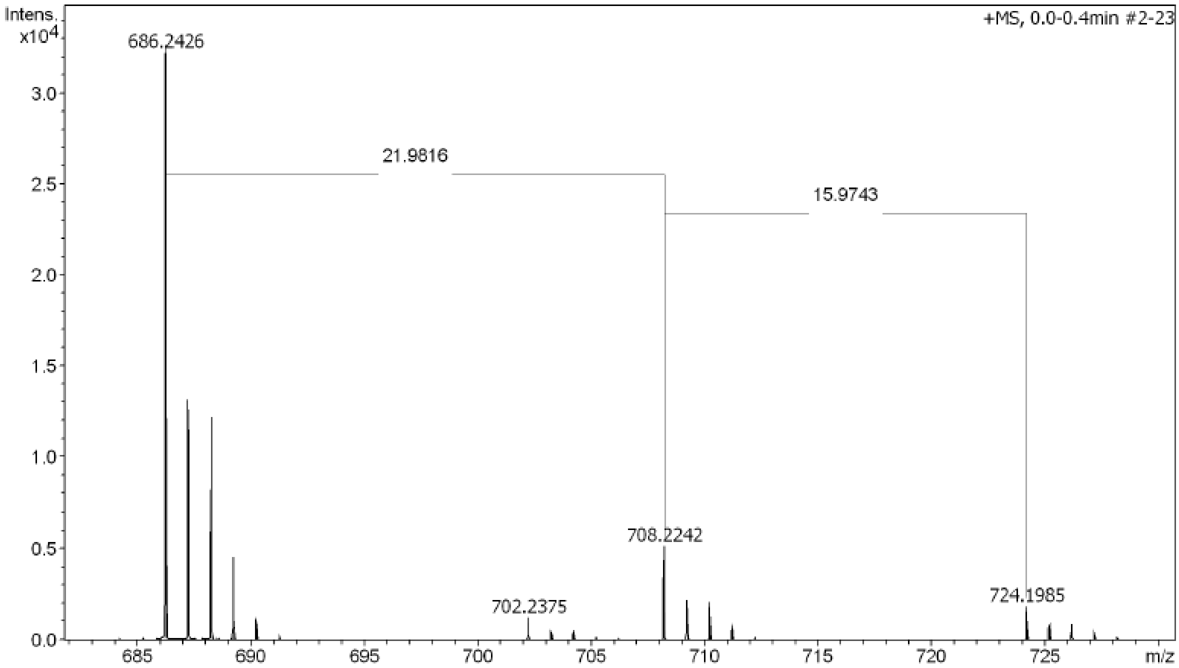

Figure S168: 4-((4-Chloro-5-((3-(2,3-dihydrobenzo[*b*][1,4]dioxin-6-yl)-2-methylbenzyl)oxy)-2-(((2-fluoroethyl)amino)methyl)phenoxy)methyl)picolinonitrile (**5i**):

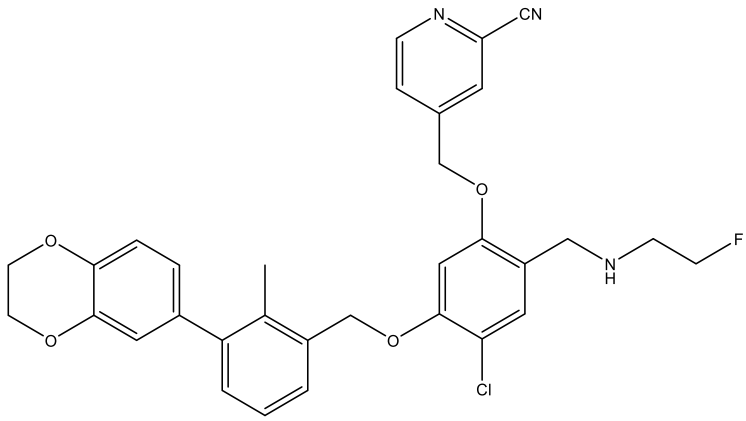

Exact Mass: 573,1831

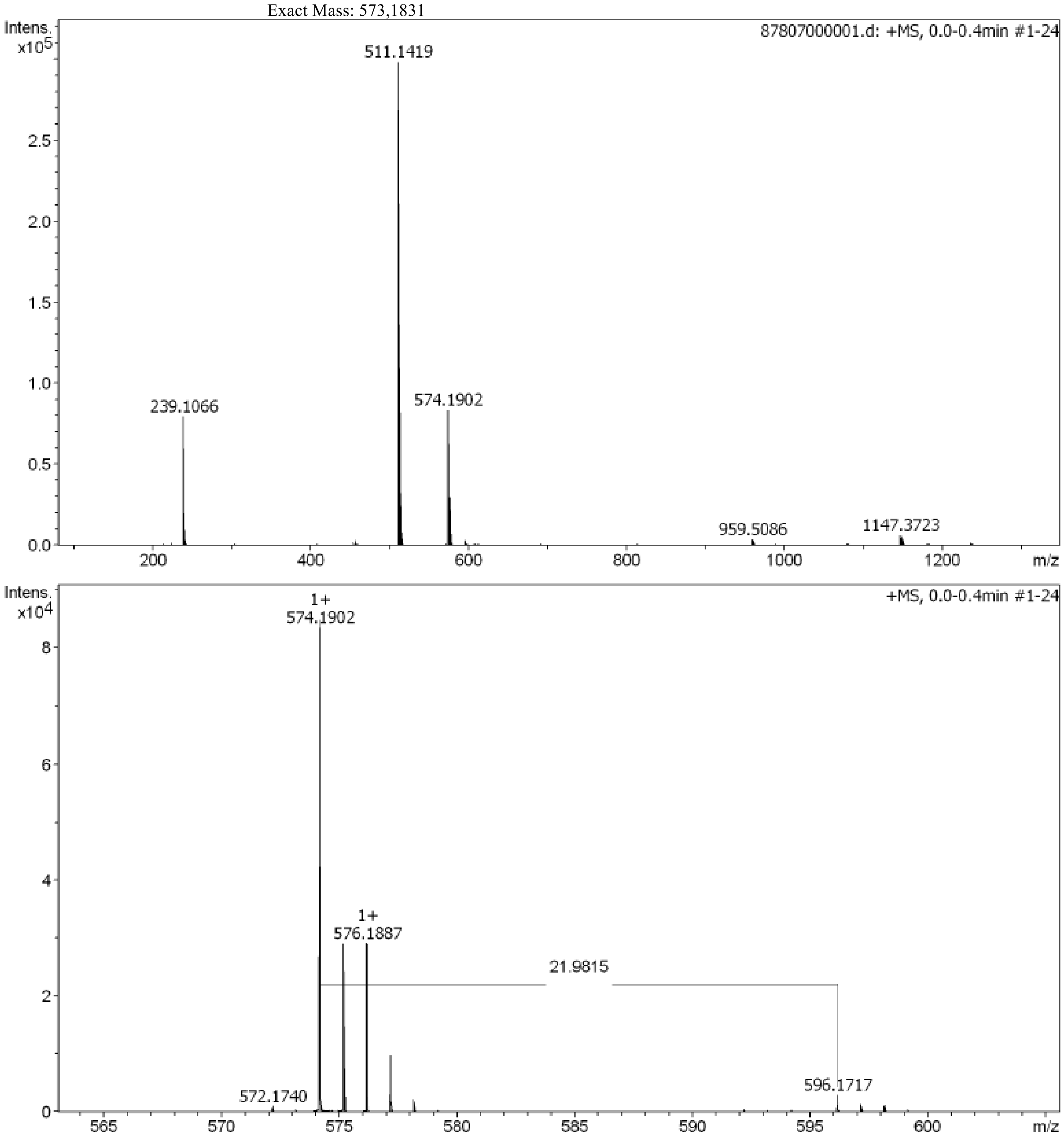

Figure S169: 2-Fluoroethyl (2-acetamidoethyl)(5-chloro-2-((3-cyanobenzyl)oxy)-4-((3-(2,3-dihydrobenzo[*b*][1,4]dioxin-6-yl)-2-methylbenzyl)oxy)benzyl)carbamate (**5j**):

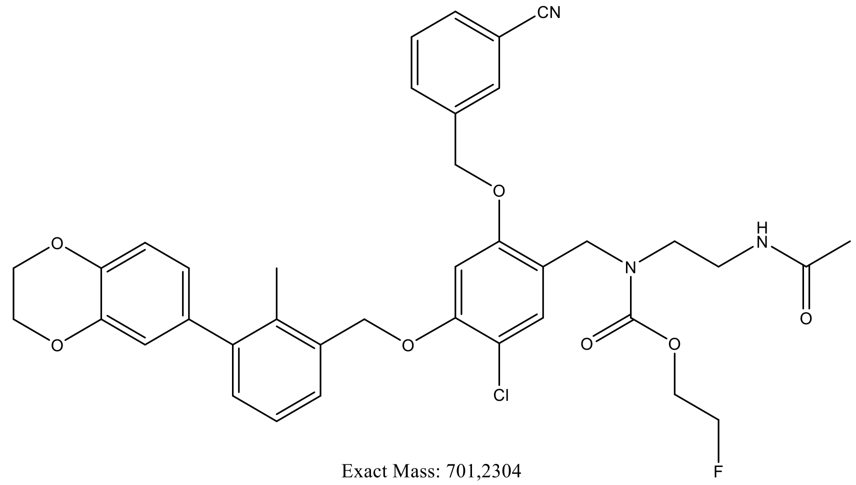

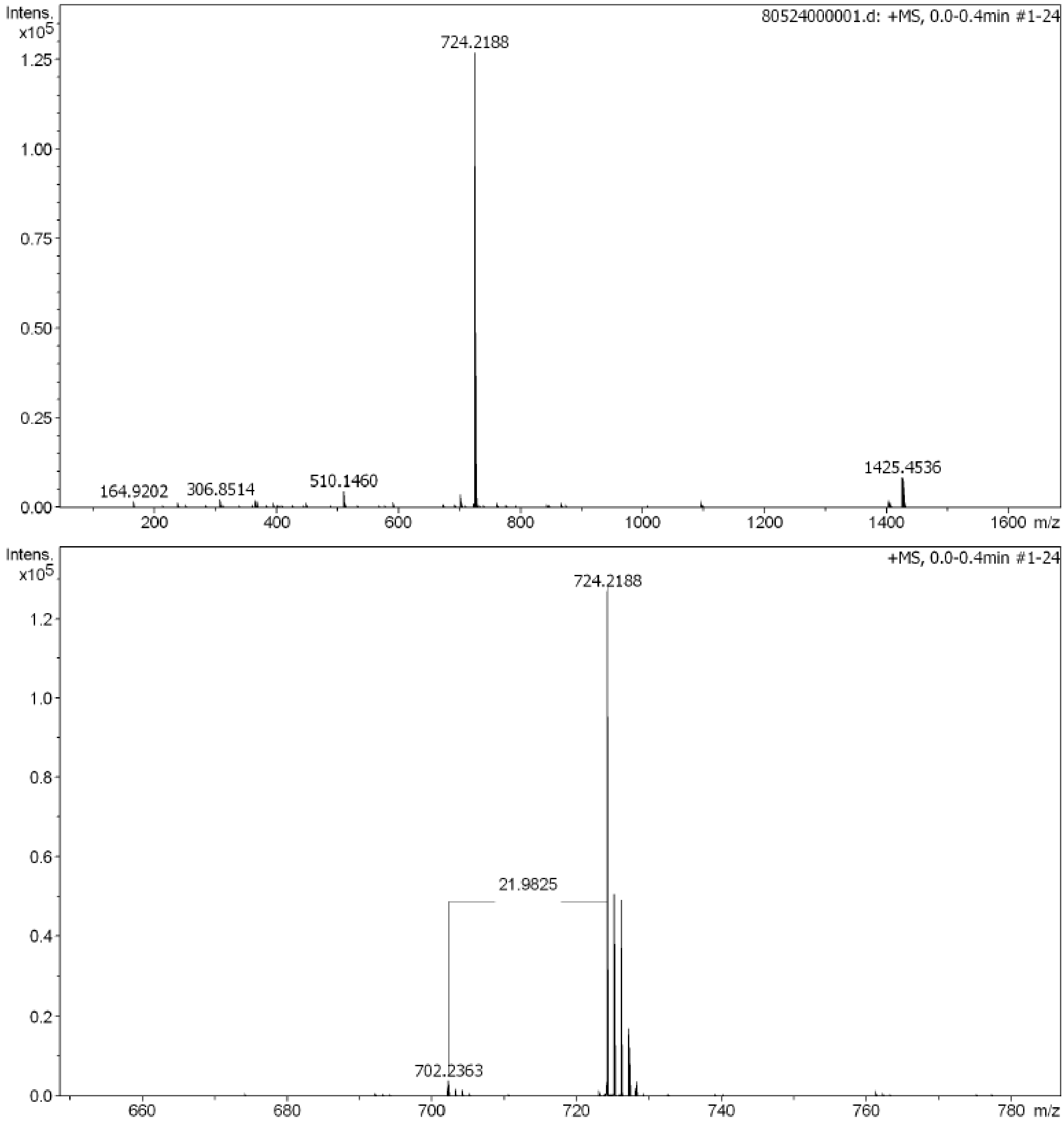

Supplement: Supplementary file 1 — jm3c02342_si_001.pdf [file jm3c02342_si_001.pdf]
